# Organocatalytic Asymmetric Peroxidation of γ,δ-Unsaturated β-Keto Esters—A Novel Route to Chiral Cycloperoxides

**DOI:** 10.3390/molecules28114317

**Published:** 2023-05-24

**Authors:** Mary C. Hennessy, Hirenkumar Gandhi, Timothy P. O’Sullivan

**Affiliations:** 1 School of Chemistry, University College Cork, T12 YN60 Cork, Ireland; maryhennessy@umail.ucc.ie (M.C.H.); 113220308@umail.ucc.ie (H.G.); 2 Analytical and Biological Chemistry Research Facility, University College Cork, T12 YN60 Cork, Ireland; 3 School of Pharmacy, University College Cork, T12 YN60 Cork, Ireland

**Keywords:** cycloperoxides, organocatalysis, δ-peroxy-β-keto esters, δ-hydroxy-β-keto esters, 1,2-dioxolanes

## Abstract

A methodology for the asymmetric peroxidation of γ,δ-unsaturated β-keto esters is presented. Using a cinchona-derived organocatalyst, the target δ-peroxy-β-keto esters were obtained in high enantiomeric ratios of up to 95:5. Additionally, these δ-peroxy esters can be readily reduced to chiral δ-hydroxy-β-keto esters without impacting the β-keto ester functionality. Importantly, this chemistry opens up a concise route to chiral 1,2-dioxolanes, a common motif in many bioactive natural products, via a novel P_2_O_5_-mediated cyclisation of the corresponding δ-peroxy-β-hydroxy esters.

## 1. Introduction

**Figure 1:**
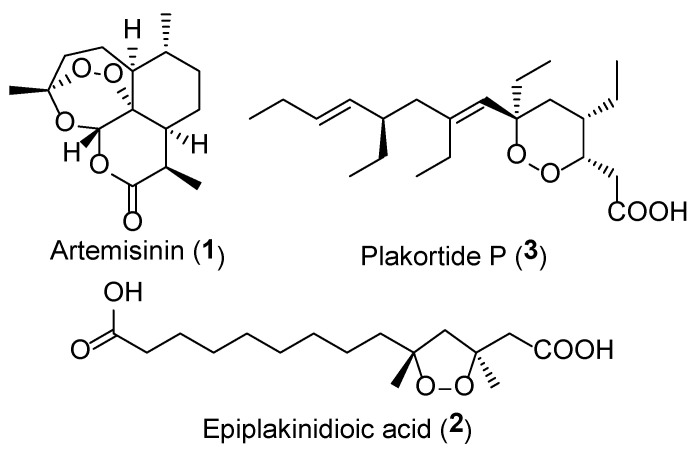
Bioactive peroxide-containing natural products.

Over the past several decades, hundreds of peroxide-containing natural products have been isolated and characterized [1,2,3,4,5]. A large number of these peroxy compounds were subsequently found to exhibit potent activity against a wide range of diseases that impact both human and animal health [6,7]. For example, artemisinin (**1**) and its semi-synthetic derivatives are highly effective against *Plasmodium falciparum*, the causative agent of malaria (Figure 1) [8,9]. Many simpler chiral peroxides are also biologically interesting. Examples include 1,2-dioxolanes such as epiplakinidioic acid (**2**), obtained from a marine sponge, which inhibits both prostate and melanoma cancer cell growth [10,11]. Similarly, plakortide P (**3**), a 1,2-dioxane derivative, exhibits potent anti-neuroinflammatory effects and disrupts TXB2 production at submicromolar levels [5]. Cycloperoxides such as **2** and **3** often incorporate stereogenic centers adjacent to a carboxyl group, and this motif represents an attractive target in organic synthesis.

**Scheme 1:**
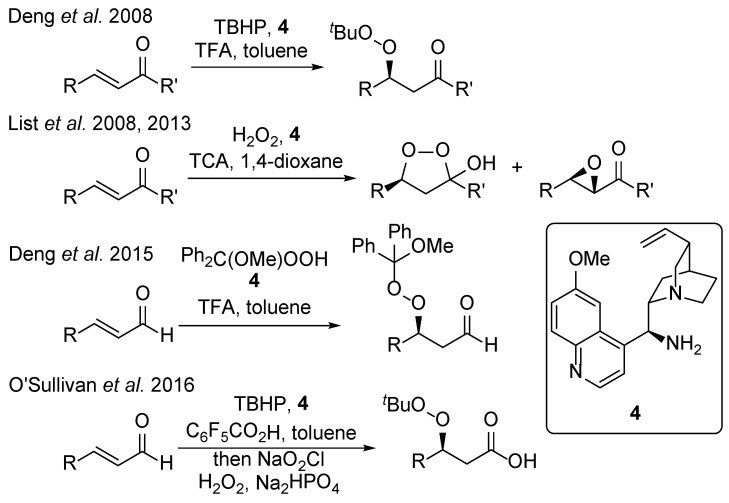
Previously reported organocatalytic peroxidations of unsaturated carbonyl compounds [28,29,30,31,32].

Despite their abundance in nature, methods for the enantioselective synthesis of chiral peroxides are still relatively scarce [12,13]. One approach with proven potential is the organocatalysed stereoselective peroxidation of unsaturated carbonyls. Asymmetric organocatalysis is an area of synthetic chemistry which is the focus of intense research, as evidenced by the many reviews on the topic since the early 2000s [14,15,16,17,18,19,20,21,22,23,24,25]. In particular, cinchona-derived organocatalysts have been successfully exploited by several groups for enantioselective peroxidations (Scheme 1) [26,27]. For example, List and Deng utilised a quinine-based catalyst for the asymmetric peroxidation of α,β-unsaturated ketones by the modification of a well-established epoxidation pathway utilising quinine-derived catalyst **4** [28,29,30]. Deng synthesised acyclic peroxides while List generated 1,2-dioxolanes and epoxides. More recently, Deng described the stereoselective peroxidation of α,β-unsaturated aldehydes [31]. In parallel work, our group investigated their asymmetric peroxidation with *tert*-butyl hydroperoxide (TBHP) followed by in situ Pinnick oxidation to afford stable β-peroxycarboxylic acids [32,33]. Outside of unsaturated ketone and aldehyde substrates, Russo and Lattanzi exploited a diaryl-2-pyrrolidinemethanol-derived catalyst to produce enantiomerically-enriched β-peroxynitroalkanes from nitrostyrenes [34]. The stereoselective peroxidation of unsaturated aromatic and aliphatic nitroalkenes has likewise been reported [35].

**Scheme 2:**
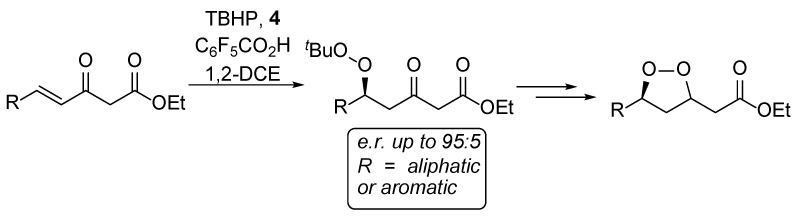
Current Work.

β-Keto esters constitute highly versatile building blocks in organic synthesis which can be readily modified through a wide variety of transformations [36,37,38,39,40,41,42]. To date, β-keto esters have been mostly employed as nucleophiles in organocatalysed reactions [43]. We are aware of only one report of a quinine-mediated addition of nitroalkanes to an α,β-unsaturated β-keto ester [44]. The main aim of this work is the development of a methodology for the asymmetric peroxidation of γ,δ-unsaturated β-keto esters (Scheme 2). This chemistry could open up a concise route to chiral 1,2-dioxolane-3-acetic acids, a recurring motif in several bioactive natural products.

## 2. Results and Discussion

Based on our prior work with α,β-unsaturated aldehydes, 9-amino cinchona derivative **4** was chosen as our catalyst along with pentafluorobenzoic acid as the co-catalyst. The choice of solvent is often a crucial aspect for achieving high enantioselectivities and yields in organocatalysed transformations. Accordingly, a solvent screen was conducted to identify the ideal reaction medium using **5** as our test substrate (Table 1). As the resulting chiral peroxides were inseparable by chiral chromatography, they were instead reduced to the corresponding δ-hydroxy-β-keto esters on reaction with zinc powder and acetic acid. This reduction strategy has been previously utilised for the analysis of β-peroxy ketones [28]. Reduction to more polar δ-hydroxy-β-keto esters affords products which are more amenable to resolution by chiral HPLC. A number of amylose and cellulose chiral columns were tested with the best results achieved with a Phenomenex AMY-2 column. An eluent system consisting of 5–15% isopropyl alcohol in hexane provided good separation. The stereochemical configuration of the major isomer was determined by comparison of the retention times with chiral δ-hydroxy-β-keto esters prepared by way of an asymmetric Mukaiyama aldol addition [45]. The (*R*)-enantiomer was found to predominate as previously observed with the peroxidation of α,β-unsaturated aldehydes and ketones [31,32]. Additionally, a negative optical rotation was recorded in each case, again supporting the formation of the (*R*)-isomer as the preferred product [45].

The least suitable solvent was acetonitrile, which resulted in a low yield accompanied by a poor e.r. of 69:31 (Table 1, entry 1). Much-improved enantiomeric ratios were recorded in tetrahydrofuran (entry 2), ethyl acetate (entry 3) and toluene (entry 4), with the latter affording a high e.r. of 95:5. This result is unsurprising, as Deng has previously demonstrated that toluene is the optimal medium for the peroxidation of unsaturated ketones and aldehydes [28,31]. However, for our substrates, these solvents were associated with moderate conversions and, consequently, yields in the range of 31%–42%. Switching to more polar solvents, such as ethanol, was no more successful (entry 5). Attempted peroxidation of **5** in ethanol instead saw the formation of a δ-ethoxy-β-keto ester as the major product in 19% yield, along with **6** in 13% yield. When this reaction was repeated in the absence of TBHP, the δ-ethoxy-β-keto ester was recovered as the sole product in 12% yield, suggesting that the solvent can add directly to the substrate, rather than displacing the δ-peroxy group. The use of water as a potential green solvent was equally disappointing (entry 6). By contrast, chlorinated solvents (entries 7–10) consistently afforded higher yields and enantiomeric ratios compared to non-chlorinated solvents (entries 1–6). 1,2-Dichloroethane afforded the best overall result with a 69% yield and a 93:7 e.r. (entry 8). Higher e.r. values were recorded with chloroform (entry 9) and carbon tetrachloride (entry 10), but at the cost of lower yields. The link between solvent choice and observed stereoselectivity is likely related to the ability of the solvent to influence the catalyst conformation [25]. Conformational investigations of the cinchona alkaloids, based on computational and spectroscopic techniques, have provided insight into how the reaction medium influences chiral induction and discrimination processes [46]. Cinchona catalysts can adopt multiple conformations and the catalytic capabilities of the cinchona scaffold are linked with its spatial arrangement [47]. Molecular mechanics calculations have demonstrated that the parent alkaloids preferentially adopt an *anti*-open conformation in non-polar solvents [48,49]. In polar solvents, the *syn*-closed and *anti*-closed conformations are strongly stabilised compared to the *anti*-open conformer [50]. The dielectric constant and the extent of hydrogen bonding can also influence the effectiveness of a catalyst in a given solvent [50].

The addition of an acid co-catalyst often has a beneficial effect on 9-amino cinchona-catalysed reactions [51]. In the specific case of asymmetric peroxidations, it has been postulated that the acid has a dual role, i.e., to firstly activate the carbonyl for condensation with the amine catalyst and to subsequently block intramolecular cyclisation and epoxide formation [51,52]. Consequently, a number of potential co-catalysts of varying acidity were examined for their impact on yields and stereoselectivity (Table 2). Although no strong relationship was apparent between the p*K*_a_ of the co-catalyst and reaction outcome, less acidic additives were generally associated with both poor conversions and reduced selectivity (entries 9–15). The highest e.r. of 96:4 was recorded in the presence of either heptafluorobutyric acid (entry 4) or trifluoroacetic acid (entry 5) with yields of 63% and 58%, respectively. Pentafluorobenzoic acid (entry 6) afforded a comparable e.r. of 93:7 but a higher yield of 69%, and this was considered a better compromise between stereoselectivity and yields. Changing to more acidic co-catalysts proved detrimental. For example, reactions in the presence of either *p*-toluenesulfonic acid (entry 2) or methanesulfonic acid (entry 3) returned high e.r. values along with poor conversions and yields. The use of highly acidic triflic acid negatively impacted both enantiomeric ratios and yields (entry 1). Finally, incorporation of a chiral center into the co-catalyst, which often imparts improved discrimination, was not successful on this occasion (entries 13–14) [53]. Attempted optimisation of the reaction by reducing the temperature afforded lower enantiomeric ratios and yields (entry 7). Surprisingly, raising the temperature to 50 °C did not affect enantioselectivity but did give rise to a poor yield of 21% (entry 8). Subsequent efforts to improve conversions (e.g., increasing the concentration or changing the catalyst/co-catalyst loadings) were ineffectual. Extending the reaction time beyond 96 h resulted in no significant improvement in conversions. In all cases, the peroxidation reactions proceeded cleanly, with no unidentified side products apparent. It is possible that keto:enol tautomerisation of the β-keto ester starting material is partly responsible for the less than complete conversions. On the other hand, the reactions furnished the target δ-peroxy esters exclusively, with no evidence for the formation of any epoxide side products as often observed with other α,β-unsaturated carbonyl compounds [28,30,54].

In order to investigate reaction scope, a library of linear, aliphatic γ,δ-unsaturated β-keto esters was initially prepared via the niobium chloride-catalysed C-H insertion of ethyl diazoacetate into the corresponding α,β-unsaturated aldehydes [55]. Additional substrates, including aromatic and branched aliphatic substrates, were synthesised using Wittig chemistry, several of which are novel (see page 5 of Supporting Information) [56]. In each case, the *E*-isomer was isolated exclusively in good to excellent yields, with no trace of the *Z*-isomer having formed. The resulting γ,δ-unsaturated β-keto esters were found to exist as a pair of keto-enol tautomers whose ratios could be determined by the integrations of the α-protons in the ^1^H-NMR spectra. Each of the substrates was subjected to our optimised peroxidation conditions and subsequently reduced to the corresponding δ-hydroxy ester (Table 3). High e.r.s over 90:10 were obtained from aliphatic esters across both non-branched (entries 1–5) and branched (entries 6–8) examples. In general, yields tended to decrease as chain length increased (entries 1–5) or with increased branching (entries 6–8). Simple aromatic substrates, incorporating either a phenyl (entry 9) or naphthyl ring (entry 10), were associated with a marked reduction in enantioselectivity. By contrast, peroxidation of a homobenzylic β-keto ester (entry 11) proceeded with an e.r of 92:8. We and others have previously noted how highly conjugated, unsaturated aldehydes and ketones are resistant to cinchona-catalysed peroxidation [28,32]. In that light, the differences in outcome observed between conjugated substrates **14–15** (entries 9–10) and non-conjugated **16** are not unexpected (entry 11). However, this trend did not hold up across all conjugated γ,δ-unsaturated β-keto esters, as the incorporation of a substituent onto the aromatic ring was found to greatly influence reaction outcomes (entries 12–23). In particular, the position of the substituent was found to be significant. *ortho*-Substituted substrates (entries 12–15) afforded the highest enantioselectivities with e.r.s comparable to those obtained with aliphatic esters. The stereoselectivity broadly decreased from *ortho*- (entries 12–15), to *meta*- (entries 16–18) and, finally, *para*-substituted analogues (entries 19–21). This outcome is most likely due to conformational effects, rather than to a direct inductive effect. No comparable pattern was apparent between the different series in terms of yields. Additionally, the choice of halogen substituent was unimportant. By contrast, aromatic esters bearing strongly electron-withdrawing groups (entry 22) or strongly electron-donating groups (entry 23) did behave differently, with the latter returning poorer e.r. values. Heteroatom-containing starting materials generally proved to be more challenging substrates (entries 24–26). Peroxidation of benzylether **29** proceeded in 26% yield and 82:18 e.r. (entry 24). The reactivity of 1,3-benzodioxole **30** was comparable and a similar outcome was observed in terms of isolated yields (entry 25). Unfortunately, we were unable to separate the enantiomers by chiral HPLC to determine the enantiomeric ratio. Furyl-substituted **31** proved to be an even less suitable substrate (entry 26) while δ-substituted substrate **32** failed to react (entry 27), a finding which mirrors that observed with other similar conjugated carbonyls [28,32]. Unlike their γ,δ-unsaturated β-keto ester precursors, the peroxide products existed primarily in the ketone form, with the exception of *para*-methoxyphenyl-substituted **54**, where a keto:enol ratio of 71:29 was noted. For most other peroxides, only trace amounts of the enol tautomer of the δ-peroxy-β-keto ester were present in the ^13^C-NMR spectra. All of the δ-peroxy compounds were readily reduced to their corresponding δ-hydroxy β-keto esters in good to excellent yields, apart from the naphthyl-substituted **67** (entry 10) and *ortho*-iodophenyl **72** (entry 15). No over-reduction of either the ester or ketone moieties was observed under the conditions employed, although some dehalogenation of *ortho*-iodophenyl **72** did occur, which likely accounts for the reduced yield (entry 15).

**Scheme 3:**
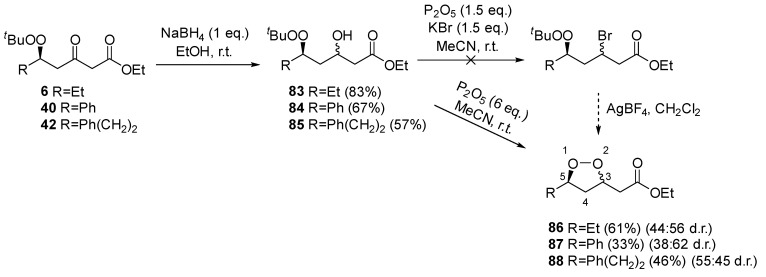
P_2_O_5_-mediated cyclisation of δ-peroxy-β-hydroxy esters.

The synthetic utility of these δ-peroxy-β-keto esters lies in their potential as precursors to 1,2-dioxolanes. We have previously demonstrated how *tert*-butylperoxyalkyl bromides can be transformed into the corresponding cycloperoxides on treatment with silver tetrafluoroborate [32,57]. Accordingly, **6** was first reduced with sodium borohydride to afford δ-peroxy-β-hydroxy ester **83** in 83% yield (Scheme 3). Attempted conversion of **83** to the corresponding δ-peroxy-β-bromo ester using the methodology of Khazdooz et al. did not proceed as planned [58]. Instead, an unexpected product was formed in <5% yield after 24 h which was subsequently identified as 1,2-dioxolane **86**. Repeating the reaction in the absence of potassium bromide saw the direct conversion of **83** to **86**, although starting material was still present by TLC analysis after 4 days. Increasing the loading of phosphorus pentoxide from two to six equivalents effected full conversion after 90 min, and **86** was recovered in 61% yield. The reaction time could be further shortened to 15 min on addition of 10 equivalents of phosphorus pentoxide, albeit with a lower yield of 43%.

**Figure 2:**
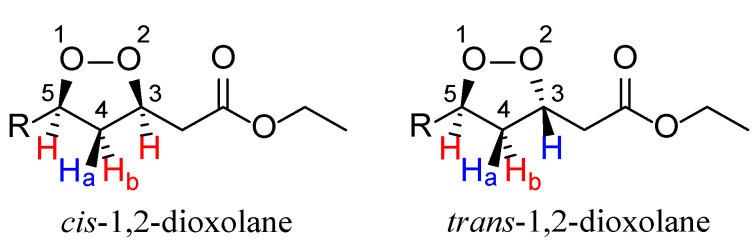
Comparison of *cis*- and *trans*-1,2 dioxolanes.

Evidence for the formation of 1,2-dioxolane **86** was provided by mass spectrometry and NMR spectroscopy. An ion of mass 211.0934 was detected by HRMS, which corresponds to the sodium adduct of **86**. Additionally, the chemical shifts of the C-3 peaks at 81.9 ppm and 82.5 ppm and the C-4 peaks at 44.9 ppm and 45.4 ppm are similar to those reported for comparable dioxolanes in the literature [59,60,61,62]. The *cis*- and *trans*-isomers may be differentiated by the distinct chemical shifts of the H-4 protons which appear at 1.89 ppm and 2.88 ppm in *cis*-**86** due to their differing chemical environments (Figure 2). Additionally, geminal coupling of H-4, and further coupling to H-3 and H-5, gives rise to a characteristic ddd splitting pattern. By contrast, in the case of *trans*-**86**, the H-4 protons have a similar chemical shift of 2.41 ppm and result in a simpler dd splitting pattern. Based on the integrations of H-4 in the ^1^H-NMR spectrum of **86**, the *cis*- and *trans*-1,2-dioxolanes were generated in a ratio of 44:56. δ-Peroxy-β-hydroxy esters **84** and **85** were also subjected to these conditions, and formation of the cycloperoxide products was again found to be successful. Cyclisation of phenyl-substituted **84** proceeded in a reduced yield of 33%, whereas the presence of the –CH_2_CH_2_– linker in **85** resulted in a slightly improved yield of 46%.

**Figure 3:**
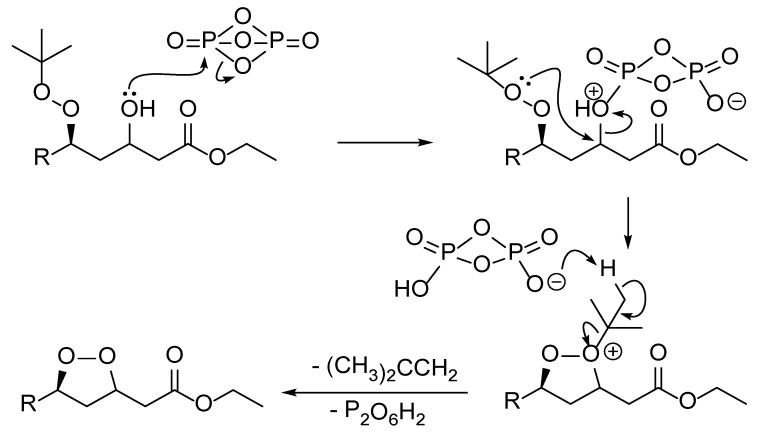
Proposed cyclisation mechanism.

Given the Lewis acidic nature of phosphorus pentoxide, it is likely that the reaction proceeds in a manner similar to that of our previously established silver-mediated cyclisation of *tert*-butylperoxyalkyl bromides, i.e., by trapping of the oxygen lone pair by the incipient carbocation, resulting in loss of the *tert*-butyl group and formation of the target cycloperoxide (Figure 3) [18,40]. In a similar vein, Khazaei and colleagues have previously demonstrated the use of phosphorus pentoxide for the etherification of benzylic alcohols [63].

## 3. Materials and Methods

**Synthesis of (4-ethoxy-2,4-dioxobutyl)triphenylphosphonium chloride**: Ethyl 4-chloroacetoacetate (6.30 g, 38.2 mmol, 1.0 eq.) and triphenylphosphine (10.00 g, 38.2 mmol, 1.0 eq.) were dissolved in toluene and stirred at 50 °C for 24 h. Solvent was removed under reduced pressure to afford 15.49 g, 36.3 mmol (4-ethoxy-2,4-dioxobutyl)triphenylphosphonium chloride as a pale yellow solid in a 95% yield without the need for purification [64].

**Wittig olefination:** (4-Ethoxy-2,4-dioxobutyl)triphenylphosphonium chloride (2.56 g, 6.0 mmol, 1.2 eq.) was dissolved in anhydrous THF (20 mL) at 0 °C and sodium hydride (360 mg, 15.0 mmol, 3.0 eq.) was added and stirred for 30 min. The aldehyde (5.0 mmol, 1.0 eq.) was added dropwise, and the reaction was allowed to come to room temperature and stirred for 5 h. The reaction was quenched with dropwise addition of water (10 mL) and extracted with ethyl acetate (30 mL) and saturated ammonium chloride solution (30 mL). The organic layer was dried with magnesium sulfate, concentrated in vacuo and the residue was subjected to silica gel column chromatography with diethyl ether:hexane in gradient ratios (2–20%) to afford the target γ,δ-unsaturated β-ketoester [56].

**Synthesis of (8*S*,9*S*)-9-amino-(9-deoxy)epiquinine (4):** Quinine (2.00 g, 6.2 mmol, 1.0 eq.) and triphenylphosphine (1.93 g, 7.4 mmol, 1.2 eq.) were dissolved in 50 mL of THF at 0 °C under nitrogen. Diisopropyl azodicarboxylate (DIAD) (1.45 mL, 7.4 mmol, 1.2 eq.) was added dropwise via syringe giving a clear solution. After 5 min, diphenylphosphoryl azide (DPPA) (1.74 mL, 8.0 mmol, 1.2 eq.) was added dropwise via syringe, turning the reaction mixture yellow. The reaction was stirred overnight at room temperature under nitrogen. The temperature was increased to 50 °C for 2 h. Additional triphenylphosphine (2.10 g, 8.0 mmol, 1.4 eq.) was added and the reaction temperature was maintained at 50 °C until gas formation ceased (approximately 2 h). The reaction was allowed to cool to room temperature. Deionised water (2.00 mL) was added, and the reaction mixture was stirred overnight. The reaction mixture was concentrated in vacuo, and the residue was partitioned between dichloromethane (100 mL) and 2N HCl (100 mL). Following vigorous shaking, the aqueous phase was separated and washed with dichloromethane (3 × 20 mL). The aqueous phase was concentrated under reduced pressure. The crude product was recrystallized by dissolving in the minimum amount of methanol and adding ethyl acetate as an anti-solvent until the opalescence became persistent. The salt was basified with excess sodium carbonate and extracted with dichloromethane (3 × 20 mL). The organic layers were combined, dried with magnesium sulfate and reduced in vacuo to give 1.22 g of a sticky brown oil in a 62% yield [65,66].

**Enantioselective peroxidation:** γ,δ-Unsaturated β-ketoester (3.5 mmol, 1 eq.), pentafluorobenzoic acid (149 mg, 0.7 mmol, 0.2 eq.), quinine-derived catalyst **4** (113 mg, 0.35 mmol, 0.1 eq.) and *tert*-butyl hydroperoxide (1.15 mL of a 5.5 M solution in decane, 6.3 mmol, 1.8 eq.) were added to 1,2-dichloroethane (5 mL) and the mixture was stirred at room temperature for 96 h. The reaction mixture was then filtered through a silica gel pad and washed with diethyl ether (40 mL), and the solvent was removed in vacuo. The resulting residue was subjected to silica gel column chromatography with diethyl ether:hexane as the eluent in gradient ratios (1–10%) to afford the target peroxide.

**Selective peroxide reduction**: The appropriate δ-peroxy β-keto ester (0.2 mmol, 1 eq.) and zinc powder (171 mg, 2.8 mmol, 14 eq.) were added to a 50% aqueous solution of acetic acid (5 mL) and stirred at room temperature until the peroxide was fully consumed, as confirmed by TLC (7 h). The reaction was quenched with saturated sodium bicarbonate solution (15 mL). Ethyl acetate (20 mL) was added, and the organic layer was separated. The aqueous layer was extracted with ethyl acetate (2 × 15 mL). The combined organic layers were washed with brine (30 mL) and dried with magnesium sulfate before the solvent was removed in vacuo. The resulting residue was subjected to silica gel column chromatography with diethyl ether:hexane as eluent in gradient ratios (20–50%) to afford the target δ-hydroxy β-keto ester.

**HPLC analysis:** The enantiopurity of chiral δ-hydroxy β-keto esters was determined by chiral stationary phase high-performance liquid chromatography (HPLC). The stereoisomers were separated using a Phenomenex AMY-2 column and an eluent system consisting of 5–15% isopropyl alcohol in hexane; the exact conditions are outlined in the Supporting Information. HPLC analysis was performed on a Waters alliance 2695 separations module equipped with a Waters 2996 photodiode array detector. All chiral stationary phase HPLC analysis was carried out at ambient temperature unless otherwise stated.

**Selective ketone reduction:** δ-Peroxy β-keto ester (0.4 mmol, 1 eq.) and sodium borohydride (15 mg, 0.4 mmol, 1 eq.) were added to ethanol (5 mL) and stirred at room temperature until the starting material was fully consumed, as confirmed by TLC (approximately 2 h). The reaction mixture was passed through a pad of silica gel and washed with diethyl ether (15 mL), and the solvent was removed in vacuo. The resulting residue was subjected to silica gel column chromatography with diethyl ether: hexane as eluent in gradient ratios (5–25%).

**P_2_O_5_-mediated cyclisation:** δ-Peroxy β-hydroxy ester (0.4 mmol, 1 eq.) was dissolved in acetonitrile (5 mL), and phosphorus pentoxide (342 mg, 2.4 mmol, 6 eq.) was added. The reaction was stirred at room temperature until the δ-peroxy β-hydroxy ester was consumed, as confirmed by TLC (90 min). The reaction mixture was then filtered through a silica gel pad and washed with diethyl ether (20 mL), and the solvent was removed in vacuo. The resulting residue was subjected to silica gel column chromatography with diethyl ether: hexane as the eluent in gradient ratios (5–25%).

## 4. Conclusions

To date, literature methods for the synthesis of chiral peroxides remain relatively rare. In this article, we have demonstrated the use of a quinine-derived organocatalyst for the asymmetric peroxidation of a range of γ,δ-unsaturated β-keto esters. The resulting δ-peroxy-β-keto esters can be obtained in good to excellent enantiomeric ratios and in moderate to good yields. Both aliphatic and aromatic substrates are amenable to enantioselective peroxidation, with higher selectivity observed with substituted aromatic rings. This peroxidation methodology may also be applicable to other substrates, such as unsaturated α-keto esters [67]. Furthermore, the use of alternative cinchona-based catalysts may help in improving conversions [68]. The subsequent selective reduction of the δ-peroxy-β-keto ester products to their corresponding chiral δ-hydroxy-β-keto esters proceeds in high yields, without affecting the important β-keto ester functionality. We have also discovered a novel phosphorus pentoxide-mediated cyclisation of δ-peroxy-β-hydroxy esters to chiral 1,2-dioxolanes. Alkyl-, aryl- and homobenzyl-substituted δ-peroxy-β-hydroxy esters were successfully cyclised under optimised conditions. We surmise that reaction proceeds by trapping of the peroxide oxygen lone pair, resulting in loss of the *tert*-butyl group and formation of the target cycloperoxide. This chemistry provides a concise route to chiral 1,2-dioxolane-3-acetic acids which are incorporated into a wide range of bioactive natural products.

## Figures and Tables

**Table 1:** Effect of solvent on yield and enantioselectivity.

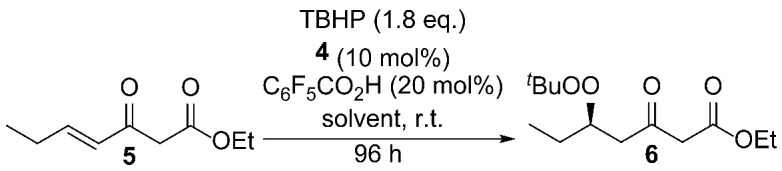				
Entry	Solvent	Conversion ^[a]^	Isolated Yield	e.r.
1	MeCN	40%	26%	69:31
2	THF	50%	31%	92:8
3	EtOAc	64%	33%	92:8
4	Tol	73%	42%	95:5
5	EtOH	n.d. ^[b]^	13% (19%) ^[c]^	79:21
6	H_2_O	54%	45%	88:12
7	CH_2_Cl_2_	75%	52%	93:7
8	1,2-DCE	87%	69%	93:7
9	CHCl_3_	73%	49%	96:4
10	CCl_4_	61%	42%	94.5:5.5

^[a]^ Conversion determined by comparison of α-proton integrals in starting material vs. product in the ^1^H-NMR spectrum. ^[b]^ Not determined due to overlapping ^1^H-NMR signals. ^[c]^ Yield of δ-ethoxy-β-keto ester.

**Table 2:** Effect of co-catalyst on yield and enantioselectivity.

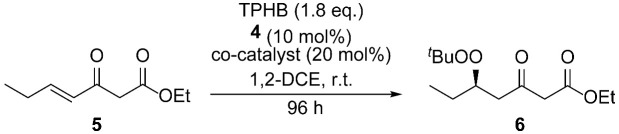						
Entry	Co-Catalyst	p*K*_a_	Temp.	Conversion ^[a]^	Isolated Yield	e.r.
1	Triflic acid	−14.7	r.t.	60%	41%	57:43
2	*p*-Toluenesulfonic acid	−2.8	r.t.	15%	10%	94:6
3	Methanesulfonic acid	−1.9	r.t.	23%	18%	95.5:4.5
4	Heptafluorobutyric acid	0.4	r.t.	84%	63%	96:4
5	Trifluoroacetic acid	0.5	r.t.	77%	58%	96:4
6	Pentafluorobenzoic acid	1.5	r.t.	87%	69%	93:7
7	Pentafluorobenzoic acid	1.5	4 °C	76%	54%	89:11
8	Pentafluorobenzoic acid	1.5	50 °C	32%	21%	94:6
9	Chloroacetic acid	2.9	r.t.	25%	17%	86.5:13.5
10	Tartaric acid	2.9	r.t.	4%	n.d.	n.d.
11	2,4-Bis(trifluoromethyl)benzoic acid	3.3	r.t.	42%	29%	86:14
12	4-(Trifluoromethyl)benzoic acid	3.6	r.t.	22%	18%	81:19
13	Boc-*L*-phenylglycine	3.9	r.t.	22%	18%	88:12
14	Boc-*D*-phenylglycine	3.9	r.t.	25%	20%	87:13
15	Benzoic acid	4.2	r.t.	8%	n.d.	n.d.

^[a]^ Conversion determined by comparison of α-proton integrals in starting material vs. product in the ^1^H-NMR spectrum.

**Table 3:** Peroxidation of γ,δ-unsaturated β-keto esters with TBHP and subsequent reduction with zinc and acetic acid.

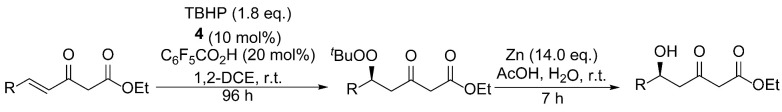										
Entry	R	β-Keto Ester	Conversion ^[a]^	Peroxide	Isolated Yield	e.r.	MW (g/mol)	Alcohol	Isolated Yield	MW (g/mol)
1	CH_3_	**7**	86%	**33**	79%	91:9	246.30	**58**	87%	174.20
2	CH_3_CH_2_	**5**	87%	**6**	69%	93:7	260.33	**59**	71%	188.22
3	CH_3_(CH_2_)_2_	**8**	74%	**34**	52%	93:7	274.36	**60**	77%	202.25
4	CH_3_(CH_2_)_3_	**9**	46%	**35**	34%	95:5	288.38	**61**	93%	216.28
5	CH_3_(CH_2_)_4_	**10**	50%	**36**	43%	95:5	302.41	**62**	93%	230.30
6	(CH_3_)_2_CH	**11**	n.d.	**37**	27%	90:10	274.36	**63**	93%	202.25
7	(CH_3_)_2_CHCH_2_	**12**	33%	**38**	30%	92:8	288.39	**64**	97%	216.28
8	Cy	**13**	42%	**39**	35%	92:8	314.42	**65**	91%	242.32
9	C_6_H_5_	**14**	51%	**40**	49%	74:26	308.37	**66**	83%	236.27
10	2-Naphthyl	**15**	24%	**41**	17%	67:33	358.43	**67**	57%	286.33
11	C_6_H_5_CH_2_CH_2_	**16**	54%	**42**	47%	92:8	336.43	**68**	74%	264.32
12	2-FC_6_H_4_	**17**	25%	**43**	22%	83:17	326.36	**69**	80%	254.26
13	2-ClC_6_H_4_	**18**	29%	**44**	25%	91:9	342.82	**70**	84%	270.71
14	2-BrC_6_H_4_	**19**	32%	**45**	29%	92:8	387.27	**71**	97%	315.16
15	2-IC_6_H_4_	**20**	29%	**46**	20%	93:7	434.27	**72**	34%	362.16
16	3-FC_6_H_4_	**21**	37%	**47**	26%	78:22	326.36	**73**	89%	254.26
17	3-ClC_6_H_4_	**22**	42%	**48**	17%	78:22	342.82	**74**	86%	270.71
18	3-BrC_6_H_4_	**23**	35%	**49**	28%	80:20	387.27	**75**	83%	315.16
19	4-FC_6_H_4_	**24**	29%	**50**	28%	73:27	326.36	**76**	83%	254.26
20	4-ClC_6_H_4_	**25**	26%	**51**	24%	76:24	342.82	**77**	80%	270.71
21	4-BrC_6_H_4_	**26**	34%	**52**	18%	78:22	387.27	**78**	90%	315.16
22	4-CF_3_C_6_H_4_	**27**	38%	**53**	31%	67:33	376.37	**79**	94%	304.27
23	4-MeOC_6_H_4_	**28**	43%	**54**	38%	57:43	338.40	**80**	74%	266.30
24	BnOCH_2_	**29**	27%	**55**	26%	82:18	352.43	**81**	61%	280.32
25	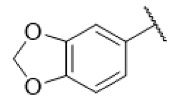	**30**	25%	**56**	22%	n.d ^[b]^	352.38	**82**	94%	280.28
26	2-Furyl	**31**	7%	**57**	6%	n.d ^[b]^	298.34	-	-	-
27	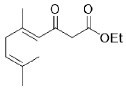	**32**	0%	-	0%	-	-	-	-	-

^[a]^ Conversion determined by comparison of α-proton integrals in starting material vs. product in the ^1^H-NMR spectrum. ^[b]^ Not separable by chiral HPLC.

## Data Availability

The data presented in this study are available in the Supplementary Materials.

## Supplementary Materials

The following supporting information can be downloaded at: https://www.mdpi.com/article/10.3390/molecules28114317/s1, Full experimental details, including compound characterisation and associated spectra, are available in the Supporting Information.
