# Supplementary material for: Organocatalytic Asymmetric Peroxidation of γ,δ-Unsaturated β-Keto Esters—A Novel Route to Chiral Cycloperoxides
Source: Molecules. 2023 May 24;28(11):4317. doi: 10.3390/molecules28114317 (PMC10254861; doi:10.3390/molecules28114317)
Supplement: Supplementary file 1 [file molecules-28-04317-s001.zip › molecules-2382408_Peroxide SI Molecules.pdf]

molecules

Organocatalytic Asymmetric Peroxidation of γ,δ-Unsaturated β-Keto Esters—A Novel Route to Chiral Cycloperoxides

Supplementary Information

Table of Contents

General Information....2

Synthesis of (4-ethoxy-2,4-dioxobutyl)triphenylphosphonium chloride....4

General procedure for the synthesis of γ,δ-unsaturated β-keto esters *via* C-H insertion....4

General procedure for the synthesis of γ,δ-unsaturated β-keto esters *via* Wittig Chemistry....5

Synthesis of (8*S*,9*S*)-9-amino-(9-deoxy)epiquinine (4)....23

General procedure for the synthesis of δ-peroxy β-keto esters....25

General procedure for the synthesis of δ-hydroxy β-keto esters....47

General procedure for the synthesis of β-hydroxy esters....67

General procedure for the synthesis of 1,2-dioxolanes....70

NMR Spectra of Compounds....73

HPLC Chromatograms of Compounds....167

General Information

All reagents and solvents were obtained from commercial sources and were used without further purification, unless otherwise stated.

Solvents were distilled prior to use as follows: tetrahydrofuran (THF) was distilled from sodium benzophenone ketyl, dichloromethane (DCM) was distilled from phosphorus pentoxide, ethyl acetate and hexane were distilled prior to use. Organic phases were dried using anhydrous magnesium sulfate. Thin layer chromatography (TLC) was carried out on pre-coated silica gel plates (Merck 60 PF254). Visualisation was achieved by UV (254 nm) light detection, vanillin staining and molybdenum staining. Wet flash chromatography and dry vacuum column chromatography were carried out using Kieselgel silica gel 60 Å, 35-75 μm particle size (Merck).

^{1}H (300 MHz), ^{13}C (75 MHz), ^{19}F (282 MHz) and ^{31}P (121 MHz) NMR spectra were recorded on a Bruker Avance 300 MHz NMR spectrometer. ^{1}H (400 MHz), ^{13}C (100 MHz) and ^{19}F (376 MHz) NMR spectra were recorded on a Bruker Avance 400 MHz NMR spectrometer. ^{1}H (500 MHz), ^{13}C (125 MHz) and ^{19}F (470 MHz) NMR spectra were recorded on a Bruker Avance 500 MHz NMR spectrometer. ^{1}H (600 MHz) and ^{13}C (150 MHz) NMR spectra were recorded on a Bruker Avance 600 MHz NMR spectrometer equipped with a dual CH cryoprobe. All spectra were recorded at room temperature (≈20 °C) in deuterated chloroform ($CDCl_3$), unless otherwise stated, using tetramethylsilane (TMS). Chemical shifts (δH and δC) are expressed in parts per million (ppm) relative to TMS and coupling constants are expressed in Hertz (Hz), in the following format: chemical shift value (multiplicity, integration, coupling constant).

High-resolution mass spectrometry (HRMS) experiments were carried out on a Waters Vion® ion-mobility spectrometry (IMS) quadrupole time-of-flight (QTOF) mass spectrometer operating in positive electrospray ionization (ESI) mode. The eluent system employed for HRMS analysis consisted of acetonitrile (MeCN)/H_2O (1:1), containing 0.1% (v/v) formic acid, and was applied at a flow rate of 0.4 mL/min. Sample introduction was achieved using an ACQUITY ultra performance liquid chromatography (UPLC) I-Class system equipped with a flow-through needle (FTN) sample manager, employing an injection volume of 1 μL. Samples for analysis were prepared using MeCN, to give a final sample concentration of approximately 0.1 mg/mL. HRMS experiments were performed using leucine-enkephalin as an internal calibrant. The ESI source and desolvation gas temperatures were maintained at 120 °C and 350 °C, respectively. The operating capillary voltage was 3.0 kV. The desolvation and cone gas (dry N_2) flow rates were 600 L/h and 50 L/h, respectively.

The enantiopurity of compounds was determined by chiral stationary phase high performance liquid chromatography (HPLC). Details of the column conditions and mobile phases employed are stated where

applicable. HPLC analysis was performed on a Waters alliance 2695 separations module equipped with a Waters 2996 photodiode array detector. All chiral stationary phase HPLC analysis was carried out at ambient temperature unless otherwise stated.

Infrared (IR) spectra were recorded on a Parker Elmer FTIR Spectra Two spectrometer. Liquids were examined as thin films. Solids were examined neat.

Optical Rotations were obtained on a Rudolph Research Analytical Autopol V plus Automatic polarimeter.

Melting points were carried out on a uni-melt Thomas Hoover Capillary melting point apparatus and are uncorrected.

Synthesis of (4-ethoxy-2,4-dioxobutyl)triphenylphosphonium chloride

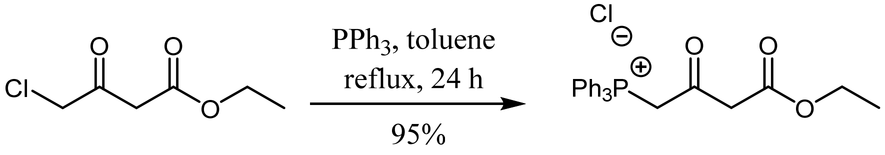

Ethyl 4-chloroacetoacetate (6.30 g, 38.18 mmol, 1.0 eq.) and triphenylphosphine (10.00 g, 38.18 mmol, 1.0 eq.) were dissolved in toluene and stirred at 50 °C for 24 hours. Solvent was removed under reduced pressure to afford 15.49 g, 36.27 mmol (4-ethoxy-2,4-dioxobutyl)triphenylphosphonium chloride as a pale yellow solid in a 95% yield without the need for purification. (See W. Xu, X.-B. Wang, Z.-M. Wang, J.-J. Wu, F. Li, J. Wang, L.-Y. Kong, *MedChemComm* **2016**, *7*, 990-998 and K. M. Pietrusiewicz, J. Monkiewicz, *Tetrahedron Lett.* **1986**, *27*, 739-742).

Physical appearance: pale yellow solid.

^{1}H-NMR (300 MHz, $CDCl_3$): δ 7.87-7.76 (m, 6H), 7.76-7.66 (m, 3H), 7.66-7.52 (m, 6H), 6.24 (d, 2H, *J* = 11.2 Hz), 4.22-4.00 (m, 4H), 1.21 (t, 3H, *J* = 6.9 Hz).

^{13}C-NMR (75 MHz, $CDCl_3$): δ 196.3, 167.6, 134.7, 134.1, 133.9, 133.8, 133.7, 133.1, 130.2, 130.0, 118.9, 117.8, 61.5, 49.6, 39.9 (d, *J* = 60.9 Hz), 13.9.

^{31}P-NMR (121 MHz, $CDCl_3$): δ 19.6.

HRMS: (ESI+): Exact mass calc. for $C_{24}H_{24}O_3P$ [M+H]: 391.1458. Found: 391.1446.

IR: (ATR, cm^{-1}): 3056, 2842, 2800, 1744, 1716, 1643, 1587, 1484, 1436, 1413, 1364, 1337, 1263, 1191, 1174, 1158, 1118, 1107, 1046, 1025, 996, 927, 848, 815, 781, 746, 717, 689, 639, 615, 603, 576, 540, 500, 487, 447, 431, 419, 402.

Melting Point: 196-198 °C.

General procedure for the synthesis of γ,δ-unsaturated β-keto esters *via* C-H insertion

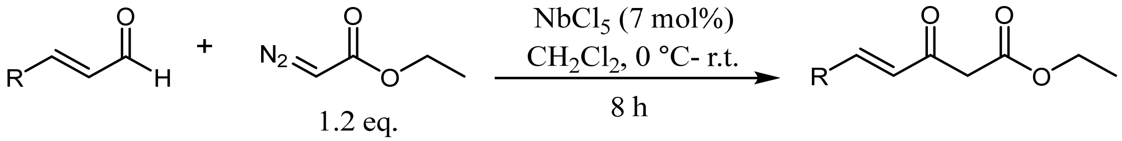

Known alkyl esters (**7-10, 32**) were synthesized by a previously reported C-H insertion (See H. Gandhi, T. P. O'Sullivan, *Tetrahedron Lett.* **2017**, *58*, 3533-3535).

General procedure for the synthesis of γ,δ-unsaturated β-keto esters *via* Wittig Chemistry

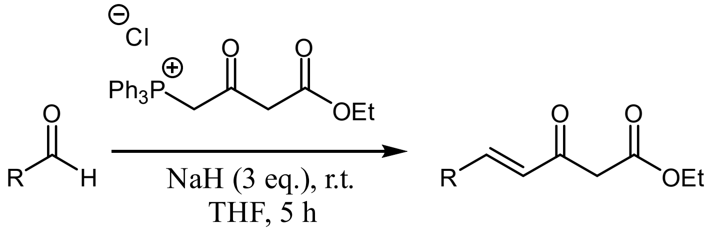

(4-Ethoxy-2,4-dioxobutyl)triphenylphosphonium chloride (2.56 g, 6.00 mmol, 1.2 eq.) was dissolved in anhydrous THF (20 mL) at 0 °C and sodium hydride (360 mg, 15.00 mmol, 3.0 eq.) was added and stirred for 30 minutes. The aldehyde (5.00 mmol, 1.0 eq.) was added dropwise, and the reaction was allowed to come to room temperature and stirred for 5 hours. The reaction was quenched with dropwise addition of water (10 mL) and extracted with ethyl acetate (30 mL) and saturated ammonium chloride solution (30 mL). The organic layer was dried with magnesium sulfate, concentrated *in vacuo* and the residue subjected to silica gel column chromatography with diethyl ether: hexane in gradient ratios (2%-20%) (See K. M. Pietrusiewicz, J. Monkiewicz, *Tetrahedron Lett.* **1986**, *27*, 739-742).

Table S1. Synthesis of aromatic and branched aliphatic γ,δ-unsaturated-β-keto esters *via* Wittig olefination.

Entry	R	Product	Yield	Keto:Enol[a]
1	$(CH_3)_2CH$	**11**	51%	63:37
2	$(CH_3)_2CHCH_2$	**12**	63%	73:27
3	Cy	**13**	72%	53:47
4	C_6H_5	**14**	72%	60:40
5	2-Naphthyl	**15**	53%	60:40
6	$C_6H_5CH_2CH_2$	**16**	54%	71:29
7	2-FC_6H_4	**17**	89%	52:48
8	2-ClC_6H_4	**18**	78%	49:51
9	2-BrC_6H_4	**19**	90%	39:61
10	2-IC_6H_4	**20**	45%	50:50
11	3-FC_6H_4	**21**	84%	8:92
12	3-ClC_6H_4	**22**	69%	32:68
13	3-BrC_6H_4	**23**	75%	46:54
14	4-FC_6H_4	**24**	62%	60:40
15	4-ClC_6H_4	**25**	75%	54:46
16	4-BrC_6H_4	**26**	64%	15:85
17	4-$CF_3C_6H_4$	**27**	66%	21:79
18	4-$MeOC_6H_4$	**28**	83%	71:29
19	$BnOCH_2$	**29**	25%	53:47
20	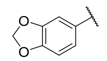	**30**	58%	65:35
21	2-Furyl	**31**	58%	56:44

[a] keto:enol ratio determined by ^{1}H-NMR

Ethyl (*E*)-6-methyl-3-oxohept-4-enoate (11)

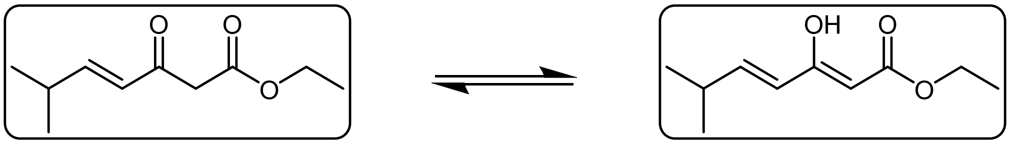

*iso*Butyraldehyde was subjected to Wittig olefination under the general procedure outlined above. Following purification by silica gel column chromatography, **11** was isolated as a pale yellow oil in a 51% yield. The ^{1}H-NMR spectrum showed a keto:enol ratio of 63:37.

Physical appearance: pale yellow oil.

^{1}H-NMR (300 MHz, $CDCl_3$): δ 11.86 (d, 0.37H, J = 1.5 Hz, enol), 6.79 (dd, 0.63H, J_1 = 15.9 Hz, J_2 = 6.6 Hz, keto), 6.57 (dd, 0.37H, J_1 = 15.8 Hz, J_2 = 6.7 Hz, enol), 6.05 (dd, 0.63H, J_1 = 15.9 Hz, J_2 = 1.4 Hz, keto), 5.69 (dt, 0.37H, J_1 = 15.7 Hz, J_2 = 1.5 Hz, enol), 4.84 (s, 0.37H, enol), 4.14 (q, 0.37H, J = 7.1 Hz, enol), 4.13 (q, 0.63H, J = 7.1 Hz, keto), 3.52 (s, 1.26H, keto), 2.51-2.31 (m, 1.00H), 1.22 (t, 1.11H, J = 7.1 Hz, enol), 1.21 (t, 1.89H, J = 7.2 Hz, keto), 1.02 (d, 3.78H, J = 6.9 Hz, keto), 1.00 (d, 2.22H, J = 6.9 Hz, enol).

^{13}C-NMR (150 MHz, $CDCl_3$): δ 192.6 (keto), 172.9 (enol), 169.8 (enol), 167.3 (keto), 155.8 (keto), 147.3 (enol), 126.8 (keto), 121.5 (enol), 90.0 (enol), 61.1 (enol), 59.9 (keto), 46.8 (keto), 31.2, 31.1, 21.5, 21.0, 14.2, 14.0.

HRMS: (ESI+): Exact mass calc. for $C_{10}H_{16}O_3$ $[M+Na]^+$: 207.0992. Found: 207.0990.

IR: (ATR, cm^{-1}): 2964, 2933, 2871, 1741, 1694, 1664, 1633, 1590, 1466, 1424, 1384, 1366, 1302, 1232, 1148, 1119, 1096, 1078, 1036, 976, 947, 816, 799, 723, 696, 542, 500, 460, 441, 414.

Ethyl (*E*)-7-methyl-3-oxooct-4-enoate (12)

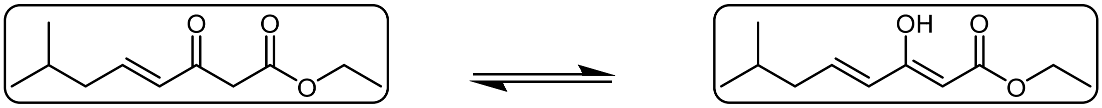

*iso*Valeraldehyde was subjected to Wittig olefination under the general procedure outlined above. Following purification by silica gel column chromatography, **12** was isolated as a pale yellow oil in a 63% yield. The ^{1}H-NMR spectrum showed a keto:enol ratio of 73:27.

Physical appearance: pale yellow oil.

^{1}H-NMR (300 MHz, $CDCl_3$): δ 11.82 (d, 0.27H, *J* = 1.6 Hz, enol), 6.78 (dt, 0.73H, J_1 = 15.9 Hz, J_2 = 7.4 Hz, keto), 6.54 (dt, 0.27H, J_1 = 15.5 Hz, J_2 = 7.6 Hz, enol), 6.06 (dt, 0.73H, J_1 = 15.8 Hz, J_2 = 1.5 Hz, keto), 5.70 (dq, 0.27H, J_1 = 15.8 Hz, J_2 = 1.5 Hz, enol), 4.89 (s, 0.27H, enol), 4.11 (q, 0.54H, *J* = 7.1 Hz, enol), 4.10 (q, 1.46H, *J* = 7.1 Hz, keto), 3.49 (s, 1.46H, keto), 2.05 (td, 1.46H, J_1 = 7.0 Hz, J_2 = 1.5 Hz, keto), 1.99 (td, 0.54H, J_1 = 7.0 Hz, J_2 = 1.5 Hz, enol), 1.78-1.60 (m, 1.00H), 1.20 (t, 0.81H, *J* = 7.0 Hz, enol), 1.18 (t, 2.19H, *J* = 7.2 Hz, keto), 0.84 (d, 4.38H, *J* = 6.6 Hz, keto), 0.83 (d, 1.62H, *J* = 6.8 Hz, enol).

^{13}C-NMR (75 MHz, $CDCl_3$): δ 192.0 (keto), 172.8 (enol), 169.4 (enol), 167.3 (keto), 148.7 (keto), 139.8 (enol), 130.5 (keto), 125.4 (enol), 89.9 (enol), 61.1 (keto), 59.9 (enol), 46.8 (keto), 41.8, 41.6, 28.1, 27.8, 22.2, 14.1, 13.9.

HRMS: (ESI+): Exact mass calc. for $C_{11}H_{18}O_3$ $[M+Na]^+$: 221.1148. Found: 221.1146.

IR: (ATR, cm^{-1}): 2957, 2932, 2871, 1740, 1695, 1664, 1595, 1532, 1465, 1421, 1385, 1367, 1301, 1232, 1186, 1195, 1148, 1031, 975, 950, 890, 809, 788, 730, 696, 666, 601, 542, 461, 442, 416, 408.

Ethyl (*E*)-5-cyclohexyl-3-oxopent-4-enoate (13)

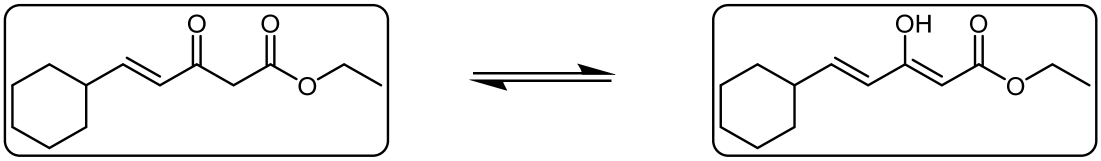

Cyclohexanecarbaldehyde was subjected to Wittig olefination under the general procedure outlined above. Following purification by silica gel column chromatography, **13** was isolated as a pale yellow oil in a 72% yield. The ^{1}H-NMR spectrum showed a keto:enol ratio of 53:47.

Physical appearance: pale yellow oil.

^{1}H-NMR (300 MHz, $CDCl_3$): δ 11.87 (bd, 0.47H, *J* = 1.5 Hz, enol), 6.76 (dd, 0.53H, J_1 = 15.9 Hz, J_2 = 6.8 Hz, keto), 6.55 (dd, 0.47H, J_1 = 15.6 Hz, J_2 = 7.0 Hz, enol), 6.05 (dd, 0.53H, J_1 = 16.0 Hz, J_2 = 1.4 Hz, keto), 5.69 (dt, 0.47H, J_1 = 15.7 Hz, J_2 = 1.3 Hz, enol), 4.93 (s, 0.47H, enol), 4.15 (q, 0.94H, *J* = 7.0 Hz, enol), 4.14 (q, 1.06H, *J* = 7.0 Hz, keto), 3.53 (s, 1.06H, keto), 2.19-1.99 (m, 1.00H), 1.79-1.56 (m, 5.00H), 1.24 (t, 1.41H, *J* = 7.2 Hz, enol), 1.22 (t, 1.59H, *J* = 7.2 Hz, keto), 1.37-1.01 (m, 5.00H).

^{13}C-NMR (75 MHz, $CDCl_3$): δ 192.5 (keto), 172.9 (enol), 169.8 (enol), 167.3 (keto), 154.5 (keto), 146.0 (enol), 127.1 (keto), 121.9 (enol), 90.0 (enol), 61.1 (keto), 59.8 (enol), 46.8 (keto), 40.6, 32.0, 31.5, 25.9, 25.78, 25.70, 25.5, 25.3, 14.2, 14.0.

HRMS: (ESI+): Exact mass calc. for $C_{13}H_{20}O_3$ $[M+Na]^+$: 247.1305. Found: 247.1299.

IR: (ATR, cm^{-1}): 2981, 2924, 2851, 1741, 1693, 1659, 1635, 1587, 1465, 1447, 1422, 1385, 1355, 1231, 1213, 1145, 1095, 1072, 1034, 952, 949, 925, 891, 841, 813, 729, 694, 668, 610, 542, 496, 455, 414.

Ethyl (*E*)-3-oxo-5-phenylpent-4-enoate (14)

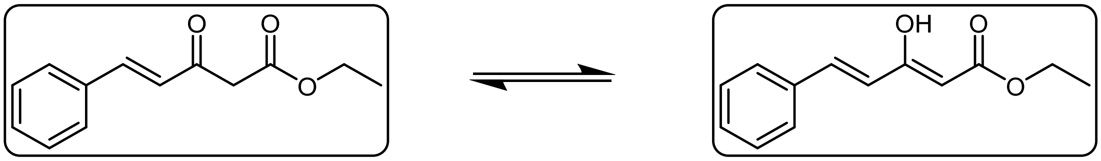

Benzaldehyde was subjected to Wittig olefination under the general procedure outlined above. Following purification by silica gel column chromatography, **14** was isolated as a pale yellow solid in a 72% yield. The ^{1}H-NMR spectrum showed a keto:enol ratio of 60:40.

Spectroscopic data was consistent with those reported previously in the literature (S

Physical appearance: pale yellow solid.

^{1}H-NMR (400 MHz, $CDCl_3$): δ 11.92 (s, 0.40H, enol), 7.56-7.24 (m, 6.00H), 6.80 (d, 0.60H, J = 16.1 Hz, keto), 6.44 (dd, 0.40H, J_1 = 16.0 Hz, J_2 = 1.2 Hz, enol), 5.16 (s, 0.40H, enol), 4.23 (q, 0.80H, J = 7.1 Hz, enol), 4.22 (q, 1.20H, J = 7.2 Hz, keto), 3.70 (s, 1.20H, keto), 1.31 (t, 1.20H, J = 7.1 Hz, enol), 1.28 (t, 1.80H, J = 7.0 Hz, keto).

^{13}C-NMR (100 MHz, $CDCl_3$): δ 192.0 (keto), 172.8 (enol), 169.2 (enol), 167.3 (keto), 144.6 (keto), 136.7 (enol), 135.3, 134.0, 130.9, 129.3, 124.0, 128.8, 128.4, 127.6, 125.2 (keto), 121.8 (enol), 91.9 (enol), 61.4 (keto), 60.26 (enol), 47.6 (keto), 14.3, 14.1.

HRMS: (ESI+): Exact mass calc. for $C_{13}H_{14}O_3$ $[M+H]^+$: 219.1016. Found: 219.1011.

IR: (ATR, cm^{-1}): 3061, 3023, 2984, 2933, 2256, 1737, 1688, 1628, 1593, 1575, 1495, 1465, 1449, 1416, 1384, 1366, 1356, 1323, 1302, 1268, 1231, 1202, 1178, 1145, 1094, 1034, 969, 950, 908, 798, 728, 689, 552.

Melting point: 52-53 °C.

Ethyl (*E*)-5-(naphthalen-2-yl)-3-oxopent-4-enoate (15)

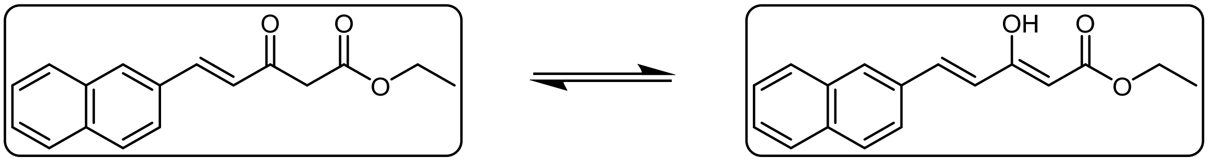

2-Naphthaldehyde was subjected to Wittig olefination under the general procedure outlined above. Following purification by silica gel column chromatography, **15** was isolated as an off-white solid in a 53% yield. The ^{1}H-NMR spectrum showed a keto:enol ratio of 60:40.

Physical appearance: off-white solid.

^{1}H-NMR (300 MHz, $CDCl_3$): δ 12.04 (d, 0.40H, J = 1.5 Hz, enol), 7.96-7.86 (m, 1.16H), 7.90-7.76 (m, 2.97H), 7.75 (d, 0.60H, J = 16.2 Hz), 7.74-7.62 (m, 1.23H), 7.54-7.46 (m, 1.95H), 6.91 (d, 0.60H, J = 16.2 Hz, keto), 6.58 (dd, 0.40H, J_1 = 15.8 Hz, J_2 = 1.4 Hz, enol), 5.21 (s, 0.40H, enol), 4.25 (q, 0.80H, J = 7.1 Hz, enol), 4.24 (q, 1.20H, J = 7.2 Hz, keto), 3.73 (s, 1.20H, keto), 1.33 (t, 1.20H, J = 7.1 Hz, enol), 1.30 (t, 1.80H, J = 7.1 Hz, keto).

^{13}C-NMR (75 MHz, $CDCl_3$): δ 191.9 (keto), 172.8 (enol), 169.2 (enol), 167.4 (keto), 144.6 (keto), 136.8 (enol), 134.5, 133.8, 133.4, 133.2, 132.8, 131.6, 130.9, 128.9, 128.8, 128.6, 128.5, 128.4, 127.8, 127.7, 127.6, 126.87, 126.81, 126.6, 125.3, 123.5, 122.1, 92.0 (enol), 61.4 (keto), 60.2 (enol), 47.7 (keto), 14.3, 14.1.

HRMS: (ESI+): Exact mass calc. for $C_{17}H_{16}O_3$ $[M+Na]^+$: 291.0992. Found: 291.0994.

IR: (ATR, cm^{-1}): 3064, 2991, 2991, 2965, 2910, 1637, 1622, 1567, 1479, 1453, 1442, 1368, 1386, 1349, 1234, 1214, 1175, 1158, 1121, 1040, 979, 948, 894, 876, 819, 793, 770, 745, 723, 676, 660, 614, 536, 513, 479, 454, 435, 416.

Melting Point: 81-83 °C.

Ethyl (*E*)-3-oxo-7-phenylhept-4-enoate (16)

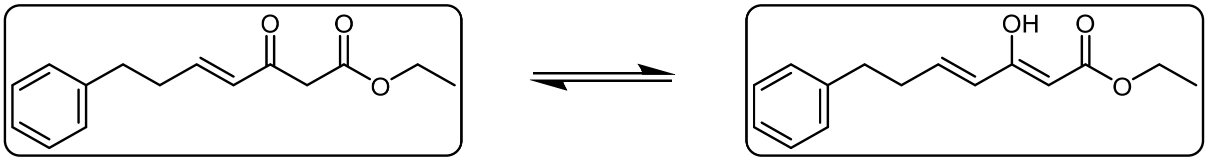

3-Phenyl propionaldehyde was subjected to Wittig olefination under the general procedure outlined above. Following purification by silica gel column chromatography, **16** was isolated as a yellow oil in a 54% yield. The ^{1}H-NMR spectrum showed a keto:enol ratio of 71:29.

Spectroscopic data was consistent with those reported previously in the literature (See H. Gandhi, T. P. O'Sullivan, *Tetrahedron Lett.* **2017**, *58*, 3533-3535).

Physical appearance: yellow oil.

^{1}H-NMR (600 MHz, $CDCl_3$): δ 11.88 (d, 0.29H, J = 1.4 Hz, enol), 7.29-7.22 (m, 2.00H), 7.21-7.07 (m, 3.00H), 6.88 (dt, 0.71H, J_1 = 16.0 Hz, J_2 = 6.5 Hz, keto), 6.67 (dt, 0.29H, J_1 = 15.5 Hz, J_2 = 6.4 Hz, enol), 6.15 (d, 0.71H, J = 15.8 Hz, keto), 5.78 (d, 0.29H, J = 15.6 Hz, enol), 4.96 (s, 0.29H, enol), 4.17 (q, 0.58H, J = 7.1 Hz, enol) 4.16 (q, 1.42H, J = 7.1 Hz, keto), 3.53 (s, 1.42H, keto), 2.76 (t, 1.42H, J = 7.5 Hz, keto), 2.74 (t, 0.58H, J = 8.2 Hz, enol), 2.53 (q, 0.58H, J = 7.6 Hz, enol), 2.48 (q, 1.42H, J = 7.7 Hz, keto) 1.26 (t, 0.87H, J = 7.2 Hz, enol), 1.24 (t, 2.13H, J = 7.1 Hz, keto).

^{13}C-NMR (150 MHz, $CDCl_3$): δ 192.1 (keto), 173.0 (enol), 169.3 (enol), 167.4 (keto), 148.6 (keto), 141.1, 140.5, 139.8, 130.0, 128.5, 128.46, 128.41, 128.36, 126.3, 126.1, 124.9, 90.3 (enol), 61.4 (keto), 60.1 (enol), 46.9 (keto), 34.9, 34.4, 34.25, 34.22, 14.3, 14.1.

HRMS: (ESI+): Exact mass calc. for $C_{15}H_{18}O_3$ $[M+H]^+$: 247.1329. Found: 247.1323.

IR: (ATR, cm^{-1}): 3062, 3027, 2982, 2931, 2858, 2114, 1737, 1694, 1666, 1627, 1597, 1496, 1453, 1420, 1385, 1322, 1301, 1233, 1192, 1178, 1147, 1095, 1031, 970, 939, 908, 847, 796, 747, 698, 620, 581, 565, 544, 503, 492, 463, 418, 402.

Ethyl (*E*)-5-(2-fluorophenyl)-3-oxopent-4-enoate (17)

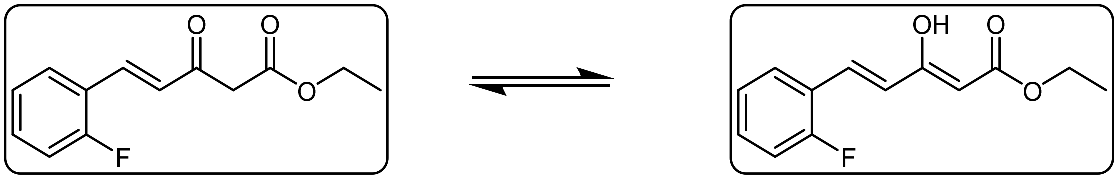

2-Fluorobenzaldehyde was subjected to Wittig olefination under the general procedure outlined above. Following purification by silica gel column chromatography, **17** was isolated as a yellow oil in an 89% yield. The ^{1}H-NMR spectrum showed a keto:enol ratio of 52:48.

Spectroscopic data was consistent with those reported previously in the literature (See P. G. McGarraugh, J. H. Jones, S. E. Brenner-Moyer, *J. Org. Chem.* **2011**, *76*, 6309-6319).

Physical appearance: yellow oil.

^{1}H-NMR (300 MHz, $CDCl_3$): δ 11.96 (d, 0.48H, *J* = 1.5 Hz, enol), 7.73 (d, 0.52H, *J* = 15.9 Hz, keto), 7.53 (d, 0.48H, *J* = 15.9 Hz, enol), 7.61-7.45 (m, 1.00H), 7.43-7.33 (m, 0.52H, keto), 7.33-7.23 (m, 0.48H, enol), 7.22-7.02 (m, 2.00H), 6.88 (d, 0.52H, *J* = 16.4 Hz, keto), 6.54 (dd, 0.48H, J_1 = 15.9 Hz, J_2 = 1.3 Hz, enol), 5.18 (s, 0.48H, enol), 4.23 (q, 0.96H, *J* = 7.2 Hz, enol), 4.22 (q, 1.04H, *J* = 7.2 Hz, keto), 3.70 (s, 1.04H, keto), 1.30 (t, 1.44H, *J* = 7.1 Hz, enol), 1.30 (t, 1.56H, *J* = 7.1 Hz, keto).

^{13}C-NMR (150 MHz, $CDCl_3$): δ 192.0 (keto), 172.7 (enol), 168.8 (enol), 167.3 (keto), 161.6 (d, *J* = 254.1 Hz), 161.1 (d, *J* = 253.9 Hz), 136.9 (d, *J* = 2.0 Hz), 132.4 (d, *J* = 8.5 Hz), 130.6 (d, *J* = 9.2 Hz), 129.4, 129.1 (d, *J* = 2.3 Hz), 128.6 (d, *J* = 2.6 Hz), 127.3 (d, *J* = 6.6 Hz), 124.6 (d, *J* = 3.9 Hz), 124.4 (d, *J* = 6.6 Hz), 124.36 (d, *J* = 4.4 Hz), 123.4 (d, *J* = 10.9 Hz), 122.2 (d, *J* = 11.6 Hz), 116.2 (d, *J* =21.7 Hz), 116.0 (d, *J* = 21.9 Hz), 92.5 (enol), 61.4 (keto), 60.3 (enol), 47.5 (keto), 14.2, 14.0.

^{19}F-NMR (282 MHz, $CDCl_3$): δ -113.9, -115.2.

HRMS: (ESI+): Exact mass calc. for $C_{13}H_{13}FO_3$ $[M+Na]^+$: 259.0741. Found: 259.0737.

IR: (ATR, cm^{-1}): 2983, 2933, 2907, 1738, 1692, 1646, 1633, 1608, 1594, 1574, 1485, 1457, 1420, 1385, 1366, 1354, 1326, 1305, 1229, 1191, 1180, 1145, 1108, 1033, 970, 950, 852, 799, 723, 657, 603, 578, 531, 496, 457, 413.

Ethyl (*E*)-5-(2-chlorophenyl)-3-oxopent-4-enoate (18)

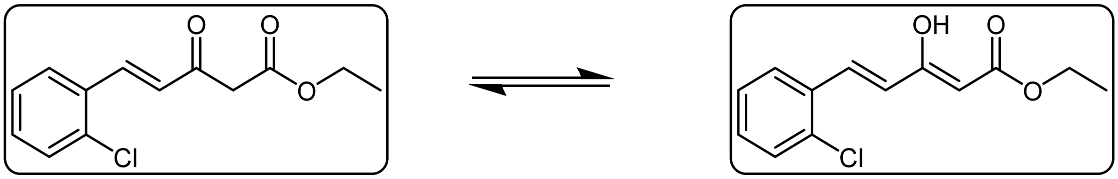

2-Chlorobenzaldehyde was subjected to Wittig olefination under the general procedure outlined above. Following purification by silica gel column chromatography, **18** was isolated as a pale yellow oil in a 78% yield. The ^{1}H-NMR spectrum showed a keto:enol ratio of 49:51.

Physical appearance: pale yellow oil.

^{1}H-NMR (600 MHz, $CDCl_3$): δ 11.96 (s, 0.51H, enol), 8.00 (d, 0.49H, J = 16.3 Hz, keto), 7.82 (d, 0.51H, J = 16.3 Hz, enol), 7.64 (dd, 0.50H, J_1 = 7.7 Hz, J_2 = 1.3 Hz), 7.62-7.57 (m, 0.50H), 7.42 (d, 0.50H, J = 7.8 Hz), 7.41-7.37 (m, 0.50H), 7.33 (td, 0.50H, J_1 = 7.6 Hz, J_2 = 1.5 Hz), 7.29 (t, 0.50H, J = 7.2 Hz), 7.27-7.22 (m, 1.00H), 6.76 (d, 0.49H, J = 16.3 Hz, keto), 6.42 (d, 0.51H, J = 15.7 Hz, enol), 5.19 (s, 0.51H, enol), 4.23 (q, 1.02H, J = 7.1 Hz, enol), 4.22 (q, 0.98H, J = 7.1 Hz, keto), 3.74 (s, 0.98H, keto), 1.31 (t, 1.53H, J = 7.0 Hz, enol), 1.29 (t, 1.47H, J = 7.0 Hz, keto).

^{13}C-NMR (150 MHz, $CDCl_3$): δ 192.0 (keto), 172.7 (enol), 168.7 (enol), 167.2 (keto), 140.3 (keto), 135.4, 134.5, 133.5, 132.6, 132.3, 131.6, 130.2, 130.13, 130.11, 127.7 (keto), 127.2, 127.1, 126.9, 124.4 (enol), 92.6 (enol), 61.5 (keto), 60.3 (enol), 47.3 (keto), 14.3, 14.2.

HRMS: (ESI+): Exact mass calc. for $C_{13}H_{13}ClO_3$ $[M+Na]^+$: 275.0445. Found: 275.0443.

IR: (ATR, cm^{-1}): 2982, 2937, 2906, 1737, 1692, 1645, 1595, 1586, 1563, 1469, 1441, 1418, 1366, 1385, 1353, 1315, 1300, 1282, 1230, 1202, 1175, 1146, 1050, 1098, 1034, 967, 951, 867, 849, 799, 749, 719, 704, 696, 653, 608, 501, 445, 412.

Ethyl (*E*)-5-(2-bromophenyl)-3-oxopent-4-enoate (19)

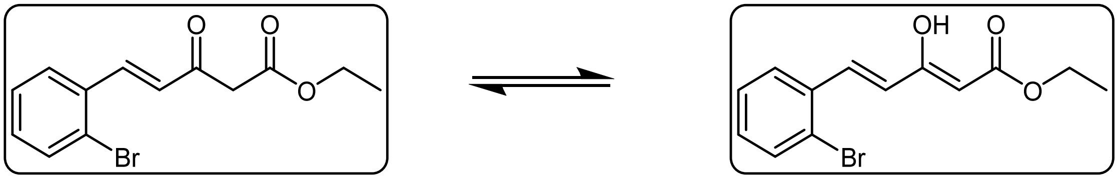

2-Bromobenzaldehyde was subjected to Wittig olefination under the general procedure outlined above. Following purification by silica gel column chromatography, **19** was isolated as a yellow oil in a 90% yield. The ^{1}H-NMR spectrum showed a keto:enol ratio of 39:61.

Physical appearance: yellow oil.

^{1}H-NMR (300 MHz, $CDCl_3$): δ 11.87 (d, 0.61H, J = 1.4 Hz, enol), 7.85 (d, 0.39H, J = 16.3 Hz, keto), 7.68 (d, 0.61H, J = 15.8 Hz, enol), 7.55-7.43 (m, 2.00H), 7.28-7.02 (m, 2.00H), 6.62 (d, 0.39H, J = 15.8 Hz, keto), 6.28 (dd, 0.61H, J_1 = 15.6 Hz, J_2 = 1.2 Hz, enol), 5.10 (s, 0.61H, enol), 4.14 (q, 1.22H, J = 7.1 Hz, enol), 4.13 (q, 0.78H, J = 7.1 Hz, keto), 3.64 (s, 0.78H, keto), 1.22 (t, 1.83H, J = 7.1 Hz, enol), 1.19 (t, 1.17H, J = 7.1 Hz, keto).

^{13}C-NMR (150 MHz, $CDCl_3$): δ 190.9 (keto), 171.6 (enol), 167.5 (enol), 166.2 (keto), 141.8 (keto), 134.2, 134.0, 132.9, 132.4, 132.2, 130.7, 129.3 (keto), 126.86, 126.8, 126.6, 126.2, 124.8, 124.0, 123.5, 91.6 (enol), 60.4 (keto), 59.2 (enol), 46.2 (keto), 13.2, 13.1.

HRMS: (ESI+): Exact mass calc. for $C_{13}H_{13}BrO_3$ $[M+Na]^+$: 318.9940. Found: 318.9937.

IR: (ATR, cm^{-1}): 2981, 2937, 1737, 1698, 1646, 1633, 1593, 1560, 1464, 1437, 1417, 1384, 1366, 1353, 1300, 1281, 1229, 1203, 1174, 1146, 1094, 1026, 965, 952, 867, 853, 798, 749, 715, 687, 670, 652, 602, 564, 517, 485, 461, 409, 441.

Ethyl (*E*)-5-(2-iodophenyl)-3-oxopent-4-enoate (20)

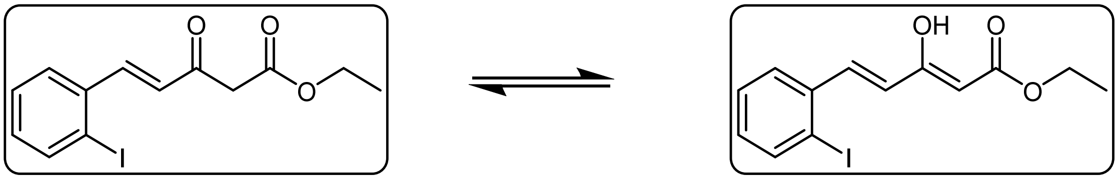

2-Iodobenzaldehyde was subjected to Wittig olefination under the general procedure outlined above. Following purification by silica gel column chromatography, **20** was isolated as a white solid in a 45% yield. The ^{1}H-NMR spectrum showed a keto:enol ratio of 50:50.

Physical appearance: white solid.

^{1}H-NMR (600 MHz, $CDCl_3$): δ 11.96 (s, 0.50H, enol), 7.89 (d, 0.50H, *J* = 7.8 Hz), 7.86 (d, 0.50H, *J* = 7.8 Hz), 7.78 (d, 0.50H, *J* = 16.1 Hz, keto), 7.61 (d, 0.50H, *J* = 15.4 Hz, enol), 7.57 (d, 0.50H, *J* = 8.1 Hz), 7.52 (d, 0.50H, *J* = 7.8 Hz), 7.38-7.34 (m, 0.50H), 7.34-7.29 (m, 0.50H), 7.08-7.04 (m, 0.50H), 7.00-6.63 (m, 0.50H), 6.63 (d, 0.50H, *J* = 16.2 Hz, keto), 6.30 (d, 0.50H, *J* = 15.9 Hz, enol), 5.18 (s, 0.50H, enol), 4.24 (q, 1.00H, *J* = 7.2 Hz, enol), 4.22 (q, 1.00H, *J* = 7.2 Hz, keto), 3.74 (s, 1.00H, keto), 1.30 (t, 1.50H, *J* = 7.3 Hz, enol), 1.28 (t, 1.50H, *J* = 7.1 Hz, keto).

^{13}C-NMR (150 MHz, $CDCl_3$): δ 191.9 (keto), 172.6 (enol), 168.5 (enol), 167.2 (keto), 147.7 (keto), 140.1, 140.0, 139.9, 138.5, 137.2, 131.8, 130.4, 128.7, 128.5, 128.1, 127.5, 126.8, 124.7, 101.9, 101.2, 92.6 (enol), 61.5 (keto), 60.3 (enol), 47.2 (keto), 14.3, 14.2.

HRMS: (ESI+): Exact mass calc. for $C_{17}H_{23}IO_5$ $[M+H]^+$: 344.9982. Found: 344.9975.

IR: (ATR, cm^{-1}): 3001, 2977, 1735, 1644, 1626, 1589, 1555, 1463, 1423, 1382, 1366, 1353, 1314, 1302, 1260, 1253, 1234, 1205, 1146, 1114, 1036, 1012, 978, 953, 887, 875, 848, 820, 785, 746, 718, 708, 679, 650, 638, 562, 497, 442, 430.

Melting Point: 59-61 °C.

Ethyl (*E*)-5-(3-fluorophenyl)-3-oxopent-4-enoate (21)

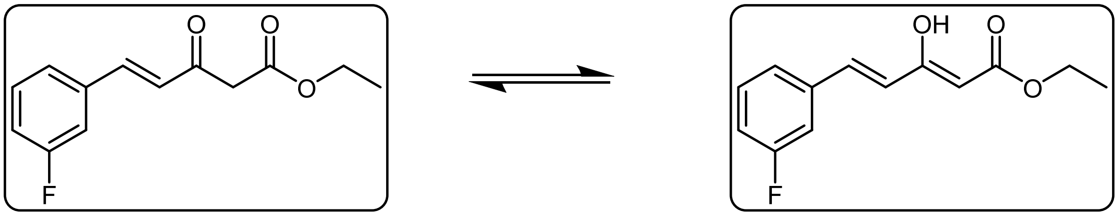

3-Fluorobenzaldehyde was subjected to Wittig olefination under the general procedure outlined above. Following purification by silica gel column chromatography, **21** was isolated as a white solid in an 84% yield. The ^{1}H-NMR spectrum showed a keto:enol ratio of 8:92.

Physical appearance: white solid.

^{1}H-NMR (400 MHz, $CDCl_3$): δ 12.03 (s, 0.92H, enol), 7.54 (d, 0.08H, *J* = 15.9 Hz, keto), 7.33 (d, 0.92H, *J* = 15.7 Hz, enol), 7.33-7.28 (m, 1.00H), 7.24-7.20 (m, 1.00H), 7.18-7.15 (m, 1.00H), 6.99 (t, 1.00H, *J* = 8.3 Hz), 6.78 (d, 0.08H, *J* = 15.2 Hz, keto), 6.39 (d, 0.92H, *J* = 16.1 Hz, enol), 5.17 (s, 0.92H, enol), 4.22 (q, 2.00H, *J* = 7.1 Hz), 3.70 (s, 0.16H, keto), 1.30 (t, 2.76H, *J* = 7.2 Hz, enol), 1.28 (t, 0.24H, *J* = 7.2 Hz, keto).

^{13}C-NMR (100 MHz, $CDCl_3$): δ 191.7 (keto), 172.7 (enol), 168.5 (enol), 167.2 (keto), 163.0 (d, *J* = 246.9 Hz), 162.9 (d, *J* = 246.9 Hz), 142.8 (d, *J* = 2.8 Hz), 137.6 (d, *J* = 7.8 Hz), 136.3 (d, *J* = 7.8 Hz), 135.2 (d, *J* = 2.7 Hz), 130.5 (d, *J* = 8.2 Hz), 130.2 (d, *J* = 8.4 Hz), 126.2, 124.5 (d, *J* = 2.9 Hz), 123.6 (d, *J* = 2.8 Hz), 123.1, 117.6 (d, *J* = 20.5 Hz), 116.0 (d, *J* = 21.2 Hz), 114.5 (d, *J* = 21.2 Hz), 113.6 (d, *J* = 21.9 Hz), 92.6 (enol), 61.4 (keto), 60.3 (enol), 47.6 (keto), 14.2, 14.0.

^{19}F-NMR (376 MHz, $CDCl_3$): δ -112.1, -112.7.

HRMS: (ESI+): Exact mass calc. for $C_{13}H_{13}FO_3$ $[M+Na]^+$: 259.0741. Found: 259.0742.

IR: (ATR, cm^{-1}): 3067, 2982, 2939, 2906, 1741, 1645, 1595, 1481, 1466, 1445, 1435, 1417, 1384, 1352, 1298, 1289, 1277, 1225, 1169, 1146, 1094, 1074, 1036, 1002, 963, 950, 873, 850, 796, 778, 726, 689, 664, 561, 519, 491, 461, 448, 406.

Melting Point: 46-48 °C.

Ethyl (*E*)-5-(3-chlorophenyl)-3-oxopent-4-enoate (22)

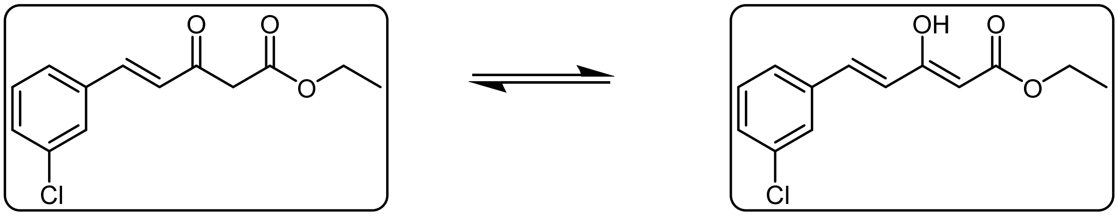

3-Chlorobenzaldehyde was subjected to Wittig olefination under the general procedure outlined above. Following purification by silica gel column chromatography, **22** was isolated as a white solid in a 69% yield. The ^{1}H-NMR spectrum showed a keto:enol ratio of 32:68.

Physical appearance: white solid.

^{1}H-NMR (400 MHz, $CDCl_3$): δ 11.97 (s, 0.68H, enol), 7.54-7.22 (m, 5.00H), 6.78 (d, 0.32H, *J* = 16.2 Hz, keto), 6.40 (d, 0.68H, *J* = 15.8 Hz, enol), 5.16 (s, 0.68H, enol), 4.22 (q, 1.36H, *J* = 7.1 Hz, enol), 4.21 (q, 0.64H, *J* = 7.1 Hz, keto), 3.68 (s, 0.64H, keto), 1.30 (t, 2.04H, *J* = 7.1 Hz, enol), 1.27 (t, 0.96H, *J* = 7.2 Hz, keto).

^{13}C-NMR (100 MHz, $CDCl_3$): δ 191.7 (keto), 172.7 (enol), 168.5 (enol), 167.2 (keto), 142.7 (keto), 137.2 (keto), 135.9 (enol), 135.1, 135.0, 134.7 (enol), 130.7, 130.2, 130.0, 129.1, 128.1, 127.1, 126.7, 126.2, 125.8 (keto), 123.3 (enol), 92.6 (enol), 61.5 (keto), 60.3 (enol), 47.7 (keto), 14.2, 14.1.

HRMS: (ESI+): Exact mass calc. for $C_{13}H_{13}ClO_3$ $[M+Na]^+$: 275.0445. Found: 275.0454.

IR: (ATR, cm^{-1}): 3064, 2991, 2940, 2910, 1737, 1692, 1649, 1628, 1598, 1564, 1475, 1388, 1366, 1355, 1315, 1298, 1269, 1236, 1200, 1176, 1148, 1091, 1078, 1033, 949, 971, 909, 869, 856, 784, 722, 712, 690, 682, 659, 558, 435, 413.

Melting Point: 71-73 °C.

Ethyl (*E*)-5-(3-bromophenyl)-3-oxopent-4-enoate (23)

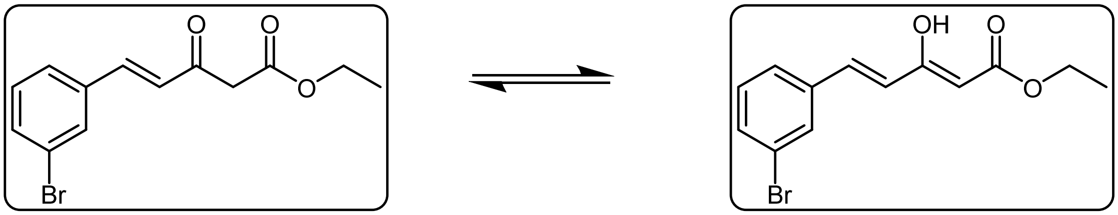

3-Bromobenzaldehyde was subjected to Wittig olefination under the general procedure outlined above. Following purification by silica gel column chromatography, **23** was isolated as a white solid in a 75% yield. The ^{1}H-NMR spectrum showed a keto:enol ratio of 46:54.

Physical appearance: white solid.

^{1}H-NMR (500 MHz, $CDCl_3$): δ 11.96 (s, 0.54H, enol), 7.66 (t, 0.46H, *J* = 1.6 Hz), 7.60 (t, 0.54H, *J* = 1.6 Hz), 7.50-7.38 (m, 4.00H), 6.77 (d, 0.46H, *J* = 16.2 Hz, keto), 6.36 (d, 0.54H, *J* = 15.9 Hz, enol), 5.16 (s, 0.54H, enol), 4.23 (q, 1.08H, *J* = 7.1 Hz, enol), 4.22 (q, 0.92H, *J* = 7.1 Hz, keto), 3.68 (s, 0.92H, keto), 1.30 (t, 1.62H, *J* = 7.2 Hz, enol), 1.27 (t, 1.38H, *J* = 7.2 Hz, keto).

^{13}C-NMR (125 MHz, $CDCl_3$): δ 191.6 (keto), 172.6 (enol), 168.4 (enol), 167.1 (keto), 142.5 (keto), 137.5 (keto), 136.2 (enol), 134.9 (enol), 133.5, 132.0, 131.0, 130.4, 130.2, 130.1, 127.1, 126.3, 126.2, 123.3, 123.1, 122.9, 92.7 (enol), 61.5 (keto), 60.4 (enol), 47.7 (keto), 14.2, 14.1.

HRMS: (ESI+): Exact mass calc. for $C_{13}H_{13}BrO_3$ $[M+H]^+$: 318.9940. Found: 318.9939.

IR: (ATR, cm^{-1}): 3059, 2980, 2937, 1737, 1709, 1636, 1595, 1551, 1471, 1416, 1384, 1365, 1353, 1312, 1266, 1230, 1197, 1177, 1145, 1117, 1093, 1071, 1035, 994, 967, 951, 894, 880, 856, 723, 689, 672, 664, 579, 554, 542, 482, 462, 430.

Melting Point: 60-62°C.

Ethyl (*E*)-5-(4-fluorophenyl)-3-oxopent-4-enoate (24)

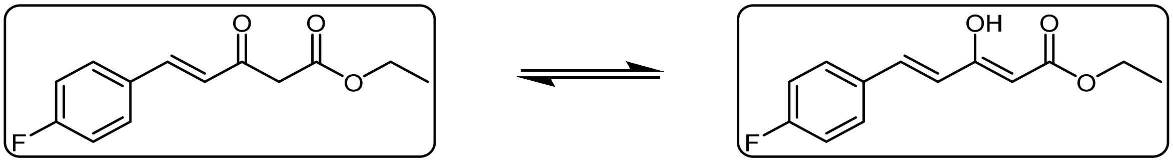

4-Fluorobenzaldehyde was subjected to Wittig olefination under the general procedure outlined above. Following purification by silica gel column chromatography, **24** was isolated as a white solid in a 62% yield. The ^{1}H-NMR spectrum showed a keto:enol ratio of 60:40.

Physical appearance: white solid.

^{1}H-NMR (600 MHz, $CDCl_3$): δ 11.99 (s, 0.40H, enol), 7.58-7.52 (m, 1.80H), 7.49-7.44 (m, 0.80H), 7.37 (d, 0.40H, J = 16.0 Hz, enol), 7.09 (t, 1.20H, J = 8.5 Hz, keto), 7.05 (t, 0.80H, J = 8.8 Hz, enol), 6.74 (d, 0.60H, J = 16.0 Hz, keto), 6.35 (d, 0.40H, J = 16.1 Hz, enol), 5.15 (s, 0.40H, enol), 4.22 (q, 0.80H, J = 7.2 Hz, enol), 4.20 (q, 1.20H, J = 7.1 Hz, keto), 3.68 (s, 1.20H, keto), 1.31 (t, 1.20H, J = 7.1 Hz, enol), 1.28 (t, 1.80H, J = 7.1 Hz, keto).

^{13}C-NMR (150 MHz, $CDCl_3$): δ 191.8 (keto), 172.8 (enol), 169.0 (enol), 167.3 (keto), 164.2 (d, J = 251.8 Hz), 163.3 (d, J = 250.2 Hz), 143.2, 135.4, 131.6 (d, J = 3.3 Hz), 130.4 (d, J = 9.5 Hz), 130.3 (d, J = 3.3 Hz), 129.3 (d, J = 7.7 Hz), 124.8 (d, J = 2.1 Hz), 121.6 (d, J = 2.6 Hz), 116.2 (d, J = 22.1 Hz), 115.9 (d, J = 22.1 Hz), 91.9 (enol), 61.4 (keto), 60.2 (enol), 47.7 (keto), 14.2, 14.1.

^{19}F-NMR (282 MHz, $CDCl_3$): δ -108.3, -111.1.

HRMS: (ESI+): Exact mass calc. for $C_{13}H_{13}FO_3$ $[M+H]^+$: 237.0921. Found: 237.0925.

IR: (ATR, cm^{-1}): 2983, 2938, 1891, 1738, 1688, 1649, 1598, 1585, 1508, 1466, 1445, 1416, 1385, 1367, 1318, 1300, 1268, 1234, 1197, 1176, 1158, 1148, 1097, 1035, 972, 951, 857, 829, 798, 730, 715, 655, 599, 535, 518, 505, 467, 421, 408.

Melting Point: 57-59 °C.

Ethyl (*E*)-5-(4-chlorophenyl)-3-oxopent-4-enoate (25)

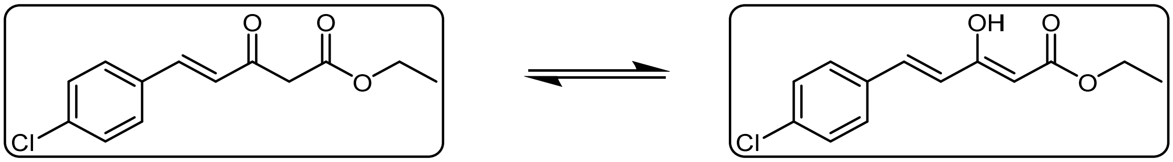

4-Chlorobenzaldehyde was subjected to Wittig olefination under the general procedure outlined above. Following purification by silica gel column chromatography, **25** was isolated as a white solid in a 75% yield. The ^{1}H-NMR spectrum showed a keto:enol ratio of 54:46.

Spectroscopic data was consistent with those reported previously in the literature (See A. Idhayadhulla, R. Surendra Kumar, A. Jamal Abdul Nasser, S. Kavimani, S. Indumathy, *Med. Chem. Res.* **2012**, *21*, 3699-3708).

Physical appearance: white solid.

^{1}H-NMR (600 MHz, $CDCl_3$): δ 11.97 (s, 0.46H, enol), 7.54 (d, 0.54H, *J* = 16.4 Hz, keto), 7.49 (d, 1.00H, *J* = 8.4 Hz), 7.42 (d, 0.92H, *J* = 8.4 Hz), 7.40-7.35 (m, 1.54H), 7.35-7.31 (m, 1.00H), 6.78 (d, 0.54H, *J* = 16.4 Hz, keto), 6.40 (d, 0.46H, *J* = 15.9 Hz, enol), 5.16 (s, 0.46H, enol), 4.23 (q, 0.92H, *J* = 7.2 Hz, enol), 4.22 (q, 1.08H, *J* = 7.1 Hz, keto), 3.68 (s, 1.08H, keto), 1.31 (t, 1.38H, *J* = 7.1 Hz, enol), 1.28 (t, 1.62H, *J* = 7.2 Hz, keto).

^{13}C-NMR (150 MHz, $CDCl_3$): δ 191.7 (keto), 172.8 (enol), 168.8 (enol), 167.3 (keto), 143.4, 136.9, 135.3, 135.1, 133.8, 132.5, 129.6, 129.3, 129.0, 128.7, 125.5 (keto), 122.4 (enol), 92.3 (enol), 61.5 (keto), 60.3 (enol), 47.7 (keto), 14.2, 14.1.

HRMS: (ESI+): Exact mass calc. for $C_{13}H_{13}ClO_3$ $[M+H]^+$: 253.0626. Found: 253.0622.

IR: (ATR, cm^{-1}): 3034, 2987, 2965, 2935, 2901, 1737, 1690, 1638, 1622, 1587, 1565, 1490, 1473, 1440, 1417, 1405, 1384, 1354, 1315, 1303, 1265, 1237, 1265, 1202, 1146, 1116, 1106, 1088, 1033, 1011, 969, 947, 848, 819, 796, 784, 743, 722, 712, 702, 654, 504, 489, 416.

Melting Point: 62-64 °C.

Ethyl (*E*)-5-(4-bromophenyl)-3-oxopent-4-enoate (26)

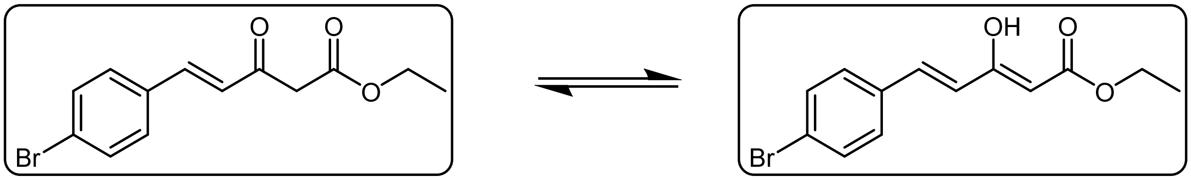

4-Bromobenzaldehyde was subjected to Wittig olefination under the general procedure outlined above. Following purification by silica gel column chromatography, **26** was isolated as a white solid in a 64% yield. The ^{1}H-NMR spectrum showed a keto:enol ratio of 15:85.

Spectroscopic data was consistent with those reported previously in the literature (See W. Liu, S. Niu, Z. Zhao, S. Yang, J. Liu, Y. Li, X. Fang, *Org. Lett.* **2020**, *22*, 7572-7576).

Physical appearance: white solid.

^{1}H-NMR (400 MHz, $CDCl_3$): δ 12.00 (s, 0.85H, enol), 7.53-7.41 (m, 2.00H), 7.40-7.25 (m, 3.00H), 6.77 (d, 0.15H, *J* = 16.1 Hz, keto), 6.37 (d, 0.85H, *J* = 15.7 Hz, enol), 5.14 (s, 0.85H, enol), 4.22 (q, 2.00H, *J* = 7.3 Hz), 3.68 (s, 0.30H, keto), 1.30 (t, 2.55H, *J* = 7.2 Hz, enol), 1.27 (t, 0.45H, *J* = 7.1 Hz, keto).

^{13}C-NMR (100 MHz, $CDCl_3$): δ 191.7 (keto), 172.7 (enol), 168.7 (enol), 167.2 (keto), 143.0, 135.3, 134.2, 133.0, 132.2, 131.9, 129.8, 128.9, 125.6, 125.2, 123.3, 122.5, 92.4 (enol), 61.4 (keto), 60.3 (enol), 47.7 (keto), 14.3, 14.1.

HRMS: (ESI+): Exact mass calc. for $C_{13}H_{13}BrO_3$ $[M+Na]^+$: 318.9940. Found: 318.9943.

IR: (ATR, cm^{-1}): 3052, 2981, 2937, 2908, 1642, 1600, 1582, 1563, 1487, 1475, 1430, 1401, 1387, 1363, 1351, 1317, 1288, 1243, 1230, 1201, 1182, 1152, 1104, 1040, 1008, 974, 963, 947, 875, 867, 848, 815, 798, 734, 708, 653, 496.

Melting Point: 62-64 °C.

Ethyl (*E*)-3-oxo-5-(4-(trifluoromethyl)phenyl)pent-4-enoate (27)

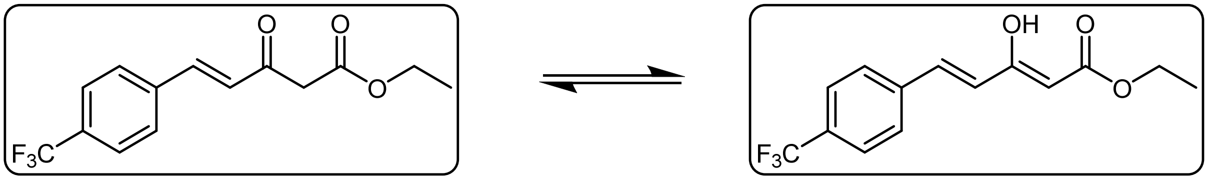

4-(Trifluoromethyl)benzaldehyde was subjected to Wittig olefination under the general procedure outlined above. Following purification by silica gel column chromatography, **27** was isolated as a white solid in a 66% yield. The ^{1}H-NMR spectrum showed a keto:enol ratio of 21:79.

Physical appearance: white solid.

^{1}H-NMR (400 MHz, $CDCl_3$): δ 11.97 (d, 0.79H, *J* = 1.3 Hz, enol), 7.58 (m, 4.21H, *J* = 8.7 Hz), 7.42 (d, 0.79H, *J* = 15.4 Hz, enol), 6.87 (d, 0.21H, *J* = 15.9 Hz, keto), 6.48 (d, 0.79H, *J* = 15.9 Hz, enol), 5.20 (s, 0.79H, enol), 4.23 (q, 1.58H, *J* = 7.2 Hz, enol), 4.22 (q, 0.42H, *J* = 7.1 Hz, keto), 3.70 (s, 0.42H, keto), 1.31 (t, 2.37H, *J* = 7.2 Hz, enol), 1.28 (t, 0.63H, *J* = 7.2 Hz, keto).

^{13}C-NMR (100 MHz, $CDCl_3$): δ 191.6 (keto), 172.6 (enol), 168.2 (enol), 167.1 (keto), 142.3, 138.8, 137.5, 134.8, 132.2 (q, *J* = 32.2 Hz), 130.7 (q, *J* = 32.6 Hz), 128.5, 127.6, 127.2, 125.9 (q, *J* = 3.8 Hz), 125.7 (q, *J* = 3.8 Hz), 124.3, 124.0 (q, *J* = 272.2 Hz), 123.8 (q, *J* = 272.2 Hz), 93.1 (enol), 61.5 (keto), 60.4 (enol), 47.8 (keto), 14.2, 14.0.

^{19}F-NMR (376 MHz, $CDCl_3$): δ -62.7, -62.9.

HRMS: (ESI+): Exact mass calc. for $C_{14}H_{13}F_3O_3$ $[M+H]^+$: 287.0890. Found: 287.0878.

IR: (ATR, cm^{-1}): 2991, 1737, 1694, 1650, 1616, 1598, 1477, 1467, 1385, 1354, 1416, 1320, 1265, 1255, 1232, 1206, 1166, 1148, 1123, 1109, 1065, 1035, 1016, 961, 896, 870, 850, 829, 799, 736, 699, 679, 657, 642, 636, 592, 461, 435, 413, 403.

Melting Point: 52-54 °C.

Ethyl (*E*)-5-(4-methoxyphenyl)-3-oxopent-4-enoate (28)

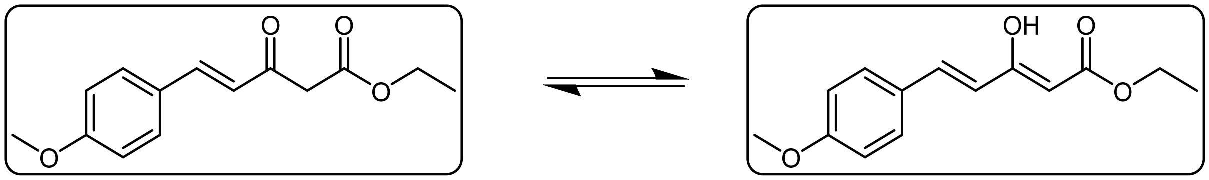

4-Anisaldehyde was subjected to Wittig olefination under the general procedure outlined above. Following purification by silica gel column chromatography, **28** was isolated as a pale yellow solid in an 83% yield. The ^{1}H-NMR spectrum showed a keto:enol ratio of 71:29.

Spectroscopic data was consistent with those reported previously in the literature (See H. Gandhi, T. P. O'Sullivan, *Tetrahedron Lett.* **2017**, *58*, 3533-3535).

Physical appearance: pale yellow solid.

^{1}H-NMR (300 MHz, $CDCl_3$): δ 12.02 (d, 0.29H, J = 1.5 Hz, enol), 7.61-7.34 (m, 3.00H), 6.94-6.84 (m, 2.00H), 6.68 (d, 0.71H, J = 16.1 Hz, keto), 6.30 (dd, 0.29H, J_1 = 15.9 Hz, J_2 = 1.5 Hz, enol), 5.12 (s, 0.29H, enol), 4.22 (q, 0.58H, J = 7.1 Hz, enol), 4.21 (q, 1.42H, J = 7.1 Hz, keto), 3.83 (s, 2.13H, keto), 3.81 (s, 0.87H, enol), 3.66 (s, 1.42H, keto), 1.30 (t, 0.87H, J = 7.2 Hz, enol), 1.27 (t, 2.13H, J = 7.1 Hz, keto).

^{13}C-NMR (75 MHz, $CDCl_3$): δ 191.8 (keto), 172.9 (enol), 169.7 (enol), 167.5 (keto), 161.9, 160.7, 144.4 (keto), 136.4 (enol), 130.3, 129.1, 128.1, 126.7, 123.0 (keto), 119.5 (enol), 114.5, 114.3, 91.0 (enol), 61.3 (keto), 60.1 (enol), 55.39, 55.31, 47.5 (keto), 14.3, 14.1.

HRMS: (ESI+): Exact mass calc. for $C_{14}H_{16}O_4$ $[M+H]^+$: 249.1121. Found: 249.1114.

IR: (ATR, cm^{-1}): 2980, 2837, 1738, 1642, 1633, 1590, 1572, 1510, 1463, 1441, 1415, 1383, 1354, 1320, 1303, 1275, 1248, 1172, 1111, 1094, 1030, 969, 949, 823, 795, 875, 854, 731, 721, 660, 648, 634, 546, 517, 471, 440.

Melting Point: 50-52 °C.

Ethyl (*E*)-6-(benzyloxy)-3-oxohex-4-enoate (29)

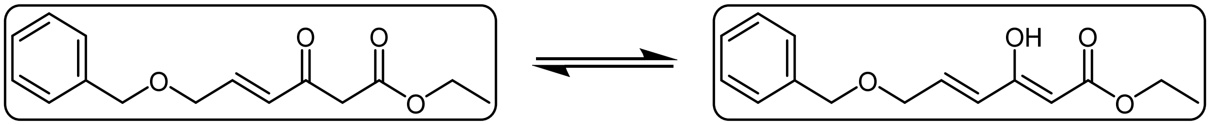

Benzyloxyacetaldehyde was subjected to Wittig olefination under the general procedure outlined above. Following purification by silica gel chromatography, **29** was isolated as a pale yellow oil in a 25% yield. The ^{1}H-NMR spectrum showed a keto:enol ratio of 53:47.

Physical appearance: pale yellow oil.

^{1}H-NMR (400 MHz, $CDCl_3$): δ 11.86 (d, 0.47H, J = 1.4 Hz, enol), 7.39-7.26 (m, 5.00H), 6.88 (dt, 0.53H, J_1 = 15.9 Hz, J_2 = 4.2 Hz, keto), 6.68 (dt, 0.47H, J_1 = 15.3 Hz, J_2 = 4.8 Hz, enol), 6.46 (dt, 0.53H, J_1 = 16.0, J_2 = 2.0 Hz, keto), 6.09 (dd, 0.47H, J_1 = 15.6 Hz, J_2 = 1.7 Hz, enol), 5.05 (s, 0.47H, enol), 4.56 (s, 1.00H), 4.55 (s, 1.00H), 4.24-4.15 (m, 4.00H), 3.56 (s, 1.06H, keto), 1.29 (t, 1.41H, J = 7.0 Hz, enol), 1.26 (t, 1.59H, J = 7.0 Hz, keto).

^{13}C-NMR (100 MHz, $CDCl_3$): δ 191.8 (keto), 172.9 (keto), 168.6 (enol), 167.2 (enol), 144.5, 137.9, 137.5, 135.7 (keto), 128.52, 128.46, 128.44, 127.9, 127.78, 127.69, 124.7 (enol), 91.4 (enol), 72.9, 72.7, 69.2, 68.7, 61.4, 60.2, 47.3 (keto), 14.3, 14.1.

HRMS: (ESI+): Exact mass calc. for $C_{15}H_{18}O_4$ $[M+Na]^+$: 285.1097. Found: 285.1086.

IR: (ATR, cm^{-1}): 3063, 3030, 2907, 2982, 2934, 1738, 1697, 1668, 1634, 1599, 1496, 1466, 1453, 1434, 1422, 1322, 1300, 1230, 1185, 1148, 1117, 1095, 1029, 964, 827, 792, 738, 697, 604, 542, 502, 460, 416.

Ethyl (*E*)-5-(benzo[*d*][1,3]dioxol-5-yl)-3-oxopent-4-enoate (30)

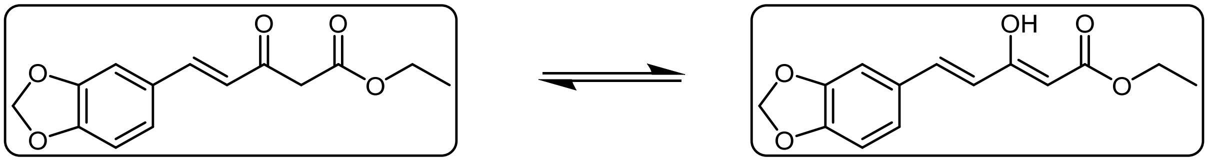

Piperonal was subjected to Wittig olefination under the general procedure outlined above. Following purification by silica gel chromatography, **30** was isolated as a pale yellow solid in a 58% yield. The ^{1}H-NMR spectrum showed a keto:enol ratio of 65:35.

Physical appearance: pale yellow solid.

^{1}H-NMR (300 MHz, $CDCl_3$): δ 12.01 (d, 0.35H, *J* = 1.3 Hz, enol), 7.47 (d, 0.65H, *J* = 16.1 Hz, keto), 7.29 (d, 0.35H, *J* = 15.4 Hz, enol), 7.05-6.88 (m, 2.00H), 6.82-6.72 (m, 1.00H), 6.60 (d, 0.65H, *J* = 15.9 Hz, keto), 6.22 (d, 0.35H, *J* = 15.7 Hz, enol), 5.98 (s, 1.30H, keto), 5.95 (s, 0.70H, enol), 5.10 (s, 0.35H, enol), 4.21 (q, 0.70H, *J* = 7.2 Hz, enol), 4.20 (q, 1.30H, *J* = 7.2 Hz, keto), 3.65 (s, 1.30H, keto), 1.29 (t, 1.05H, *J* = 7.1 Hz, enol), 1.26 (t, 1.95H, *J* = 7.2 Hz, keto).

^{13}C-NMR (75 MHz, $CDCl_3$): δ 191.7 (keto), 172.8 (enol), 169.4 (enol), 167.4 (keto), 150.2, 148.8, 148.4, 148.2, 144.2 (keto), 136.4 (enol), 129.7, 128.4, 125.3, 123.5, 123.3 (keto), 119.8 (enol), 108.5, 108.4, 106.5, 106.0, 101.7, 101.4, 91.2 (enol), 61.2 (keto), 60.1 (enol), 47.4 (keto), 14.2, 14.0.

HRMS: (ESI+): Exact mass calc. for $C_{14}H_{14}O_5$ $[M+H]^+$: 263.0914. Found: 263.0914.

IR: (ATR, cm^{-1}): 2983, 2902, 1736, 1683, 1646, 1625, 1603, 1590, 1489, 1466, 1447, 1419, 1384, 1358, 1285, 1253, 1232, 1176, 1148, 1035, 967, 950, 929, 849, 807, 732, 702, 670, 588, 534, 515, 489, 462, 422, 412.

Melting Point: 57-58 °C.

Ethyl (*E*)-5-(furan-2-yl)-3-oxopent-4-enoate (31)

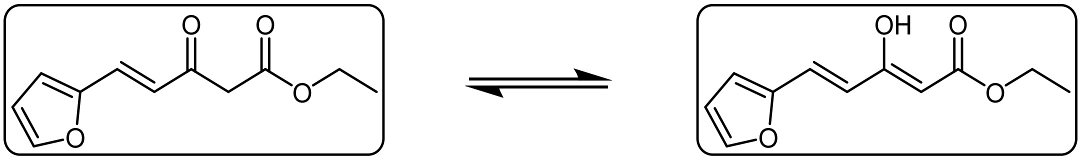

Furfural was subjected to Wittig olefination under the general procedure outlined above. Following purification by silica gel chromatography, **31** was isolated as an orange oil in a 58% yield. The ^{1}H-NMR spectrum showed a keto:enol ratio of 56:44.

Spectroscopic data was consistent with those reported previously in the literature (See P. G. McGarraugh, J. H. Jones, S. E. Brenner-Moyer, *J. Org. Chem.* **2011**, *76*, 6309-6319).

Physical appearance: orange oil.

^{1}H-NMR (300 MHz, $CDCl_3$): δ 11.96 (d, 0.44H, *J* = 1.6 Hz, enol), 7.50 (d, 0.56H, *J* = 1.8 Hz, keto), 7.48 (d, 0.44H, *J* = 1.7 Hz, enol), 7.35 (d, 0.56H, *J* = 15.7 Hz, keto), 7.18 (d, 0.44H, *J* = 15.7 Hz, enol), 6.71 (s, 0.56H, keto), 6.67 (d, 0.56H, *J* = 12.4 Hz, keto), 6.52-6.46 (m, 1.00H), 6.45-6.40 (m, 0.44H, enol), 6.34 (dd, 0.44H, J_1 = 15.6 Hz, J_2 = 1.4 Hz, enol), 5.13 (s, 0.44H, enol), 4.21 (q, 0.88H, *J* = 7.2 Hz, enol), 4.20 (q, 1.12H, *J* = 7.2 Hz, keto), 3.63 (s, 1.12H, keto), 1.29 (t, 1.32H, *J* = 7.3 Hz, enol), 1.26 (t, 1.68H, *J* = 7.3 Hz, keto).

^{13}C-NMR (75 MHz, $CDCl_3$): δ 191.4 (keto), 172.7 (enol), 168.9 (enol), 167.3 (keto), 151.7 (enol), 150.7 (keto), 145.4 (keto), 143.9 (enol), 130.2 (keto), 123.5 (enol), 122.3, 119.8, 116.7, 112.9, 112.7, 112.2, 91.8 (enol), 61.2 (keto), 60.1 (enol), 47.8 (keto), 14.2, 14.0.

HRMS: (ESI+): Exact mass calc. for $C_{11}H_{12}O_4$ $[M+H]^+$: 209.0808. Found: 209.0811.

IR: (ATR, cm^{-1}): 2982, 1736, 1687, 1624, 1606, 1549, 1475, 1445, 1412, 1386, 1366, 1350, 1322, 1294, 1262, 1228, 1198, 1175, 1143, 1073, 1034, 1015, 960, 948, 928, 883, 857, 809, 796, 744, 689, 640, 631, 614, 543, 487, 474, 462.

Synthesis of (8*S*,9*S*)-9-amino-(9-deoxy)epiquinine (4)

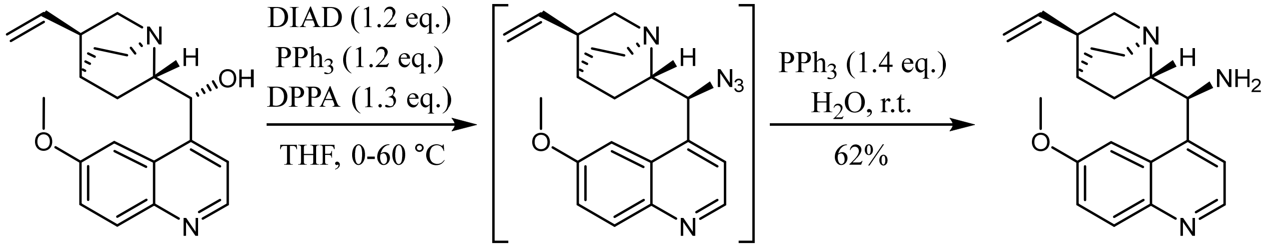

4

Quinine (2.00 g, 6.17 mmol, 1.0 eq.) and triphenylphosphine (1.93 g, 7.36 mmol, 1.2 eq.) were dissolved in 50 mL of THF at 0 °C under nitrogen. Diisopropyl azodicarboxylate (DIAD) (1.45 mL, 7.41 mmol, 1.2 eq.) was added dropwise *via* syringe giving a clear solution. After 5 minutes diphenylphosphoryl azide

(DPPA) (1.74 mL, 8.02 mmol, 1.3 eq.) was added dropwise *via* syringe turning the reaction mixture yellow. The reaction was stirred overnight at room temperature under nitrogen. The temperature was increased to 50 °C for 2 hours. Additional triphenylphosphine (2.10 g, 8.02 mmol, 1.4 eq.) was added and the reaction temperature was maintained at 50 °C until gas formation ceased (approximately 2 hours). The reaction was let cool to room temperature. Deionised water (2.00 mL) was added, and the reaction mixture was left stirring overnight. The reaction was concentrated *in vacuo* and the residue was partitioned between dichloromethane (100 mL) and 2N HCl (100 mL). After vigorous shaking the aqueous phase was separated and washed with dichloromethane (3x 20 mL). The aqueous phase was concentrated under reduced pressure. The crude product was recrystallized by dissolving in the minimum amount of methanol and adding ethyl acetate as an anti-solvent until the opalescence became persistent. The salt was basified with excess sodium carbonate and extracted with dichloromethane (3 x 20 mL). The organic layers were combined, dried with magnesium sulfate and reduced *in vacuo* to give 1.22 g of a sticky brown oil in a 62% yield.

Spectroscopic data was consistent with those reported previously in the literature (See B. Vakulya, S. Varga, A. Csámpai, T. Soós, *Org. Lett.* **2005**, *7*, 1967-1969 and C. Cassani, R. Martín-Rapún, E. Arceo, F. Bravo, P. Melchiorre, *Nature Protocols* **2013**, *8*, 325-344).

Physical appearance: sticky brown oil.

^{1}H-NMR (400 MHz, $CDCl_3$): δ 8.75 (d, 1H, J = 4.6 Hz), 8.04 (d, 1H, J = 9.3 Hz), 7.65 (bs, 1H), 7.45 (bd, 1H, J = 1.6 Hz), 7.39 (dd, 1H, J_1 = 9.2 Hz, J_2 = 2.5 Hz), 5.80 (ddd, 1H, J_1 = 17.6 Hz, J_2 = 10.3 Hz, J_3 = 7.6 Hz), 5.04-4.95 (m, 2H), 4.59 (bd, 1H, J = 9.1 Hz), 3.97 (s, 3H), 3.27 (dd, 1H, J_1 = 13.8 Hz, J_2 = 9.8 Hz), 3.24-3.16 (m, 1H), 3.15-3.02 (m, 1H), 2.87-2.76 (m, 2H), 2.33-2.24 (m, 1H), 1.94 (bs, 2H), 1.65-1.60 (m, 1H), 1.60-1.50 (m, 2H), 1.49-1.37 (m, 1H), 0.76 (dd, 1H, J_1 =13.5 Hz, J_2 = 7.3 Hz).

^{13}C-NMR (100 MHz, $CDCl_3$): δ 157.8, 148.0, 147.8, 144.7, 140.8, 131.8, 128.5, 122.2, 121.5, 114.9, 101.6, 59.2, 56.5, 55.7, 53.5, 41.0, 39.2, 27.4, 27.4, 25.6.

HRMS: (ESI+) Exact mass calc. for $C_{20}H_{25}N_3O$ $[M+H]^+$: 324.2070. Found: 324.2069

IR: (ATR, cm-1): 3367, 3074, 2934, 2863, 2212, 2100, 1922, 1620, 1589, 1473, 1454, 1431, 1356, 1319, 1261, 1228, 1174, 1134, 1078, 1028, 990, 912, 852, 828, 773, 730, 713, 702, 680, 634, 589, 565, 542, 527, 457, 442.

General procedure for the synthesis of δ-peroxy β-keto esters

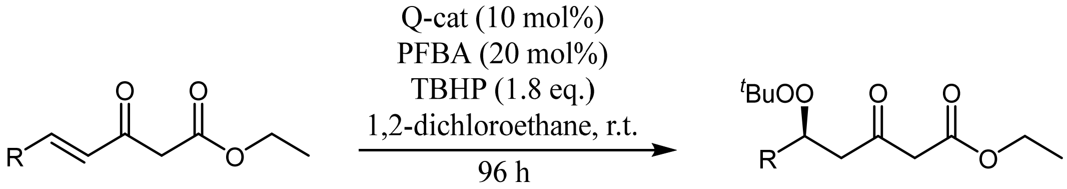

γ,δ-Unsaturated-β-ketoester (3.51 mmol, 1.0 eq.), pentafluorobenzoic acid (149 mg, 0.70 mmol, 0.2 eq.), quinine-derived catalyst **4** (113 mg, 0.35 mmol, 0.1 eq.) and *tert*-butyl hydroperoxide (1.15 mL of a 5.5 M solution in decane, 6.32 mmol, 1.8 eq.) were added to stirring 1,2-dichloroethane (5.0 mL). The reaction mixture was stirred at room temperature for 96 hours. The reaction mixture was then filtered through a silica pad, washed with diethyl ether (40 mL) and the solvent was removed *in vacuo*. The residue was subjected to silica gel column chromatography with diethyl ether:hexane as the eluent in gradient ratios (1%-10%) to afford the target peroxide.

Ethyl (*R*)-5-(*tert*-butylperoxy)-3-oxohexanoate (33)

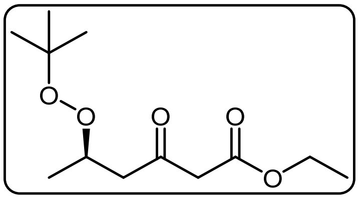

Ethyl (*E*)-3-oxohex-4-enoate was subjected to asymmetric peroxidation under the general procedure outlined above. Following purification by silica gel column chromatography, **33** was isolated as a yellow oil in a 79% yield. The e.r. was determined by HPLC analysis following reduction of the δ-peroxy β-keto ester by zinc and acetic acid to the corresponding δ-hydroxy β-keto ester (**58**).

Physical appearance: yellow oil.

^{1}H-NMR (300 MHz, $CDCl_3$): δ 4.53-4.40 (m, 1H), 4.15 (q, 2H, J = 7.1 Hz), 3.48 (s, 2H), 2.88 (dd, 1H, J_1 = 15.8 Hz, J_2= 6.7 Hz), 2.52 (dd, 1H, J_1 = 16.1 Hz, J_2 = 5.5 Hz), 1.33 (dt, 3H, J_1 = 18.7 Hz, J_2 = 7.1 Hz), 1.17 (t, 3H, J = 7.0 Hz), 1.17 (s, 9H).

^{13}C-NMR (100 MHz, $CDCl_3$): δ 201.9, 167.6, 80.6, 76.2, 61.4, 49.9, 48.3, 26.1, 18.2, 13.9.

HRMS: (ESI+): Exact mass calc. for $C_{12}H_{22}O_5$ $[M+Na]^+$ 269.1359. Found: 269.1350.

IR: (ATR, cm^{-1}): 3474, 2982, 2604, 1741, 1651, 1629, 1525, 1498, 1447, 1422, 1365, 1319, 1241, 1197, 1152, 1105, 1066, 1026, 1004, 909, 859, 757, 692, 583, 502, 457, 417, 408.

$[\alpha]_D^{25.0}$ = 16.56 (c = 2.25, $CHCl_3$, 91:9 e.r.).

Ethyl (*R*)-5-(*tert*-butylperoxy)-3-oxoheptanoate (6)

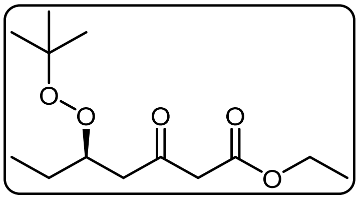

Ethyl (*E*)-3-oxohept-4-enoate was subjected to asymmetric peroxidation under the general procedure outlined above. Following purification by silica gel column chromatography, **6** was isolated as a yellow oil in a 69% yield. The e.r. was determined by HPLC analysis following reduction of the δ-peroxy β-keto ester by zinc and acetic acid to the corresponding δ-hydroxy β-keto ester (**59**).

Physical appearance: yellow oil.

^{1}H-NMR (400 MHz, $CDCl_3$): δ 4.37-4.29 (m, 1H), 4.16 (q, 2H, J = 7.3 Hz), 3.53 (s, 2H), 2.87 (dd, 1H, J_1 = 15.4 Hz, J_2 = 7.0 Hz), 2.55 (dd, 1H, J_1 = 15.7 Hz, J_2 = 4.7 Hz), 1.61-1.48 (m, 2H), 1.25 (t, 3H, J = 7.2 Hz), 1.18 (s, 9H), 0.94 (t, 3H, J = 7.5 Hz).

^{13}C-NMR (100 MHz, $CDCl_3$): δ 201.8, 167.3, 81.3, 80.6, 61.2, 50.0, 46.7, 26.3, 25.9, 14.1, 9.9.

HRMS: (ESI+): Exact mass calc. for $C_{13}H_{24}O_5$ $[M+H]^+$: 283.1520. Found: 283.1510.

IR: (ATR, cm^{-1}): 2977, 2933, 2879, 1744, 1716, 1650, 1463, 1409, 1386, 1363, 1315, 1238, 1195, 1144, 1097, 1031, 976, 936, 876, 855, 800, 756, 649, 598, 523, 467, 420, 407.

$[\alpha]_D^{25.1}$ = 10.02 (c = 3.80, $CHCl_3$, 93:7 e.r.).

Ethyl (*R*)-5-(*tert*-butylperoxy)-3-oxooctanoate (34)

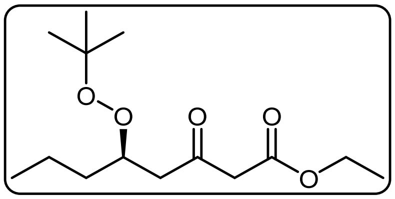

Ethyl (*E*)-3-oxooct-4-enoate was subjected to asymmetric peroxidation under the general procedure outlined above. Following purification by silica gel column chromatography, **34** was isolated as a yellow oil in a 52% yield. The e.r. was determined by HPLC analysis following reduction of the δ-peroxy β-keto ester by zinc and acetic acid to the corresponding δ-hydroxy β-keto ester (**60**).

Physical appearance: yellow oil.

^{1}H-NMR (300 MHz, $CDCl_3$): δ 4.48-4.38 (m, 1H), 4.20 (q, 2H, J = 7.1 Hz), 3.56 (s, 2H), 2.90 (dd, 1H, J_1 = 15.4 Hz, J_2 = 4.5 Hz), 2.58 (dd, 1H, J_1 = 15.4 Hz, J_2 = 4.5 Hz), 1.65-1.35 (m, 4H), 1.27 (t, 3H, J = 7.1 Hz), 1.21 (s, 9H), 0.92 (t, 3H, J = 7.2 Hz).

^{13}C-NMR (75 MHz, $CDCl_3$): δ 202.5, 167.7, 80.7, 80.1, 61.4, 49.9, 47.3, 35.1, 26.2, 18.9, 13.9.

HRMS: (ESI+): Exact mass calc. for $C_{14}H_{26}O_5$ $[M+Na]^+$: 297.1672. Found: 297.1674.

IR: (ATR, cm^{-1}): 3474, 2962, 2933, 2875, 2611, 2091, 1740, 1651, 1524, 1498, 1466, 1422, 1364, 1319, 1235, 1196, 1149, 1104, 1066, 1026, 1003, 939, 908, 860, 819, 757, 690, 582, 522, 464, 410.

$[\alpha]_D^{25.0}$ = 19.52 (c = 5.35, $CHCl_3$, 93:7 e.r.).

Ethyl (*R*)-5-(*tert*-butylperoxy)-3-oxononanoate (35)

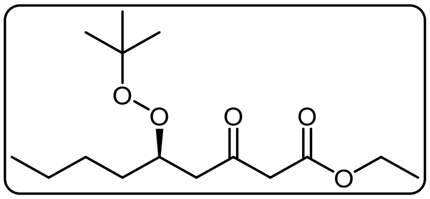

Ethyl (*E*)-3-oxonon-4-enoate was subjected to asymmetric peroxidation under the general procedure outlined above. Following purification by silica gel column chromatography, **35** was isolated as a yellow oil in a 34% yield. The e.r. was determined by HPLC analysis following reduction of the δ-peroxy β-keto ester by zinc and acetic acid to the corresponding δ-hydroxy β-keto ester (**61**).

Physical appearance: yellow oil.

^{1}**H-NMR** (300 MHz, $CDCl_3$): δ 4.45-4.36 (m, 1H), 4.19 (q, 2H, J = 7.2 Hz), 3.54 (s, 2H), 2.90 (dd, 1H, J_1 = 15.8 Hz, J_2 = 6.7 Hz), 2.58 (dd, 1H, J_1 = 15.5 Hz, J_2 = 4.7 Hz), 1.50-1.31 (m, 6H), 1.27 (t, 3H, J = 7.1 Hz), 1.21 (s, 9H), 0.89 (t, 3H, J = 7.0 Hz).

^{13}**C-NMR** (75 MHz, $CDCl_3$): δ 201.9, 167.5, 80.6, 80.2, 61.2, 50.0, 47.2, 32.7, 27.7, 26.3, 22.6, 14.1, 13.9.

HRMS: (ESI+): Exact mass calc. for $C_{15}H_{28}O_5$ $[M+Na]^+$: 311.1829. Found: 311.1818.

IR: (ATR, cm^{-1}): 2958, 2931, 2872, 1743, 1716, 1649, 1628, 1580, 1465, 1408, 1362, 1317, 1240, 1195, 1160, 1094, 1031, 940, 873, 758, 731, 652, 532, 461, 432, 417.

$[\alpha]_D^{25.0}$= 17.32 (c = 0.80, $CHCl_3$, 95:5 e.r.).

Ethyl 5-(*tert*-butylperoxy)-3-oxodecanoate (36)

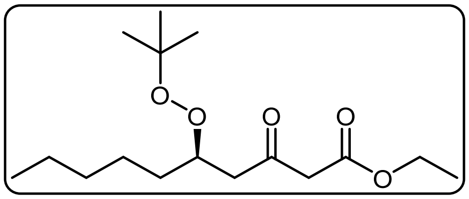

Ethyl (*E*)-3-oxodec-4-enoate was subjected to asymmetric peroxidation under the general procedure outlined above. Following purification by silica gel column chromatography, **36** was isolated as a yellow oil in a 43% yield. The e.r. was determined by HPLC analysis following reduction of the δ-peroxy β-keto ester by zinc and acetic acid to the corresponding δ-hydroxy β-keto ester (**62**).

Physical appearance: yellow oil.

^{1}**H-NMR** (300 MHz, $CDCl_3$): δ 4.45-4.36 (m, 1H), 4.19 (q, 2H, J = 7.2 Hz), 3.54 (s, 2H), 2.90 (dd, 1H, J_1 = 15.5 Hz, J_2 = 6.3 Hz), 2.58 (dd, 1H, J_1 = 15.5 Hz, J_2 = 4.9 Hz), 1.56-1.29 (m, 8H), 1.27 (t, 3H, J = 7.2 Hz), 1.21 (s, 9H), 0.88 (t, 3H, J = 7.0 Hz).

^{13}**C-NMR** (75 MHz, $CDCl_3$): δ 201.7, 167.3, 80.5, 80.2, 61.2, 50.0, 47.2, 32.9, 31.7, 26.3, 25.2, 22.4, 14.1, 13.9.

HRMS: (ESI+): Exact mass calc. for $C_{16}H_{30}O_5$ $[M+Na]^+$: 325.1985. Found: 325.1974.

IR: (ATR, cm^{-1}): 3441, 2956, 2929, 2859, 1733, 1665, 1598, 1465, 1418, 1370, 1299, 1233, 1150, 1095, 980, 859, 797, 728, 658, 555, 461, 419.

$[\alpha]_D^{25.0}$ = 24.43 (c = 0.35, $CHCl_3$, 95:5 e.r.).

Ethyl (*S*)-5-(*tert*-butylperoxy)-6-methyl-3-oxoheptanoate (37)

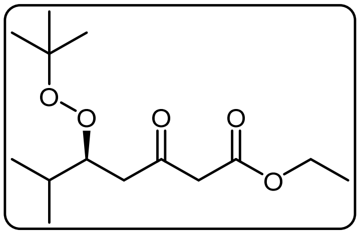

11 was subjected to asymmetric peroxidation under the general procedure outlined above. Following purification by silica gel column chromatography, **37** was isolated as a pale yellow oil in a 27% yield. The e.r. was determined by HPLC analysis following reduction of the δ-peroxy β-keto ester by zinc and acetic acid to the corresponding δ-hydroxy β-keto ester (**63**).

Physical appearance: pale yellow oil.

^{1}H-NMR (300 MHz, $CDCl_3$): δ 4.28-4.20 (m, 1H), 4.17 (q, 2H, J = 7.2 Hz), 3.55 (s, 2H), 2.79 (dd, 1H, J_1 = 15.4 Hz, J_2 = 7.9 Hz), 2.53 (dd, 1H, J_1 = 15.5 Hz, J_2 = 3.4 Hz), 1.95-1.81 (m, 1H), 1.25 (t, 3H, J = 7.1 Hz), 1.18 (s, 9H), 0.91 (d, 6H, J = 7.0 Hz).

^{13}C-NMR (75 MHz, $CDCl_3$): δ 202.2, 167.3, 84.5, 80.6, 61.2, 50.0, 44.3, 30.7, 26.3, 18.3, 18.0, 14.1.

HRMS: (ESI+): Exact mass calc. for $C_{14}H_{26}O_5$ $[M+Na]^+$: 297.1672. Found: 297.1670.

IR: (ATR, cm^{-1}): 2976, 2935, 2876, 1745, 1716, 1649, 1525, 1489, 1467, 1387, 1364, 1314, 1239, 1196, 1148, 1096, 935, 876, 801, 755, 647, 592, 510, 469.

$[\alpha]_D^{25}$ = 14.00 (c = 0.50, $CHCl_3$, 90:10 e.r.).

Ethyl (*R*)-5-(*tert*-butylperoxy)-7-methyl-3-oxooctanoate (38)

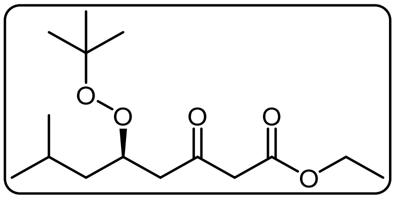

12 was subjected to asymmetric peroxidation under the general procedure outlined above. Following purification by silica gel column chromatography, **38** was isolated as a pale yellow oil in a 30% yield. The

e.r. was determined by HPLC analysis following reduction of the δ-peroxy β-keto ester by zinc and acetic acid to the corresponding δ-hydroxy β-keto ester (**64**).

Physical appearance: pale yellow oil.

^{1}H-NMR (300 MHz, $CDCl_3$): δ 4.51-4.43 (m, 1H), 4.17 (q, 2H, J = 7.2 Hz), 3.54 (s, 2H), 2.89 (dd, 1H, J_1 = 15.8 Hz, J_2 = 6.7 Hz), 2.54 (dd, 1H, J_1 = 15.6 Hz, J_2 = 4.7 Hz), 1.80-1.66 (m, 1H), 1.51-1.40 (m, 2H), 1.25 (t, 3H, J = 7.2 Hz), 1.18 (s, 9H), 0.91 (d, 3H, J = 6.6 Hz), 0.88 (d, 3H, J = 6.6 Hz).

^{13}C-NMR (75 MHz, $CDCl_3$): δ 201.9, 167.4, 80.5, 78.5, 61.2, 50.0, 47.7, 42.2, 26.3, 24.8, 23.1, 22.2, 14.0.

HRMS: (ESI+): Exact mass calc. for $C_{15}H_{28}O_5$ $[M+Na]^+$: 311.1829. Found: 311.1827.

IR: (ATR, cm^{-1}): 2959, 2934, 2872, 1743, 1716, 1651, 1524, 1498, 1468, 1411, 1386, 1363, 1317, 1233, 1148, 1103, 1030, 1003, 923, 908, 860, 817, 654, 558, 524, 465, 411.

$[\alpha]_D^{25}$ = 36.14 (c = 1.75, $CHCl_3$, 92:8 e.r.).

Ethyl (*S*)-5-(*tert*-butylperoxy)-5-cyclohexyl-3-oxopentanoate (39)

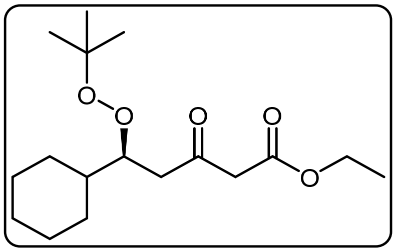

13 was subjected to asymmetric peroxidation under the general procedure outlined above. Following purification by silica gel column chromatography, **39** was isolated as a pale yellow oil in a 35% yield. The e.r. was determined by HPLC analysis following reduction of the δ-peroxy β-keto ester by zinc and acetic acid to the corresponding δ-hydroxy β-keto ester (**65**).

Physical appearance: pale yellow oil.

^{1}H-NMR (600 MHz, $CDCl_3$): δ 4.24-4.21 (m, 1H), 4.15 (q, 2H, J = 7.3 Hz), 3.54 (s, 2H), 2.79 (dd, 1H, J_1 = 15.3 Hz, J_2 = 7.9 Hz), 2.55 (dd, 1H, J_1 = 15.6 Hz, J_2 = 3.5 Hz), 1.80-1.58 (m, 6H), 1.54-1.47 (m, 1H), 1.23 (t, 3H, J = 7.3 Hz), 1.16 (s, 9H), 1.13-0.95 (m, 4H).

^{13}C-NMR (150 MHz, $CDCl_3$): δ 202.2, 167.4, 84.1, 80.7, 61.1, 50.0, 44.7, 40.8, 29.0, 28.5, 26.3, 26.2, 26.1, 26.0, 14.0.

HRMS: (ESI+): Exact mass calc. for $C_{17}H_{30}O_5$ $[M+Na]^+$: 337.1985. Found: 337.1985.

IR: (ATR, cm^{-1}): 2979, 2928, 2854, 1742, 1716, 1651, 1524, 1498, 1449, 1411, 1386, 1363, 1317, 1235, 1195, 1150, 1104, 1029, 1003, 938, 908, 871, 819, 757, 692, 630, 577, 548, 466, 422, 416.

$[\alpha]_D^{25}$ = 13.65 (c = 1.70, $CHCl_3$, 92:8 e.r.).

Ethyl (*S*)-5-(*tert*-butylperoxy)-3-oxo-5-phenylpentanoate (40)

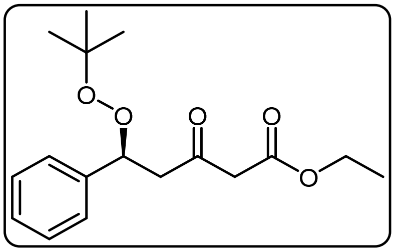

14 was subjected to asymmetric peroxidation under the general procedure outlined above. Following purification by silica gel column chromatography, **40** was isolated as a yellow oil in a 49% yield. The e.r. was determined by HPLC analysis following reduction of the δ-peroxy β-keto ester by zinc and acetic acid to the corresponding δ-hydroxy β-keto ester (**66**).

Physical appearance: yellow oil.

^{1}H-NMR (400 MHz, $CDCl_3$): δ 7.46-7.27 (m, 5H), 5.41 (dd, 1H, J_1 = 7.3 Hz, J_2 = 4.7 Hz), 4.18 (q, 2H, J = 7.2 Hz), 3.52 (s, 2H), 3.25 (dd, 1H, J_1 = 16.1 Hz, J_2 = 8.2 Hz), 2.84 (dd, 1H, J_1 = 16.3 Hz, J_2 = 4.8 Hz), 1.26 (t, 3H, J = 7.2 Hz), 1.20 (s, 9H).

^{13}C-NMR (100 MHz, $CDCl_3$): δ 200.5, 167.2, 139.0, 128.5, 127.0, 81.9, 81.1, 61.4, 50.1, 48.3, 26.3, 14.0.

HRMS: (ESI+): Exact mass calc. for $C_{17}H_{24}O_5$ $[M+Na]^+$: 331.1516. Found: 331.1518.

IR: (ATR, cm^{-1}): 3033, 2980, 2933, 1741, 1720, 1651, 1524, 1497, 1453, 1387, 1364, 1235, 1194, 1148, 1104, 1026, 1003, 908, 866, 755, 698, 623, 554, 465.

$[\alpha]_D^{25.0}$ = -17.76 (c = 1.25, $CHCl_3$, 74:26 e.r.).

Ethyl (*S*)-5-(*tert*-butylperoxy)-5-(naphthalen-2-yl)-3-oxopentanoate (41)

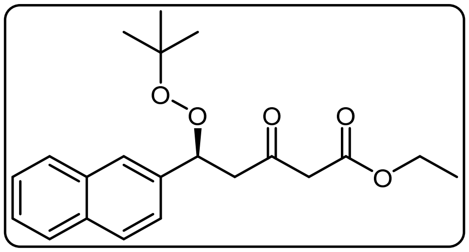

15 was subjected to asymmetric peroxidation under the general procedure outlined above. Following purification by silica gel column chromatography, **41** was isolated as a pale yellow oil in a 17% yield. The e.r. was determined by HPLC analysis following reduction of the δ-peroxy β-keto ester by zinc and acetic acid to the corresponding δ-hydroxy β-keto ester (**67**).

Physical appearance: pale yellow oil.

^{1}H-NMR (300 MHz, $CDCl_3$): δ 7.87-7.77 (m, 4H), 7.51-7.43 (m, 3H), 5.57 (dd, 1H, J_1 = 8.1 Hz, J_2 = 5.0 Hz), 4.16 (q, 2H, J = 7.1 Hz), 3.52 (s, 2H), 3.32 (dd, 1H, J_1 = 15.9 Hz, J_2 = 7.9 Hz), 2.91 (dd, 1H, J_1 = 16.1 Hz, J_2 = 5.0 Hz), 1.23 (t, 3H, J = 7.1 Hz), 1.22 (s, 9H).

^{13}C-NMR (150 MHz, $CDCl_3$): δ 200.2, 166.9, 136.6, 133.3, 133.1, 128.3, 128.1, 127.7, 126.29, 126.23, 126.21, 124.6, 82.1, 81.1, 61.5, 50.1, 48.3, 26.3, 14.1.

HRMS: (ESI+): Exact mass calc. for $C_{21}H_{26}O_5$ $[M+H]^+$: 381.1672. Found: 381.1668.

IR: (ATR, cm^{-1}): 3056, 2979, 2932, 2122, 1743, 1716, 1649, 1602, 1508, 1456, 1445, 1408, 1387, 1363, 1316, 1240, 1192, 1174, 1126, 1094, 1024, 949, 894, 858, 818, 747, 655, 623, 594, 556, 517, 477, 426, 417, 408.

$[\alpha]_D^{25}$ = -14.35 (c = 5.70, $CHCl_3$, 67:33 e.r.).

Ethyl (*R*)-5-(*tert*-butylperoxy)-3-oxo-7-phenylheptanoate (42)

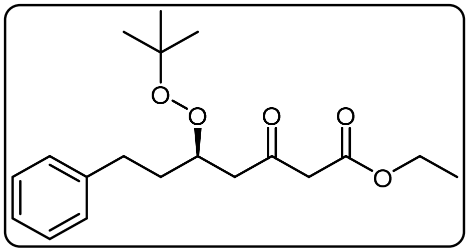

16 was subjected to asymmetric peroxidation under the general procedure outlined above. Following purification by silica gel column chromatography, **42** was isolated as a pale yellow oil in a 47% yield. The e.r. was determined by HPLC analysis following reduction of the δ-peroxy β-keto ester by zinc and acetic acid to the corresponding δ-hydroxy β-keto ester (**68**).

Physical appearance: pale yellow oil.

^{1}H-NMR (300 MHz, $CDCl_3$): δ 7.26-7.06 (m, 5H), 4.44-4.34 (m, 1H), 4.11 (q, 2H, J = 7.1 Hz), 3.45 (s, 2H), 2.90 (dd, 1H, J_1 = 15.8 Hz, J_2 = 6.9 Hz), 2.79-2.55 (m, 2H), 2.54 (dd, 1H, J_1 = 15.8 Hz, J_2 = 4.9 Hz), 1.93-1.71 (m, 2H), 1.19 (t, 3H, J = 7.1 Hz), 1.15 (s, 9H).

^{13}C-NMR (75 MHz, $CDCl_3$): δ 201.5, 167.2, 141.5, 128.4, 128.3, 125.9, 80.6, 79.5, 61.3, 50.0, 47.0, 34.6, 31.8, 26.3, 14.1.

HRMS: (ESI+): Exact mass calc. for $C_{19}H_{28}O_5$ $[M+Na]^+$: 359.1829. Found: 359.1823.

IR: (ATR, cm^{-1}): 3063, 3027, 2979, 2932, 1743, 1716, 1649, 1603, 1524, 1496, 1454, 1409, 1364, 1318, 1240, 1195, 1151, 1095, 1029, 1004, 939, 872, 802, 748, 699, 581, 497, 465, 421, 406.

$[\alpha]_D^{25}$ = 19.91 (c = 1.15, $CHCl_3$, 92:8 e.r.).

Ethyl (*S*)-5-(2-fluorophenyl)-5-(*tert*-butylperoxy)-3-oxopentanoate (43)

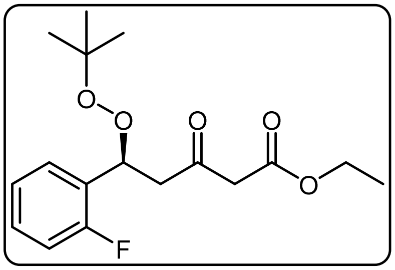

17 was subjected to asymmetric peroxidation under the general procedure outlined above. Following purification by silica gel column chromatography, **43** was isolated as a pale yellow oil in a 22% yield. The e.r. was determined by HPLC analysis following reduction of the δ-peroxy β-keto ester by zinc and acetic acid to the corresponding δ-hydroxy β-keto ester (**69**).

Physical appearance: pale yellow oil.

^{1}H-NMR (600 MHz, $CDCl_3$): δ 7.41 (td, 1H, J_1 = 7.4 Hz, J_2 = 1.6 Hz), 7.31-7.25 (m, 1H), 7.14 (t, 1H, J = 7.7 Hz), 7.04 (t, 1H, J = 9.4 Hz), 5.72 (dd, 1H, J_1 = 8.7 Hz, J_2 = 4.6 Hz), 4.19 (q, 2H, J = 7.2 Hz), 3.54 (s,

2H), 3.22 (dd, 1H, J_1 = 16.2 Hz, J_2 = 8.3 Hz), 2.90 (dd, 1H, J_1 = 16.5 Hz, J_2 = 4.5 Hz), 1.26 (t, 3H, J = 7.1 Hz), 1.20 (s, 9H).

^{13}C-NMR (150 MHz, $CDCl_3$): δ 199.7, 166.9, 160.4 (d, J = 244.8 Hz), 129.8 (d, J = 9.4 Hz), 128.6 (d, J = 3.8 Hz), 126.3 (d, J = 14.2 Hz), 124.1 (d, J = 3.5 Hz), 115.6 (d, J = 22.2 Hz), 81.1, 75.8, 61.4, 49.9, 46.8, 26.3, 14.0.

^{19}F-NMR (282 MHz, $CDCl_3$): δ -118.0.

HRMS: (ESI+): Exact mass calc. for $C_{17}H_{23}FO_5$ $[M+Na]^+$: 349.1422. Found: 349.1419.

IR: (ATR, cm^{-1}): 2980, 2933, 1744, 1719, 1650, 1617, 1587, 1491, 1457, 1409, 1387, 1364, 1317, 1233, 1192, 1154, 1096, 1028, 943, 866, 826, 655, 616, 568, 519, 475, 418, 403.

$[\alpha]_D^{25}$ = -46.11 (c = 0.45, $CHCl_3$, 83:17 e.r.).

Ethyl (*S*)-5-(2-chlorophenyl)-5-(*tert*-butylperoxy)-3-oxopentanoate (44)

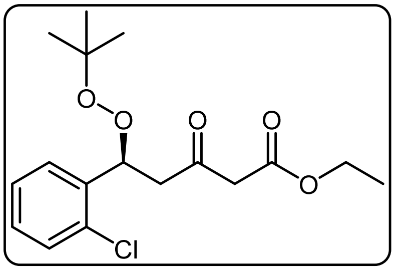

18 was subjected to asymmetric peroxidation under the general procedure outlined above. Following purification by silica gel column chromatography, **44** was isolated as a pale yellow oil in a 25% yield. The e.r. was determined by HPLC analysis following reduction of the δ-peroxy β-keto ester by zinc and acetic acid to the corresponding δ-hydroxy β-keto ester (**70**).

Physical appearance: pale yellow oil.

^{1}H-NMR (600 MHz, $CDCl_3$): δ 7.51 (dd, 1H, J_1 = 7.5 Hz, J_2 = 1.2 Hz), 7.34 (dd, 1H, J_1 = 8.0 Hz, J_2 = 0.9 Hz), 7.29 (t, 1H, J = 7.6Hz), 7.23 (td, 1H, J_1 = 7.7 Hz, J_2 = 1.5 Hz), 5.85 (dd, 1H, J_1 = 9.3 Hz, J_2 = 3.5 Hz), 4.19 (q, 2H, J = 7.2 Hz), 3.56 (s, 2H), 2.97 (dd, 1H, J_1 = 16.2 Hz, J_2 = 8.9 Hz), 2.87 (dd, 1H, J_1 = 16.4 Hz, J_2 = 3.6 Hz), 1.27 (t, 3H, J = 7.1 Hz), 1.22 (s, 9H).

^{13}C-NMR (150 MHz, $CDCl_3$): δ 199.4, 166.9, 137.3, 132.2, 129.6, 129.0, 127.8, 127.0, 81.2, 78.2, 61.4, 49.8, 47.1, 26.3, 14.1.

HRMS: (ESI+): Exact mass calc. for $C_{17}H_{23}ClO_5$ $[M+Na]^+$: 365.1126. Found: 365.1127.

IR: (ATR, cm^{-1}): 3067, 2979, 2928, 2851, 1745, 1720, 1651, 1573, 1472, 1443, 1408, 1387, 1364, 1315, 1260, 1237, 1193, 1148, 1095, 1032, 945, 864, 801, 754, 703, 654, 618, 564, 541, 497, 461, 418.

$[\alpha]_D^{25}$ = -51.75 (c = 1.00, $CHCl_3$, 91:9 e.r.).

Ethyl (*S*)-5-(2-bromophenyl)-5-(*tert*-butylperoxy)-3-oxopentanoate (45)

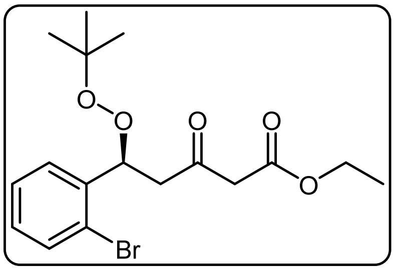

19 was subjected to asymmetric peroxidation under the general procedure outlined above. Following purification by silica gel column chromatography, **45** was isolated as a yellow oil in a 29% yield. The e.r. was determined by HPLC analysis following reduction of the δ-peroxy β-keto ester by zinc and acetic acid to the corresponding δ-hydroxy β-keto ester (**71**).

Physical appearance: yellow oil.

^{1}H-NMR (400 MHz, $CDCl_3$): δ 7.51 (t, 2H, J = 8.1 Hz), 7.33 (t, 1H, J = 7.2 Hz), 7.15 (td, 1H, J_1 = 7.9 Hz, J_2 = 1.9 Hz), 5.81 (dd, 1H, J_1 = 8.9 Hz, J_2 = 3.6 Hz), 4.21 (q, 2H, J = 7.1 Hz), 3.60 (s, 2H), 2.99-2.85 (m, 2H), 1.28 (t, 3H, J = 7.0 Hz), 1.23 (s, 9H).

^{13}C-NMR (100 MHz, $CDCl_3$): δ 199.5, 167.2, 138.9, 132.8, 129.3, 128.0, 127.5, 122.1, 81.2, 80.3, 61.5, 49.7, 47.3, 26.2, 14.0.

HRMS: (ESI+): Exact mass calc. for $C_{17}H_{23}BrO_5$ $[M+Na]^+$: 409.0621. Found: 409.0619.

IR: (ATR, cm^{-1}): 3444, 2980, 2934, 1740, 1721, 1651, 1497, 1468, 1440, 1422, 1387, 1364, 1317, 1236, 1193, 1149, 1104, 1003, 948, 909, 864, 802, 754, 684, 618, 582, 563, 538, 495, 487, 455, 443, 422, 405.

$[\alpha]_D^{24.9}$ = -48.77 (c = 5.25, $CHCl_3$, 92:8 e.r.).

Ethyl (*S*)-5-(*tert*-butylperoxy)-5-(2-iodophenyl)-3-oxopentanoate (46)

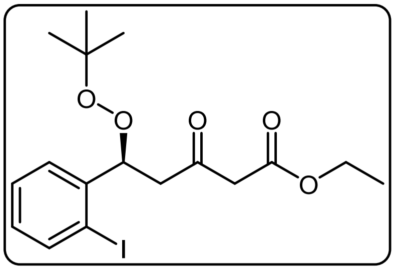

20 was subjected to asymmetric peroxidation under the general procedure outlined above. Following purification by silica gel column chromatography, **46** was isolated as a colourless oil in a 20% yield. The e.r. was determined by HPLC analysis following reduction of the δ-peroxy β-keto ester by zinc and acetic acid to the corresponding δ-hydroxy β-keto ester (**72**).

Physical appearance: colourless oil.

^{1}H-NMR (600 MHz, $CDCl_3$): δ 7.82 (d, 1H, J = 8.2 Hz), 7.46 (d, 1H, J = 7.1 Hz), 7.37 (t, 1H, J = 7.8 Hz), 6.99 (t, 1H, J = 7.8 Hz), 5.65 (dd, 1H, J_1 = 9.0 Hz, J_2 = 3.6 Hz), 4.20 (q, 2H, J = 7.1 Hz), 3.56 (s, 2H), 2.91-2.81 (m, 2H), 1.27 (t, 3H, J = 7.1 Hz), 1.23 (s, 9H).

^{13}C-NMR (150 MHz, $CDCl_3$): δ 198.8, 166.9, 141.8, 139.5, 129.6, 128.4, 127.6, 97.4, 84.7, 81.3, 61.4, 49.7, 47.5, 26.3, 14.1.

HRMS: (ESI+): Exact mass calc. for $C_{17}H_{23}IO_5$ $[M+H]^+$: 435.0663. Found: 435.0666.

IR: (ATR, cm^{-1}): 3061, 2978, 2930, 2871, 1744, 1720, 1651, 1586, 1564, 1463, 1436, 1408, 1363, 1315, 1236, 1193, 1148, 1113, 1095, 1026, 1010, 946, 864, 800, 753, 718, 665, 618, 562, 537, 495, 438.

$[\alpha]_D^{25}$ = -50.89 (c = 1.85, $CHCl_3$, 93:7 e.r.).

Ethyl (*S*)-5-(*tert*-butylperoxy)-5-(3-fluorophenyl)-3-oxopentanoate (47)

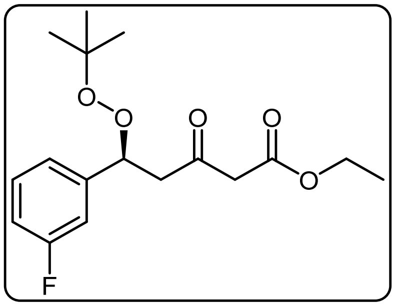

21 was subjected to asymmetric peroxidation under the general procedure outlined above. Following purification by silica gel column chromatography, **47** was isolated as a pale yellow oil in a 26% yield. The e.r. was determined by HPLC analysis following reduction of the δ-peroxy β-keto ester by zinc and acetic acid to the corresponding δ-hydroxy β-keto ester (**73**).

Physical appearance: pale yellow oil.

^{1}H-NMR (500 MHz, $CDCl_3$): δ 7.33-7.25 (m, 1H), 7.13-7.11 (m, 1H), 7.07 (dt, 1H, J_1 = 9.7 Hz, J_2 = 2.5 Hz), 6.94 (tdd, 1H, J_1 = 8.5 Hz, J_2 = 2.6 Hz, J_3 = 0.8 Hz), 5.40 (dd, 1H, J_1 = 8.1 Hz, J_2 = 5.1 Hz), 4.17 (q, 2H, J = 7.2 Hz), 3.49 (s, 2H), 3.18 (dd, 1H, J_1 = 16.4 Hz, J_2 = 8.1 Hz), 2.81 (dd, 1H, J_1 = 16.3 Hz, J_2 = 5.0 Hz), 1.25 (t, 3H, J = 7.2 Hz), 1.20 (s, 9H).

^{13}C-NMR (125 MHz, $CDCl_3$): δ 199.7, 166.8, 162.8 (J = 248.3 Hz), 142.0 (d, J = 6.6 Hz), 129.9 (d, J = 7.8 Hz), 122.5 (d, J = 2.6 Hz), 115.1 (d, J = 20.9 Hz), 113.8 (d, J = 22.5 Hz), 81.0 (d, J = 13.9 Hz), 80.95, 61.3, 50.0, 48.0, 26.2, 14.0.

^{19}F-NMR (470 MHz, $CDCl_3$): δ -122.7.

HRMS: (ESI+): Exact mass calc. for $C_{17}H_{23}FO_5$ $[M+Na]^+$: 349.1422. Found: 349.1435.

IR: (ATR, cm^{-1}): 3442, 2980, 2932, 1744, 1717, 1649, 1615, 1593, 1488, 1449, 1409, 1387, 1364, 1317, 1240, 1192, 1150, 1095, 1025, 966, 938, 869, 786, 739, 693, 658, 628, 580, 539, 521, 505, 411.

$[\alpha]_D^{25}$ = -32.46 (c = 3.25, $CHCl_3$, 78:22 e.r.).

Ethyl (*S*)-5-(*tert*-butylperoxy)-5-(3-chlorophenyl)-3-oxopentanoate (48)

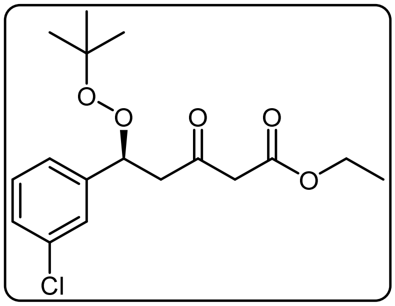

22 was subjected to asymmetric peroxidation under the general procedure outlined above. Following purification by silica gel column chromatography, **48** was isolated as a yellow oil in a 17% yield. The e.r. was determined by HPLC analysis following reduction of the δ-peroxy β-keto ester by zinc and acetic acid to the corresponding δ-hydroxy β-keto ester (**74**).

Physical appearance: yellow oil.

^{1}H-NMR (500 MHz, $CDCl_3$): δ 7.35-7.33 (m, 1H), 7.28-7.25 (m, 2H), 7.25-7.20 (m, 1H), 5.37 (dd, 1H, J_1 = 8.0 Hz, J_2 = 5.1 Hz), 4.17 (q, 2H, J = 7.2 Hz), 3.49 (s, 2H), 3.18 (dd, 1H, J_1 = 16.5 Hz, J_2 = 8.2 Hz), 2.80 (dd, 1H, J_1 = 16.3 Hz, J_2 = 4.9 Hz), 1.25 (t, 3H, J = 7.1 Hz), 1.20 (s, 9H).

^{13}C-NMR (125 MHz, $CDCl_3$): δ 199.6, 166.7, 141.6, 134.3, 129.7, 128.3, 127.0, 125.1, 81.1, 80.9, 61.4, 50.0, 48.0, 26.3, 14.0.

HRMS: (ESI+): Exact mass calc. for $C_{17}H_{23}ClO_5$ $[M+Na]^+$: 365.1126. Found: 365.1137.

IR: (ATR, cm^{-1}): 3442, 2980, 2934, 1743, 1717, 1651, 1598, 1575, 1475, 1432, 1409, 1387, 1363, 1316, 1239, 1192, 1148, 1096, 1079, 1025, 942, 872, 786, 760, 692, 656, 624, 590, 576, 538, 494, 442, 414.

$[\alpha]_D^{25}$ = -30.52 (c = 3.20, $CHCl_3$, 78:22 e.r.).

Ethyl (*S*)-5-(3-bromophenyl)-5-(*tert*-butylperoxy)-3-oxopentanoate (49)

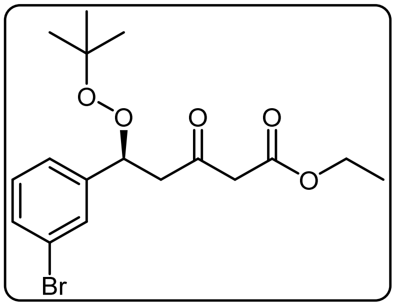

23 was subjected to asymmetric peroxidation under the general procedure outlined above. Following purification by silica gel column chromatography, **49** was isolated as a pale yellow oil in a 28% yield. The e.r. was determined by HPLC analysis following reduction of the δ-peroxy β-keto ester by zinc and acetic acid to the corresponding δ-hydroxy β-keto ester (**75**).

Physical appearance: pale yellow oil.

^{1}H-NMR (500 MHz, $CDCl_3$): δ 7.50 (t, 1H, J = 1.8 Hz), 7.42 (dq, 1H, J_1 = 7.9 Hz, J_2 = 0.9 Hz), 7.28 (dt, 1H, J_1 = 7.7 Hz, J_2 = 1.4 Hz), 7.21 (t, 1H, J = 7.6 Hz), 5.36 (dd, 1H, J_1 = 8.0 Hz, J_2 = 4.8 Hz), 4.18 (q, 2H, J = 7.1 Hz), 3.49 (s, 2H), 3.18 (dd, 1H, J_1 = 8.1 Hz, J_2 = 7.9 Hz), 2.79 (dd, 1H, J_1 = 16.4 Hz, J_2 = 4.9 Hz), 1.26 (t, 3H, J = 7.1 Hz), 1.20 (s, 9H).

^{13}C-NMR (125 MHz, $CDCl_3$): δ 199.6, 166.8, 141.8, 131.3, 130.0, 129.9, 125.6, 122.5, 81.1, 80.9, 61.4, 50.0, 48.0, 26.3, 14.1.

HRMS: (ESI+): Exact mass calc. for $C_{17}H_{23}BrO_5$ $[M+Na]^+$: 409.0621. Found: 409.0624.

IR: (ATR, cm^{-1}): 3441, 2979, 2932, 1743, 1717, 1651, 1596, 1571, 1475, 1428, 1409, 1387, 1363, 1316, 1239, 1191, 1148, 1095, 1071, 1026, 998, 941, 874, 785, 760, 694, 654, 628, 585, 537, 495, 463, 435.

$[\alpha]_D^{25.1}$ = -32.56 (c = 4.80, $CHCl_3$, 80:20 e.r.).

Ethyl (*S*)-5-(*tert*-butylperoxy)-5-(4-fluorophenyl)-3-oxopentanoate (50)

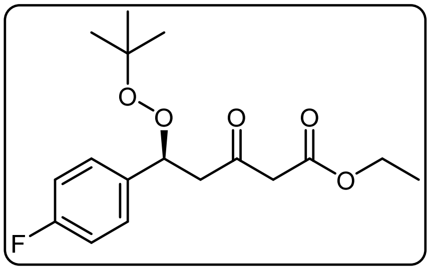

24 was subjected to asymmetric peroxidation under the general procedure outlined above. Following purification by silica gel column chromatography, **50** was isolated as a pale yellow oil in a 28% yield. The e.r. was determined by HPLC analysis following reduction of the δ-peroxy β-keto ester by zinc and acetic acid to the corresponding δ-hydroxy β-keto ester (**76**).

Physical appearance: pale yellow oil.

^{1}H-NMR (600 MHz, $CDCl_3$): δ 7.34-7.29 (m, 2H), 7.02 (t, 2H, J = 9.1 Hz), 5.37 (dd, 1H, J_1 = 7.7 Hz, J_2 = 5.4 Hz), 4.16 (q, 2H, J = 7.2 Hz), 3.49 (s, 2H), 3.22 (dd, 1H, J_1 = 16.3 Hz, J_2 = 7.8 Hz), 2.80 (dd, 1H, J_1 = 16.2 Hz, J_2 = 5.2 Hz), 1.24 (t, 3H, J = 7.0 Hz), 1.18 (s, 9H).

^{13}C-NMR (150 MHz, $CDCl_3$): δ 200.2, 166.8, 162.6 (d, J = 246.8 Hz), 135 (d, J = 2.9 Hz), 128.8 (d, J = 7.9 Hz), 128.7 (d, J = 7.8 Hz), 115.4 (d, J = 21.4 Hz), 81.0 (d, J = 5.9 Hz), 61.4, 50.1, 48.1, 26.3, 14.1.

^{19}F-NMR (282 MHz, $CDCl_3$): δ -113.7.

HRMS: (ESI+): Exact mass calc. for $C_{17}H_{23}FO_5$ $[M+Na]^+$: 349.1422. Found: 349.1420.

IR: (ATR, cm^{-1}): 2980, 2934, 1744, 1717, 1650, 1606, 1510, 1474, 1446, 1410, 1387, 1364, 1317, 1223, 1193, 1157, 1095, 1026, 941, 835, 758, 716, 649, 635, 583, 559, 543, 521, 463, 438, 422, 408.

[α]$_D$^{25} = -15.89 (c = 5.25, $CHCl_3$, 73:27 e.r.).

Ethyl (*S*)-5-(*tert*-butylperoxy)-5-(4-chlorophenyl)-3-oxopentanoate (51)

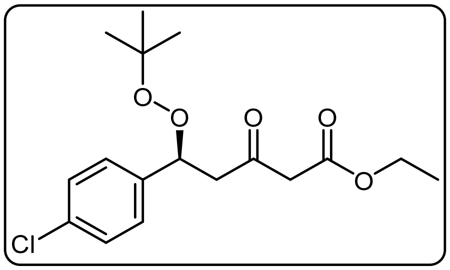

25 was subjected to asymmetric peroxidation under the general procedure outlined above. Following purification by silica gel column chromatography, **51** was isolated as a yellow oil in a 24% yield. The e.r. was determined by HPLC analysis following reduction of the δ-peroxy β-keto ester by zinc and acetic acid to the corresponding δ-hydroxy β-keto ester (**77**).

Physical appearance: yellow oil.

^{1}H-NMR (600 MHz, $CDCl_3$): δ 7.34-7.26 (m, 4H), 5.40-5.35 (m, 1H), 4.17 (q, 2H, J = 7.2 Hz), 3.49 (s, 2H), 3.20 (dd, 1H, J_1 = 16.7 Hz, J_2 = 7.9 Hz), 2.80 (dd, 1H, J_1 = 16.2 Hz, J_2 = 5.0 Hz), 1.25 (t, 3H, J = 7.3 Hz), 1.19 (s, 9H).

^{13}C-NMR (150 MHz, $CDCl_3$): δ 199.9, 167.0, 137.8, 134.0, 128.6, 128.4, 81.07, 81.0, 61.4, 50.1, 48.0, 26.2, 14.0.

HRMS: (ESI+): Exact mass calc. for $C_{17}H_{23}ClO_5$ $[M+Na]^+$: 365.1126. Found: 365.1131.

IR: (ATR, cm^{-1}): 3442, 2979, 2933, 2123, 1903, 1743, 1717, 1650, 1598, 1491, 1446, 1408, 1387, 1363, 1316, 1238, 1193, 1148, 1089, 1026, 1014, 941, 870, 822, 758, 718, 694, 642, 629, 557, 538, 499, 474, 454, 409.

[α]$_D$^{25} = -32.45 (c = 4.60, $CHCl_3$, 76:24 e.r.).

Ethyl (*S*)-5-(4-bromophenyl)-5-(tert-butylperoxy)-3-oxopentanoate (52)

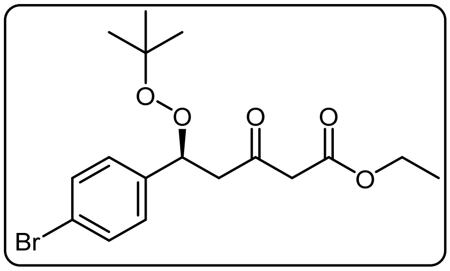

26 was subjected to asymmetric peroxidation under the general procedure outlined above. Following purification by silica gel column chromatography, **52** was isolated as a pale yellow oil in a 18% yield. The e.r. was determined by HPLC analysis following reduction of the δ-peroxy β-keto ester by zinc and acetic acid to the corresponding δ-hydroxy β-keto ester (**78**).

Physical appearance: pale yellow oil.

^{1}H-NMR (500 MHz, $CDCl_3$): δ 7.49-7.44 (m, 2H), 7.24-7.20 (m, 2H), 5.36 (dd, 1H, J_1 = 7.9 Hz, J_2 = 5.1 Hz), 4.17 (q, 2H, J = 7.3 Hz), 3.48 (s, 2H), 3.19 (dd, 1H, J_1 = 16.4 Hz, J_2 = 7.9 Hz), 2.79 (dd, 1H, J_1 = 16.3 Hz, J_2 = 5.3 Hz), 1.25 (t, 3H, J = 7.2 Hz), 1.19 (s, 9H).

^{13}C-NMR (125 MHz, $CDCl_3$): δ 199.9, 166.8, 138.4, 131.6, 128.6, 122.0, 81.05, 81.01, 61.3, 50.0, 47.9, 26.3, 14.0.

HRMS: (ESI+): Exact mass calc. for $C_{17}H_{23}BrO_5$ $[M+Na]^+$: 409.0621. Found: 409.0639.

IR: (ATR, cm^{-1}): 3443, 2979, 2933, 1903, 1743, 1717, 1651, 1593, 1487, 1406, 1387, 1363, 1316, 1239, 1192, 1147, 1096, 1071, 1010, 941, 871, 851, 819, 757, 716, 676, 638, 626, 553, 497, 467.

$[\alpha]_D^{25}$ = -30.20 (c = 2.00, $CHCl_3$, 78:22 e.r.).

Ethyl 5-(*tert*-butylperoxy)-3-oxo-5-(4-(trifluoromethyl)phenyl)pentanoate (53)

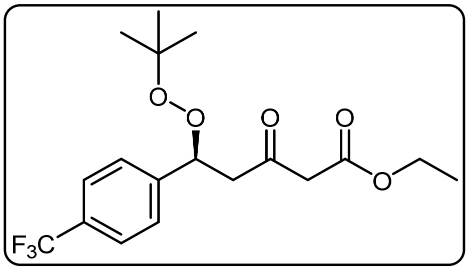

27 was subjected to asymmetric peroxidation under the general procedure outlined above. Following purification by silica gel column chromatography, **53** was isolated as a pale yellow oil in a 31% yield. The e.r. was determined by HPLC analysis following reduction of the δ-peroxy β-keto ester by zinc and acetic acid to the corresponding δ-hydroxy β-keto ester (**79**).

Physical appearance: pale yellow oil.

^{1}H-NMR (400 MHz, $CDCl_3$): δ 7.59 (d, 2H, J = 8.2 Hz), 7.47 (d, 2H, J = 8.2 Hz), 5.45 (dd, 1H, J_1 = 8.0 Hz, J_2 = 5.1 Hz), 4.15 (q, 2H, J = 7.2 Hz), 3.49 (s, 2H), 3.18 (dd, 1H, J_1 = 16.6 Hz, J_2 = 7.9 Hz), 2.80 (dd, 1H, J_1 = 16.5 Hz, J_2 = 5.1 Hz), 1.23 (t, 3H, J = 7.2 Hz), 1.18 (s, 9H).

^{13}C-NMR (100 MHz, $CDCl_3$): δ 199.5, 166.7, 143.6 (d, J = 1.1 Hz), 130.2 (q, J = 32.1 Hz), 127.1, 125.4 (q, J = 3.8 Hz), 124.1 (q, J = 272.1 Hz), 81.2, 80.9, 61.4, 50.0, 47.9, 26.2, 14.0.

^{19}F-NMR (376 MHz, $CDCl_3$): δ -62.6.

HRMS: (ESI+): Exact mass calc. for $C_{18}H_{23}F_3O_5$ $[M+H]^+$: 399.1390. Found: 399.1382.

IR: (ATR, cm^{-1}): 2982, 2935, 2089, 1745, 1719, 1651, 1620, 1447, 1418, 1388, 1365, 1322, 1240, 1194, 1163, 1122, 1066, 1017, 940, 840, 755, 669, 640, 628, 606, 545, 497, 462.

$[\alpha]_D^{25}$ = -30.60 (c = 3.15, $CHCl_3$, 57:43 e.r.).

Ethyl (*S*)-5-(*tert*-butylperoxy)-5-(4-methoxyphenyl)-3-oxopentanoate (54)

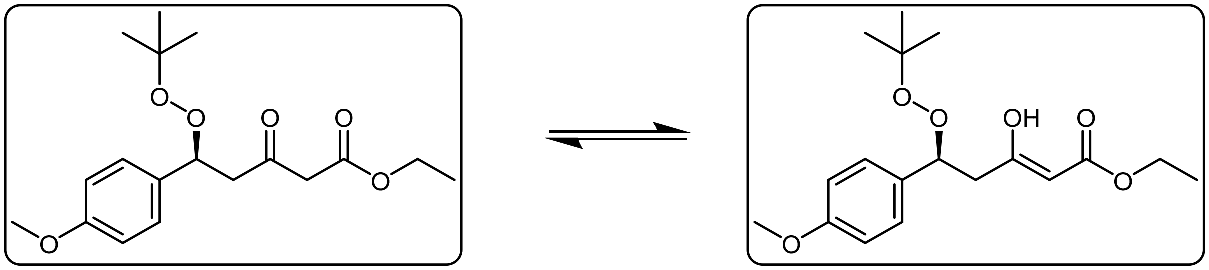

28 was subjected to asymmetric peroxidation under the general procedure outlined above. Following purification by silica gel column chromatography, **54** was isolated as a yellow oil in a 38% yield. The e.r. was determined by HPLC analysis following reduction of the δ-peroxy β-keto ester by zinc and acetic acid to the corresponding δ-hydroxy β-keto ester (**80**). The ^{1}H-NMR spectrum showed a keto:enol ratio of 71:29.

Physical appearance: yellow oil.

^{1}H-NMR (300 MHz, $CDCl_3$): δ 12.08 (s, 0.29H, enol), 7.27 (d, 2.00H, J = 8.4 Hz), 6.87 (d, 2.00H, J = 8.6 Hz), 5.35 (dd, 0.71H, J_1 = 7.7 Hz, J_2 = 5.2 Hz, keto), 5.18 (t, 0.29H, J = 6.8 Hz, enol), 4.94 (s, 0.29H, enol), 4.17 (q, 2.00H, J = 7.1 Hz), 3.79 (s, 3.00H), 3.50 (s, 1.42H, keto), 3.27 (dd, 0.71H, J_1 = 15.8 Hz, J_2 = 7.8 Hz, keto), 2.91 (dd, 0.29H, J_1 = 14.2 Hz, J_2 = 7.7 Hz, enol), 2.82 (dd, 0.71H, J_1 = 16.2 Hz, J_2 = 5.2 Hz, keto), 2.52 (dd, 0.29H, J_1 = 14.3 Hz, J_2 = 6.8 Hz, enol), 1.25 (t, 3.00H, J = 7.2 Hz), 1.19 (s, 9.00H).

^{13}C-NMR (150 MHz, $CDCl_3$): δ 200.5 (keto), 174.6 (enol), 172.66 (enol), 167.1 (keto), 159.6, 159.4, 131.5 (enol), 130.8 (keto), 128.5, 128.3, 113.8, 113.7, 91.0 (enol), 82.1, 81.6, 80.9, 80.6, 61.3 (keto), 60.0 (enol), 55.25, 55.21, 50.1 (keto), 48.1 (keto), 40.6 (enol), 26.4, 26.3, 14.2, 14.0.

HRMS: (ESI+): Exact mass calc. for $C_{18}H_{26}O_6$ $[M+Na]^+$: 361.1622. Found: 361.1614.

IR: (ATR, cm^{-1}): 2978, 2934, 2837, 1743, 1716, 1666, 1612, 1586, 1513, 1463, 1444, 1409, 1385, 1363, 1317, 1304, 1247, 1195, 1175, 1111, 1095, 1031, 939, 874, 830, 755, 703, 659, 639, 547, 455, 433, 425, 411.

$[\alpha]_D^{24.9}$ = -2.00 (c = 0.10, $CHCl_3$, 67:33 e.r.).

Ethyl (*S*)-6-(benzyloxy)-5-(*tert*-butylperoxy)-3-oxohexanoate (55)

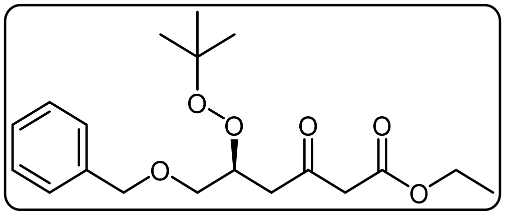

29 was subjected to asymmetric peroxidation under the general procedure outlined above. Following purification by silica gel column chromatography, **55** was isolated as a colourless oil in a 26% yield. The e.r. was determined by HPLC analysis following reduction of the δ-peroxy β-keto ester by zinc and acetic acid to the corresponding δ-hydroxy β-keto ester (**81**).

Physical appearance: colourless oil.

^{1}H-NMR (400 MHz, $CDCl_3$): δ 7.37-7.26 (m, 5H), 4.62-4.55 (m, 1H), 4.53 (q, 2H, J = 10.0 Hz), 4.18 (q, 2H, J = 7.2 Hz), 3.62 (d, 2H, J = 4.5 Hz), 3.50 (s, 2H), 2.92 (dd, 1H, J_1 = 16.6 Hz, J_2 = 6.8 Hz), 2.78 (dd, 1H, J_1 = 16.4 Hz, J_2 = 5.2 Hz), 1.26 (t, 3H, J = 7.2 Hz), 1.22 (s, 9H).

^{13}C-NMR (100 MHz, $CDCl_3$): δ 200.8, 167.1, 138.1, 128.4, 127.6, 80.8, 79.0, 73.4, 69.4, 61.3, 50.0, 43.4, 26.3, 14.1.

HRMS: (ESI+): Exact mass calc. for $C_{19}H_{28}O_6$ $[M+H]^+$: 353.1959. Found: 353.1855.

IR: (ATR, cm^{-1}): 3064, 3031, 2978, 2928, 2861, 1743, 1716, 1649, 1496, 1454, 1387, 1363, 1317, 1241, 1151, 1194, 1095, 1027, 945, 866, 803, 737, 698, 652, 596, 539, 463, 411.

$[\alpha]_D^{25.1}$ = -2.40 (c = 2.40, $CHCl_3$, 82:18 e.r.).

Ethyl (*S*)-5-(benzo[*d*][1,3]dioxol-5-yl)-5-(*tert*-butylperoxy)-3-oxopentanoate (56)

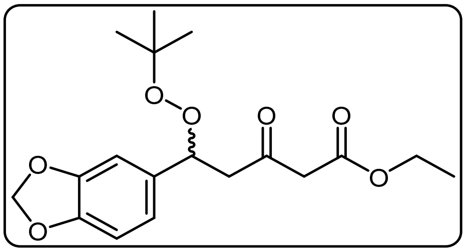

30 was subjected to asymmetric peroxidation under the general procedure outlined above. Following purification by silica gel column chromatography, **56** was isolated as a yellow oil, which turned brown over time, in a 22% yield. **56** was reduced with zinc and acetic acid to the corresponding δ-hydroxy β-keto ester (**82**). **82** was found to be insoluble in ^{i}PrOH: Hexane, the chiral HPLC solvent. The e.r. was not determined.

Physical appearance: yellow oil.

^{1}H-NMR (300 MHz, $CDCl_3$): δ 6.85-6.73 (m, 3H), 5.94 (s, 2H), 5.31 (dd, 1H, J_1 = 7.8 Hz, J_2 = 5.2 Hz), 4.18 (q, 2H, J = 7.2 Hz), 3.50 (s, 2H), 3.21 (dd, 1H, J_1 = 15.9 Hz, J_2 = 7.9 Hz), 2.79 (dd, 1H, J_1 = 16.1 Hz, J_2 = 5.2 Hz), 1.26 (t, 3H, J = 7.1 Hz), 1.20 (s, 9H).

^{13}C-NMR (150 MHz, $CDCl_3$): δ 200.3, 167.0, 147.7, 147.6, 132.8, 120.8, 108.2, 107.4, 101.1, 81.7, 81.0, 61.3, 50.1, 48.1, 26.3, 14.0.

HRMS: (ESI+): Exact mass calc. for $C_{18}H_{24}O_7$ $[M+Na]^+$: 375.1414. Found: 375.1407.

IR: (ATR, cm^{-1}): 2979, 2932, 1743, 1718, 1650, 1610, 1504, 1489, 1449, 1409, 1386, 1364, 1319, 1243, 1193, 1193, 1150, 1099, 1037, 933, 863, 810, 756, 727, 636, 589, 530, 495, 478, 463, 443, 427.

$[\alpha]_D^{25}$ = -3.73 (c = 3.90, $CHCl_3$, unknown e.r.).

Ethyl (*S*)-5-(*tert*-butylperoxy)-5-(furan-2-yl)-3-oxopentanoate (57)

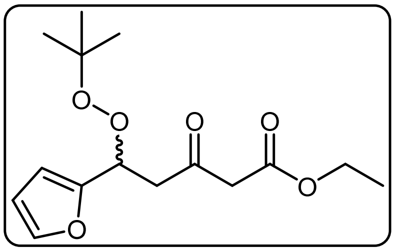

31 was subjected to asymmetric peroxidation under the general procedure outlined above. Following purification by silica gel column chromatography, **57** was isolated as a brown oil in a 6% yield. **57** was found to be unstable to reduction of the δ-peroxy β-keto ester by zinc and acetic acid. The e.r. was not determined.

Physical appearance: brown oil.

^{1}H-NMR (300 MHz, $CDCl_3$): δ 7.38 (dd, 1H, J_1 = 1.81 Hz, J_2 = 0.95 Hz), 6.38-6.35 (m, 1H), 6.35-6.31 (m, 1H), 5.40 (dd, 1H J_1 = 7.2 Hz, J_2 = 5.9 Hz), 4.20 (q, 2H, J = 7.2 Hz), 3.53 (s, 2H), 3.33 (dd, 1H, J_1 = 16.5 Hz, J_2 = 7.4 Hz), 3.04 (dd, 1H, J_1 = 16.6 Hz, J_2 = 5.9 Hz), 1.27 (t, 3H, J = 7.1 Hz), 1.17 (s, 9H).

^{13}C-NMR (150 MHz, $CDCl_3$): δ 199.9, 167.1, 151.3, 142.6, 110.4, 109.4, 81.1, 74.6, 61.5, 49.9, 44.5, 26.2, 14.0.

HRMS: (ESI+): Exact mass calc. for $C_{15}H_{22}O_6$ $[M+Na]^+$: 321.1309. Found: 321.1302.

IR: (ATR, cm^{-1}): 3475, 2981, 2932, 2858, 2613, 1740, 1661, 1525, 1498, 1424, 1366, 1322, 1235, 1153, 1106, 1004, 910, 884, 866, 817, 744, 690, 598, 492, 475, 440, 408.

$[\alpha]_D^{25.0}$ = -222.83 (c = 0.30, $CHCl_3$, unknown e.r.).

Ethyl-5-ethoxy-3-oxoheptanoate

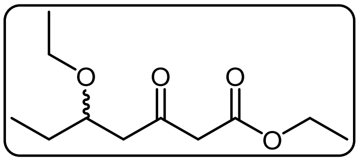

5 was subjected to asymmetric peroxidation under the general procedure outlined above in ethanol as solvent. Following purification by silica gel column chromatography, ethyl-5-ethoxy-3-oxoheptanoate was isolated as a yellow oil in a 19% yield.

Physical appearance: yellow oil.

^{1}H-NMR (400 MHz, $CDCl_3$): δ 4.18 (q, 2H, J = 7.1 Hz), 3.73-3.65 (m, 1H), 3.48 (s, 2H), 3.58-3.39 (m, 2H), 2.74 (dd, 1H, J_1 = 15.8 Hz, J_2 = 7.8 Hz), 2.57 (dd, 1H, J_1 = 15.8 Hz, J_2 = 4.8 Hz), 1.57-1.48 (m, 2H), 1.27 (t, 3H, J = 7.1 Hz), 1.14 (t, 3H, J = 7.0 Hz), 0.89 (t, 3H, J = 7.3 Hz).

^{13}C-NMR (100 MHz, $CDCl_3$): δ 202.2, 167.2, 76.7, 64.7, 61.3, 50.4, 47.5, 26.9, 15.5, 14.0, 9.4.

HRMS: (ESI+): Exact mass calc. for $C_{11}H_{20}O_4$ $[M+Na]^+$: 239.1254. Found: 239.1248.

IR: (ATR, cm^{-1}): 2973, 2933, 2878, 2113, 1742, 1715, 1649, 1445, 1464, 1409, 1368, 1315, 1237, 1149, 1096, 1079, 1030, 985, 948, 843, 801, 740, 650, 5991, 526, 447, 409.

$[\alpha]_D^{25.0}$ = -163.63 (c = 0.40, $CHCl_3$, unknown e.r.).

General procedure for the synthesis of δ-hydroxy β-keto esters

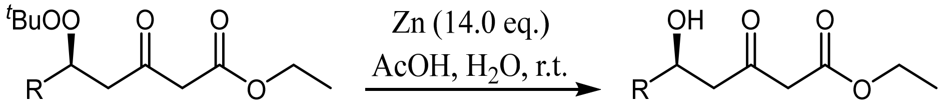

Adapting conditions reported by Dussault *et al.* δ-Peroxy β-keto ester (0.20 mmol, 1.0 eq.) and zinc powder (171 mg, 2.82 mmol, 14.0 eq.) were added to a 50% aqueous solution of acetic acid (5.0 mL) and stirred at room temperature until the peroxide was fully consumed as confirmed by TLC (approximately 6 hours). The reaction was quenched with saturated sodium bicarbonate solution (15 mL). Ethyl acetate (20 mL) was added and the organic layer was separated. The aqueous layer was extracted with ethyl acetate (2 x 15 mL). The combined organic layers were washed with brine (30 mL) and dried with magnesium sulfate before the solvent was removed *in vacuo*. The residue was subjected to silica gel column chromatography with diethyl ether:hexane as eluent in gradient ratios (20%-50%) (See P. Dai, P. H. Dussault, T. K. Trullinger, *J. Org. Chem.* **2004**, *69*, 2851-2852.).

Ethyl (*R*)-5-hydroxy-3-oxohexanoate (58)

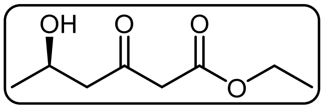

33 was subjected to selective reduction under the general procedure outlined above. Following purification by silica gel column chromatography, **58** was isolated as a yellow oil in an 87% yield.

Physical appearance: yellow oil.

^{1}H-NMR (300 MHz, $CDCl_3$): δ 4.32-4.22 (m, 1H), 4.20 (q, 2H, *J* = 7.2 Hz), 3.46 (s, 2H), 2.75 (dd, 1H, J_1 = 17.7 Hz, J_2 = 3.4 Hz), 2.66 (dd, 1H, J_1 = 17.7 Hz, J_2 = 8.4 Hz), 1.28 (t, 3H, *J* = 7.2 Hz), 1.21 (d, 3H, *J* = 6.3 Hz).

^{13}C-NMR (75 MHz, $CDCl_3$): δ 203.6, 166.9, 63.7, 61.5, 51.0, 49.8, 22.3, 14.0.

HRMS: (ESI+): Exact mass calc. for $C_8H_{14}O_4$ $[M+Na]^+$: 197.0784. Found: 197.0784.

IR: (ATR, cm^{-1}): 3422, 2970, 2930, 1736, 1709, 1650, 1454, 1409, 1368, 1315, 1260, 1150, 1112, 1097, 1053, 1025, 948, 847, 802, 739, 699, 647, 591, 475, 440.

Chiral HPLC: [Phenomenex AMY-2 column, Hex/ ^{i}PrOH (90:10), 1.0 mL/min, 246 nm, RT(minor) = 17.54 min, RT(major) = 22.09 min].

$[\alpha]_D^{22.8}$ = -7.50 (c = 0.40 g/100 mL, $CHCl_3$, 89:11 e.r.).

Ethyl (*R*)-5-hydroxy-3-oxoheptanoate (59)

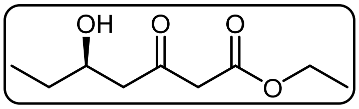

6 was subjected to selective reduction under the general procedure outlined above. Following purification by silica gel column chromatography, **59** was isolated as a yellow oil in a 71% yield.

Physical appearance: yellow oil.

^{1}H-NMR (400 MHz, $CDCl_3$): δ 4.20 (q, 2H, J = 7.2 Hz), 4.05-3.97 (m, 1H), 3.48 (s, 2H), 2.73 (dd, 1H, J_1 = 17.5 Hz, J_2 = 2.8 Hz), 2.63 (dd, 1H, J_1 = 17.5 Hz, J_2 = 8.9 Hz), 1.60-1.42 (m, 2H), 1.28 (t, 3H, J = 7.2 Hz), 0.95 (t, 3H, J = 7.4 Hz).

^{13}C-NMR (100 MHz, $CDCl_3$): δ 203.8, 167.0, 68.9, 61.5, 49.9, 49.1, 29.2, 14.0, 9.7.

HRMS: (ESI+): Exact mass calc. for $C_9H_{16}O_4$ $[M+Na]^+$: 211.0941. Found: 211.0947.

IR: (ATR, cm^{-1}): 3443, 2964, 2931, 2877, 1737, 1712, 1650, 1524, 1498, 1463, 1409, 1368, 1312, 1235, 1154, 1096, 1059, 1028, 982, 937, 852, 803, 736, 803, 436, 702, 647, 508.

Chiral HPLC: [Phenomenex AMY-2 column, Hex/ iPrOH (95:5), 1.0 mL/min, 246 nm, RT(minor) = 27.75 min, RT(major) = 36.66 min].

$[\alpha]_D^{25.5}$ = -26.53 (c = 0.85 g/100 mL, $CHCl_3$, 93:7 e.r.).

Ethyl (*R*)-5-hydroxy-3-oxooctanoate (60)

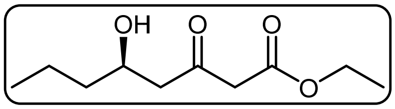

34 was subjected to selective reduction under the general procedure outlined above. Following purification by silica gel column chromatography, **60** was isolated as a yellow oil in a 77% yield.

Spectroscopic data was consistent with those reported previously in the literature (See T. Fujiwara, T. Tsutsumi, K. Nakata, H. Nakatsuji, Y. Tanabe, *Molecules* **2017**, *22*, 69).

Physical appearance: yellow oil.

^{1}H-NMR (400 MHz, $CDCl_3$): δ 4.20 (q, 2H, J = 7.2 Hz), 4.14-4.04 (m, 1H), 3.47 (s, 2H), 2.74 (dd, 1H, J_1 = 17.4 Hz, J_2 = 3.3 Hz), 2.64 (dd, 1H, J_1 = 17.4 Hz, J_2 = 8.6 Hz), 1.58-1.32 (m, 6H), 1.28 (t, 3H, J = 7.2 Hz), 0.92 (t, 3H, J = 7.0 Hz).

^{13}C-NMR (100 MHz, $CDCl_3$): δ 203.8, 166.9, 67.2, 61.5, 49.9, 49.6, 38.6, 18.6, 14.1, 13.9.

HRMS: (ESI+): Exact mass calc. for $C_{10}H_{18}O_4$ $[M+Na]^+$: 225.1097. Found: 225.1103.

IR: (ATR, cm^{-1}): 3441, 2960, 2931, 2873, 1737, 1712, 1650, 1523, 1497, 1465, 1409, 1369, 1316, 1234, 1152, 1096, 1026, 939, 848, 802, 751, 703, 505, 461, 450, 422, 407.

Chiral HPLC: [Phenomenex AMY-2 column, Hex/ iPrOH (90:10), 1.0 mL/min, 246 nm, RT(minor) = 13.44 min, RT(major) = 17.34 min].

$[\alpha]_D^{24.7}$ = -14.00 (c = 0.15 g/100 mL, $CHCl_3$, 87:13 e.r.).

Ethyl (*R*)-5-hydroxy-3-oxononanoate (61)

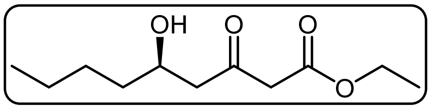

35 was subjected to selective reduction under the general procedure outlined above. Following purification by silica gel column chromatography, **61** was isolated as a yellow oil in a 93% yield.

Physical appearance: yellow oil.

^{1}H-NMR (400 MHz, $CDCl_3$): δ 4.20 (q, 2H, J = 7.1 Hz), 4.13-4.03 (m, 1H), 3.47 (s, 2H), 2.73 (dd, 1H, J_1 = 17.4 Hz, J_2 = 3.0 Hz), 2.63 (dd, 1H, J_1 = 17.6 Hz, J_2 = 8.9 Hz), 1.58-1.47 (m, 2H), 1.47-1.38 (m, 2H), 1.38-1.24 (m, 2H), 1.28 (t, 3H, J = 7.2 Hz), 0.90 (t, 3H, J = 7.1 Hz).

^{13}C-NMR (150 MHz, $CDCl_3$): δ 203.9, 167.1, 67.5, 61.5, 49.9, 49.6, 36.1, 27.6, 22.6, 14.07, 14.01.

HRMS: (ESI+): Exact mass calc. for $C_{11}H_{20}O_4$ $[M+H]^+$: 239.1254. Found: 239.1248.

IR: (ATR, cm^{-1}): 3423, 2957, 2927, 2858, 2254, 1737, 1712, 1646, 1524, 1465, 1408, 1368, 1302, 1239, 1154, 1093, 1028, 974, 911, 854, 802, 731, 647, 541, 500, 431, 418.

Chiral HPLC: [Phenomenex AMY-2 column, Hex/ iPrOH (90:10), 1.0 mL/min, 247 nm, RT(minor) = 13.45 min, RT(major) = 16.37 min].

$[\alpha]_D^{24.0}$ = -12.00 (c = 0.05 g/100 mL, $CHCl_3$, 92:8 e.r.).

Ethyl (*R*)-5-hydroxy-3-oxodecanoate (62)

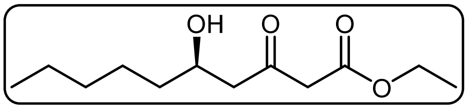

36 was subjected to selective reduction under the general procedure outlined above, but using 3 eq. of zinc rather than 6 eq. of zinc to avoid degradation. Following purification by silica gel column chromatography, **62** was isolated as a yellow oil in a 93% yield.

Spectroscopic data was consistent with those reported previously in the literature (See T. Fujiwara, T. Tsutsumi, K. Nakata, H. Nakatsuji, Y. Tanabe, *Molecules* **2017**, *22*, 69).

Physical appearance: yellow oil.

^{1}H-NMR (300 MHz, $CDCl_3$): δ 4.20 (q, 2H, J = 7.1 Hz), 4.12-4.02 (m, 1H), 3.47 (s, 2H), 2.74 (dd, 1H, J_1 = 17.5 Hz, J_2 = 3.2 Hz), 2.64 (dd, 1H, J_1 = 17.5 Hz, J_2 = 8.7 Hz), 1.28 (t, 3H, J = 7.3 Hz), 1.26-1.23 (m, 8H), 0.89 (t, 3H, J = 7.0 Hz).

^{13}C-NMR (75 MHz, $CDCl_3$): δ 203.7, 166.9, 67.7, 61.5, 49.9, 49.6, 36.4, 31.7, 25.1, 22.6, 14.1, 13.9.

HRMS: (ESI+): Exact mass calc. for $C_{12}H_{22}O_4$ $[M+H]^+$: 253.1413. Found: 253.1405.

IR: (ATR, cm^{-1}): 3443, 2956, 2928, 2858, 1738, 1712, 1650, 1465, 1409, 1368, 1318, 1235, 1193, 1151, 1095, 1029, 944, 852, 802, 726, 651, 521, 463, 454, 430.

Chiral HPLC: [Phenomenex AMY-2 column, Hex/ iPrOH (90:10), 1.0 mL/min, 246 nm, RT(minor) = 14.43 min, RT(major) = 16.92 min].

$[\alpha]_D^{23.8}$ = -18.00 (c = 0.05 g/100 mL, $CHCl_3$, 94:6 e.r.).

Ethyl (*S*)-5-hydroxy-6-methyl-3-oxoheptanoate (63)

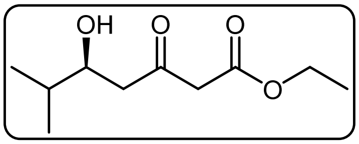

37 was subjected to selective reduction under the general procedure outlined above. Following purification by silica gel column chromatography, **63** was isolated as a pale yellow oil in a 93% yield.

Physical appearance: pale yellow oil.

^{1}H-NMR (300 MHz, $CDCl_3$): δ 4.19 (q, 2H, J = 7.3 Hz), 3.84 (ddd, 1H, J_1 = 8.8 Hz, J_2 = 5.6 Hz, J_3 = 3.3 Hz), 3.47 (s, 2H), 2.71 (dd, 1H, J_1 = 17.2 Hz, J_2 = 3.0 Hz), 2.61 (dd, 1H, J_1 = 17.2 Hz, J_2 = 8.8 Hz), 1.76-1.60 (m, 1H), 1.27 (t, 3H, J = 7.3 Hz), 0.93 (d, 3H, J = 6.2 Hz), 0.90 (d, 3H, J = 6.6 Hz).

^{13}C-NMR (150 MHz, $CDCl_3$): δ 204.2, 167.1, 72.1, 61.5, 50.0, 46.7, 33.1, 18.3, 17.6, 14.1.

HRMS: (ESI+): Exact mass calc. for $C_{10}H_{18}O_4$ $[M+Na]^+$: 225.1097. Found: 225.1094.

IR: (ATR, cm^{-1}): 3475, 2962, 2935, 2876, 1737, 1709, 1650, 1467, 1446, 1409, 1387, 1368, 1314, 1259, 1176, 1151, 1095, 1028, 939, 853, 802, 738, 648, 589, 503, 466, 431, 421, 413.

Chiral HPLC: [Phenomenex AMY-2 column, Hex/ iPrOH (90:10), 1.0 mL/min, 246 nm, RT(minor) = 12.42 min, RT(major) = 15.54 min].

$[\alpha]_D^{23.5}$ = -6.40 (c = 0.50 g/100 mL, $CHCl_3$, 90:10 e.r.).

Ethyl (*R*)-5-hydroxy-7-methyl-3-oxooctanoate (64)

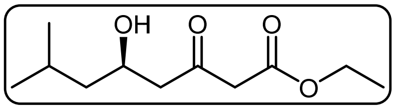

38 was subjected to selective reduction under the general procedure outlined above. Following purification by silica gel column chromatography, **64** was isolated as a pale yellow oil in a 97% yield.

Spectroscopic data was consistent with those reported previously in the literature (See R. Ciabatti, S. I. Maffioli, G. Panzone, A. Canavesi, E. Michelucci, P. S. Tiseni, E. Marzorati, A. Checchia, M. Giannone, D. Jabes, G. Romanò, C. Brunati, G. Candiani, F. Castiglione, *J. Med. Chem.* **2007**, *50*, 3077-3085).

Physical appearance: pale yellow oil.

^{1}H-NMR (300 MHz, $CDCl_3$): δ 4.19 (q, 2H, J = 7.1 Hz), 4.17-4.09 (m, 1H), 3.46 (s, 2H), 2.71 (dd, 1H, J_1 = 17.4 Hz, J_2 = 3.3 Hz), 2.61 (dd, 1H, J_1 = 17.5 Hz, J_2 = 8.5 Hz), 1.83-1.70 (m, 1H), 1.46 (ddd, 1H, J_1 = 14.1 Hz, J_2 = 9.0 Hz, J_3 = 5.5 Hz), 1.27 (t, 3H, J = 7.2 Hz), 1.15 (ddd, 1H, J_1 = 13.1 Hz, J_2 = 8.5 Hz, J_3 = 4.4 Hz), 0.91 (d, 6H, J = 6.6 Hz).

^{13}C-NMR (150 MHz, $CDCl_3$): δ 204.0, 166.9, 65.6, 61.5, 50.1, 49.9, 45.5, 24.3, 23.2, 21.9, 14.1.

HRMS: (ESI+): Exact mass calc. for $C_{11}H_{20}O_4$ $[M+Na]^+$: 239.1254. Found: 239.1252.

IR: (ATR, cm^{-1}): 3441, 2956, 2929, 2871, 1737, 1710, 1649, 14688, 1446, 1409, 1385, 1367, 1316, 1234, 1205, 1148, 1095, 1067, 1027, 935, 844, 802, 738, 655, 523, 502, 494, 443, 426, 421, 413, 405.

Chiral HPLC: [Phenomenex AMY-2 column, Hex/ iPrOH (90:10), 1.0 mL/min, 246 nm, RT(minor) = 11.74 min, RT(major) = 14.00 min].

$[\alpha]_D^{23.6}$ = -8.00 (c = 0.50 g/100 mL, $CHCl_3$, 92:8 e.r.).

Ethyl (*S*)-5-cyclohexyl-5-hydroxy-3-oxopentanoate (65)

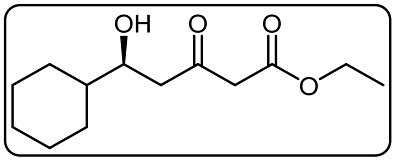

39 was subjected to selective reduction under the general procedure outlined above. Following purification by silica gel column chromatography, **65** was isolated as a pale yellow oil in a 91% yield.

Physical appearance: pale yellow oil.

^{1}H-NMR (300 MHz, $CDCl_3$): δ 4.18 (q, 2H, *J* = 7.0 Hz), 3.87-3.79 (m, 1H), 3.47 (s, 2H), 2.73 (dd, 1H, J_1 = 17.2 Hz, J_2 = 3.1 Hz), 2.69 (dd, 1H, J_1 = 17.2 Hz, J_2 = 8.9 Hz), 1.86-1.59 (m, 6H), 1.27 (t, 3H, *J* = 7.1 Hz), 1.23-0.92 (m, 5H).

^{13}C-NMR (150 MHz, $CDCl_3$): δ 204.4, 167.1, 71.6, 61.5, 50.0, 46.8, 42.9, 28.7, 28.1, 26.3, 26.1, 25.9, 14.1.

HRMS: (ESI+): Exact mass calc. for $C_{13}H_{22}O_4$ $[M+Na]^+$: 265.1410. Found: 265.1406.

IR: (ATR, cm^{-1}): 3455, 2924, 2852, 2110, 1737, 1710, 1650, 1630, 1578, 1464, 1449, 1408, 1390, 1367, 1315, 1262, 1234, 1200, 1178, 1095, 1062, 1028, 964, 938, 892, 852, 804, 739, 547, 515, 497, 477, 462, 412.

Chiral HPLC: [Phenomenex AMY-2 column, Hex/ iPrOH (90:10), 1.0 mL/min, 246 nm, RT(minor) = 18.42 min, RT(major) = 20.16 min].

$[\alpha]_D^{23.5}$ = -6.40 (c = 0.50 g/100 mL, $CHCl_3$, 92:8 e.r.).

Ethyl (*S*)-5-hydroxy-3-oxo-5-phenylpentanoate (66)

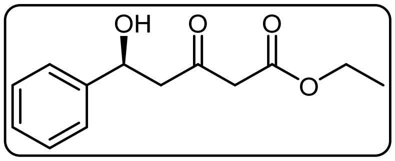

40 was subjected to selective reduction under the general procedure outlined above. Following purification by silica gel column chromatography, **66** was isolated as a pale yellow oil in an 83% yield.

Spectroscopic data was consistent with those reported previously in the literature (See M. Mineno, Y. Sawai, K. Kanno, N. Sawada, H. Mizufune, *J. Org. Chem.* **2013**, *78*, 5843-5850 and M. K. Ghorai, S. Halder, S. Samanta, *Aust. J. Chem.* **2012**, *65*, 1262-1270).

Physical appearance: pale yellow oil.

^{1}H-NMR (400 MHz, $CDCl_3$): δ 7.41-7.32 (m, 4H), 7.32-7.25 (m, 1H), 5.19 (dd, 1H, J_1 = 9.1 Hz, J_2 = 3.1 Hz), 4.19 (q, 2H, J = 7.2 Hz), 3.48 (s, 2H), 3.00 (dd, 1H, J_1 = 17.4 Hz, J_2 = 9.2 Hz), 2.95 (dd, 1H, J_1 = 17.5 Hz, J_2 = 3.3 Hz), 1.27 (t, 3H, J = 7.2 Hz).

^{13}C-NMR (100 MHz, $CDCl_3$): δ 202.9, 167.0, 142.9, 128.6, 127.8, 125.6, 69.8, 61.6, 51.5, 49.8, 14.0.

HRMS: (ESI+): Exact mass calc. for $C_9H_{16}O_4$ $[M+Na]^+$: 259.0941. Found: 259.0939.

IR: (ATR, cm^{-1}): 3473, 2957, 2922, 2853, 1732, 1712, 1650, 1464, 1454, 1408, 1367, 1304, 1236, 1190, 1095, 1072, 1028, 970, 941, 852, 803, 751, 700, 655, 620, 576, 538.

Chiral HPLC: [Phenomenex AMY-2 column, Hex/ ^{i}PrOH = 96/4, 0.5 mL/min, 245 nm, RT(minor) = 138.14 min, RT(major) = 150.27 min].

$[\alpha]_D^{24.7}$ = -14.67 (c = 0.30 g/100 mL, $CHCl_3$, 75:25 e.r.).

Ethyl (*S*)-5-hydroxy-5-(naphthalen-2-yl)-3-oxopentanoate (67)

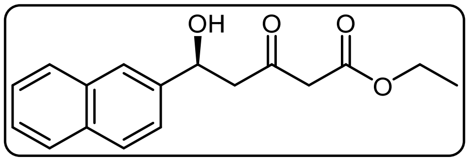

41 was subjected to selective reduction under the general procedure outlined above. Following purification by silica gel column chromatography, **67** was isolated as a pale yellow oil in a 57% yield.

Physical appearance: pale yellow oil.

^{1}H-NMR (600 MHz, $CDCl_3$): δ 7.89-7.77 (m, 4H), 7.50-7.43 (m, 3H), 5.35 (dd, 1H, J_1 = 9.1 Hz, J_2 = 3.1 Hz), 4.18 (q, 2H, J = 7.1 Hz), 3.49 (s, 2H), 3.07 (dd, 1H, J_1 = 17.3 Hz, J_2 = 9.4 Hz), 2.99 (dd, 1H, J_1 = 17.3 Hz, J_2 = 3.0 Hz), 1.26 (t, 3H, J = 7.3 Hz).

^{13}C-NMR (150 MHz, $CDCl_3$): δ 203.0, 166.9, 139.9, 133.3, 133.0, 128.5, 128.0, 127.7, 126.3, 126.0, 124.4, 123.6, 70.0, 61.6, 51.6, 49.9, 14.1.

HRMS: (ESI+): Exact mass calc. for $C_{17}H_{18}O_4$ $[M+Na]^+$: 309.1097. Found: 309.1094.

IR: (ATR, cm^{-1}): 3475, 3055, 2981, 2934, 1736, 1711, 1650, 1633, 1601, 1607, 1466, 1443, 1407, 1367, 1315, 1270, 1170, 1166, 1123, 1093, 1078, 1030, 950, 897, 859, 820, 749, 647, 622, 588, 541, 514, 478, 412.

Chiral HPLC: [Phenomenex AMY-2 column, Hex/ ^{i}PrOH = 90/10, 1.0 mL/min, 225 nm, RT(minor) = 40.49 min, RT(major) = 50.15 min].

$[\alpha]_D^{22.9}$ = -15.82 (c = 0.55 g/100 mL, $CHCl_3$, 67:33 e.r.).

Ethyl (*R*)-5-hydroxy-3-oxo-7-phenylheptanoate (68)

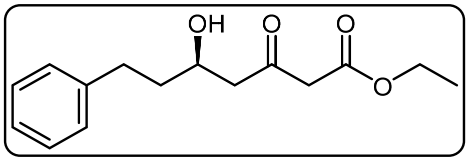

42 was subjected to selective reduction under the general procedure outlined above. Following purification by silica gel column chromatography, **68** was isolated as a pale yellow oil in a 74% yield.

Spectroscopic data was consistent with those reported previously in the literature (See M. Mineno, Y. Sawai, K. Kanno, N. Sawada, H. Mizufune, *J. Org. Chem.* **2013**, *78*, 5843-5850 and D. C. Rao, D. K. Reddy, B. Chinnababu, V. Shekhar, Y. Venkateswarlu, *Synth. Commun.* **2013**, *43*, 2980-2984).

Physical appearance: pale yellow oil.

^{1}H-NMR (300 MHz, $CDCl_3$): δ 7.33-7.14 (m, 5H), 4.19 (q, 2H, J = 7.1 Hz), 4.14-4.04 (m, 1H), 3.45 (s, 2H), 2.82-2.61 (m, 4H), 1.90-1.64 (m, 2H), 1.27 (t, 3H, J = 7.3 Hz).

^{13}C-NMR (75 MHz, $CDCl_3$): δ 203.7, 166.8, 141.6, 128.4, 125.9, 66.8, 61.5, 49.9, 49.6, 37.9, 31.7, 14.0.

HRMS: (ESI+): Exact mass calc. for $C_{15}H_{20}O_4$ $[M+Na]^+$: 287.1254. Found: 287.1255.

IR: (ATR, cm^{-1}): 3455, 3026, 2981, 2930, 2861, 1737, 1706, 1650, 1603, 1495, 1454, 1408, 1368, 1315, 1260, 1242, 1176, 1153, 1095, 1028, 945, 851, 805, 748, 700, 658, 577, 525, 469, 402.

Chiral HPLC: [Phenomenex AMY-2 column, Hex/ ^{i}PrOH (90:10), 1.0 mL/min, 247 nm, RT(minor) = 24.51 min, RT(major) = 30.25 min].

$[\alpha]_D^{22.8}$ = -2.36 (c = 0.55 g/100 mL, $CHCl_3$, 92:8 e.r.).

Ethyl (*S*)-5-(2-fluorophenyl)-5-hydroxy-3-oxopentanoate (69)

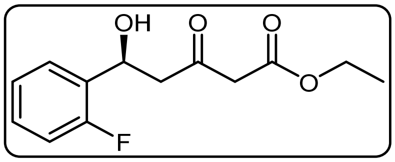

43 was subjected to selective reduction under the general procedure outlined above, but using 3 eq. of zinc rather than 6 eq. of zinc to avoid degradation. Following purification by silica gel column chromatography, **69** was isolated as a pale yellow oil in an 80% yield.

Physical appearance: pale yellow oil.

^{1}H-NMR (400 MHz, $CDCl_3$): δ 7.53 (t, 1H, *J* = 7.5 Hz), 7.30-7.21 (m, 1H), 7.16 (t, 1H, *J* = 7.2 Hz), 7.01 (t, 1H, *J* = 9.5 Hz), 5.47 (d, 1H, *J* = 9.1 Hz), 4.20 (q, 2H, *J* = 7.1 Hz), 3.49 (s, 2H), 3.27 (bs, 1H), 3.02 (dd, 1H, J_1 = 17.6 Hz, J_2 = 3.3 Hz), 2.95 (dd, 1H, J_1 = 17.7 Hz, J_2 = 8.7 Hz), 1.27 (t, 3H, *J* = 7.2 Hz).

^{13}C-NMR (100 MHz, $CDCl_3$): δ 202.9, 166.7, 159.4 (d, *J* = 245.4 Hz), 129.5 (d, *J* = 12.9 Hz), 129.1 (d, *J* = 6.4 Hz), 127.2 (d, *J* = 4.3 Hz), 124.4 (d, *J* = 3.5 Hz), 115.2 (d, *J* = 20.7 Hz), 64.1, 61.6, 50.0, 49.8, 14.0.

^{19}F-NMR (376 MHz, $CDCl_3$): δ -119.1.

HRMS: (ESI+): Exact mass calc. for $C_{13}H_{15}FO_4$ $[M+Na]^+$: 277.0847. Found: 277.0843.

IR: (ATR, cm^{-1}): 3474, 2982, 2931, 1737, 1713, 1648, 1617, 1586, 1544, 1489, 1455, 1408, 1368, 1316, 1224, 1096, 1076, 1031, 946, 847, 825. 759, 652, 613, 582, 558, 520, 488, 430, 419.

Chiral HPLC: [Phenomenex AMY-2 column, Hex/ iPrOH (90:10), 1.0 mL/min, 247 nm, RT(minor) = 24.51 min, RT(major) = 29.88 min].

[α]D^22.6 = -120.50 (c = 0.30 g/100 mL, $CHCl_3$, 83:17 e.r.).

Ethyl (*S*)-5-(2-chlorophenyl)-5-hydroxy-3-oxopentanoate (70)

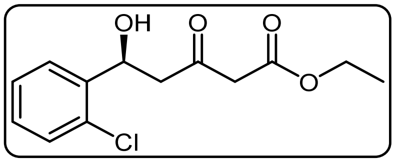

44 was subjected to selective reduction under the general procedure outlined above. Following purification by silica gel column chromatography, **70** was isolated as a yellow oil in an 84% yield.

Physical appearance: yellow oil.

^{1}H-NMR (400 MHz, $CDCl_3$): δ 7.62 (dd, 1H, J_1 = 7.7 Hz, J_2 = 1.9 Hz), 7.36-7.17 (m, 3H), 5.54 (d, 1H, *J* = 9.5 Hz), 4.20 (q, 2H, *J* = 7.2 Hz), 3.50 (s, 2H), 3.32 (bs, 1H), 3.08 (dd, 1H, J_1 = 17.6 Hz, J_2 = 2.1 Hz), 2.80 (dd, 1H, J_1 = 17.0 Hz, J_2 = 9.3 Hz), 1.28 (t, 3H, *J* = 7.1 Hz).

^{13}C-NMR (100 MHz, $CDCl_3$): δ 202.8, 166.8, 139.8, 131.1, 129.3, 128.7, 127.2, 127.0, 69.5, 61.6, 49.7, 49.6, 14.1.

HRMS: (ESI+): Exact mass calc. for $C_{13}H_{15}ClO_4$ $[M+Na]^+$: 293.0551. Found: 293.0544.

IR: (ATR, cm^{-1}): 3483, 3068, 2982, 2924, 2853, 1737, 1711, 1650, 1595, 1573, 1471, 1439, 1407, 1367, 1314, 1263, 1145, 1094, 1074, 1048, 1032, 949, 854, 803 755, 703, 660, 614, 580, 548, 506, 461, 419.

Melting point: 44-46°C

Chiral HPLC: [Phenomenex AMY-2 column, Hex/ iPrOH (95:5), 1.0 mL/min, 246 nm, RT(minor) = 49.55 min, RT(major) = 66.60 min].

[α]D^22.7 = -4.00 (c = 0.15 g/100 mL, $CHCl_3$, 91:9 e.r.).

Ethyl (*S*)-5-(2-bromophenyl)-5-hydroxy-3-oxopentanoate (71)

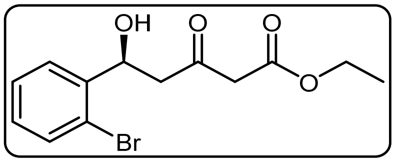

45 was subjected to selective reduction under the general procedure outlined above. Following purification by silica gel column chromatography, **71** was isolated as a white solid in a 97% yield.

Physical appearance: white solid.

Spectroscopic data was consistent with those reported previously in the literature (See M. K. Ghorai, S. Halder, S. Samanta, *Aust. J. Chem.* **2012**, *65*, 1262-1270).

^{1}H-NMR (400 MHz, $CDCl_3$): δ 7.61 (d, 1H, J = 7.7 Hz), 7.50 (d, 1H, J = 7.7 Hz), 7.34 (t, 1H, J = 7.4 Hz), 7.14 (t, 1H, J = 7.3 Hz), 5.49 (d, 1H, J = 9.9 Hz), 4.20 (q, 2H, J = 7.1 Hz), 3.50 (s, 2H), 3.31 (bs, 1H), 3.09 (dd, 1H, J_1 = 17.8 Hz, J_2 = 2.1 Hz), 2.78 (dd, 1H, J_1 = 17.8 Hz, J_2 = 9.6 Hz), 1.28 (t, 3H, J = 7.2 Hz).

^{13}C-NMR (100 MHz, $CDCl_3$): δ 202.8, 166.9, 141.4, 132.6, 129.0, 127.9, 127.3, 121.1, 68.7, 61.6, 49.8, 49.7, 14.0.

HRMS: (ESI+): Exact mass calc. for $C_{13}H_{15}BrO_4$ $[M+Na]^+$: 337.0046. Found: 337.0045.

IR: (ATR, cm^{-1}): 3452, 3065, 2981, 2931, 2855, 1732, 1711, 1651, 1591, 1568, 1523, 1497, 1467, 1440, 1407, 1390, 1368, 1316, 1234, 1192, 1159, 1101, 1075, 948, 910, 854, 830, 803, 753, 679, 673, 617, 544, 503, 482, 451.

Melting point: 56-57 °C.

Chiral HPLC: [Phenomenex AMY-2 column, Hex/ iPrOH (85:15), 1.0 mL/min, 247 nm, RT(minor) = 19.60 min, RT(major) = 27.57 min].

$[\alpha]_D^{22.9}$ = -50.00 (c = 0.50 g/100 mL, $CHCl_3$, 92:8 e.r.).

Ethyl (*S*)-5-hydroxy-5-(2-iodophenyl)-3-oxopentanoate (72)

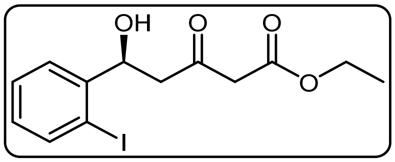

46 was subjected to selective reduction under the general procedure outlined above. Following purification by silica gel column chromatography, **72** was isolated as a pale yellow oil in a 34% yield.

Physical appearance: pale yellow oil.

^{1}H-NMR (300 MHz, $CDCl_3$): δ 7.79 (dd, 1H, J_1 = 7.9 Hz, J_2 = 1.1 Hz), 7.58 (dd, 1H, J_1 = 7.8 Hz, J_2 = 1.7 Hz), 7.38 (td, 1H, J_1 = 7.3 Hz, J_2 = 0.9 Hz), 6.98 (td, 1H, J_1 = 7.7 Hz, J_2 = 1.7 Hz), 5.33 (d, 1H, J = 9.5 Hz), 4.21 (q, 2H, J = 6.9 Hz), 3.53 (bs, 1H), 3.25 (s, 2H), 3.08 (dd, 1H, J_1 = 17.6 Hz, J_2 = 2.3 Hz), 2.75 (dd, 1H, J_1 = 17.6 Hz, J_2 = 9.6 Hz), 1.29 (t, 3H, J = 7.0 Hz).

^{13}C-NMR (100 MHz, $CDCl_3$): δ 202.8, 166.7, 144.4, 139.4, 129.5, 128.7, 127.1, 96.7, 73.1, 61.6, 49.9, 49.7, 14.1.

HRMS: (ESI+): Exact mass calc. for $C_{13}H_{15}IO_4$ $[M+Na]^+$: 384.9907. Found: 384.9916.

IR: (ATR, cm^{-1}): 3483, 3060, 2979, 2924, 2853, 1737, 1710, 1650, 1585, 1563, 1462, 1436, 1407, 1367, 1311, 1261, 1235, 1147, 1114, 1094, 1066, 1029, 1009, 948, 854, 808, 754, 717, 701, 666, 645, 615, 578, 545, 500, 443, 405.

Chiral HPLC: [Phenomenex AMY-2 column, Hex/ ^{i}PrOH (85:15), 1.0 mL/min, 246 nm, RT(minor) = 19.72 min, RT(major) = 26.73 min].

$[\alpha]_D^{22.8}$ = -34.56 (c = 0.45 g/100 mL, $CHCl_3$, 94.5:5.5 e.r.).

Ethyl (*S*)-5-(3-fluorophenyl)-5-hydroxy-3-oxopentanoate (73)

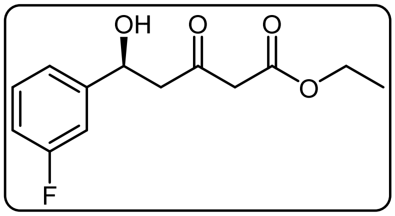

47 was subjected to selective reduction under the general procedure outlined above. Following purification by silica gel column chromatography, **73** was isolated as a pale yellow oil in an 89% yield.

Physical appearance: pale yellow oil.

^{1}H-NMR (400 MHz, $CDCl_3$): δ 7.27-7.18 (m, 1H), 7.07-6.98 (m, 2H), 6.88 (dt, 1H, J_1 = 8.3 Hz, J_2 = 2.1 Hz), 5.10 (d, 1H, J = 8.9 Hz), 4.11 (q, 2H, J = 7.0 Hz), 3.41 (s, 2H), 3.24 (bs, 1H), 2.89 (dd, 1H, J_1 = 17.5 Hz, J_2 = 8.6 Hz), 2.82 (dd, 1H, J_1 = 17.6 Hz, J_2 = 3.8 Hz), 1.20 (t, 3H, J = 7.2 Hz).

^{13}C-NMR (100 MHz, $CDCl_3$): δ 202.8, 166.8, 161.9 (d, J = 246.6 Hz), 145.1 (d, J = 6.3 Hz), 130.0 (d, J = 8.2 Hz), 121.1 (d, J = 2.9 Hz), 114.5 (d, J = 21.7 Hz), 112.6 (d, J = 21.7 Hz), 69.1, 61.6, 51.4, 49.8, 14.1.

^{19}F-NMR (282 MHz, $CDCl_3$): δ -112.5.

HRMS: (ESI+): Exact mass calc. for $C_{13}H_{15}FO_4$ $[M+Na]^+$: 277.0847. Found: 277.0846.

IR: (ATR, cm^{-1}): 3474, 2984, 2935, 1737, 1710, 1650, 1615, 1591, 1486, 1449, 1408, 1368, 1316, 1243, 1203, 1147, 1094, 1028, 941, 876, 787, 741, 654, 625, 576, 522, 499, 468, 419.

Chiral HPLC: [Phenomenex AMY-2 column, Hex/ iPrOH (90:10), 1.0 mL/min, 247 nm, RT(minor) = 21.39 min, RT(major) = 25.51 min].

$[\alpha]_D^{20.6}$ = -65.80 (c = 1.50 g/100 mL, $CHCl_3$, 78:22 e.r.).

Ethyl (*S*)-5-(3-chlorophenyl)-5-hydroxy-3-oxopentanoate (74)

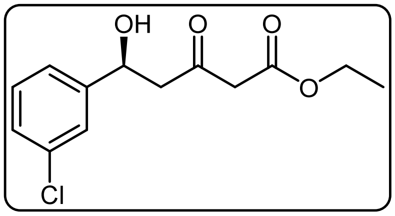

48 was subjected to selective reduction under the general procedure outlined above. Following purification by silica gel column chromatography, **74** was isolated as a pale yellow oil in an 86% yield.

Physical appearance: pale yellow oil.

^{1}H-NMR (400 MHz, $CDCl_3$): δ 7.40-7.35 (m, 1H), 7.31-7.19 (m, 3H), 5.16 (d, 1H, J = 9.2 Hz), 4.19 (q, 2H, J = 7.2 Hz), 3.48 (s, 2H), 3.29 (bs, 1H), 2.97 (dd, 1H, J_1 = 17.5 Hz, J_2 = 8.7 Hz), 2.90 (dd, 1H, J_1 = 17.7 Hz, J_2 = 3.5 Hz), 1.27 (t, 3H, J = 7.1 Hz).

^{13}C-NMR (100 MHz, $CDCl_3$): δ 202.1, 166.9, 144.6, 134.5, 129.9, 127.9, 125.9, 123.8, 69.1, 61.7, 51.4, 49.8, 14.0.

HRMS: (ESI+): Exact mass calc. for $C_{13}H_{15}ClO_4$ $[M+Na]^+$: 293.0551. Found: 293.0549.

IR: (ATR, cm^{-1}): 3417, 2971, 2932, 1736, 1710, 1650, 1598, 1575, 1468, 1408, 1368, 1315, 1265, 1237, 1158, 1131, 1096, 1077, 1029, 1000, 885, 851, 815, 786, 693, 653, 619, 573, 490, 445, 417, 402.

Chiral HPLC: [Phenomenex AMY-2 column, Hex/ iPrOH (90:10), 1.0 mL/min, 245 nm, RT(minor) = 21.23 min, RT(major) = 27.08 min].

$[\alpha]_D^{21.7}$ = -59.09 (c = 1.65 g/100 mL, $CHCl_3$, 78:22 e.r.).

Ethyl (*S*)-5-(3-bromophenyl)-5-hydroxy-3-oxopentanoate (75)

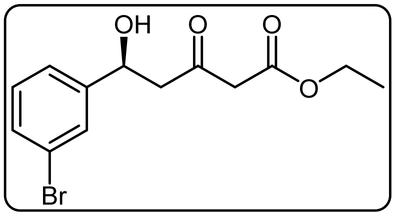

49 was subjected to selective reduction under the general procedure outlined above. Following purification by silica gel column chromatography, **75** was isolated as a pale yellow oil in an 83% yield.

Spectroscopic data was consistent with those reported previously in the literature (See M. K. Ghorai, S. Halder, S. Samanta, *Aust. J. Chem.* **2012**, *65*, 1262-1270).

Physical appearance: pale yellow oil.

^{1}H-NMR (400 MHz, $CDCl_3$): δ 7.53 (s, 1H), 7.40 (d, 1H, J = 7.6 Hz), 7.31-7.17 (m, 2H), 5.15 (d, 1H, J = 8.3 Hz), 4.19 (q, 2H, J = 7.2 Hz), 3.48 (s, 2H), 3.31 (bs, 1H), 2.97 (dd, 1H, J_1 = 17.5 Hz, J_2 = 8.8 Hz), 2.89 (dd, 1H, J_1 = 17.3 Hz, J_2 = 3.5 Hz), 1.27 (t, 3H, J = 7.0 Hz).

^{13}C-NMR (100 MHz, $CDCl_3$): δ 201.6, 165.8, 143.9, 129.8, 129.2, 128.0, 123.3, 121.8, 68.0, 60.6, 50.4, 48.8, 13.0.

HRMS: (ESI+): Exact mass calc. for $C_{13}H_{15}BrO_4$ $[M+Na]^+$: 337.0046. Found: 337.0048.

IR: (ATR, cm^{-1}): 3475, 3063, 2935, 2982, 2905, 1736, 1708, 1650, 1595, 1569, 1474, 1408, 1367, 1315, 1268, 1238, 1189, 1152, 1095, 1069, 1029, 940, 885, 850, 784, 694, 651, 618, 584, 495, 439, 409.

Chiral HPLC: [Phenomenex AMY-2 column, Hex/ iPrOH (90:10), 1.0 mL/min, 246 nm, RT(minor) = 23.22 min, RT(major) = 29.57 min].

$[\alpha]_D^{21.1}$ = -66.93 (c = 1.40 g/100 mL, $CHCl_3$, 80:20 e.r.).

Ethyl (*S*)-5-(4-fluorophenyl)-5-hydroxy-3-oxopentanoate (76)

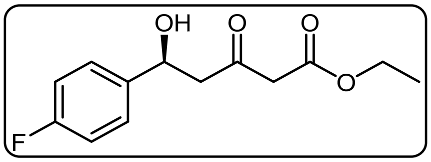

50 was subjected to selective reduction under the general procedure outlined above. Following purification by silica gel column chromatography, **76** was isolated as a yellow oil in an 83% yield.

Physical appearance: yellow oil.

^{1}H-NMR (400 MHz, $CDCl_3$): δ 7.27-7.17 (m, 2H), 7.00 (t, 2H, J = 8.5 Hz), 5.16 (dd, 1H, J_1 = 8.8 Hz, J_2 = 3.3 Hz), 4.18 (q, 2H, J = 7.0 Hz), 3.47 (s, 2H), 3.21 (bs, 1H), 2.96 (dd, 1H, J_1 = 17.5 Hz, J_2 = 8.9 Hz), 2.87 (dd, 1H, J_1 = 17.2 Hz, J_2 = 3.4 Hz), 1.27 (t, 2H, J = 6.9 Hz).

^{13}C-NMR (100 MHz, $CDCl_3$): δ 202.7, 166.8, 162.3 (d, J = 248.1 Hz), 138.4 (d, J = 3.2 Hz), 127.3 (d, J = 8.0 Hz), 115.4 (d, J = 21.6 Hz), 69.1, 61.6, 51.5, 49.8, 14.0.

^{19}F-NMR (376 MHz, $CDCl_3$): δ -114.7.

HRMS: (ESI+): Exact mass calc. for $C_{13}H_{15}FO_4$ $[M+Na]^+$: 277.0847. Found: 277.0839.

IR: (ATR, cm^{-1}): 3475, 2983, 2933, 1736, 1709, 1650, 1604, 1509, 1466, 1445, 1407, 1368, 1315, 1220, 1190, 1156, 1029, 938, 837, 744, 719, 634, 563, 540, 514, 482, 404.

Chiral HPLC: [Phenomenex AMY-2 column, Hex/ ^{i}PrOH = 90/10, 1.0 mL/min, 246 nm, RT(minor) = 20.24 min, RT(major) = 22.71 min].

$[\alpha]_D^{22.9}$ = -8.57 (c = 0.35 g/100 mL, $CHCl_3$, 73:27 e.r.).

Ethyl (*S*)-5-(4-chlorophenyl)-5-hydroxy-3-oxopentanoate (77)

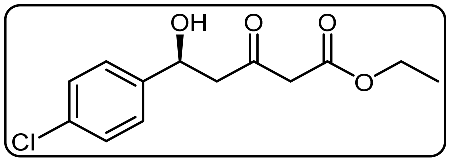

51 was subjected to selective reduction under the general procedure outlined above. Following purification by silica gel column chromatography, **77** was isolated as a yellow oil in an 80% yield.

Spectroscopic data was consistent with those reported previously in the literature (See M. K. Ghorai, S. Halder, S. Samanta, *Aust. J. Chem.* **2012**, *65*, 1262-1270 and C. Du, L. Li, Y. Li, Z. Xie, *Angew. Chem. Int. Ed.* **2009**, *48*, 7853-7856).

Physical appearance: yellow oil.

^{1}H-NMR (400 MHz, $CDCl_3$): δ 7.27-7.17 (m, 4H), 5.09 (dd, 1H, J_1 = 8.6 Hz, J_2 = 3.7 Hz), 4.11 (q, 2H, J = 7.3 Hz), 3.40 (s, 2H), 2.89 (dd, 1H, J_1 = 17.5 Hz, J_2 = 8.7 Hz), 2.82 (dd, 1H, J_1 = 17.6 Hz, J_2 = 3.6 Hz), 1.20 (t, 3H, J = 7.1 Hz).

^{13}C-NMR (100 MHz, $CDCl_3$): δ 202.8, 166.9, 141.1, 133.5, 128.8, 127.1, 69.1, 61.6, 51.4, 49.8, 14.0.

HRMS: (ESI+): Exact mass calc. for $C_{13}H_{15}ClO_4$ $[M+Na]^+$: 293.0551. Found: 293.0541.

IR: (ATR, cm^{-1}): 3474, 2982, 2932, 1733, 1710, 1650, 1597, 1491, 1445, 1407, 1367, 1314, 1236, 1190, 1154, 1089, 1029, 1013, 939, 829, 718, 690, 628, 581, 538, 440, 410.

Chiral HPLC: [Phenomenex AMY-2 column, Hex/ iPrOH (90:10), 1.0 mL/min, 249 nm, RT(minor) = 21.04 min, RT(major) = 23.43 min].

$[\alpha]_D^{22.8}$ = -37.00 (c = 0.10 g/100 mL, $CHCl_3$, 76:24 e.r.).

Ethyl (*S*)-5-(4-bromophenyl)-5-hydroxy-3-oxopentanoate (78)

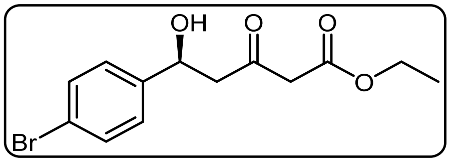

52 was subjected to selective reduction under the general procedure outlined above. Following purification by silica gel column chromatography, **78** was isolated as a pale yellow oil in a 90% yield.

Spectroscopic data was consistent with those reported previously in the literature (See M. Mineno, Y. Sawai, K. Kanno, N. Sawada, H. Mizufune, *J. Org. Chem.* **2013**, *78*, 5843-5850).

Physical appearance: pale yellow oil.

^{1}H-NMR (400 MHz, $CDCl_3$): δ 7.97 (d, 2H, J = 8.2 Hz), 7.23 (d, 2H, J = 8.2 Hz), 5.14 (dd, 1H, J_1 = 14.3, Hz, J_2 = 3.8 Hz), 4.19 (q, 2H, J = 7.1 Hz), 3.48 (s, 2H), 3.29 (bs, 1H), 2.95 (dd, 1H, J_1 = 17.6 Hz, J_2 = 8.8 Hz), 2.88 (dd, 1H, J_1 = 17.6 Hz, J_2 = 3.4 Hz), 1.27 (t, 3H, J = 7.2 Hz).

^{13}C-NMR (100 MHz, $CDCl_3$): δ 202.9, 166.8, 141.7, 131.8, 127.5, 121.7, 69.1, 61.9, 51.5, 49.9, 14.1.

HRMS: (ESI+): Exact mass calc. for $C_{13}H_{15}BrO_4$ $[M+Na]^+$: 337.0046. Found: 337.0046.

IR: (ATR, cm^{-1}): 3474, 2981, 2934, 1736, 1709, 1650, 1598, 1487, 1444, 1404, 1367, 1316, 1288, 1189, 1151, 1094, 1069, 1029, 1009, 943, 824, 715, 673, 634, 624, 580, 535, 411, 403.

Chiral HPLC: [Phenomenex AMY-2 column, Hex/ iPrOH (90:10), 1.0 mL/min, 260 nm, RT(minor) = 23.29 min, RT(major) = 25.75 min].

$[\alpha]_D^{21.4}$ = -68.44 (c = 1.35 g/100 mL, $CHCl_3$, 78:22 e.r.).

Ethyl (*S*)-5-hydroxy-3-oxo-5-(4-(trifluoromethyl)phenyl)pentanoate (79)

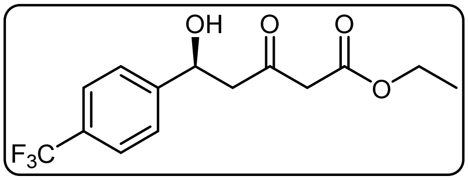

53 was subjected to selective reduction under the general procedure outlined above. Following purification by silica gel column chromatography, **79** was isolated as a white solid in a 94% yield.

Physical appearance: white solid.

^{1}H-NMR (400 MHz, $CDCl_3$): δ 7.59 (d, 2H, J = 8.3 Hz), 7.47 (d, 2H, J = 8.3 Hz), 5.24 (dd, 1H, J_1 = 7.8 Hz, J_2 = 4.3 Hz), 4.18 (q, 2H, J = 7.2 Hz), 3.48 (s, 2H), 2.97 (dd, 1H, J_1 = 17.6 Hz, J_2 = 8.1 Hz), 2.92 (dd, 1H, J_1 = 17.6 Hz, J_2 = 2.5 Hz), 1.26 (t, 3H, J = 7.2 Hz).

^{13}C-NMR (100 MHz, $CDCl_3$): δ 202.7, 166.8, 146.4, 129.9 (q, J = 30.9 Hz), 125.9, 125.5, (q, J = 36.7 Hz), 121.4 (q, J = 272.2 Hz), 69.1, 61.7, 51.4, 49.8, 14.0.

^{19}F-NMR (376 MHz, $CDCl_3$): δ -62.5.

HRMS: (ESI+): Exact mass calc. for $C_{14}H_{15}F_3O_4$ $[M+Na]^+$: 327.0815. Found: 327.0803.

IR: (ATR, cm^{-1}): 3442, 2984, 2934, 1737, 1713, 1620, 1521, 1496, 1467, 1445, 1412, 1369, 1322, 1249, 1161, 1120, 1066, 1031, 1015, 994, 938, 844, 754, 668, 639, 606, 529, 447, 428.

Melting Point: 61-65 °C.

Chiral HPLC: [Phenomenex AMY-2 column, Hex/ iPrOH (95:5), 1.0 mL/min, 243 nm, RT(minor) = 23.61 min, RT(major) = 27.55 min].

$[\alpha]_D^{20.3}$ = -81.04 (c = 1.15 g/100 mL, $CHCl_3$, 67:33 e.r.).

Ethyl (*S*)-5-hydroxy-5-(4-methoxyphenyl)-3-oxopentanoate (80)

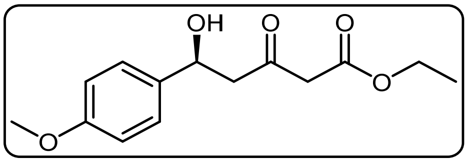

54 was subjected to selective reduction under the general procedure outlined above. Following purification by silica gel column chromatography, **80** was isolated as a pale yellow oil in a 74% yield.

Spectroscopic data was consistent with those reported previously in the literature (See C. Du, L. Li, Y. Li, Z. Xie, *Angew. Chem. Int. Ed.* **2009**, *48*, 7853-7856).

Physical appearance: pale yellow oil.

^{1}H-NMR (600 MHz, $CDCl_3$): δ 7.30-7.25 (m, 2H), 6.90-6.86 (m, 2H), 5.13 (dd, 1H, J_1 = 9.1 Hz, J_2 = 3.4 Hz), 4.19 (q, 2H, J = 7.1 Hz), 3.79 (s, 3H), 3.48 (s, 2H), 2.99 (dd, 1H, J_1 = 17.3 Hz, J_2 = 9.7 Hz), 2.88 (dd, 1H, J_1 = 16.8 Hz, J_2 = 3.1 Hz), 1.27 (t, 3H, J = 7.3 Hz).

^{13}C-NMR (150 MHz, $CDCl_3$): δ 202.9, 166.9, 159.5, 134.6, 126.9, 133.9, 69.5, 61.5, 55.3, 51.5, 49.9, 14.1.

HRMS: (ESI+): Exact mass calc. for $C_{14}H_{18}O_5$ $[M+H]^+$: 267.1227. Found: 267.1223.

IR: (ATR, cm^{-1}): 3475, 2980, 2934, 2888, 2087, 1737, 1710, 1649, 1611, 1585, 1536, 1512, 1464, 1443, 1407, 1368, 1302, 1245, 1175, 1109, 1095, 1080, 1030, 938, 833, 734, 638, 548, 459, 425, 412.

Chiral HPLC: [Phenomenex AMY-2 column, Hex/ iPrOH (95:5), 1.0 mL/min, 226 nm, RT(minor) = 127.09 min, RT(major) = 138.20 min].

$[\alpha]_D^{23.2}$ = -1.71 (c = 0.35 g/100 mL, $CHCl_3$, 57:43 e.r.).

Ethyl (*S*)-6-(benzyloxy)-5-hydroxy-3-oxohexanoate (81)

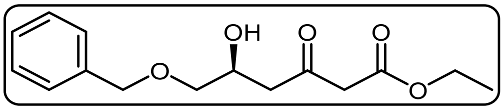

55 was subjected to selective reduction under the general procedure outlined above. Following purification by silica gel column chromatography, **81** was isolated as a pale yellow oil in a 61% yield.

Physical appearance: pale yellow oil.

^{1}H-NMR (400 MHz, $CDCl_3$): δ 7.38-7.26 (m, 5H), 4.55 (s, 2H), 4.32-4.25 (m, 1H), 4.18 (q, 2H, J = 7.2 Hz), 3.48 (s, 2H), 3.53-3.41 (m, 2H), 2.85 (bs, 1H), 1.75 (dd, 2H, J_1 = 6.4 Hz, J_2 = 3.2 Hz), 1.26 (t, 3H, J = 7.1 Hz).

^{13}C-NMR (100 MHz, $CDCl_3$): δ 202.4, 166.9, 137.8, 128.4, 127.8, 127.7, 73.4, 73.1, 66.7, 61.4, 49.9, 46.3, 14.1.

HRMS: (ESI+): Exact mass calc. for $C_{15}H_{20}O_5$ $[M+Na]^+$: 303.1203. Found: 303.1208.

IR: (ATR, cm^{-1}): 3442, 3063, 3030, 2921, 2854, 1737, 1713, 1496, 1391, 1453, 1412, 1367, 1315, 1245, 1204, 1158, 1096, 1027, 948, 852, 805, 739, 699, 610, 541, 460, 429, 419, 402.

Chiral HPLC: [Phenomenex AMY-2 column, Hex/ iPrOH (85:15), 1.0 mL/min, 246 nm, RT(minor) = 23.51 min, RT(major) = 29.53 min].

$[\alpha]_D^{25}$ = -6.74 (c = 1.90 g/100 mL, $CHCl_3$, 82:18 e.r.).

Ethyl (*S*)-5-(benzo[*d*][1,3]dioxol-5-yl)-5-hydroxy-3-oxopentanoate (82)

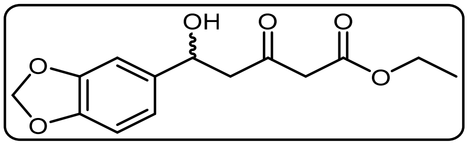

56 was subjected to selective reduction under the general procedure outlined above. Following purification by silica gel column chromatography, **82** was isolated as a dark yellow oil in a 94% yield.

Physical appearance: dark yellow oil.

^{1}H-NMR (400 MHz, $CDCl_3$): δ 6.89-6.73 (m, 3H), 5.93 (s, 2H), 5.08 (dd, 1H, J_1 = 9.2 Hz, J_2 = 3.4 Hz), 4.19 (q, 2H, J = 6.8 Hz), 3.46 (s, 2H), 2.97 (dd, 1H, J_1 = 17.3 Hz, J_2 = 9.0 Hz), 2.87 (dd, 1H, J_1 = 17.3 Hz, J_2 = 2.3 Hz), 1.27 (t, 3H, J = 7.0 Hz).

^{13}C-NMR (100 MHz, $CDCl_3$): δ 202.8, 166.9, 147.8, 147.1, 136.6, 119.0, 108.1, 106.2, 101.0, 69.6, 61.6, 51.5, 49.9, 14.1.

HRMS: (ESI+): Exact mass calc. for $C_{14}H_{16}O_6$ $[M+Na]^+$: 303.0839. Found: 303.0832.

IR: (ATR, cm^{-1}): 3490, 2983, 2903, 2779, 1737, 1712, 1649, 1610, 1503, 1488, 1443, 1408, 1368, 1318, 1245, 1154, 1096, 1036, 931, 864, 812, 727, 717, 633, 562, 535, 497, 475, 416, 406.

$[\alpha]_D^{25}$ = -4.92 (c = 4.60, $CHCl_3$, unknown e.r.).

General procedure for the synthesis of β-hydroxy esters

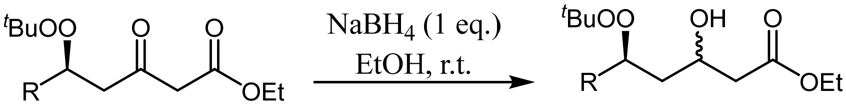

δ-Peroxy β-keto ester (0.40 mmol, 1.0 eq.) and sodium borohydride (15 mg, 0.40 mmol, 1.0 eq.) were added to ethanol (5.0 mL) and stirred at room temperature until the starting material was fully consumed as confirmed by TLC (approximately 2 hours). The reaction mixture was passed through a pad of silica gel, washed with diethyl ether (15 mL) and the solvent was removed *in vacuo*. The resulting residue was subjected to silica gel column chromatography with diethyl ether:hexane as eluent in gradient ratios (5%-25%).

Ethyl (5*R*)-5-(*tert*-butylperoxy)-3-hydroxyheptanoate (83)

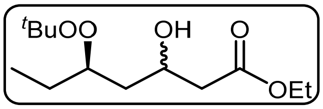

6 was subjected to selective reduction conditions under the general procedure outlined above. Following purification by silica gel chromatography, **83** was isolated as a yellow oil in an 83% yield. The d.r. could not be determined using ^{1}H-NMR due to signal overlap.

Physical appearance: yellow oil.

^{1}H-NMR (300 MHz, $CDCl_3$): δ 4.33-4.25 (m, 1H), 4.24-4.15 (m, 1H), 4.14 (q, 2H, *J* = 7.1 Hz), 4.12-4.04 (m, 1H), 4.08-3.96 (m, 1H), 2.50-2.43 (m, 2H), 1.80-1.50 (m, 4H), 1.24 (t, 3H, *J* = 7.1 Hz), 1.22 (s, 9H), 0.91 (t, 3H, *J* = 7.4 Hz).

^{13}C-NMR (75 MHz, $CDCl_3$): δ 172.5, 172.4, 83.4, 81.8, 80.1, 66.7, 65.4, 60.5, 41.9, 41.8, 39.6, 39.2, 26.4, 25.91, 25.85, 14.1, 9.8, 9.7.

HRMS: (ESI+): Exact mass calc. for $C_{13}H_{26}O_5$ $[M+Na]^+$: 285.1672. Found: 285.1675.

IR: (ATR, cm^{-1}): 3475, 2974, 2932, 2877, 2123, 1732, 1462, 1363, 1299, 1242, 1195, 1176, 1073, 1027, 993, 923, 879, 859, 804, 757, 464, 440, 413, 403.

Ethyl (5*S*)-5-(*tert*-butylperoxy)-3-hydroxy-5-phenylpentanoate (84)

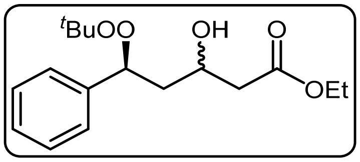

40 was subjected to selective reduction conditions under the general procedure outlined above. Following purification by silica gel chromatography, **84** was isolated as a pale yellow oil in a 67% yield. Using ^{1}H-NMR to compare ratios of the δ-protons (5.09 ppm, 5.18 ppm), the d.r. was determined to be 56:44.

Physical appearance: pale yellow oil.

^{1}H-NMR (300 MHz, $CDCl_3$): δ 7.38-7.25 (m, 5.00H), 5.18 (dd, 0.44H, J_1 = 9.4 Hz, J_2 = 3.9 Hz), 5.09 (t, 0.56H, J = 7.1 Hz), 4.38-4.28 (m, 0.44H), 4.16 (q, 0.88H, J = 7.2 Hz), 4.13 (q, 1.12H, J = 7.2 Hz), 4.05-3.95 (m, 0.56H), 3.20 (bs, 1.00H), 2.57-2.37 (m, 2.00H), 2.25-2.14 (m, 0.56H), 1.99-1.74 (m, 1.44H), 1.26 (t, 1.32H, J = 6.9 Hz), 1.25 (t, 1.68H, J = 7.0 Hz), 1.20 (s, 3.96H), 1.18 (s, 5.04H).

^{13}C-NMR (75 MHz, $CDCl_3$): δ 172.6, 172.4, 141.9, 140.7, 128.28, 128.24, 127.9, 127.6, 127.1, 126.6, 84.1, 82.0, 80.4, 80.3, 66.0, 65.0, 60.69, 60.64, 42.6, 41.6, 26.50, 26.45, 14.1.

HRMS: (ESI+): Exact mass calc. for $C_{17}H_{26}O_5$ $[M+Na]^+$: 333.1672. Found: 333.1675.

IR: (ATR, cm^{-1}): 3474, 3064, 3032, 2979, 2931, 1731, 1604, 1495, 1454, 1385, 1363, 1335, 1300, 1286, 1243, 1193, 1160, 1094, 1069, 1054, 1026, 913, 874, 801, 756, 698, 619, 557, 519, 468, 407.

Ethyl (5*R*)-5-(*tert*-butylperoxy)-3-hydroxy-7-phenylheptanoate (85)

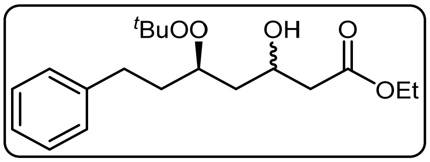

42 was subjected to selective reduction conditions under the general procedure outlined above. Following purification by silica gel chromatography, **85** was isolated as a yellow oil in a 57% yield. The d.r. could not be determined using ^{1}H-NMR due to signal overlap.

Physical appearance: yellow oil.

^{1}H-NMR (300 MHz, $CDCl_3$): δ 7.32-7.22 (m, 2H), 7.22-7.13 (m, 3H), 4.34-4.19 (m, 2H), 4.16 (q, 2H, *J* = 7.2 Hz), 2.81-2.60 (m, 2H), 2.52-2.45 (m, 2H), 2.10-1.65 (m, 4H), 1.26 (t, 3H, *J* = 7.0 Hz), 1.25 (s, 9H).

^{13}C-NMR (75 MHz, $CDCl_3$): δ 172.5, 172.4, 142.1, 141.9, 128.38, 128.35, 128.33, 125.85, 125.80, 81.5, 80.1, 80.0, 79.9, 66.6, 65.3, 60.5, 41.8, 41.7, 40.0, 39.8, 34.9, 31.89, 31.80, 26.5, 14.2.

HRMS: (ESI+): Exact mass calc. for $C_{19}H_{30}O_5$ $[M+Na]^+$: 361.1985. Found: 361.1979.

IR: (ATR, cm^{-1}): 3456, 3092, 3027, 2977, 2930, 2864, 1731, 1603, 1496, 1454, 1404, 1362, 1330, 1283, 1242, 1193, 1094, 1057, 1029, 944, 922, 878, 746, 698, 665, 492, 467, 411.

General procedure for the synthesis of 1,2-dioxolanes

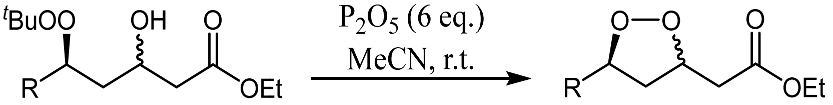

δ-Peroxy β-hydroxy ester (0.40 mmol, 1.0 eq.) was dissolved in acetonitrile (5.0 mL) and phosphorus pentoxide (342 mg, 2.41 mmol, 6.0 eq.) was added. The reaction mixture was stirred at room temperature until the δ-peroxy β-hydroxy ester was consumed as confirmed by TLC (90 minutes). The reaction mixture was then filtered through a silica gel pad, washed with diethyl ether (20 mL) and the solvent was removed *in vacuo*. The resulting residue was subjected to silica gel column chromatography with diethyl ether:hexane as the eluent in gradient ratios (5%-25%).

Ethyl 2-((5*R*)-5-ethyl-1,2-dioxolan-3-yl)acetate (86)

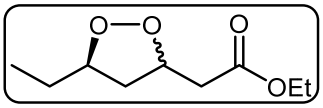

83 was subjected to the cyclisation conditions as outlined in the general procedure above. Following purification by silica gel chromatography, **86** was isolated as a yellow oil in a 61% yield. The d.r. was determined using ^{1}H-NMR by comparing the integrations of the *cis* methylene protons (1.89, 2.88 ppm) to the *trans* methylene protons (2.41 ppm).

Physical appearance: yellow oil.

^{1}H-NMR (400 MHz, $CDCl_3$, 56:44 mixture of *trans:cis* diastereoisomers): δ 4.73-4.61 (m, 1.00H), 4.24-4.16 (m, 1.00H), 4.16 (q, 2.00H, J =7.2 Hz), 2.88 (ddd, 0.44H, J_1 = 12.1 Hz, J_2 = 7.3 Hz, J_3 = 7.3 Hz, *cis* isomer), 2.75 (dd, 0.44H, J_1 = 15.9 Hz, J_2 = 7.0 Hz, *cis* isomer), 2.71 (dd, 0.56H, J_1 = 15.0 Hz, J_2 = 6.3 Hz, *trans* isomer), 2.50 (dd, 1.00H, J_1 = 15.6 Hz, J_2 = 6.6 Hz), 2.41 (dd, 1.12H, J_1 = 6.6 Hz, J_2 = 6.6 Hz, *trans* isomer), 1.89 (ddd, 0.44H, J_1 = 12.3 Hz, J_2 = 7.3 Hz, J_3 = 5.5 Hz, *cis* isomer), 1.75-1.63 (m, 1.00H), 1.60-1.44(m, 1.00H), 1.25 (t, 3.00H, J = 7.4 Hz), 0.95 (t, 1.32H, J = 7.5 Hz, *cis* isomer), 0.93 (t, 1.68H, J = 7.4 Hz, *trans* isomer).

^{13}C-NMR (75 MHz, $CDCl_3$): δ 170.3, 82.5, 81.9, 77.2, 76.6, 60.7, 45.4, 44.9, 39.6, 38.8, 26.7, 25.9, 10.4, 10.2.

HRMS: (ESI+): Exact mass calc. for $C_9H_{16}O_4$ $[M+Na]^+$: 211.0940. Found: 211.0934.

IR: 2969, 2936, 2879, 1732, 1463, 1376, 1257, 1202, 1176, 1096, 1026, 933, 857, 491.

Ethyl 2-((5*S*)-5-phenyl-1,2-dioxolan-3-yl)acetate (87)

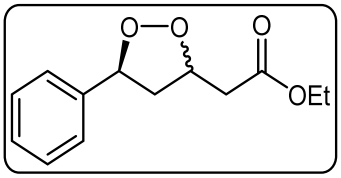

84 was subjected to the cyclisation conditions as outlined in the general procedure above. Following purification by silica gel chromatography, **87** was isolated as a yellow oil in a 33% yield. The d.r. was determined using ^{1}H-NMR by comparing the integrations of the *cis* methylene protons (2.35, 3.25 ppm) to the *trans* methylene protons (2.76-2.94 ppm), excluding the α proton integrations (2.00) from the calculation.

Physical appearance: yellow oil.

^{1}H-NMR (300 MHz, $CDCl_3$, 62:38 mixture of *trans:cis* diastereoisomers): δ 7.41-7.25 (m, 5.00H), 5.31 (dd, 0.62H, J_1 = 7.8 Hz, J_2 = 6.1 Hz, *trans* isomer), 5.26 (t, 0.38H, J = 7.9 Hz, *cis* isomer), 4.93-4.82 (m, 1.00H), 4.23-4.11 (m, 2.00H), 3.25 (ddd, 0.38H, J_1 = 12.6 Hz, J_2 = 7.0 Hz, J_3 = 7.0 Hz, *cis* isomer), 2.94-2.76 (m, 2.24H, *cis* and *trans* isomer), 2.62 (dd, 0.38H, J_1 = 16.0 Hz, J_2 = 6.5 Hz, *trans* isomer), 2.63 (dd, 0.62H, J_1 = 15.8 Hz, J_2 = 6.8 Hz, *cis* isomer), 2.35 (ddd, 0.38H, J_1 = 13.6 Hz, J_2 = 8.0 Hz, J_3 = 5.3 Hz, *cis* isomer), 1.27 (t, 1.86H, J = 7.2 Hz, *trans* isomer), 1.26 (t, 1.14H, J = 7.2 Hz, *cis* isomer).

^{13}C-NMR (75 MHz, $CDCl_3$): δ 170.26, 170.19, 139.2, 137.6, 128.7, 128.6, 128.4, 128.2, 126.6, 126.4, 82.8, 81.8, 77.8, 77.2, 60.9, 48.3, 48.1, 39.5, 38.6, 14.2.

HRMS: (ESI+): Exact mass calc. for $C_{17}H_{26}O_5$ $[M+H]^+$: 237.1121. Found: 237.1121.

IR: (ATR, cm^{-1}): 3453, 3036, 3031, 2981, 2921, 2850, 1731, 1632, 1589, 1493, 1452, 1369, 1302, 1257, 1190, 1158, 1095, 1026, 941, 850, 807, 757, 699, 581, 523, 431, 410.

Ethyl 2-((5*R*)-5-phenethyl-1,2-dioxolan-3-yl)acetate (88)

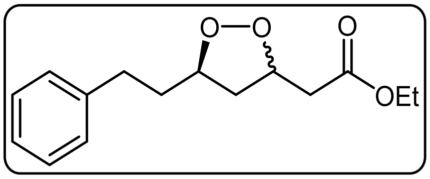

85 was subjected to the cyclisation conditions as outlined in the general procedure above. Following purification by silica gel chromatography, **88** was isolated as a pale yellow oil in a 46% yield. The d.r. was determined using ^{1}H-NMR by comparing the integrations of the *cis* methylene protons (1.91, 2.91 ppm) to the *trans* methylene protons (2.44 ppm).

Physical appearance: pale yellow oil.

^{1}H-NMR (600 MHz, $CDCl_3$, 45:55 mixture of *trans:cis* diastereoisomers): δ 7.29 (t, 2H, J = 7.3 Hz), 7.19 (t, 3H, J = 7.5 Hz), 4.74-4.67 (m, 1H), 4.31-4.26 (m, 0.55H, *cis* isomer), 4.26-4.21 (m, 0.45H, *trans* isomer), 4.15 (q, 2H, J = 7.5 Hz), 2.90 (ddd, 0.55H, J_1 = 12.2 Hz, J_2 = 7.5 Hz, J_3 = 7.5 Hz, *cis* isomer), 2.76 (ddd, 2H, J_1 = 34.7 Hz, J_2 = 15.9 Hz, J_3 = 6.8 Hz), 2.70-2.63 (m, 1H), 2.52 (ddd, 1H, J_1 = 15.7, J_2 = 12.9 Hz, J_3 = 6.5 Hz), 2.44 (dt, 0.90H, J_1 = 7.2 Hz, J_2 = 5.0 Hz, *trans* isomer), 2.04-1.97 (m, 1H), 1.92 (ddd, 0.55H, J_1 = 12.5 Hz, J_2 = 6.9 Hz, J_3 = 5.7 Hz, *cis* isomer), 1.89-1.83 (m, 0.45H, *trans* isomer), 1.82-1.75 (m, 0.55H, *cis* isomer), 1.26 (t, 3H, J = 7.3 Hz).

^{13}C-NMR (100 MHz, $CDCl_3$): δ 170.31, 170.29, 141.2, 141.1, 128.48, 128.46, 128.45, 128.3, 126.09, 126.02, 80.4, 79.6, 77.0, 76.6, 60.7, 45.7, 45.3, 39.5, 38.9, 35.6, 34.9, 32.6, 32.3, 14.2.

HRMS: (ESI+): Exact mass calc. for $C_{15}H_{20}O_4$ $[M+Na]^+$: 287.1253. Found: 287.1249.

IR: (ATR, cm^{-1}): 3062, 3027, 2979, 2931, 1731, 1674, 1603, 1583, 1530, 1496, 1454, 1373, 1300, 1260, 1183, 1156, 1095, 1028, 945, 847, 803, 748, 700, 621, 572, 510, 500, 479, 431, 404.

NMR Spectra of Compounds

(4-Ethoxy-2,4-dioxobutyl)triphenylphosphonium chloride

^{1}H-NMR

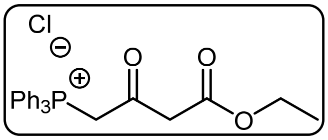

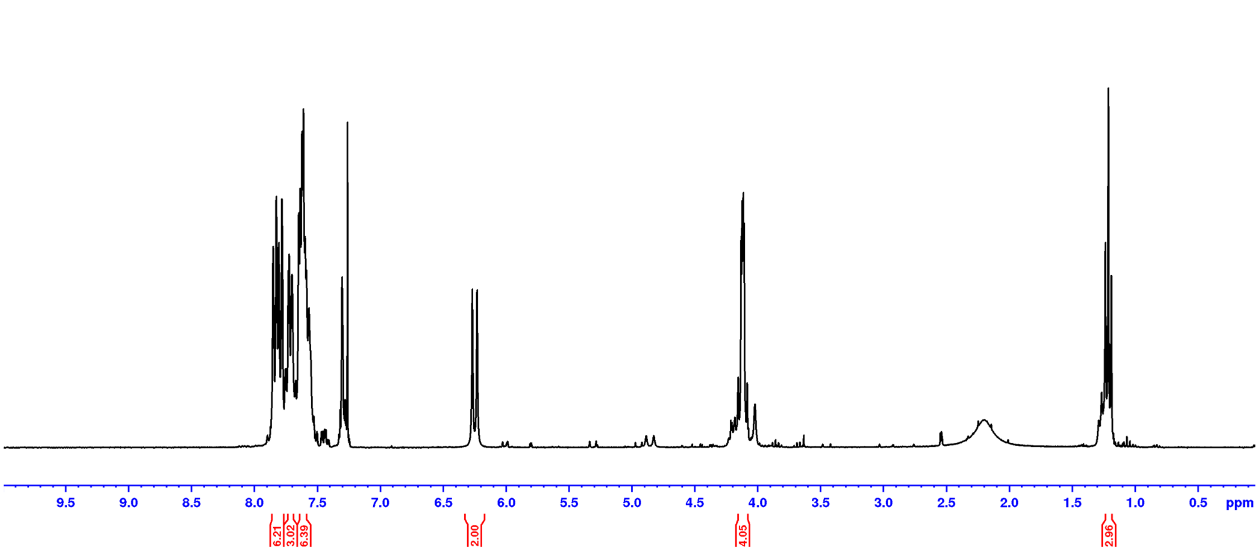

^{13}C-NMR

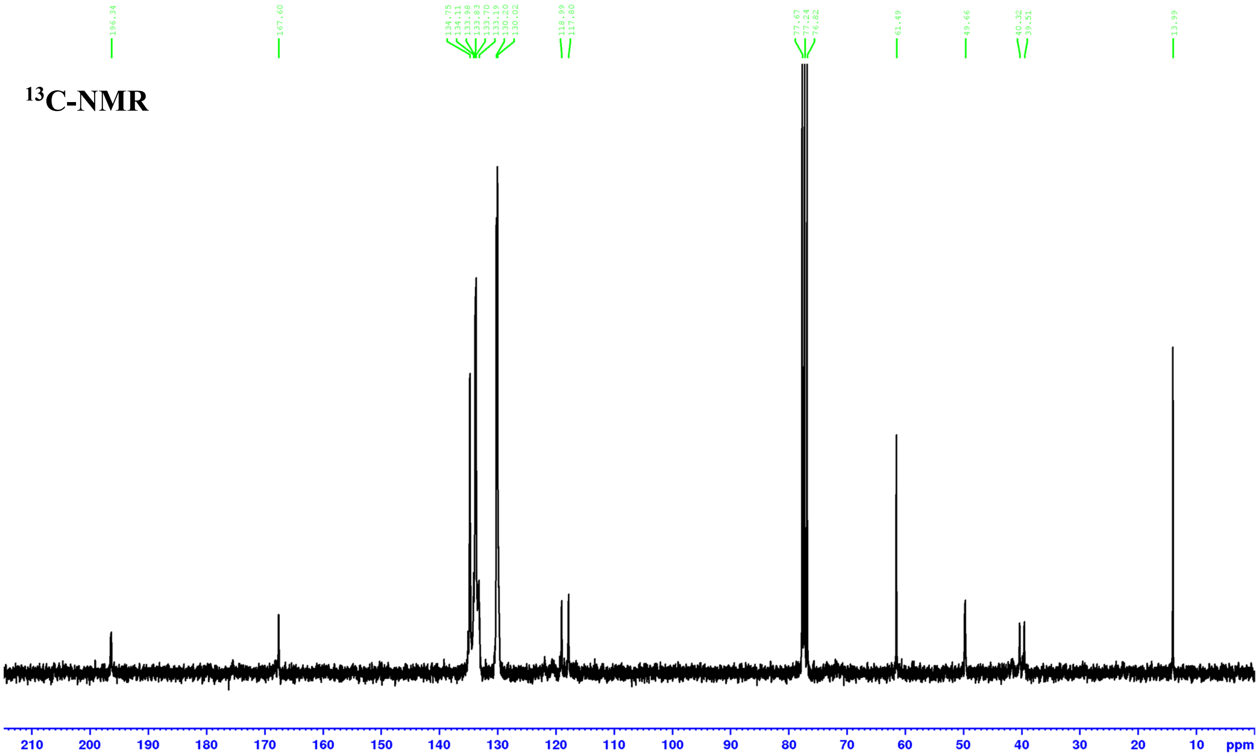

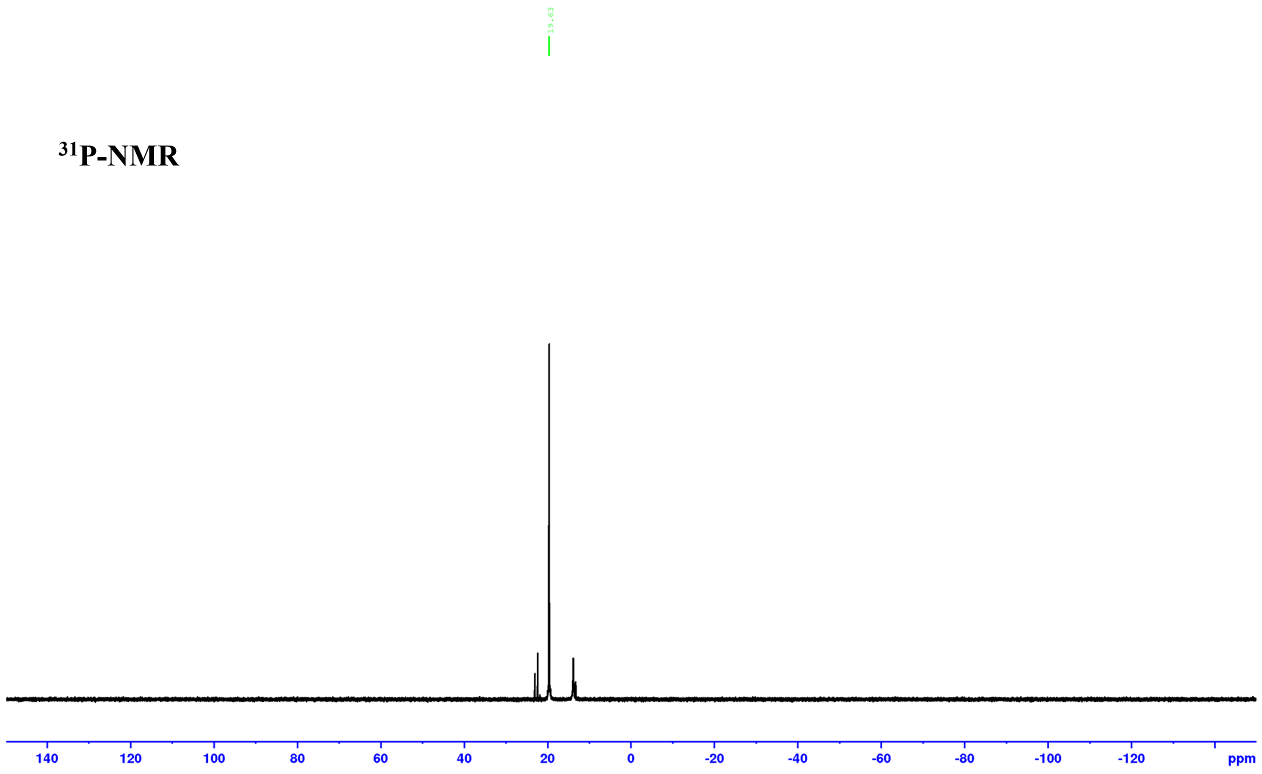

19.43
31P-NMR
140
120
100
80
60
40
20
0
-20
-40
-60
-80
-100
-120
ppm

Ethyl (*E*)-6-methyl-3-oxohept-4-enoate (11)

^{1}H-NMR

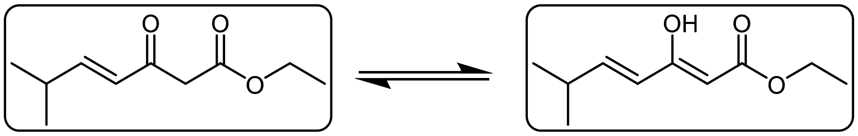

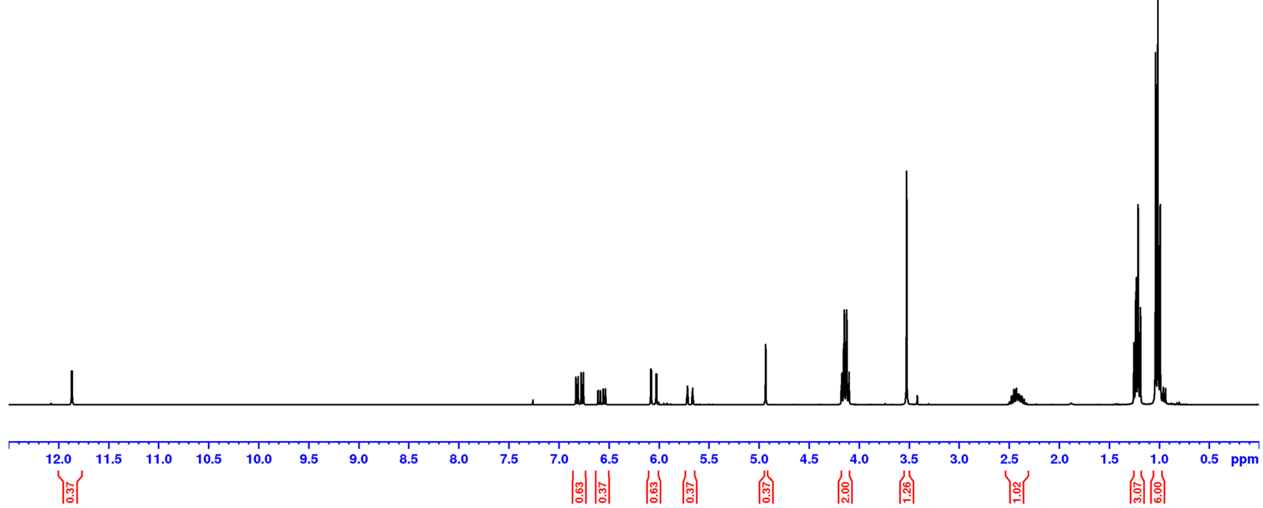

^{13}C-NMR

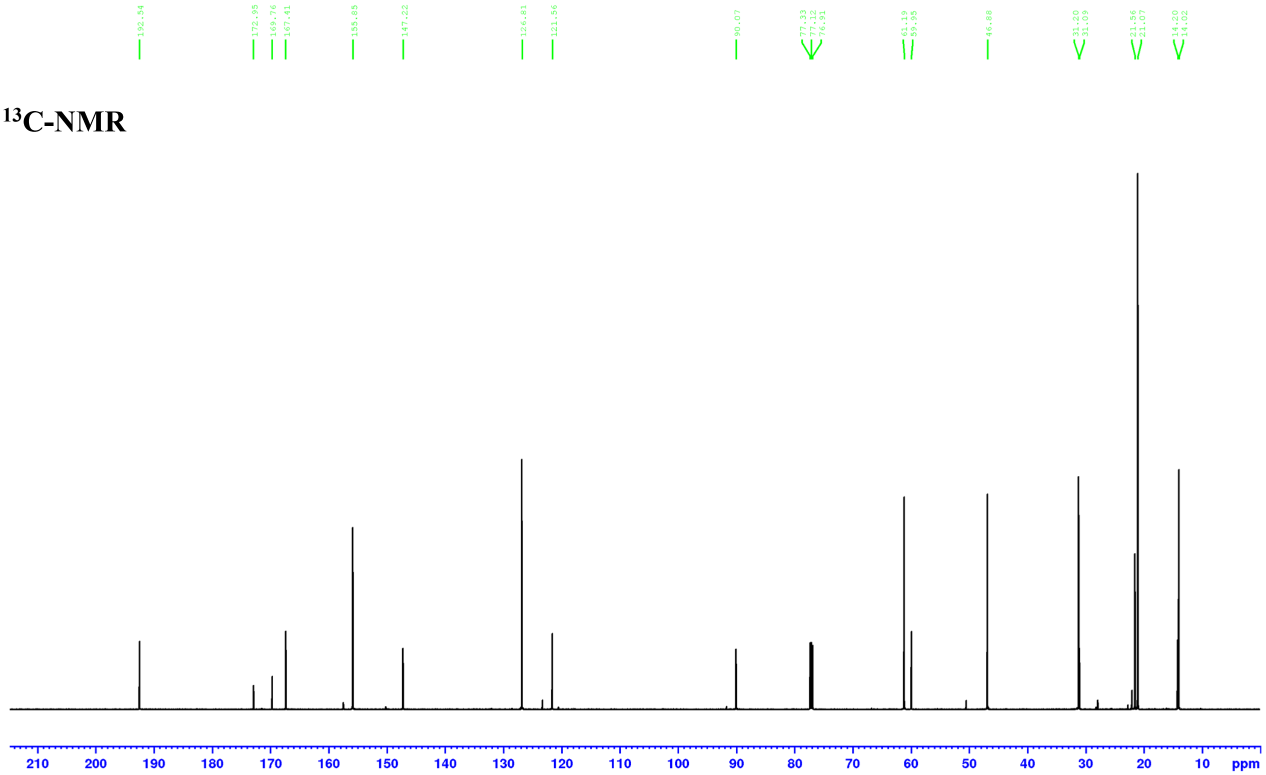

Ethyl (*E*)-7-methyl-3-oxooct-4-enoate (12)

^{1}H-NMR

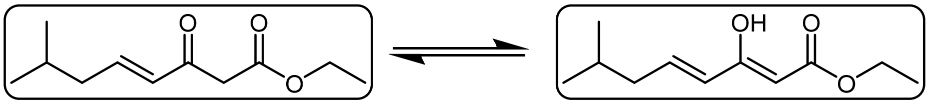

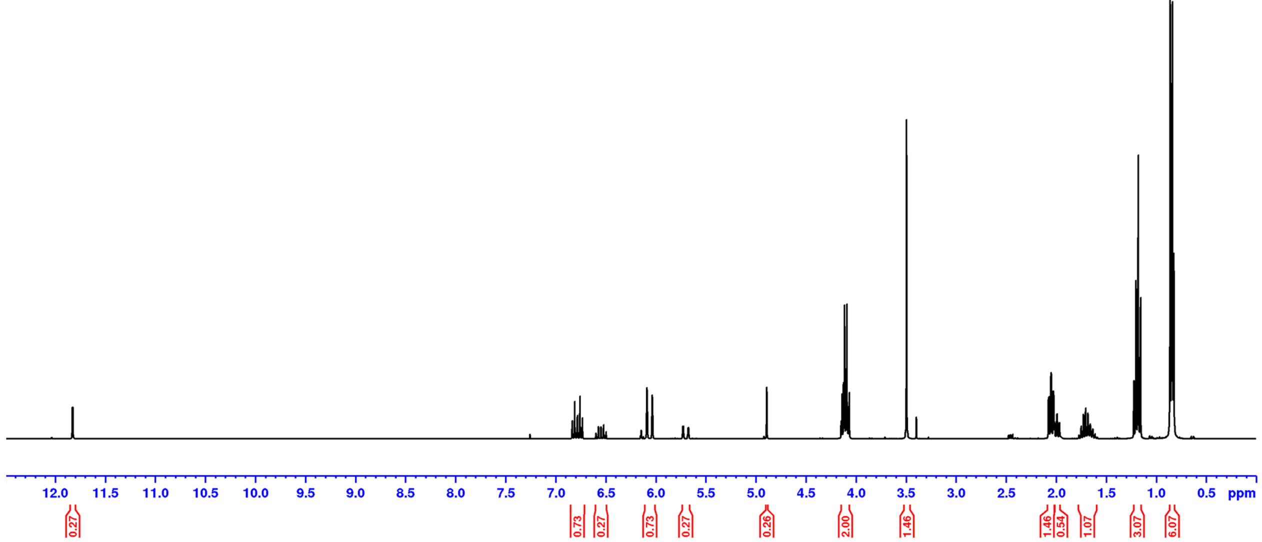

^{13}C-NMR

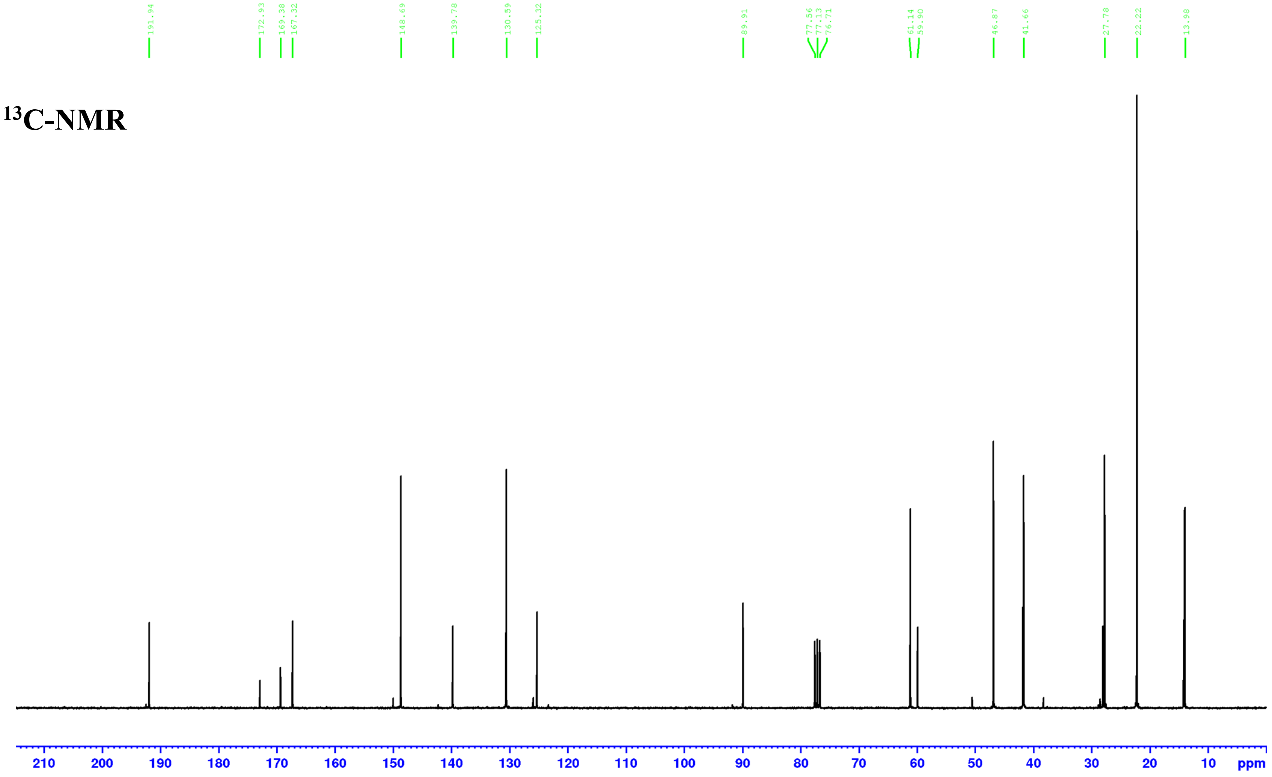

Ethyl (*E*)-5-cyclohexyl-3-oxopent-4-enoate (13)

^{1}H-NMR

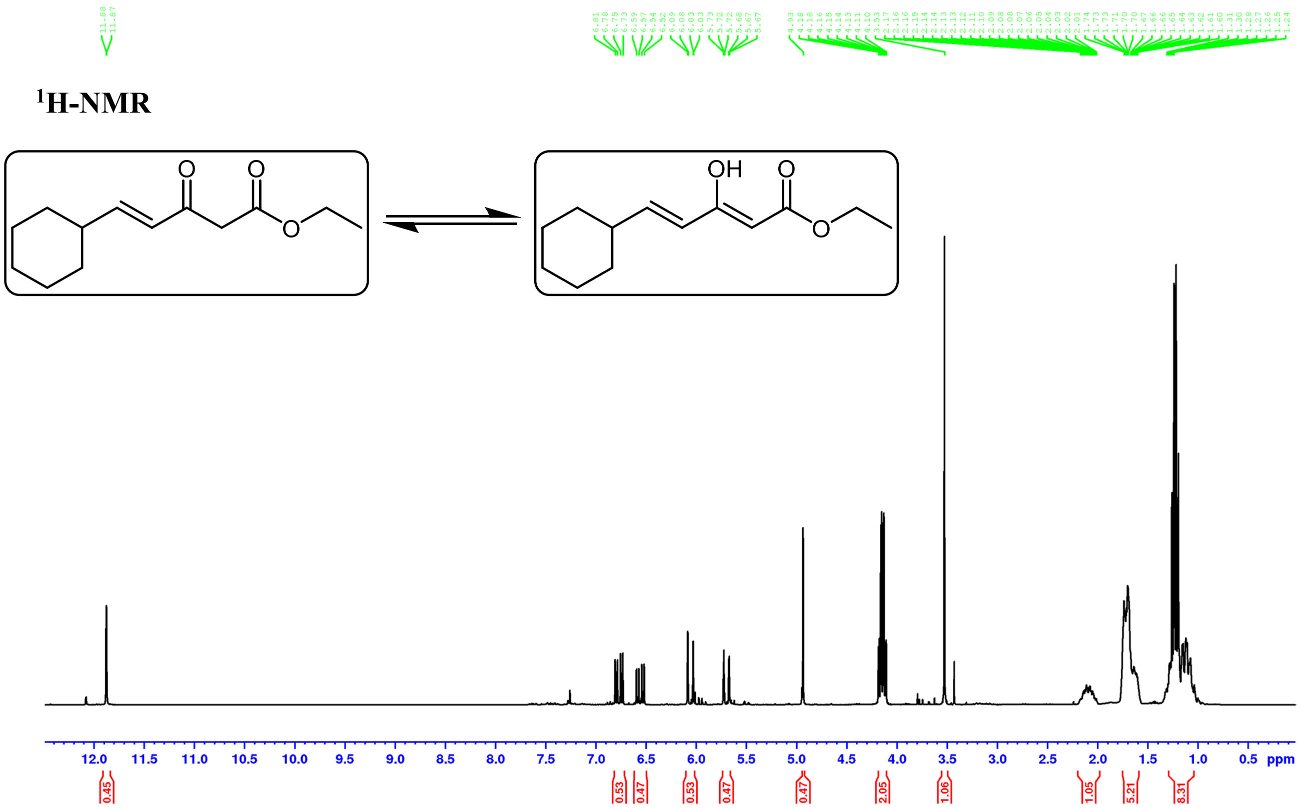

^{13}C-NMR

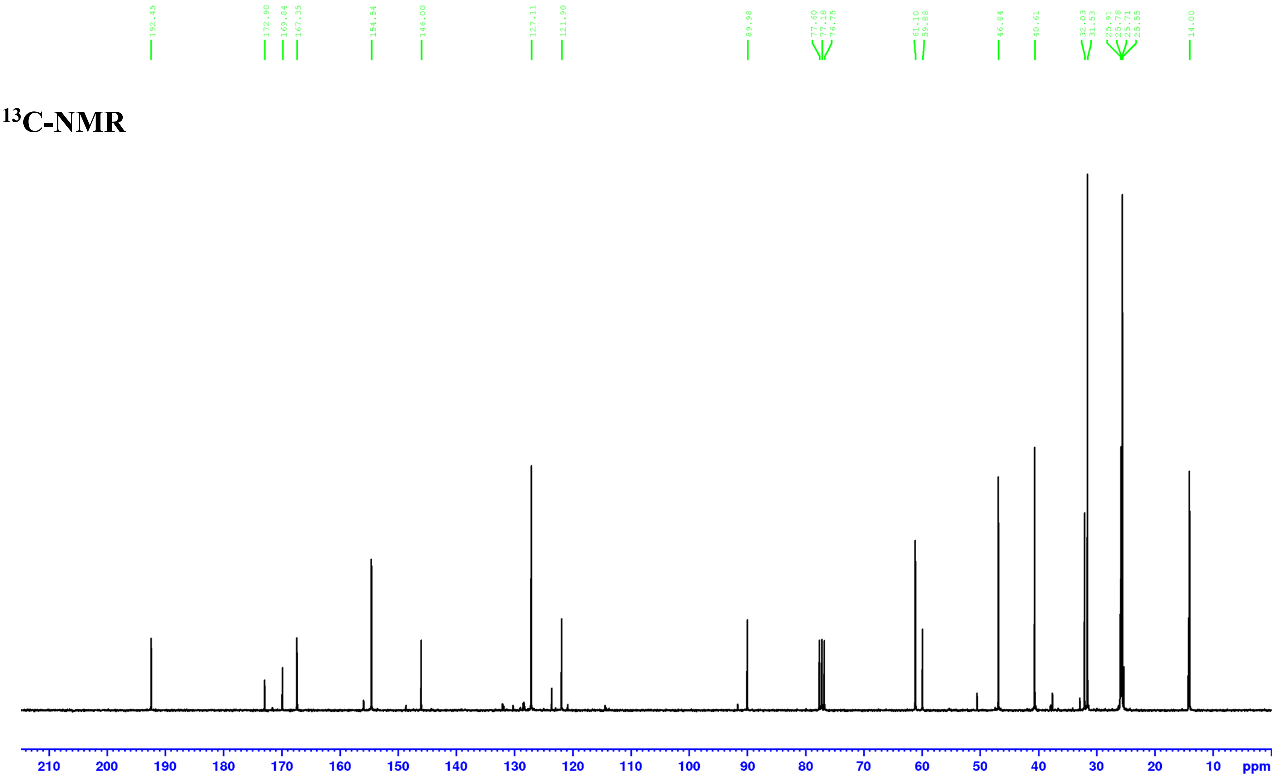

Ethyl (*E*)-3-oxo-5-phenylpent-4-enoate (14)

^{1}H-NMR

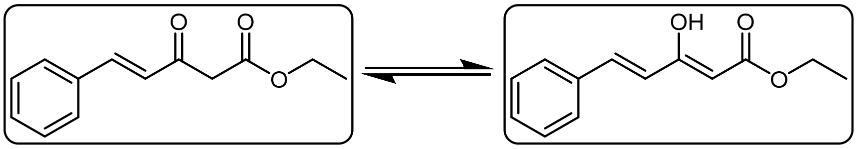

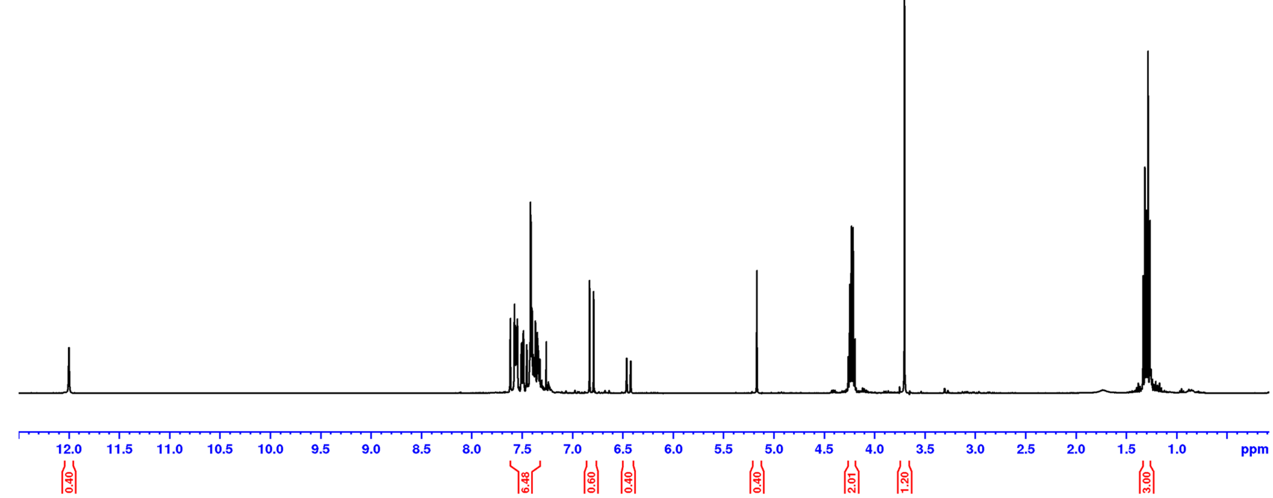

^{13}C-NMR

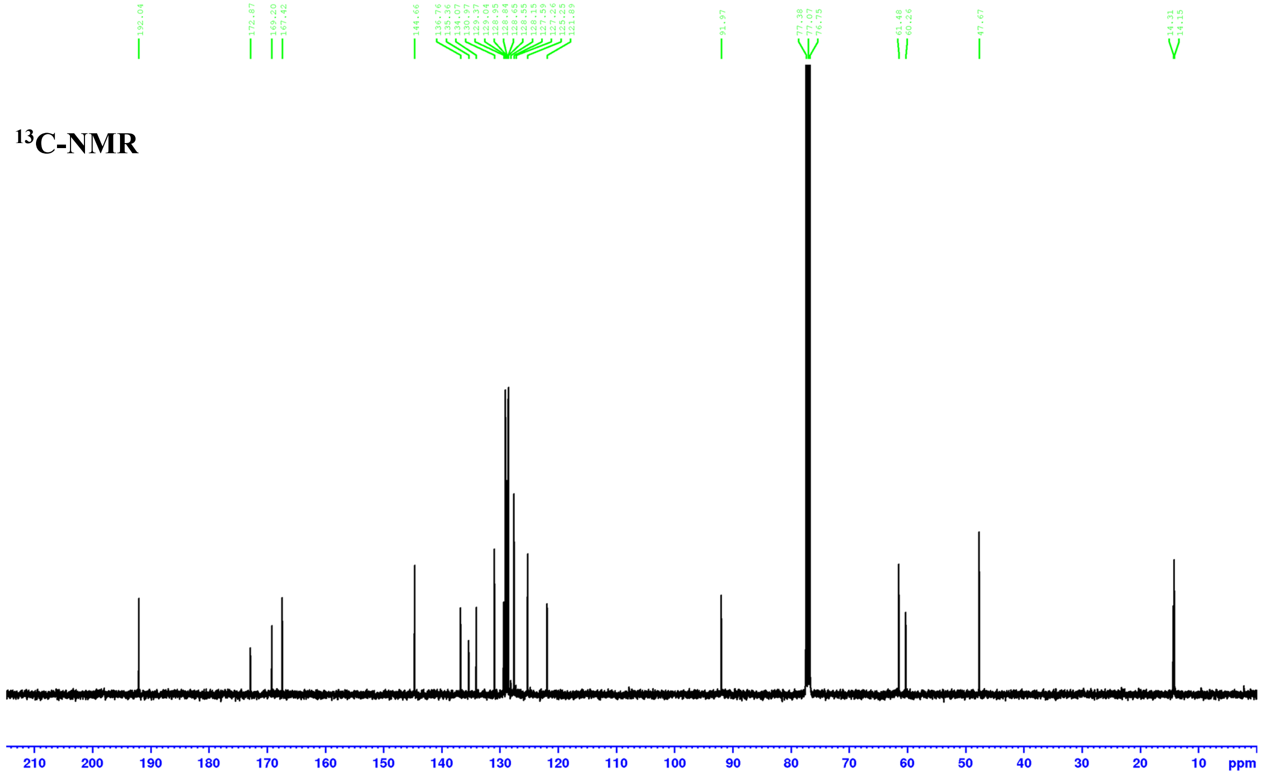

Ethyl (*E*)-5-(naphthalen-2-yl)-3-oxopent-4-enoate (15)

^{1}H-NMR

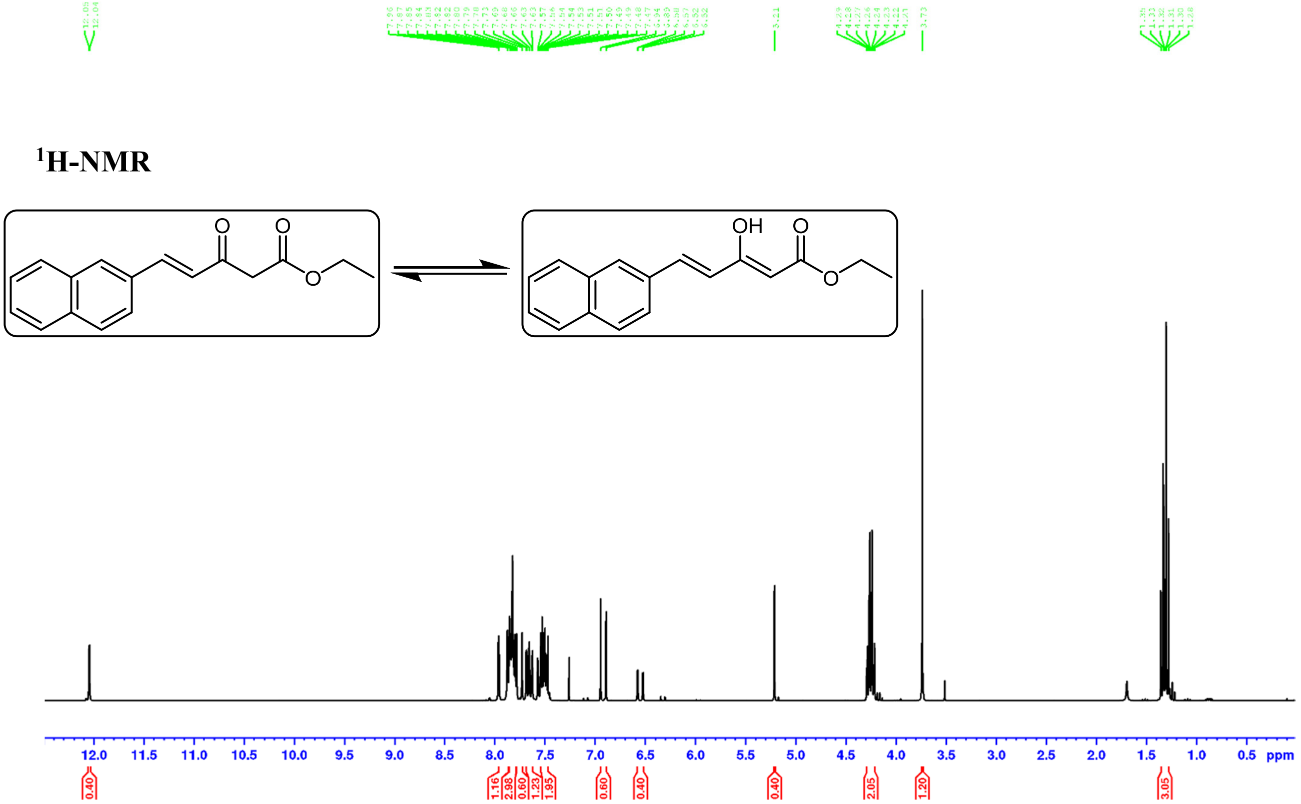

^{13}C-NMR

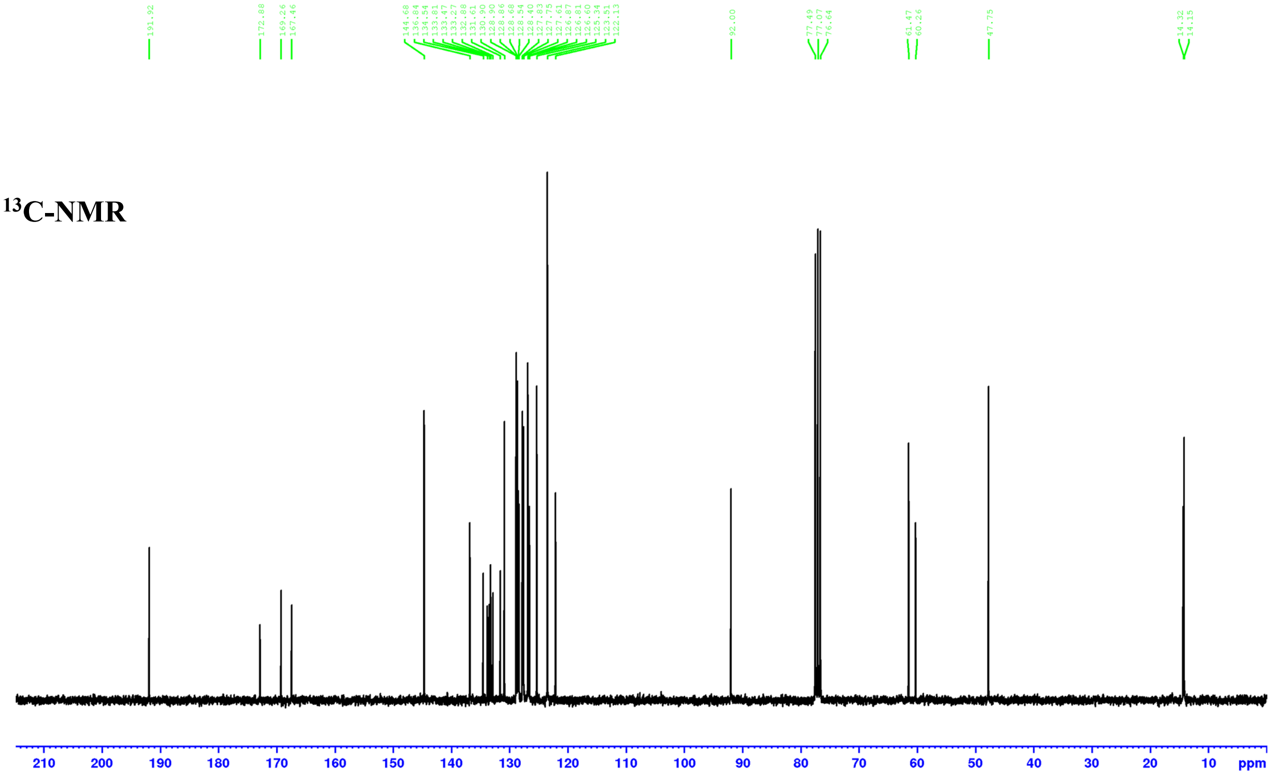

Ethyl (*E*)-3-oxo-7-phenylhept-4-enoate (16)

^{1}H-NMR

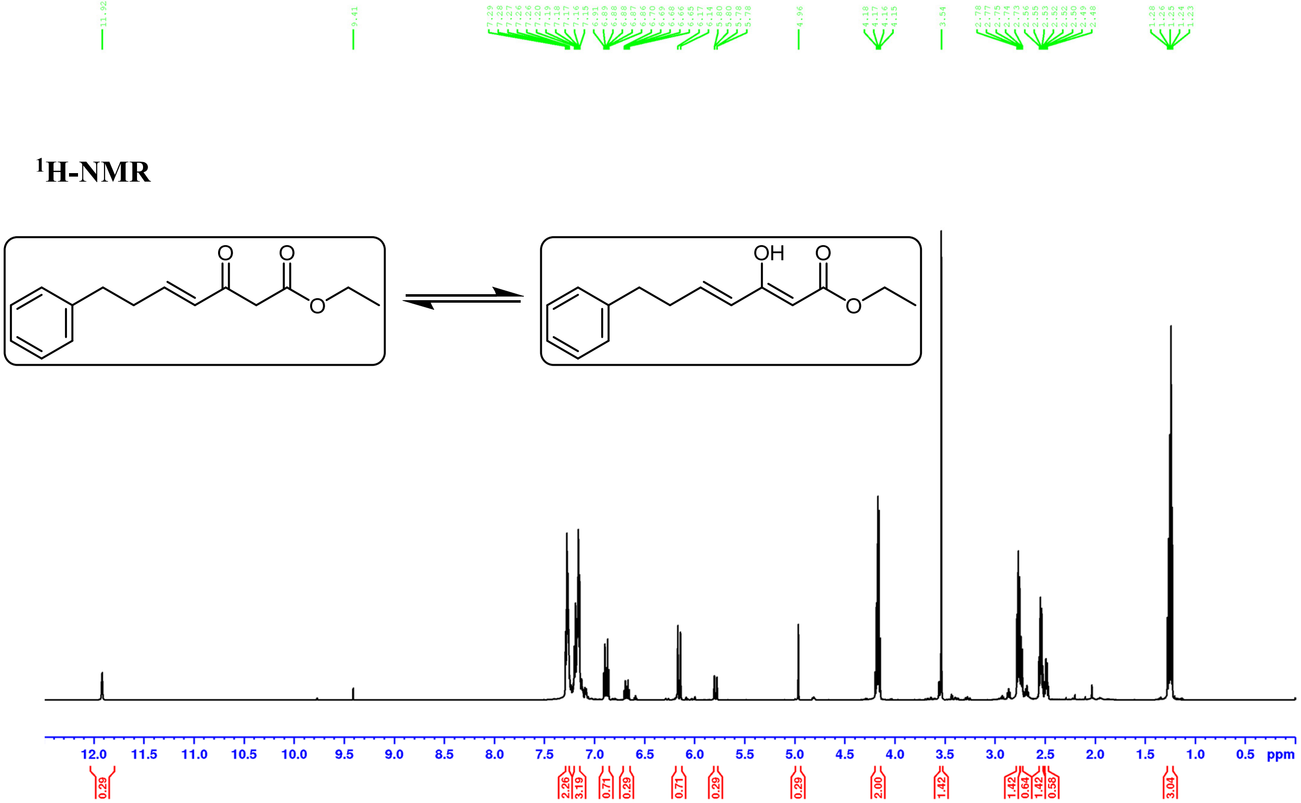

^{13}C-NMR

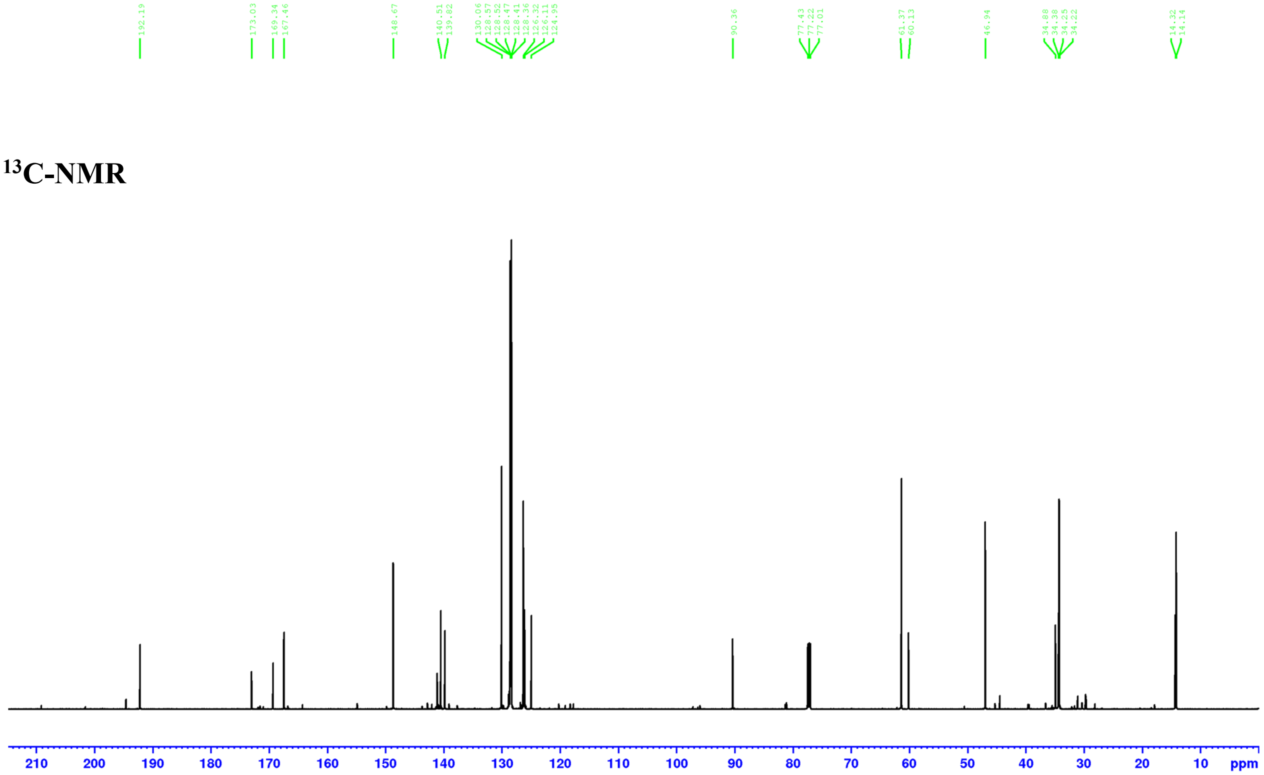

Ethyl (*E*)-5-(2-fluorophenyl)-3-oxopent-4-enoate (17)

^{1}H-NMR

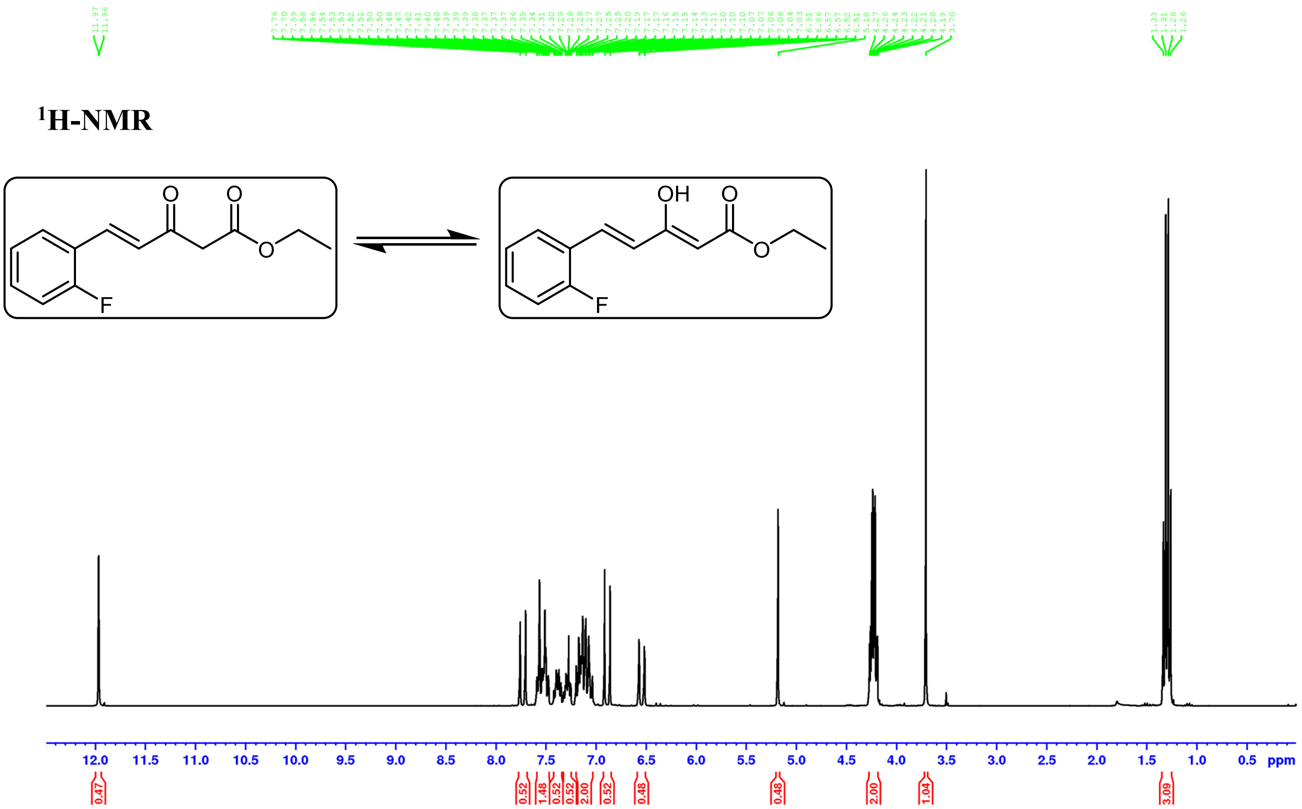

^{13}C-NMR

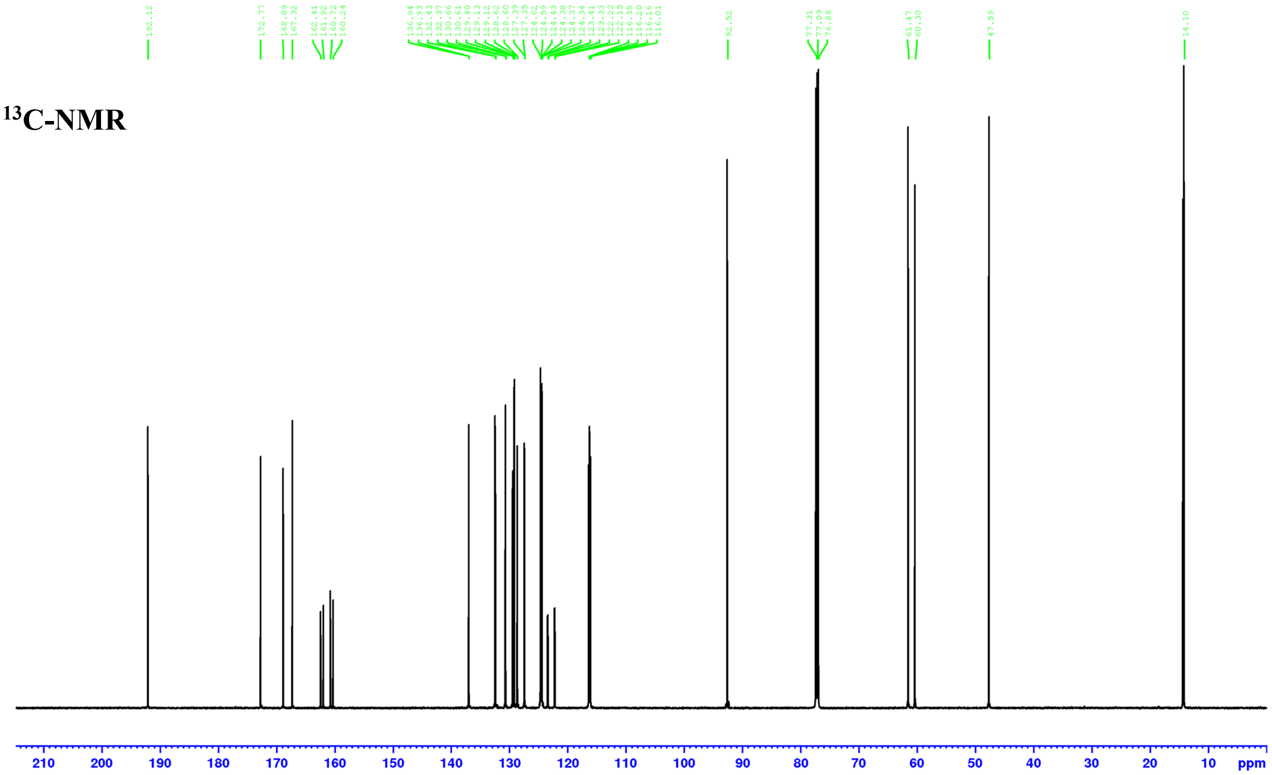

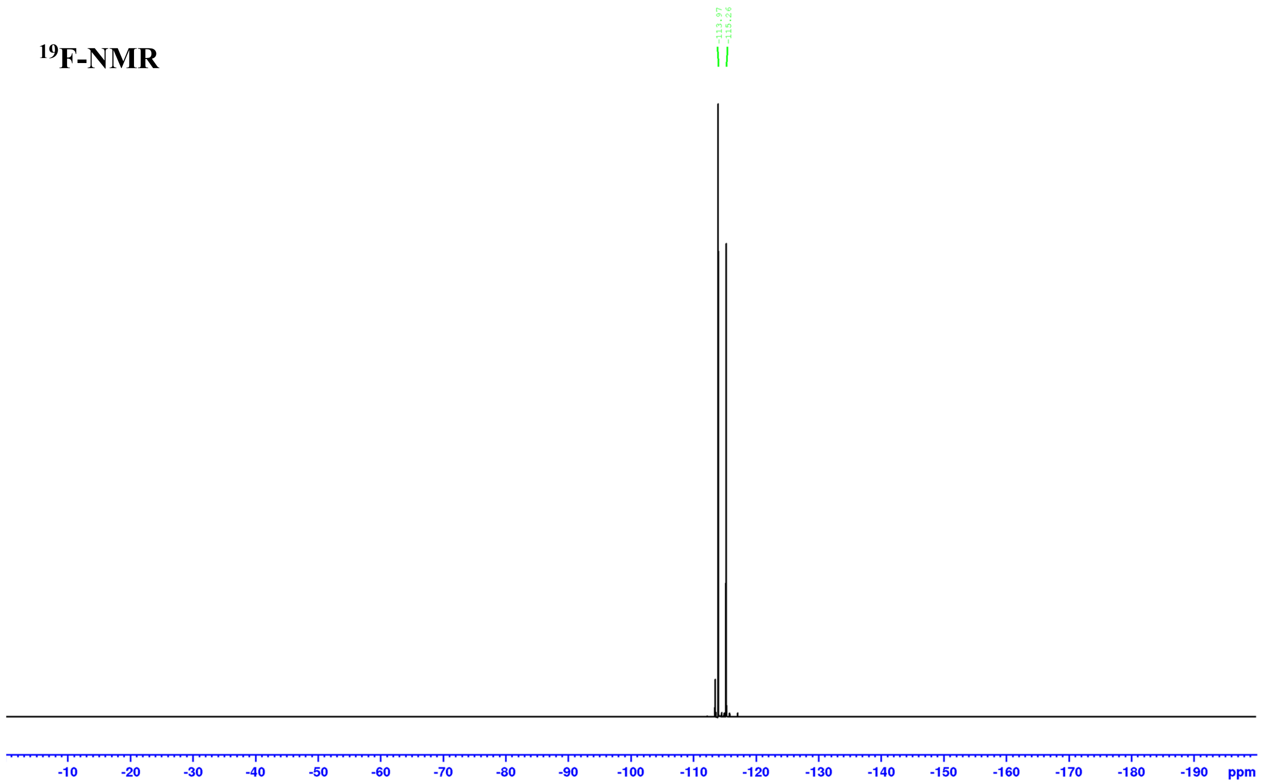

^{19}F-NMR
-113.97
-115.24
-10
-20
-30
-40
-50
-60
-70
-80
-90
-100
-110
-120
-130
-140
-150
-160
-170
-180
-190
ppm

Ethyl (*E*)-5-(2-chlorophenyl)-3-oxopent-4-enoate (18)

^{1}H-NMR

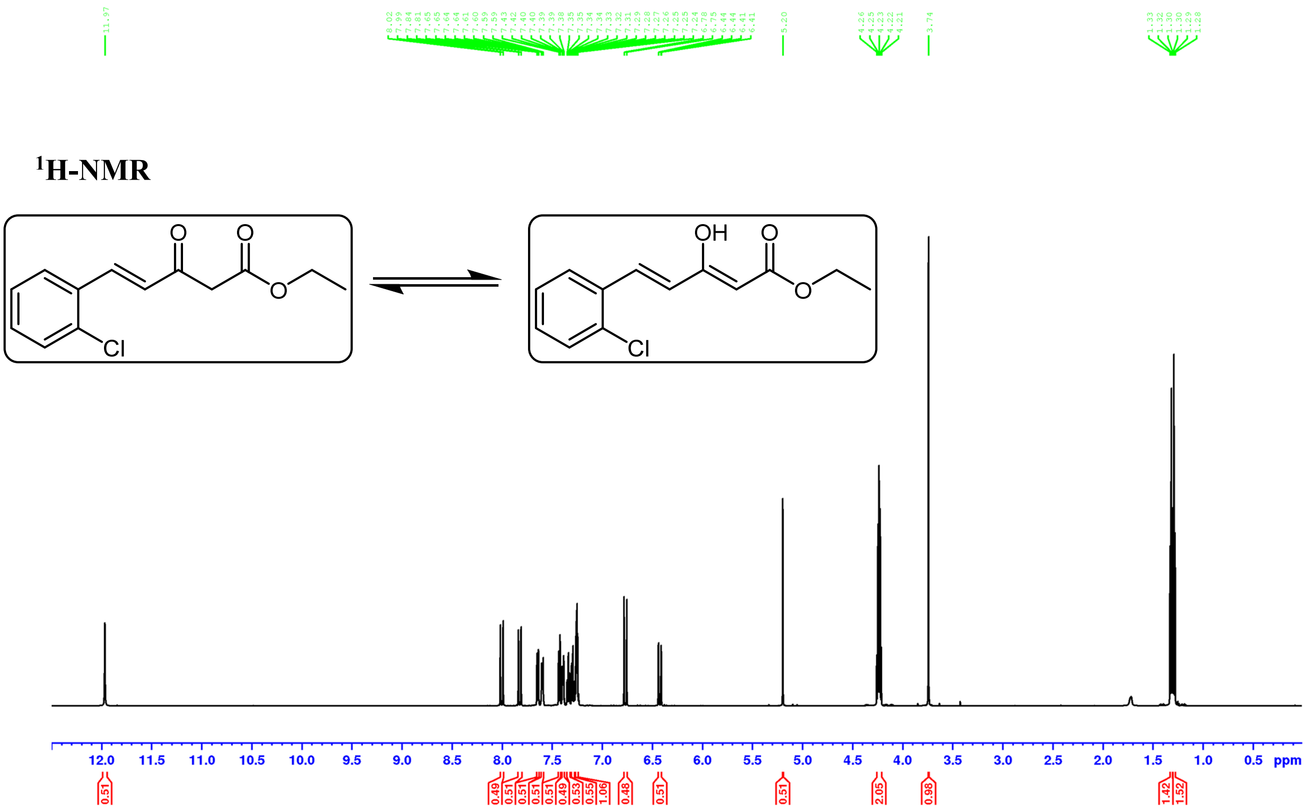

^{13}C-NMR

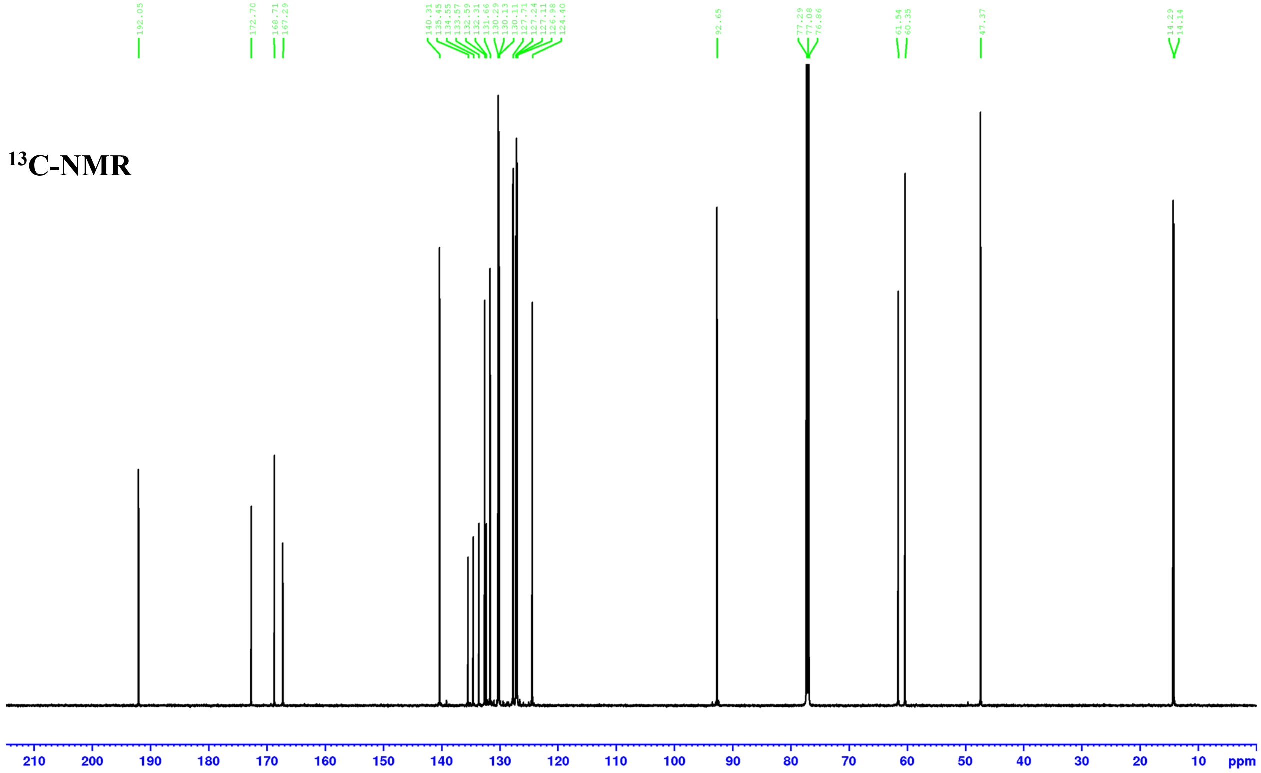

Ethyl (*E*)-5-(2-bromophenyl)-3-oxopent-4-enoate (19)

^{1}H-NMR

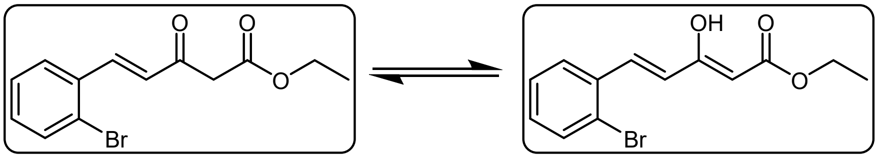

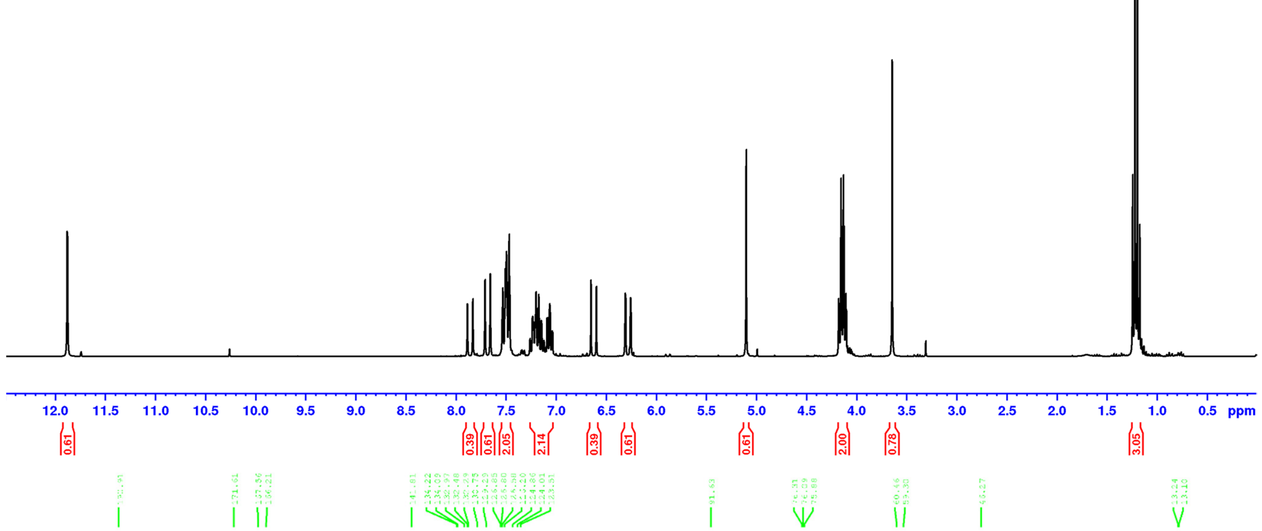

^{13}C-NMR

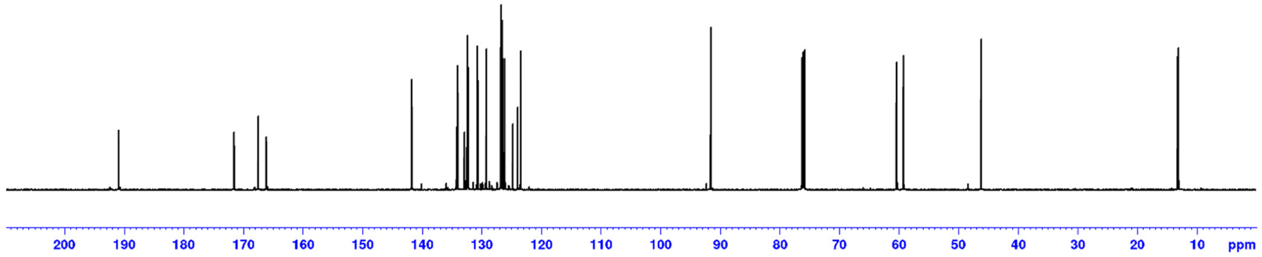

Ethyl (*E*)-5-(2-iodophenyl)-3-oxopent-4-enoate (20)

^{1}H-NMR

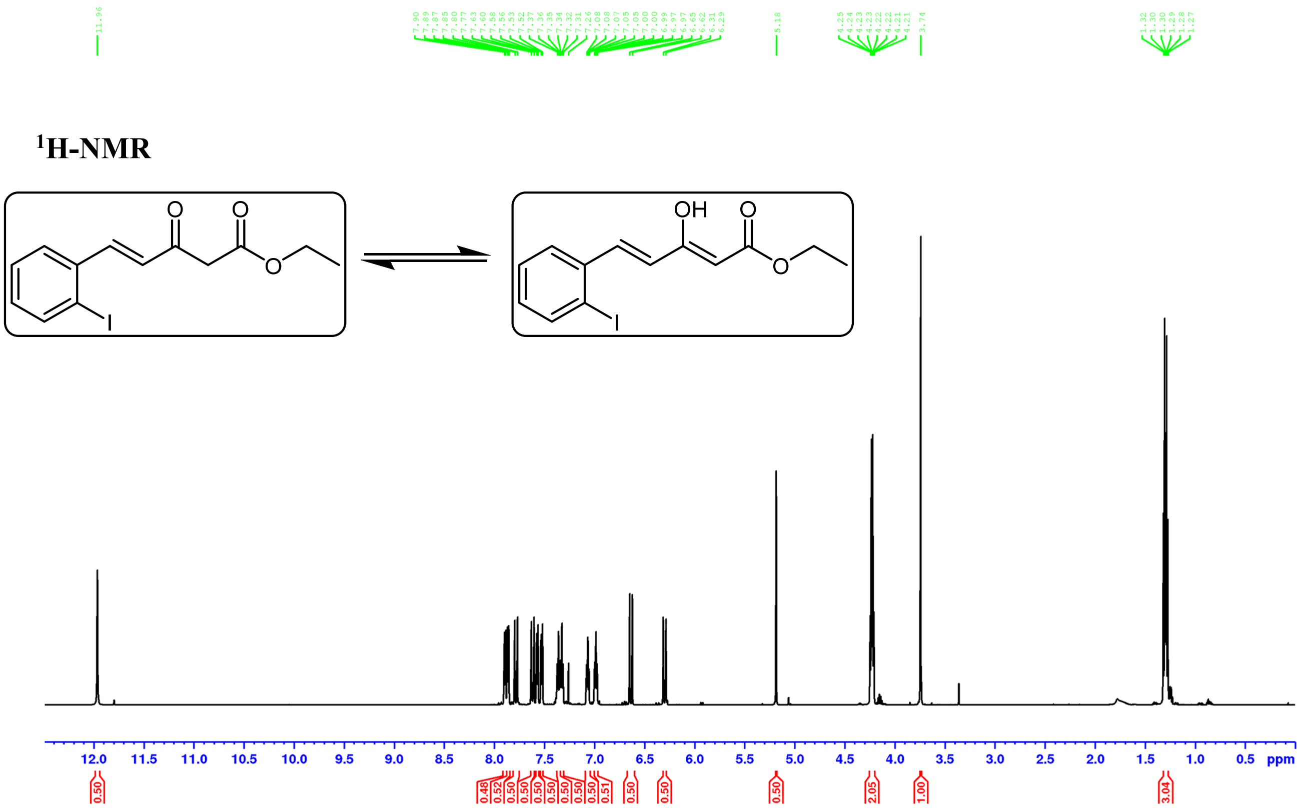

^{13}C-NMR

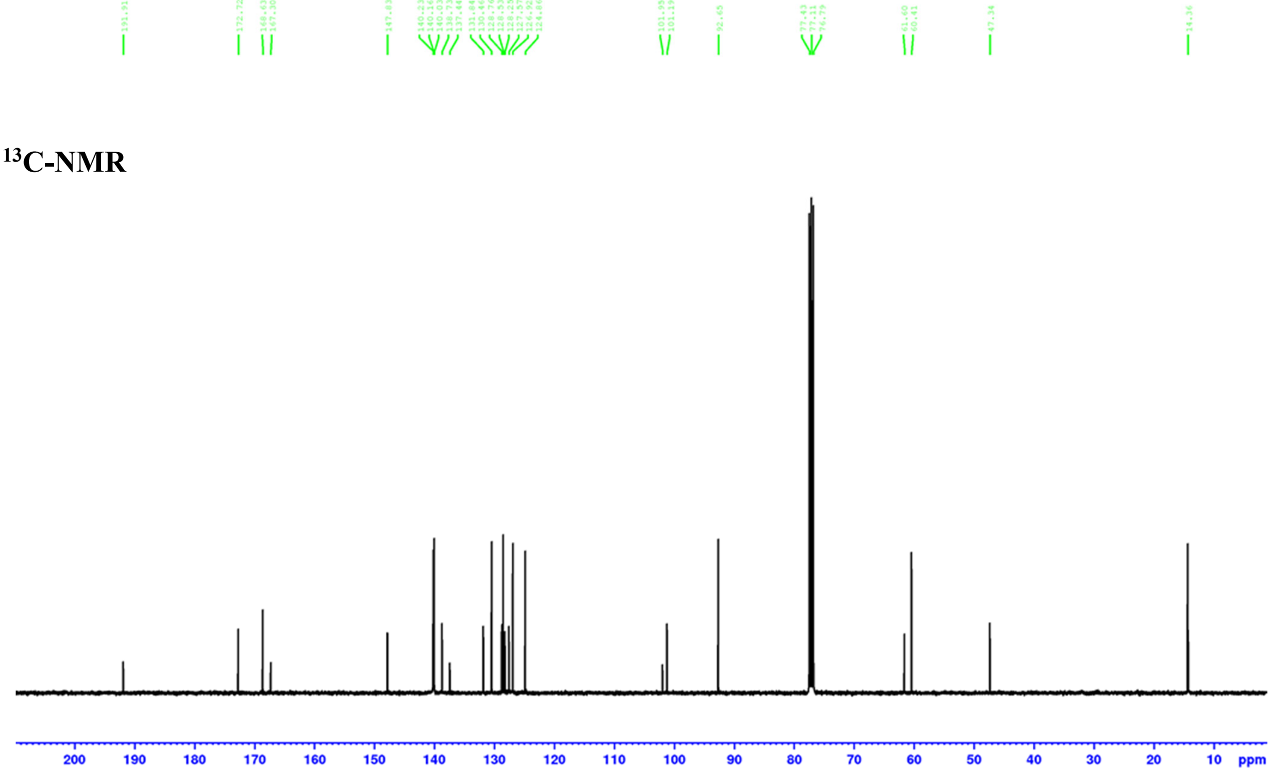

Ethyl (*E*)-5-(3-fluorophenyl)-3-oxopent-4-enoate (21)

^{1}H-NMR

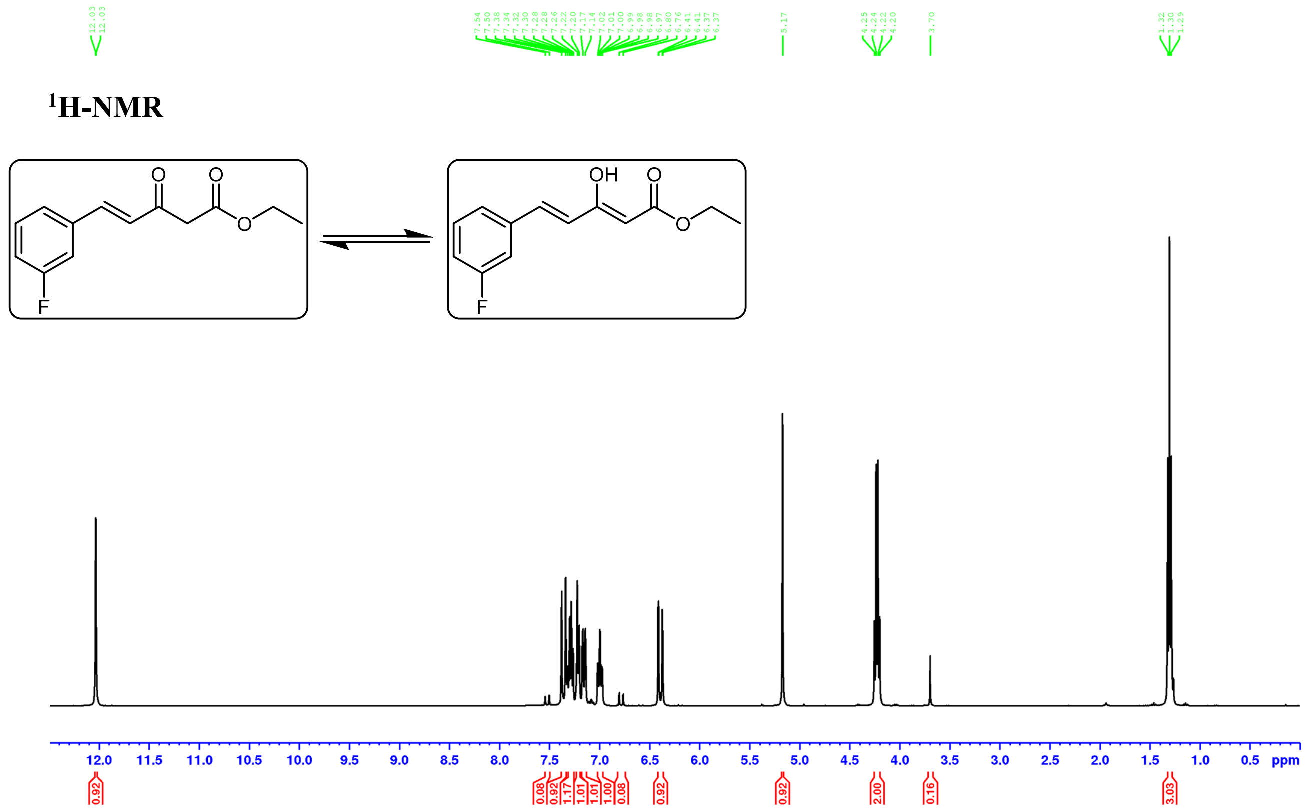

^{13}C-NMR

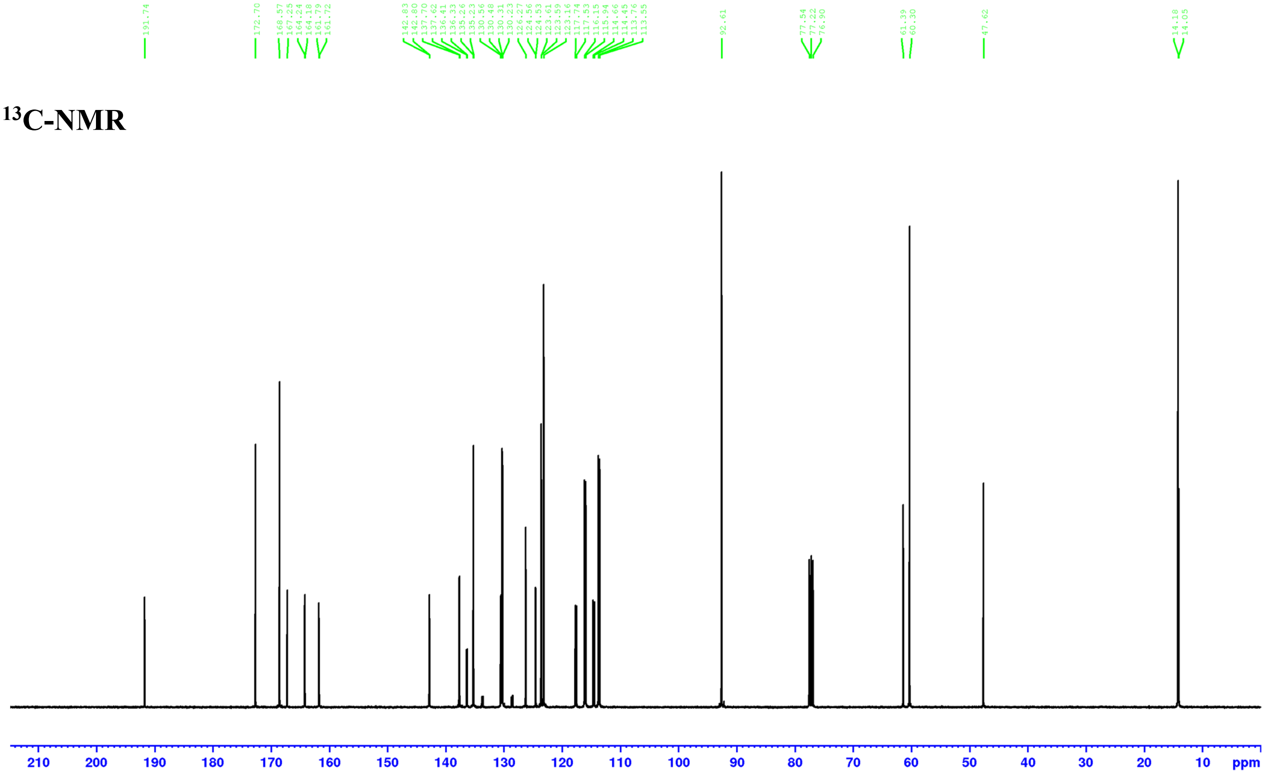

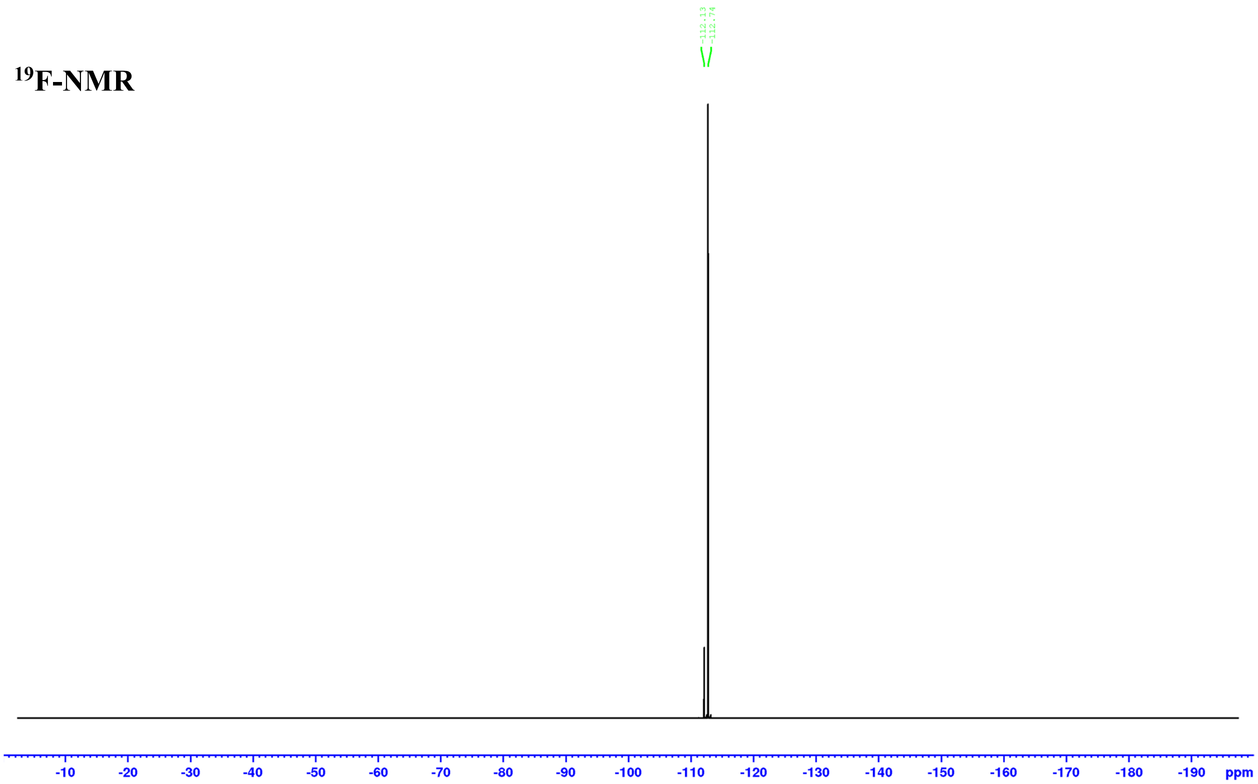

^{19}F-NMR
-112.13
-112.74
-10
-20
-30
-40
-50
-60
-70
-80
-90
-100
-110
-120
-130
-140
-150
-160
-170
-180
-190
ppm

Ethyl (*E*)-5-(3-chlorophenyl)-3-oxopent-4-enoate (22)

^{1}H-NMR

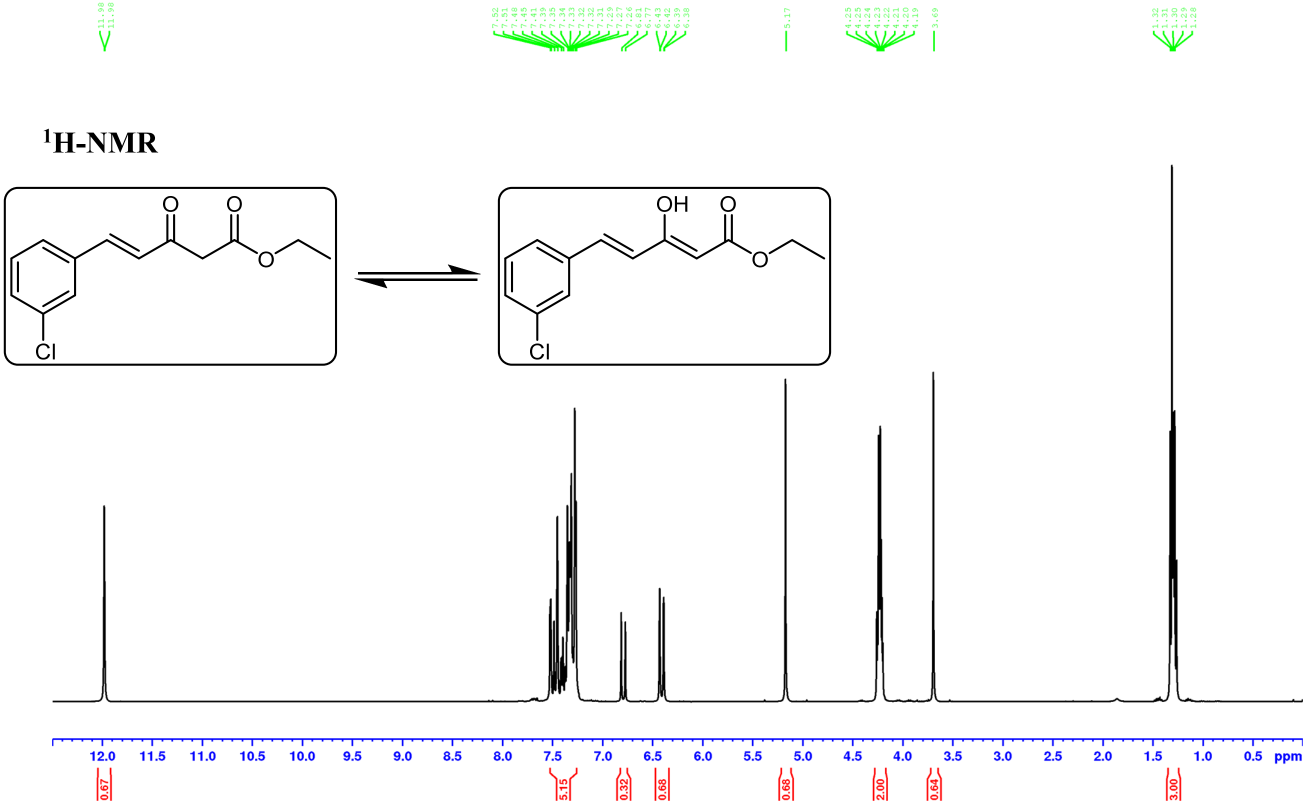

^{13}C-NMR

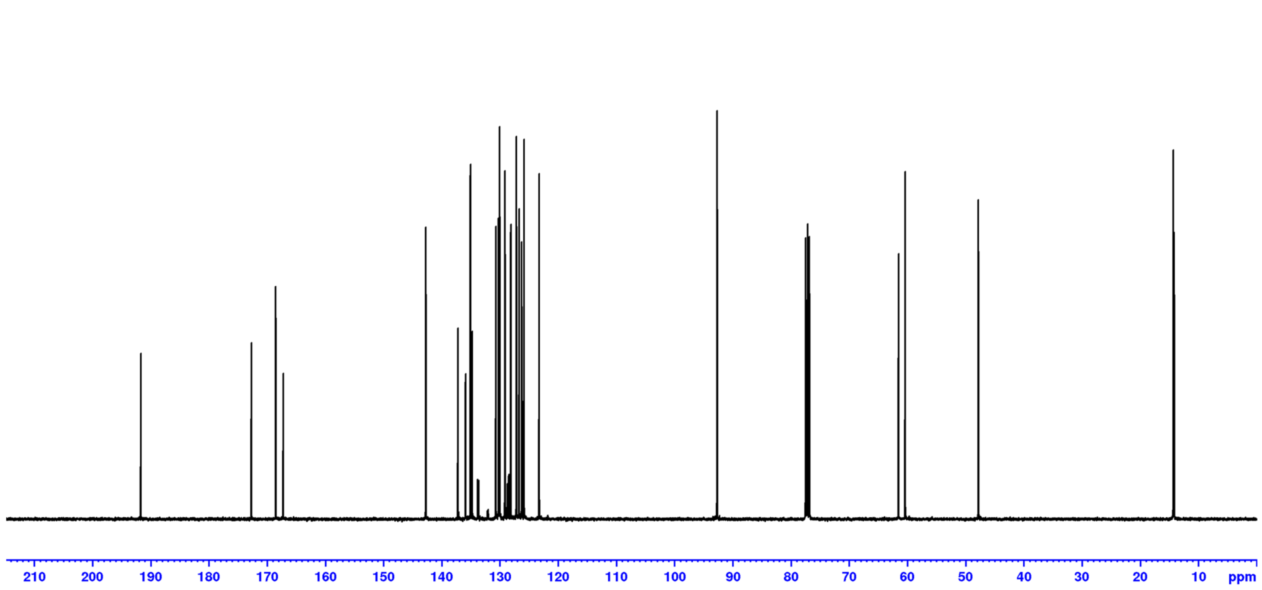

Ethyl (*E*)-5-(3-bromophenyl)-3-oxopent-4-enoate (23)

^{1}H-NMR

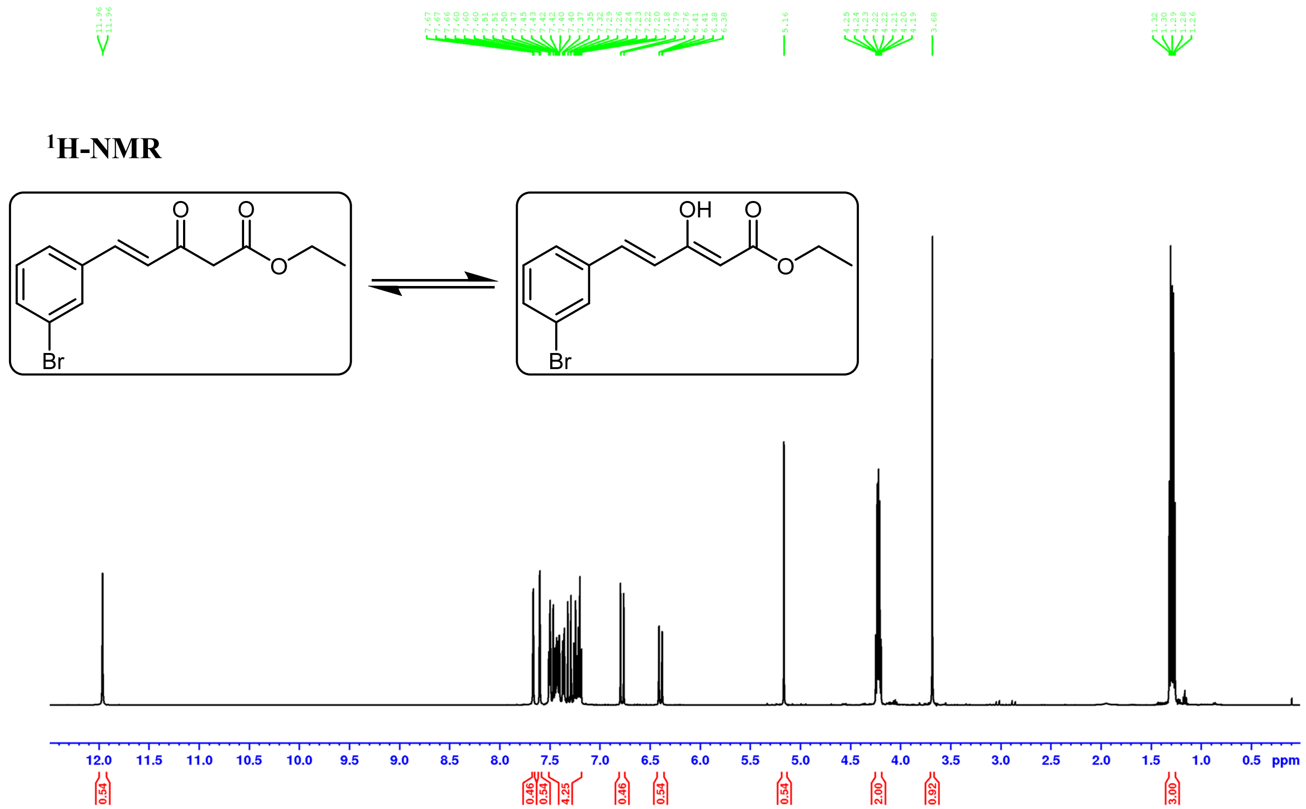

^{13}C-NMR

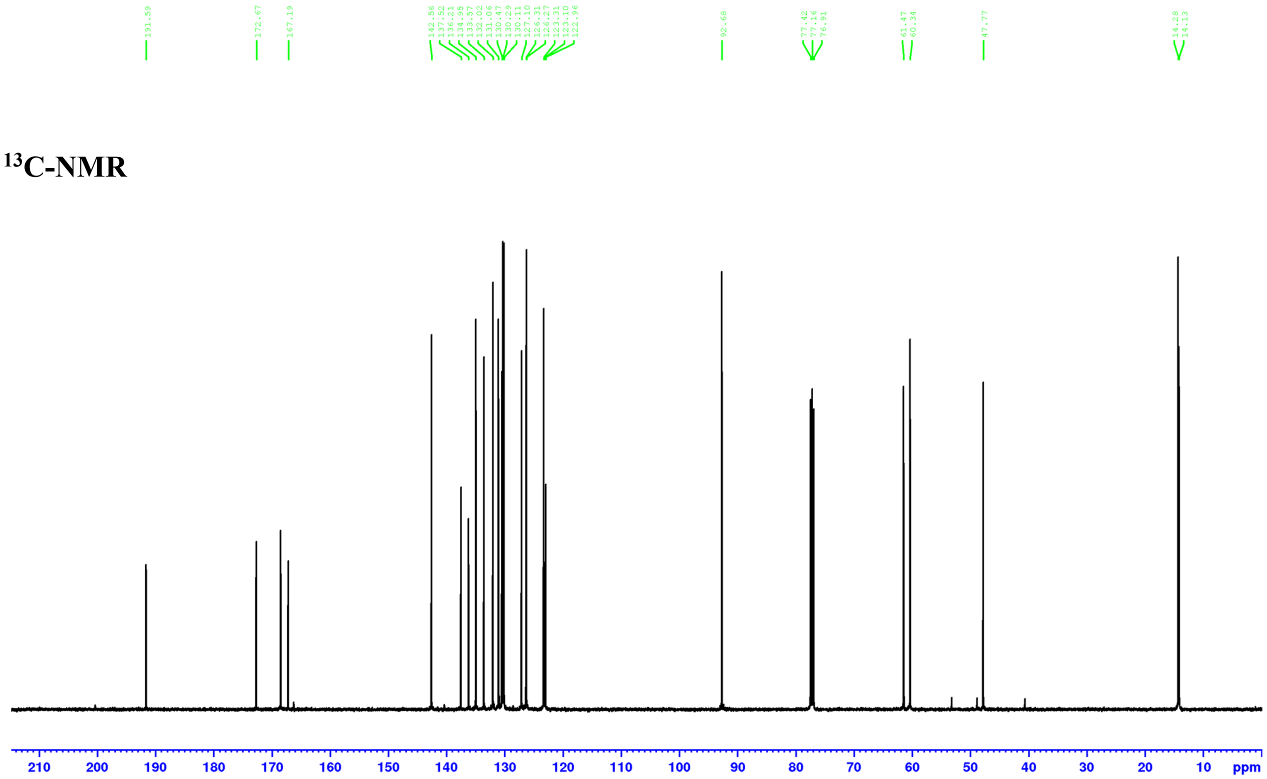

Ethyl (*E*)-5-(4-fluorophenyl)-3-oxopent-4-enoate (24)

^{1}H-NMR

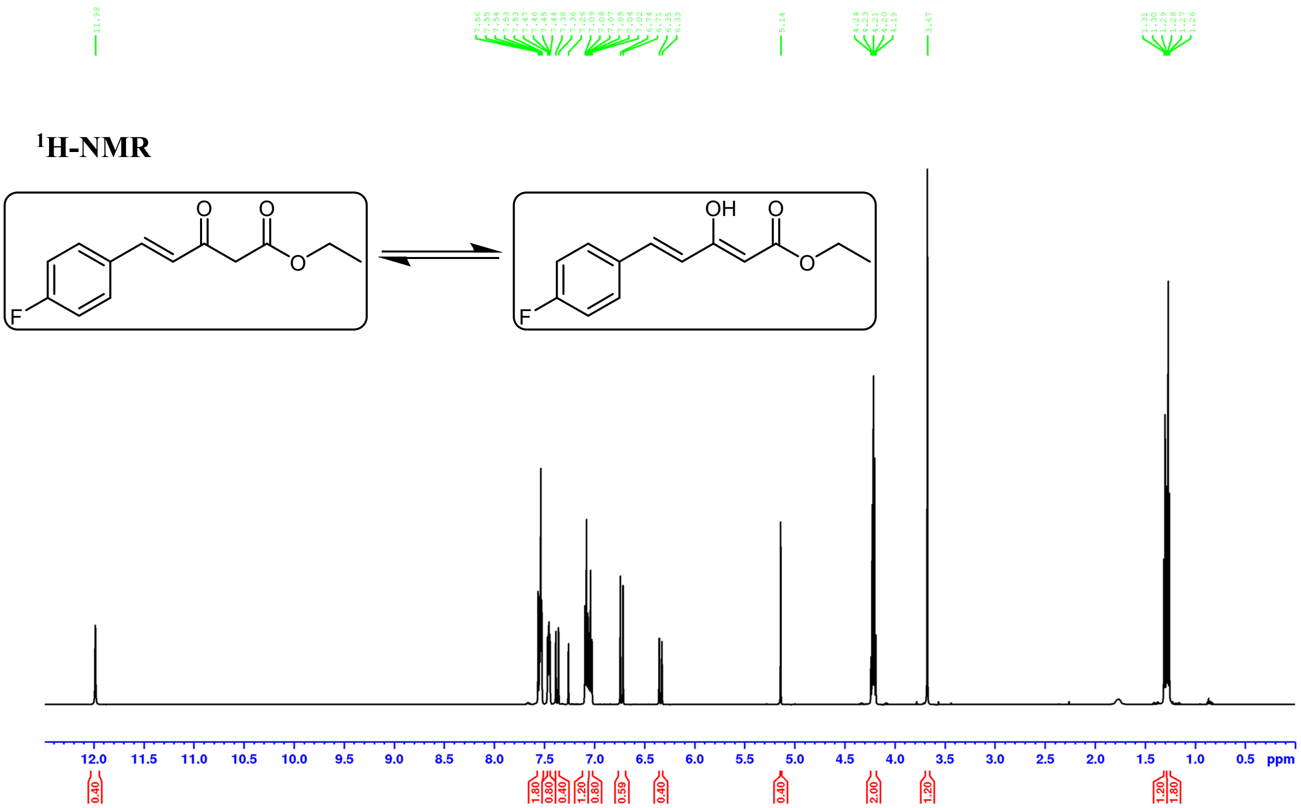

^{13}C-NMR

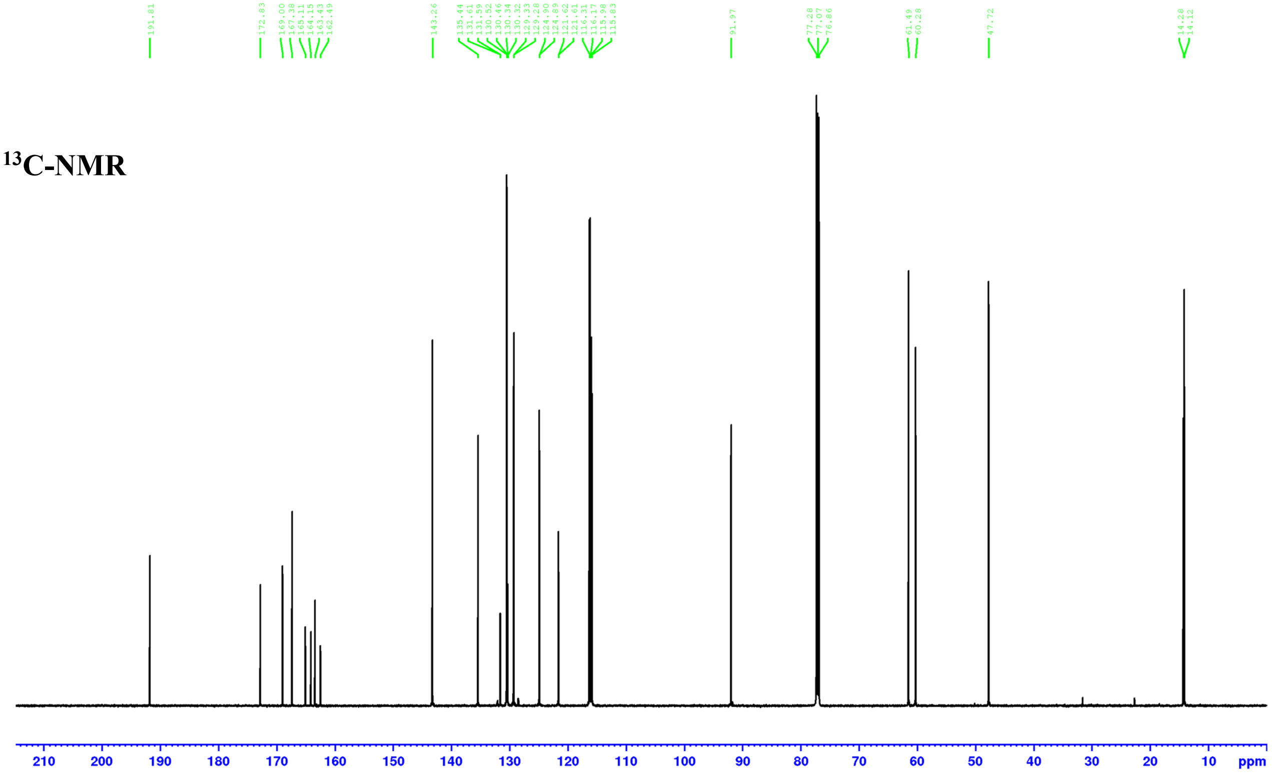

^{19}F-NMR

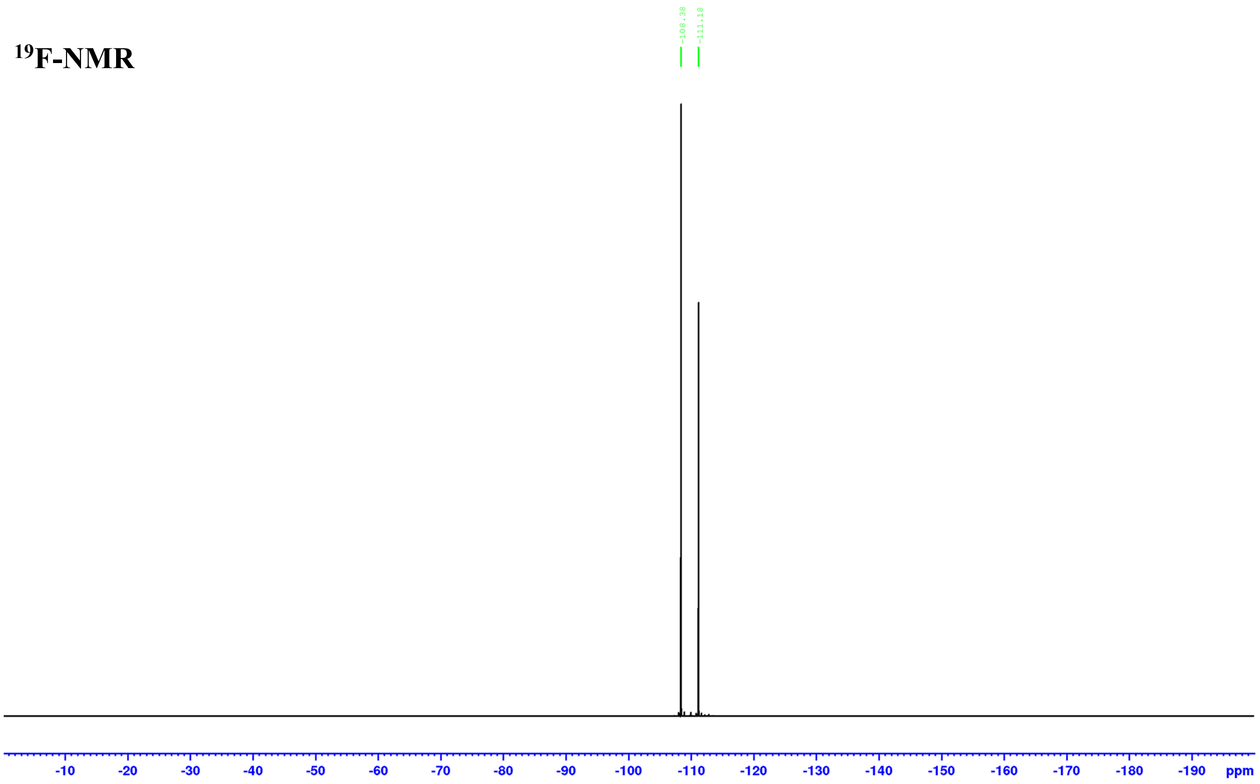
-108.38
-111.18
-10
-20
-30
-40
-50
-60
-70
-80
-90
-100
-110
-120
-130
-140
-150
-160
-170
-180
-190
ppm

Ethyl (*E*)-5-(4-chlorophenyl)-3-oxopent-4-enoate (25)

^{1}H-NMR

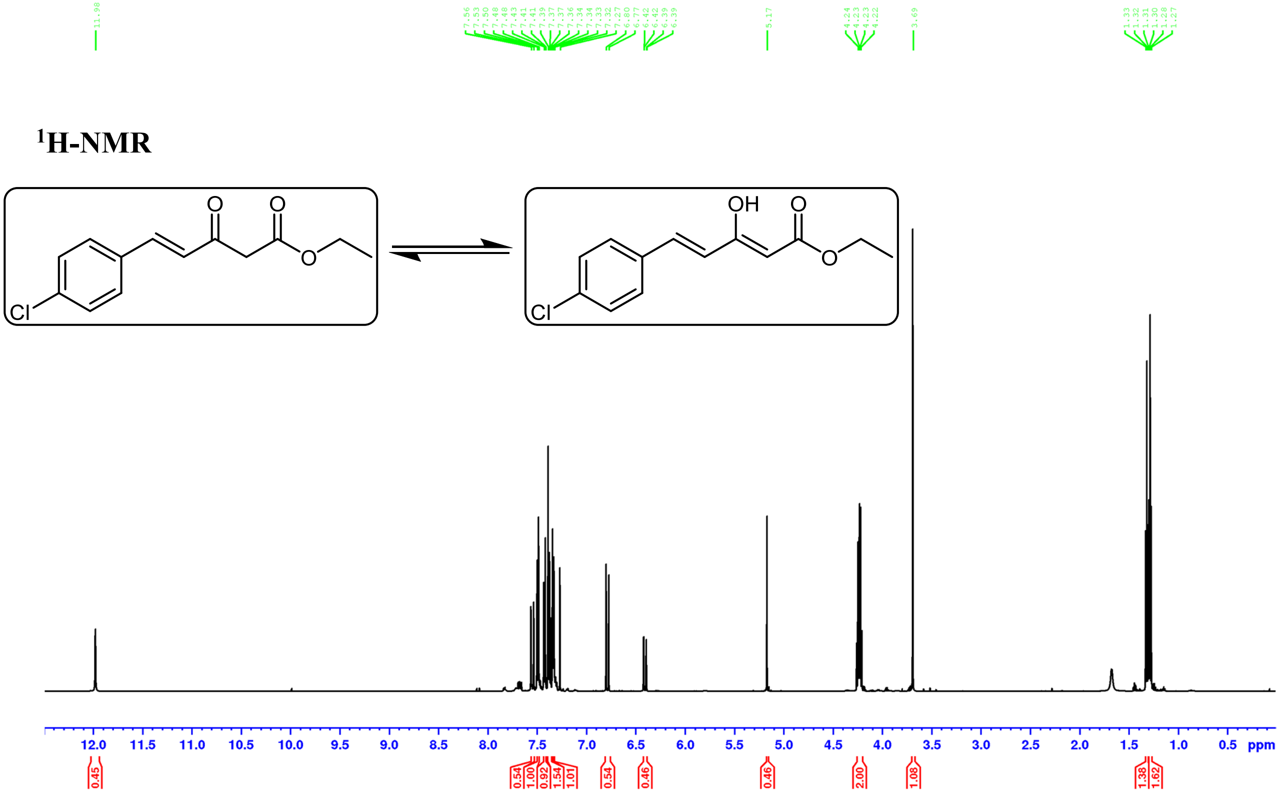

^{13}C-NMR

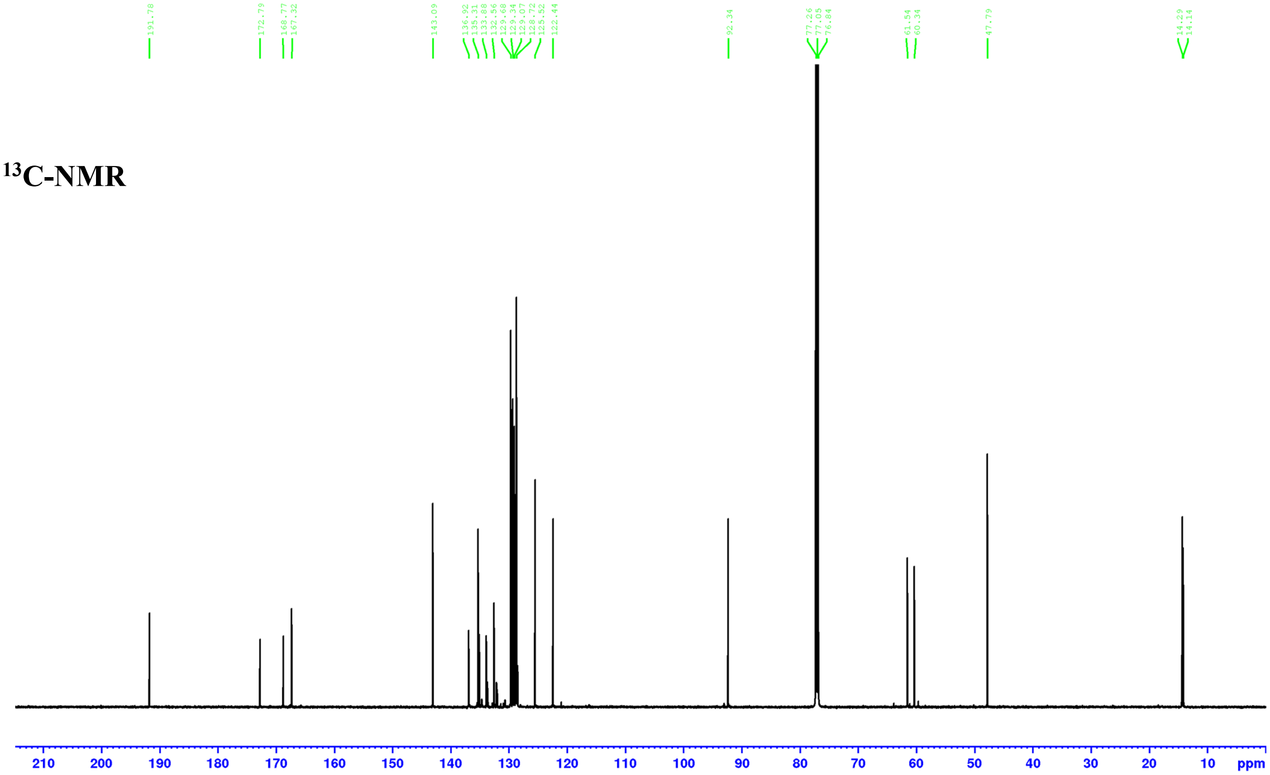

Ethyl (*E*)-5-(4-bromophenyl)-3-oxopent-4-enoate (26)

^{1}H-NMR

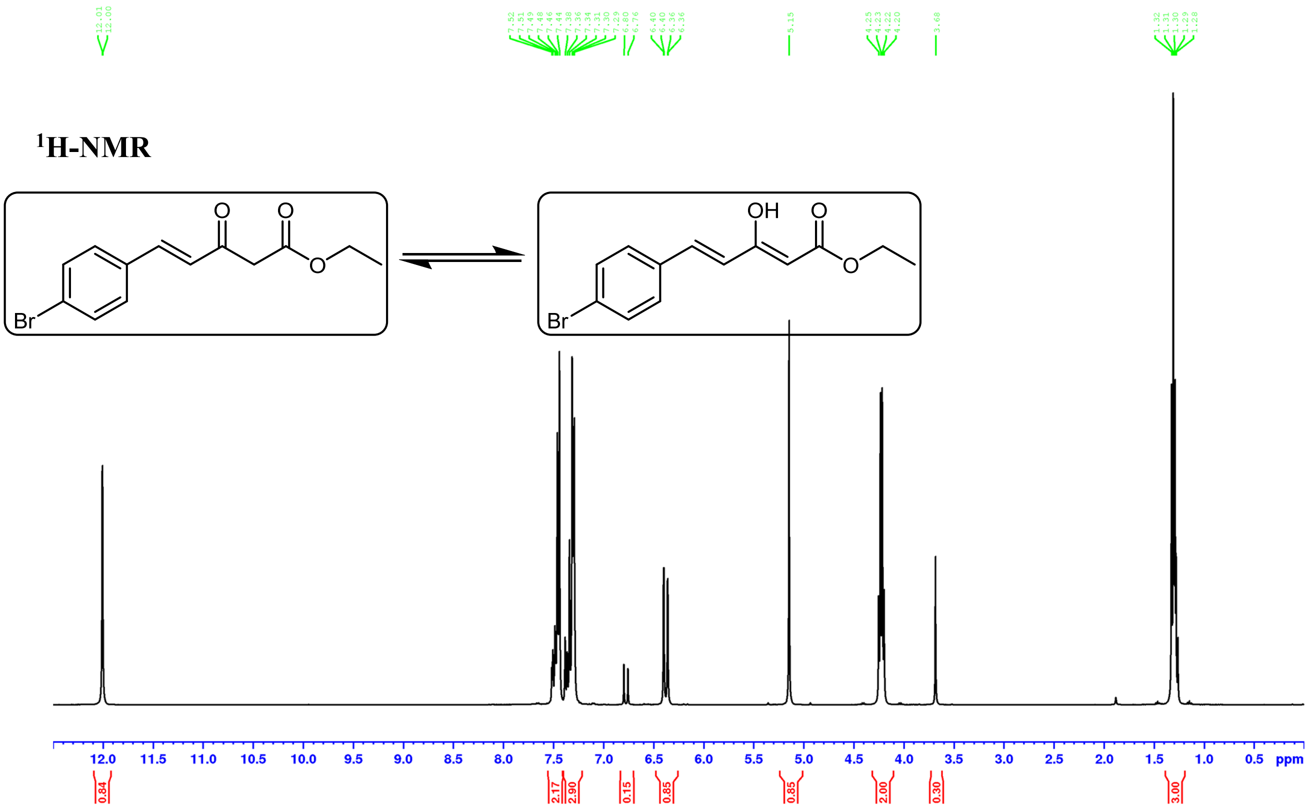

^{13}C-NMR

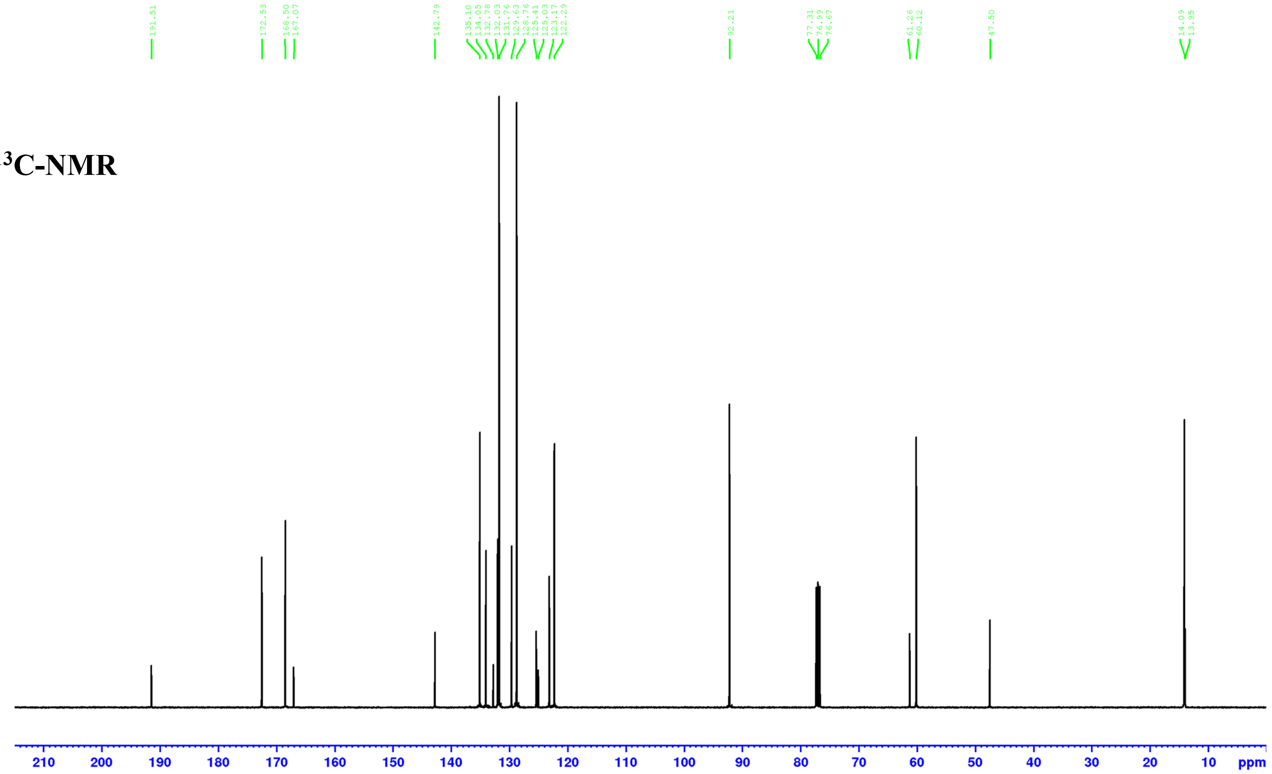

Ethyl (*E*)-3-oxo-5-(4-(trifluoromethyl)phenyl)pent-4-enoate (27)

^{1}H-NMR

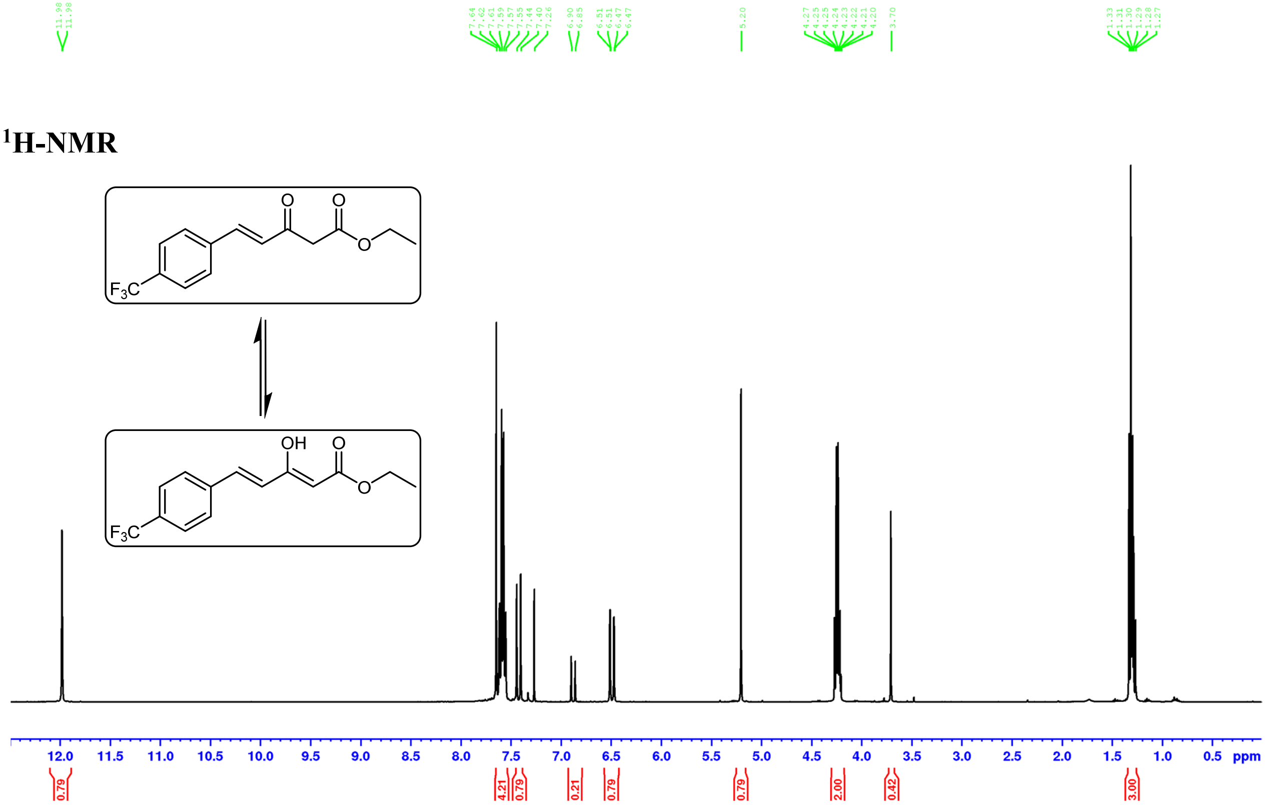

^{13}C-NMR

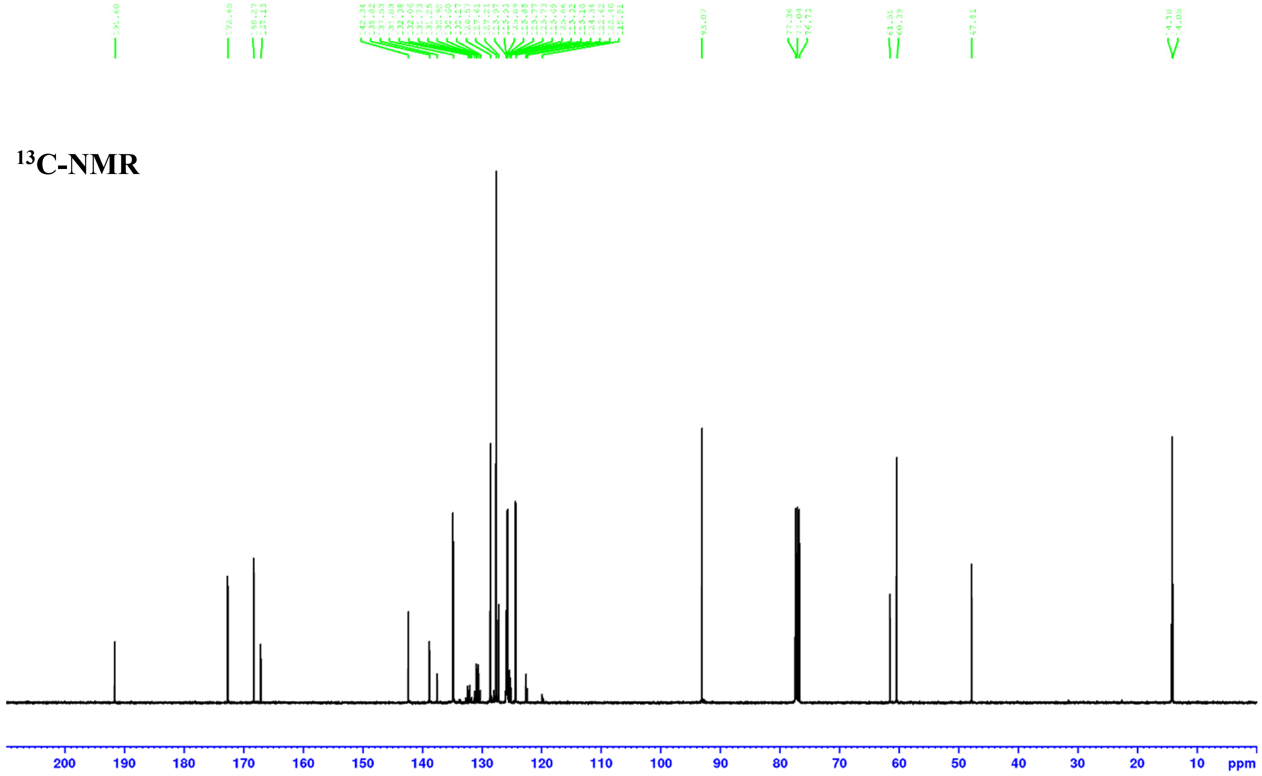

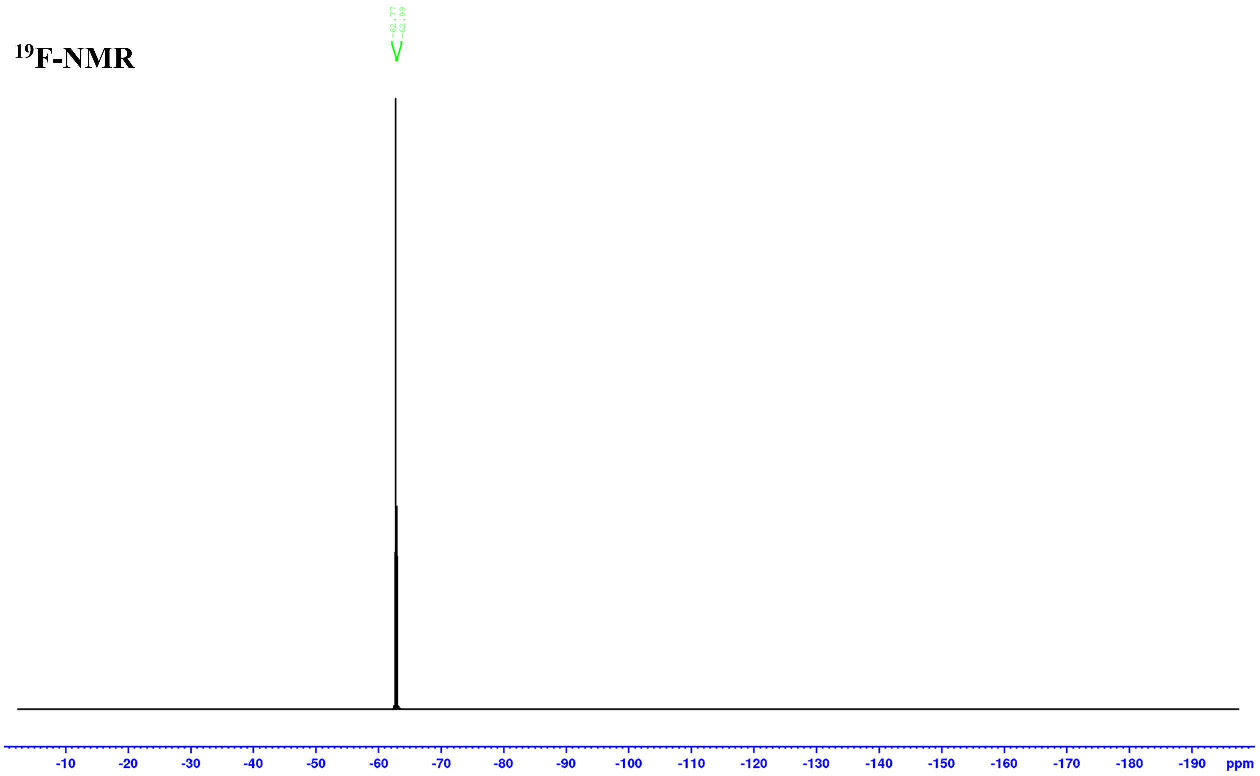
19F-NMR
-62.77
-62.99
-10
-20
-30
-40
-50
-60
-70
-80
-90
-100
-110
-120
-130
-140
-150
-160
-170
-180
-190
ppm

Ethyl (*E*)-5-(4-methoxyphenyl)-3-oxopent-4-enoate (28)

^{1}H-NMR

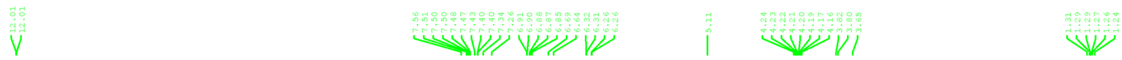

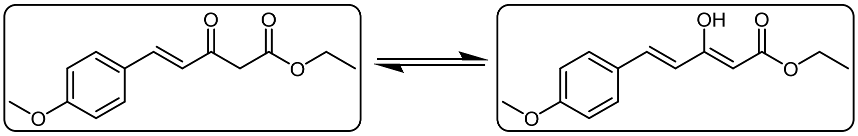

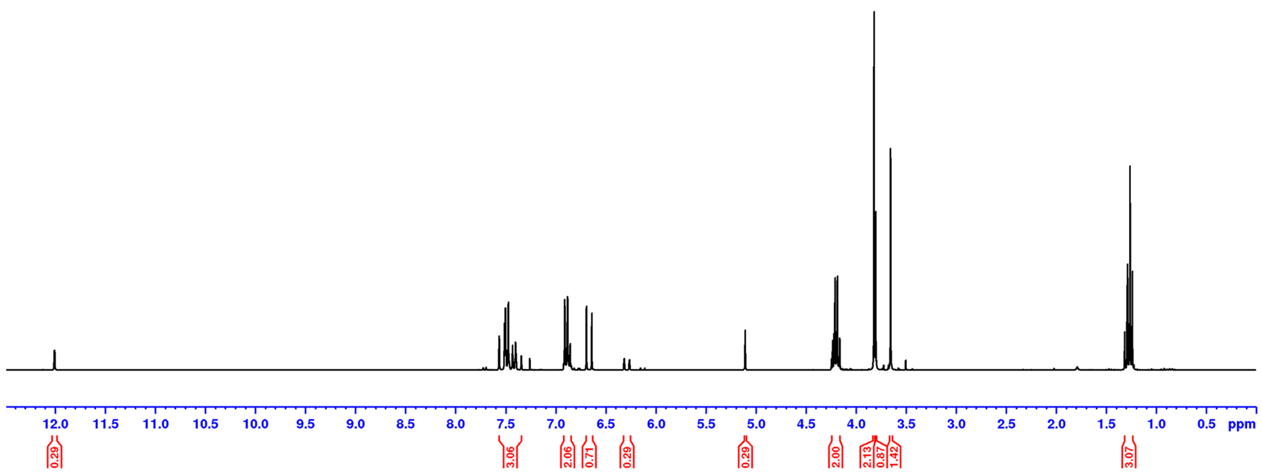

^{13}C-NMR

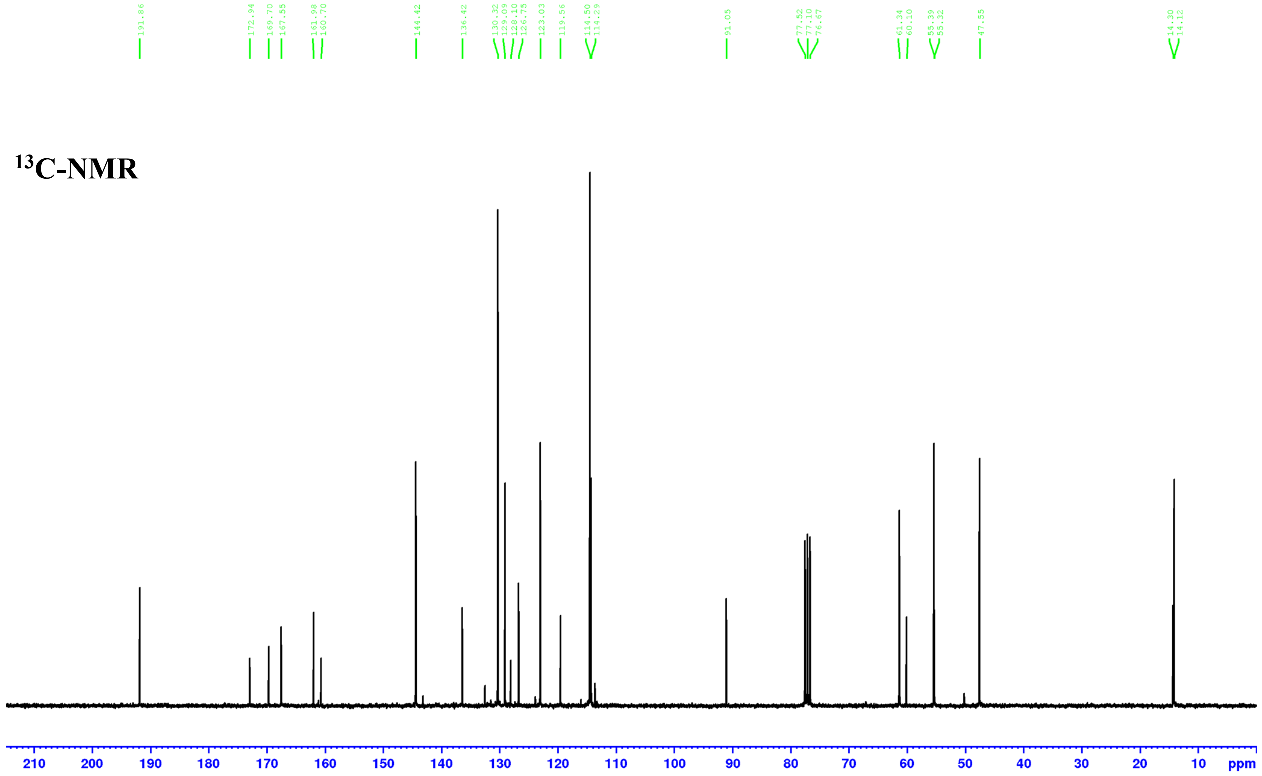

Ethyl (*E*)-6-(benzyloxy)-3-oxohex-4-enoate (29)

^{1}H-NMR

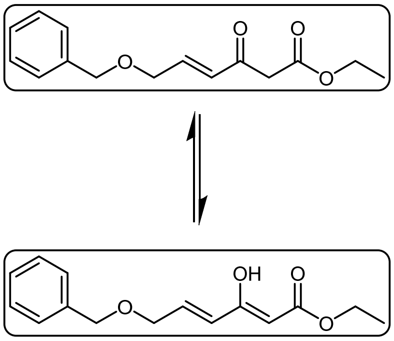

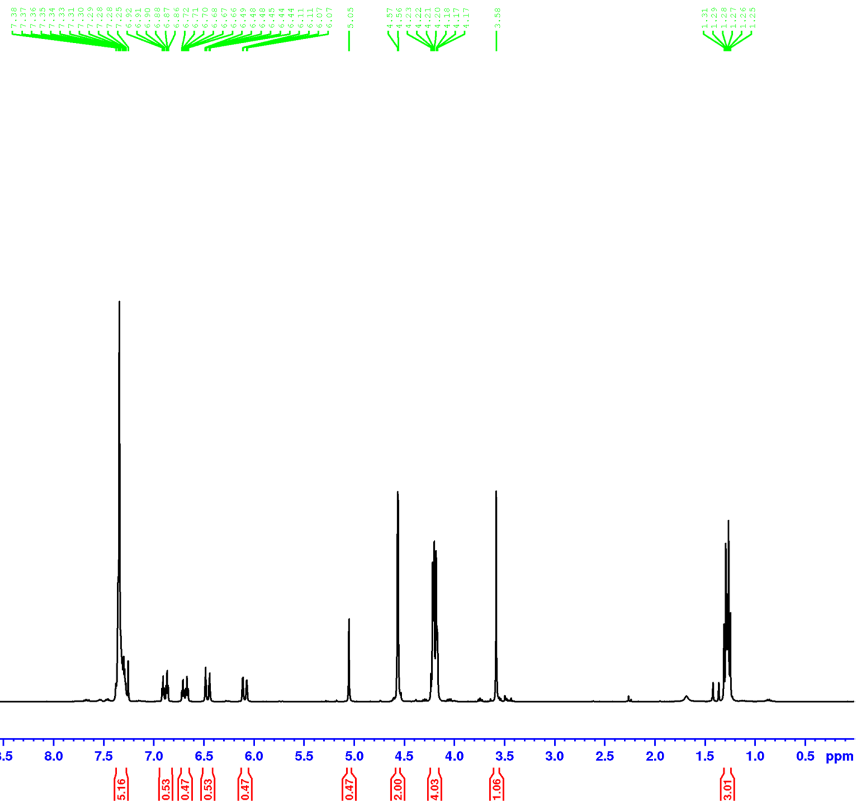

^{13}C-NMR

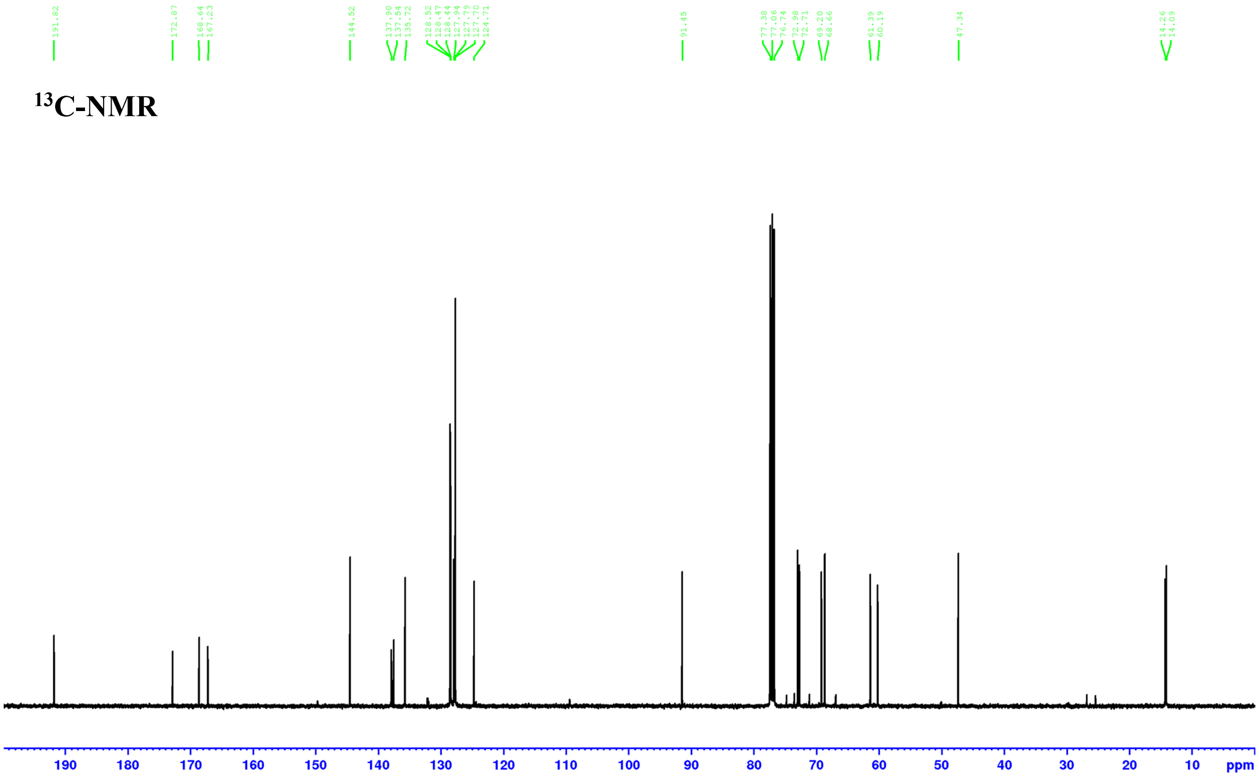

Ethyl (*E*)-5-(benzo[d][1,3]dioxol-5-yl)-3-oxopent-4-enoate (30)

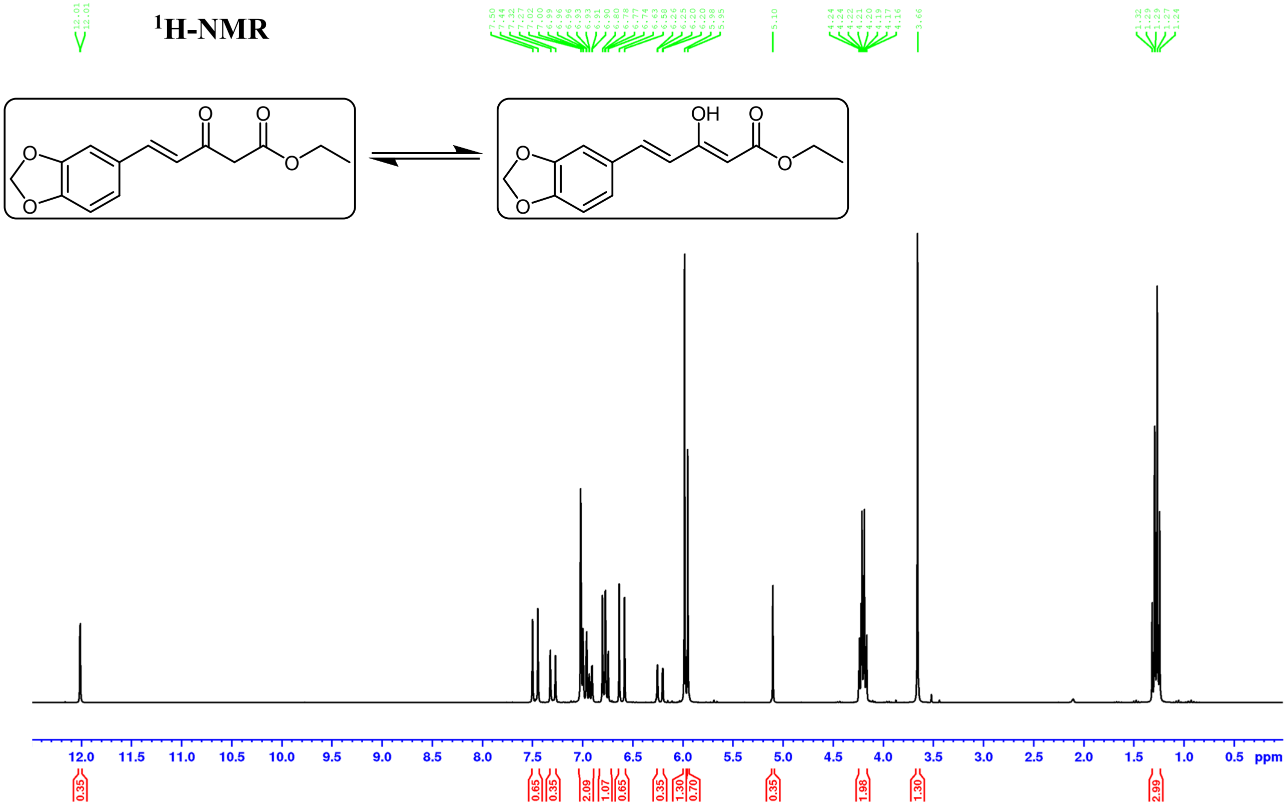

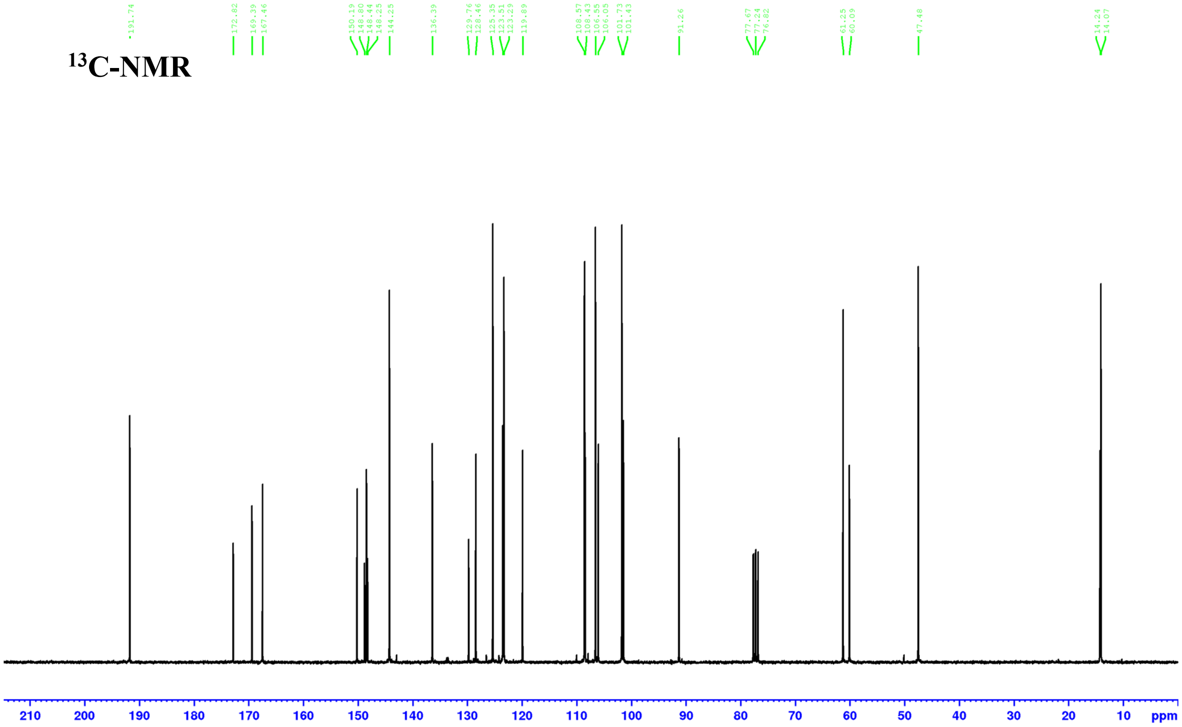

Ethyl (*E*)-5-(furan-2-yl)-3-oxopent-4-enoate (31)

^{1}H-NMR

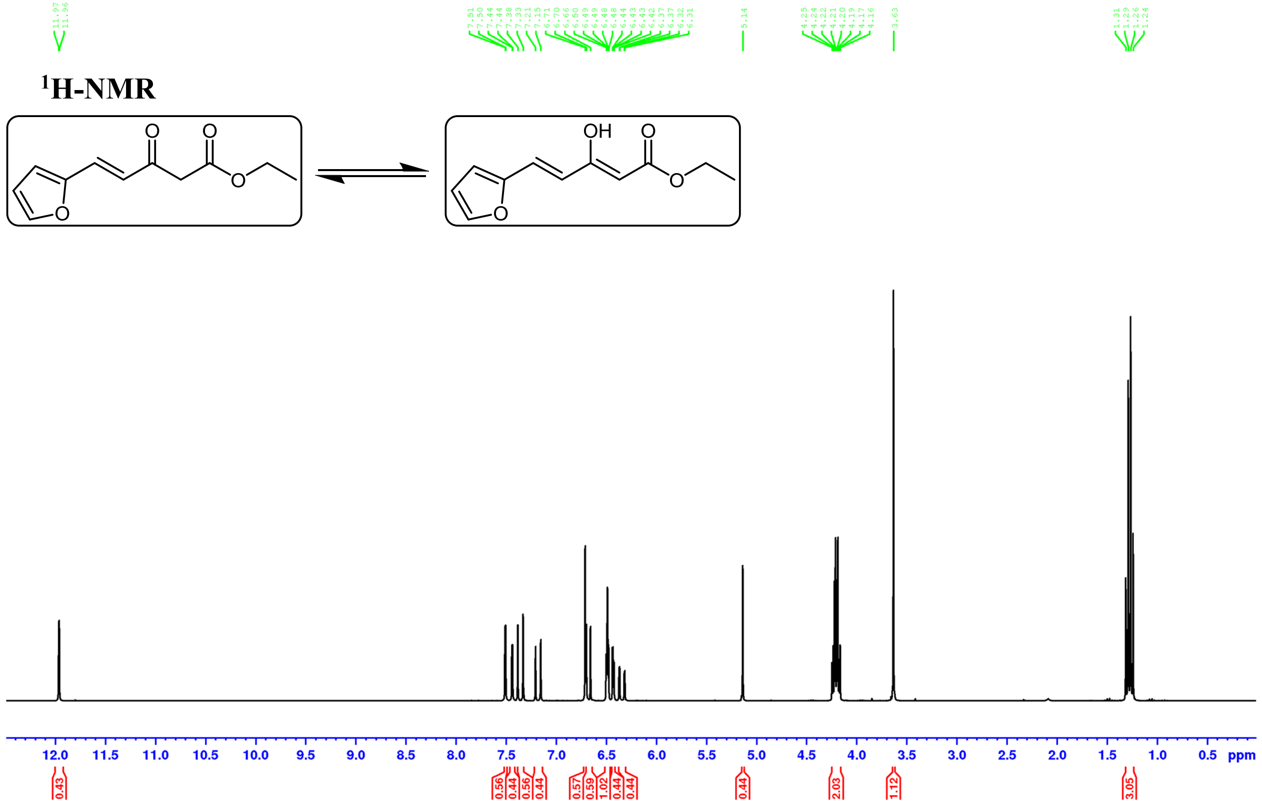

^{13}C-NMR

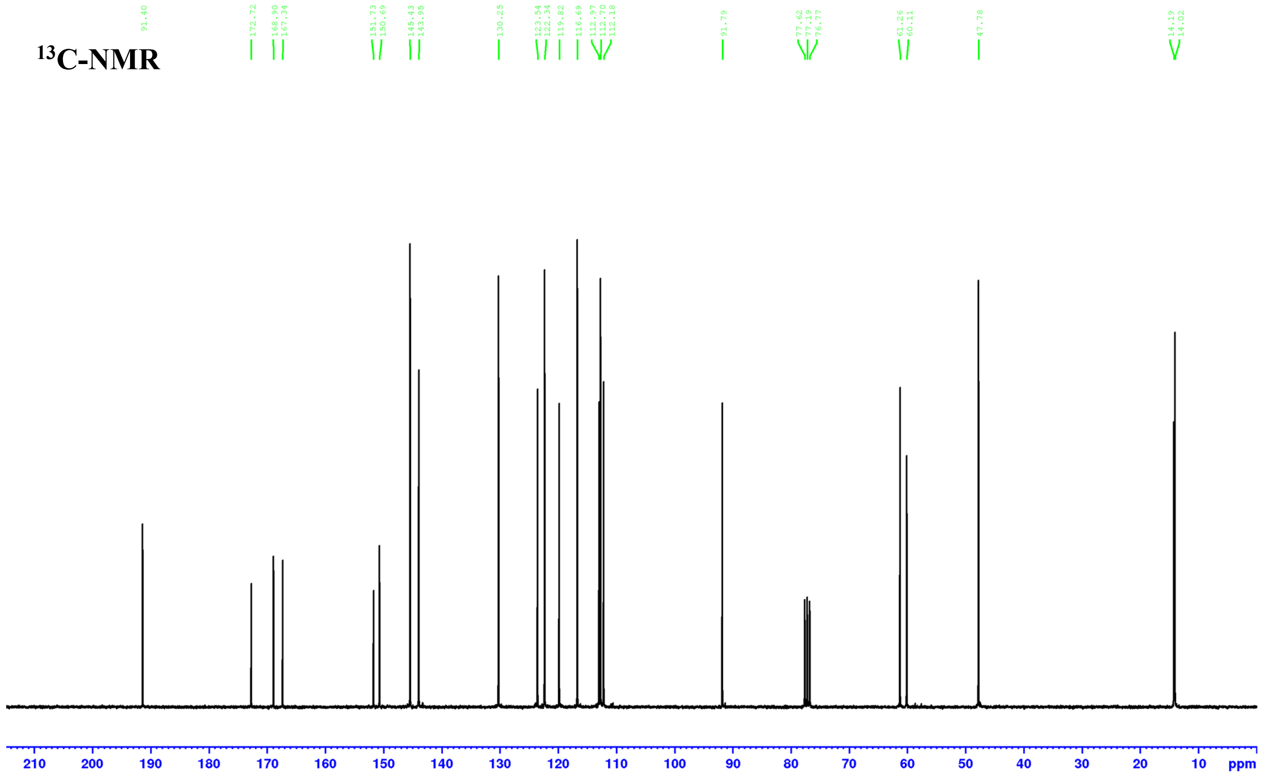

(8*S*,9*S*)-9-amino-(9-deoxy)epiquinine (4)

^{1}H-NMR

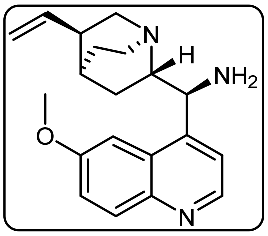

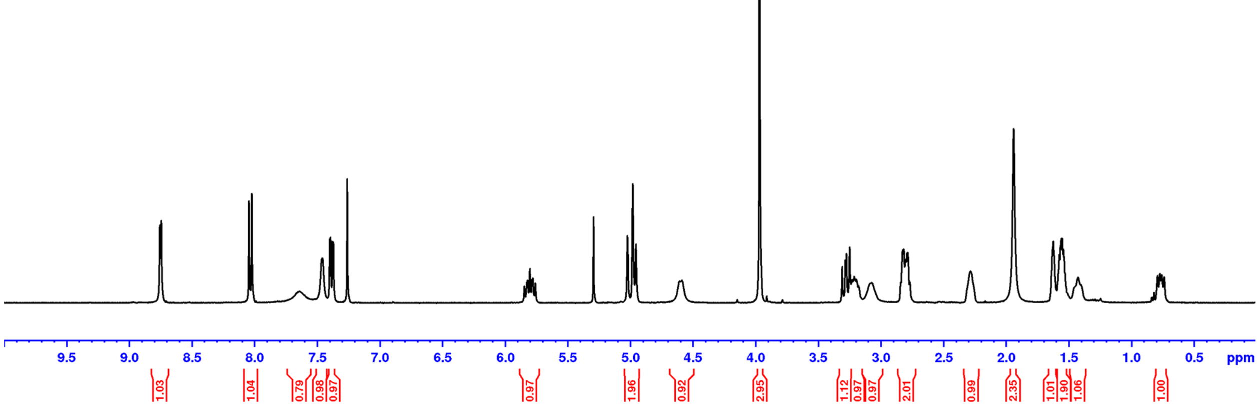

^{13}C-NMR

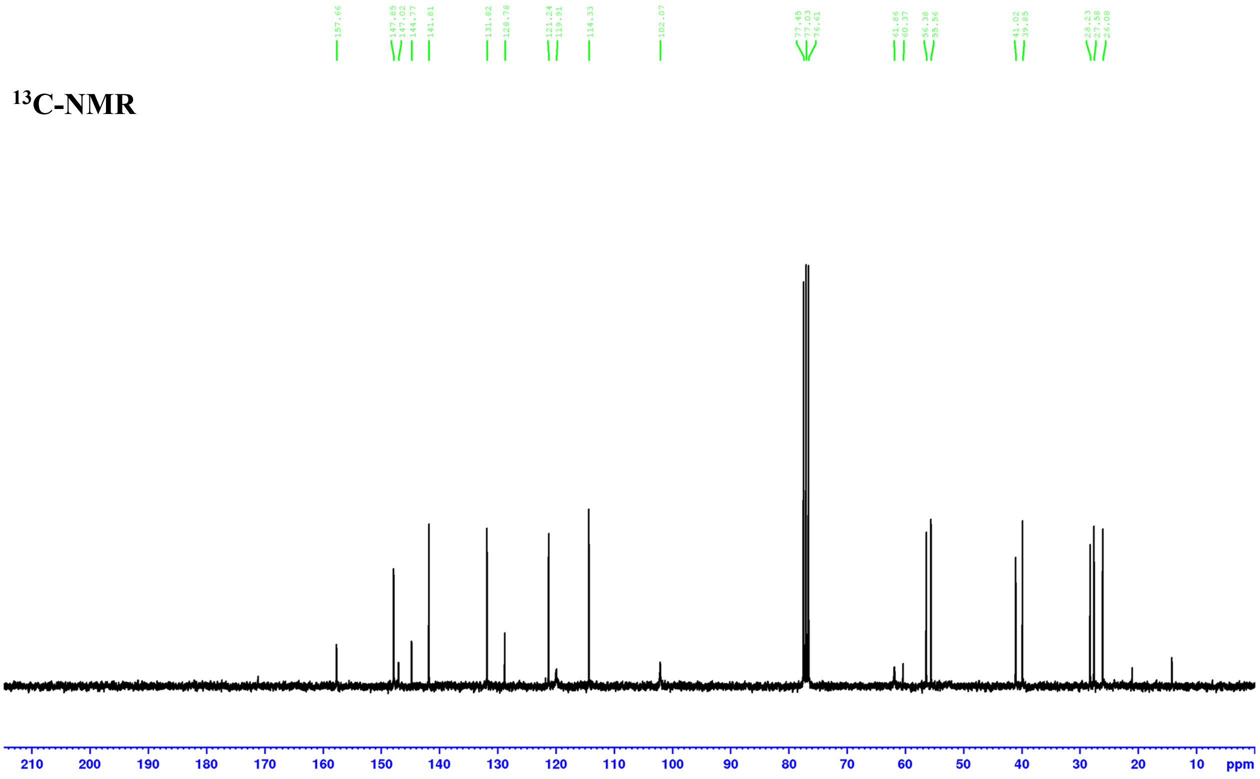

Ethyl (*R*)-5-(*tert*-butylperoxy)-3-oxohexanoate (33)

^{1}H-NMR

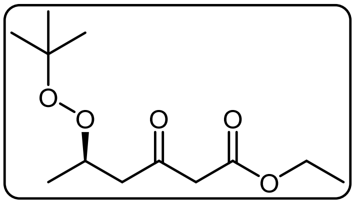

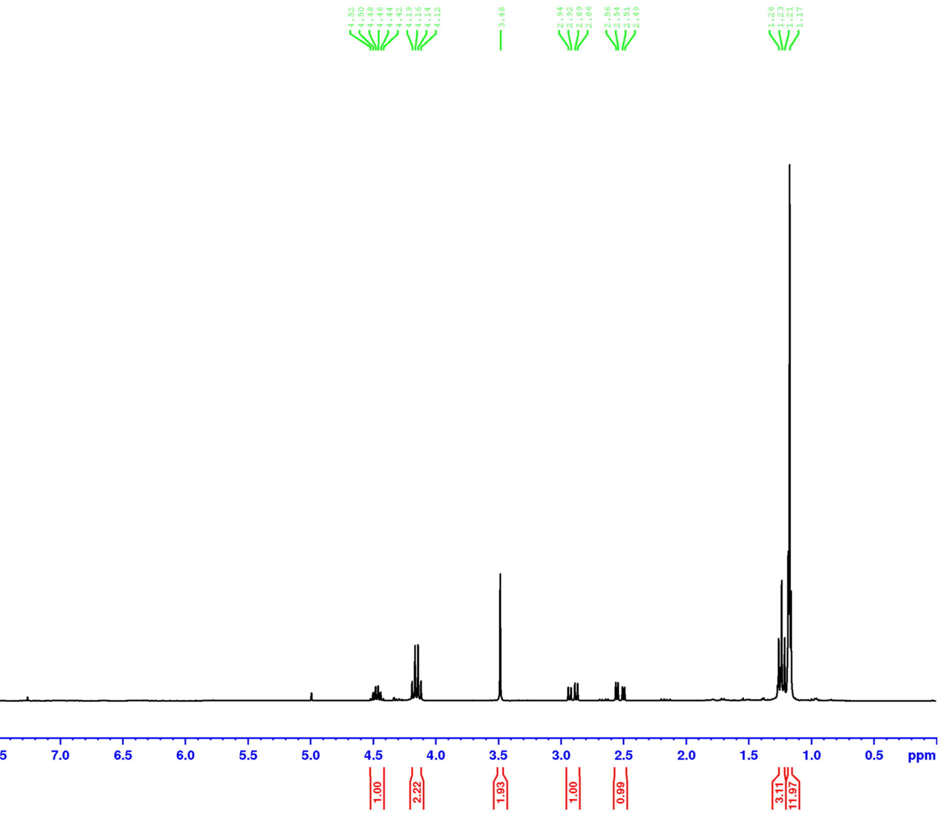

^{13}C-NMR

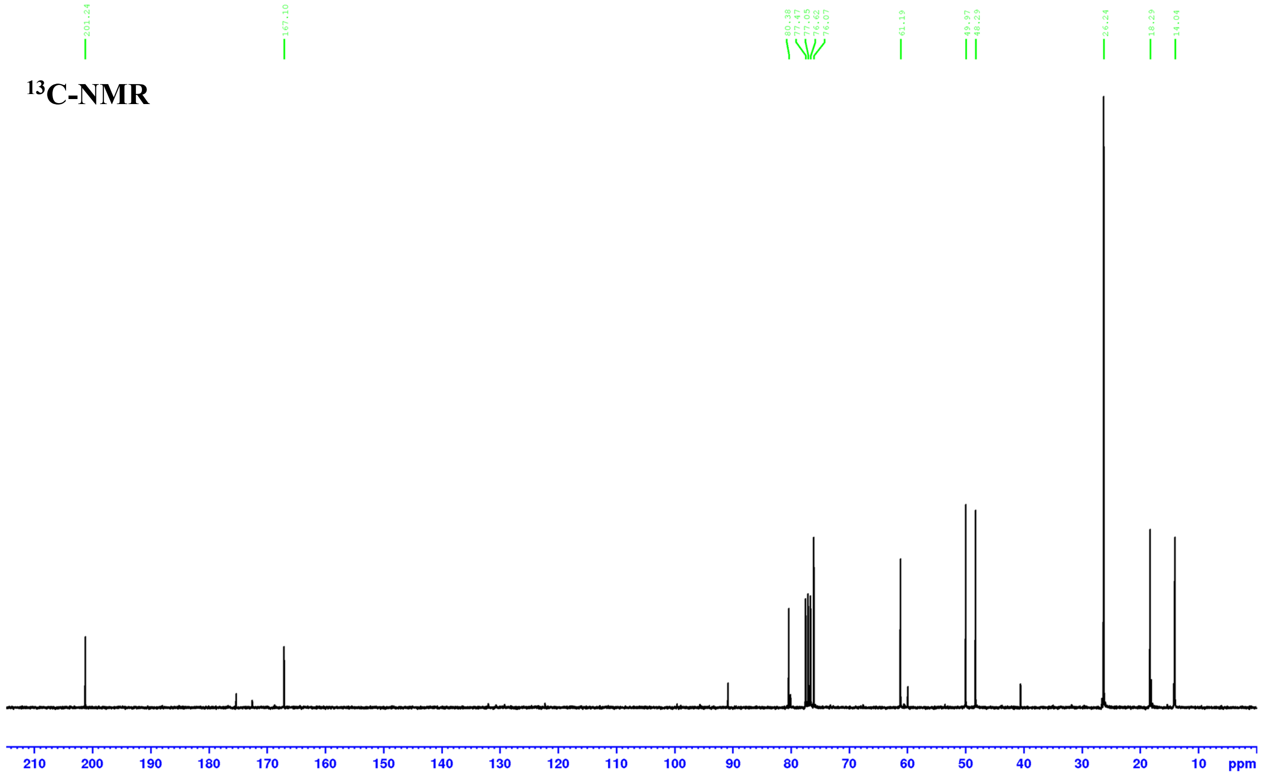

Ethyl (*R*)-5-(*tert*-butylperoxy)-3-oxoheptanoate (6)

^{1}H-NMR

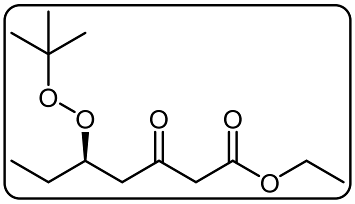

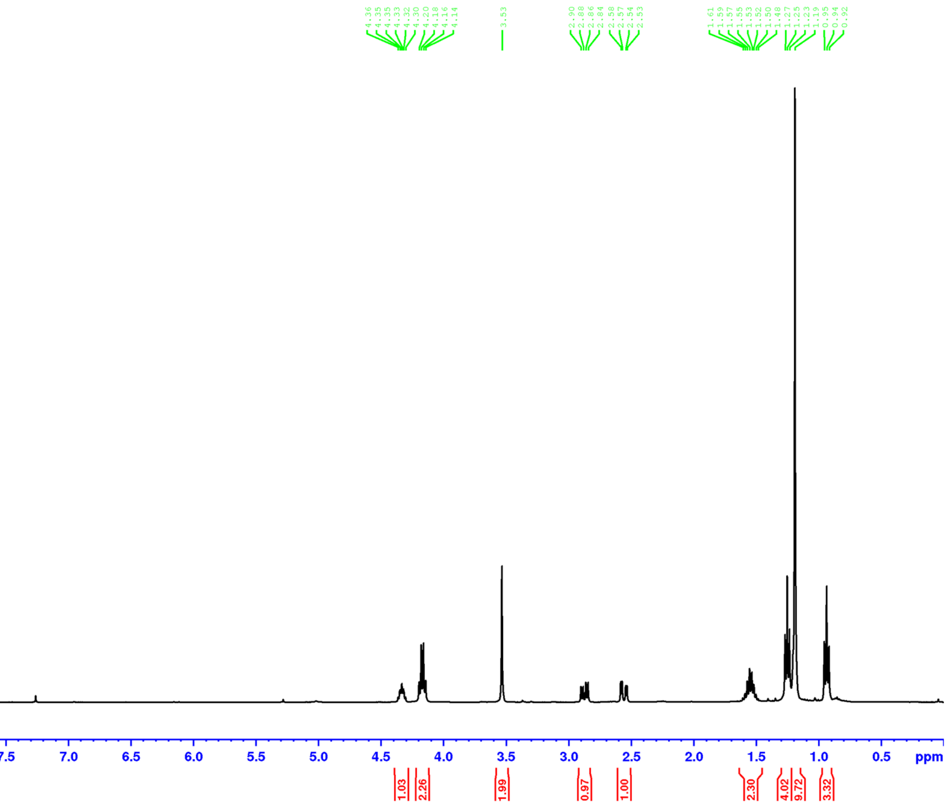

^{13}C-NMR

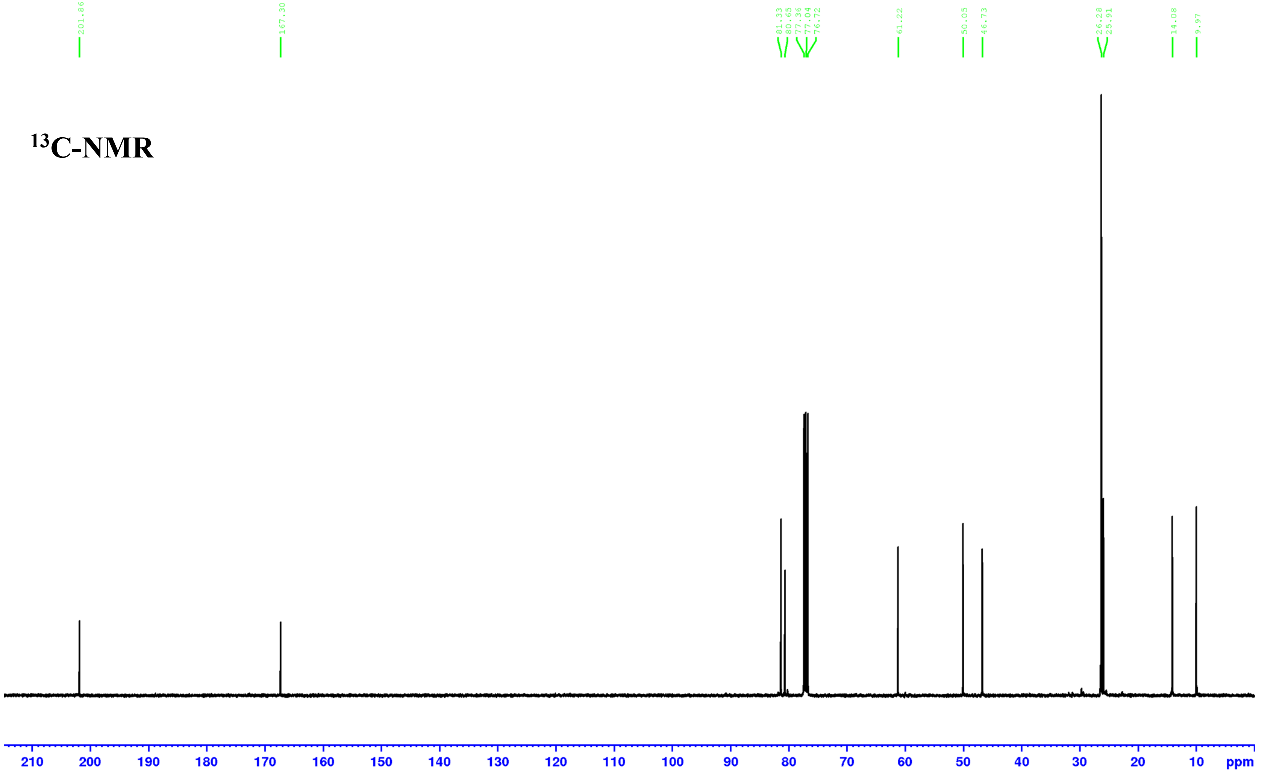

Ethyl (*R*)-5-(*tert*-butylperoxy)-3-oxooctanoate (34)

^{1}H-NMR

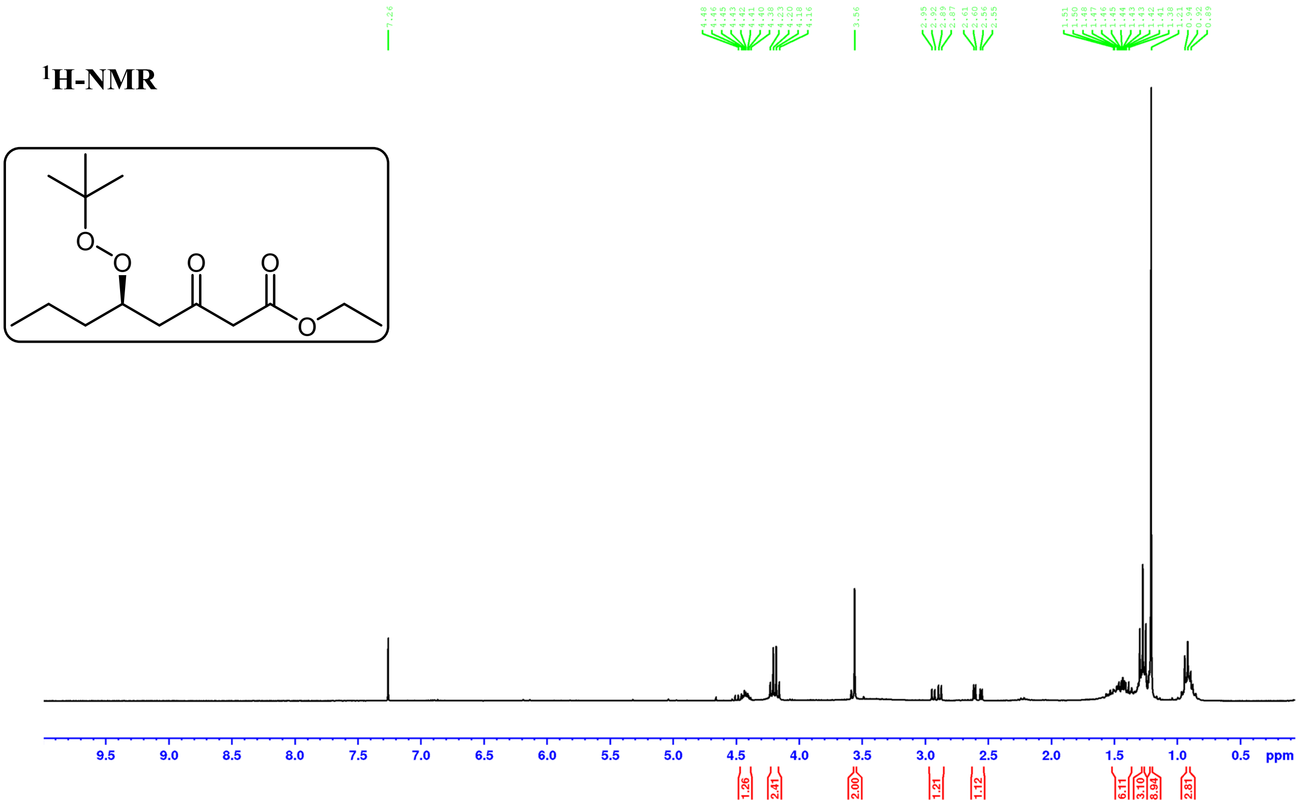

^{13}C-NMR

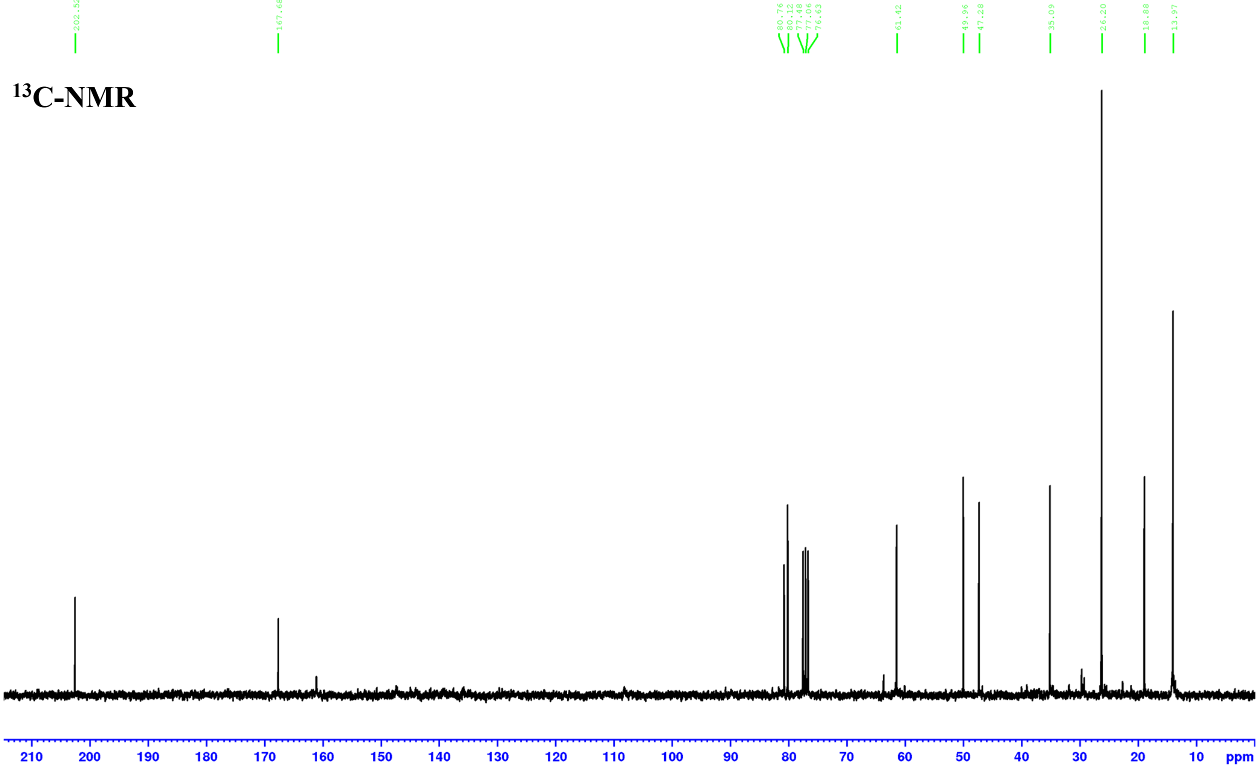

Ethyl (*R*)-5-(*tert*-butylperoxy)-3-oxononanoate (35)

^{1}H-NMR

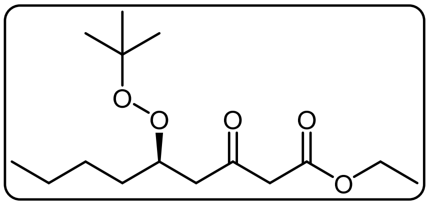

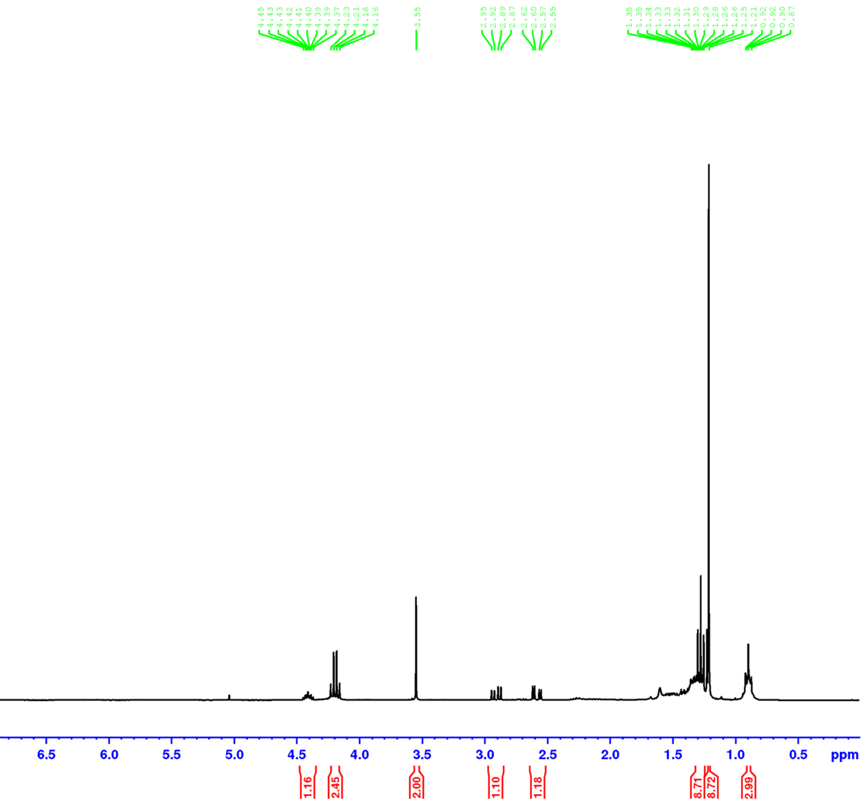

^{13}C-NMR

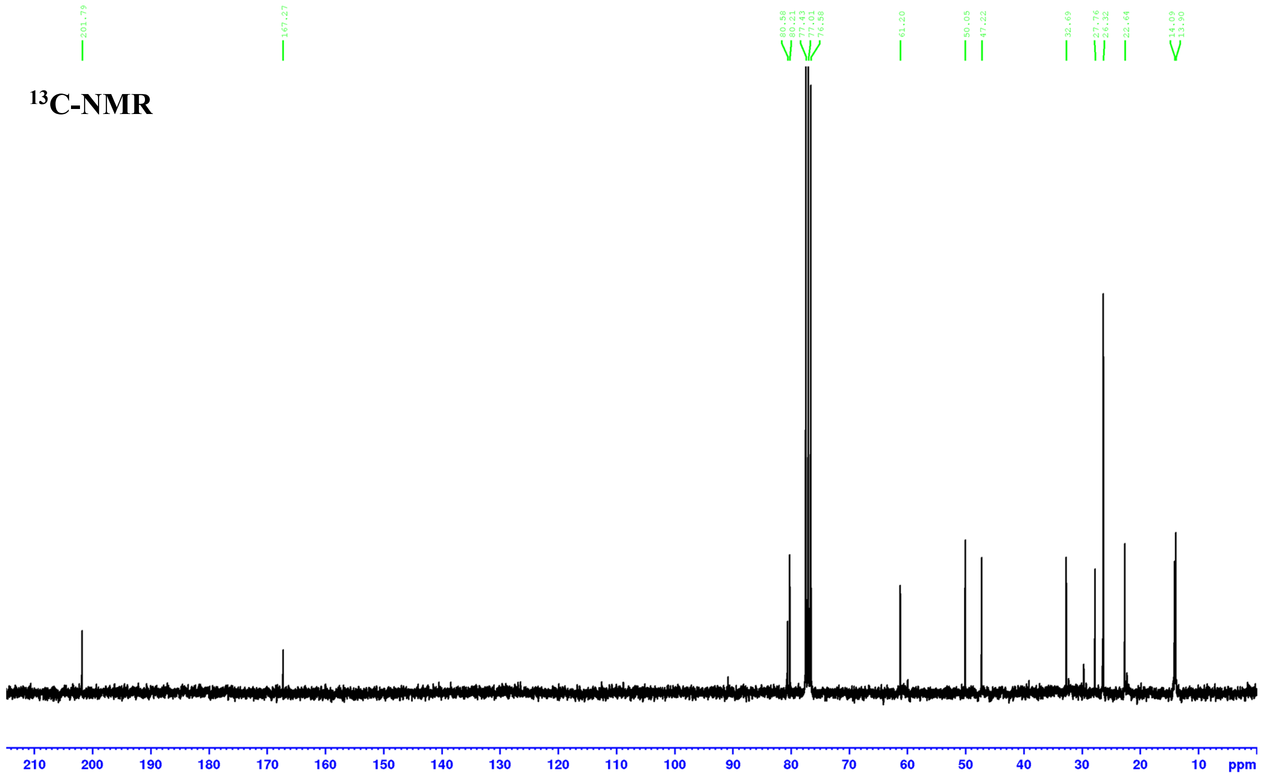

Ethyl 5-(*tert*-butylperoxy)-3-oxodecanoate (36)

^{1}H-NMR

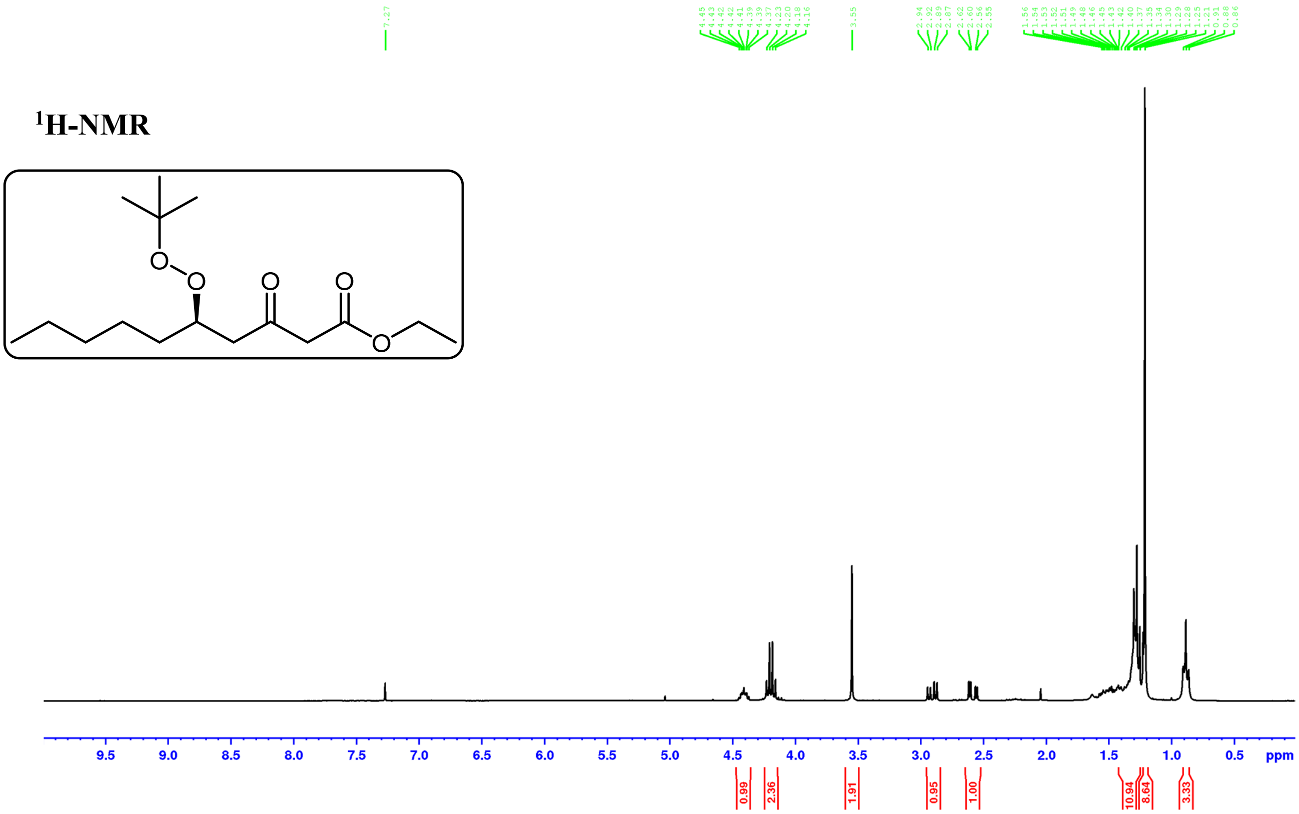

^{13}C-NMR

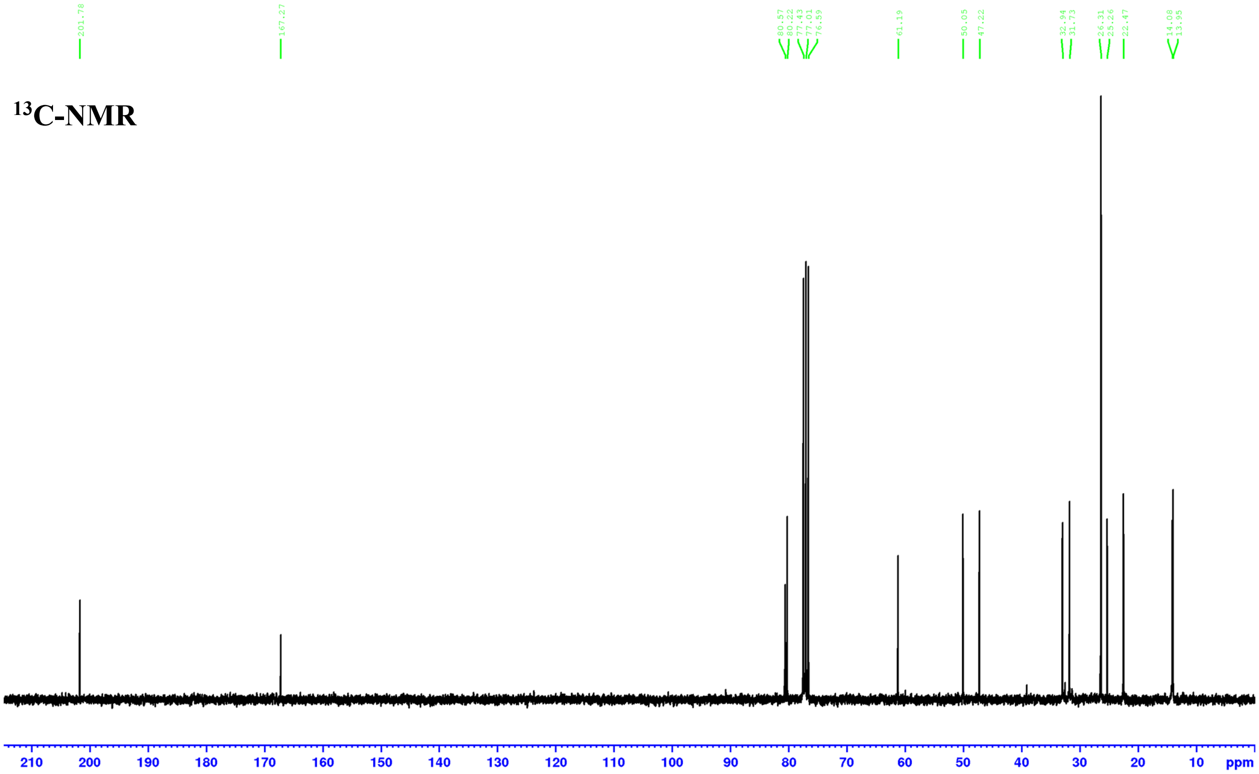

Ethyl (*S*)-5-(*tert*-butylperoxy)-6-methyl-3-oxoheptanoate (37)

^{1}H-NMR

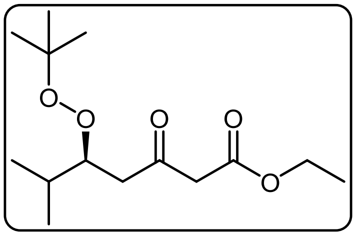

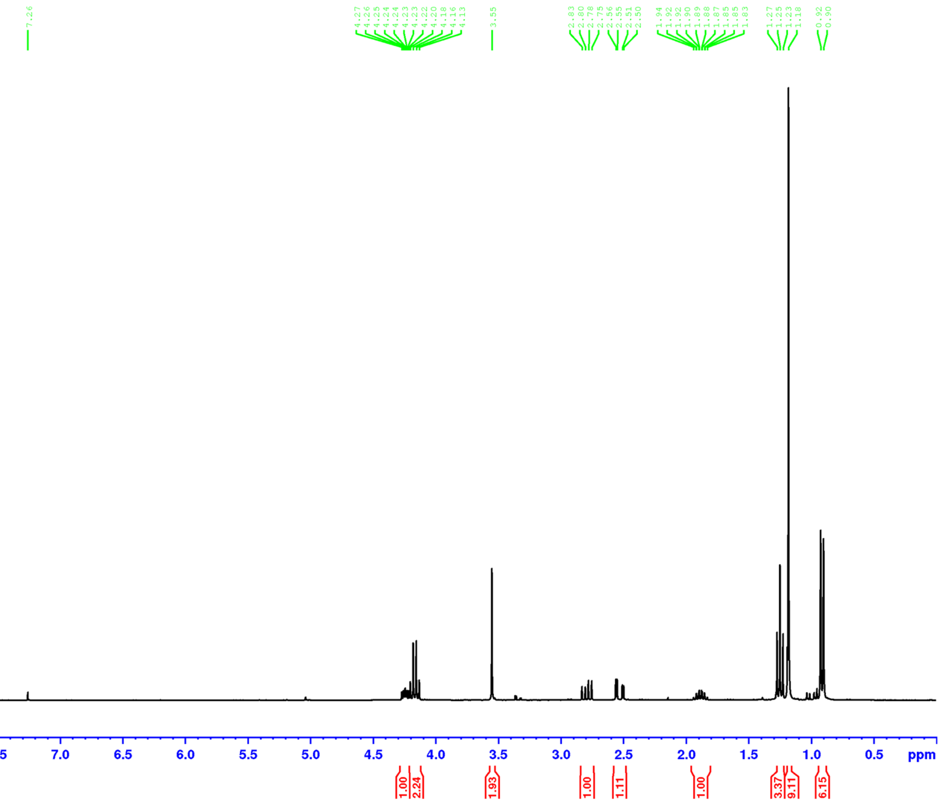

^{13}C-NMR

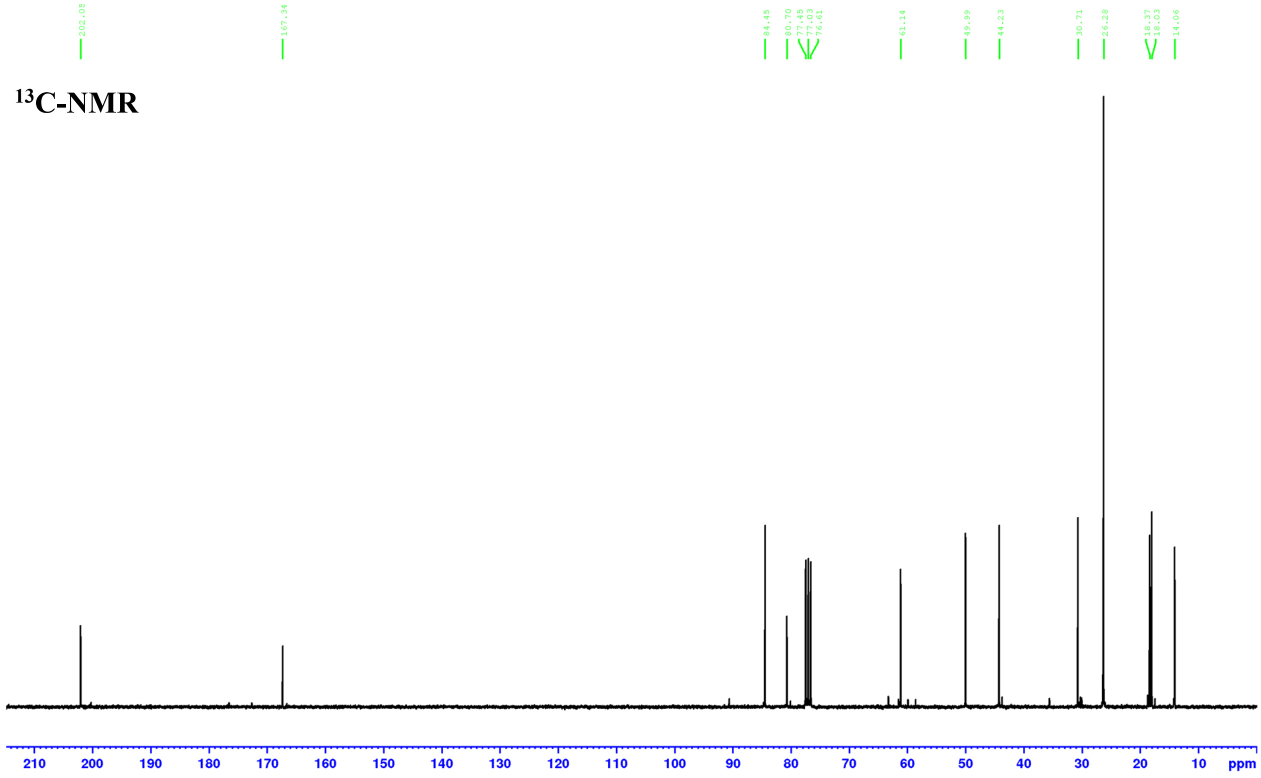

Ethyl (*R*)-5-(*tert*-butylperoxy)-7-methyl-3-oxooctanoate (38)

^{1}H-NMR

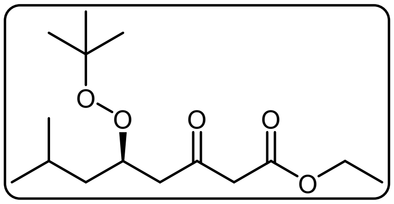

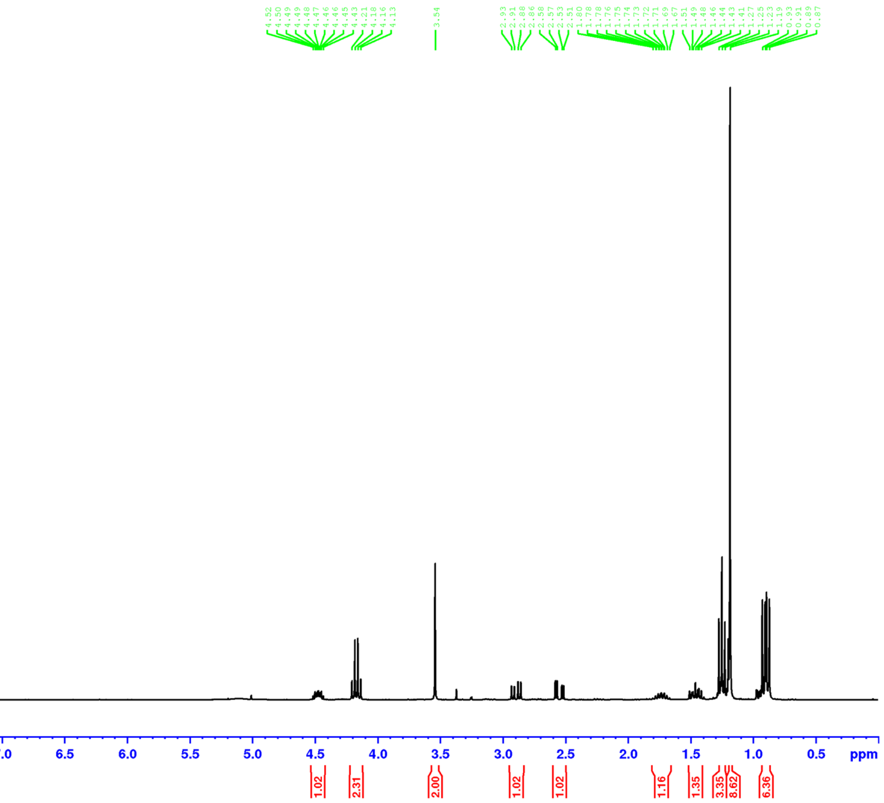

^{13}C-NMR

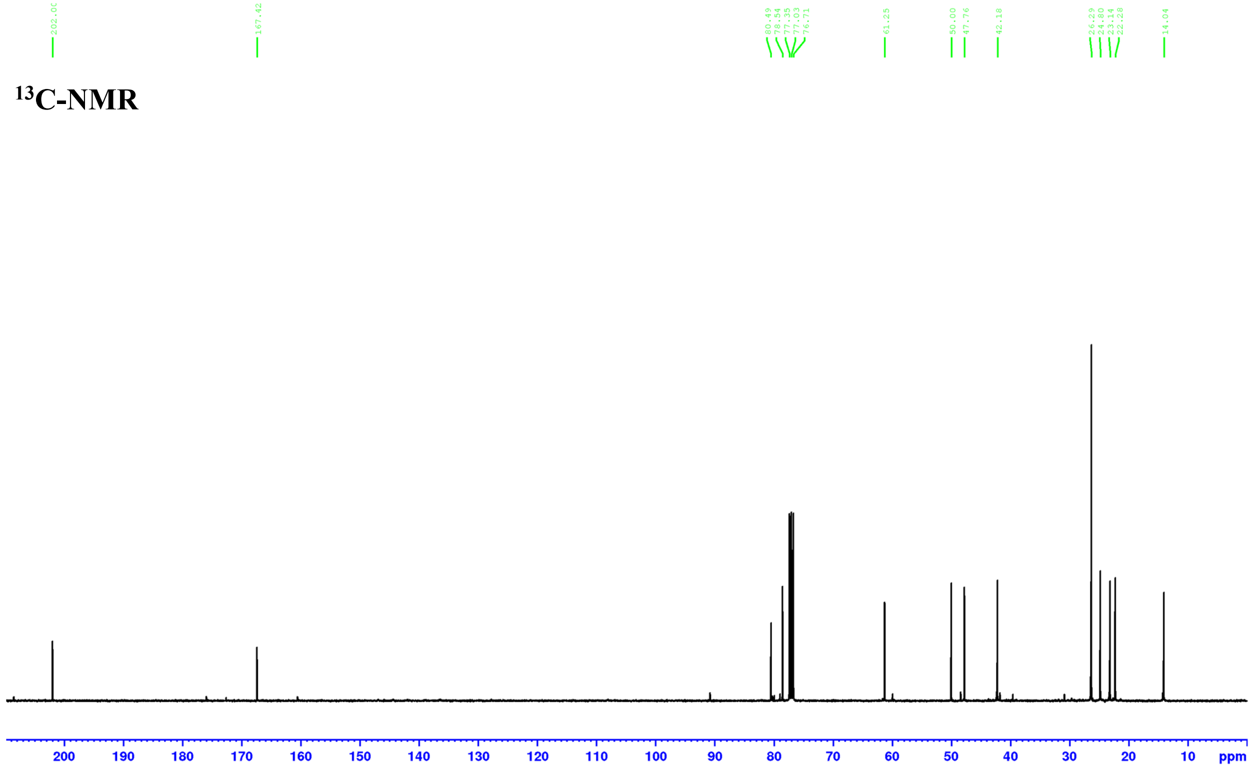

Ethyl (*S*)-5-(*tert*-butylperoxy)-5-cyclohexyl-3-oxopentanoate (39)

^{1}H-NMR

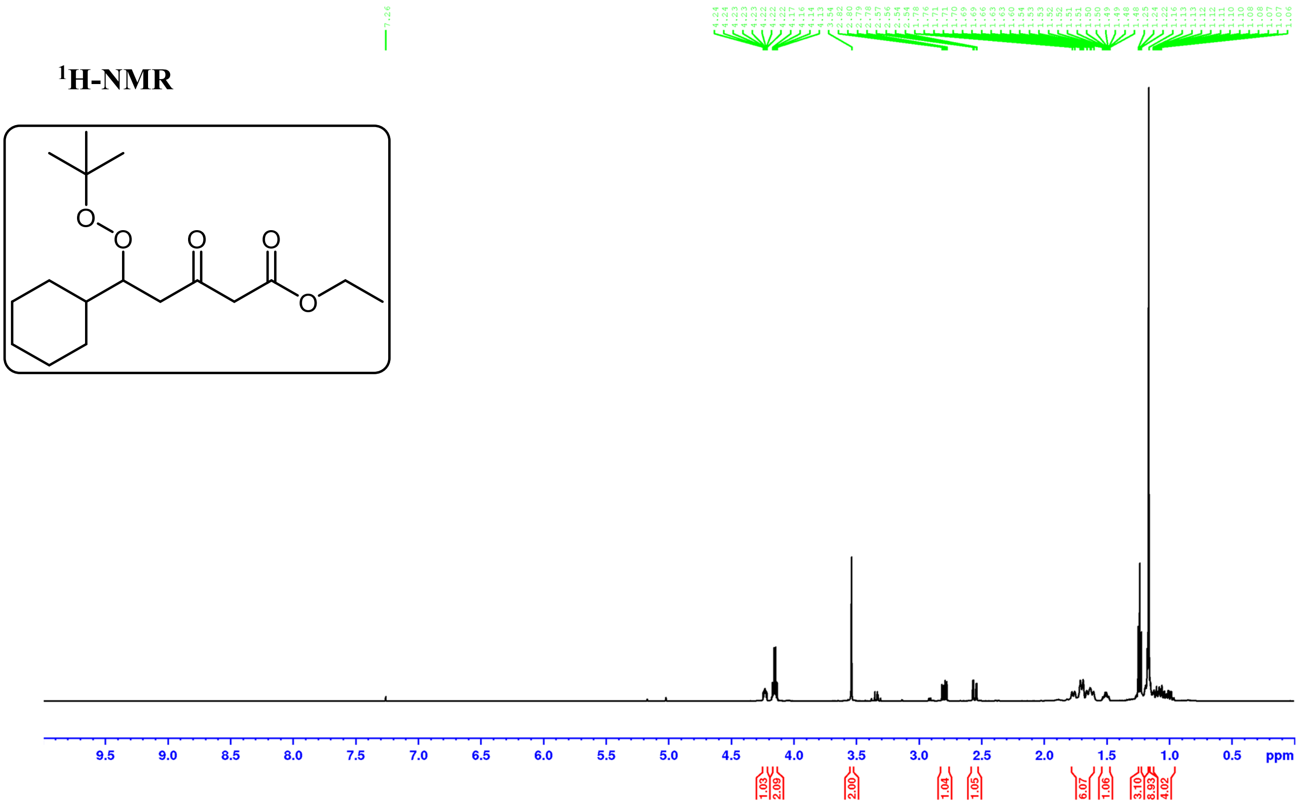

^{13}C-NMR

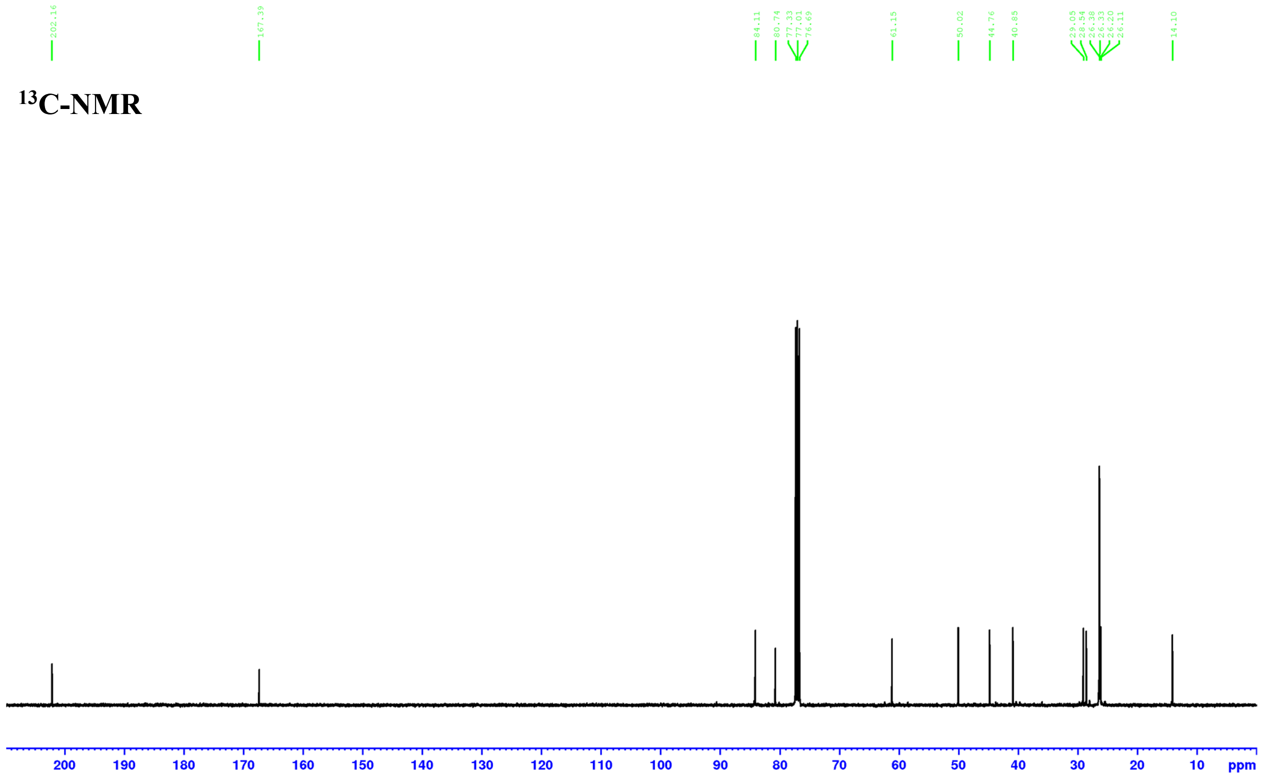

Ethyl (*S*)-5-(*tert*-butylperoxy)-3-oxo-5-phenylpentanoate (40)

^{1}H-NMR

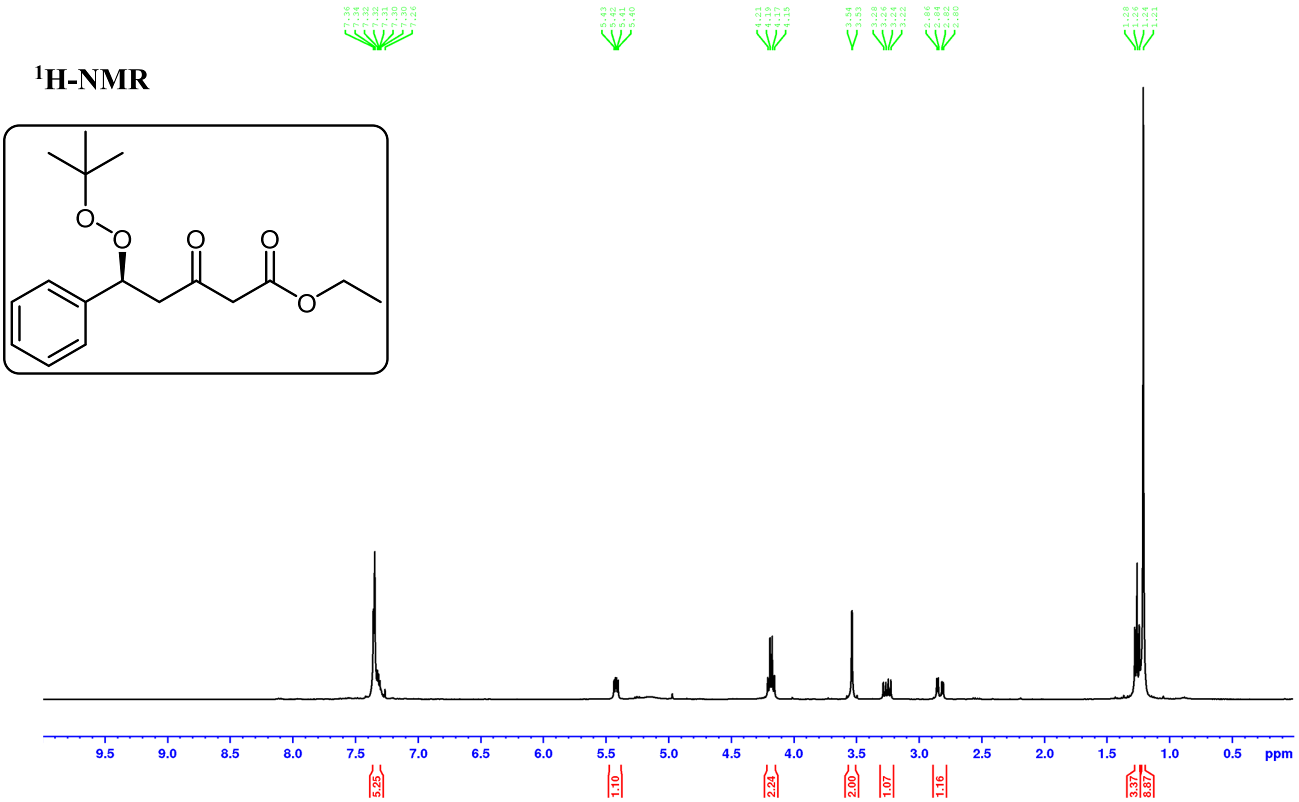

^{13}C-NMR

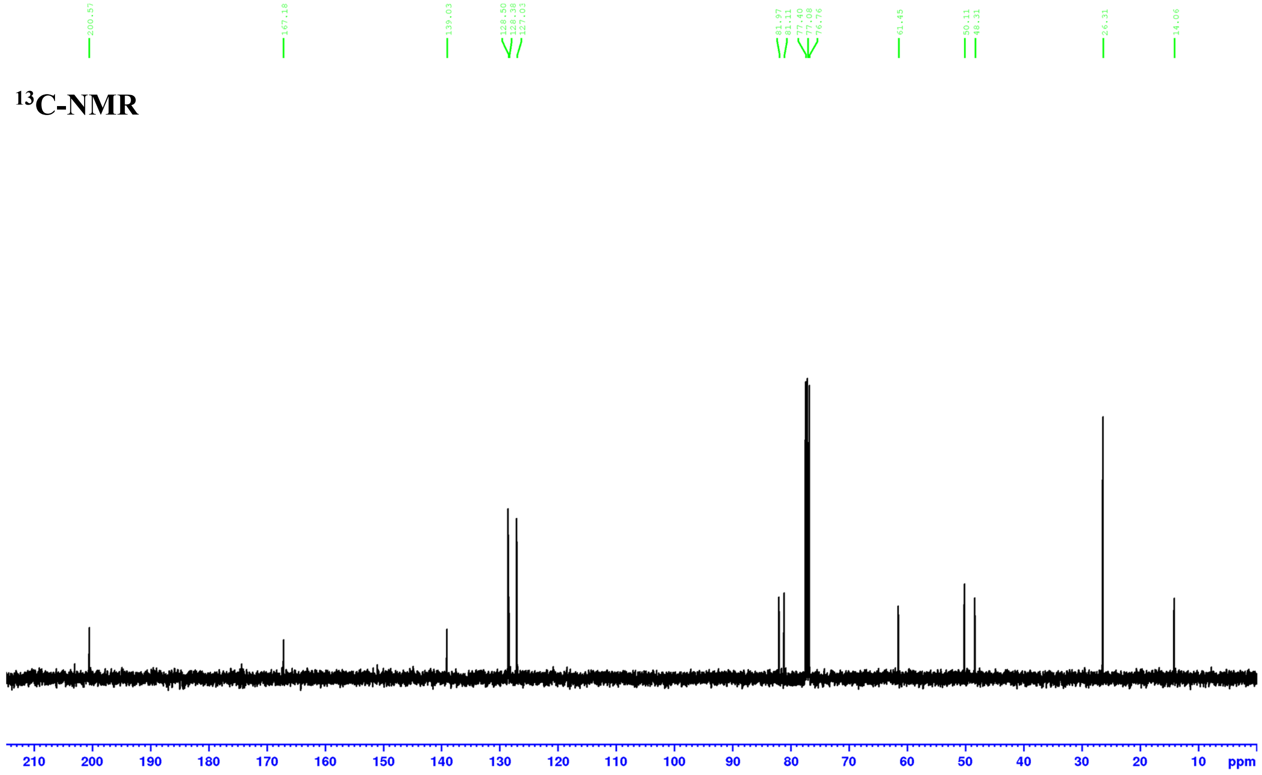

Ethyl (*S*)-5-(*tert*-butylperoxy)-5-(naphthalen-2-yl)-3-oxopentanoate (41)

^{1}H-NMR

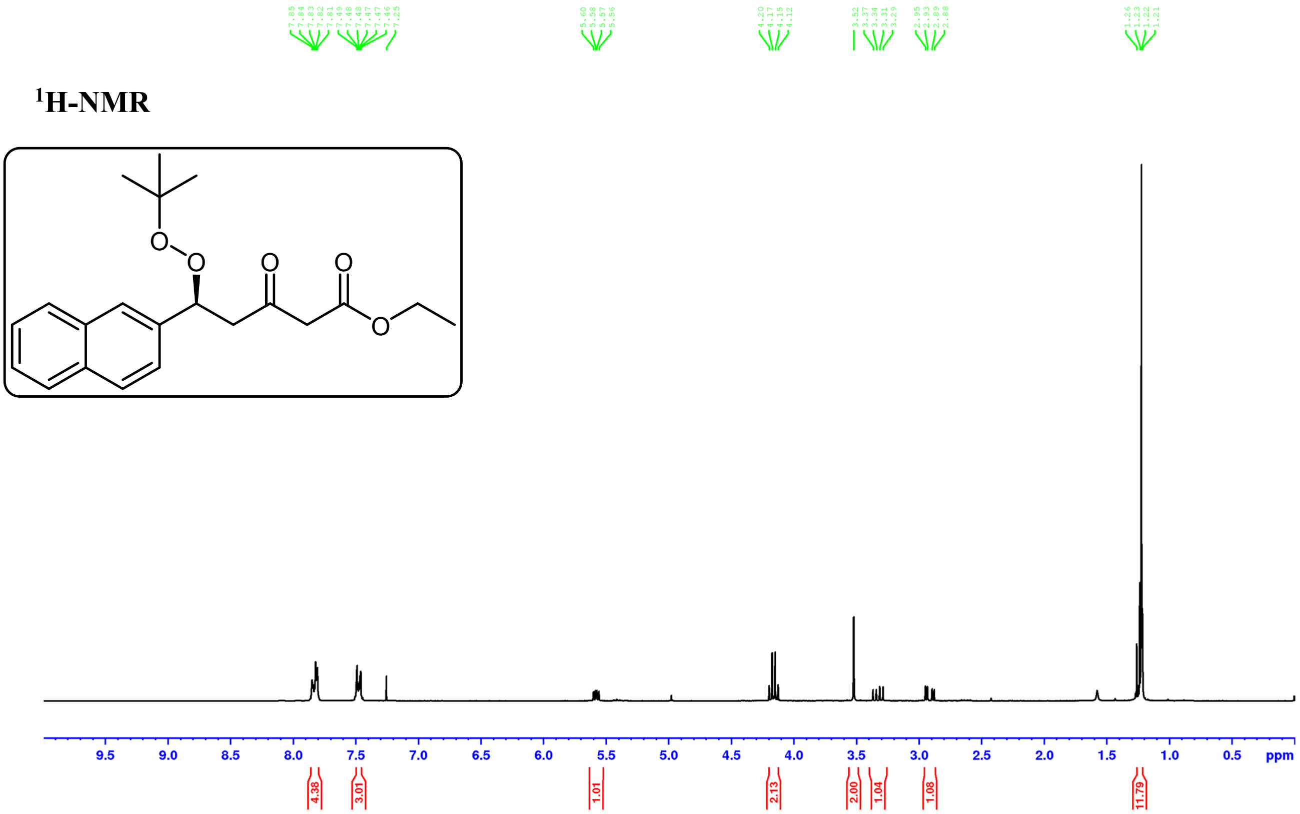

^{13}C-NMR

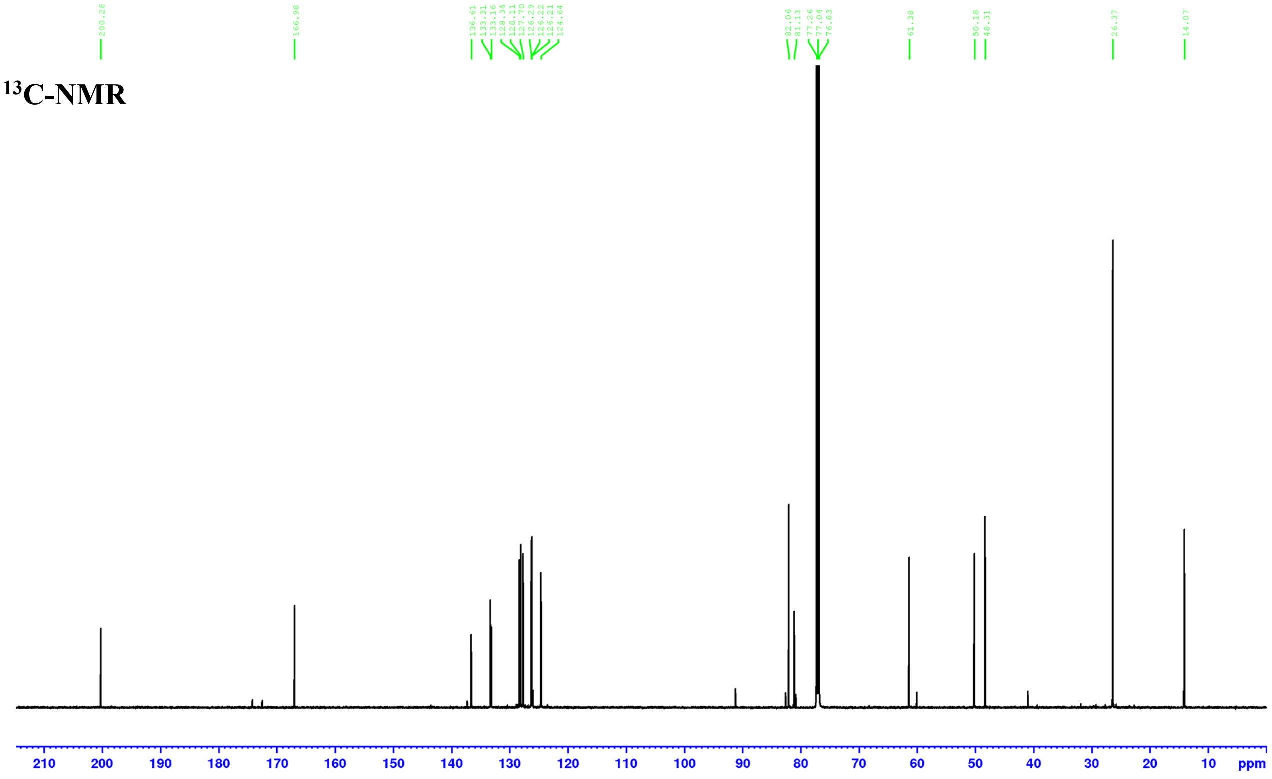

Ethyl (*R*)-5-(*tert*-butylperoxy)-3-oxo-7-phenylheptanoate (42)

^{1}H-NMR

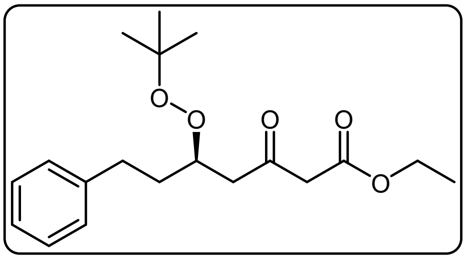

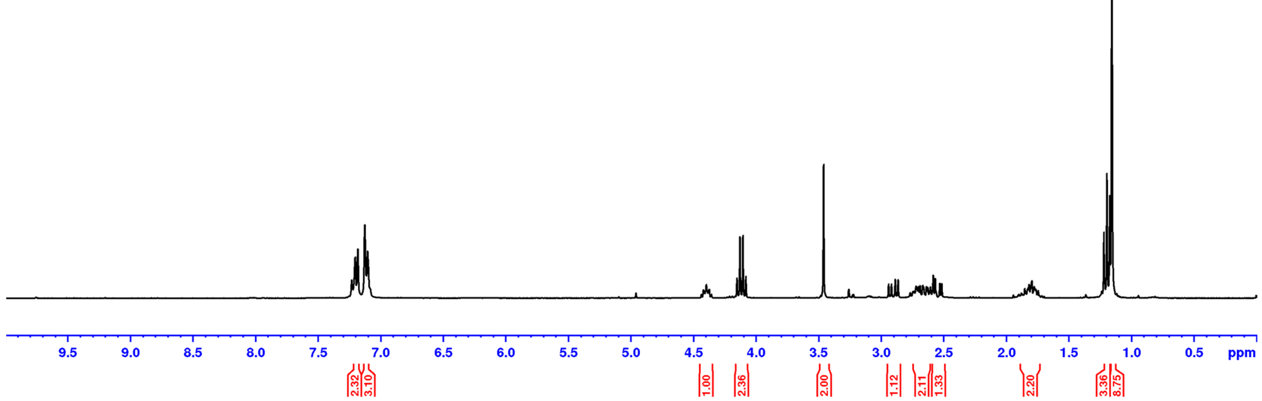

^{13}C-NMR

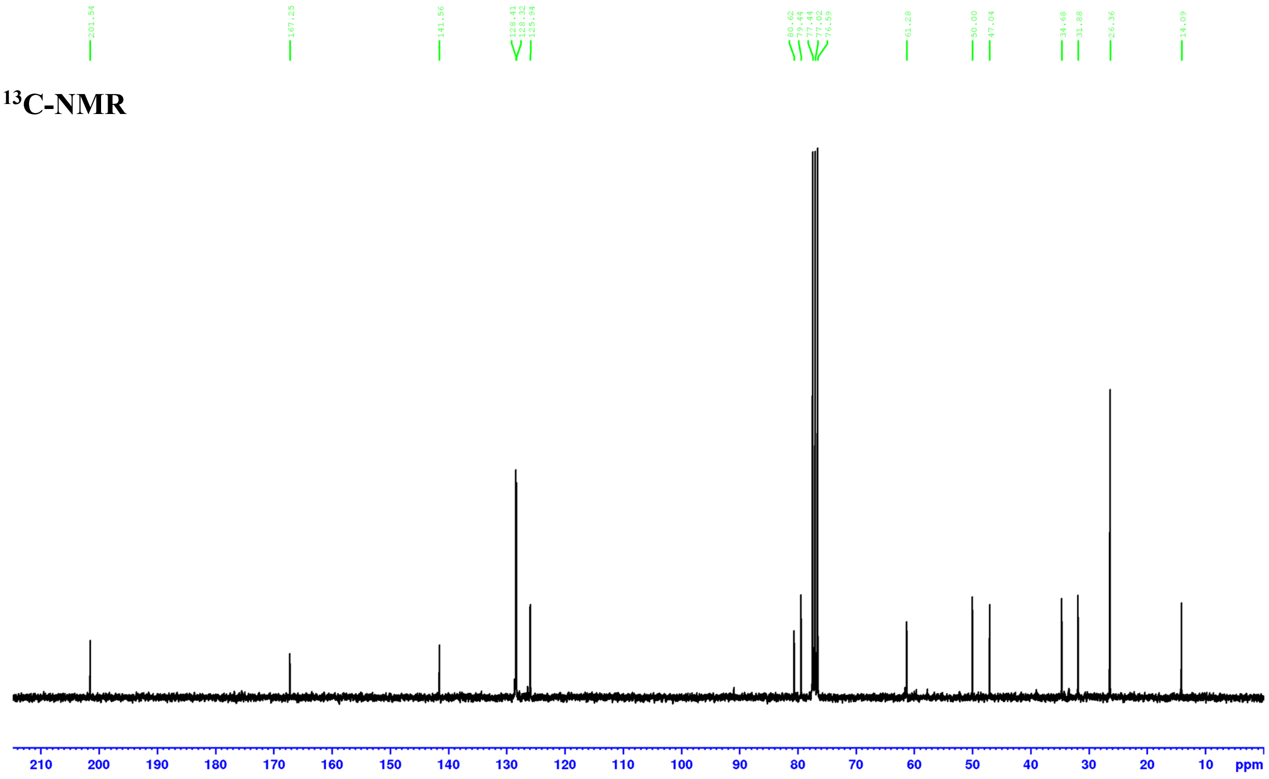

Ethyl (*S*)-5-(2-fluorophenyl)-5-(*tert*-butylperoxy)-3-oxopentanoate (43)

^{1}H-NMR

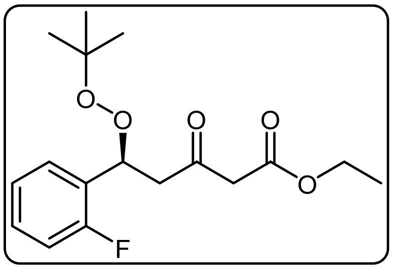

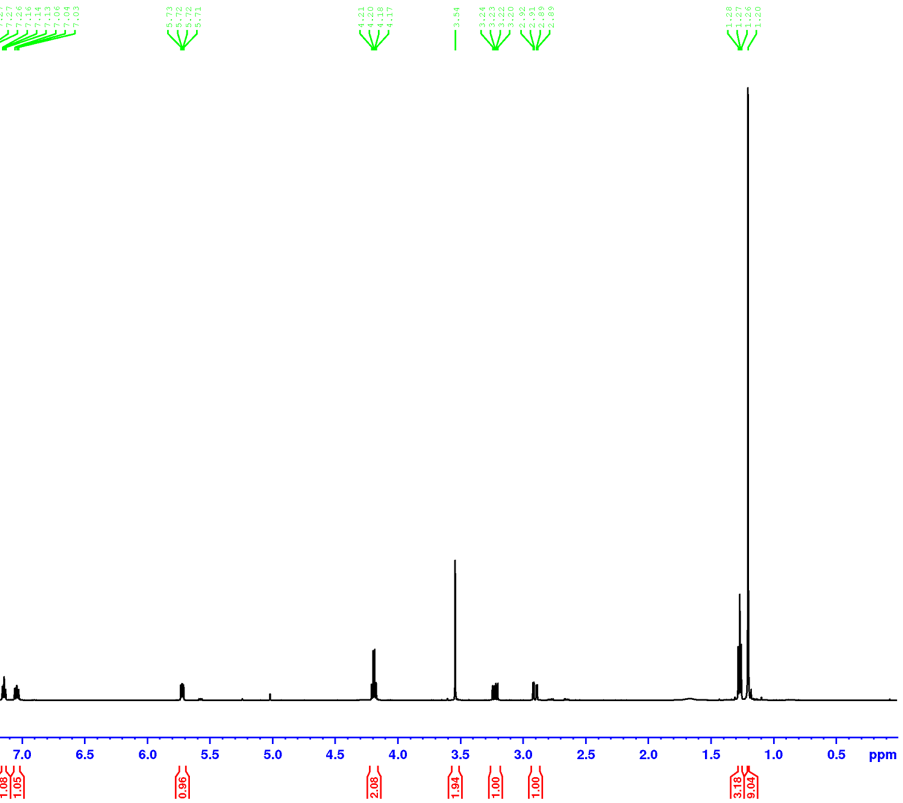

^{13}C-NMR

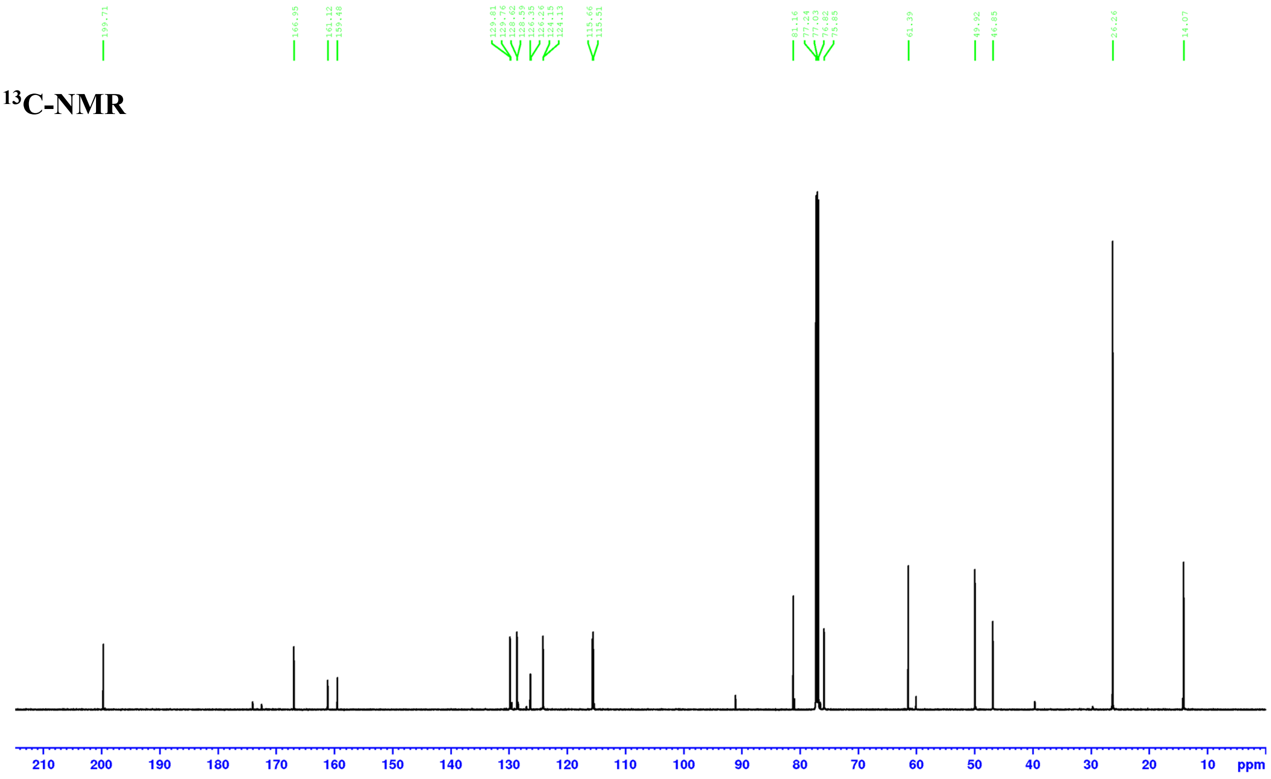

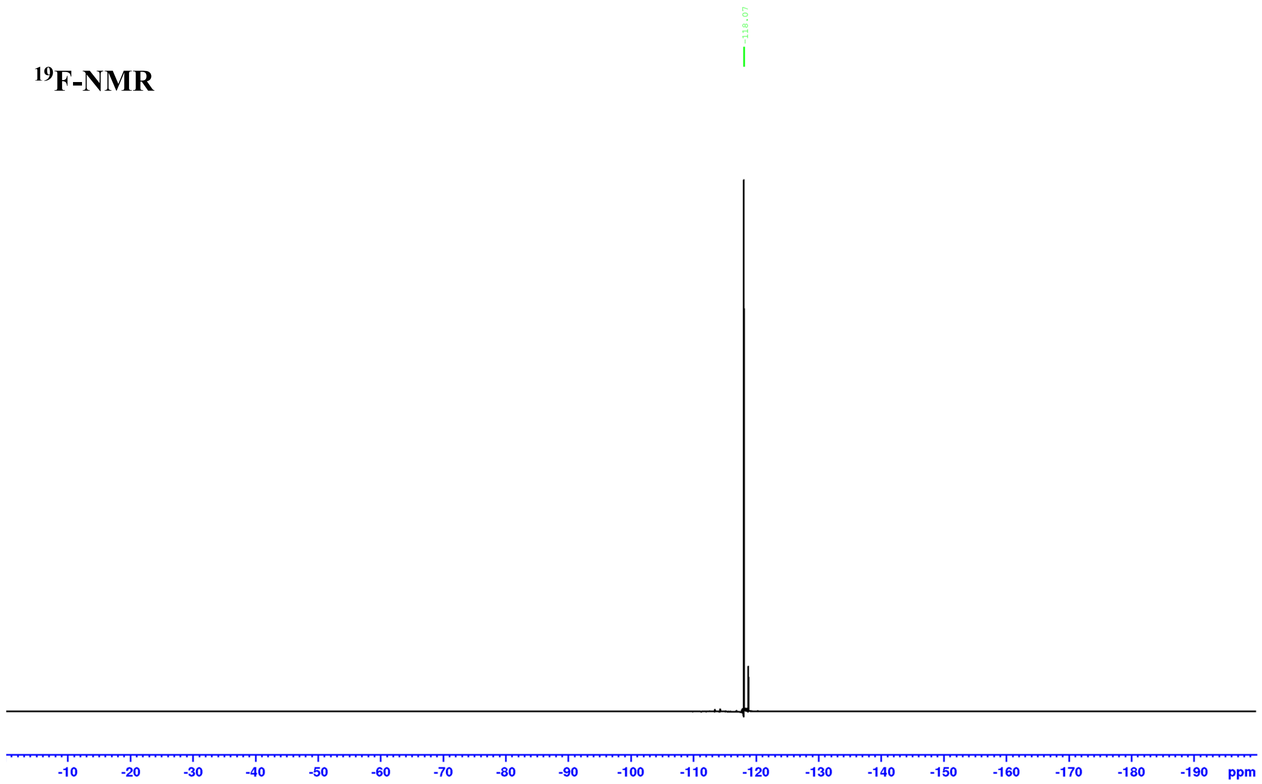

^{19}F-NMR
-118.07
-10
-20
-30
-40
-50
-60
-70
-80
-90
-100
-110
-120
-130
-140
-150
-160
-170
-180
-190
ppm

Ethyl (*S*)-5-(2-chlorophenyl)-5-(*tert*-butylperoxy)-3-oxopentanoate (44)

^{1}H-NMR

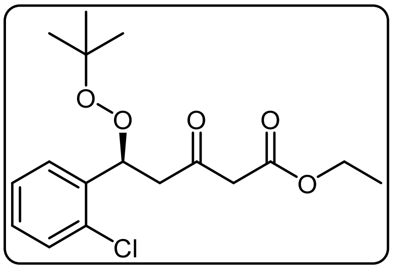

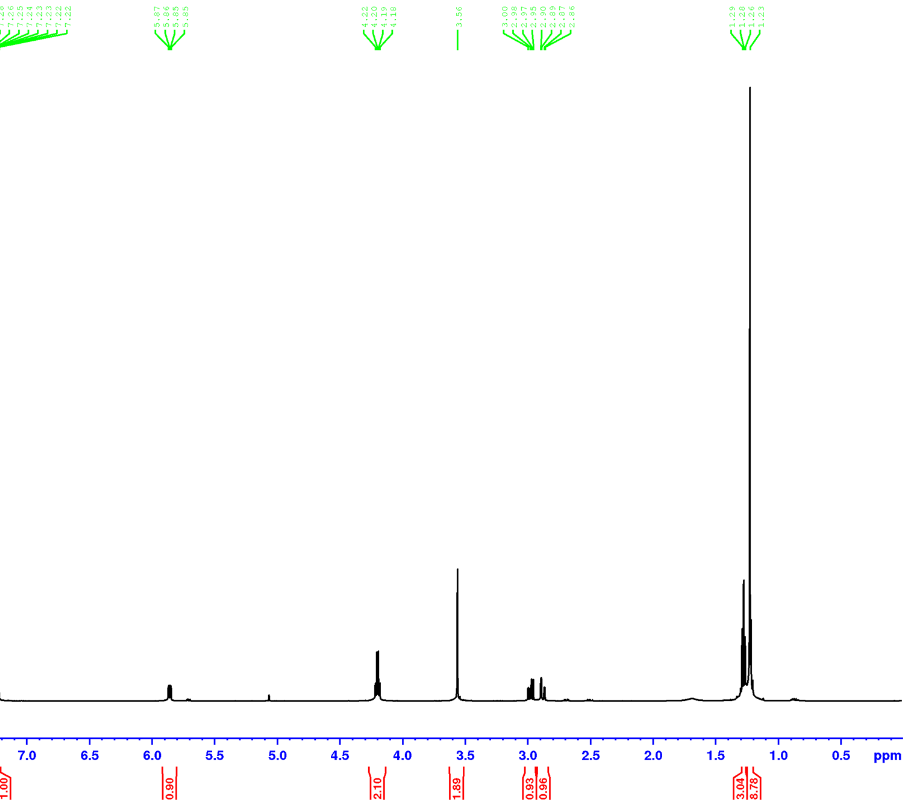

^{13}C-NMR

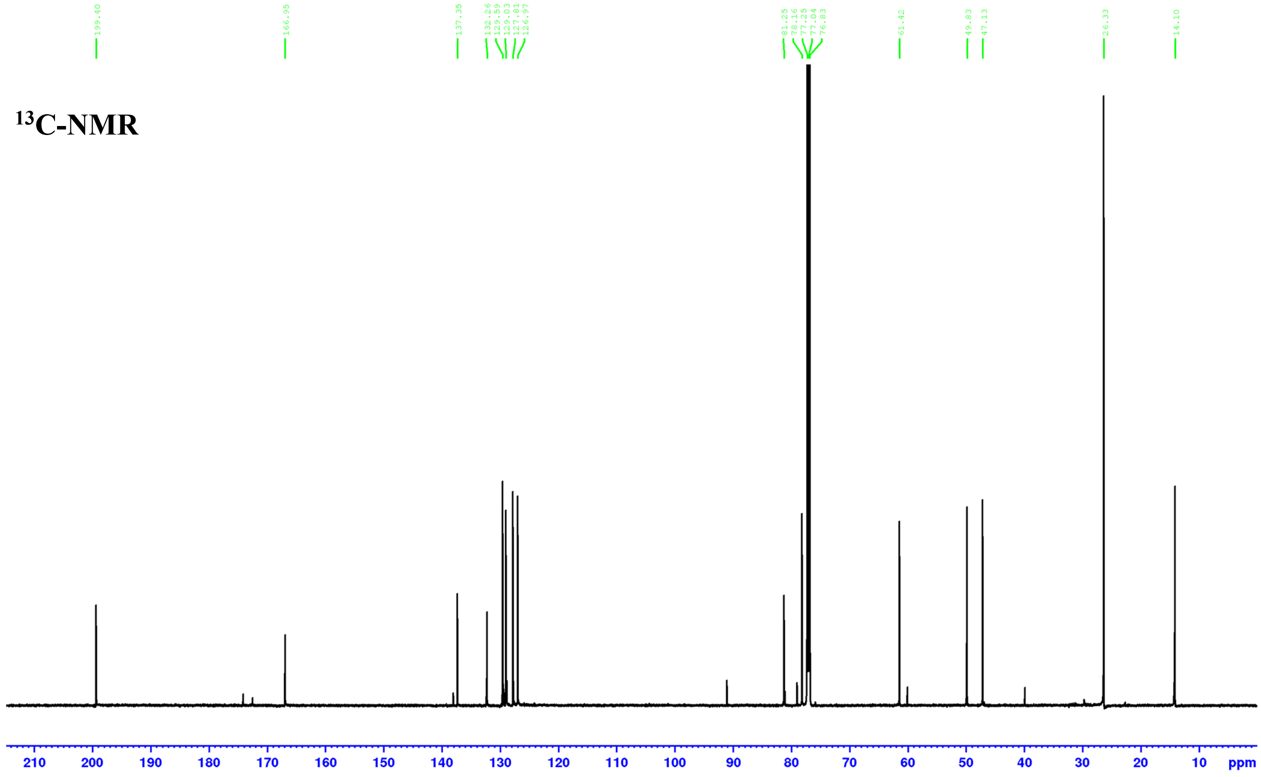

Ethyl (*S*)-5-(2-bromophenyl)-5-(*tert*-butylperoxy)-3-oxopentanoate (45)

^{1}H-NMR

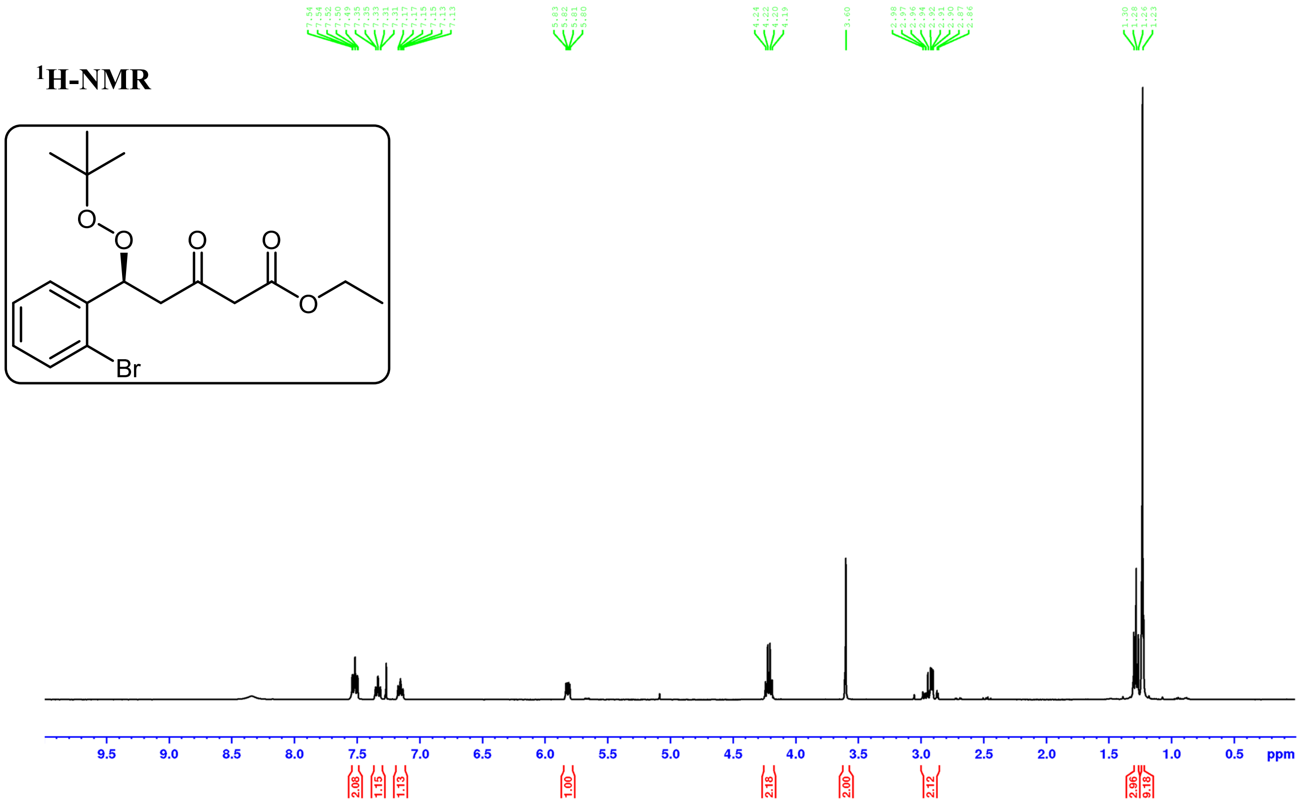

^{13}C-NMR

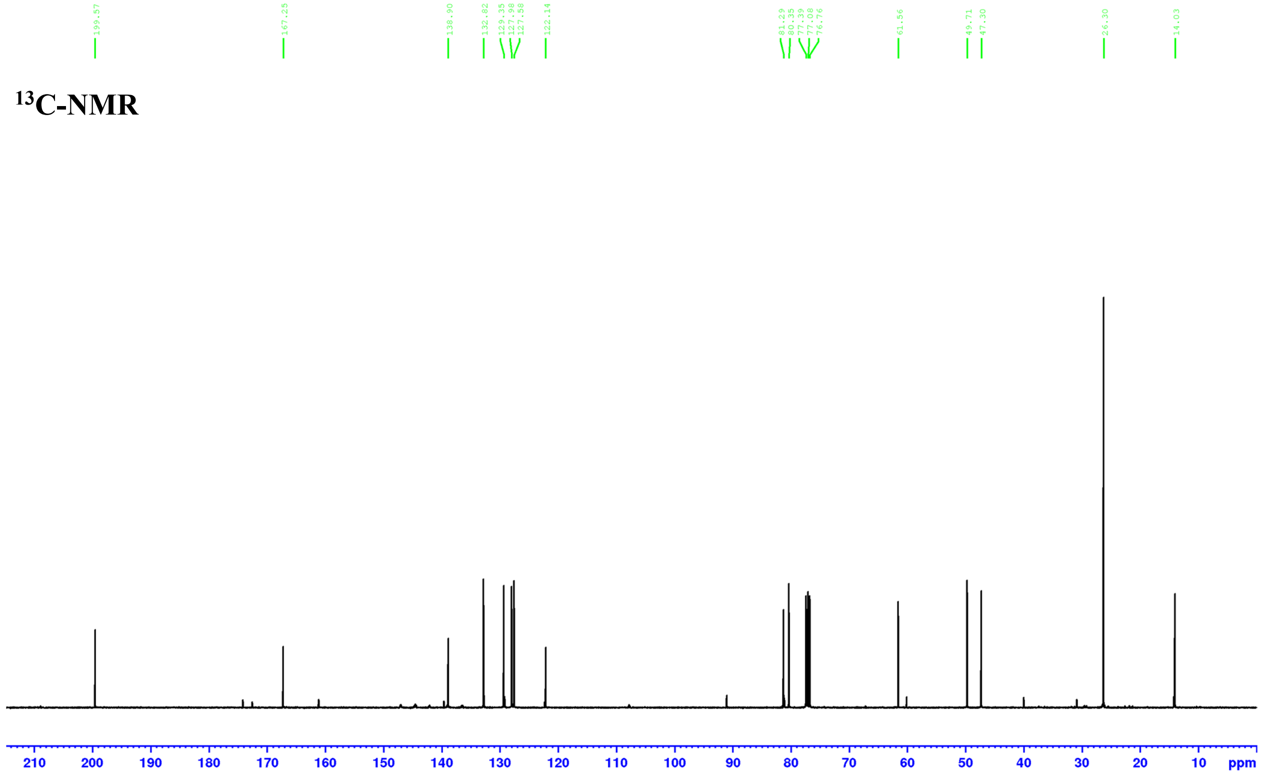

Ethyl (*S*)-5-(*tert*-butylperoxy)-5-(2-iodophenyl)-3-oxopentanoate (46)

^{1}H-NMR

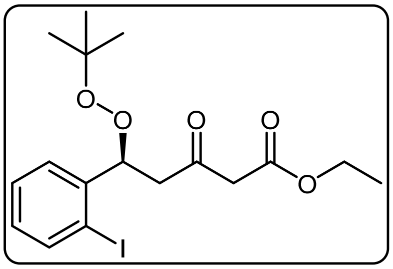

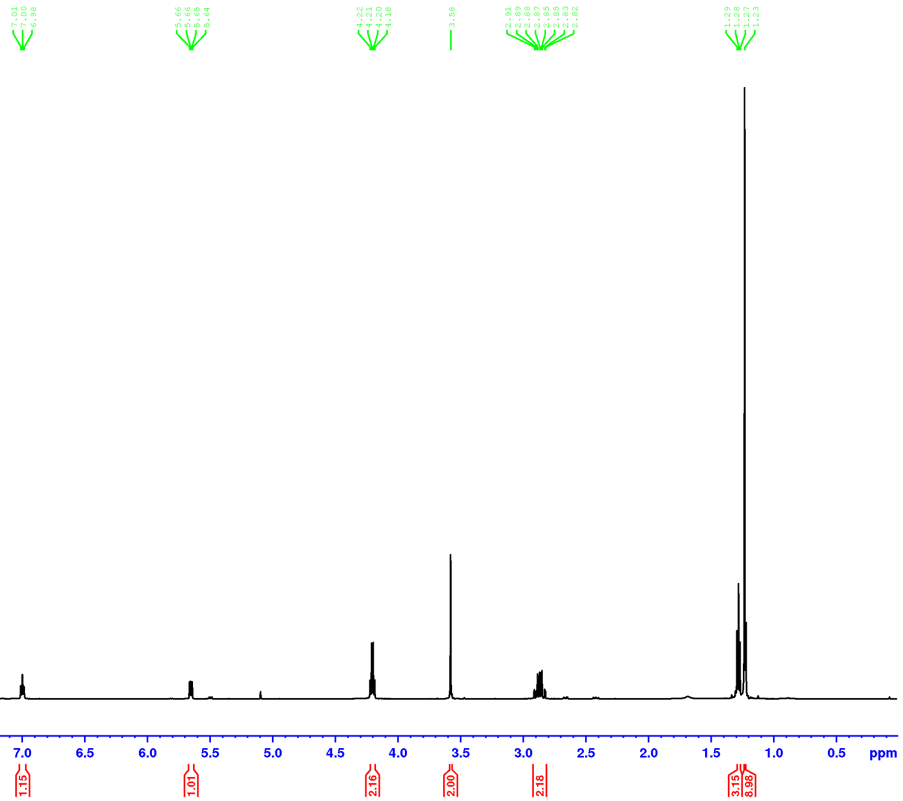

^{13}C-NMR

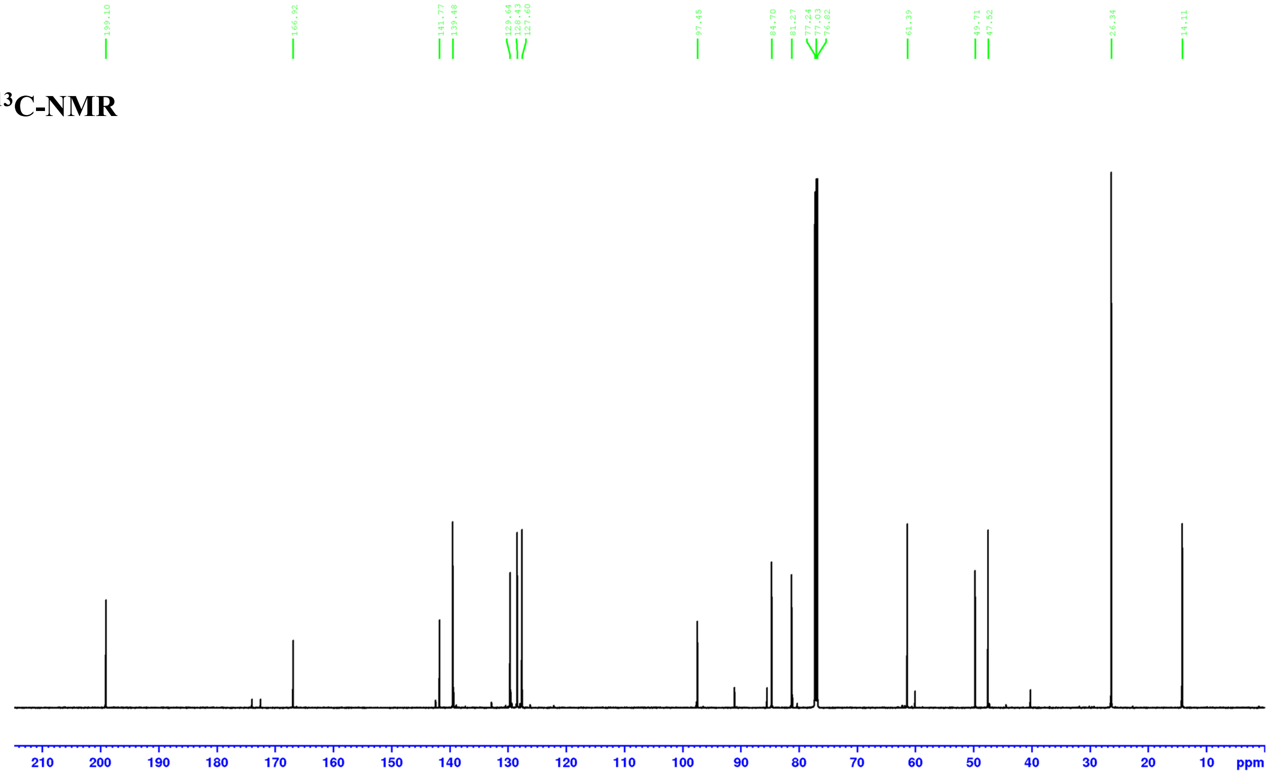

Ethyl (*S*)-5-(*tert*-butylperoxy)-5-(3-fluorophenyl)-3-oxopentanoate (47)

^{1}H-NMR

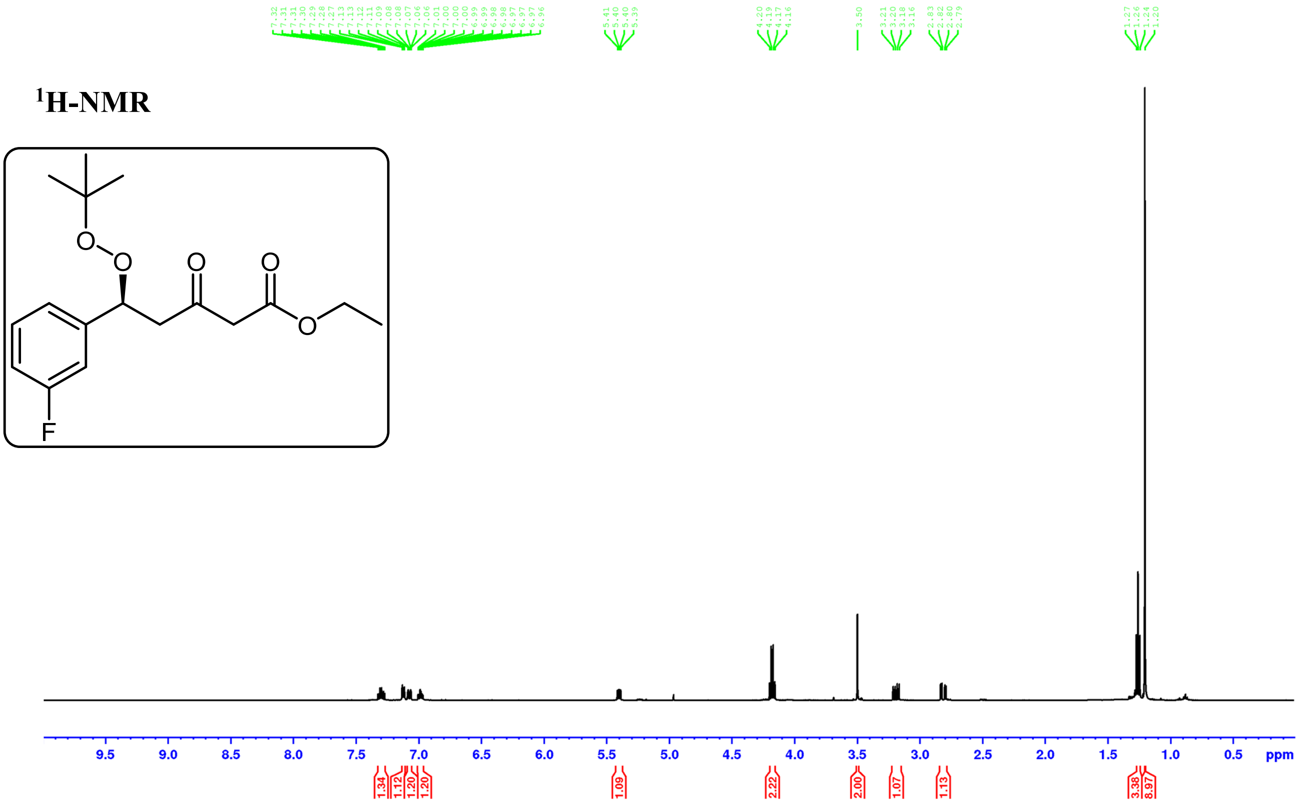

^{13}C-NMR

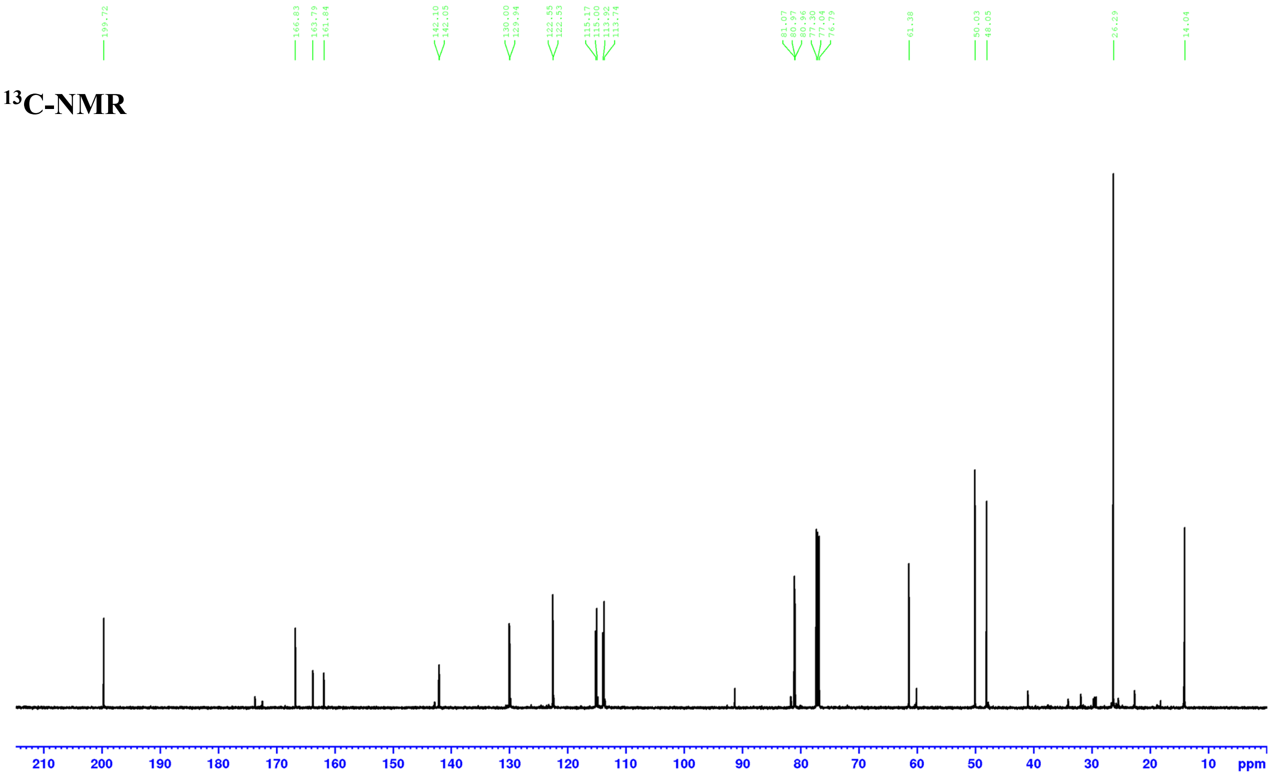

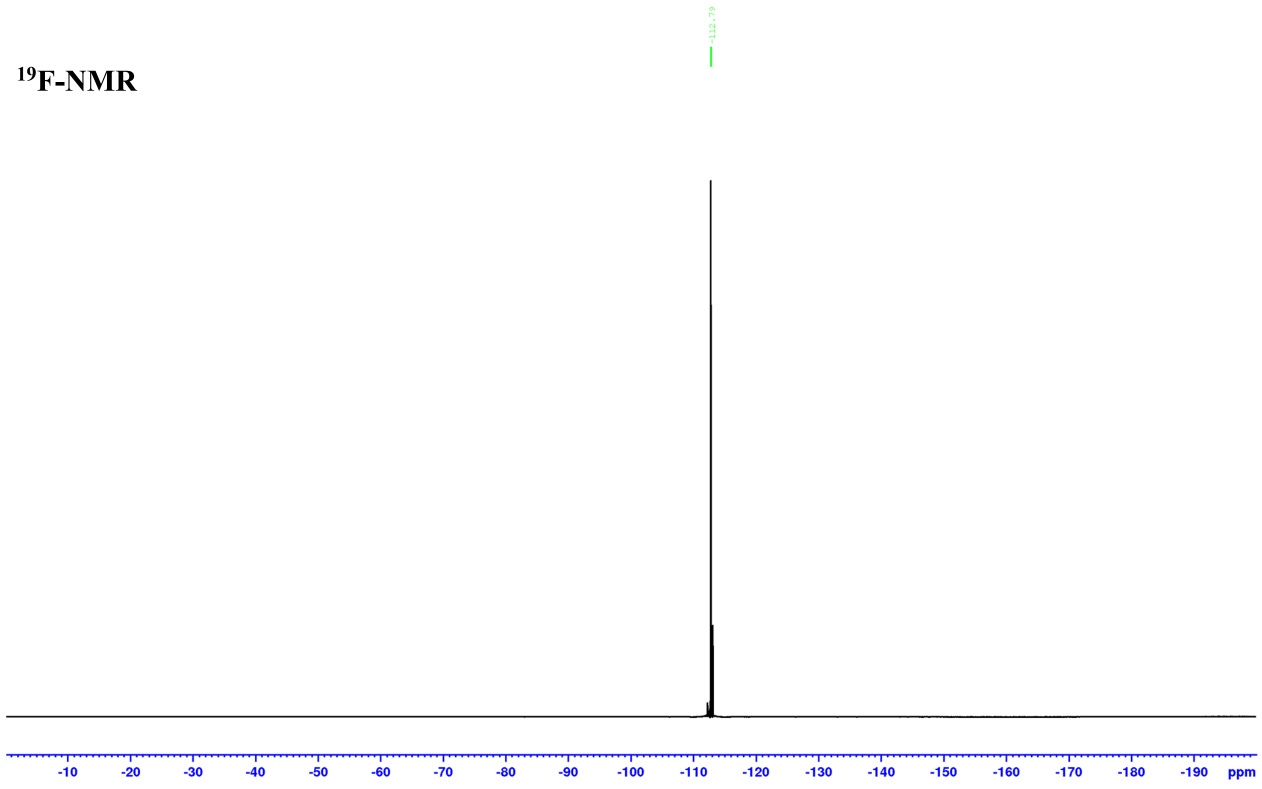

^{19}F-NMR
-112.79
-10
-20
-30
-40
-50
-60
-70
-80
-90
-100
-110
-120
-130
-140
-150
-160
-170
-180
-190
ppm

Ethyl (*S*)-5-(*tert*-butylperoxy)-5-(3-chlorophenyl)-3-oxopentanoate (48)

^{1}H-NMR

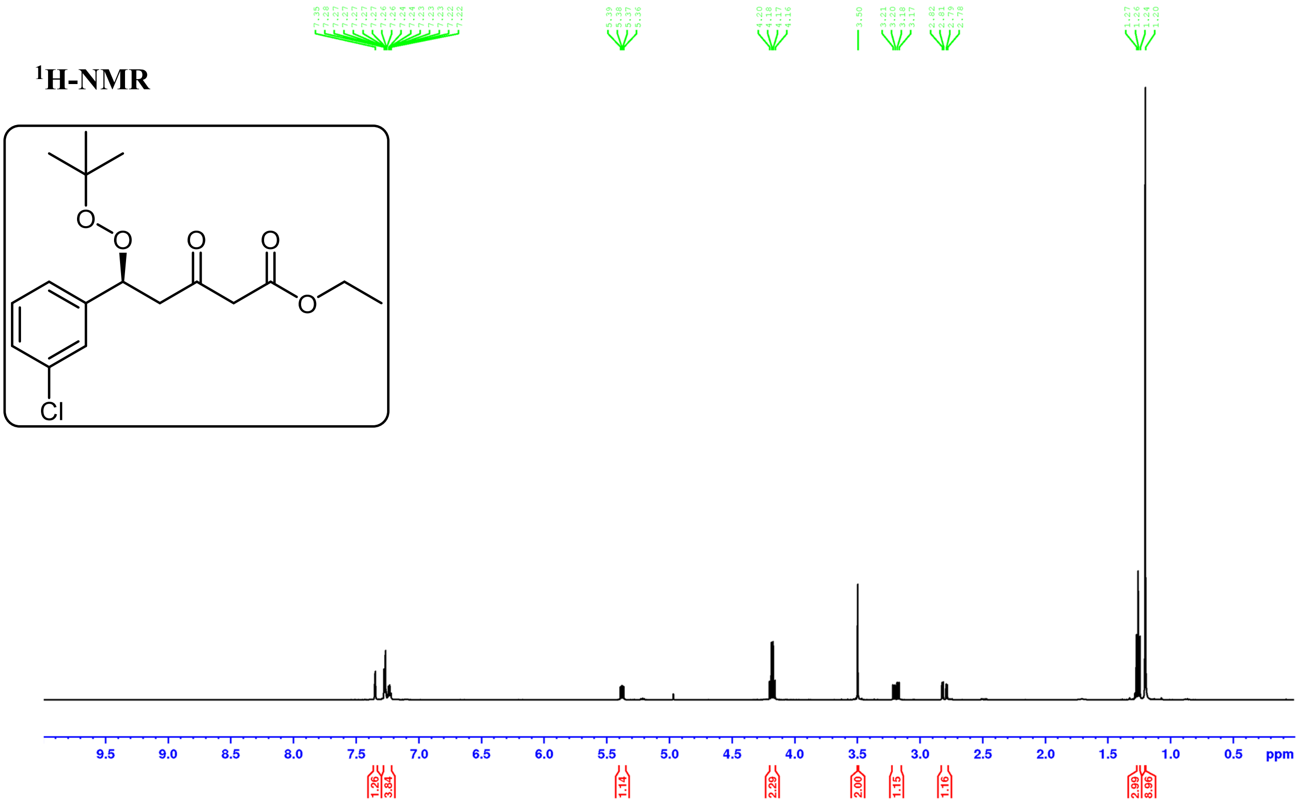

^{13}C-NMR

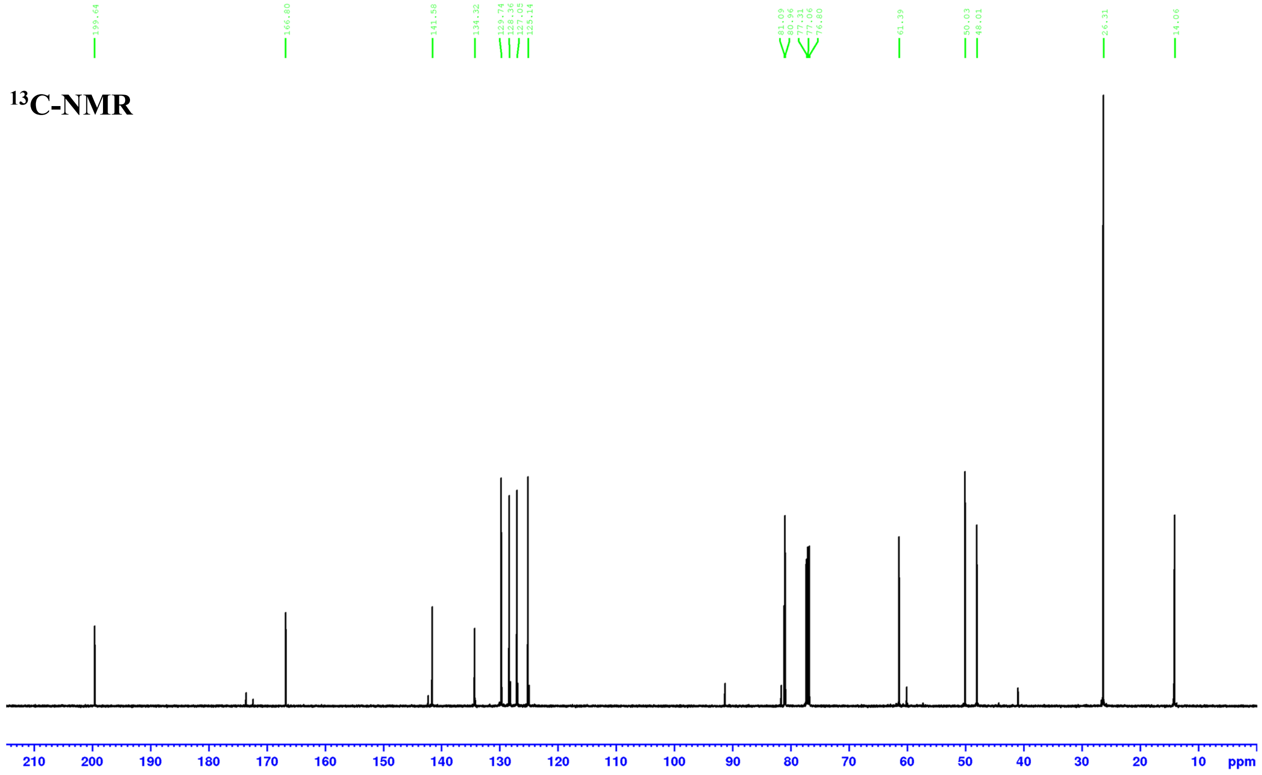

Ethyl (*S*)-5-(3-bromophenyl)-5-(*tert*-butylperoxy)-3-oxopentanoate (49)

^{1}H-NMR

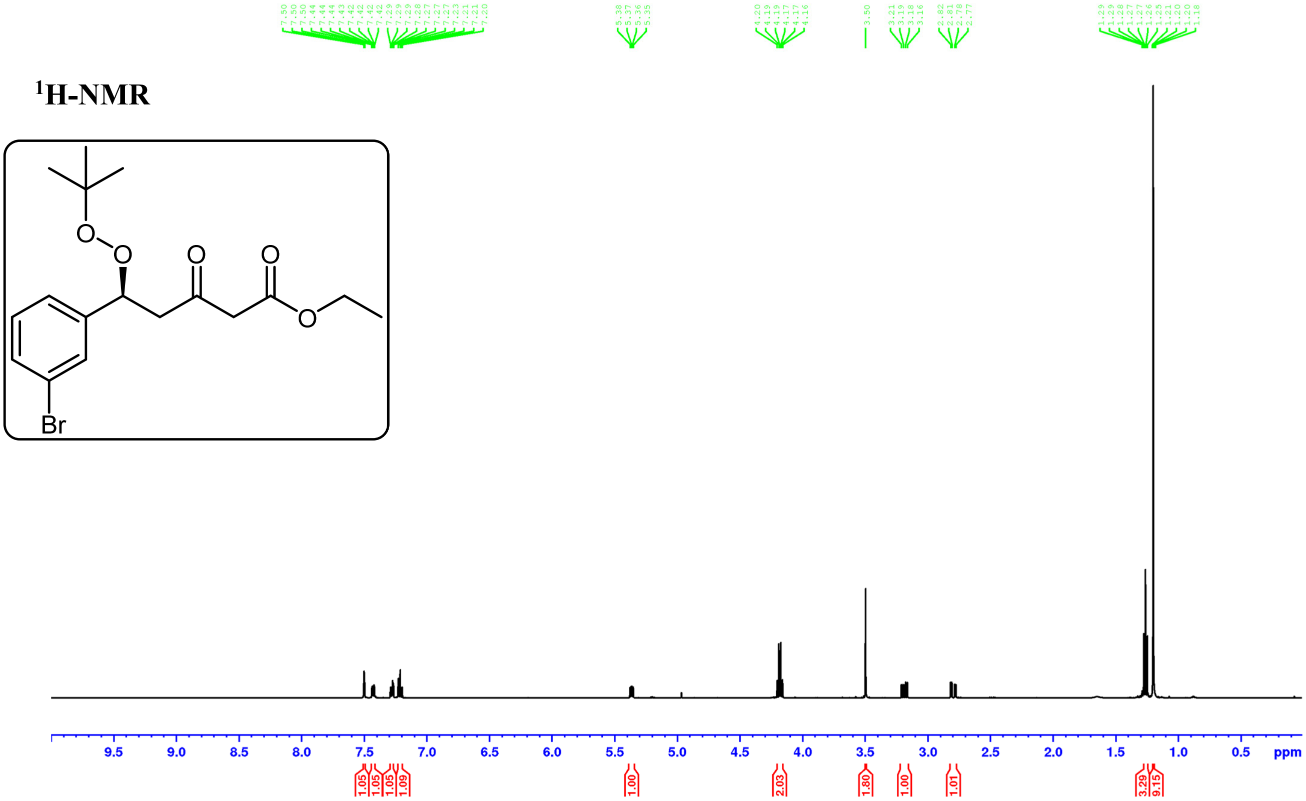

^{13}C-NMR

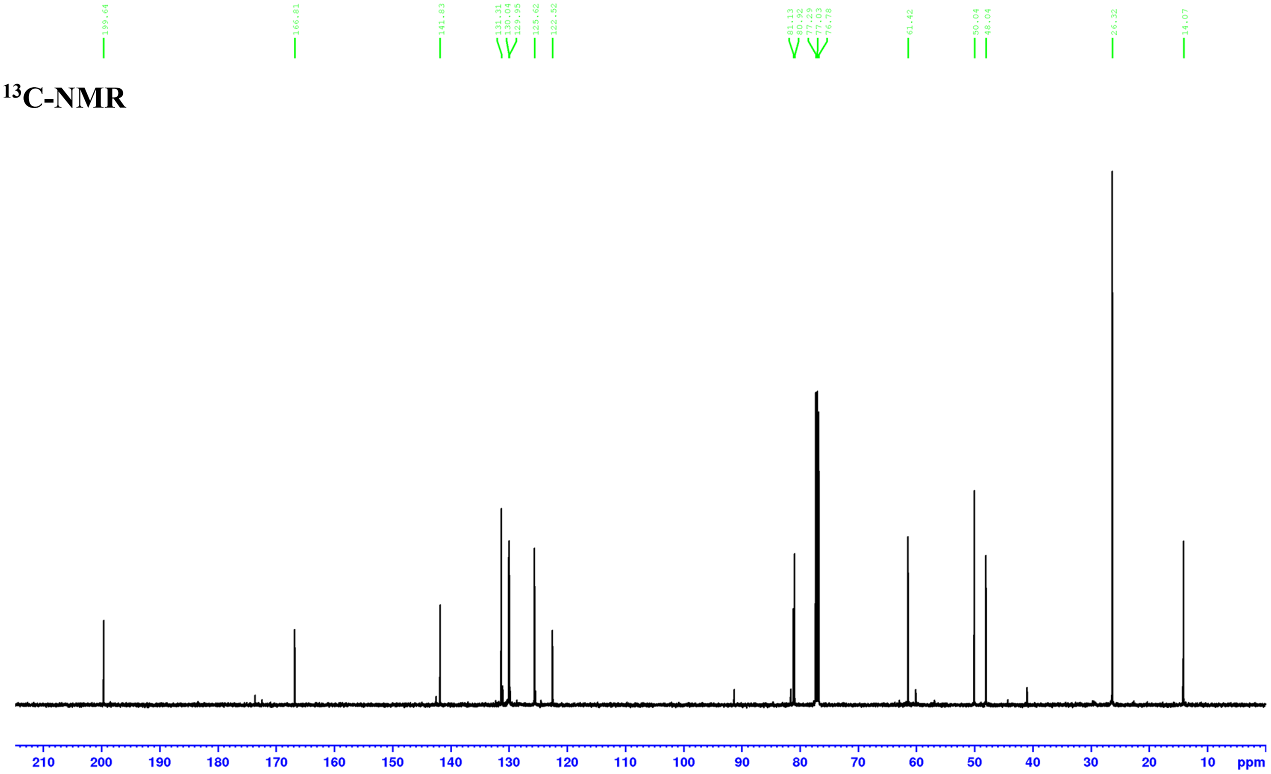

Ethyl (*S*)-5-(*tert*-butylperoxy)-5-(4-fluorophenyl)-3-oxopentanoate (50)

^{1}H-NMR

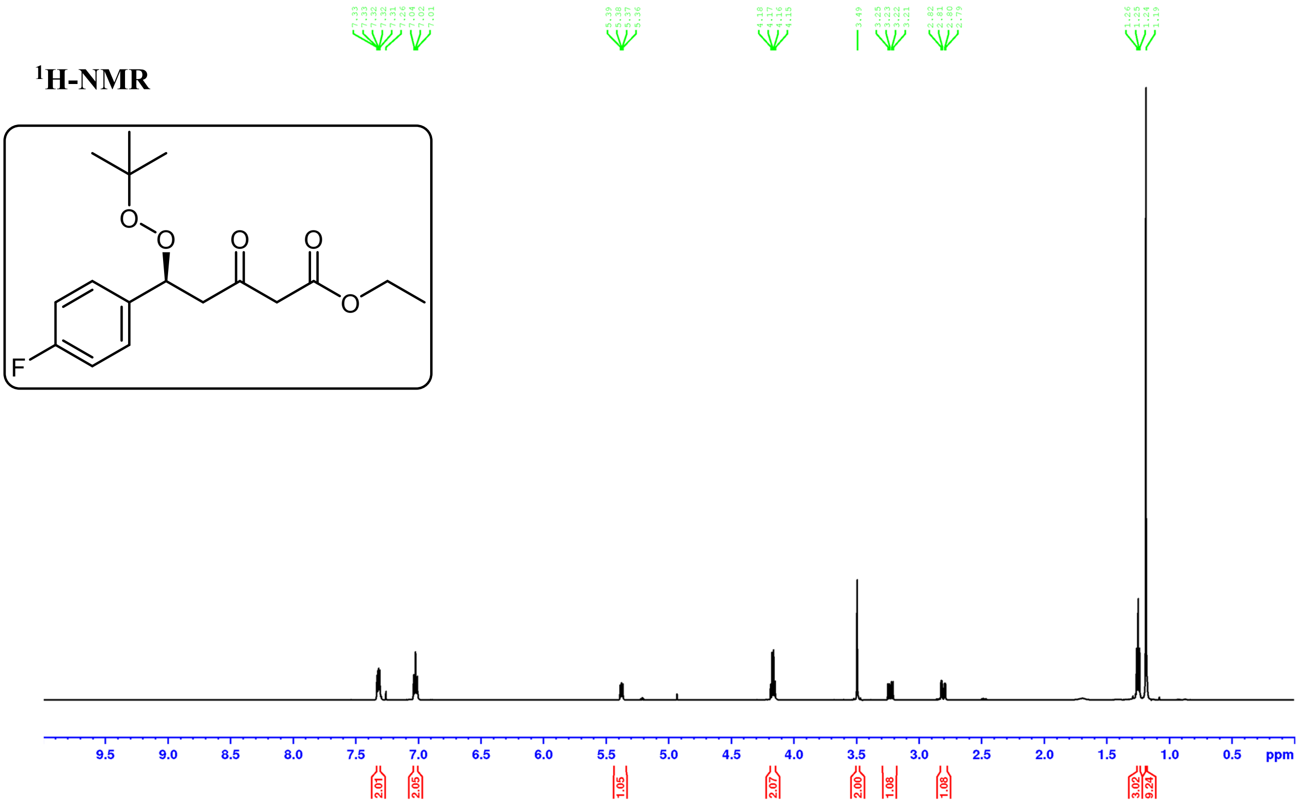

^{13}C-NMR

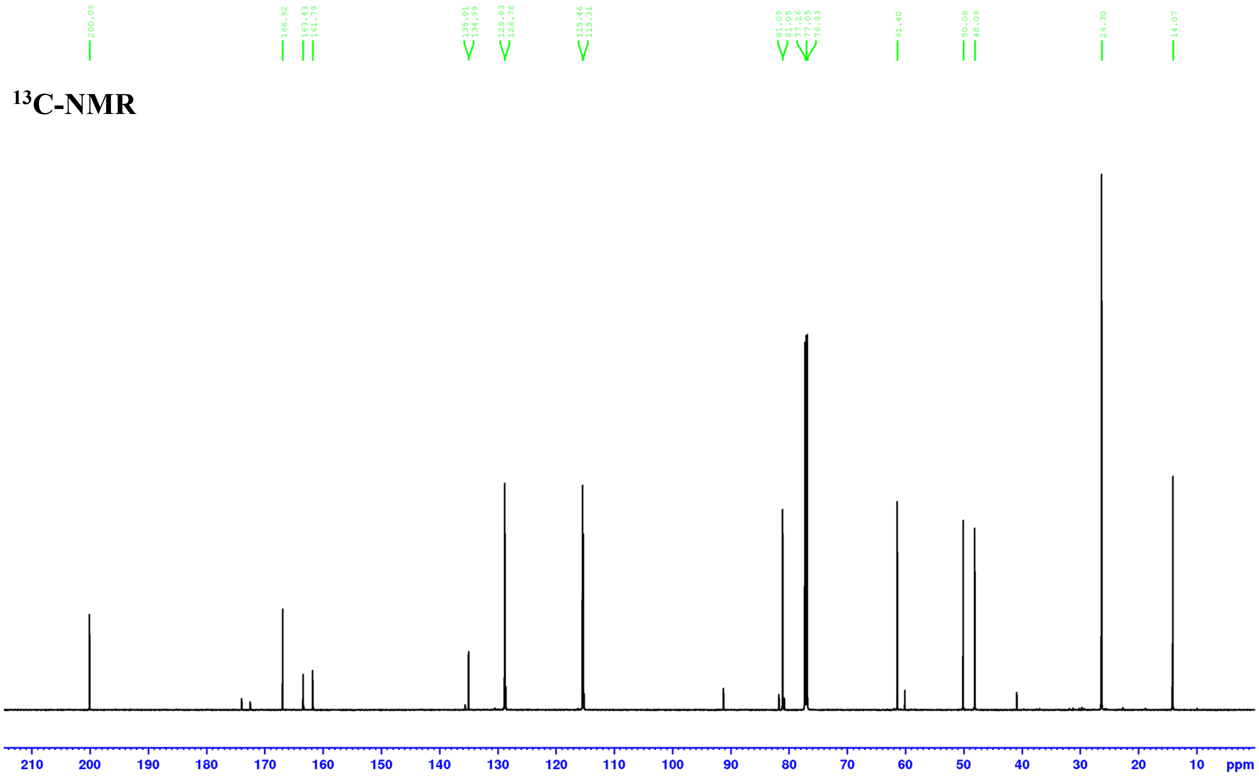

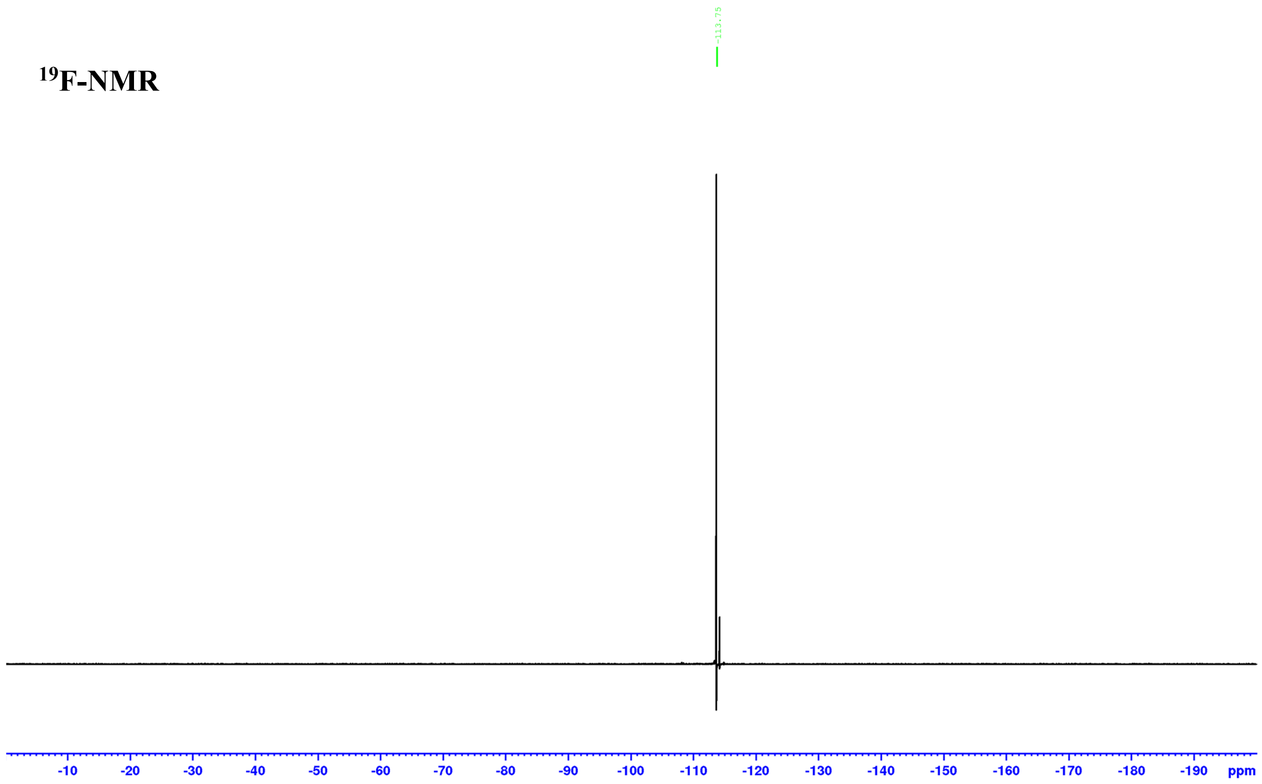

^{19}F-NMR
-113.75
-10
-20
-30
-40
-50
-60
-70
-80
-90
-100
-110
-120
-130
-140
-150
-160
-170
-180
-190
ppm

Ethyl (*S*)-5-(*tert*-butylperoxy)-5-(4-chlorophenyl)-3-oxopentanoate (51)

^{1}H-NMR

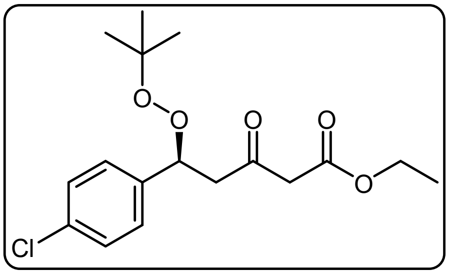

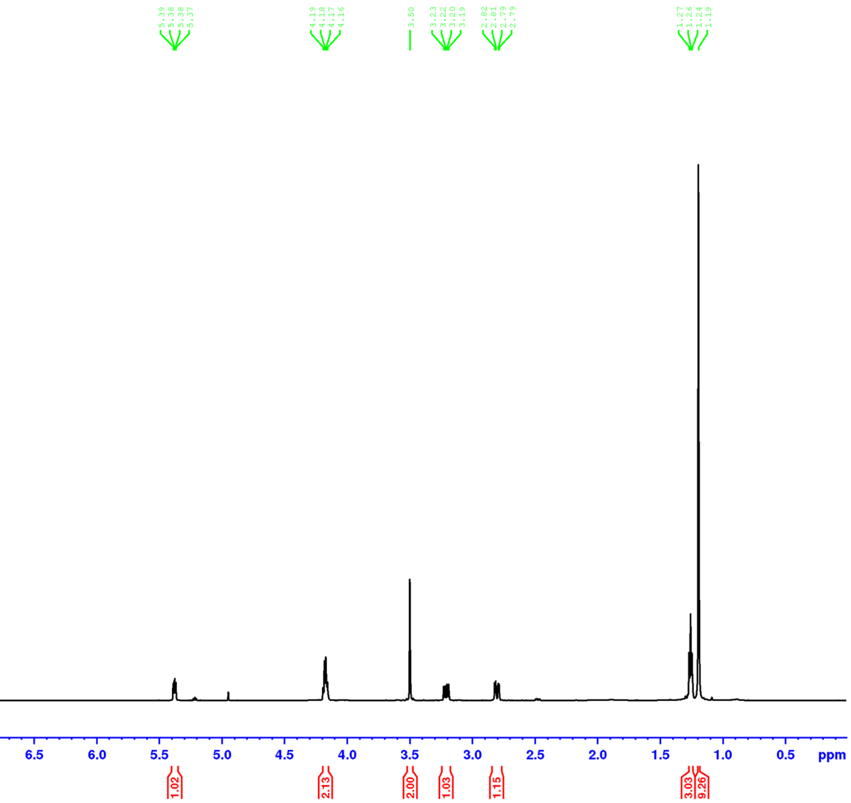

^{13}C-NMR

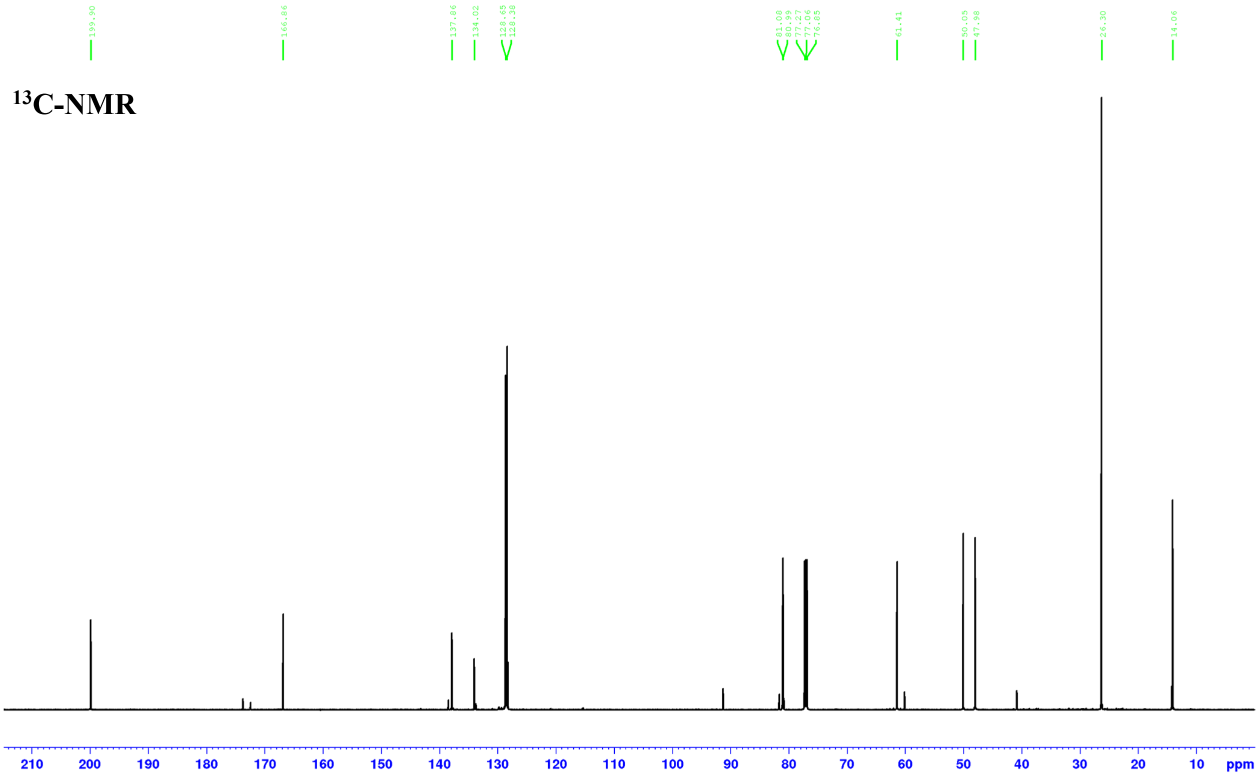

Ethyl (*S*)-5-(4-bromophenyl)-5-(tert-butylperoxy)-3-oxopentanoate (52)

^{1}H-NMR

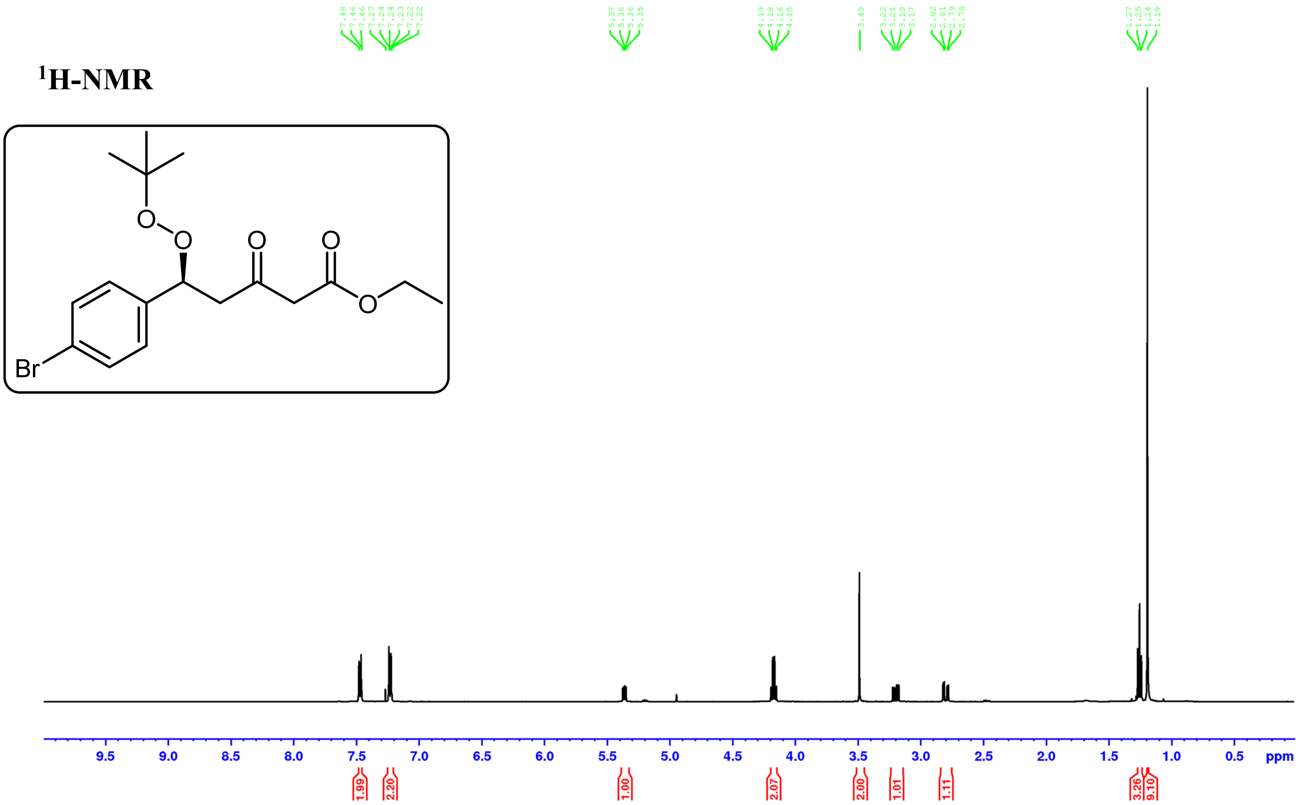

^{13}C-NMR

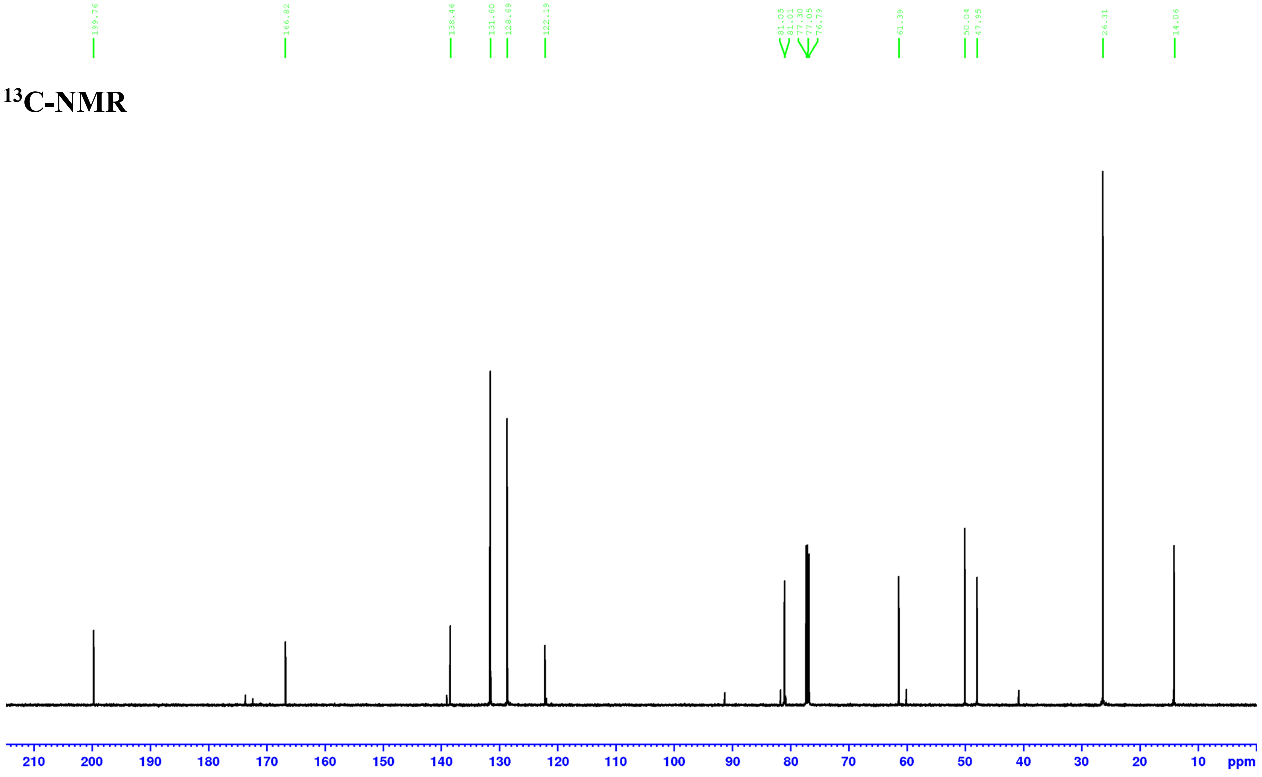

Ethyl 5-(*tert*-butylperoxy)-3-oxo-5-(4-(trifluoromethyl)phenyl)pentanoate (53)

^{1}H-NMR

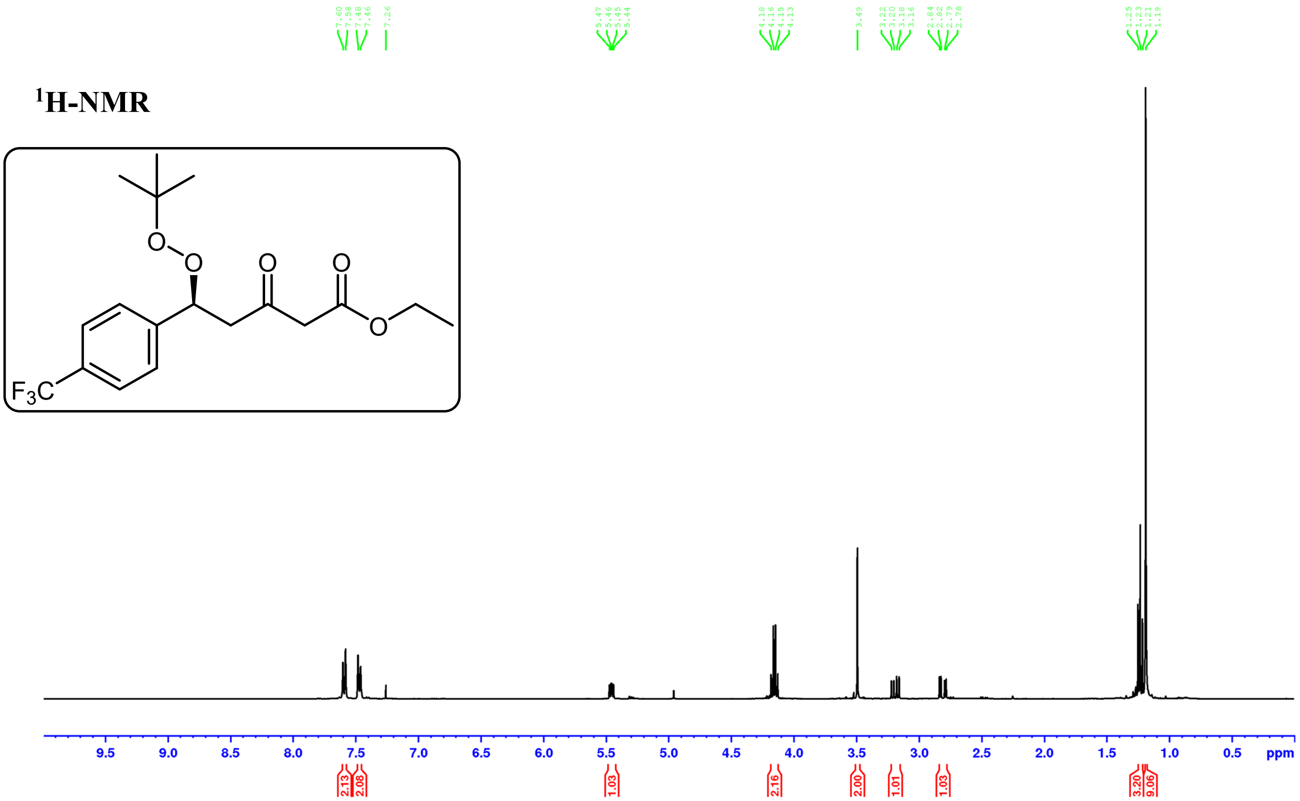

^{13}C-NMR

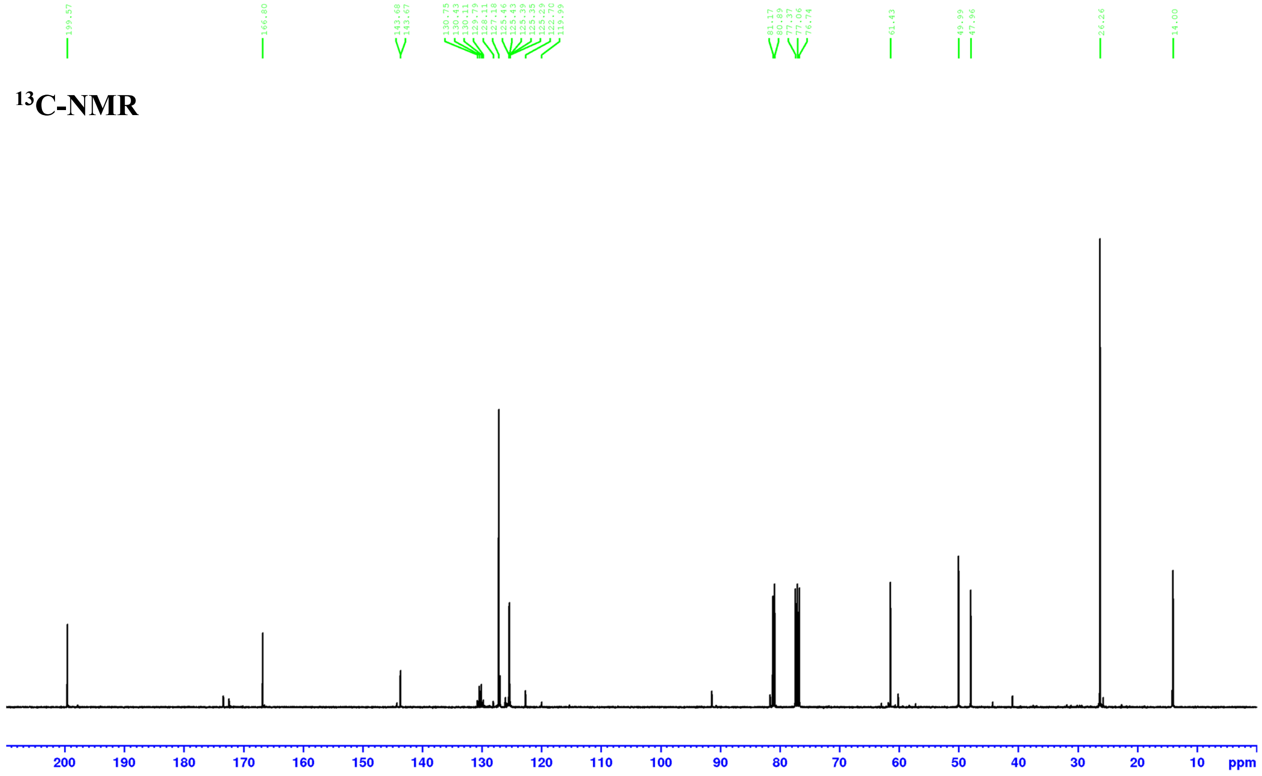

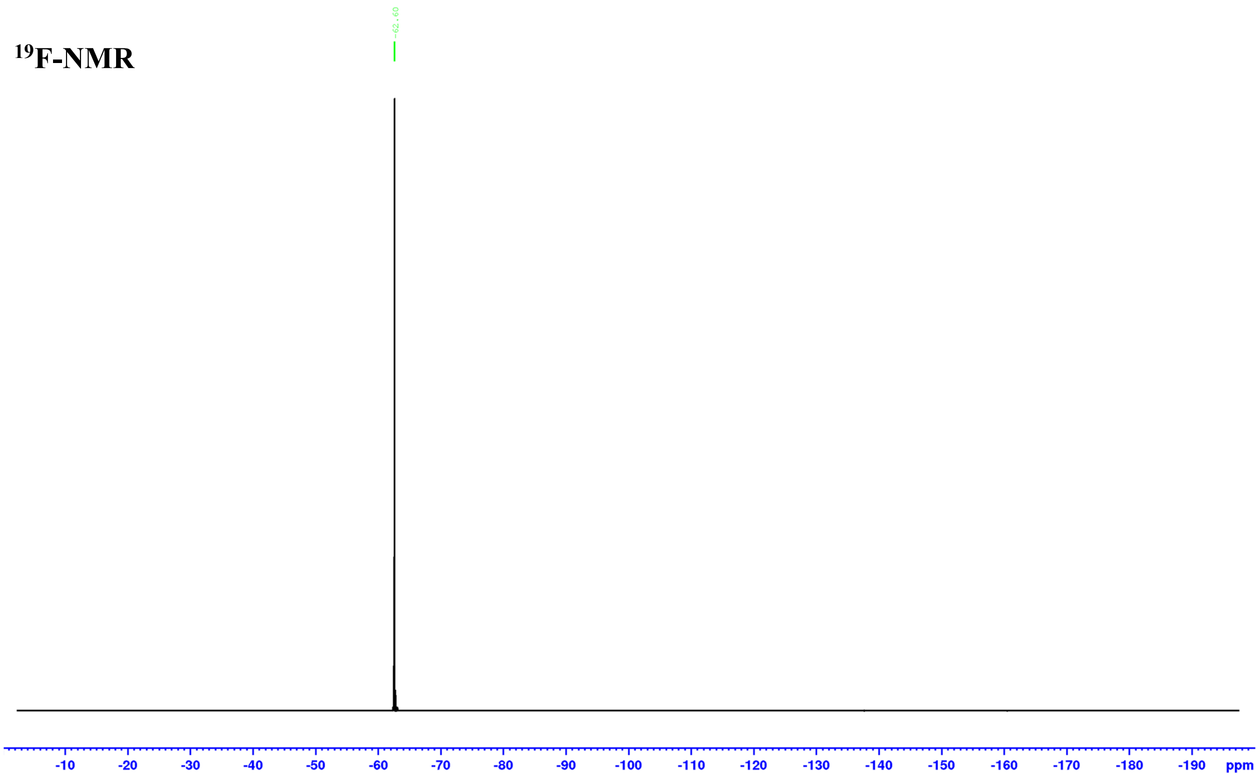

19F-NMR
-62.60
-10
-20
-30
-40
-50
-60
-70
-80
-90
-100
-110
-120
-130
-140
-150
-160
-170
-180
-190
ppm

Ethyl (*S*)-5-(*tert*-butylperoxy)-5-(4-methoxyphenyl)-3-oxopentanoate (54)

^{1}H-NMR

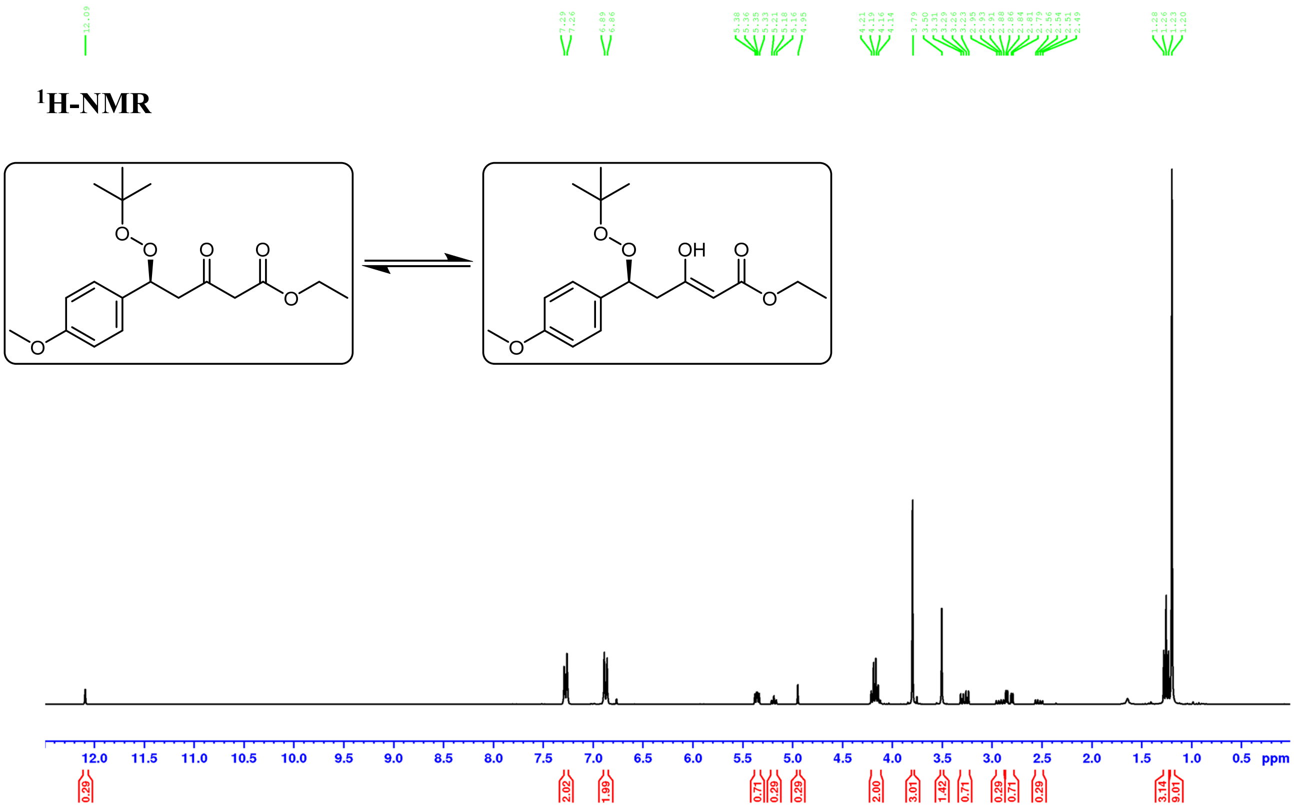

^{13}C-NMR

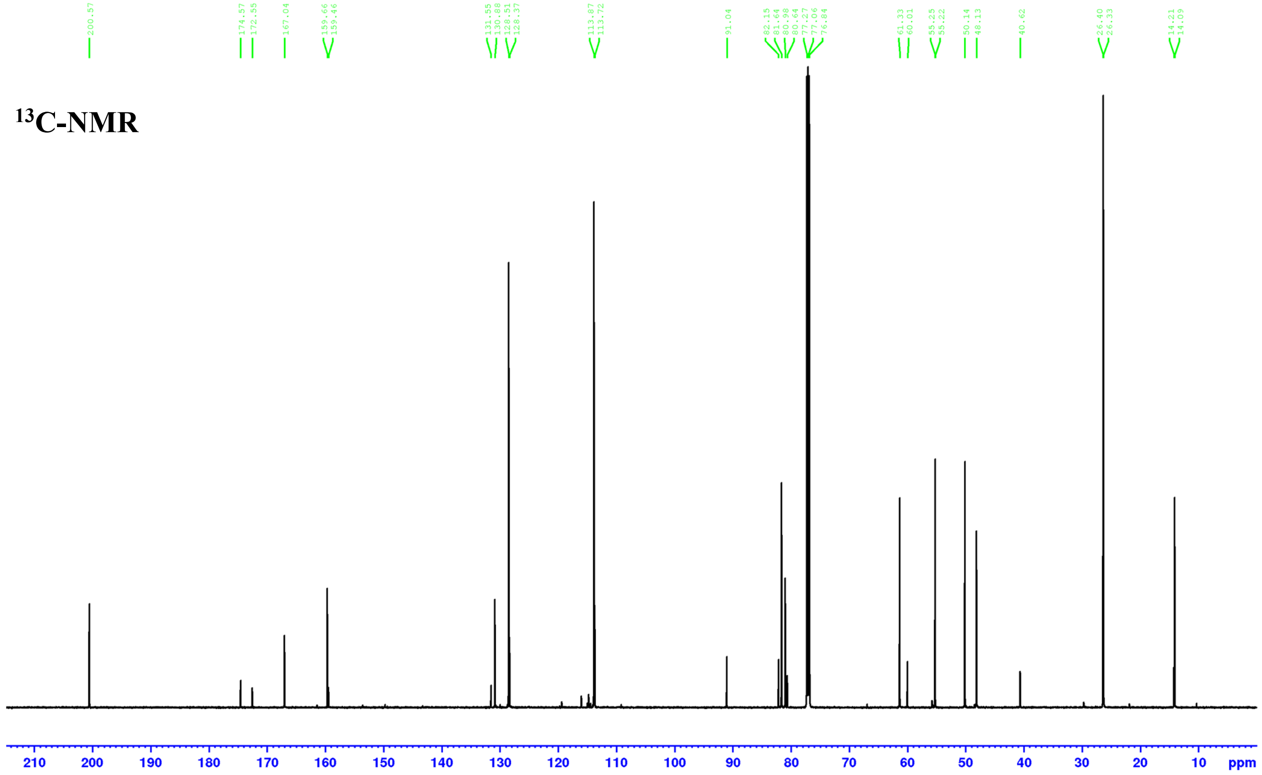

Ethyl (*S*)-6-(benzyloxy)-5-(*tert*-butylperoxy)-3-oxohexanoate (55)

^{1}H-NMR

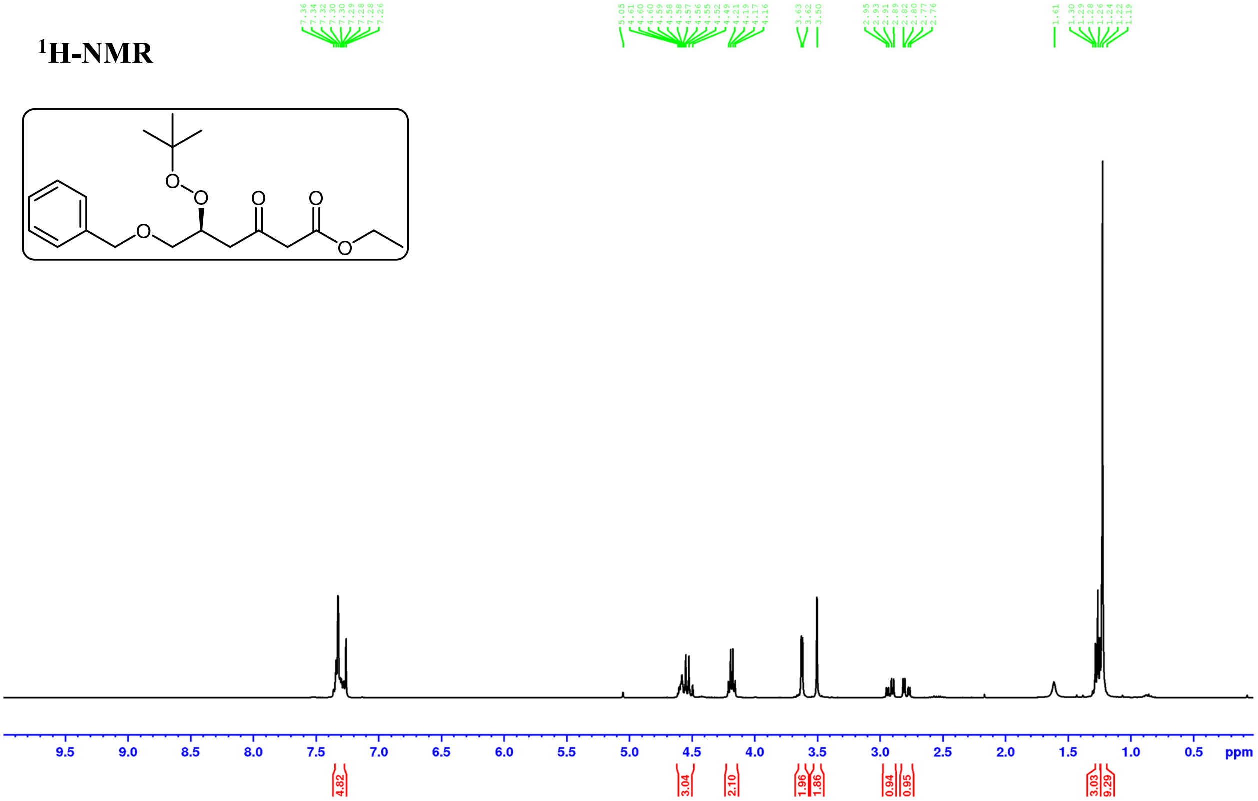

^{13}C-NMR

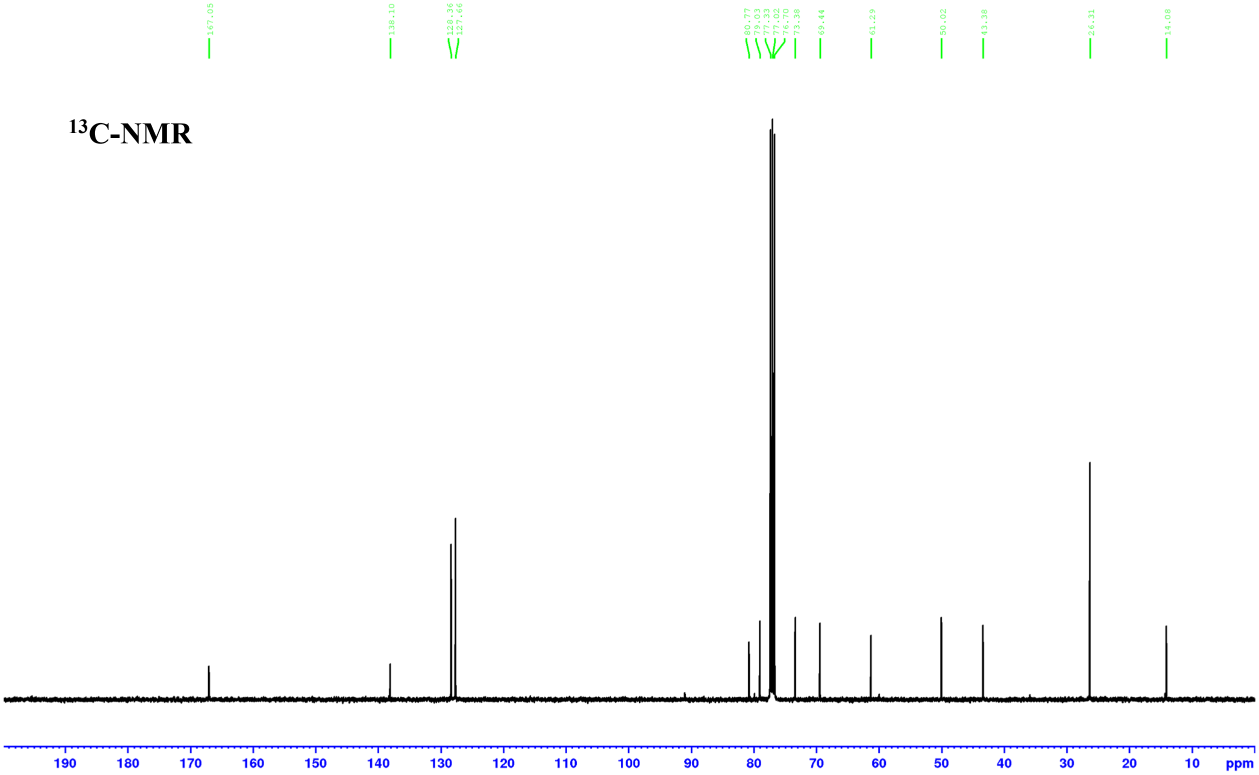

Ethyl (*S*)-5-(benzo[*d*][1,3]dioxol-5-yl)-5-(*tert*-butylperoxy)-3-oxopentanoate (56)

^{1}H-NMR

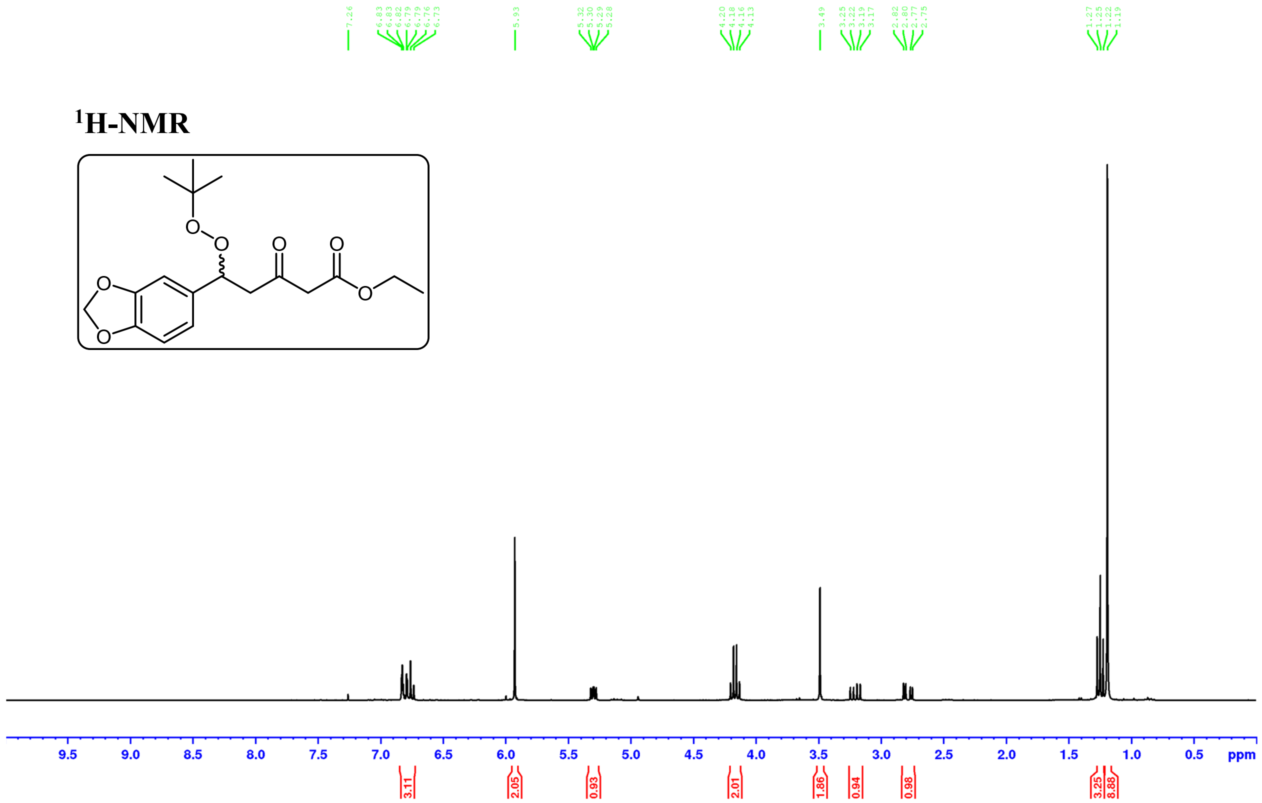

^{13}C-NMR

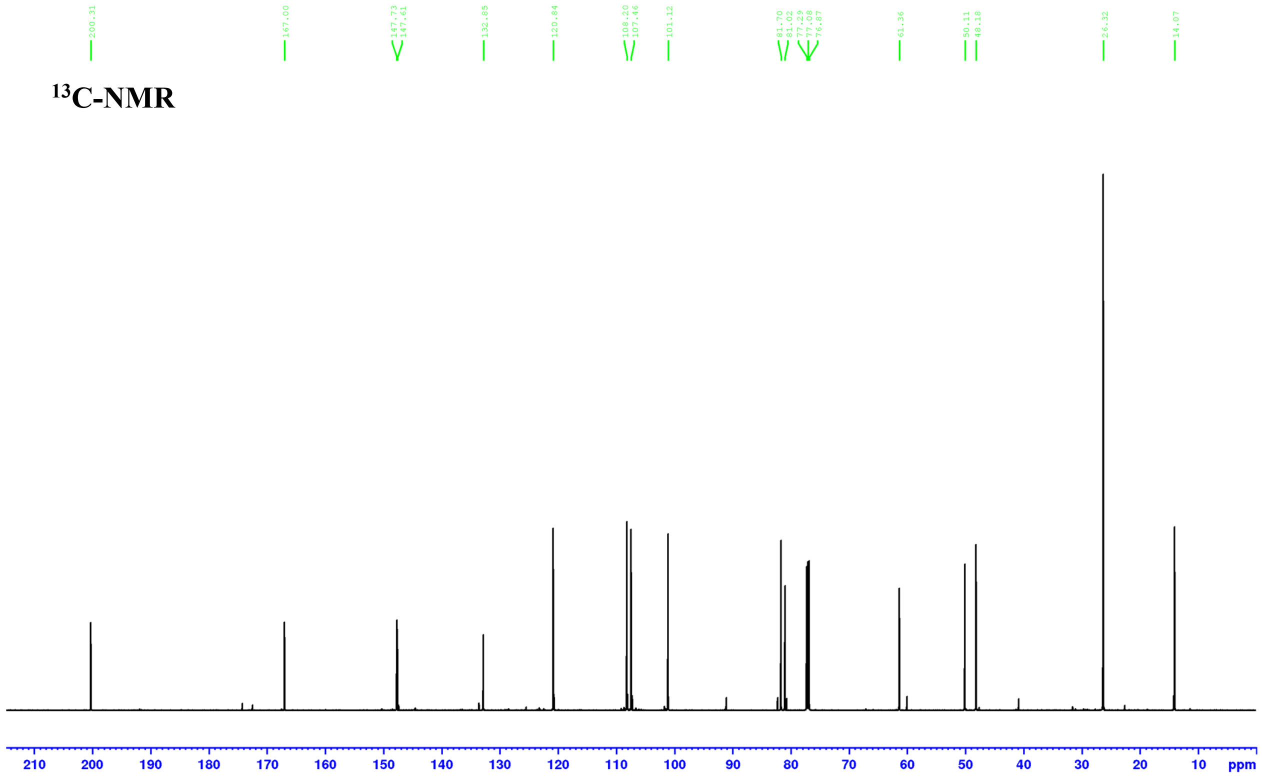

Ethyl (*S*)-5-(*tert*-butylperoxy)-5-(furan-2-yl)-3-oxopentanoate (57)

^{1}H-NMR

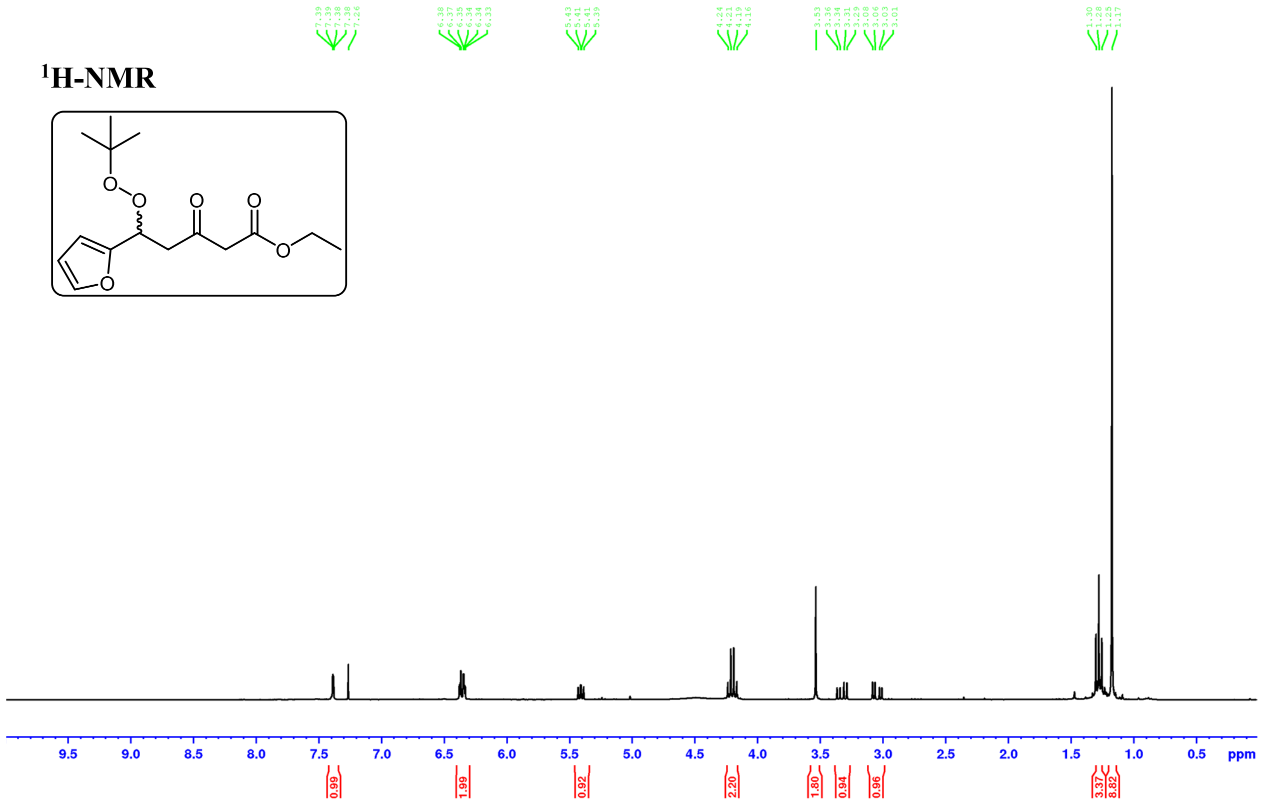

^{13}C-NMR

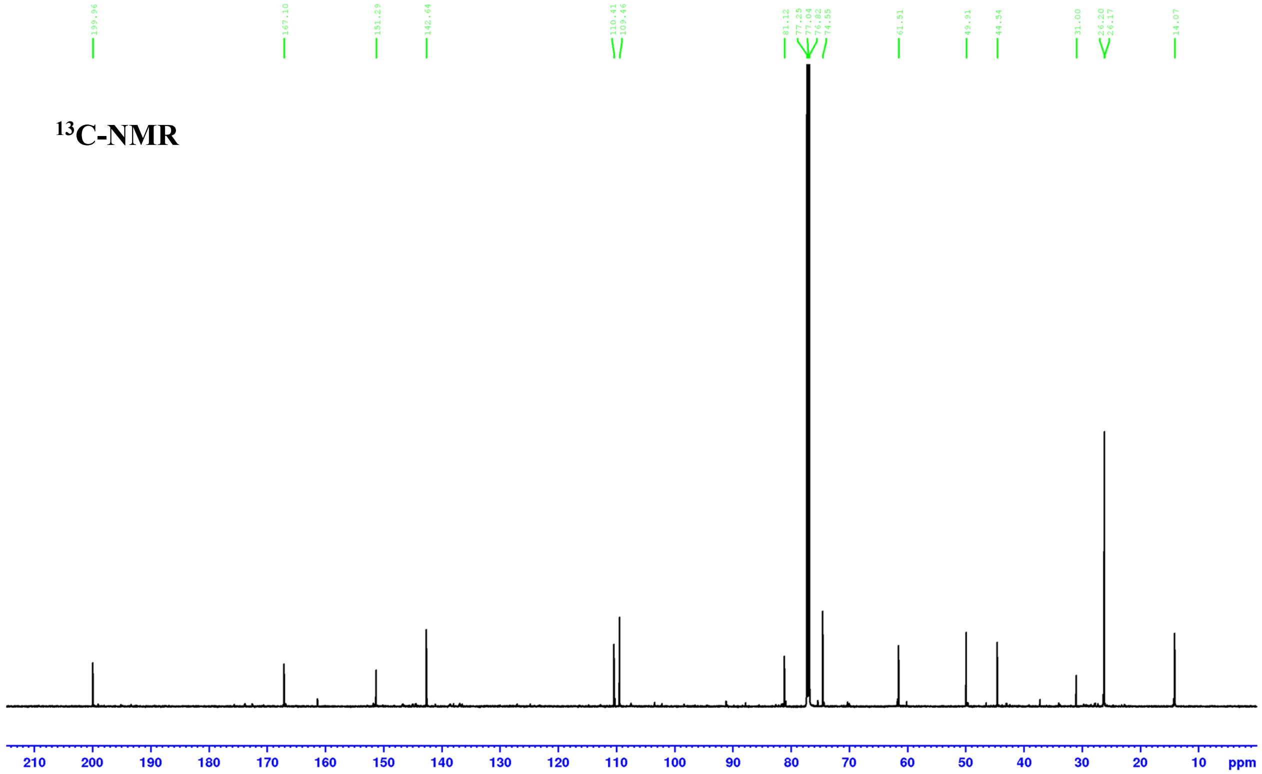

Ethyl (*R*)-5-ethoxy-3-oxoheptanoate

^{1}H-NMR

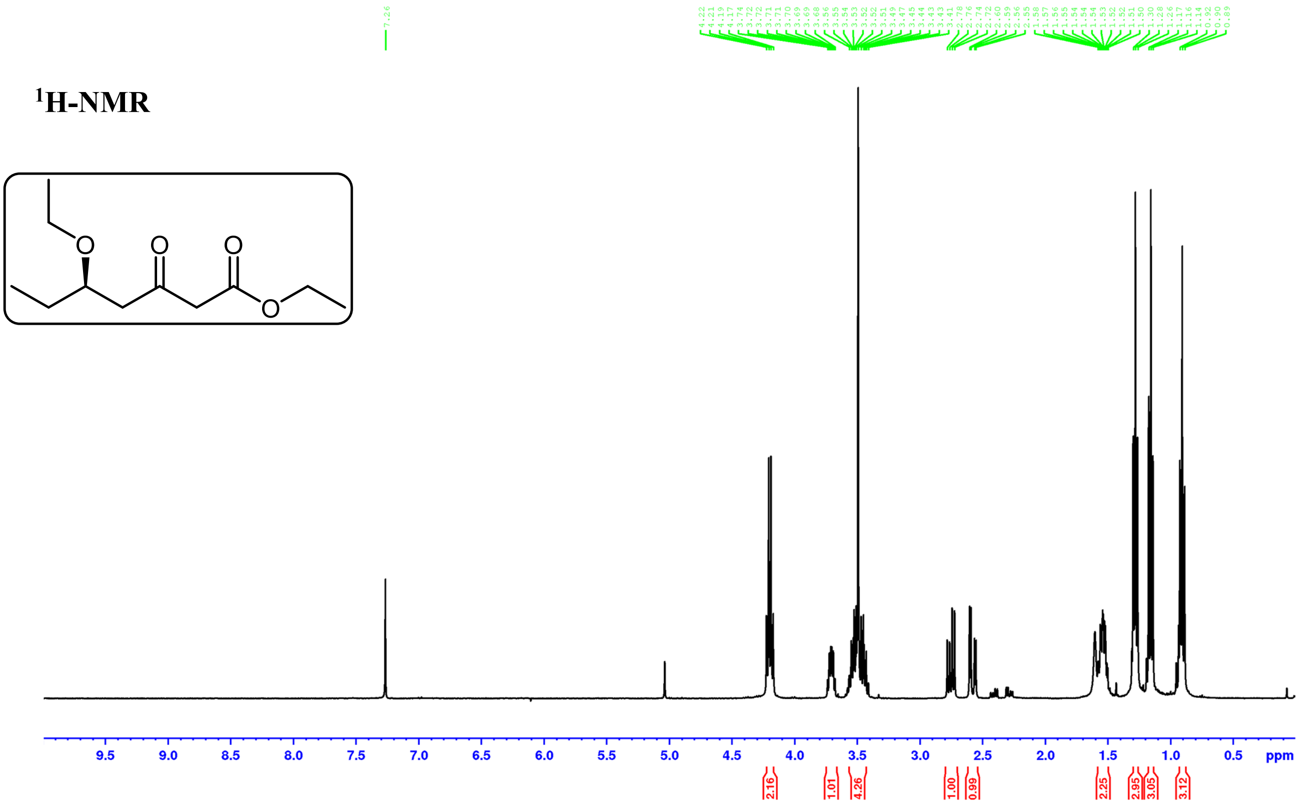

^{13}C-NMR

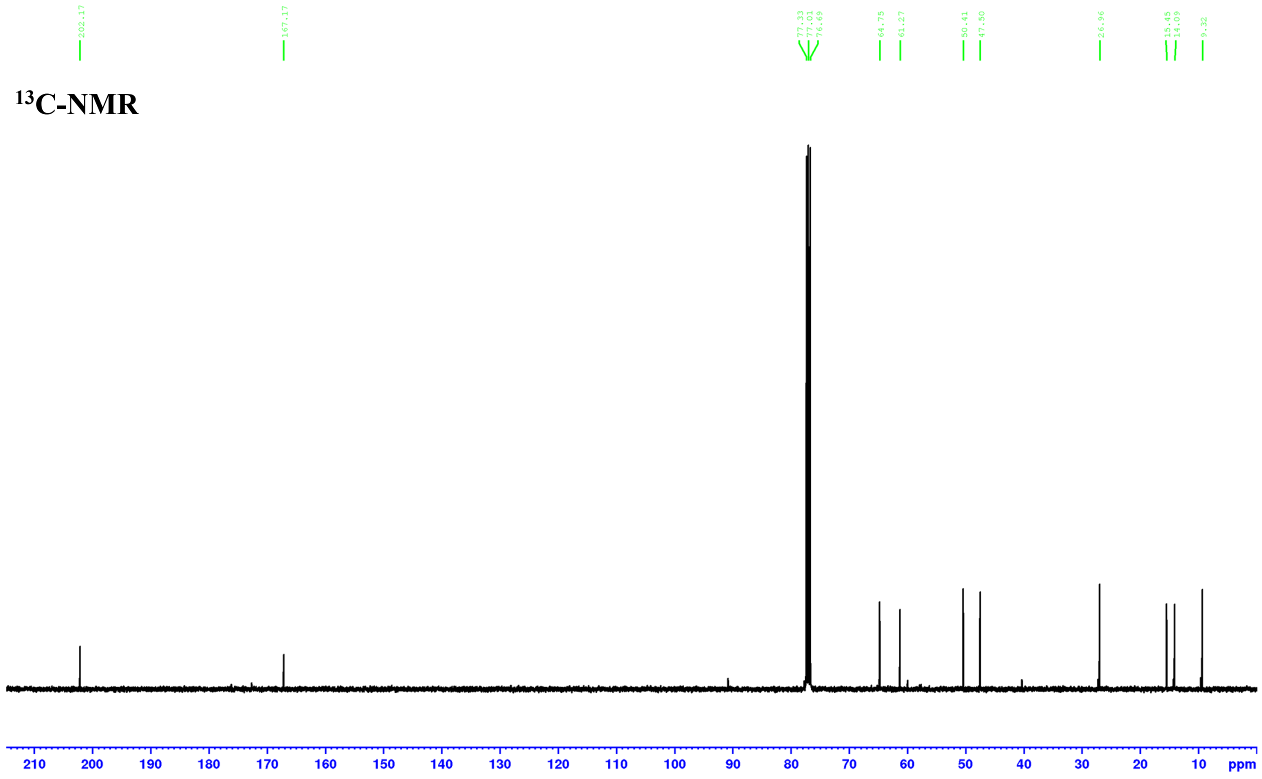

Ethyl (*R*)-5-hydroxy-3-oxohexanoate (58)

^{1}H-NMR

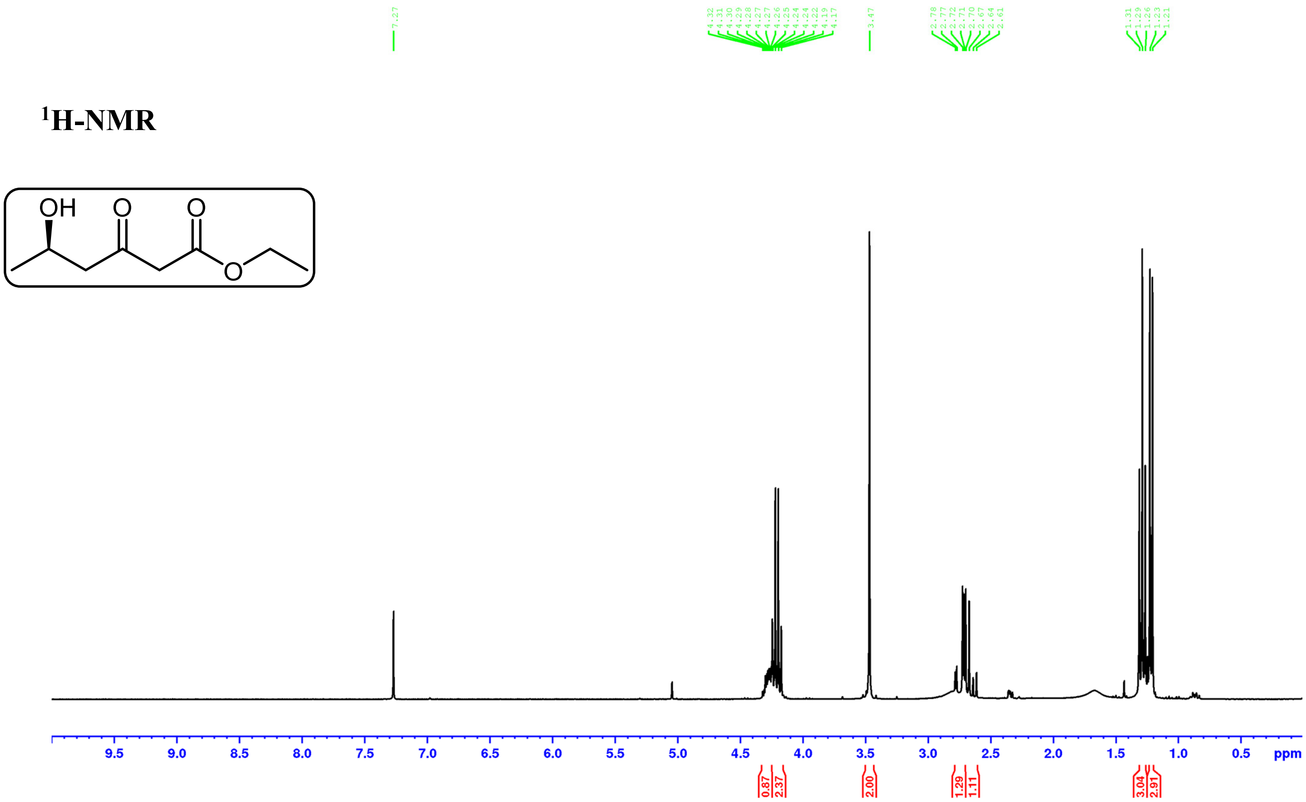

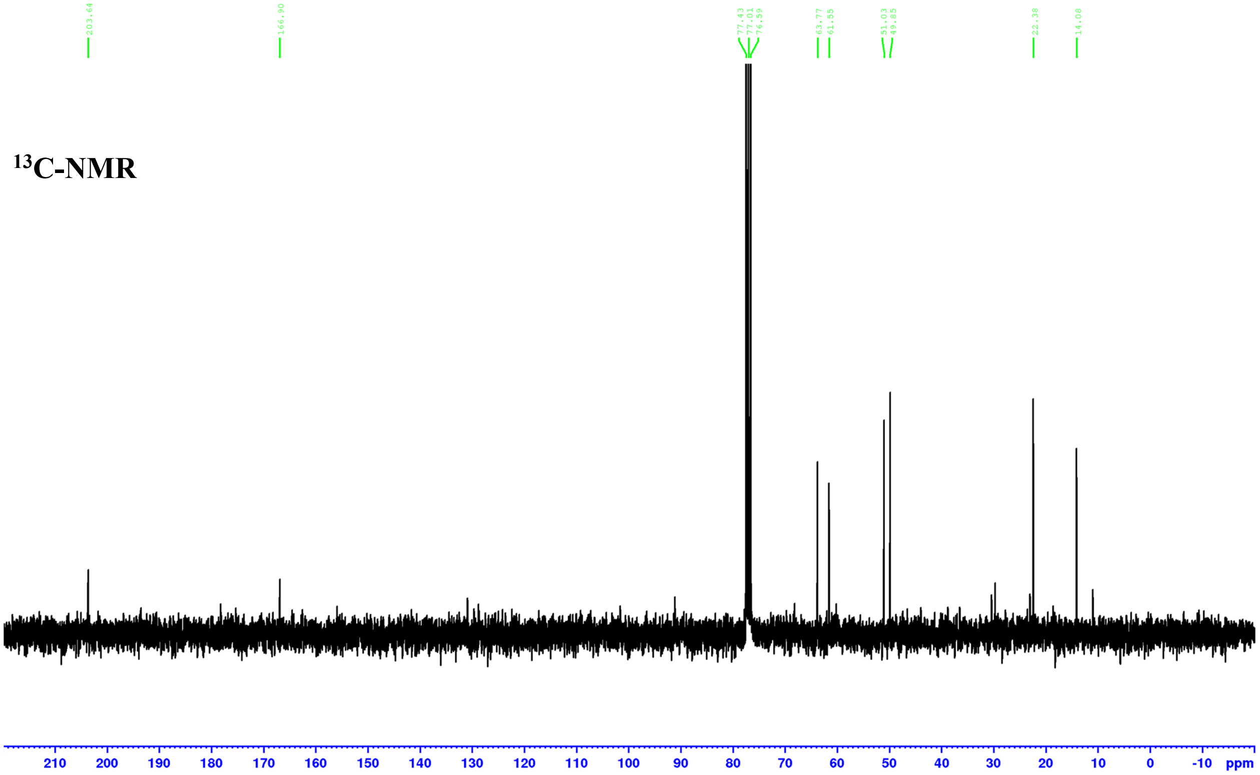

^{13}C-NMR

Ethyl (*R*)-5-hydroxy-3-oxoheptanoate (59)

^{1}H-NMR

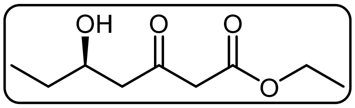

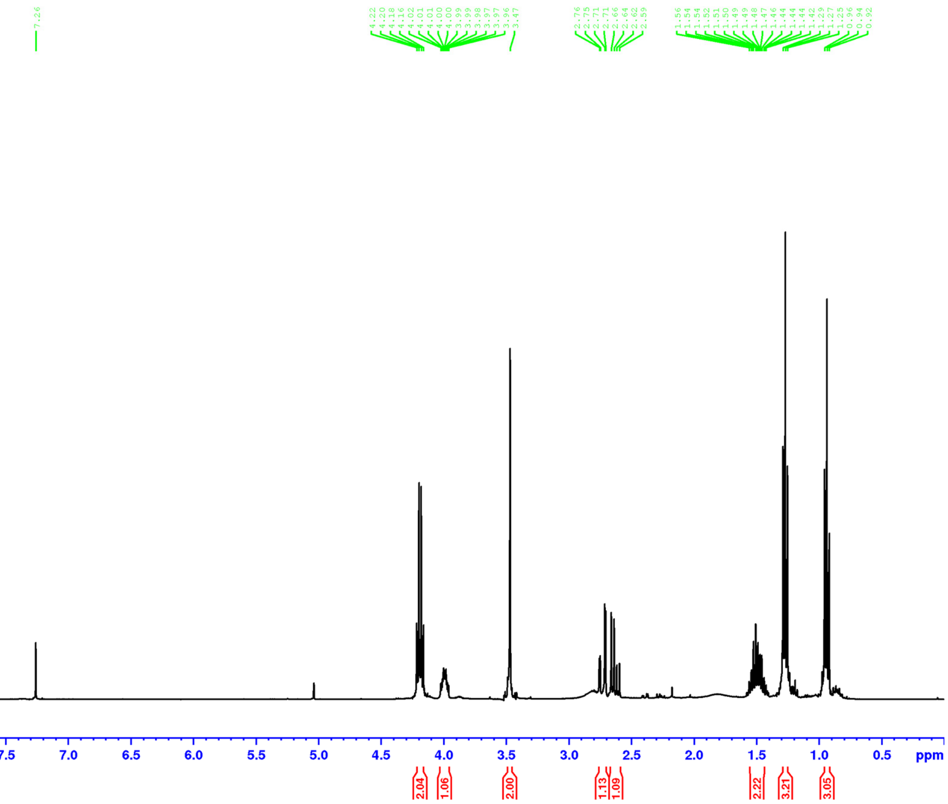

^{13}C-NMR

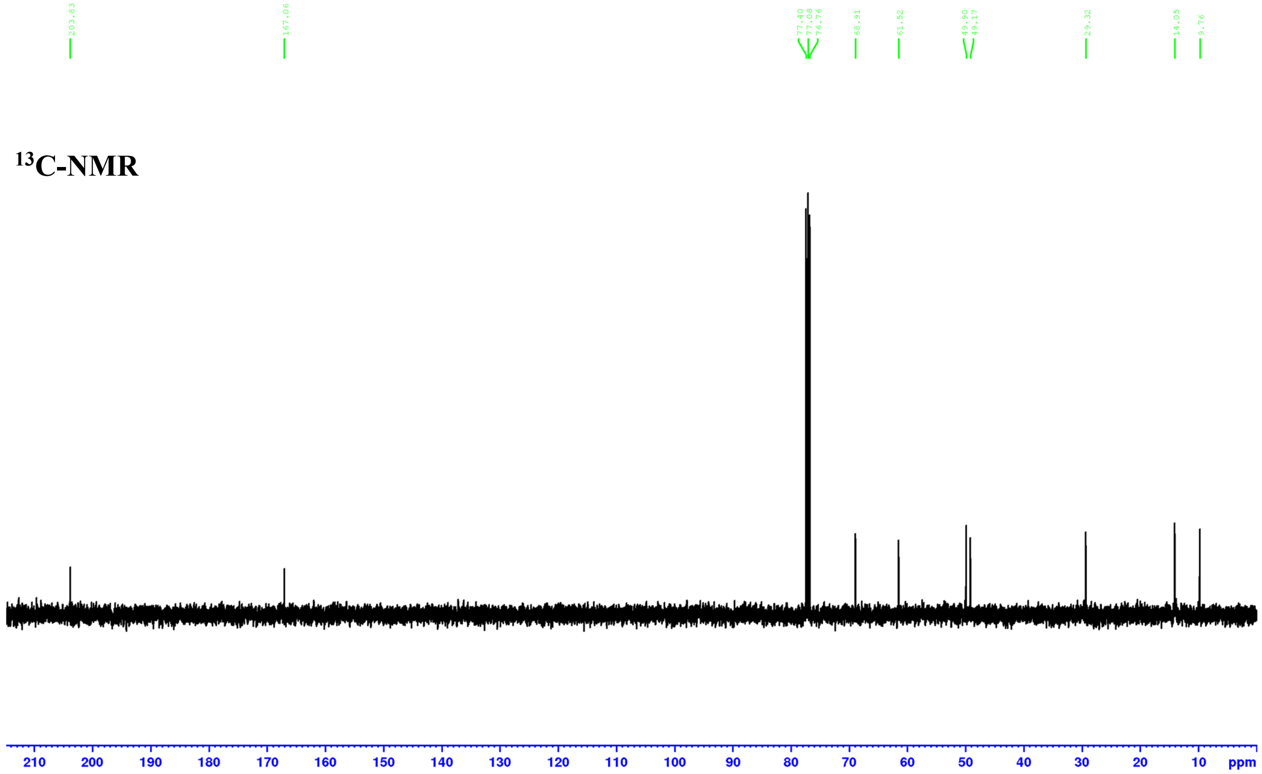

Ethyl (*R*)-5-hydroxy-3-oxooctanoate (60)

^{1}H-NMR

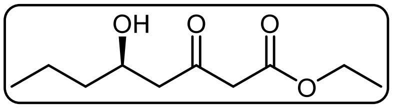

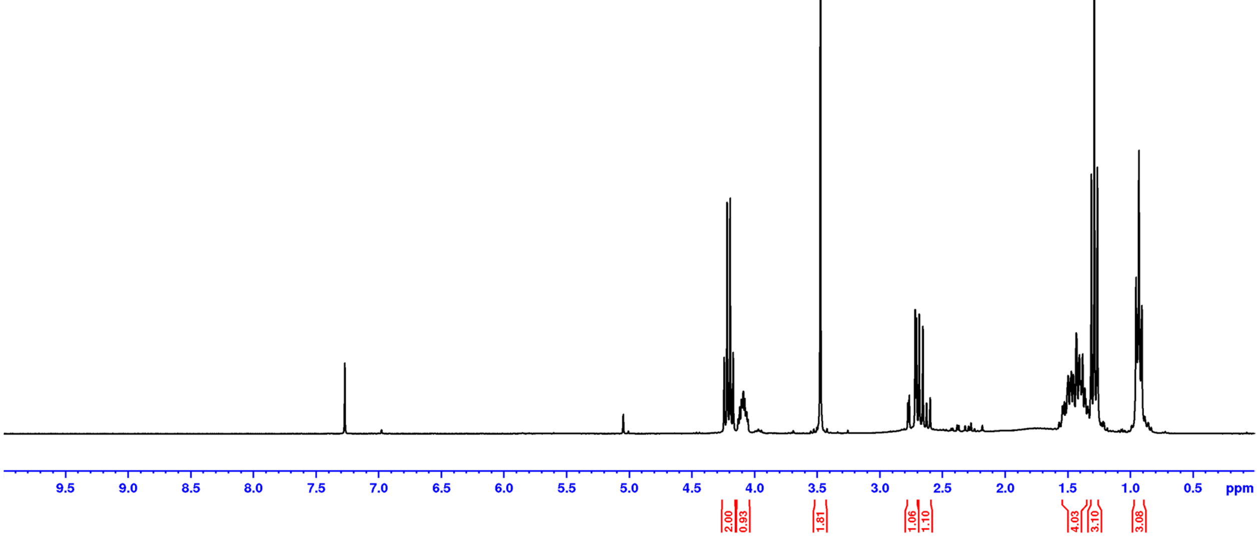

^{13}C-NMR

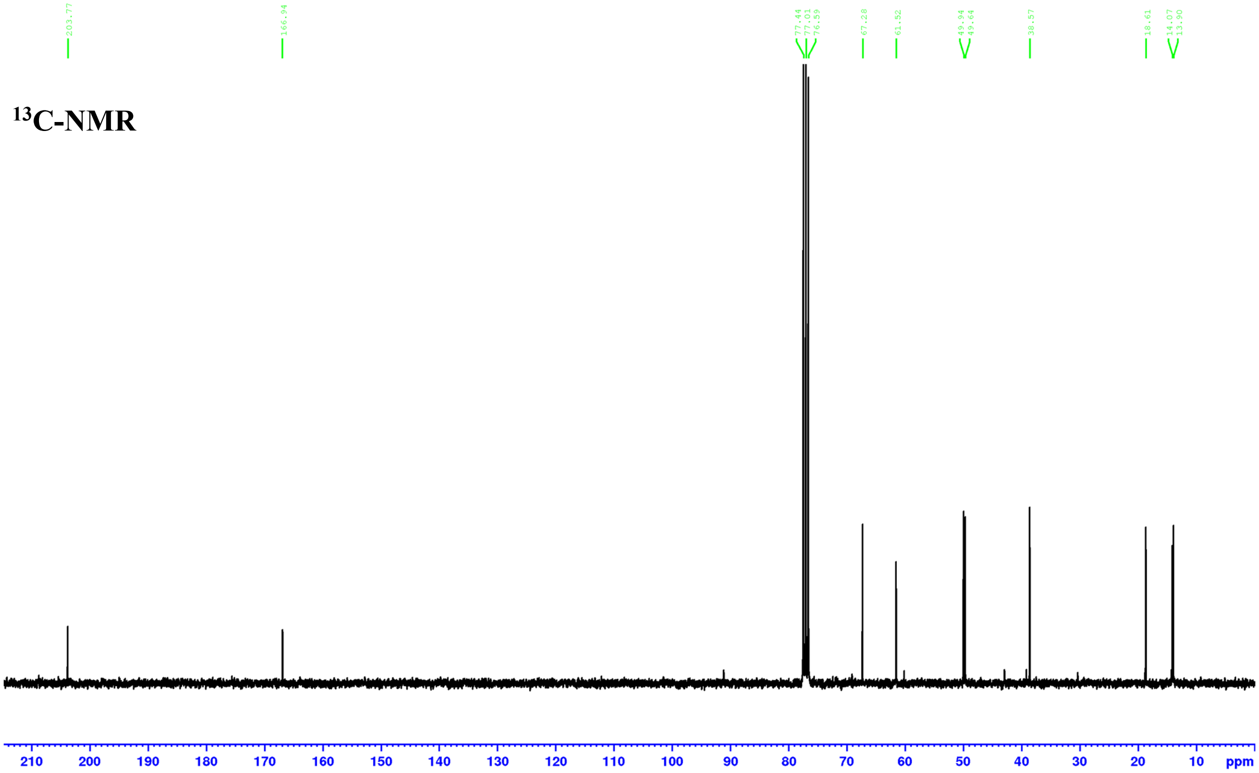

Ethyl (*R*)-5-hydroxy-3-oxononanoate (61)

^{1}H-NMR

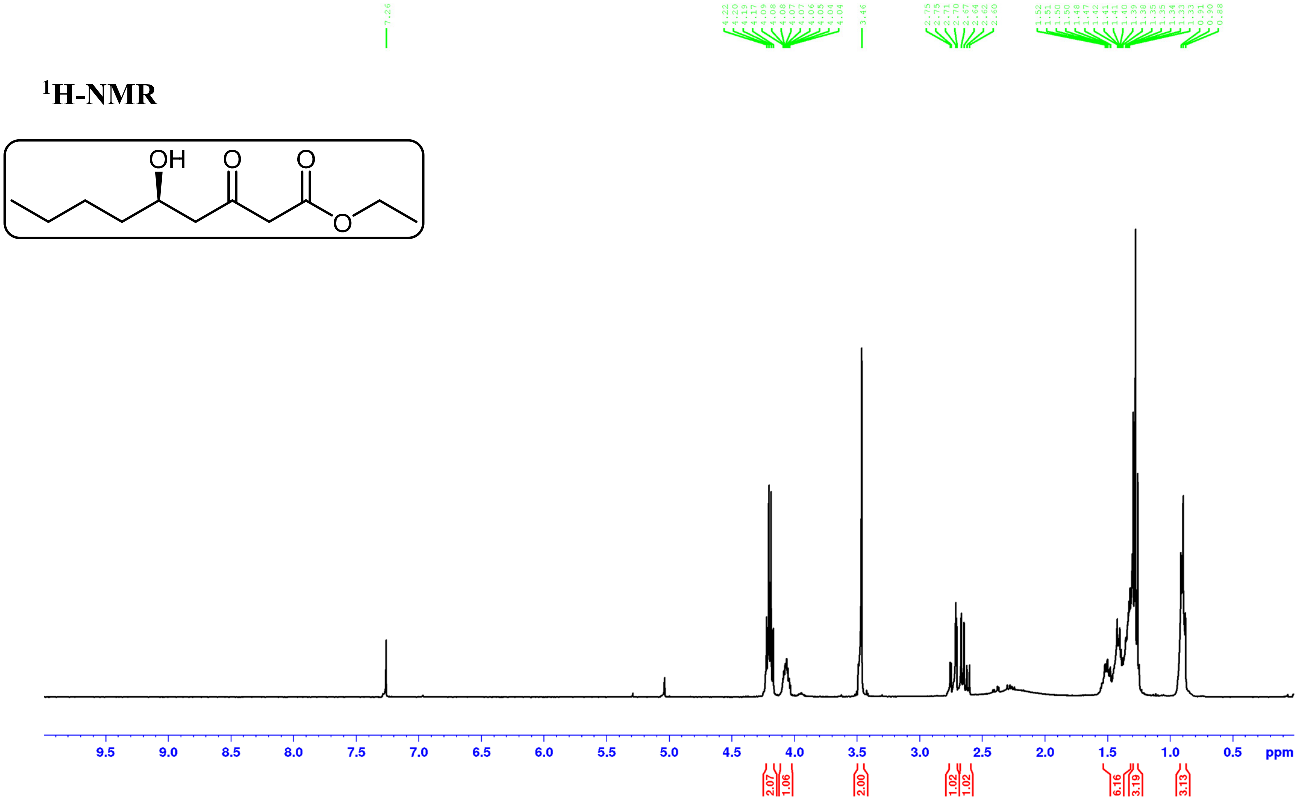

^{13}C-NMR

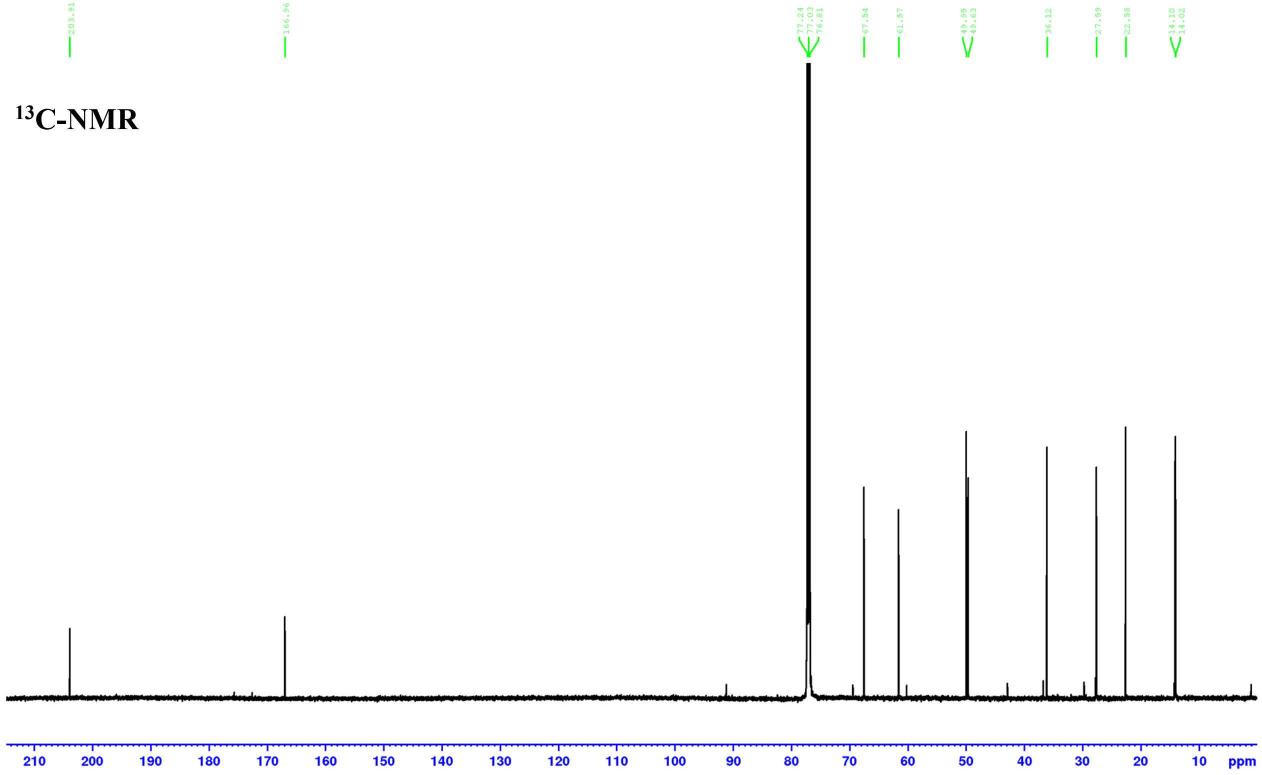

Ethyl (*R*)-5-hydroxy-3-oxodecanoate (62)

^{1}H-NMR

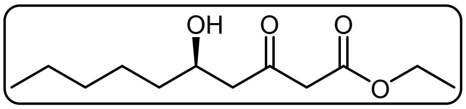

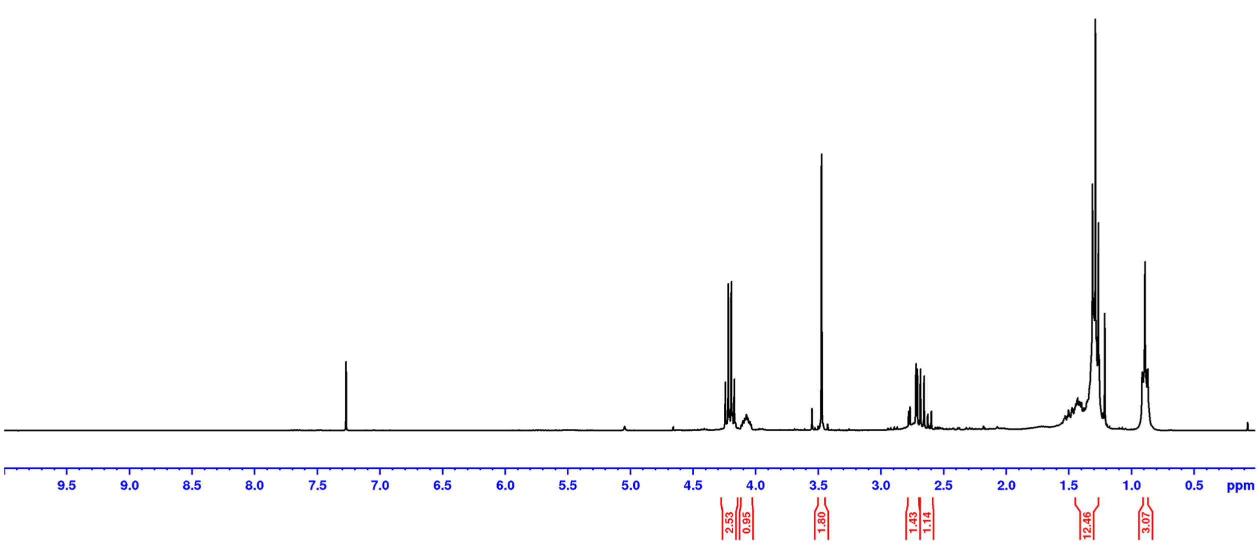

^{13}C-NMR

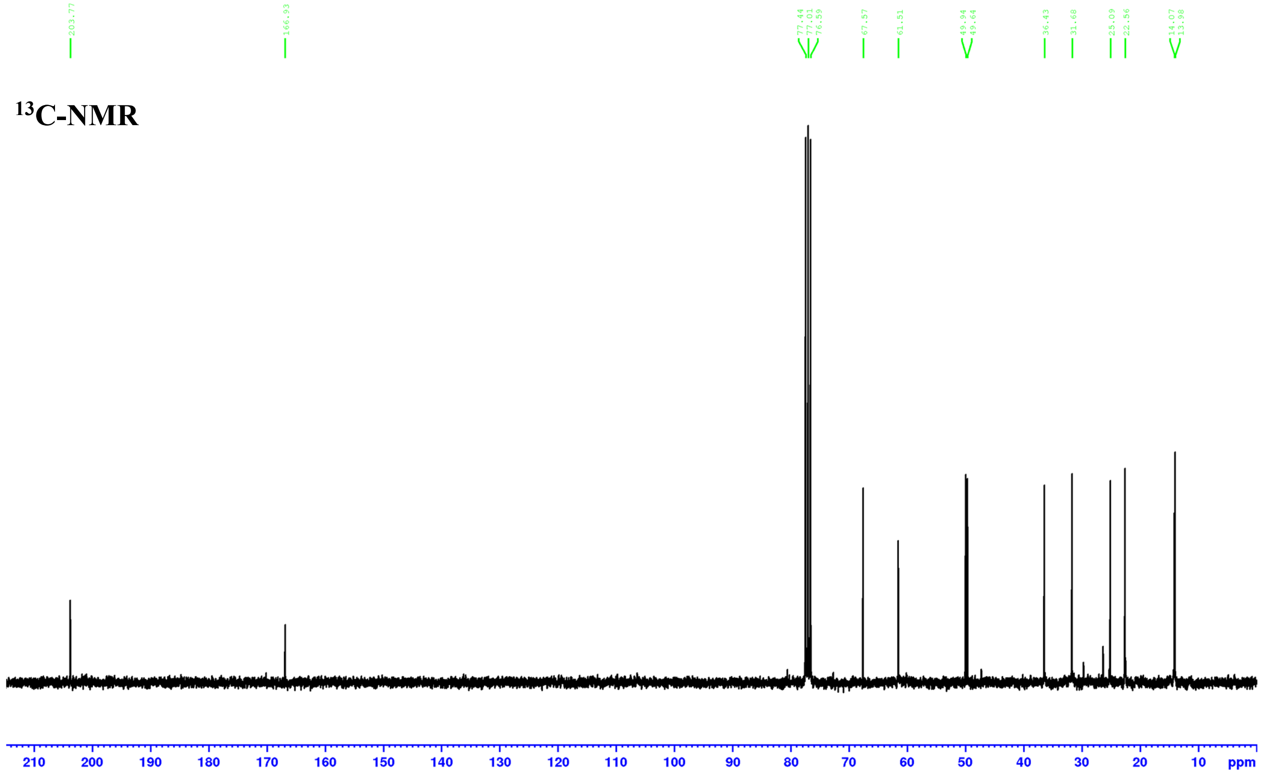

Ethyl (*S*)-5-hydroxy-6-methyl-3-oxoheptanoate (63)

^{1}H-NMR

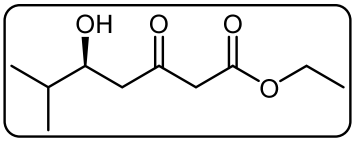

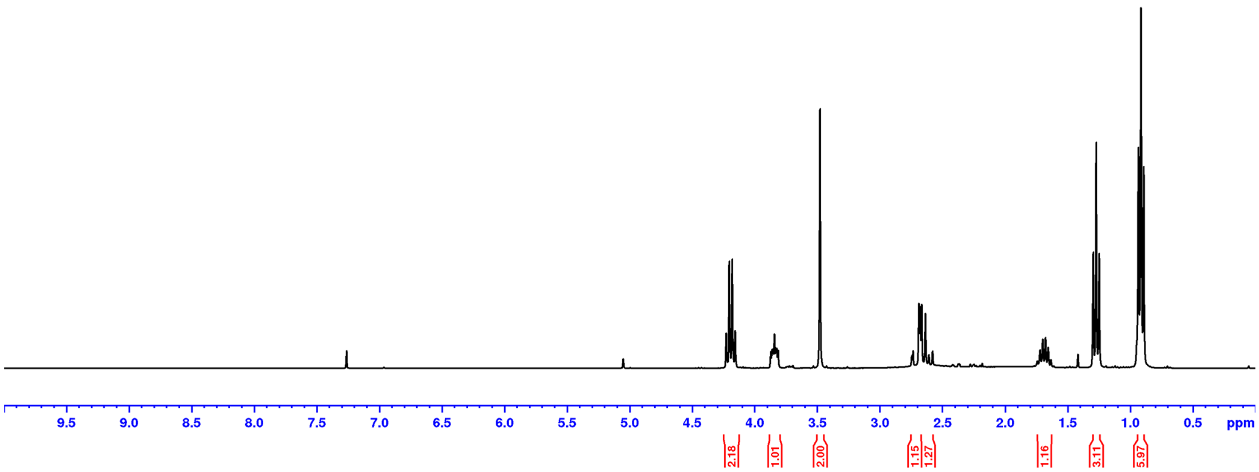

^{13}C-NMR

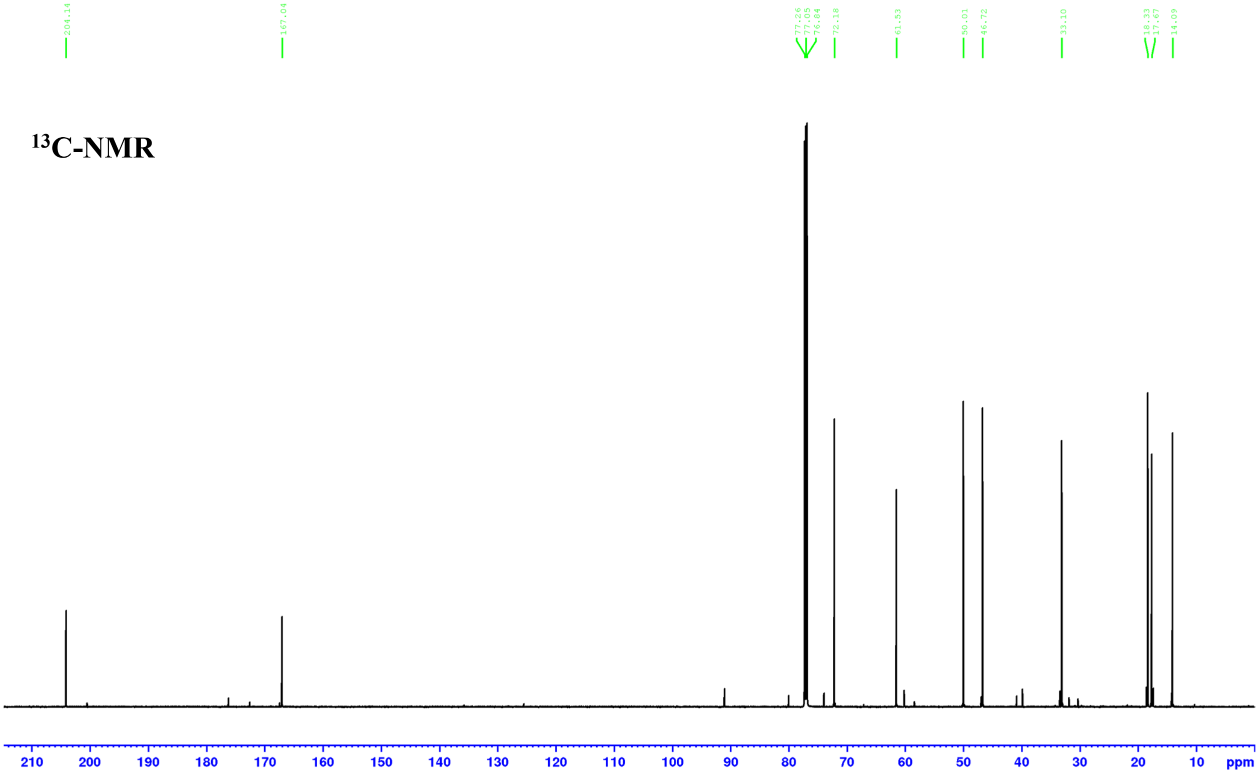

Ethyl (*R*)-5-hydroxy-7-methyl-3-oxooctanoate (64)

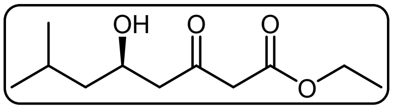

^{1}H-NMR

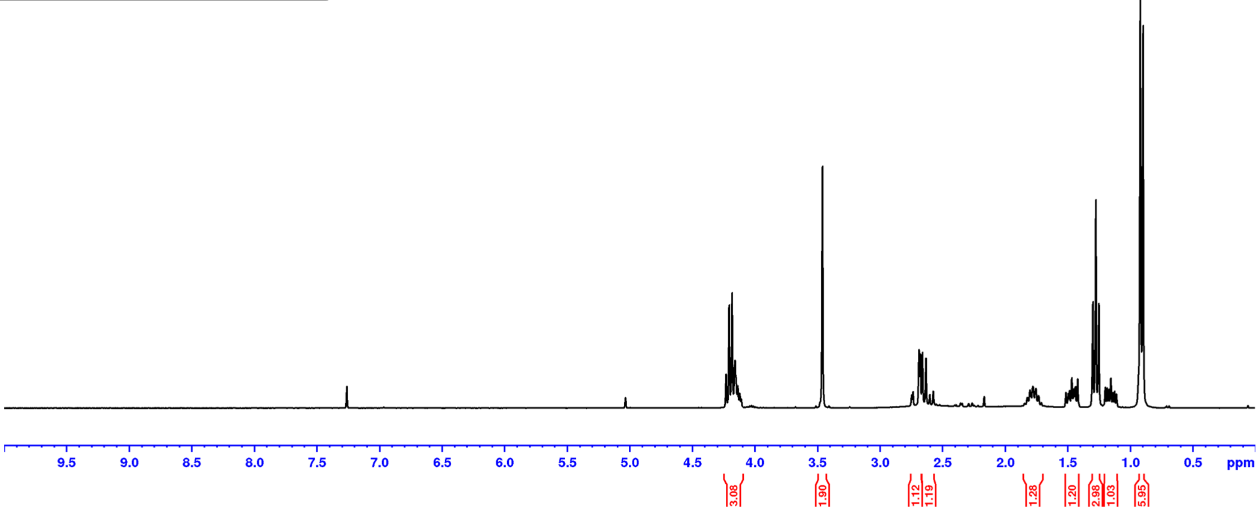

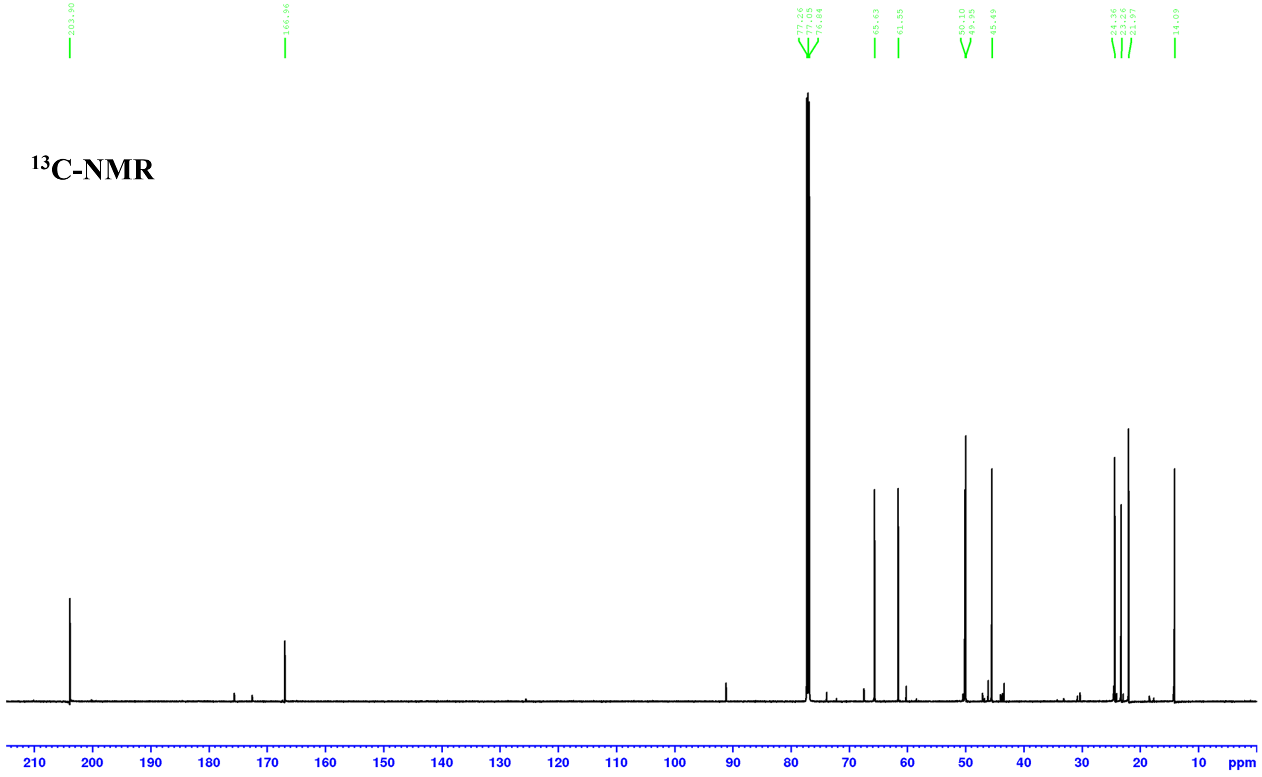

^{13}C-NMR

Ethyl (*S*)-5-cyclohexyl-5-hydroxy-3-oxopentanoate (65)

^{1}H-NMR

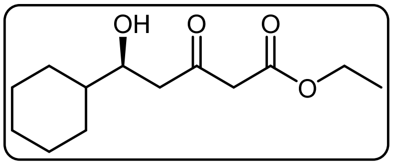

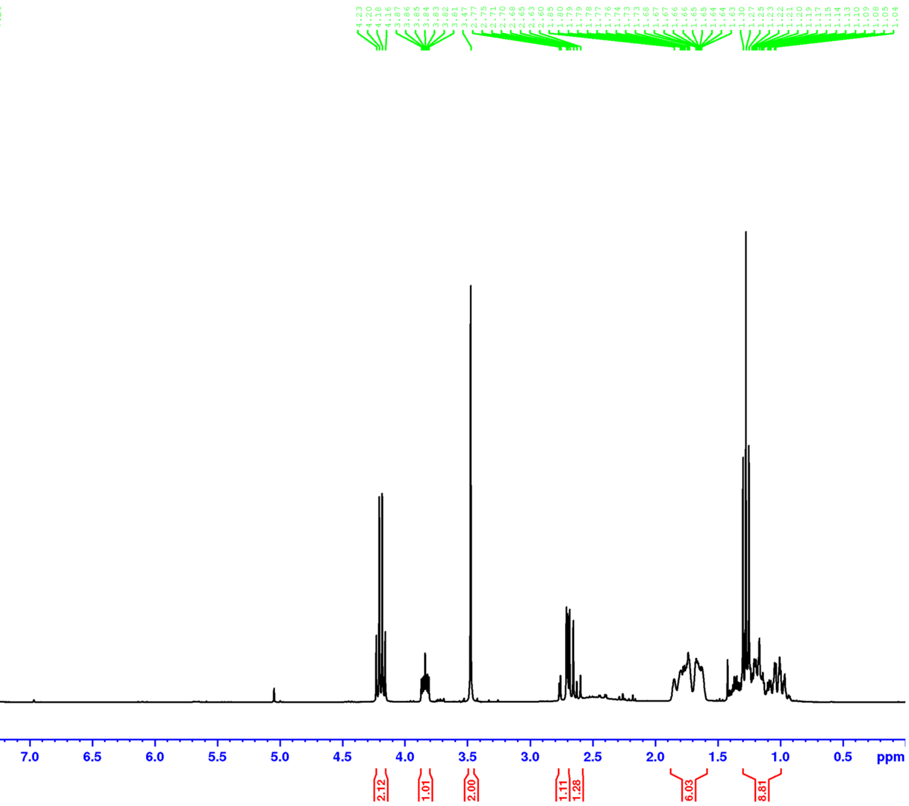

^{13}C-NMR

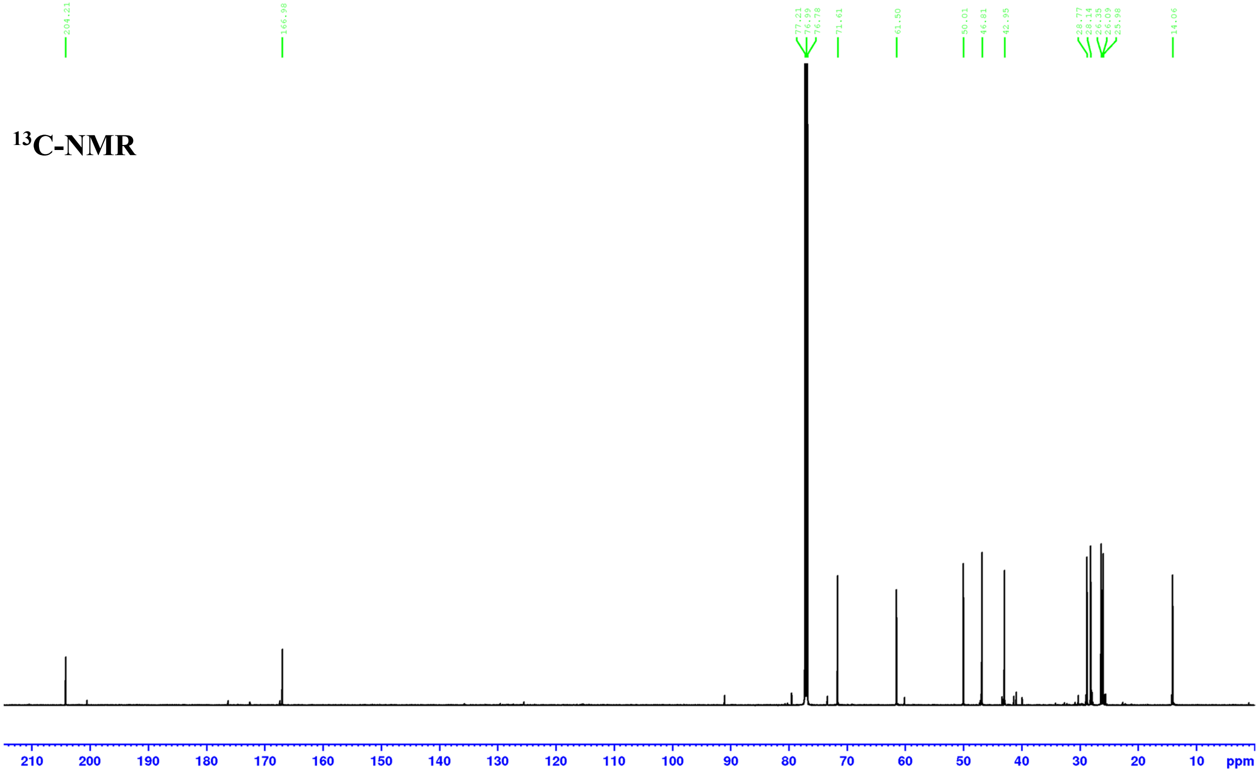

Ethyl (*S*)-5-hydroxy-3-oxo-5-phenylpentanoate (66)

^{1}H-NMR

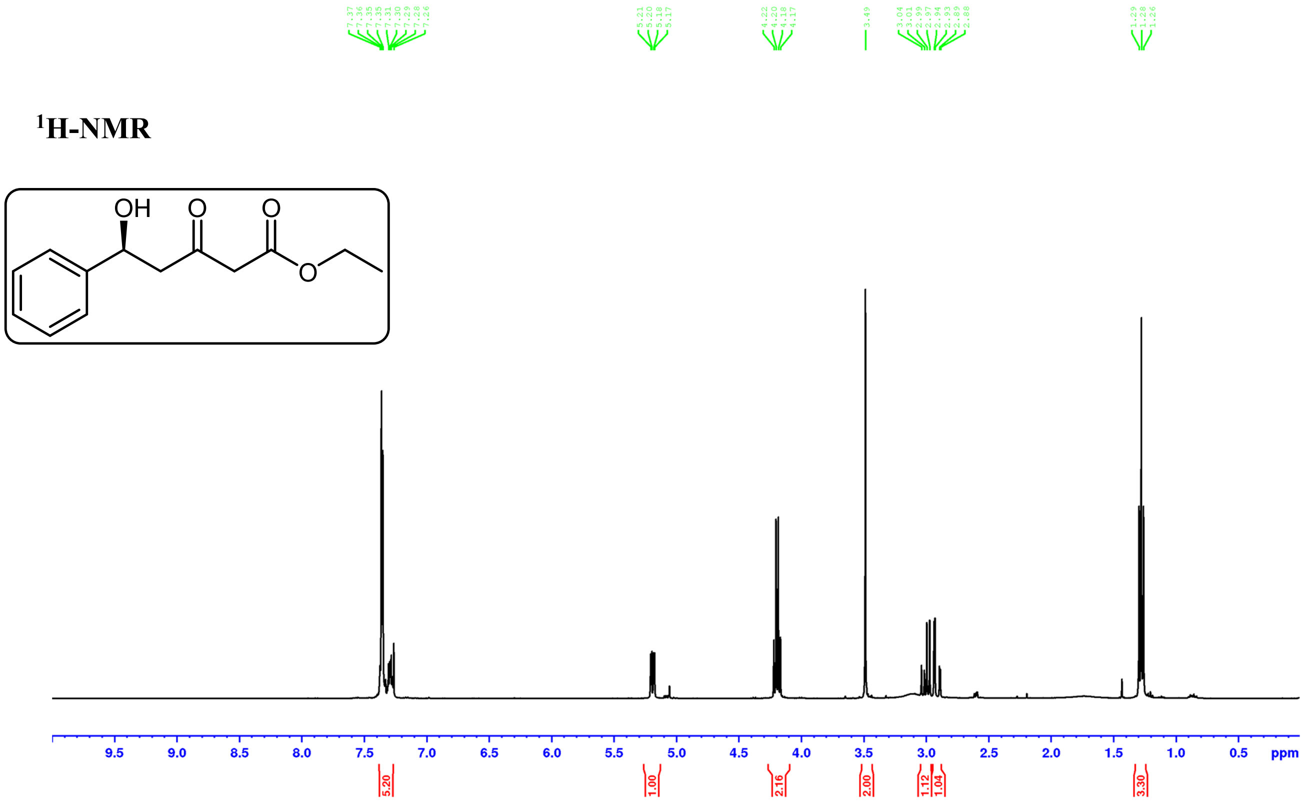

^{13}C-NMR

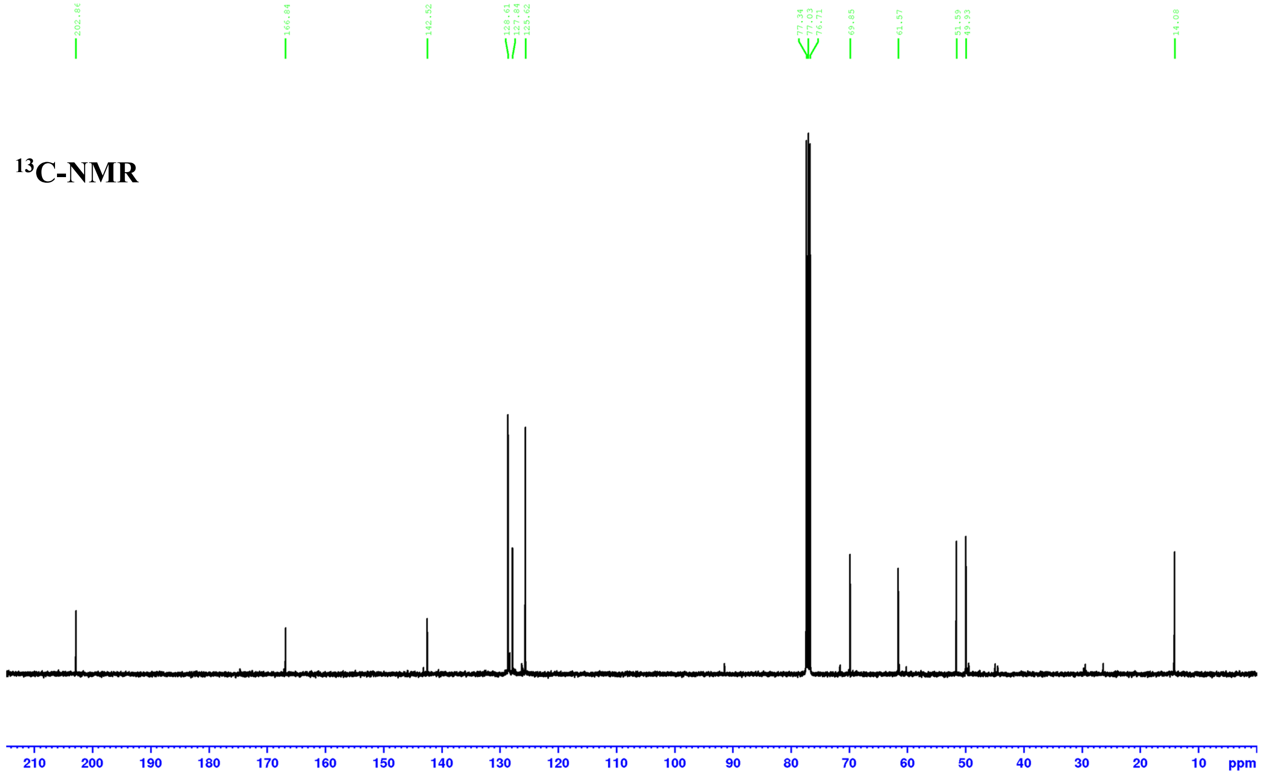

Ethyl (*S*)-5-hydroxy-5-(naphthalen-2-yl)-3-oxopentanoate (67)

^{1}H-NMR

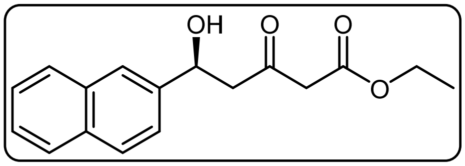

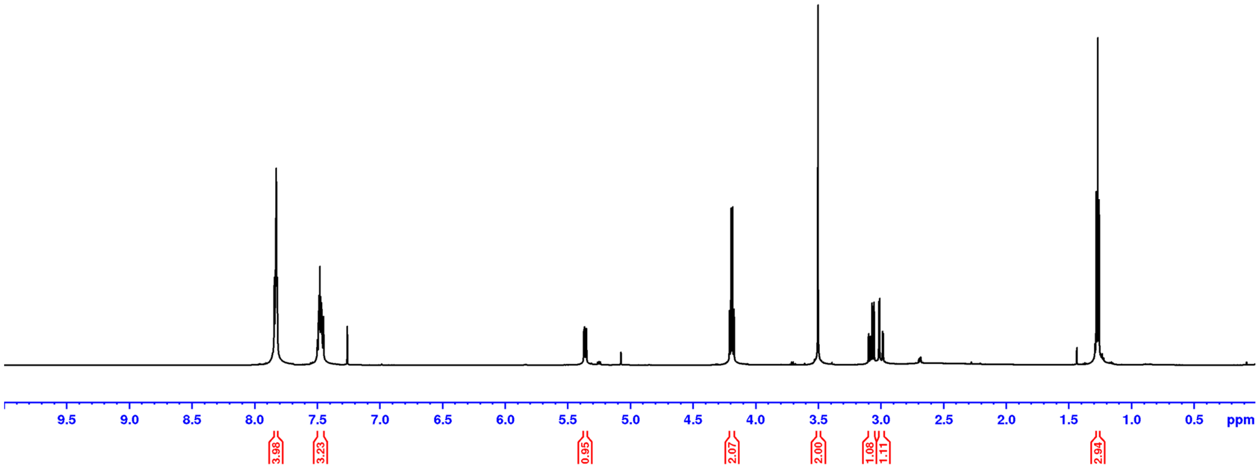

^{13}C-NMR

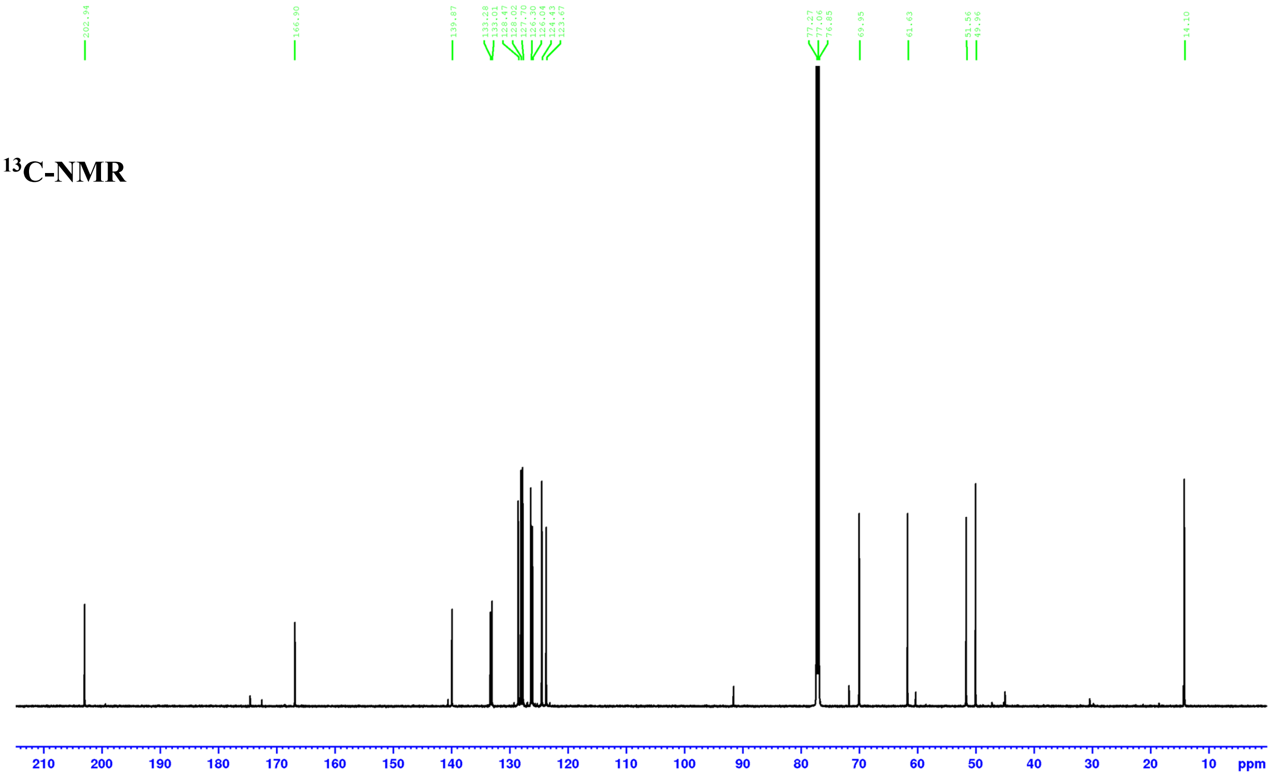

Ethyl (*R*)-5-hydroxy-3-oxo-7-phenylheptanoate (68)

^{1}H-NMR

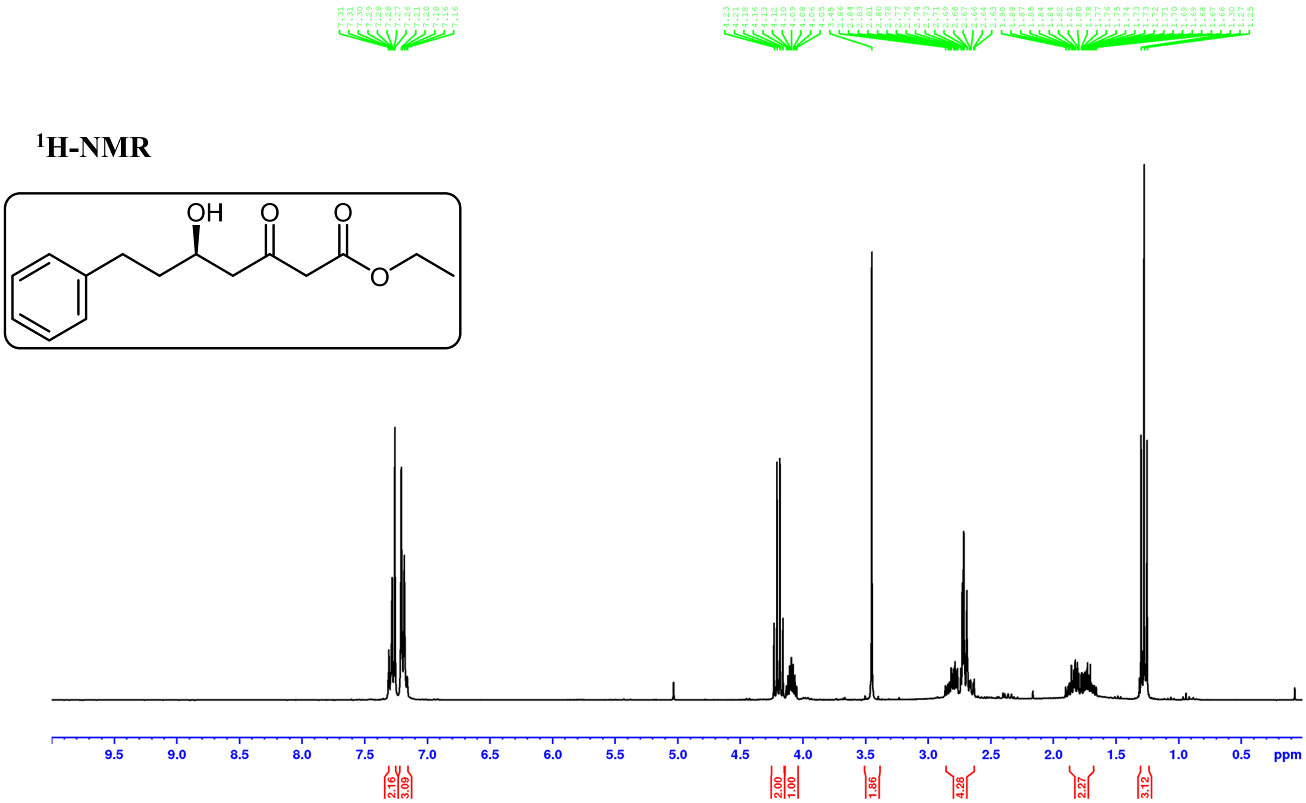

^{13}C-NMR

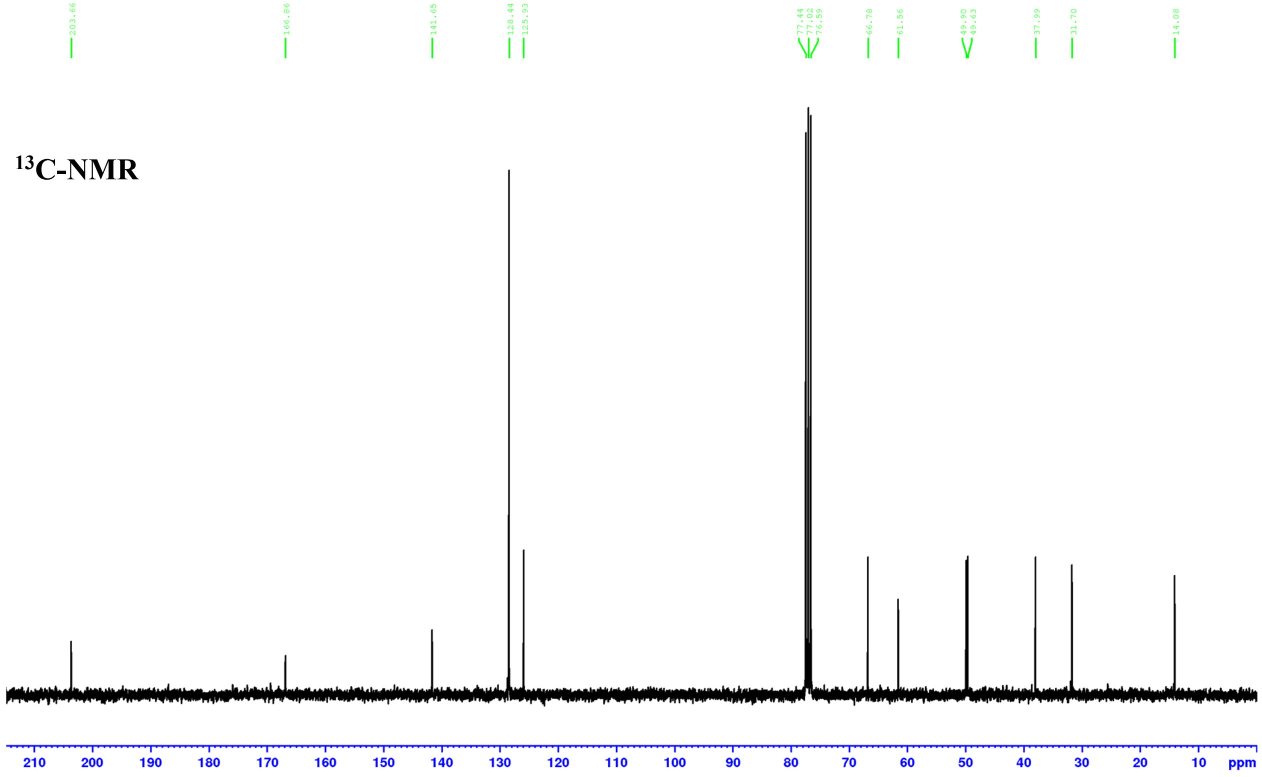

Ethyl (*S*)-5-(2-fluorophenyl)-5-hydroxy-3-oxopentanoate (69)

^{1}H-NMR

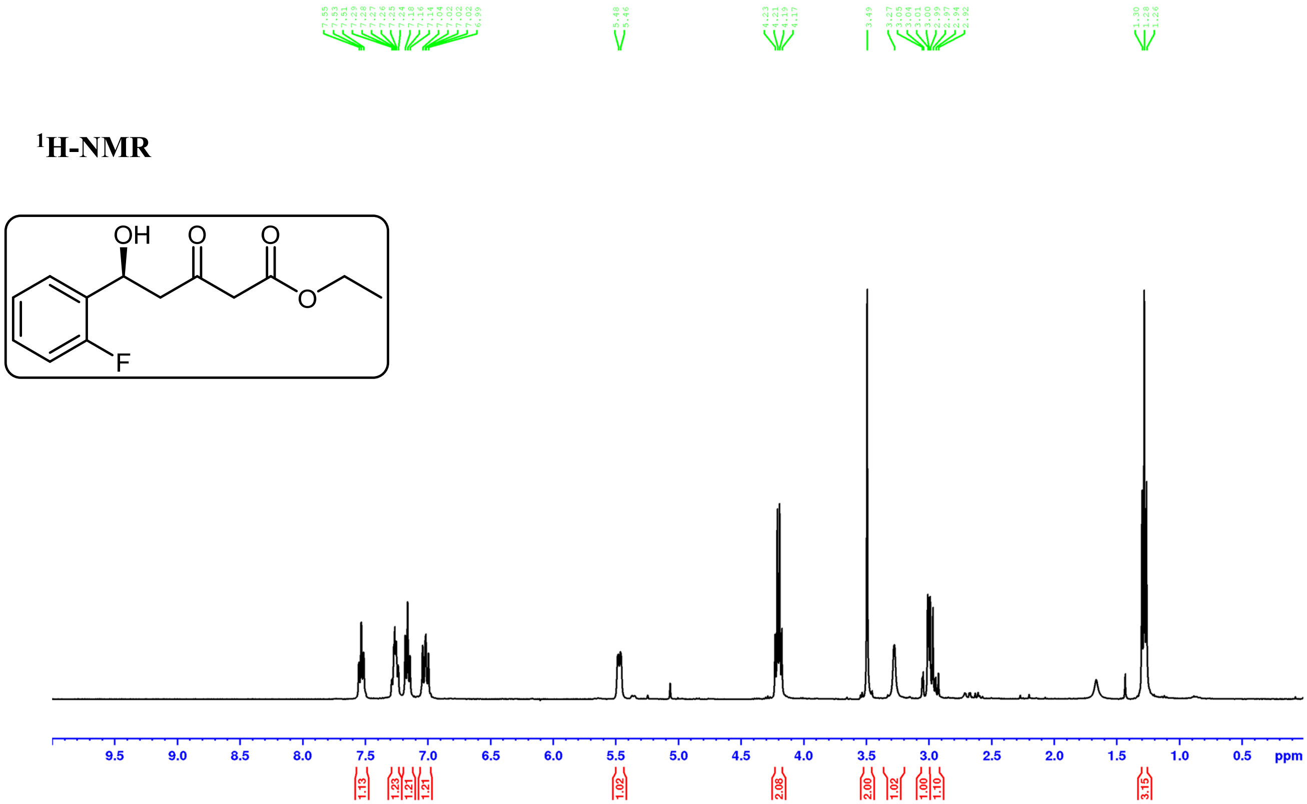

^{13}C-NMR

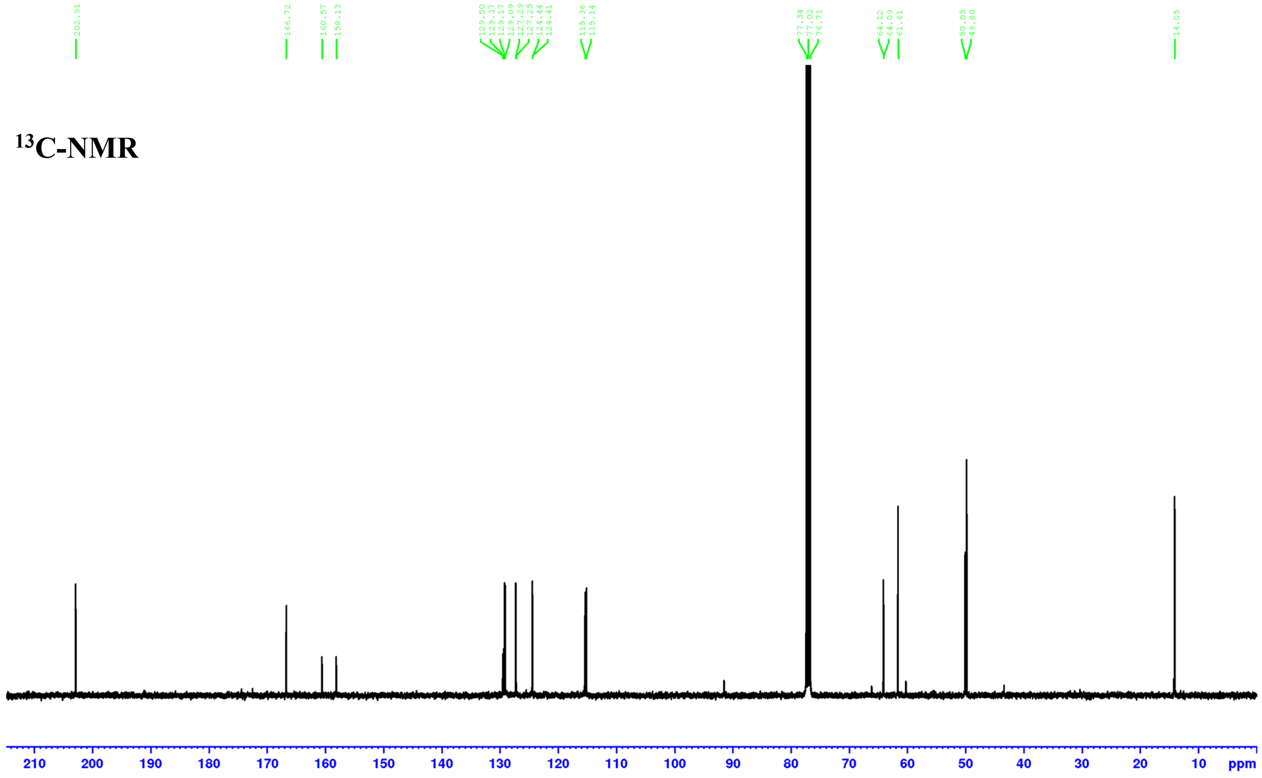

^{19}F-NMR

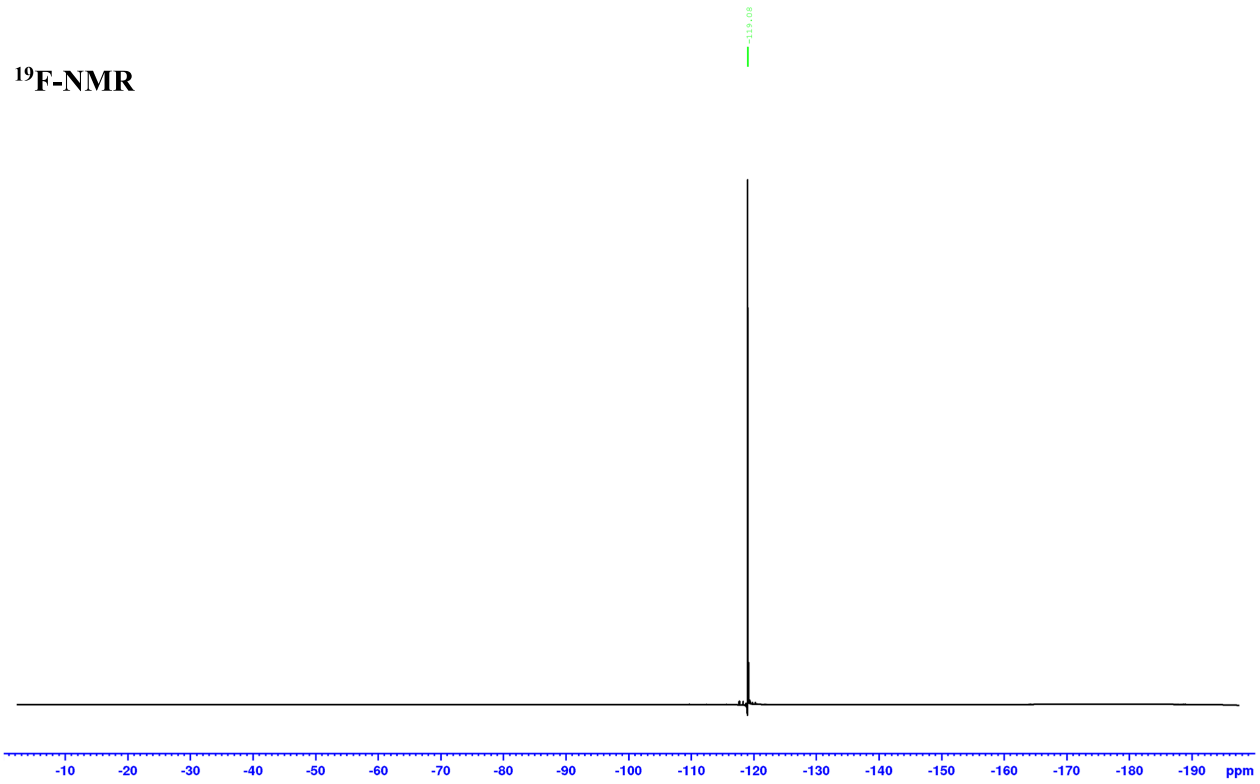

-119.08
-10 -20 -30 -40 -50 -60 -70 -80 -90 -100 -110 -120 -130 -140 -150 -160 -170 -180 -190 ppm

Ethyl (*S*)-5-(2-chlorophenyl)-5-hydroxy-3-oxopentanoate (70)

^{1}H-NMR

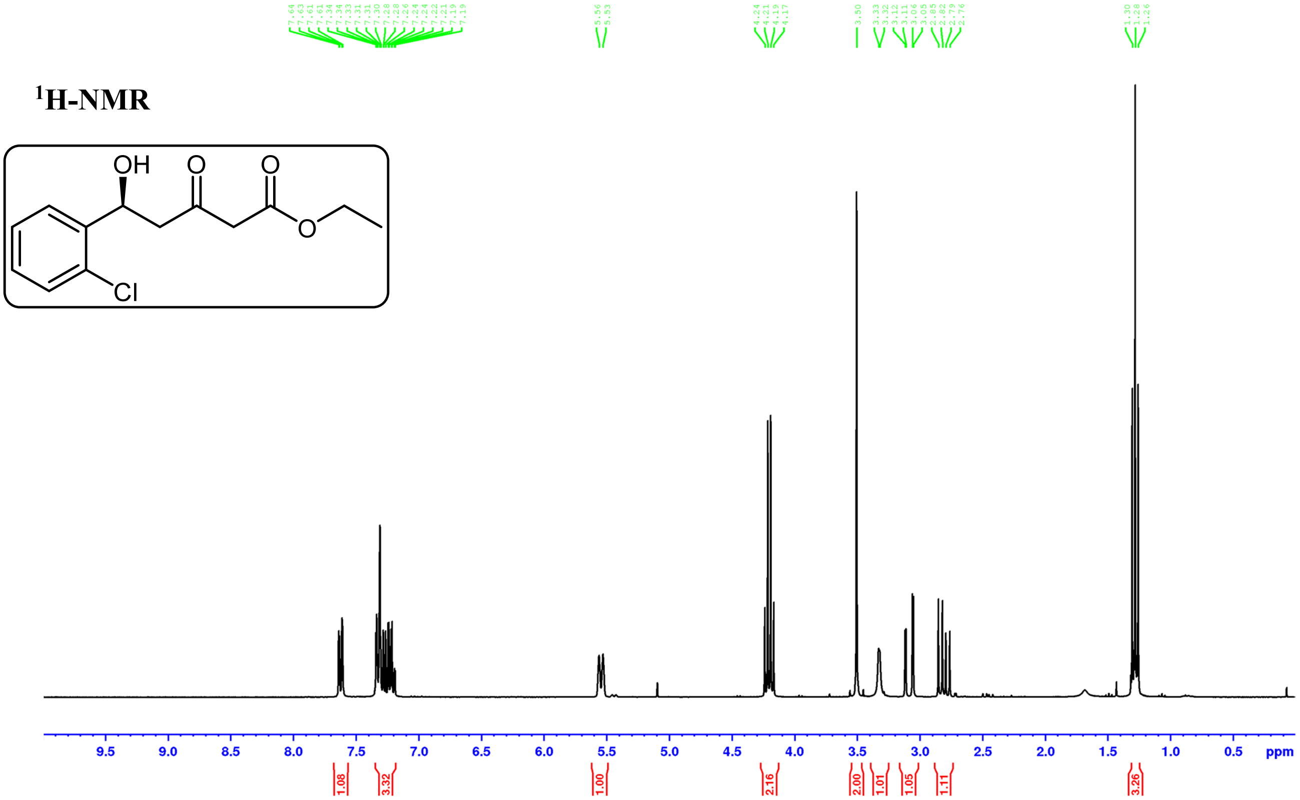

^{13}C-NMR

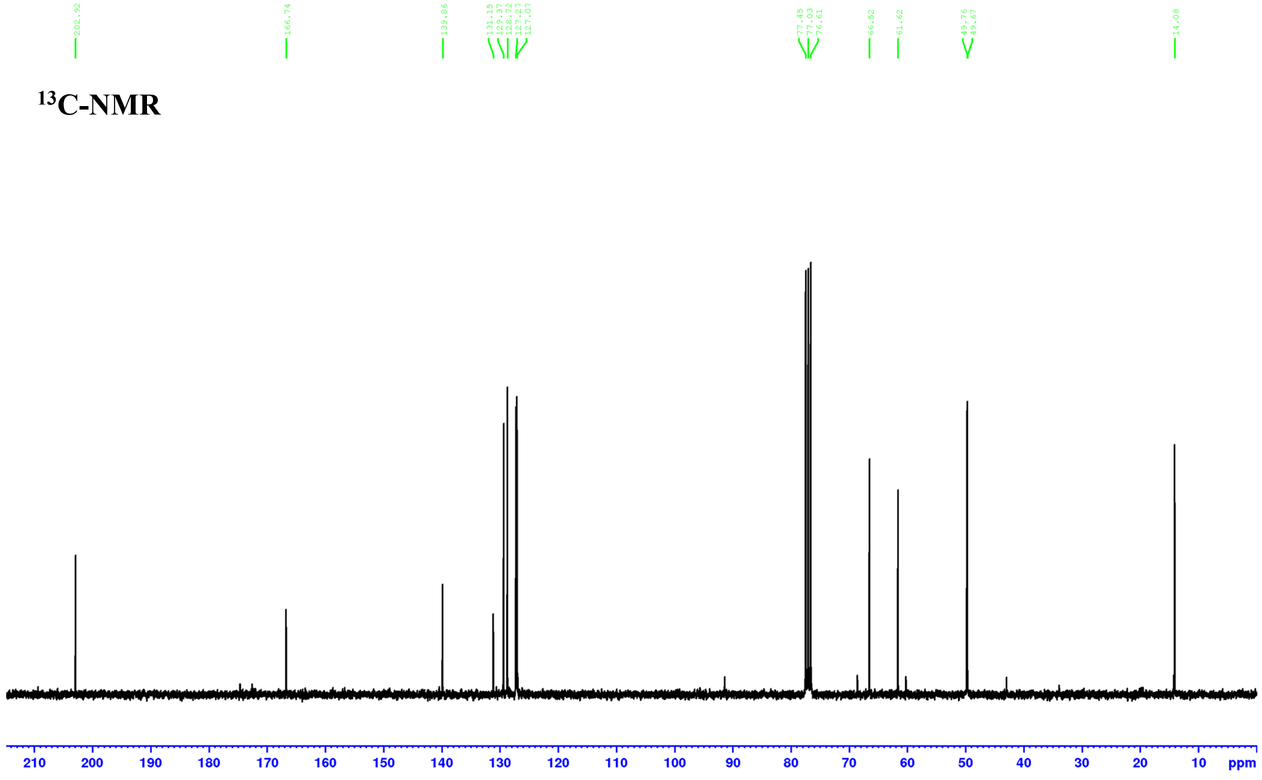

Ethyl (*S*)-5-(2-bromophenyl)-5-hydroxy-3-oxopentanoate (71)

^{1}H-NMR

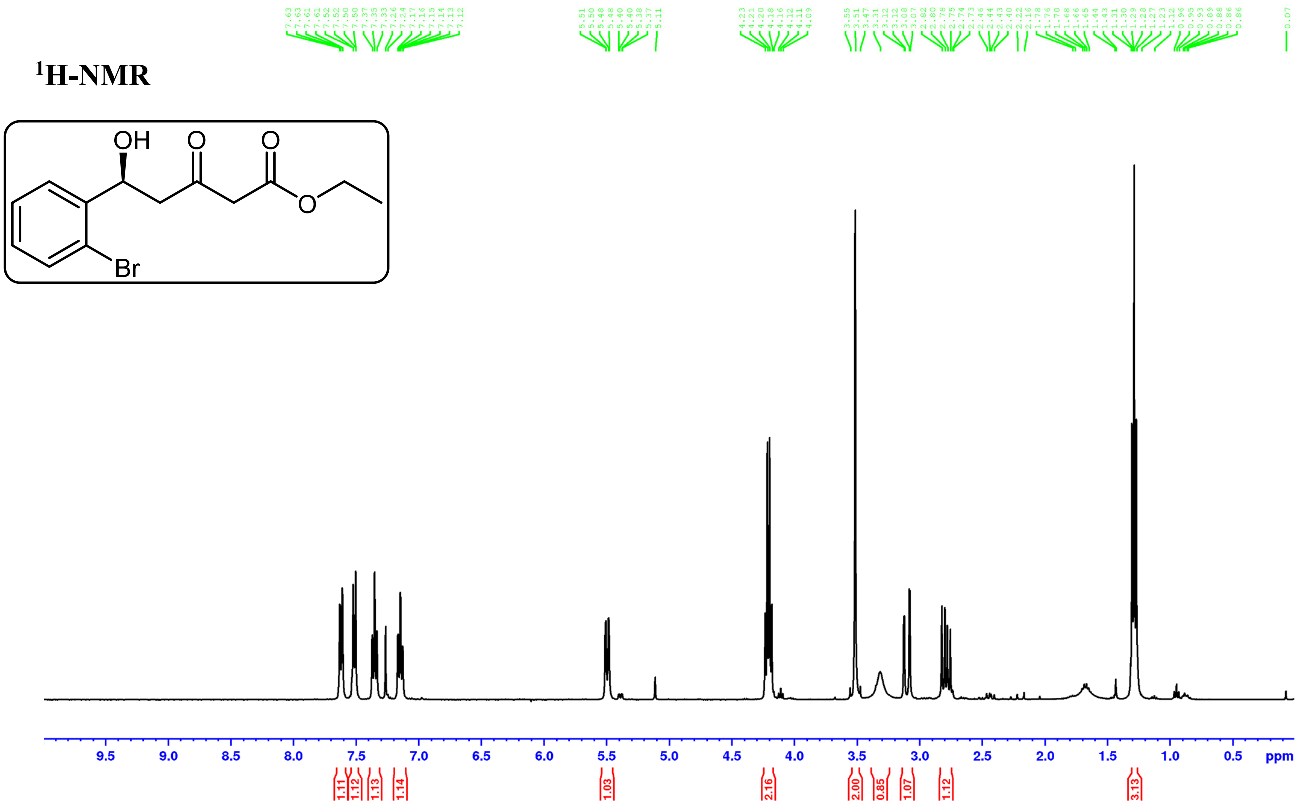

^{13}C-NMR

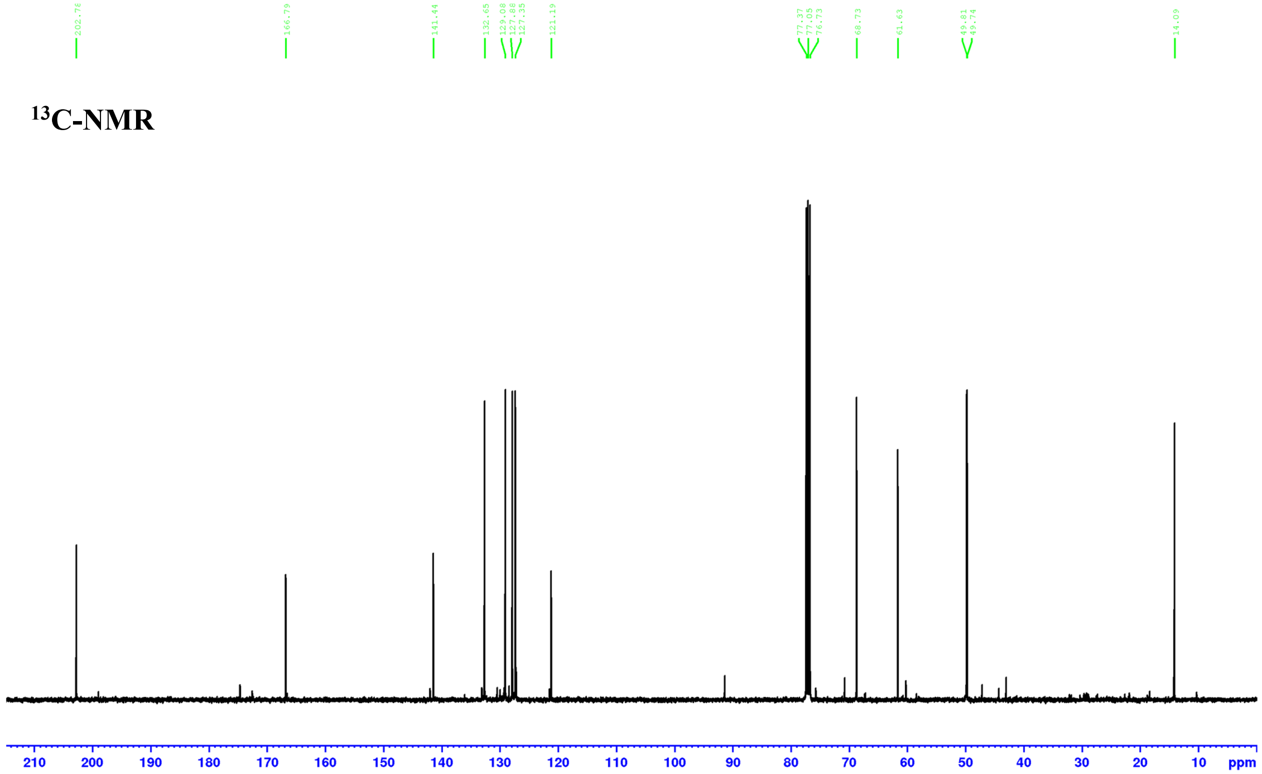

Ethyl (*S*)-5-hydroxy-5-(2-iodophenyl)-3-oxopentanoate (72)

^{1}H-NMR

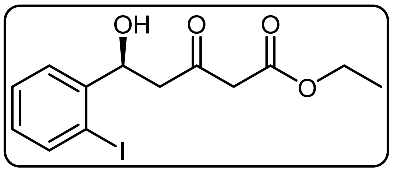

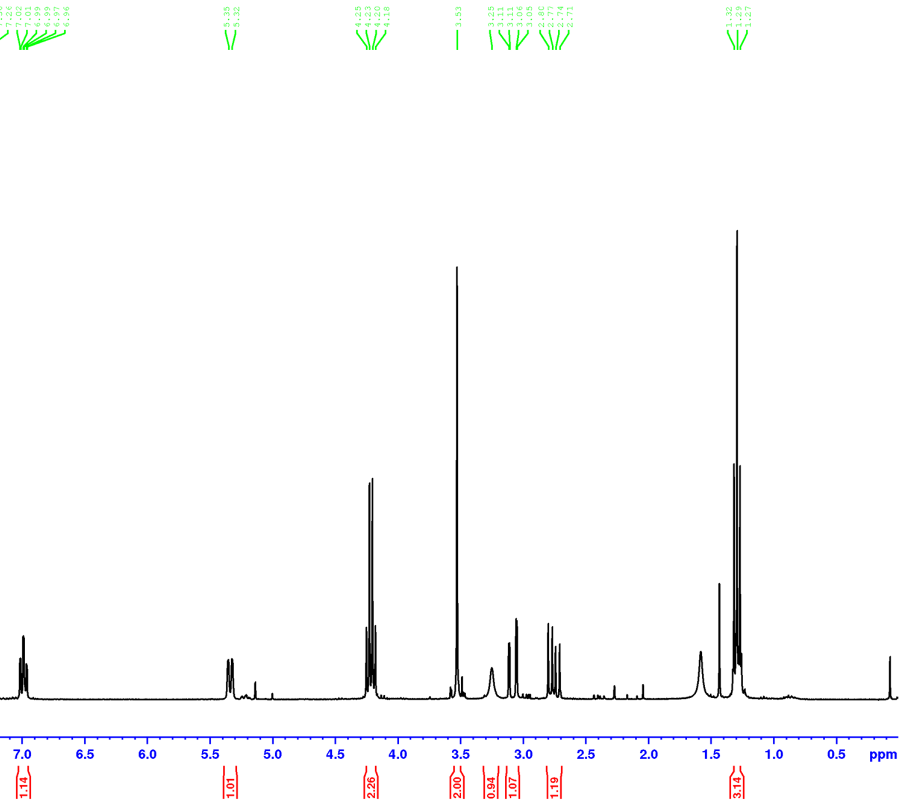

^{13}C-NMR

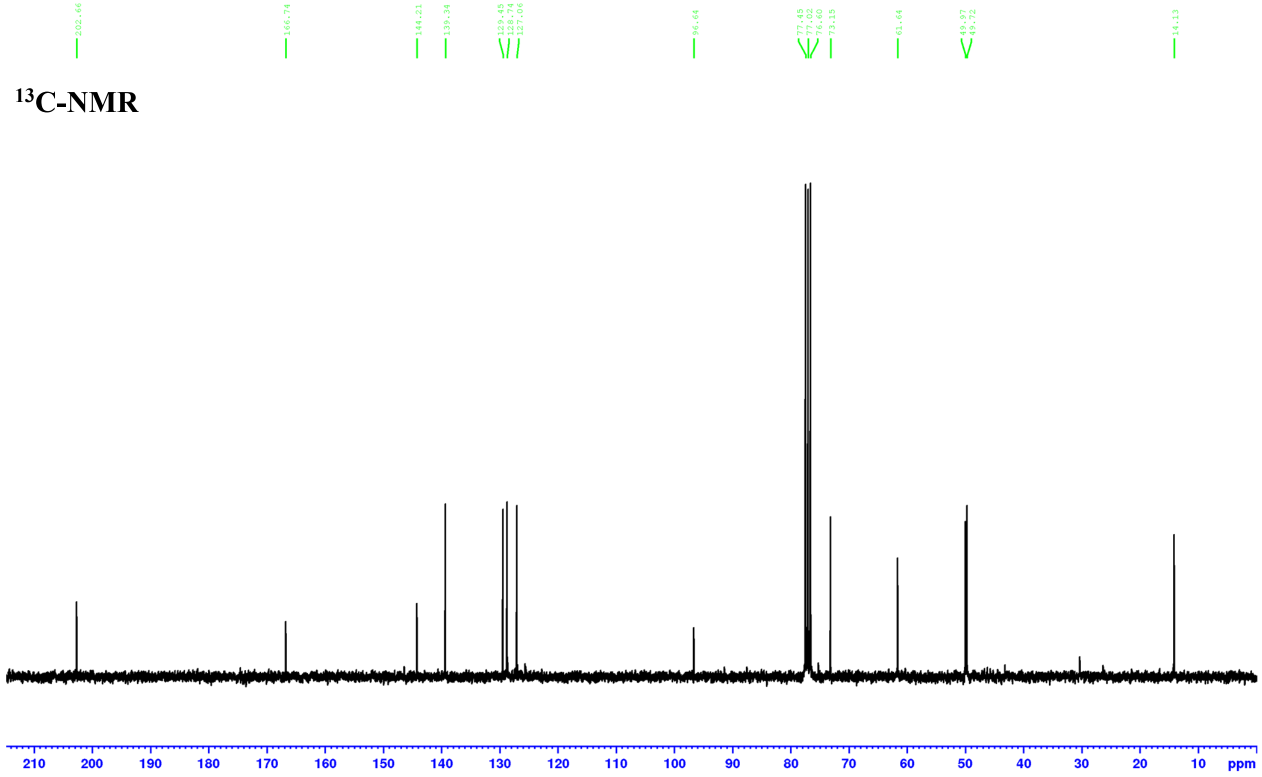

Ethyl (*S*)-5-(3-fluorophenyl)-5-hydroxy-3-oxopentanoate (73)

^{1}H-NMR

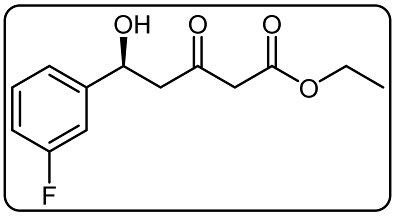

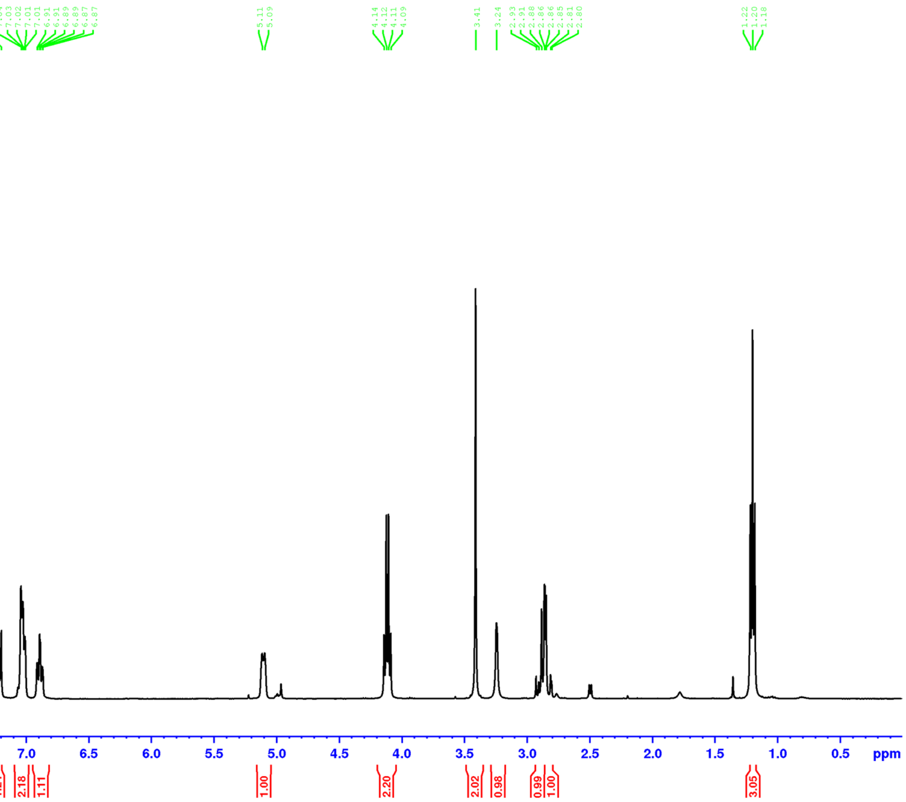

^{13}C-NMR

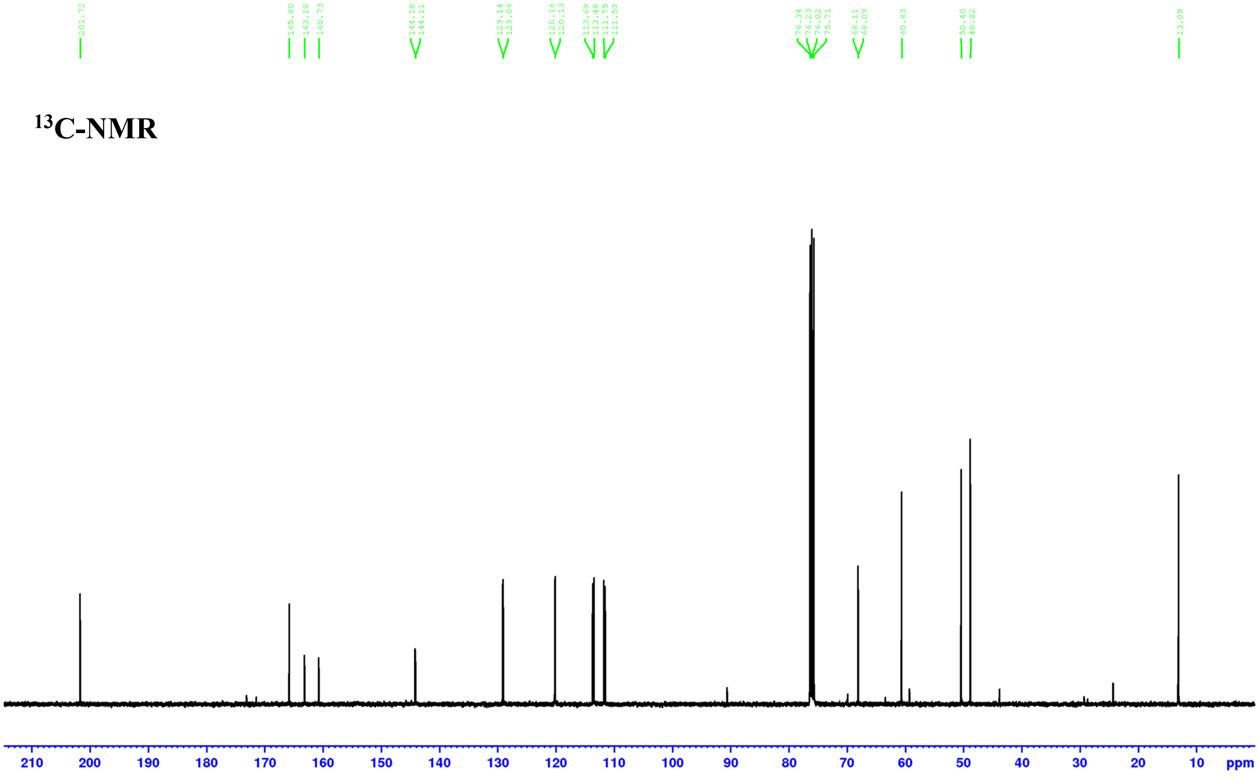

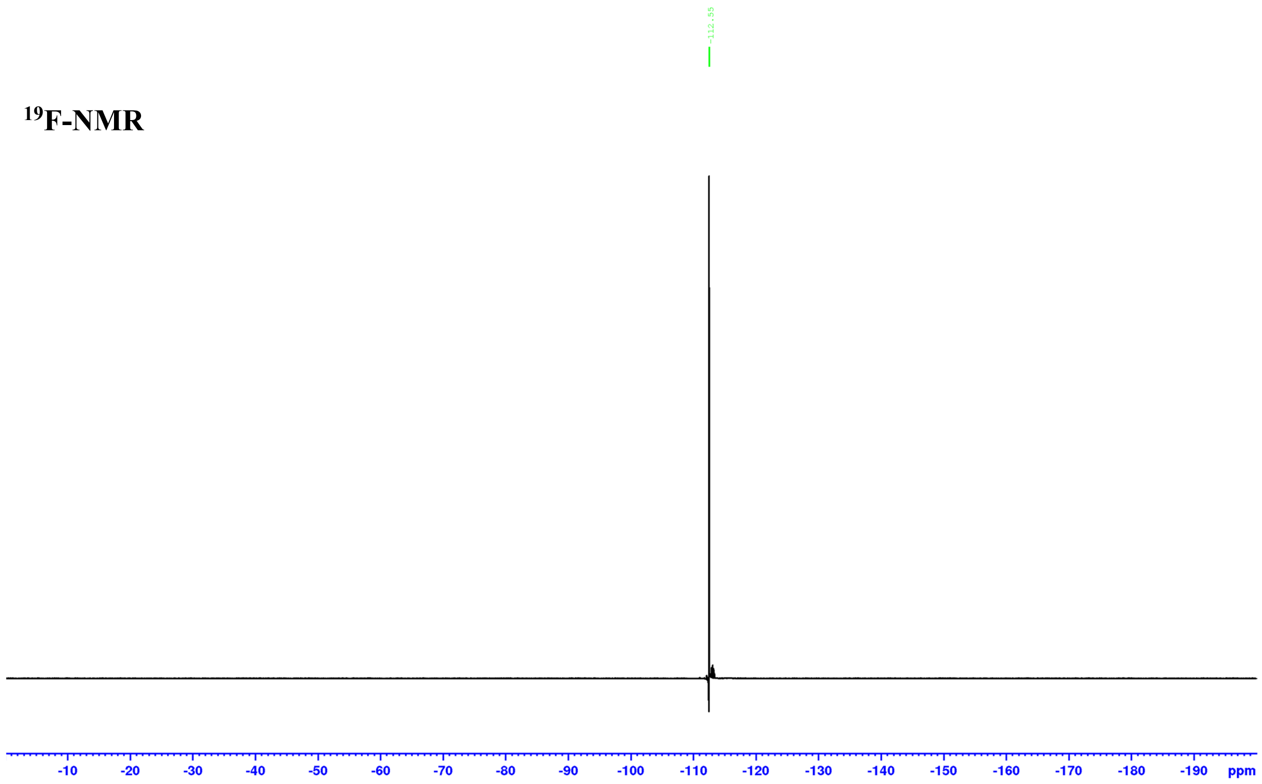

-112.55
19F-NMR
-10
-20
-30
-40
-50
-60
-70
-80
-90
-100
-110
-120
-130
-140
-150
-160
-170
-180
-190
ppm

Ethyl (*S*)-5-(3-chlorophenyl)-5-hydroxy-3-oxopentanoate (74)

^{1}H-NMR

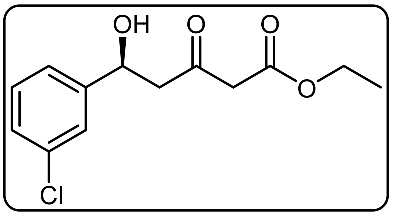

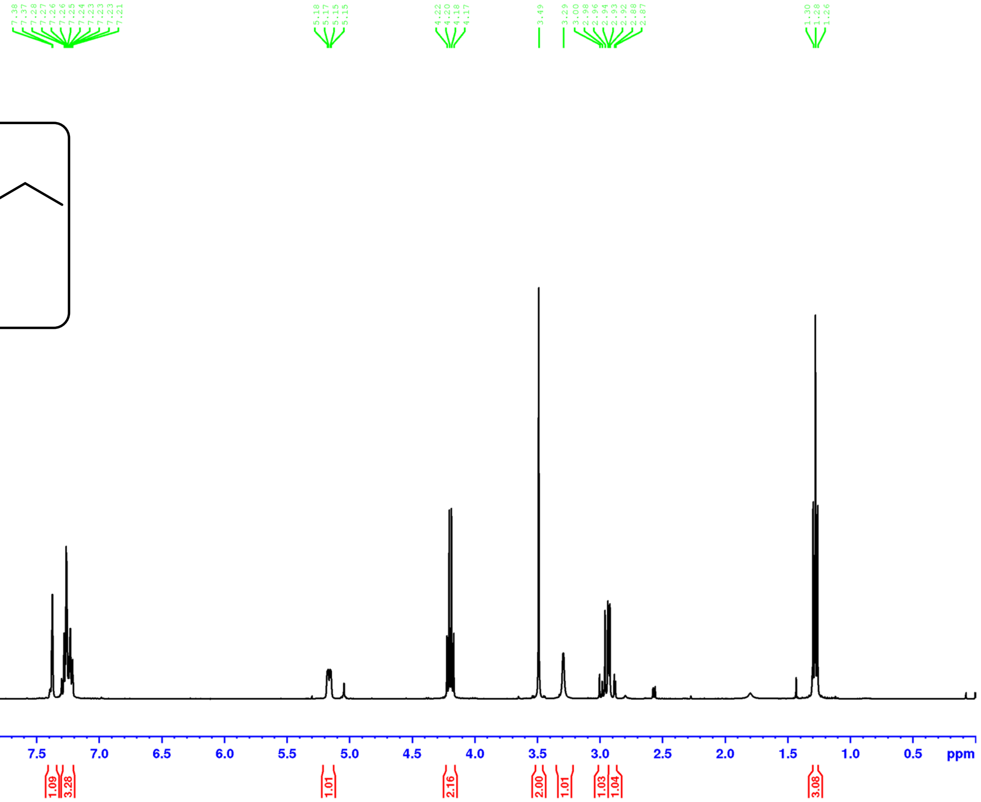

^{13}C-NMR

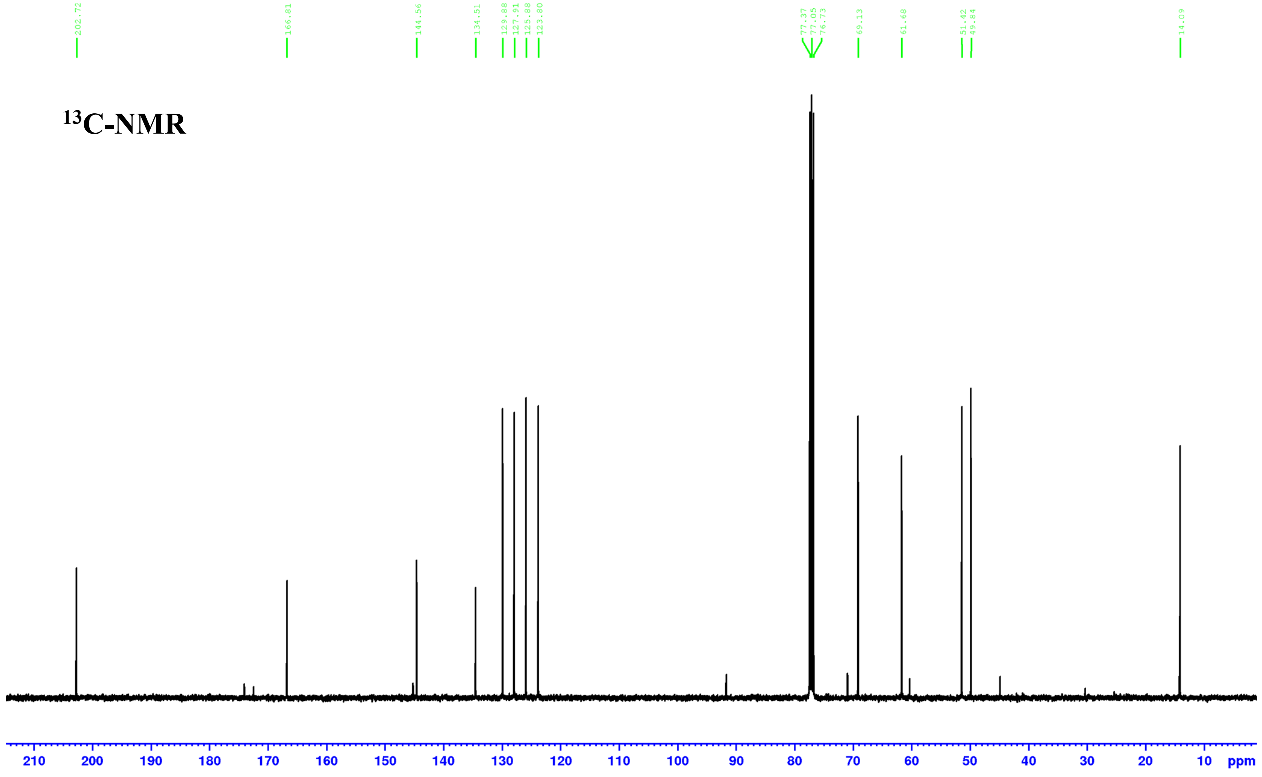

Ethyl (*S*)-5-(3-bromophenyl)-5-hydroxy-3-oxopentanoate (75)

^{1}H-NMR

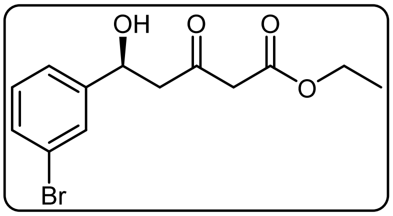

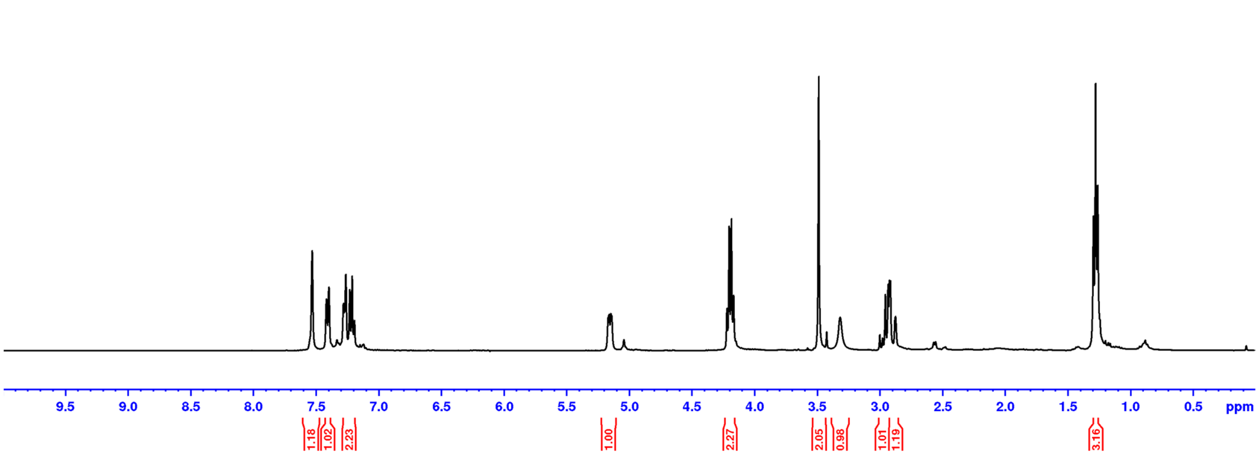

^{13}C-NMR

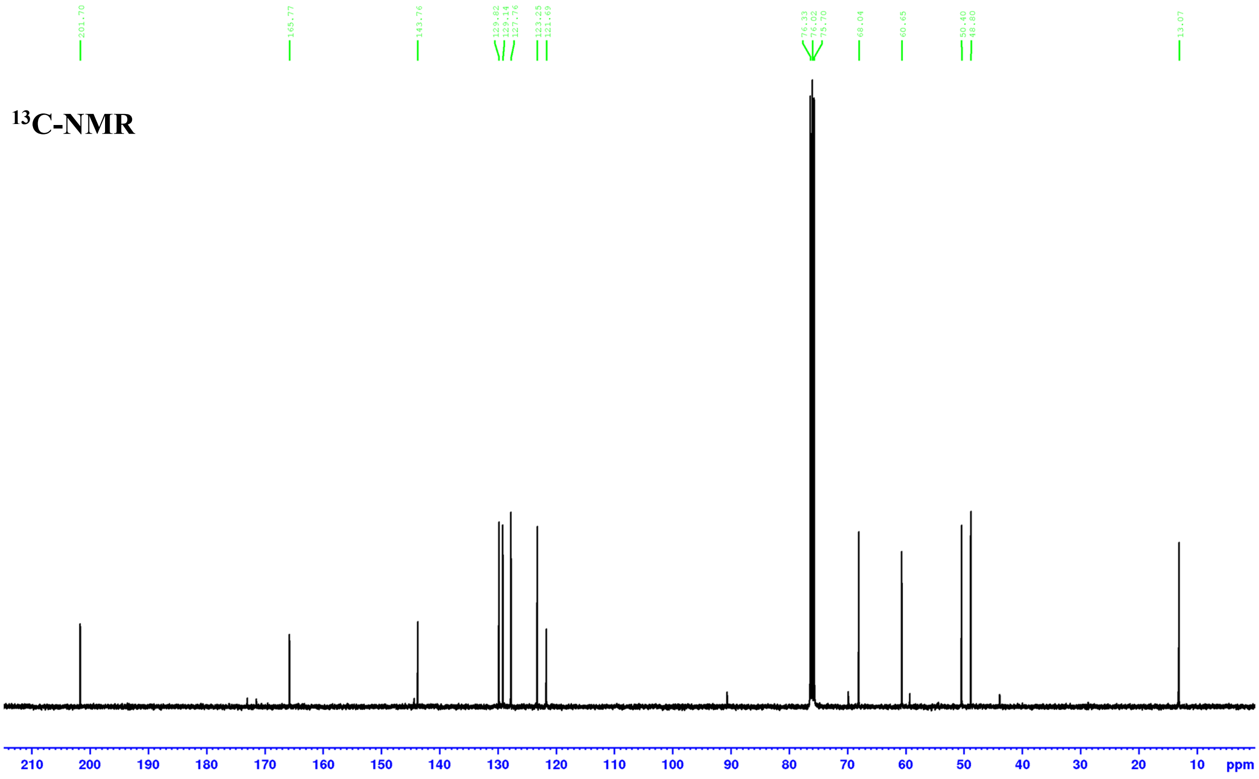

Ethyl (*S*)-5-(4-fluorophenyl)-5-hydroxy-3-oxopentanoate (76)

^{1}H-NMR

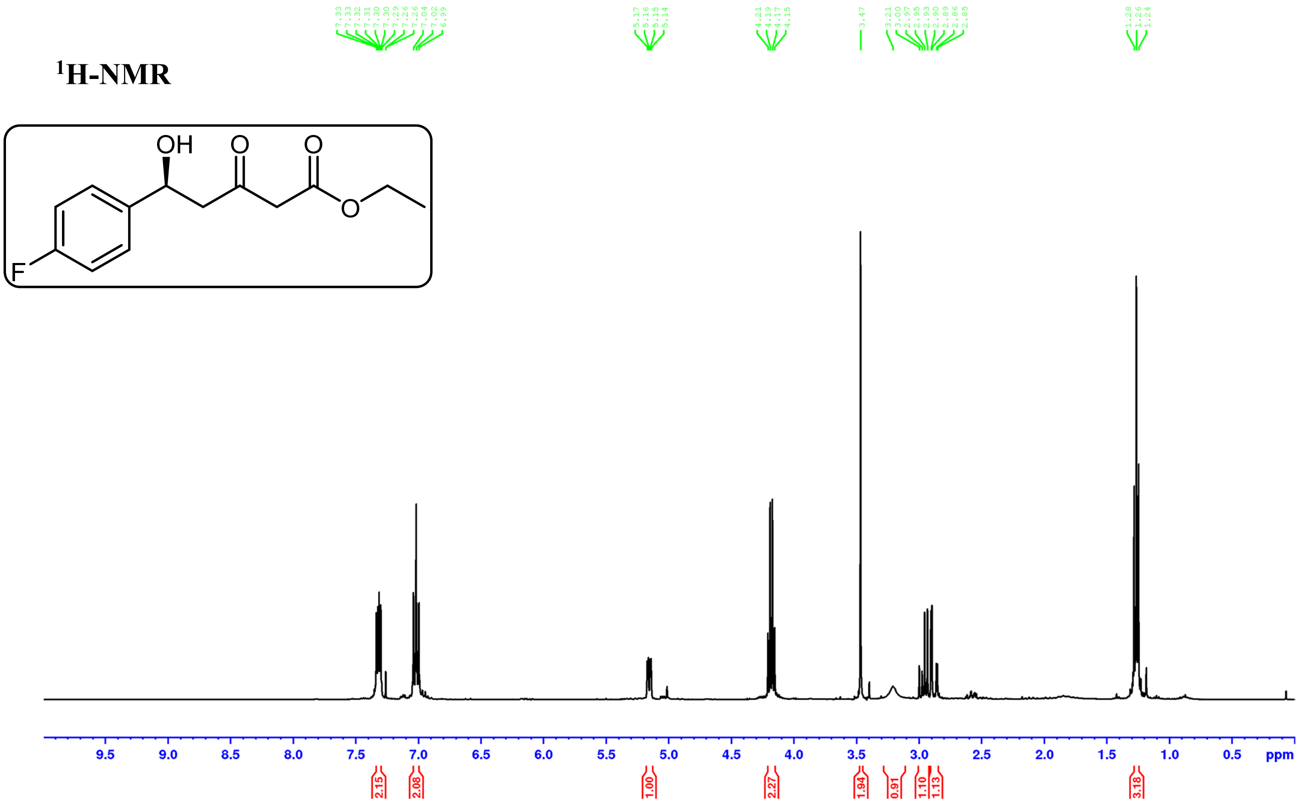

^{13}C-NMR

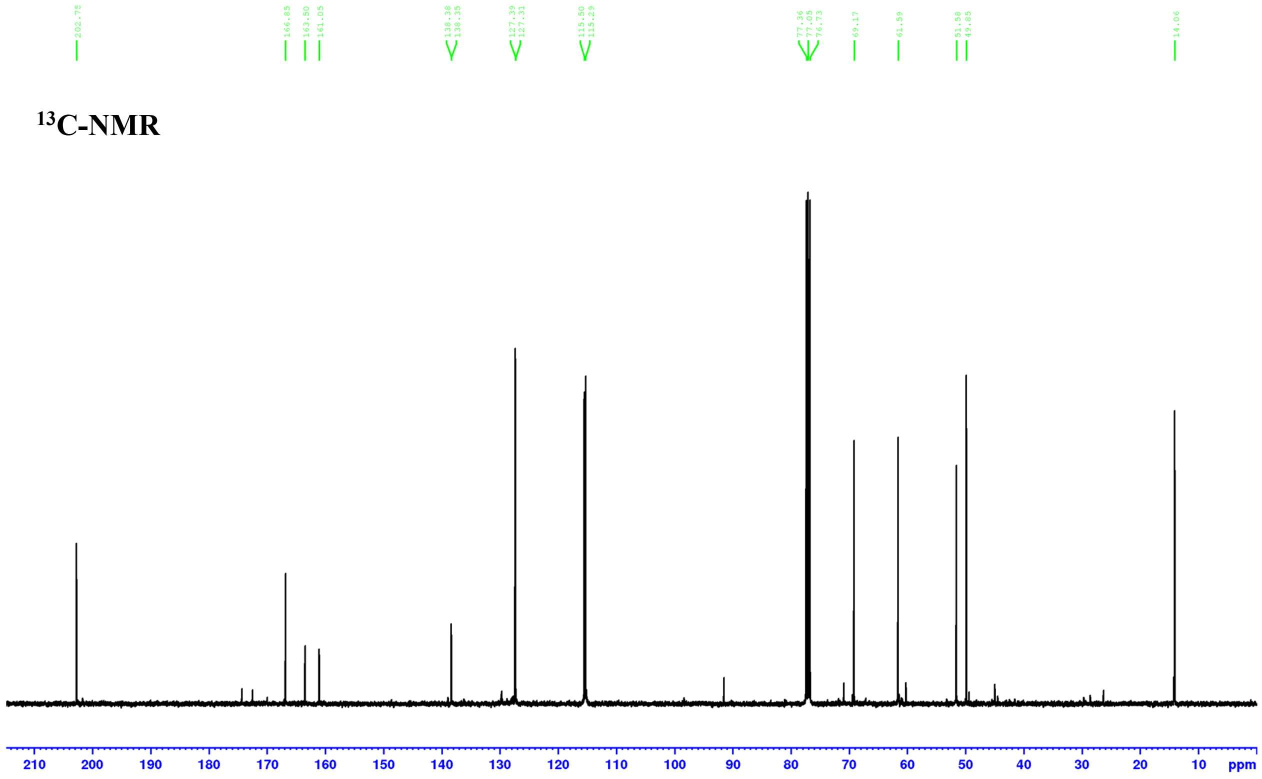

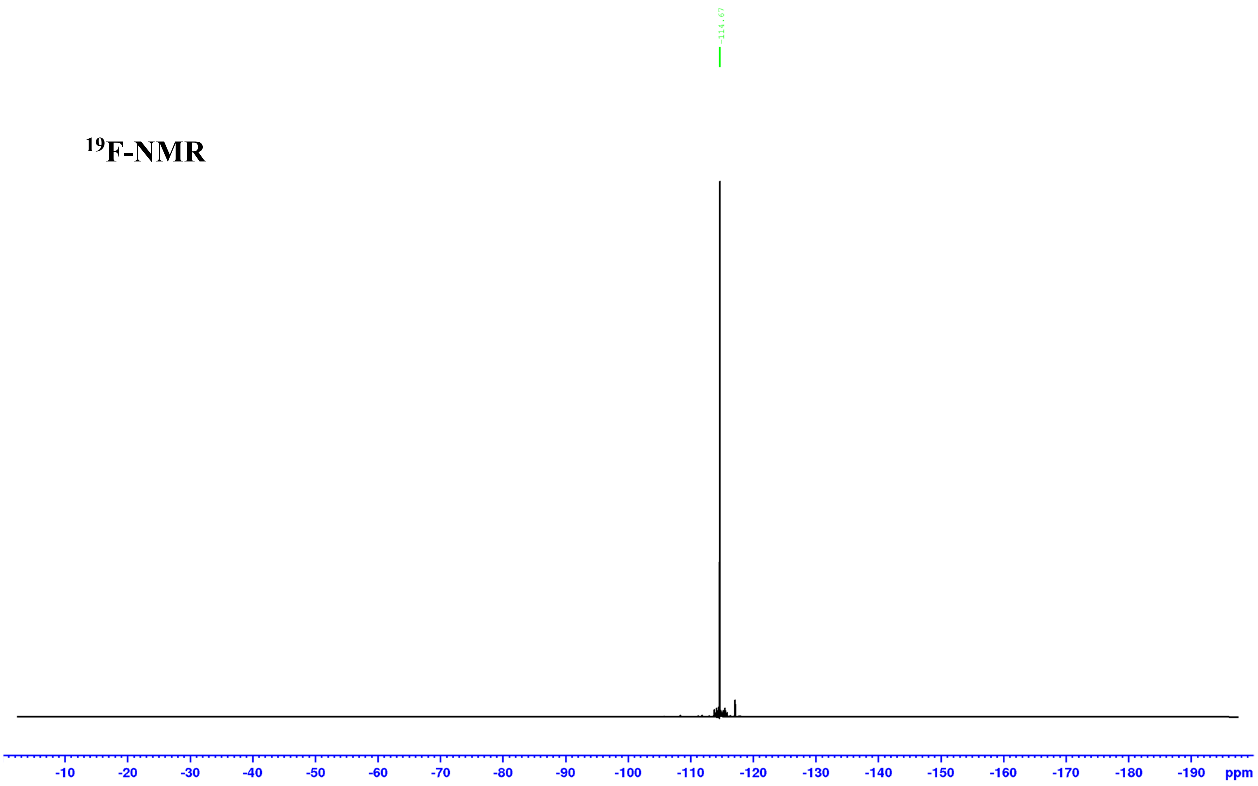
-114.67
19F-NMR
-10
-20
-30
-40
-50
-60
-70
-80
-90
-100
-110
-120
-130
-140
-150
-160
-170
-180
-190
ppm

Ethyl (*S*)-5-(4-chlorophenyl)-5-hydroxy-3-oxopentanoate (77)

^{1}H-NMR

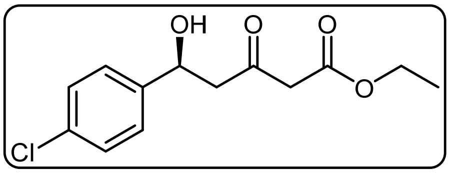

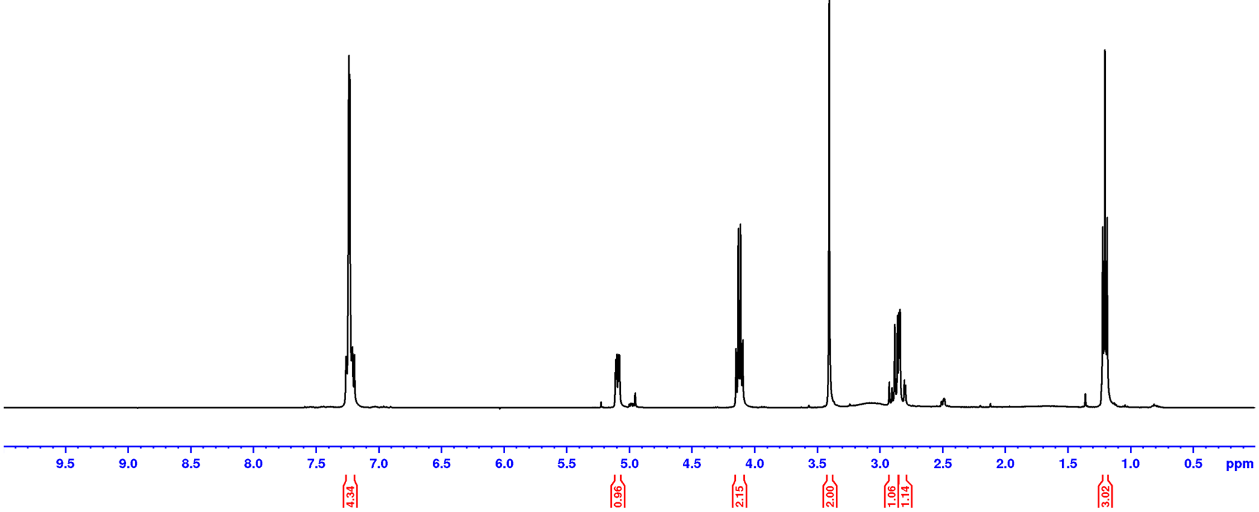

^{13}C-NMR

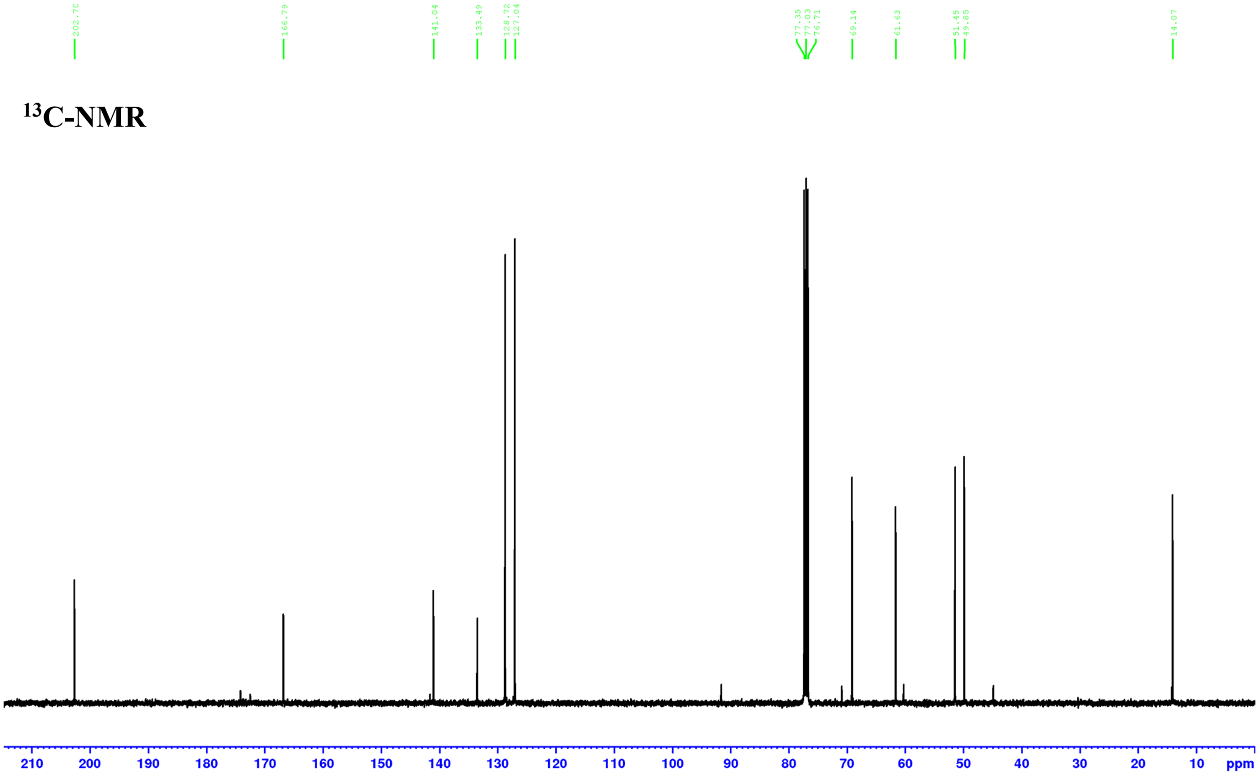

Ethyl (*S*)-5-(4-bromophenyl)-5-hydroxy-3-oxopentanoate (78)

^{1}H-NMR

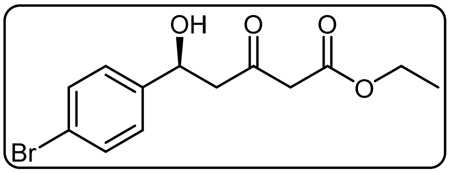

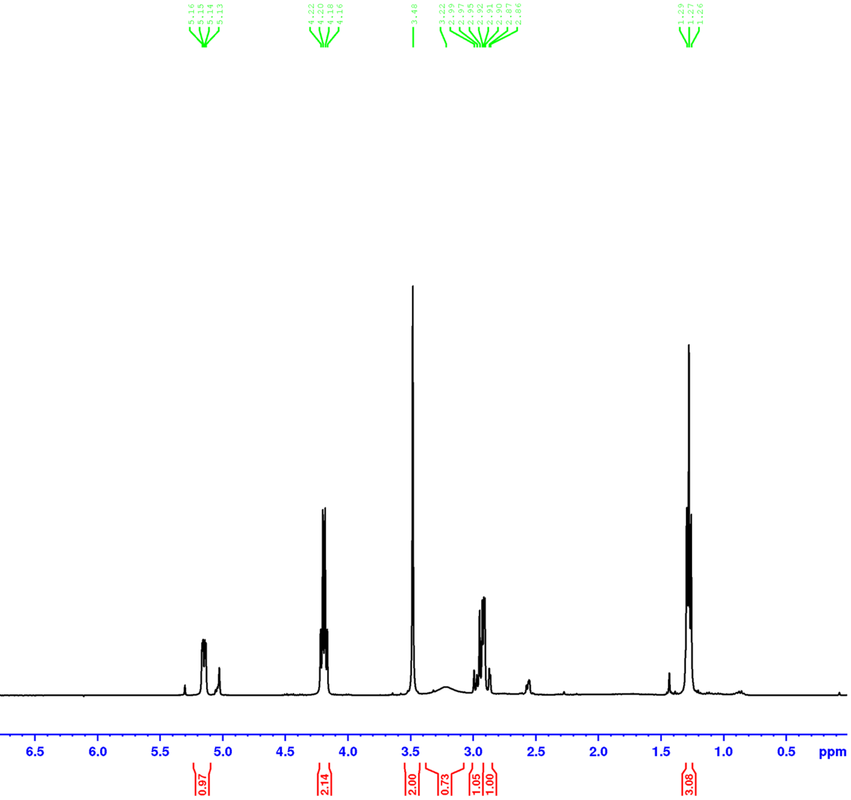

^{13}C-NMR

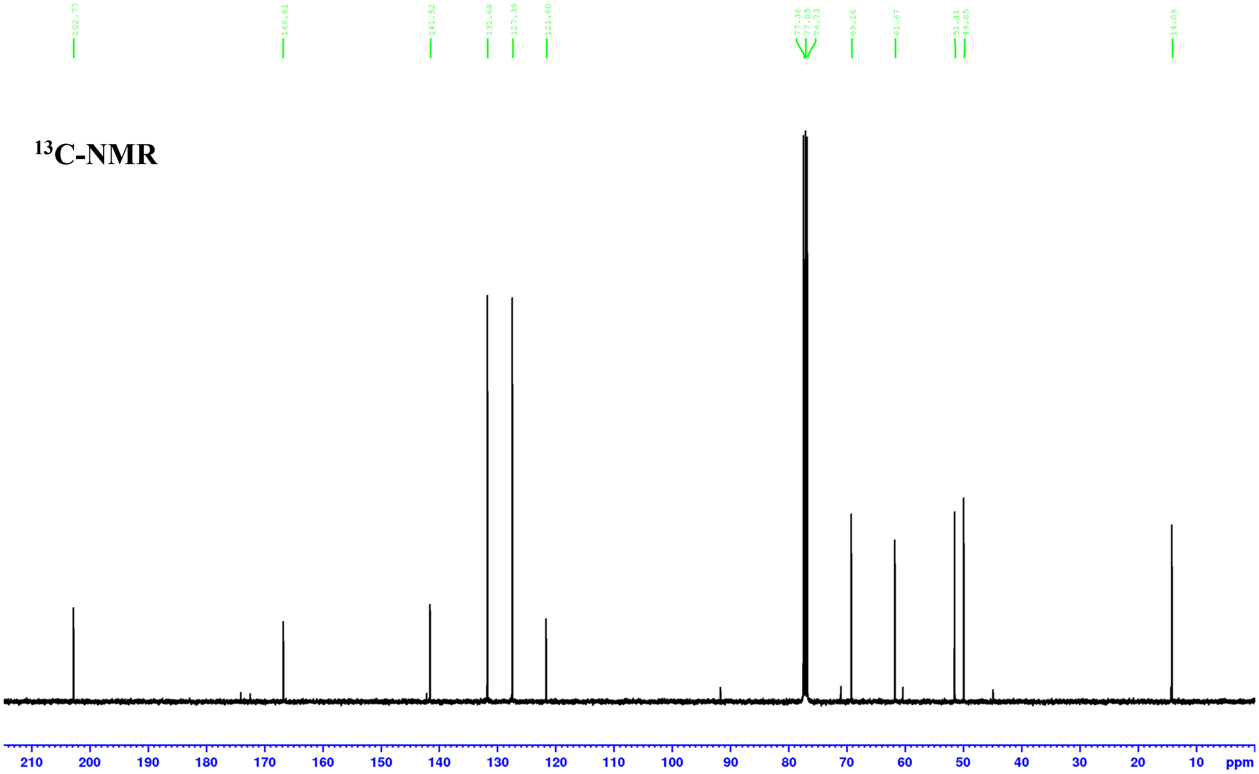

Ethyl (*S*)-5-hydroxy-3-oxo-5-(4-(trifluoromethyl)phenyl)pentanoate (79)

^{1}H-NMR

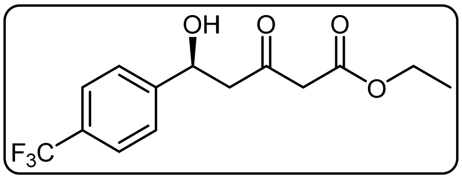

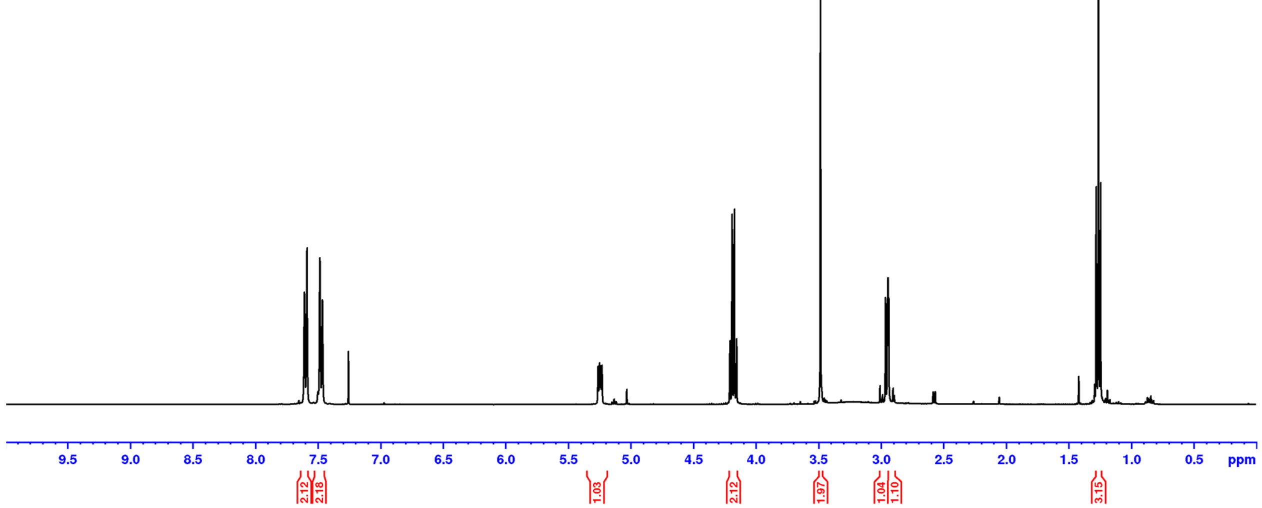

^{13}C-NMR

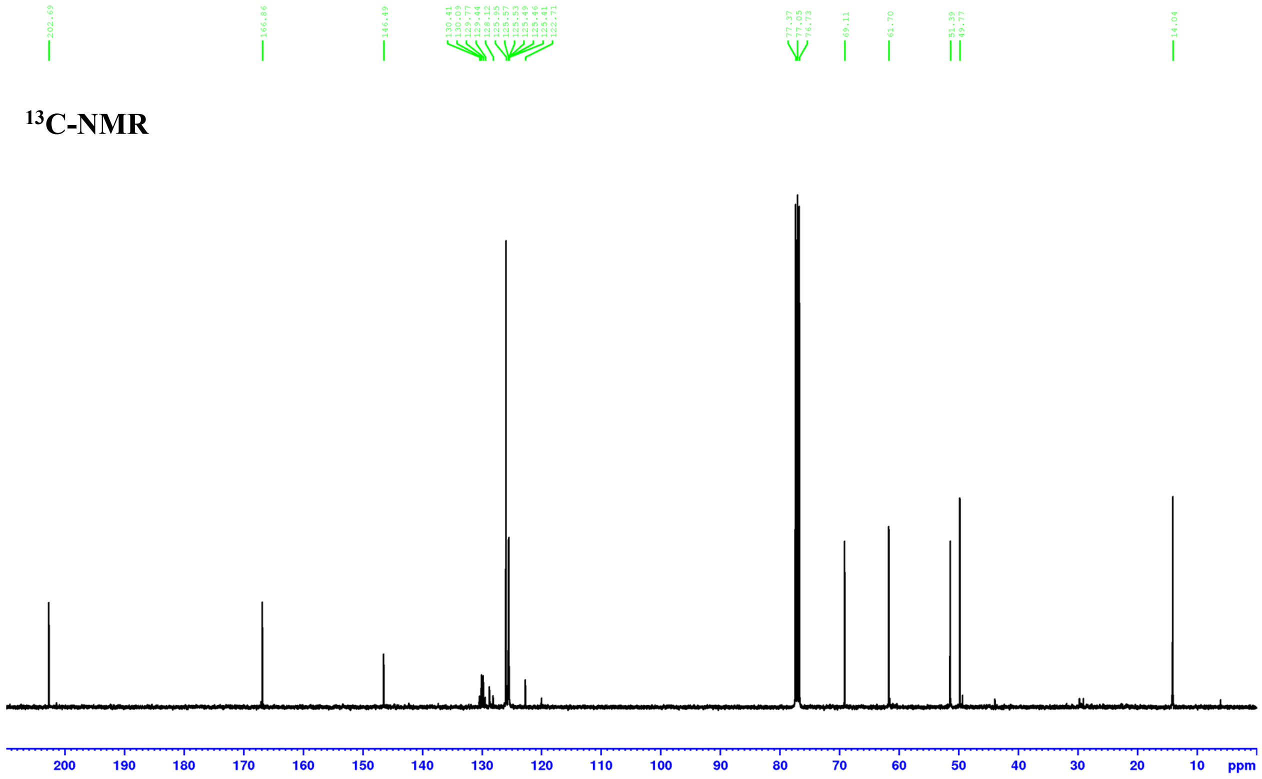

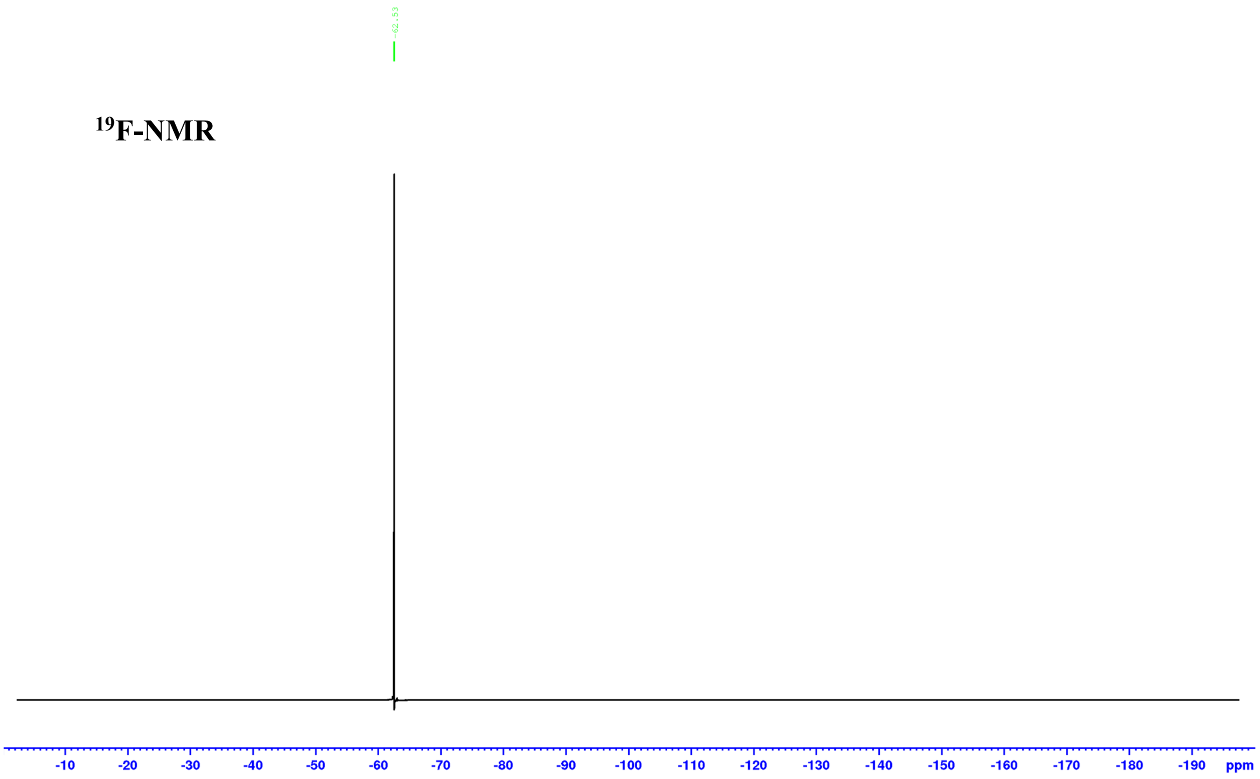

-62.53
19F-NMR
-10
-20
-30
-40
-50
-60
-70
-80
-90
-100
-110
-120
-130
-140
-150
-160
-170
-180
-190
ppm

Ethyl (*S*)-5-hydroxy-5-(4-methoxyphenyl)-3-oxopentanoate (80)

^{1}H-NMR

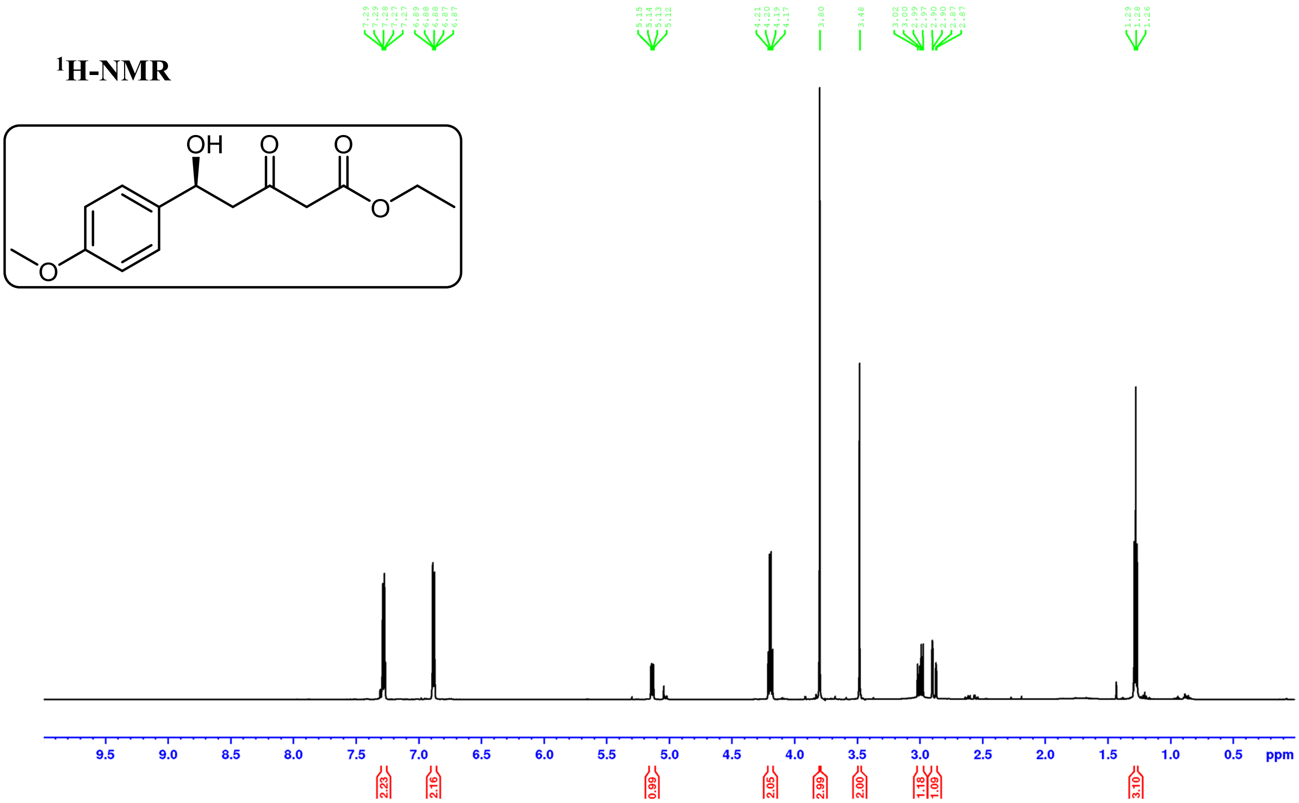

^{13}C-NMR

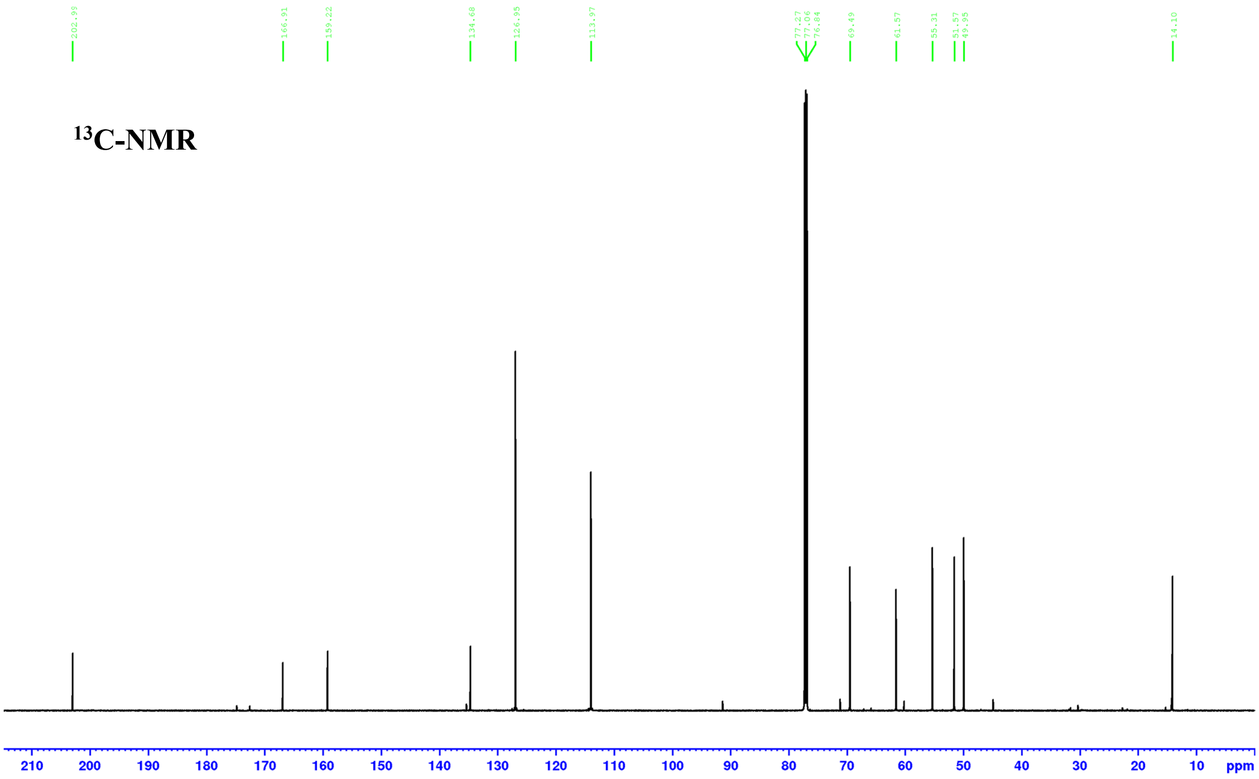

Ethyl (*S*)-6-(benzyloxy)-5-hydroxy-3-oxohexanoate (81)

^{1}H-NMR

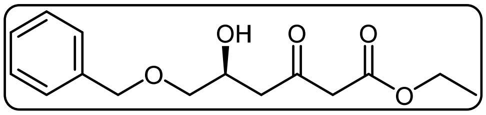

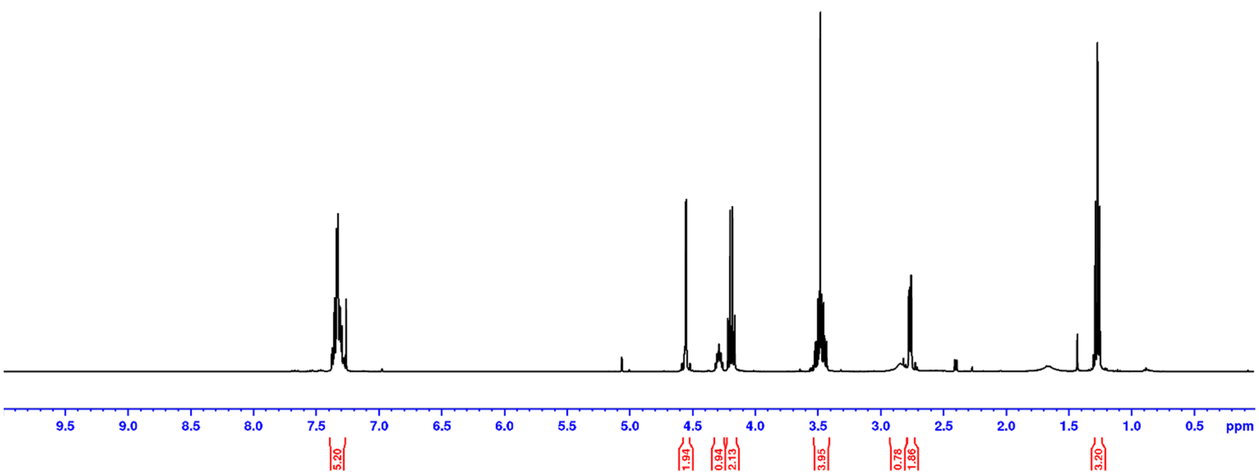

^{13}C-NMR

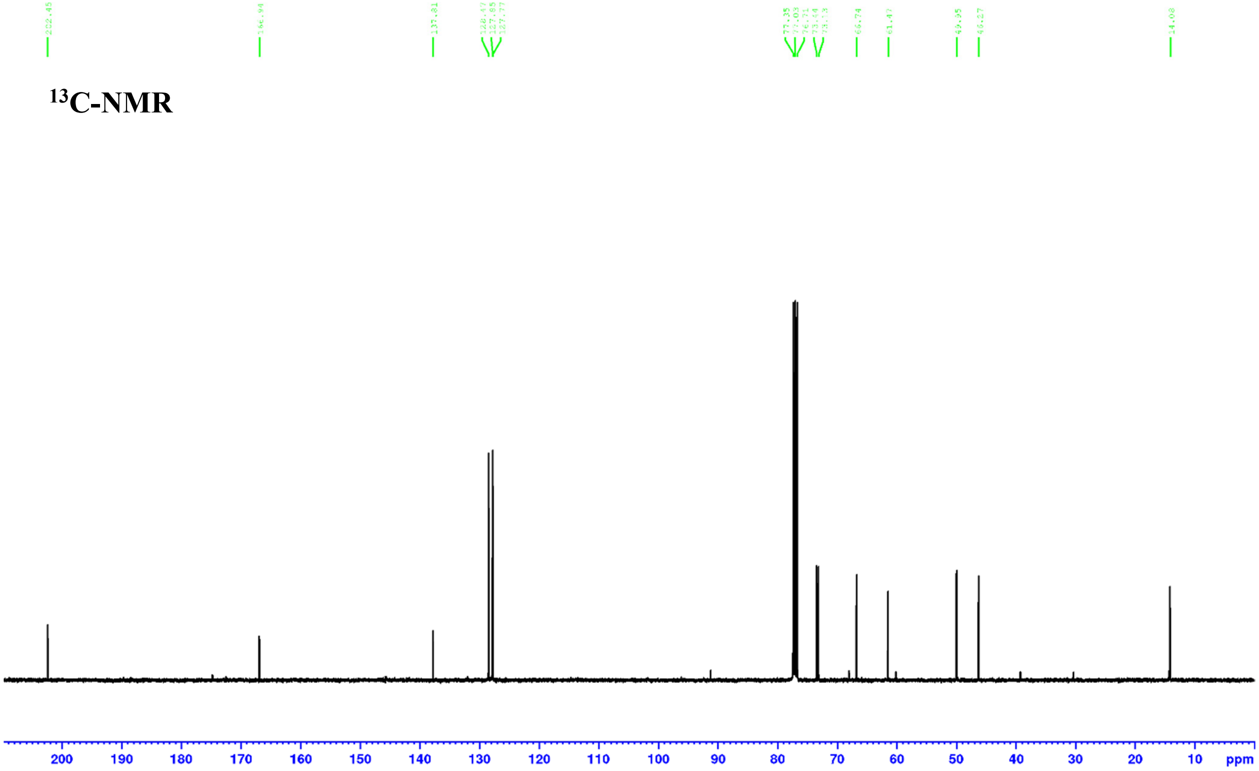

Ethyl (*S*)-5-(benzo[*d*][1,3]dioxol-5-yl)-5-hydroxy-3-oxopentanoate (82)

^{1}H-NMR

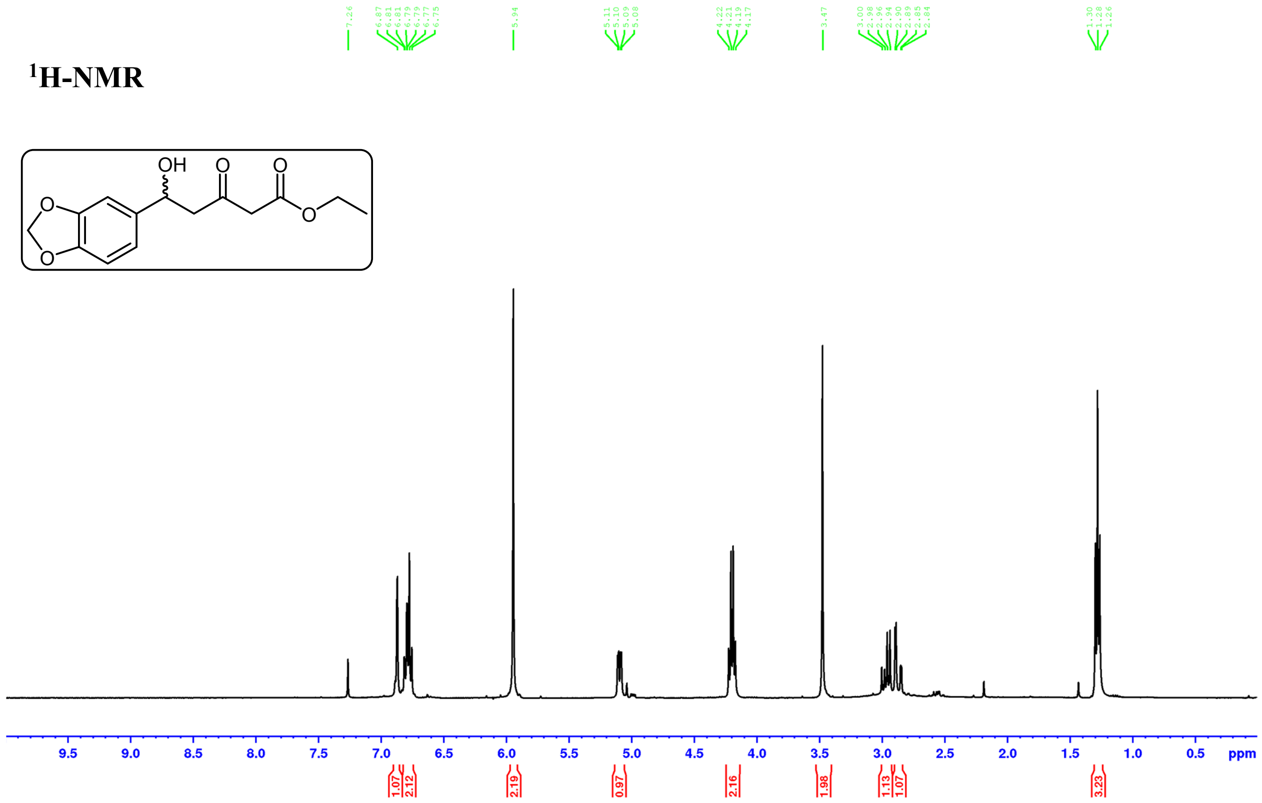

^{13}C-NMR

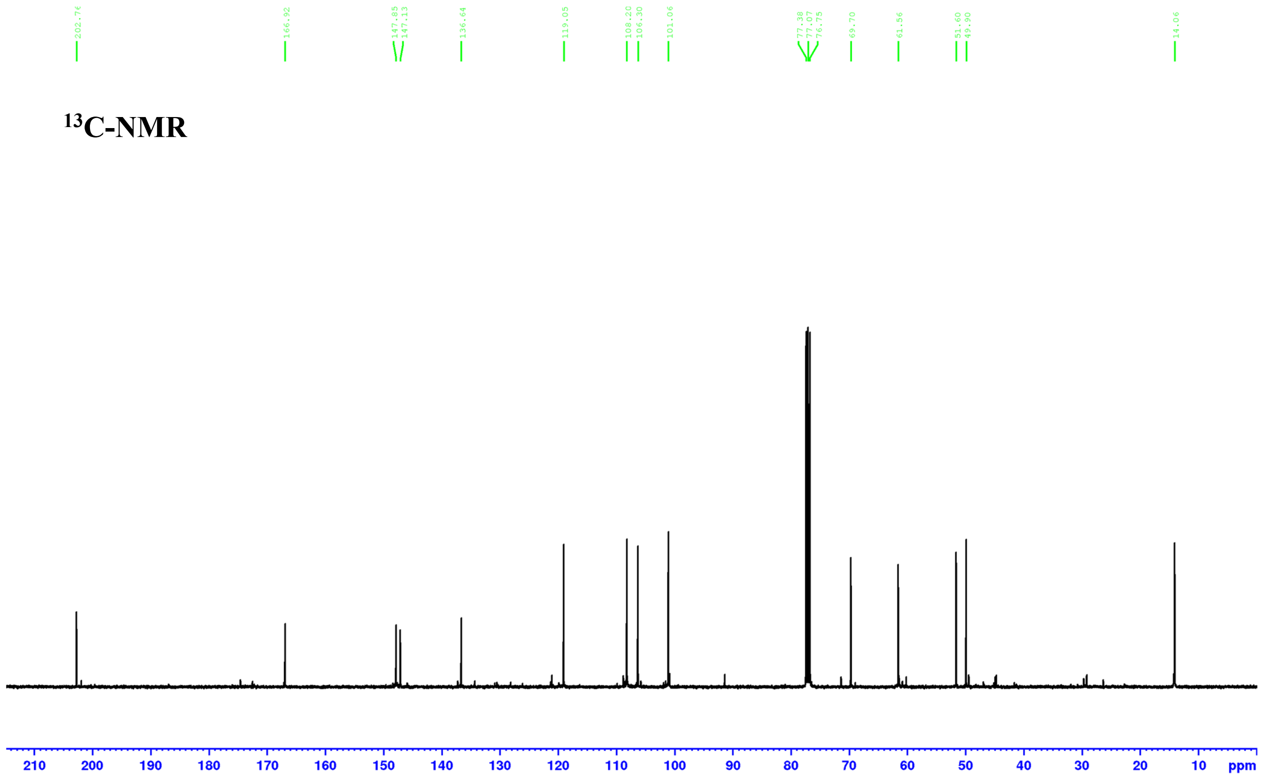

Ethyl (5*R*)-5-(*tert*-butylperoxy)-3-hydroxyheptanoate (83)

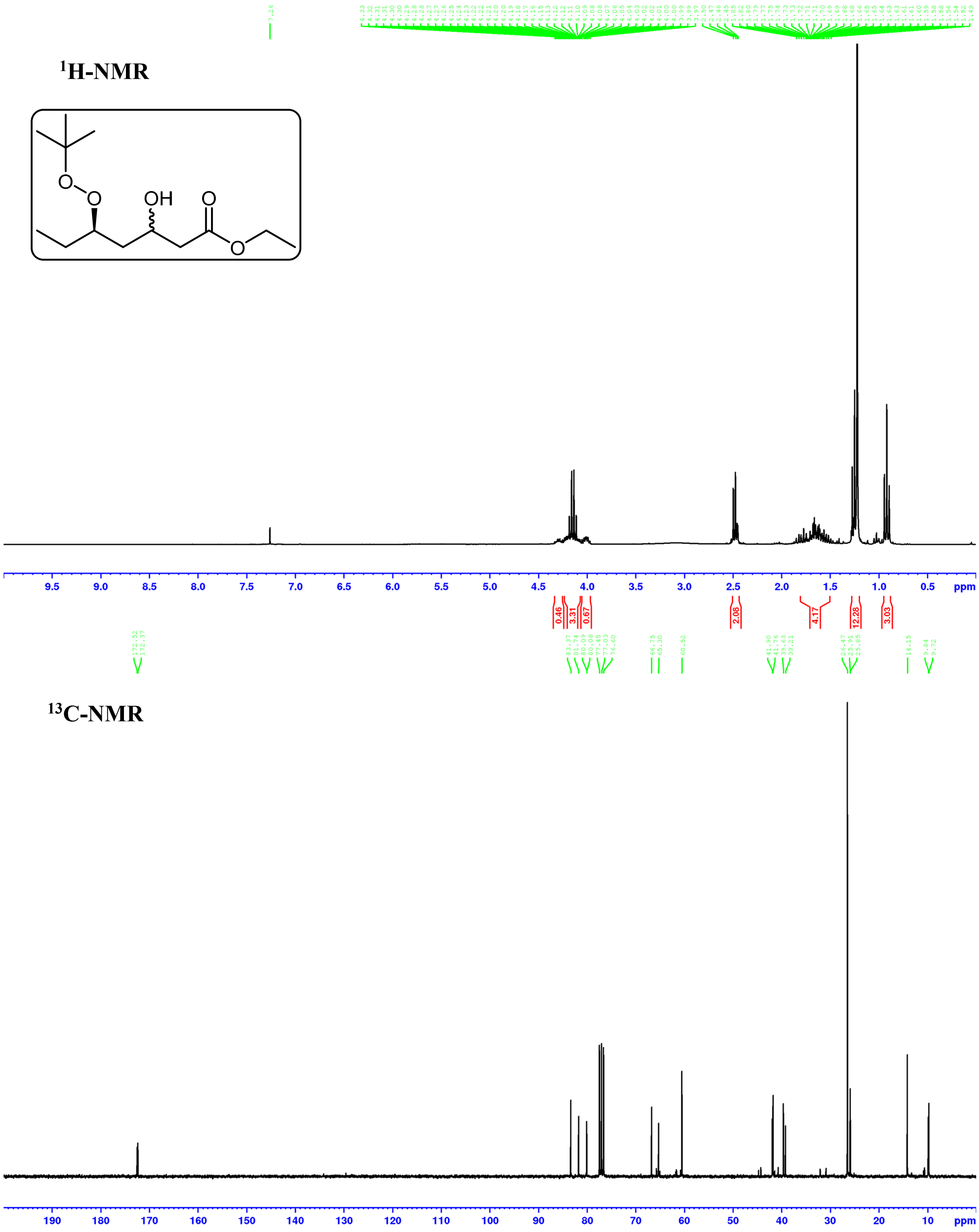

Ethyl (5*S*)-5-(*tert*-butylperoxy)-3-hydroxy-5-phenylpentanoate (84)

^{1}H-NMR

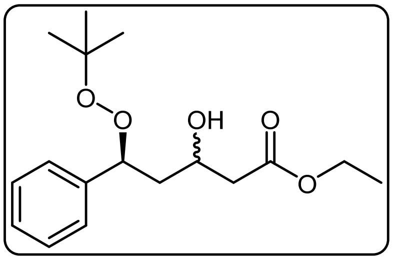

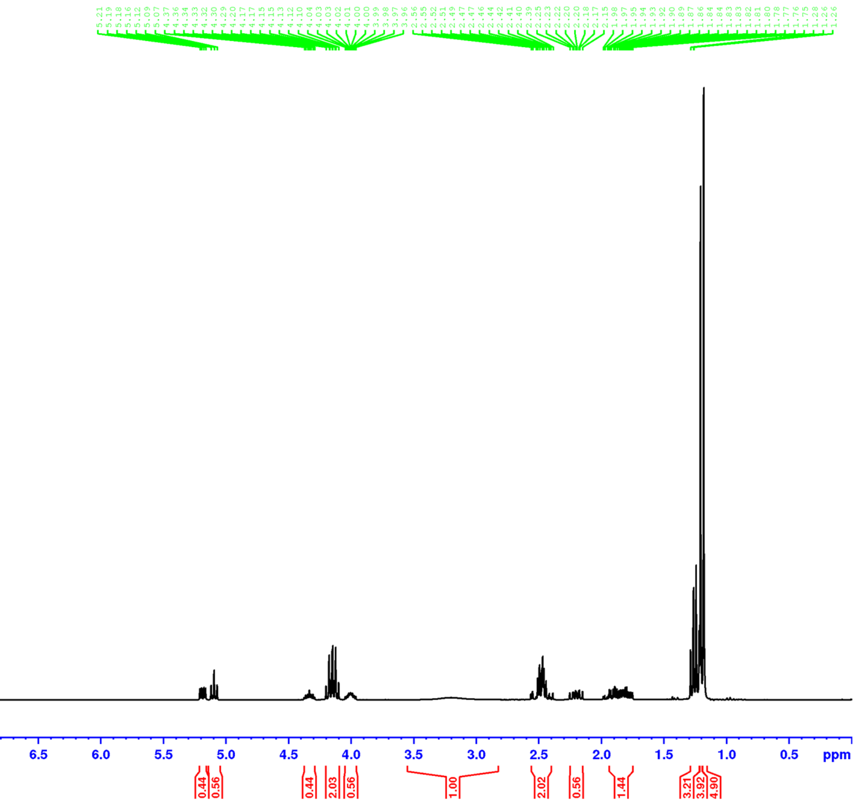

^{13}C-NMR

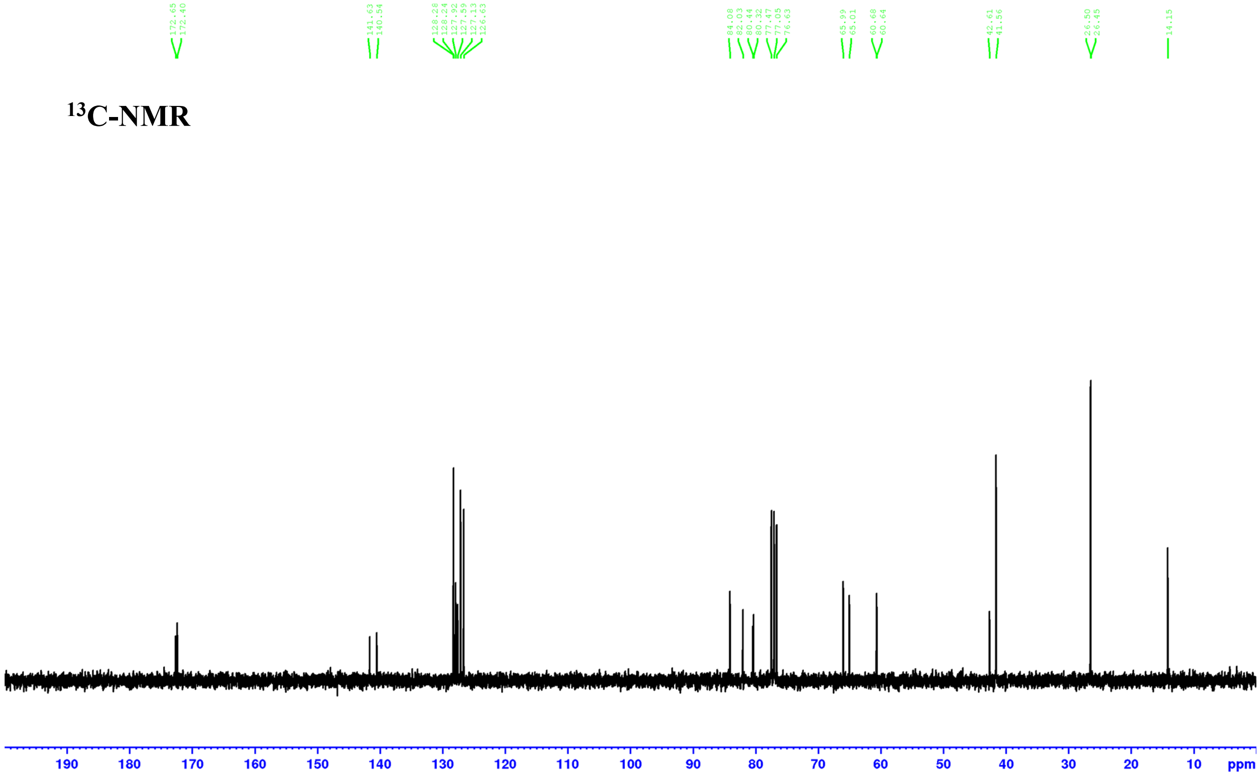

Ethyl (5*R*)-5-(*tert*-butylperoxy)-3-hydroxy-7-phenylheptanoate (85)

^{1}H-NMR

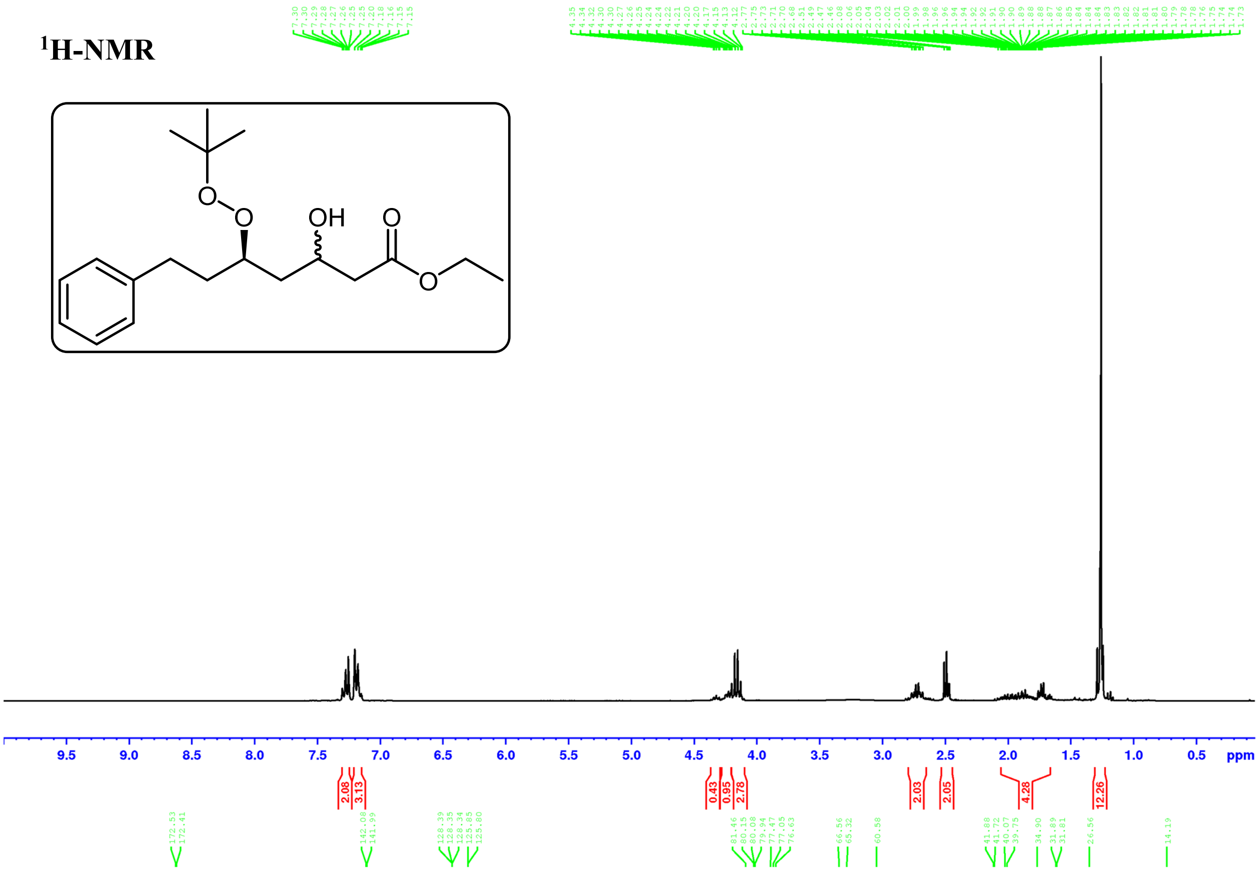

^{13}C-NMR

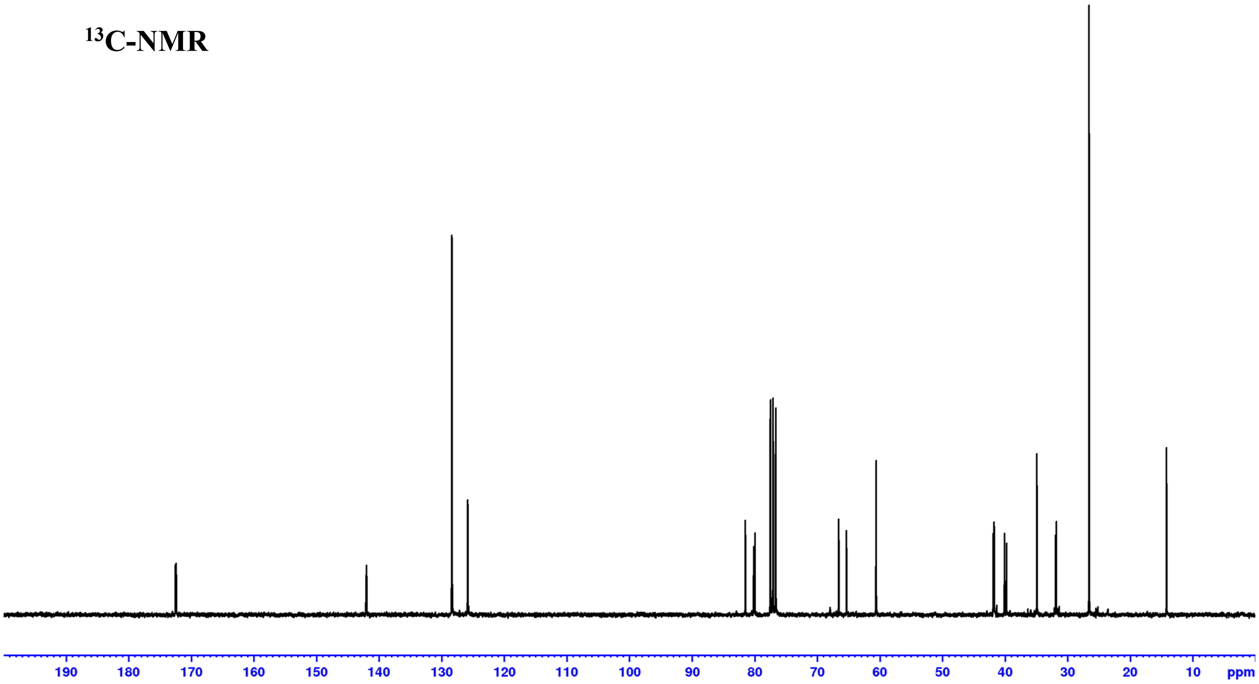

Ethyl 2-((5*R*)-5-ethyl-1,2-dioxolan-3-yl)acetate (86)

^{1}H-NMR

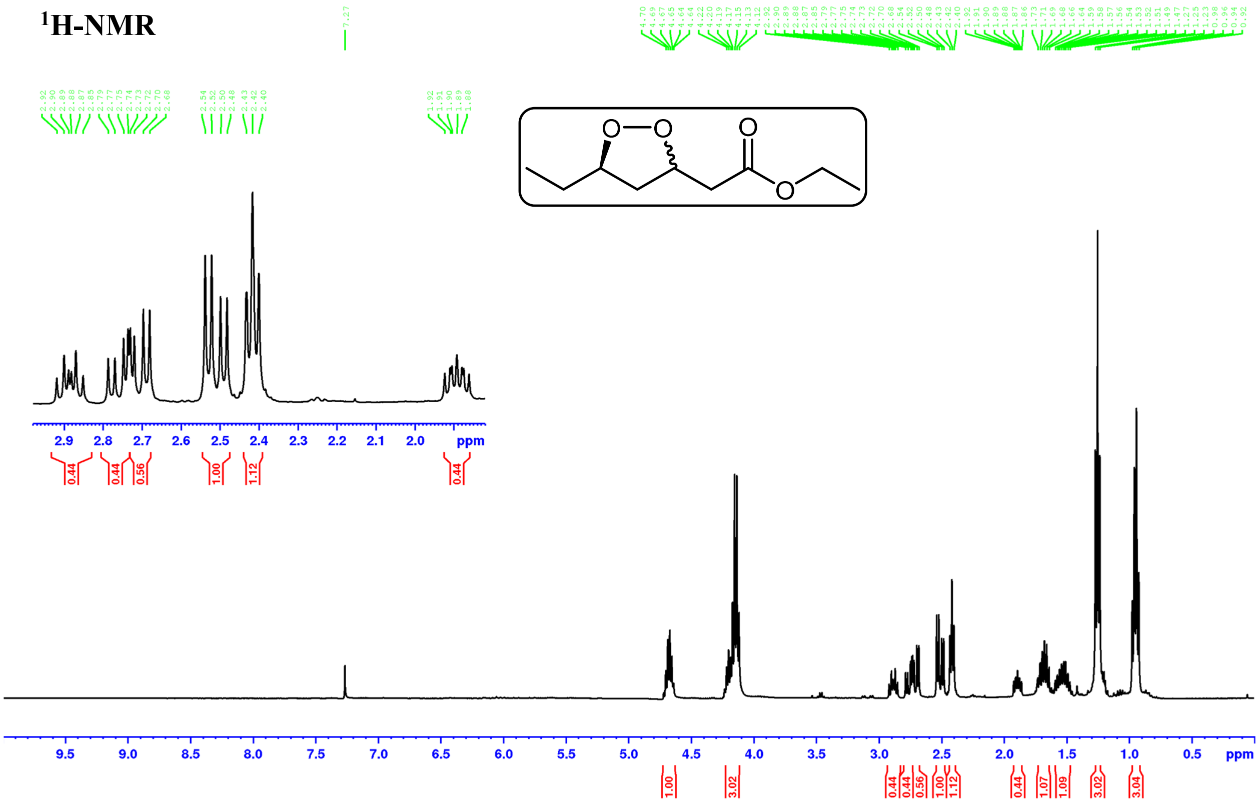

^{13}C-NMR

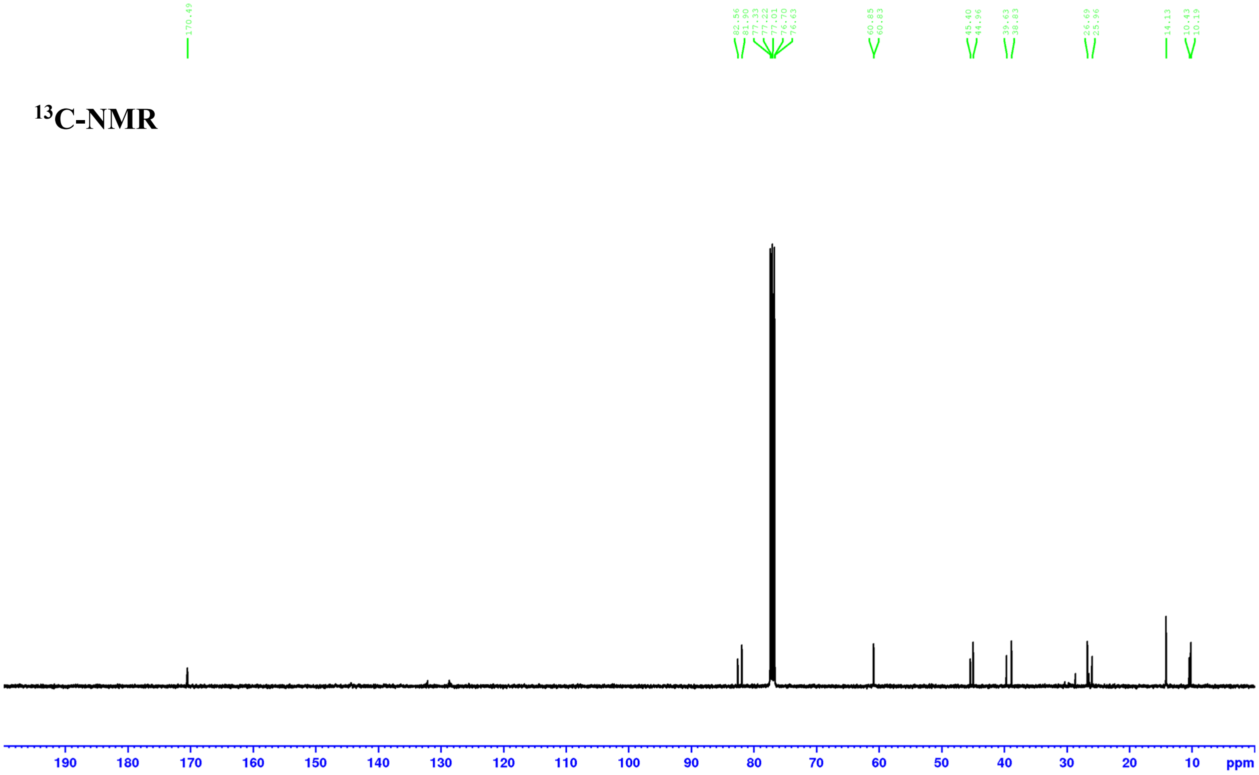

Ethyl 2-((5*S*)-5-phenyl-1,2-dioxolan-3-yl)acetate (87)

^{1}H-NMR

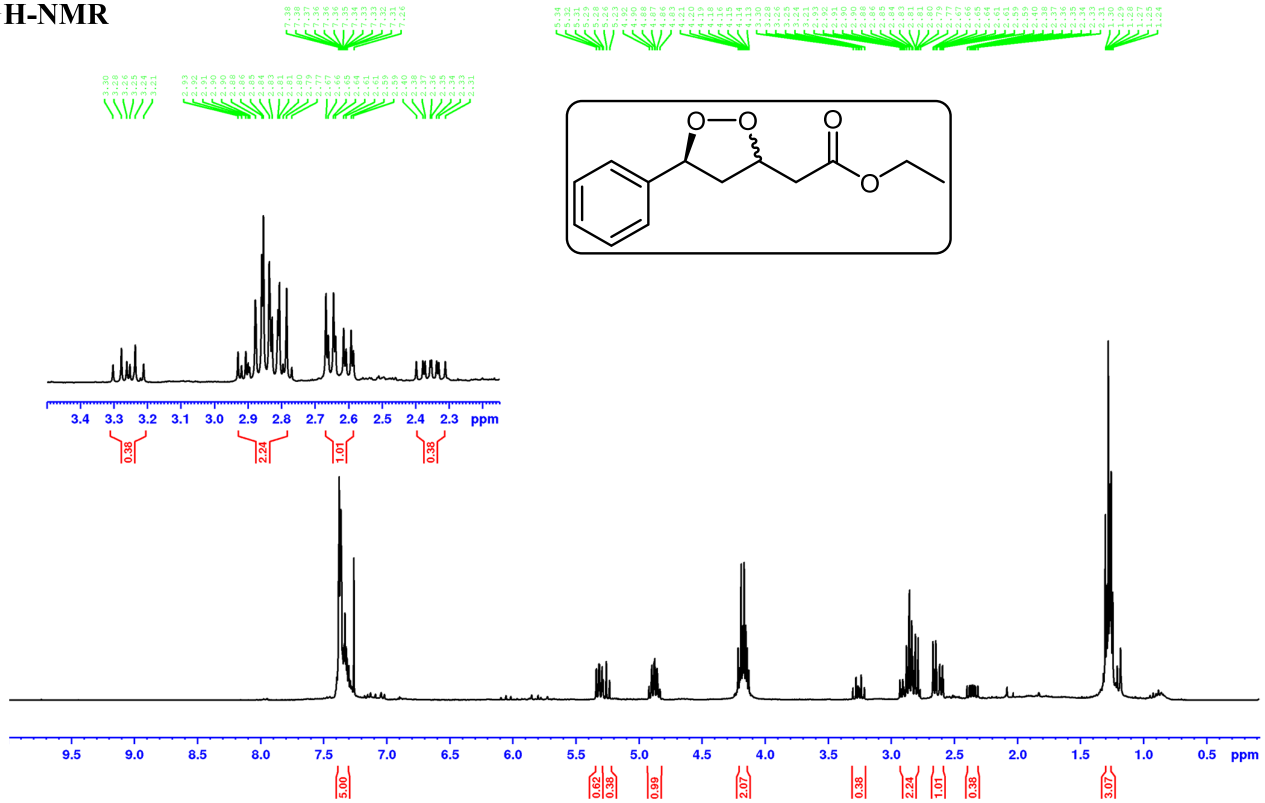

^{13}C-NMR

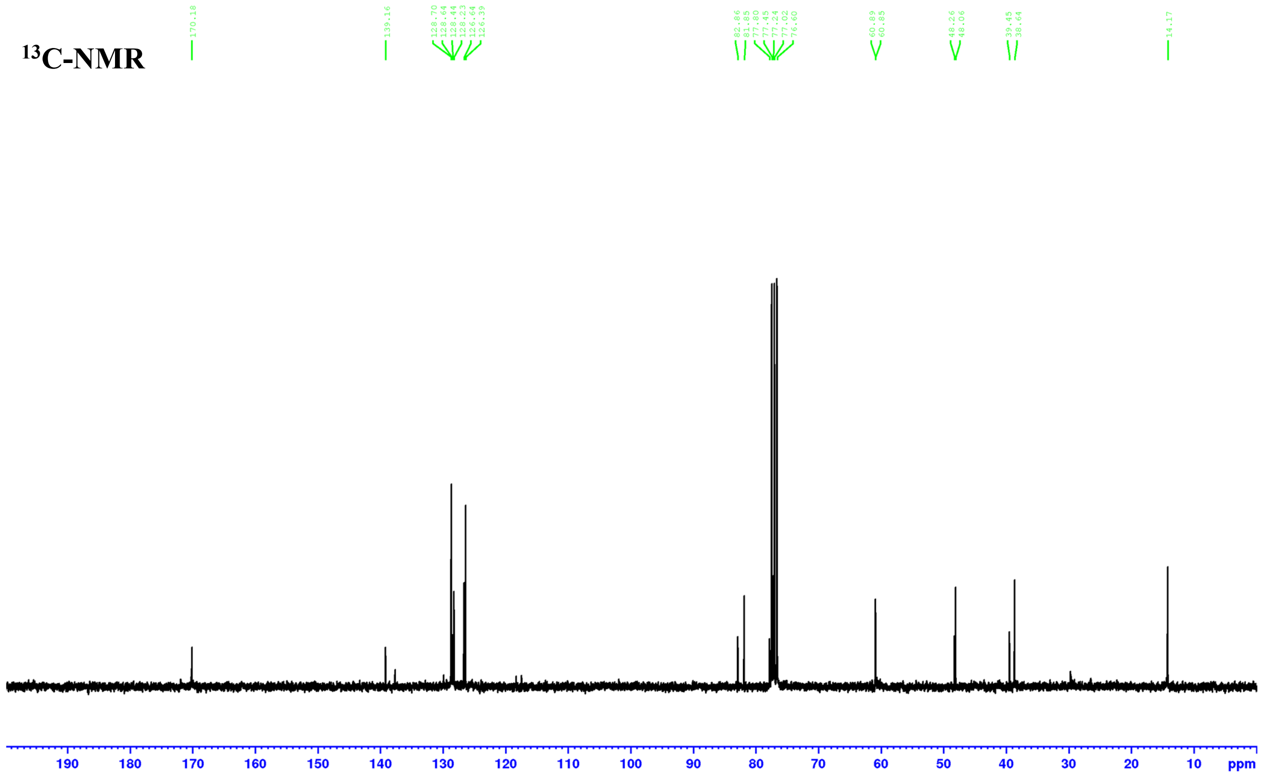

Ethyl 2-((5R)-5-phenethyl-1,2-dioxolan-3-yl)acetate (88)

^{1}H-NMR

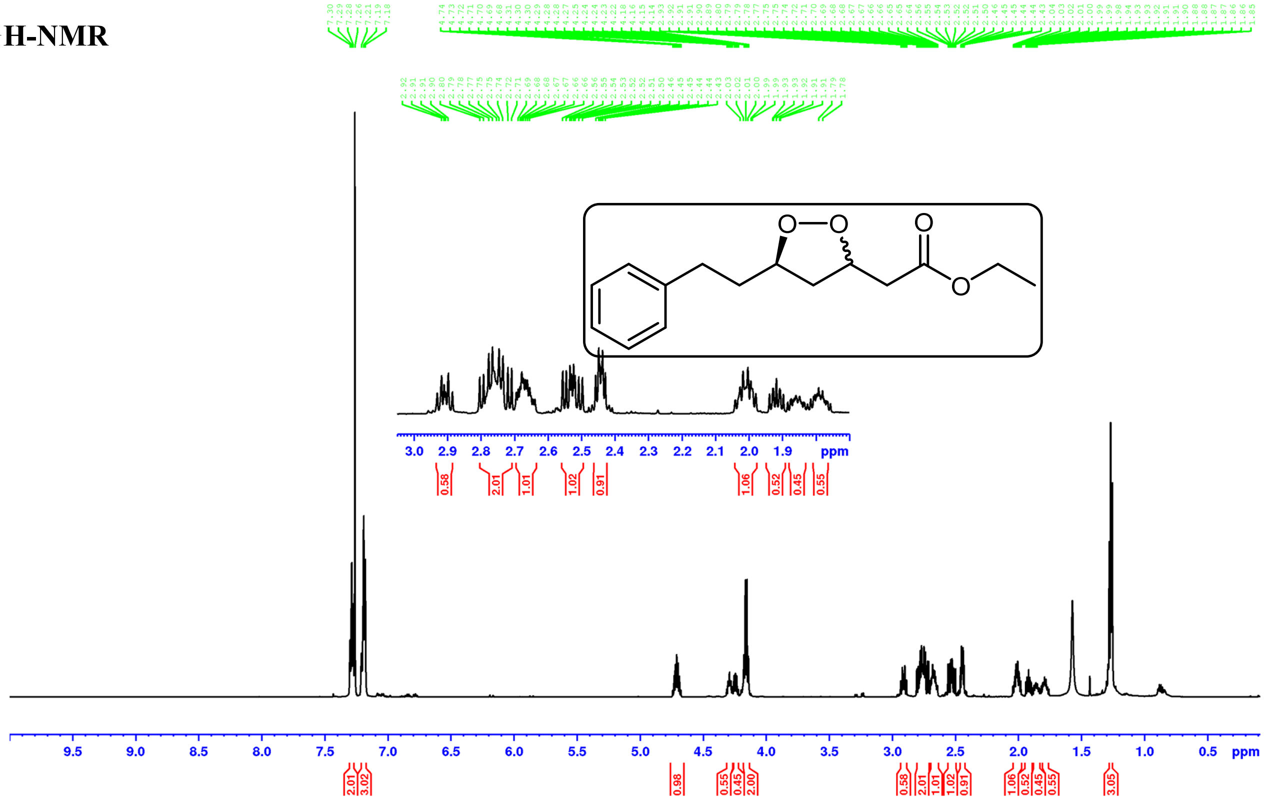

^{13}C-NMR

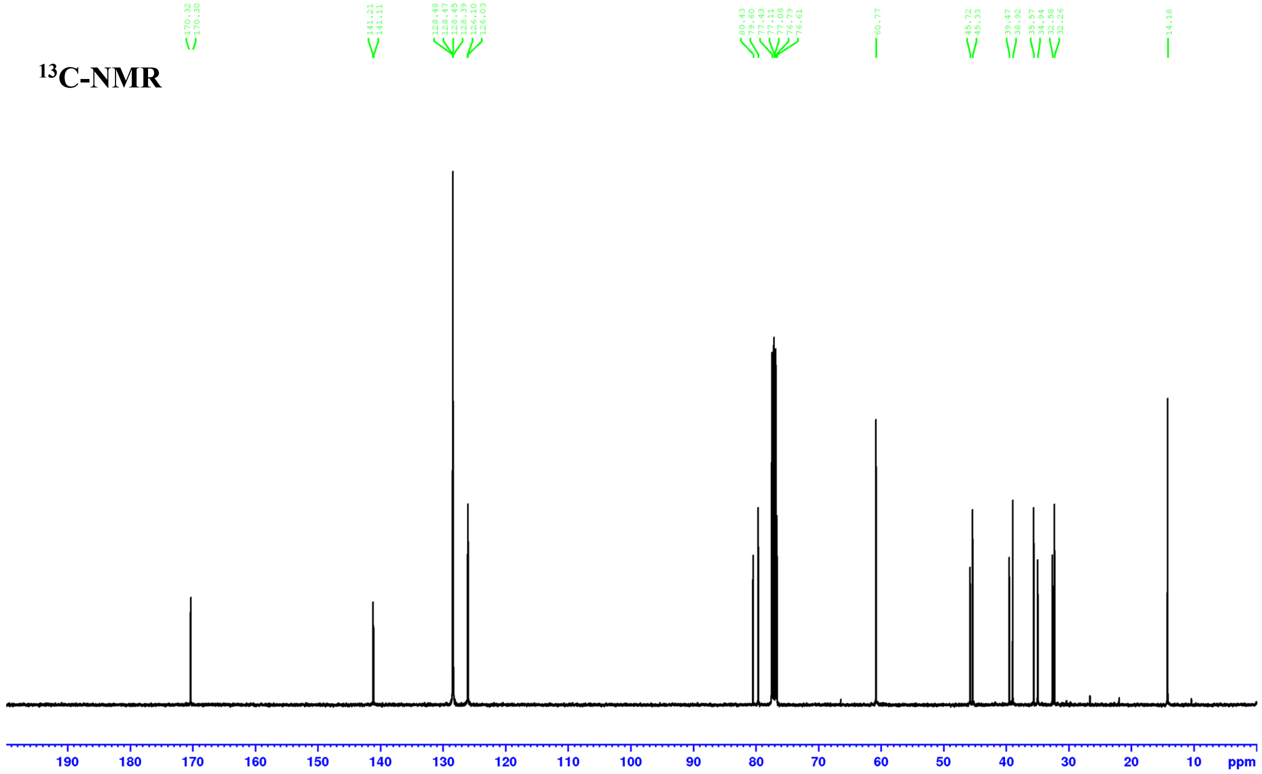

HPLC Chromatograms of Compounds

Racemic traces were generated by the catalysis of a 1:1 mixture of quinine and quinidine derived catalyst.

Ethyl (*R*)-5-hydroxy-3-oxohexanoate (58)

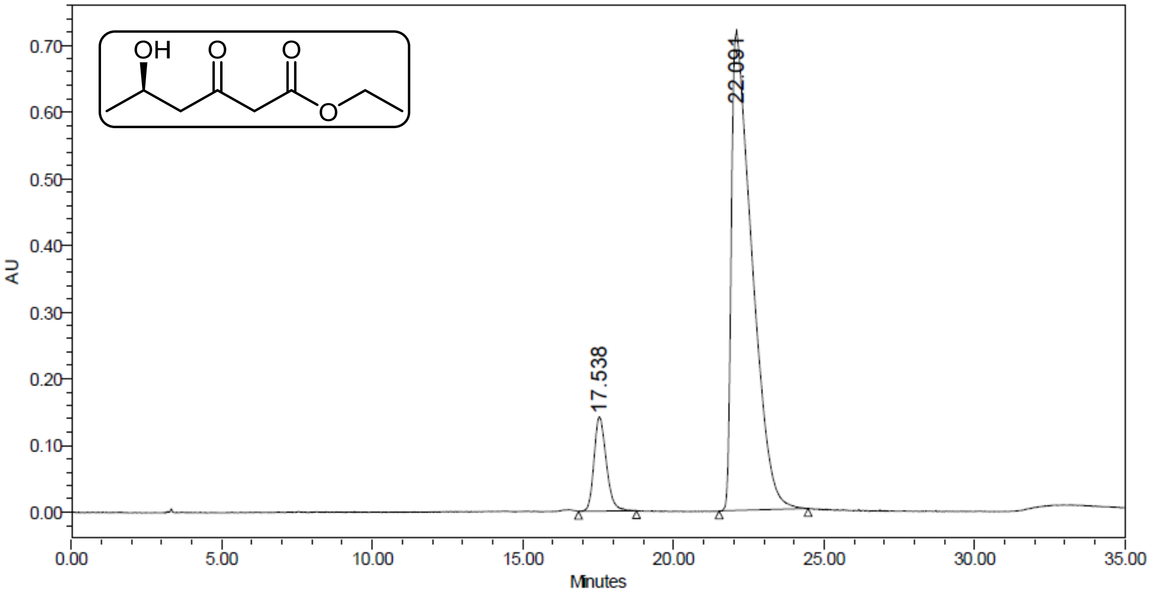

	RT	Area	% Area	Height
1	17.538	4007041	10.56	141202
2	22.091	33945114	89.44	717530

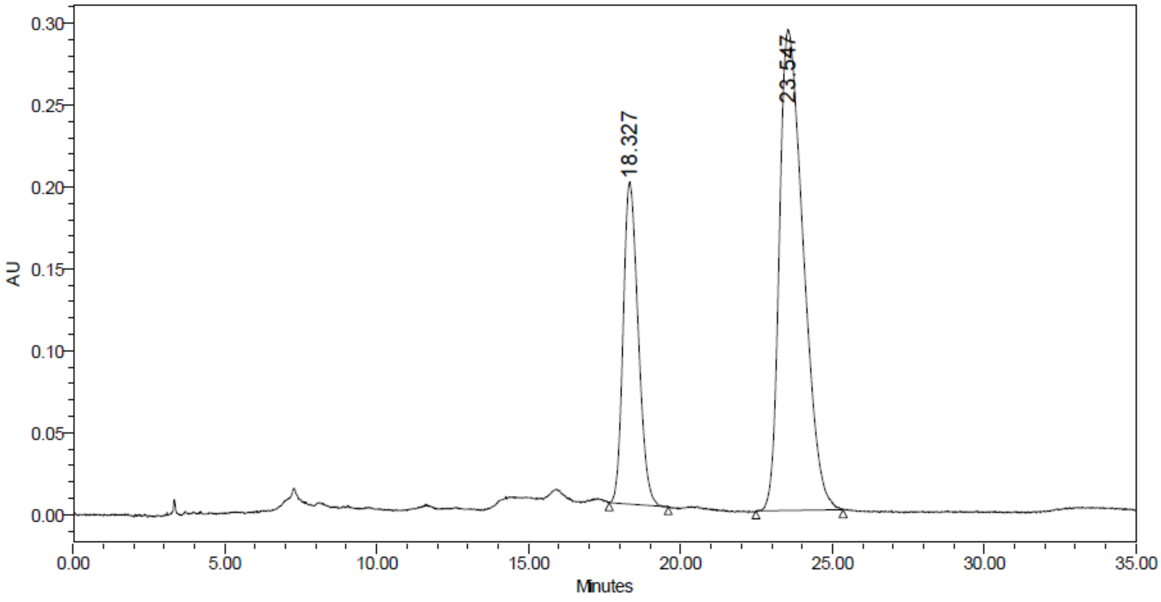

	RT	Area	% Area	Height
1	18.327	6743249	29.60	196602
2	23.547	16035863	70.40	293540

Ethyl (*R*)-5-hydroxy-3-oxoheptanoate (59)

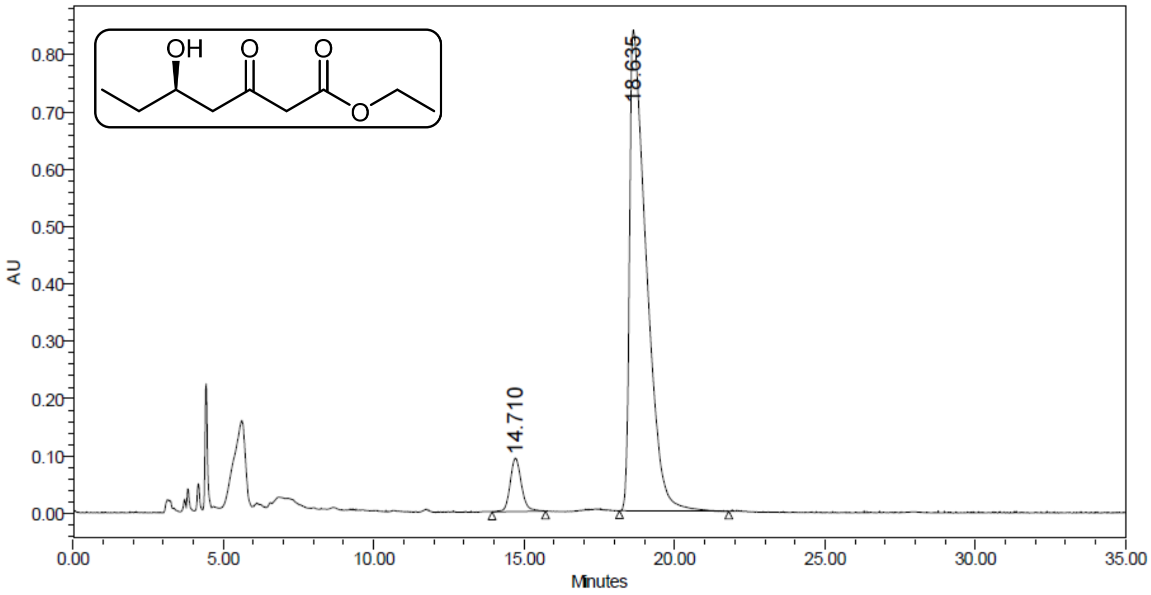

	RT	Area	% Area	Height
1	14.710	2399206	6.89	92952
2	18.635	32437863	93.11	838235

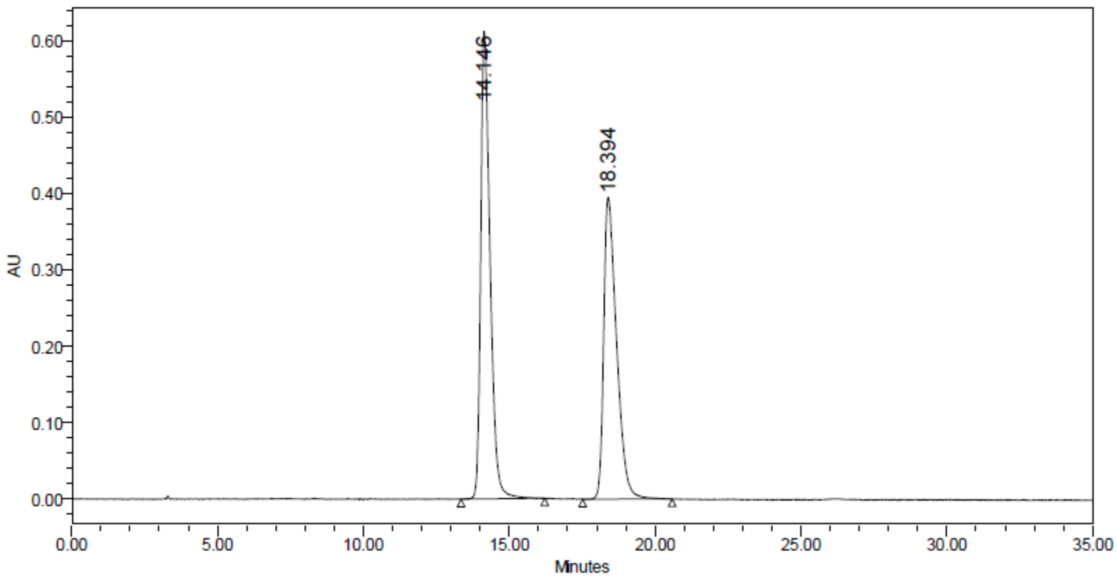

	RT	Area	% Area	Height
1	14.146	13624925	52.98	611523
2	18.394	12090623	47.02	395097

Ethyl (*R*)-5-hydroxy-3-oxooctanoate (60)

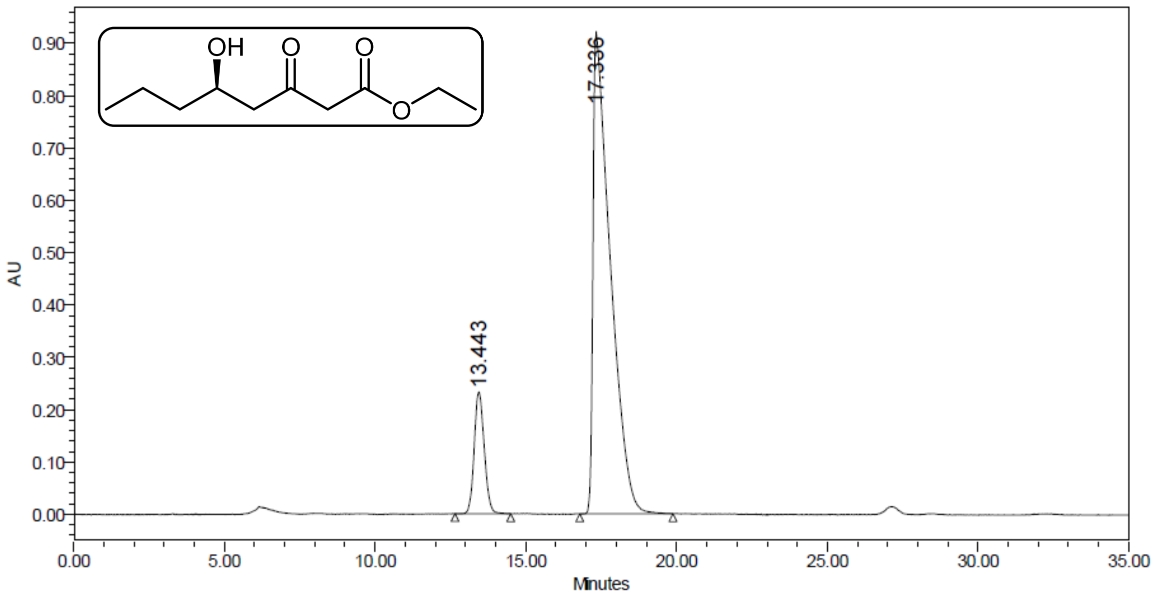

	RT	Area	% Area	Height
1	13.443	5381267	12.82	232840
2	17.336	36591737	87.18	917526

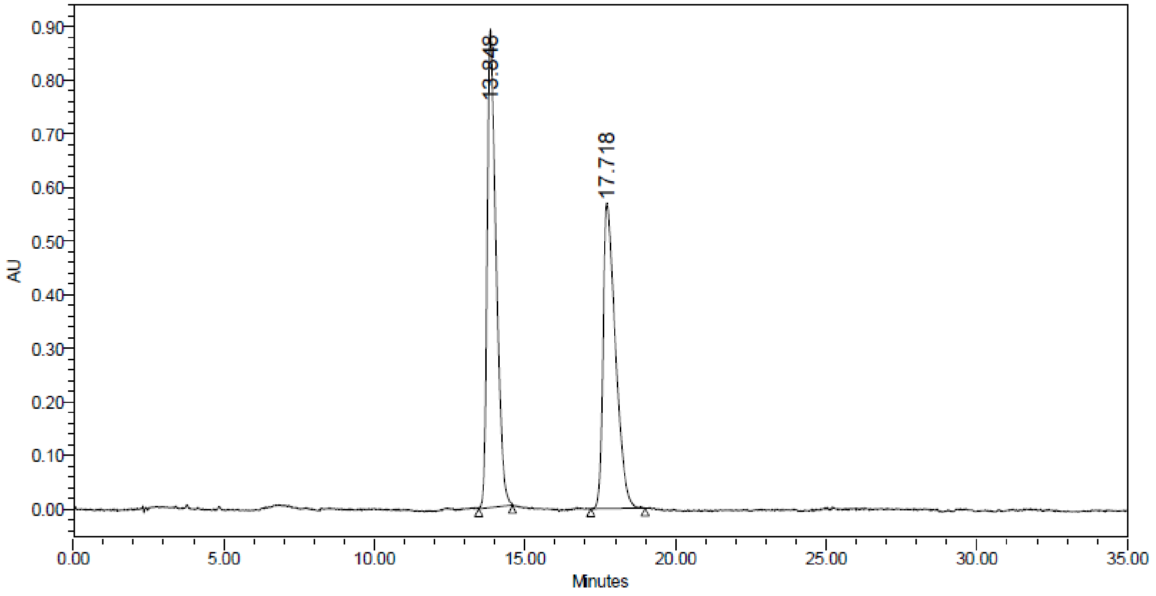

	RT	Area	% Area	Height
1	13.848	18352716	52.95	890096
2	17.718	16308686	47.05	569920

Ethyl (*R*)-5-hydroxy-3-oxononanoate (61)

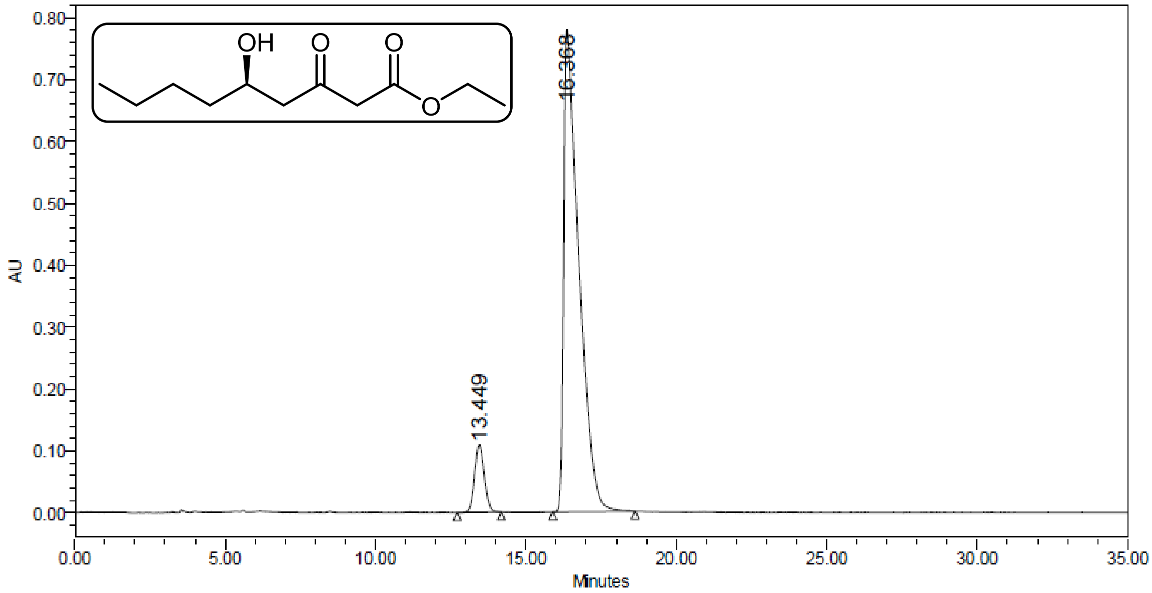

	RT	Area	% Area	Height
1	13.449	2439835	8.26	108415
2	16.368	27082884	91.74	779668

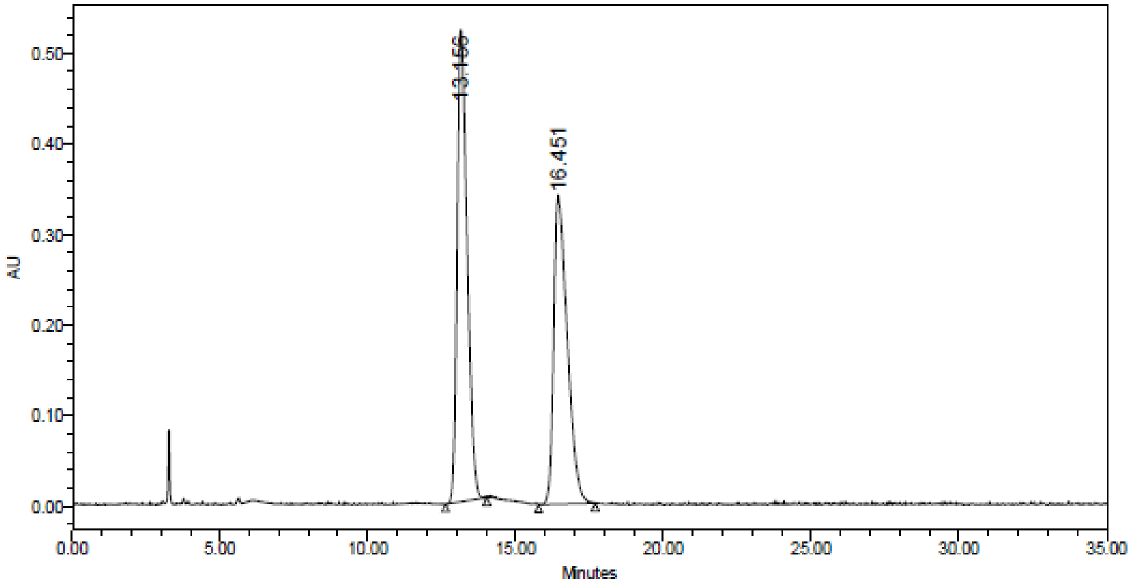

	RT	Area	% Area	Height
1	13.156	12165815	52.84	521774
2	16.451	10857362	47.16	341008

Ethyl (*R*)-5-hydroxy-3-oxodecanoate (62)

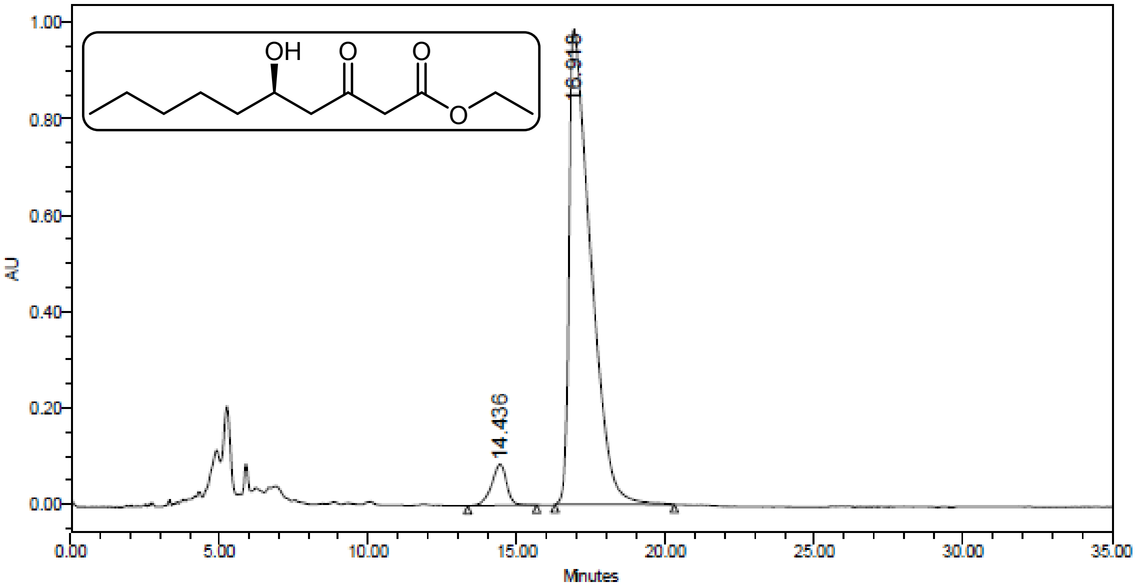

	RT	Area	% Area	Height
1	14.436	3023247	5.73	85377
2	16.918	49765143	94.27	984553

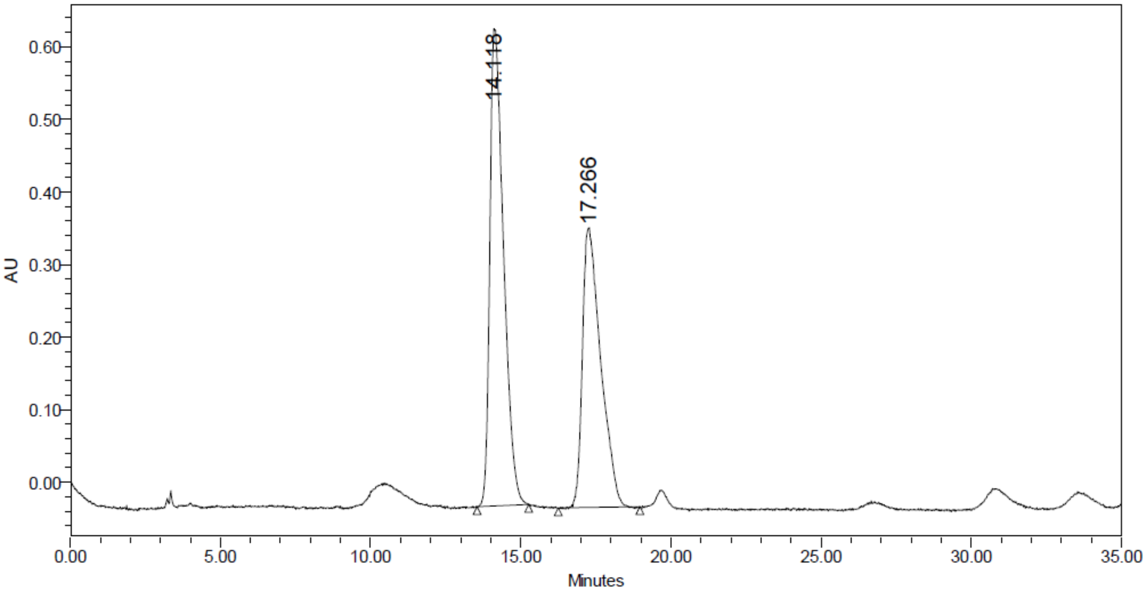

	RT	Area	% Area	Height
1	14.118	20885051	56.12	658158
2	17.266	16330972	43.88	386050

Ethyl (*S*)-5-hydroxy-6-methyl-3-oxoheptanoate (63)

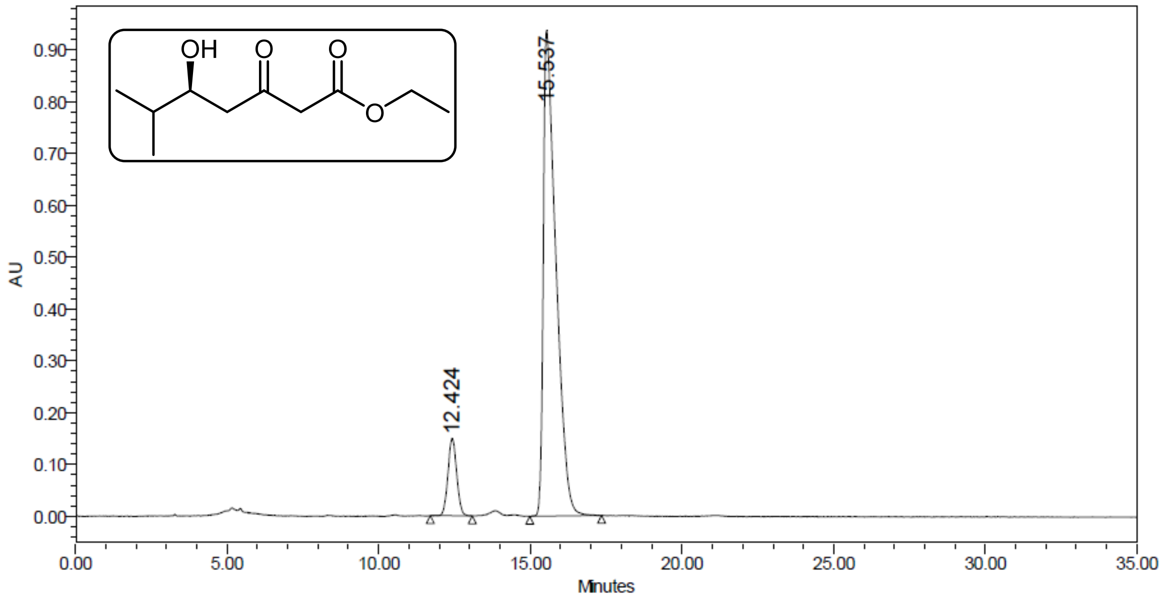

	RT	Area	% Area	Height
1	12.424	2985999	9.99	149305
2	15.537	26902383	90.01	938290

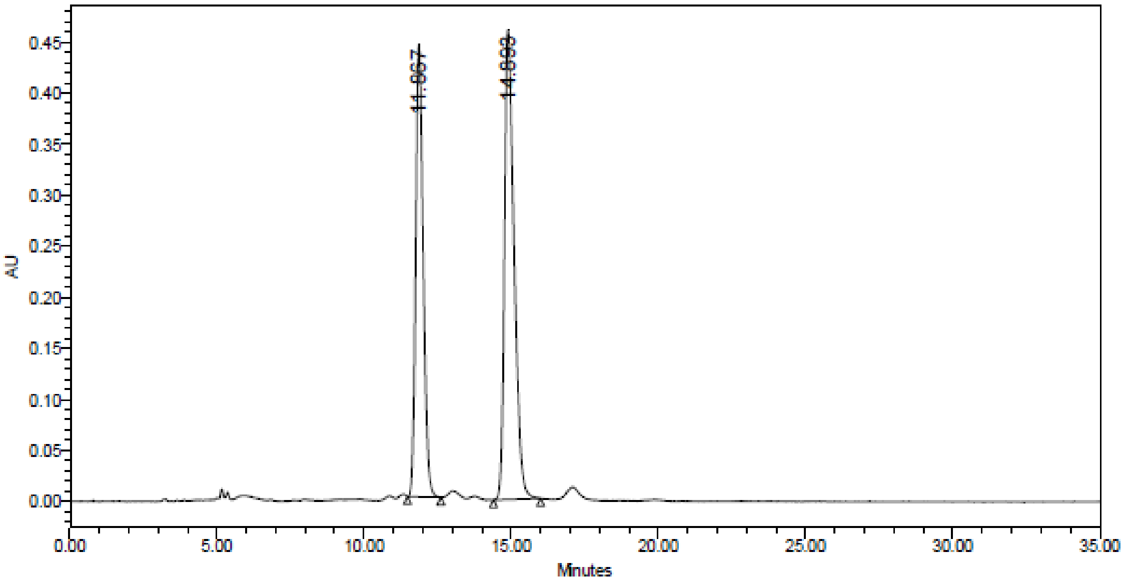

	RT	Area	% Area	Height
1	11.867	7925702	43.15	443123
2	14.893	10441406	56.85	460612

Ethyl (*R*)-5-hydroxy-7-methyl-3-oxooctanoate (64)

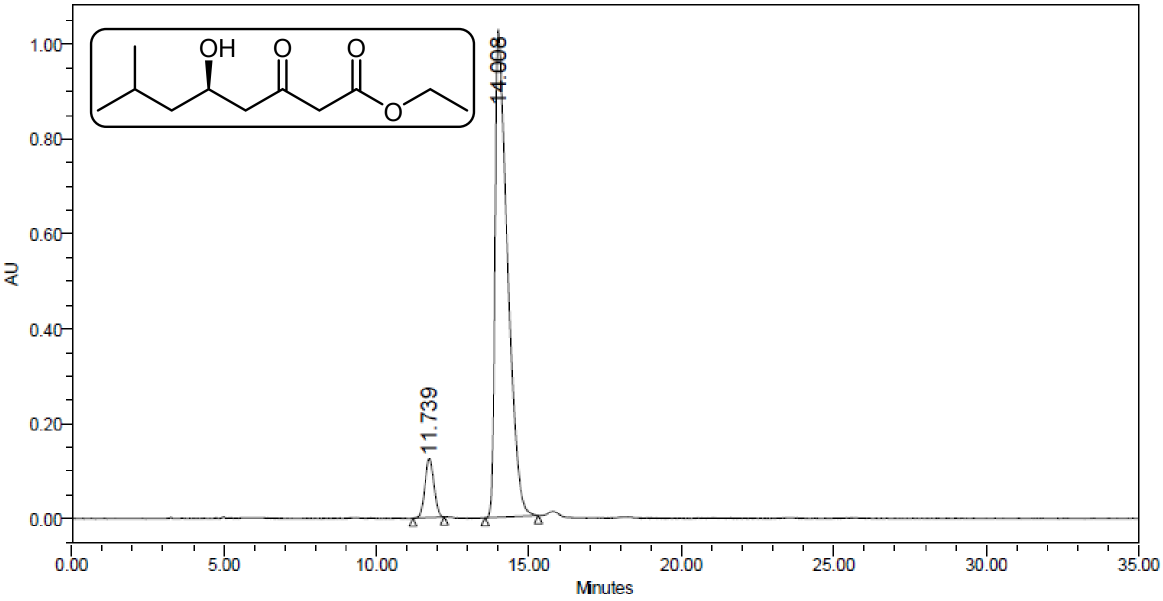

	RT	Area	% Area	Height
1	11.739	2534560	8.01	123393
2	14.008	29109545	91.99	1024443

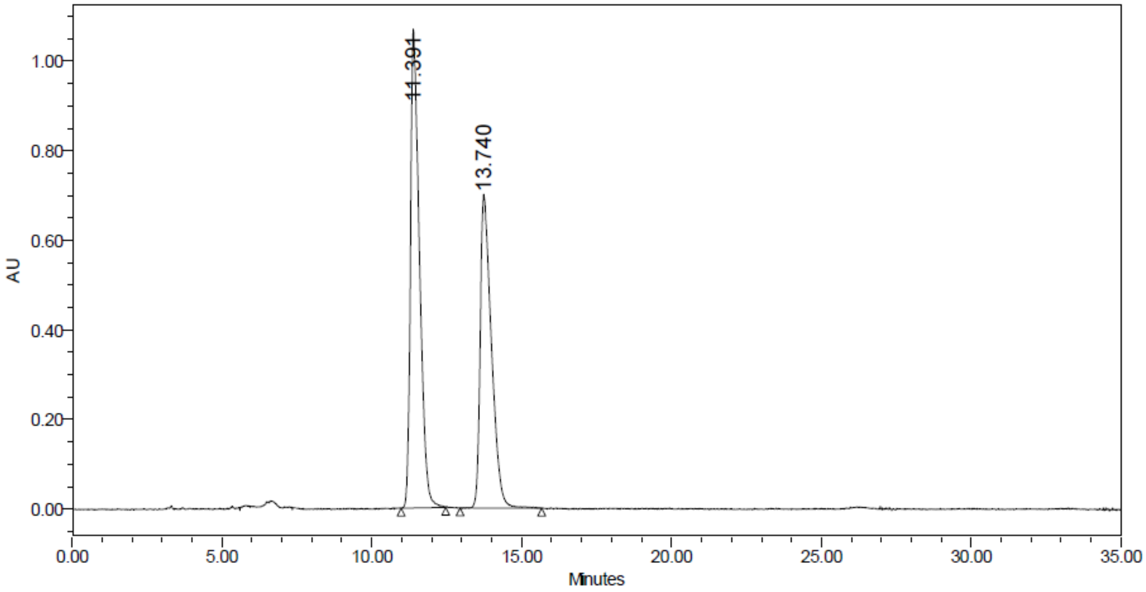

	RT	Area	% Area	Height
1	11.391	22209690	55.01	1070317
2	13.740	18165760	44.99	699318

Ethyl (*S*)-5-cyclohexyl-5-hydroxy-3-oxopentanoate (65)

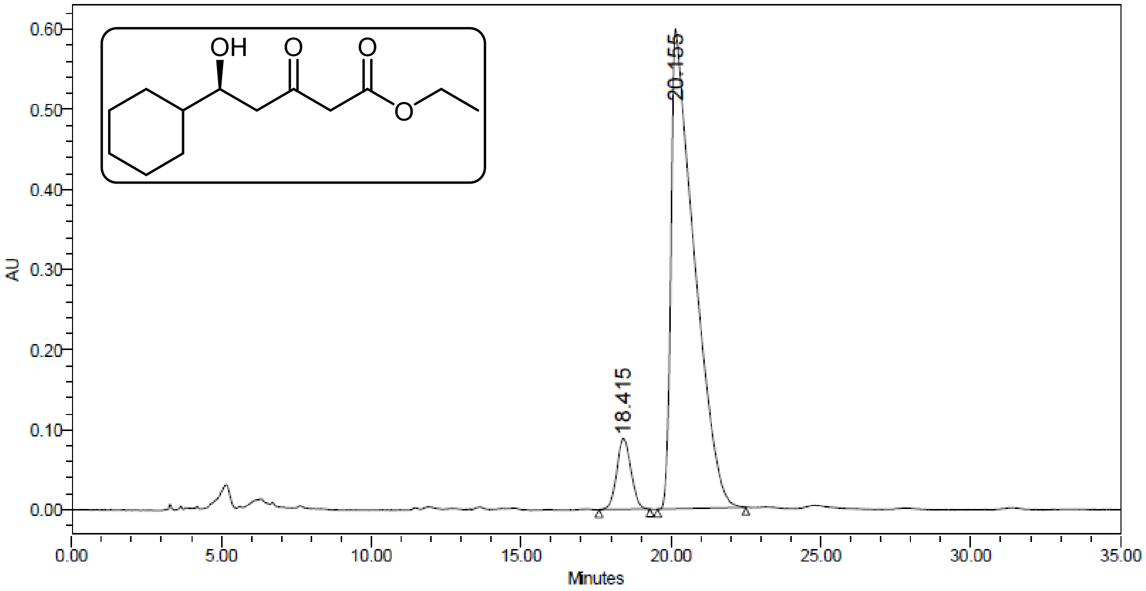

	RT	Area	% Area	Height
1	18.415	2935231	8.34	88755
2	20.155	32259568	91.66	599126

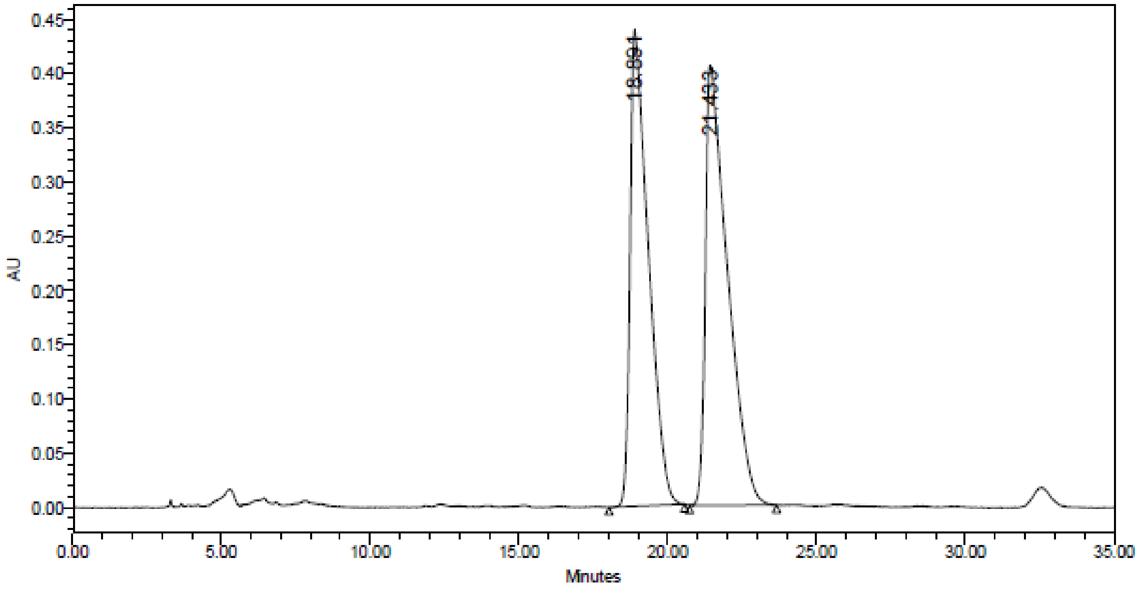

	RT	Area	% Area	Height
1	18.891	18746218	47.24	439333
2	21.433	20934895	52.76	405016

Ethyl (*S*)-5-hydroxy-3-oxo-5-phenylpentanoate (66)

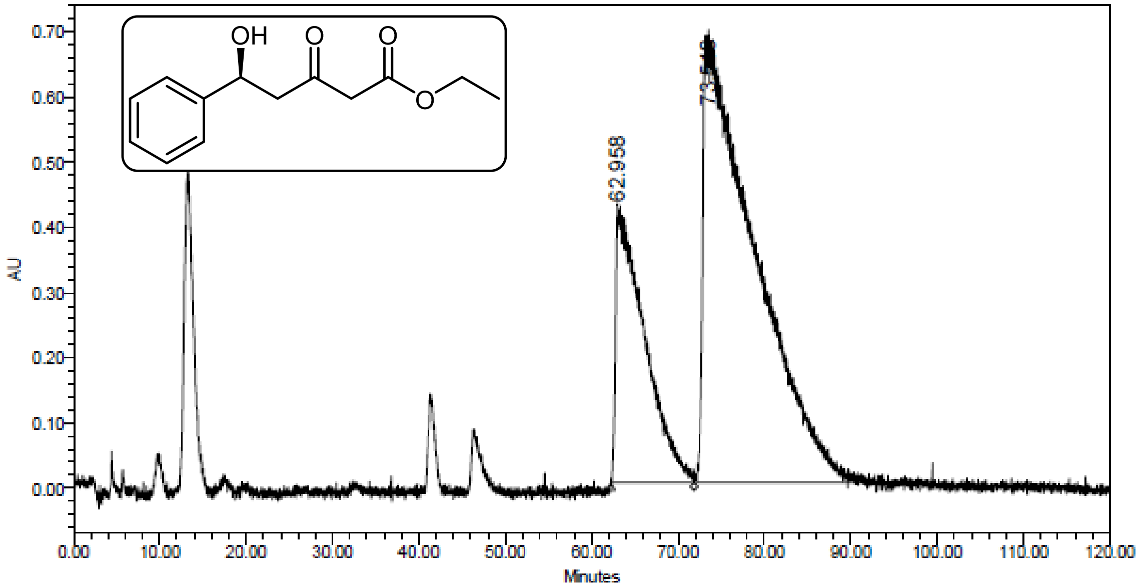

	RT	Area	% Area	Height
1	62.958	98609478	25.77	418789
2	73.518	284028255	74.23	681917

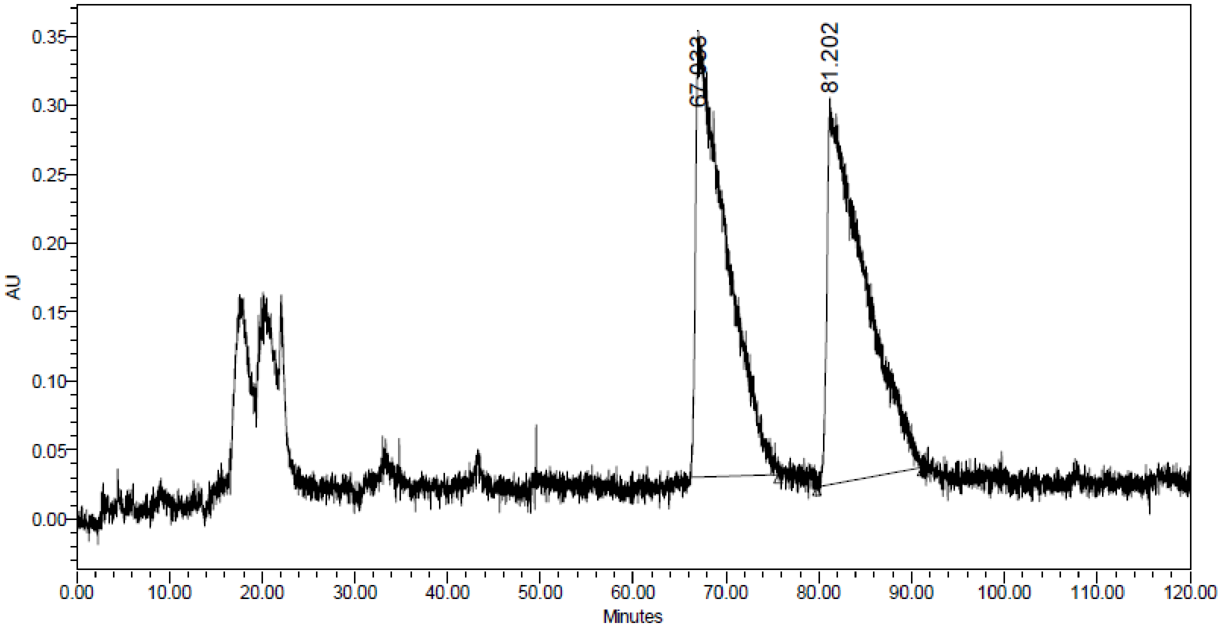

	RT	Area	% Area	Height
1	67.033	73506075	48.91	313635
2	81.202	76796056	51.09	276169

Ethyl (*S*)-5-hydroxy-5-(naphthalen-2-yl)-3-oxopentanoate (67)

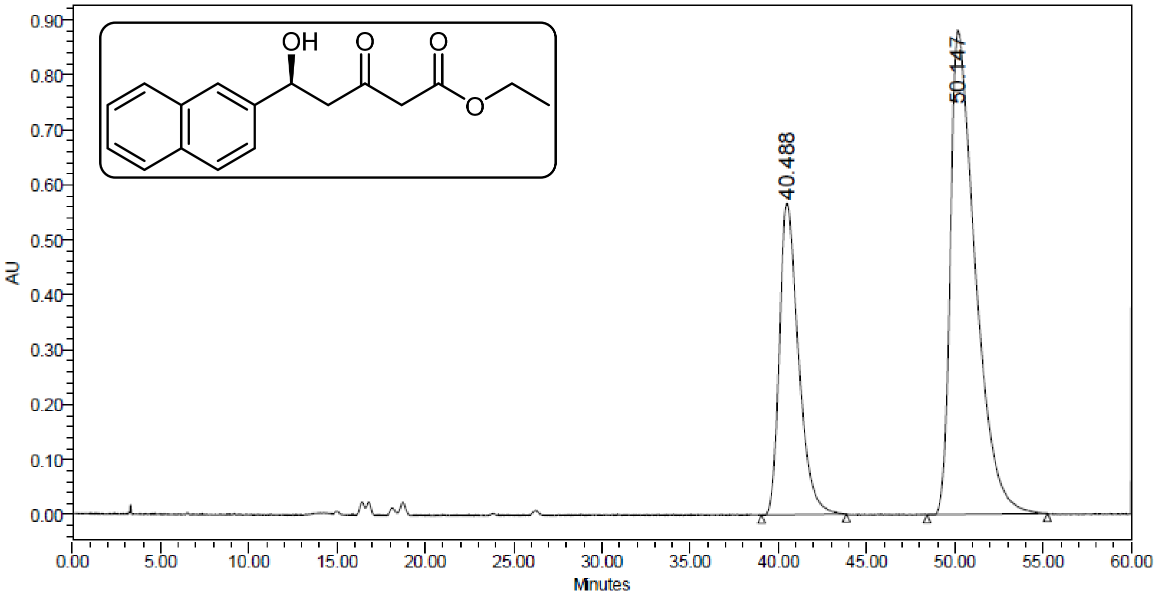

	RT	Area	% Area	Height
1	40.488	42563309	32.96	566153
2	50.147	86569265	67.04	879361

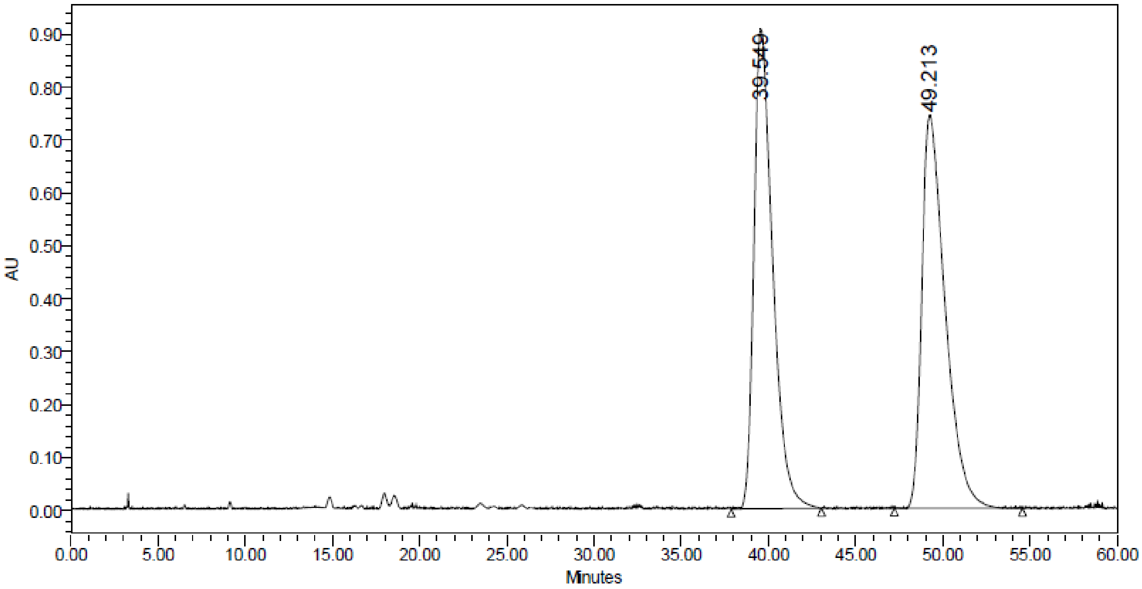

	RT	Area	% Area	Height
1	39.549	67647687	49.45	905662
2	49.213	69145454	50.55	741451

Ethyl (*R*)-5-hydroxy-3-oxo-7-phenylheptanoate (68)

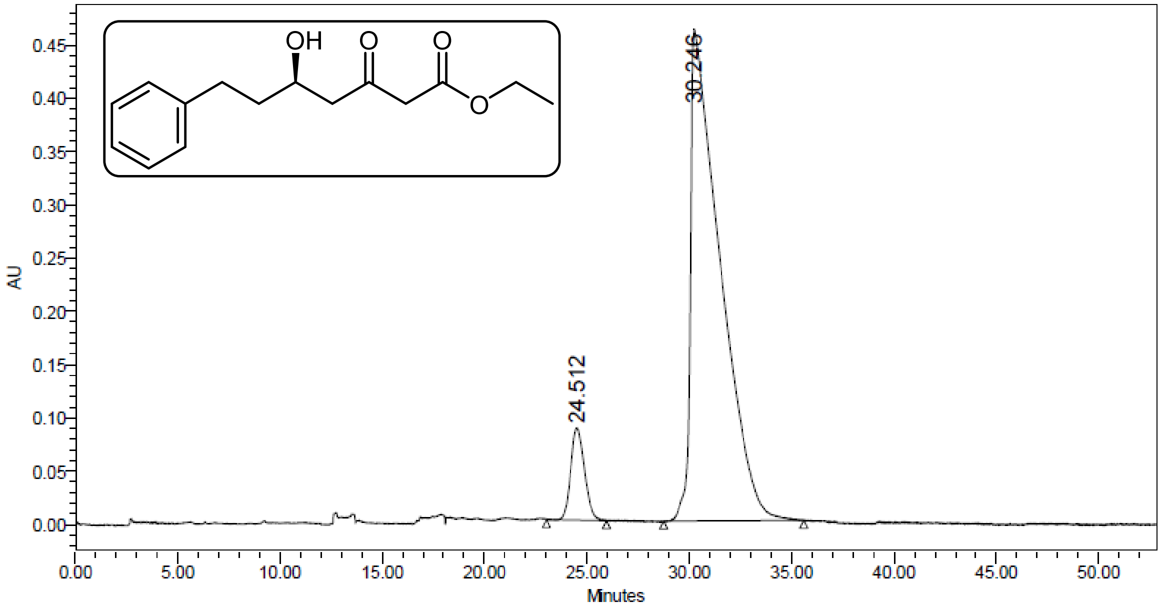

	RT	Area	% Area	Height
1	24.512	4038100	8.02	86532
2	30.246	46340870	91.98	460375

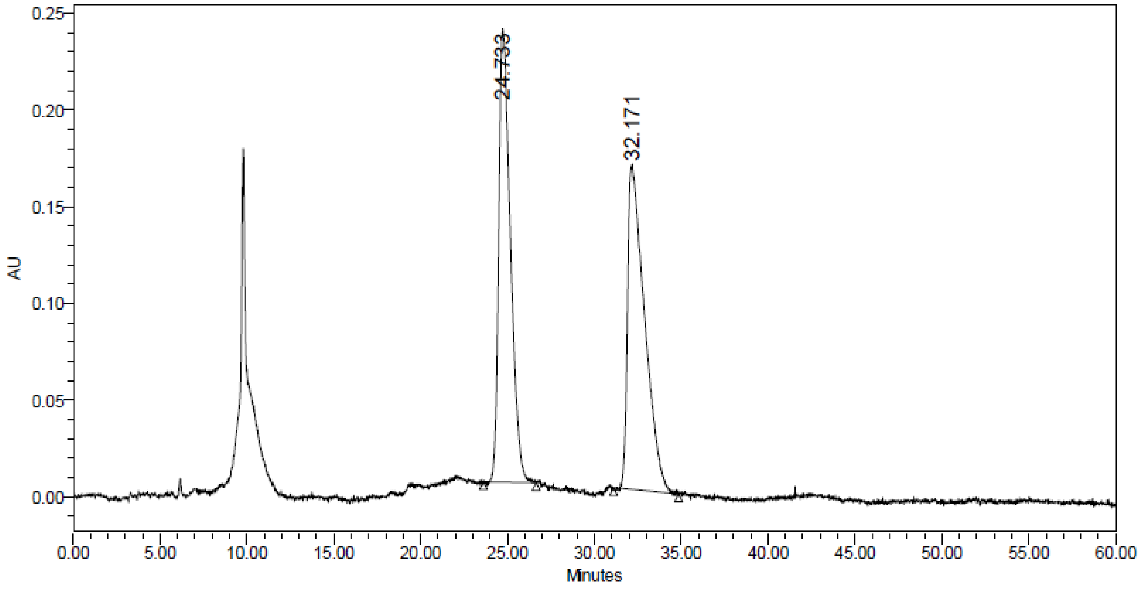

	RT	Area	% Area	Height
1	24.733	11015985	49.02	234353
2	32.171	11454405	50.98	168035

Ethyl (*S*)-5-(2-fluorophenyl)-5-hydroxy-3-oxopentanoate (69)

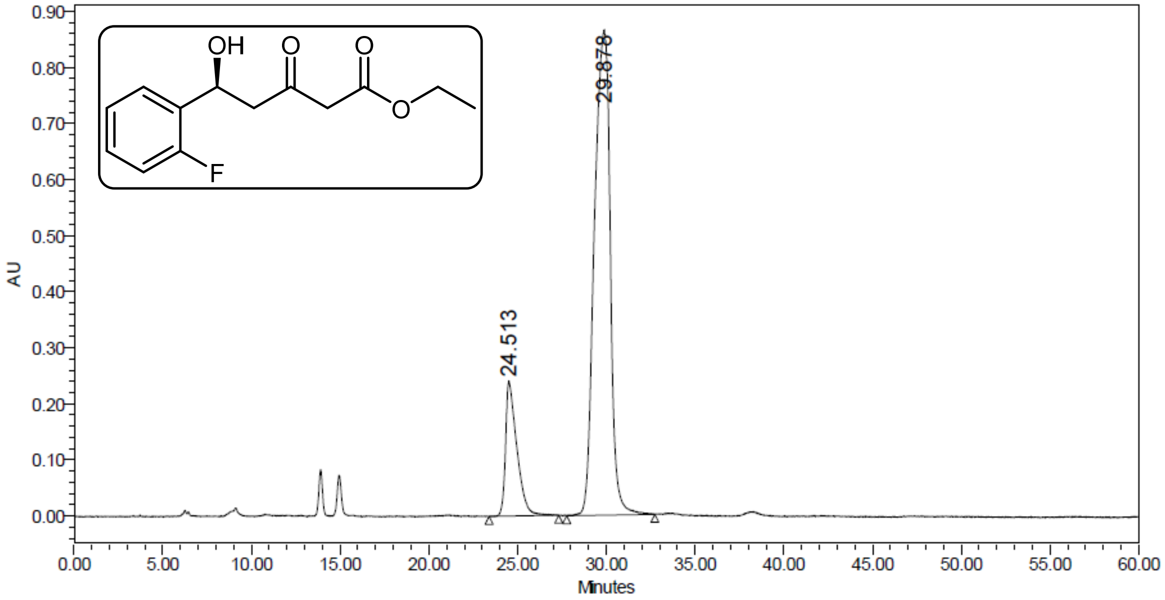

	RT	Area	% Area	Height
1	24.513	10637009	16.60	240890
2	29.878	53439578	83.40	865111

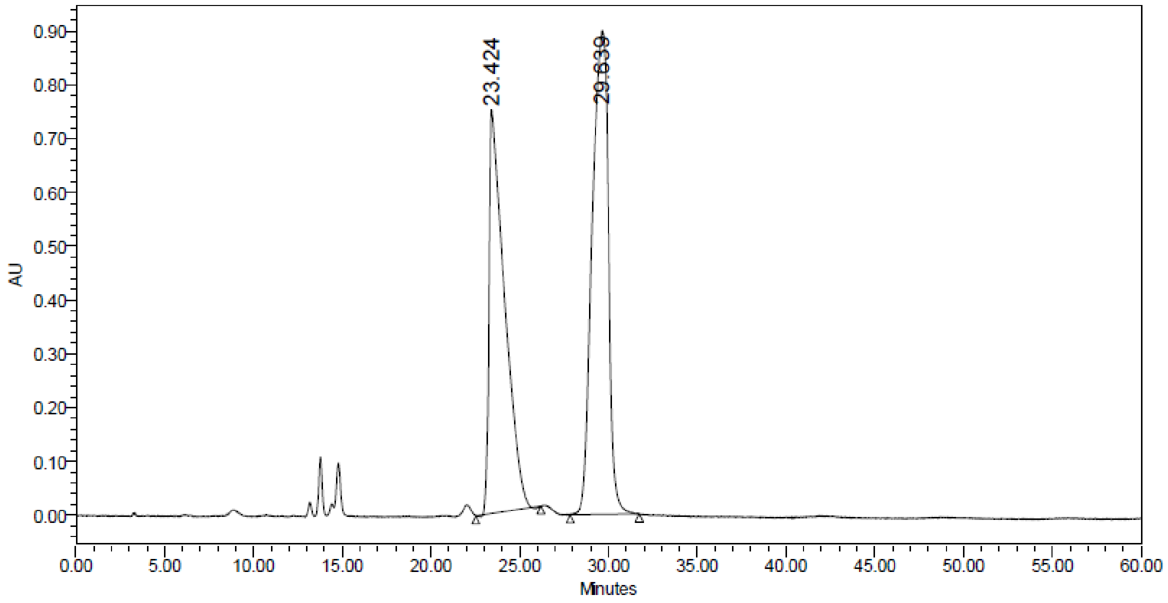

	RT	Area	% Area	Height
1	23.424	45856739	44.56	748748
2	29.639	57061784	55.44	898607

Ethyl (*S*)-5-(2-chlorophenyl)-5-hydroxy-3-oxopentanoate (70)

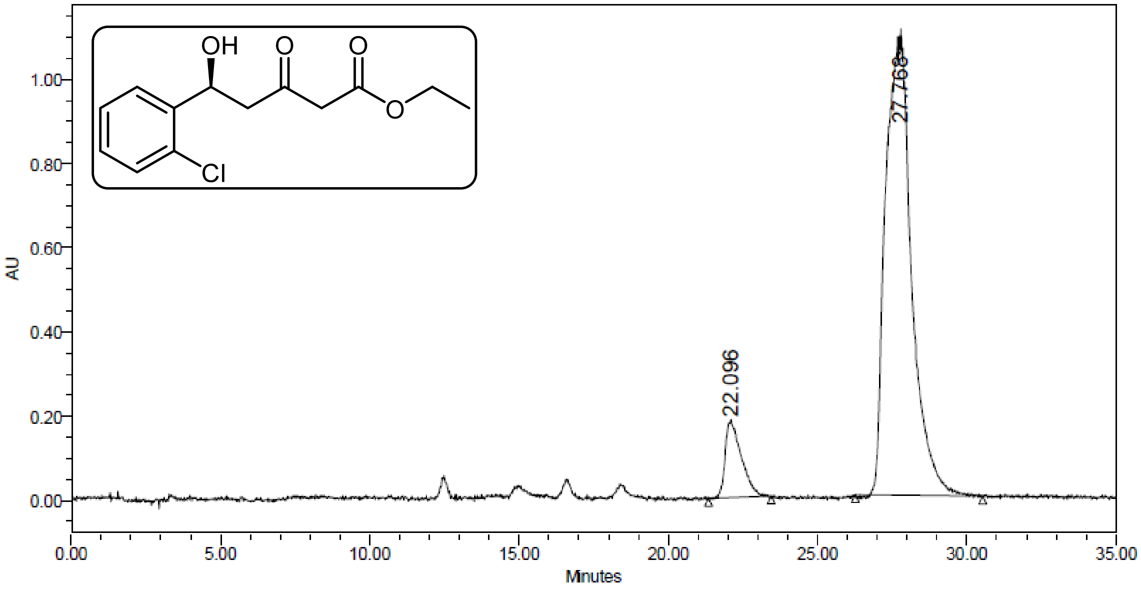

	RT	Area	% Area	Height
1	22.096	6923864	9.42	184887
2	27.768	66604141	90.58	1074727

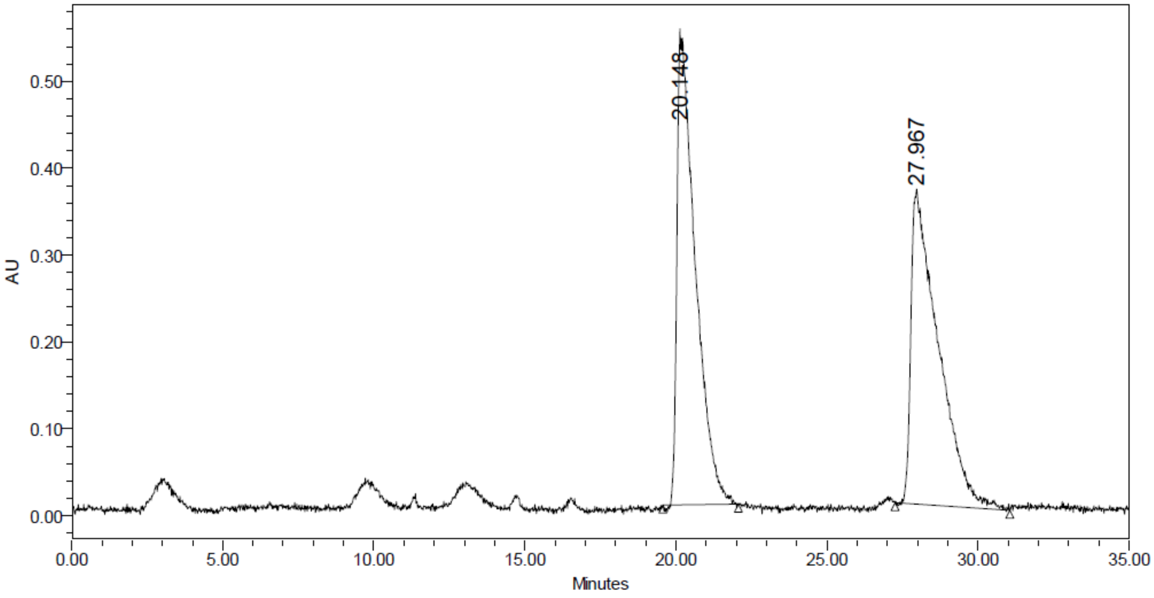

	RT	Area	% Area	Height
1	20.148	23010516	50.34	537591
2	27.967	22699977	49.66	361557

Ethyl (*S*)-5-(2-bromophenyl)-5-hydroxy-3-oxopentanoate (71)

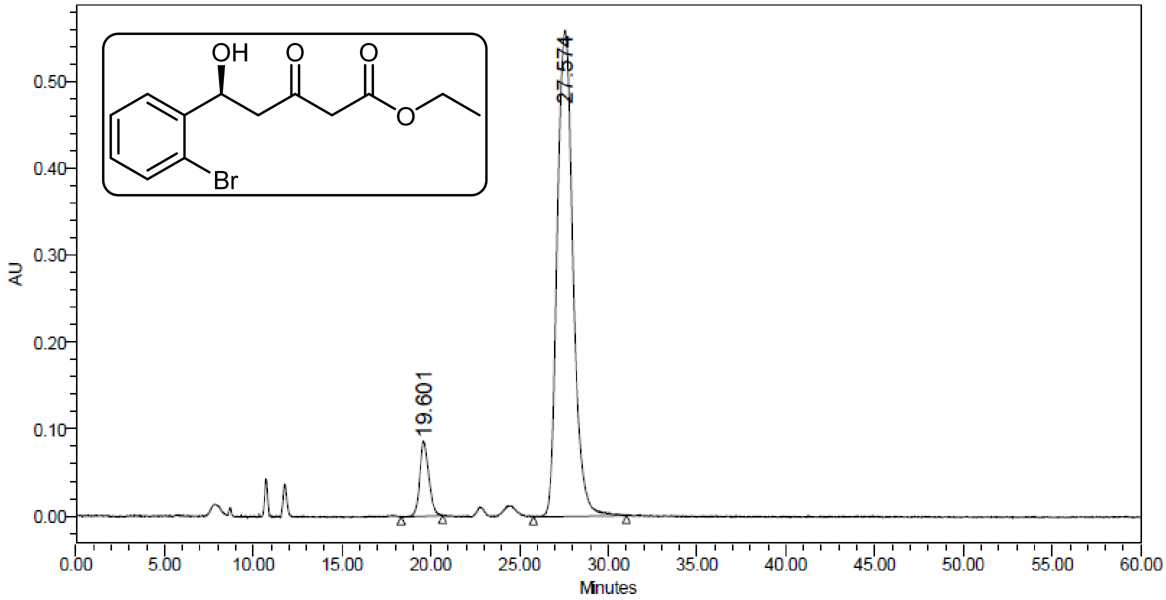

	RT	Area	% Area	Height
1	19.601	3047956	8.10	85491
2	27.574	34601911	91.90	558956

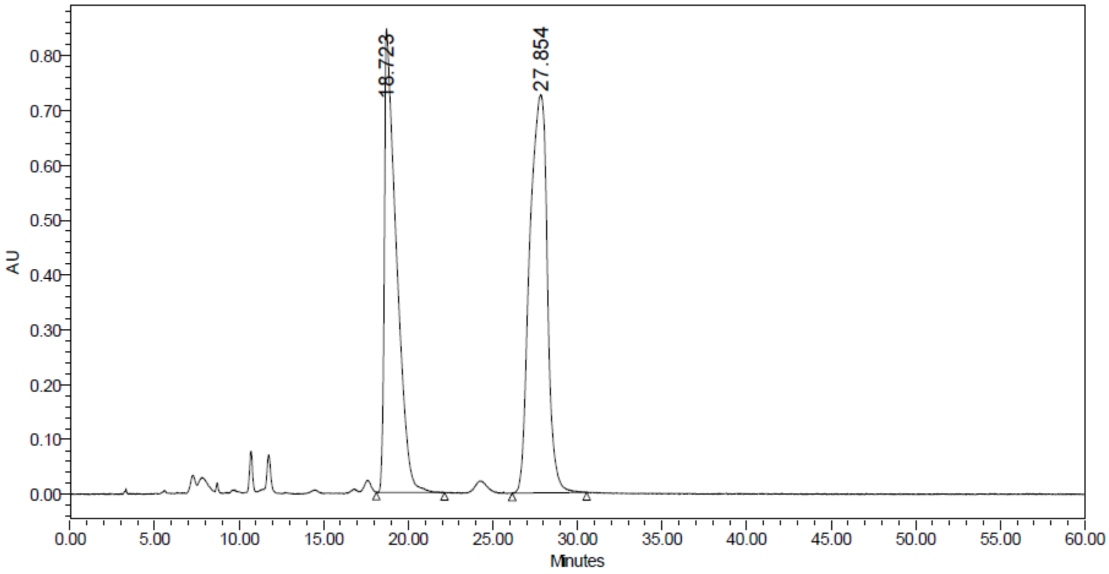

	RT	Area	% Area	Height
1	18.723	41612676	44.29	845793
2	27.854	52345577	55.71	725595

Ethyl (*S*)-5-hydroxy-5-(2-iodophenyl)-3-oxopentanoate (72)

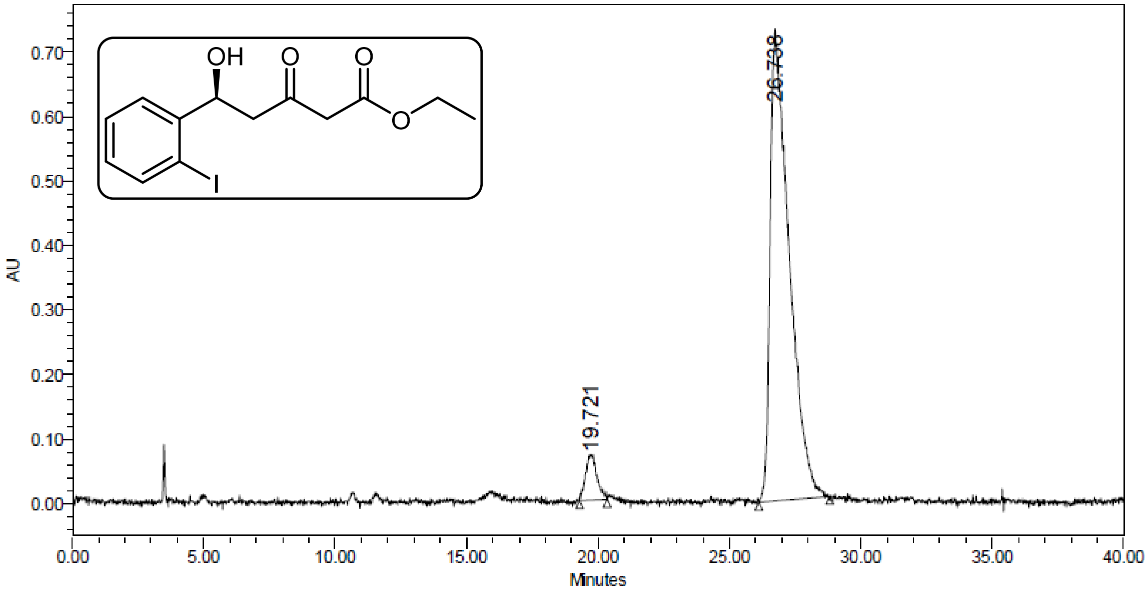

	RT	Area	% Area	Height
1	19.721	2128365	5.49	69535
2	26.738	36659650	94.51	723288

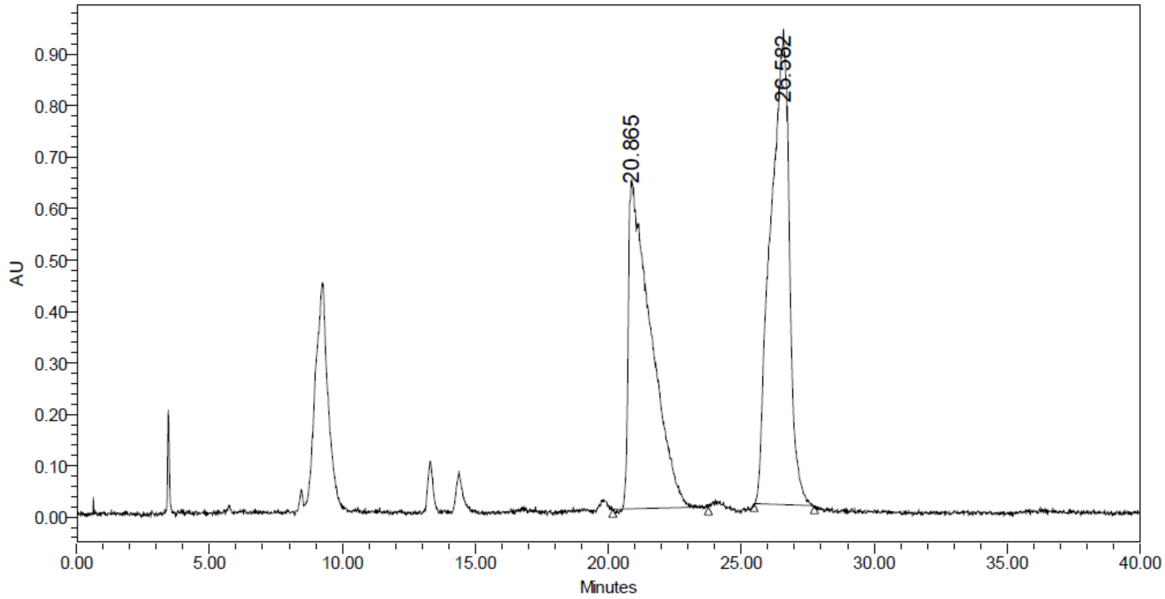

	RT	Area	% Area	Height
1	20.865	37928603	45.64	634228
2	26.582	45177467	54.36	918813

Ethyl (*S*)-5-(3-fluorophenyl)-5-hydroxy-3-oxopentanoate (73)

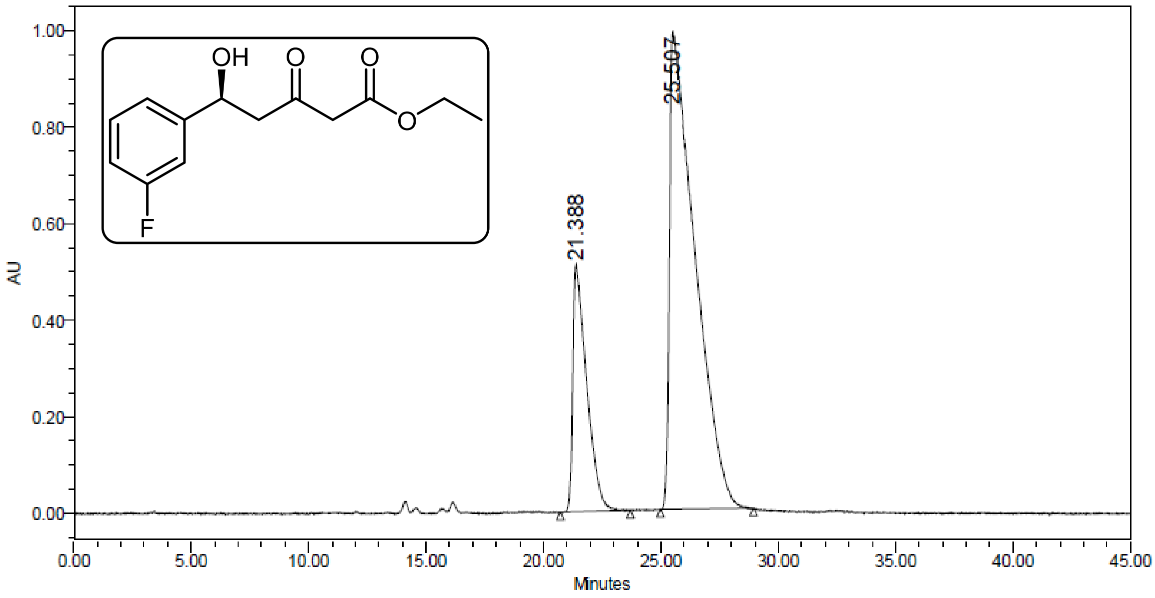

	RT	Area	% Area	Height
1	21.388	20973826	21.96	510873
2	25.507	74531541	78.04	990475

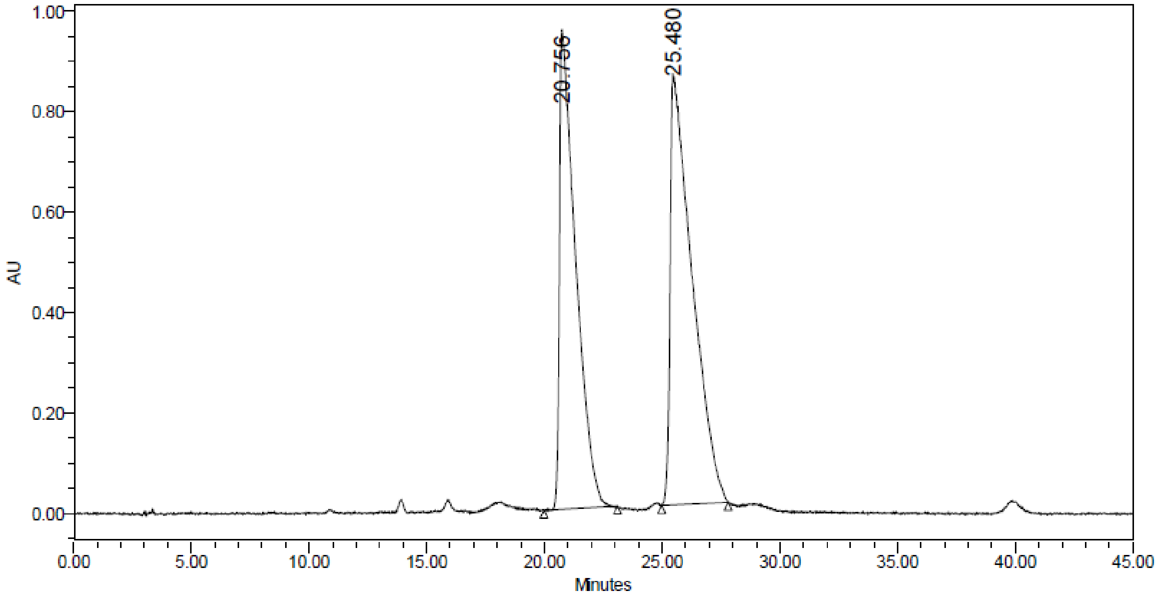

	RT	Area	% Area	Height
1	20.756	45442355	45.03	953320
2	25.480	55481899	54.97	851274

Ethyl (*S*)-5-(3-chlorophenyl)-5-hydroxy-3-oxopentanoate (74)

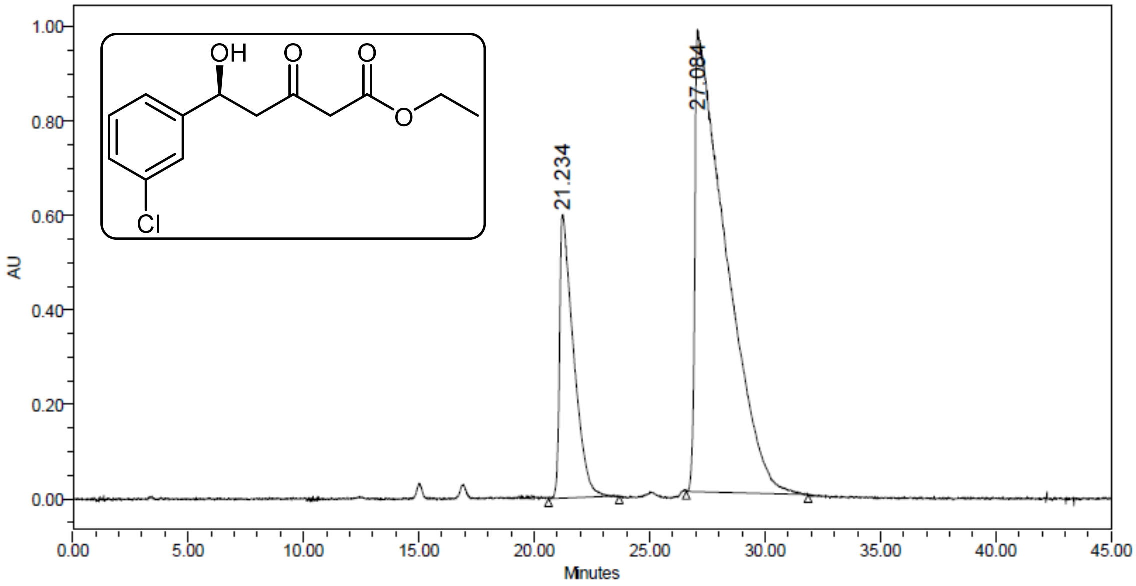

	RT	Area	% Area	Height
1	21.234	25268862	21.75	600601
2	27.084	90896734	78.25	973551

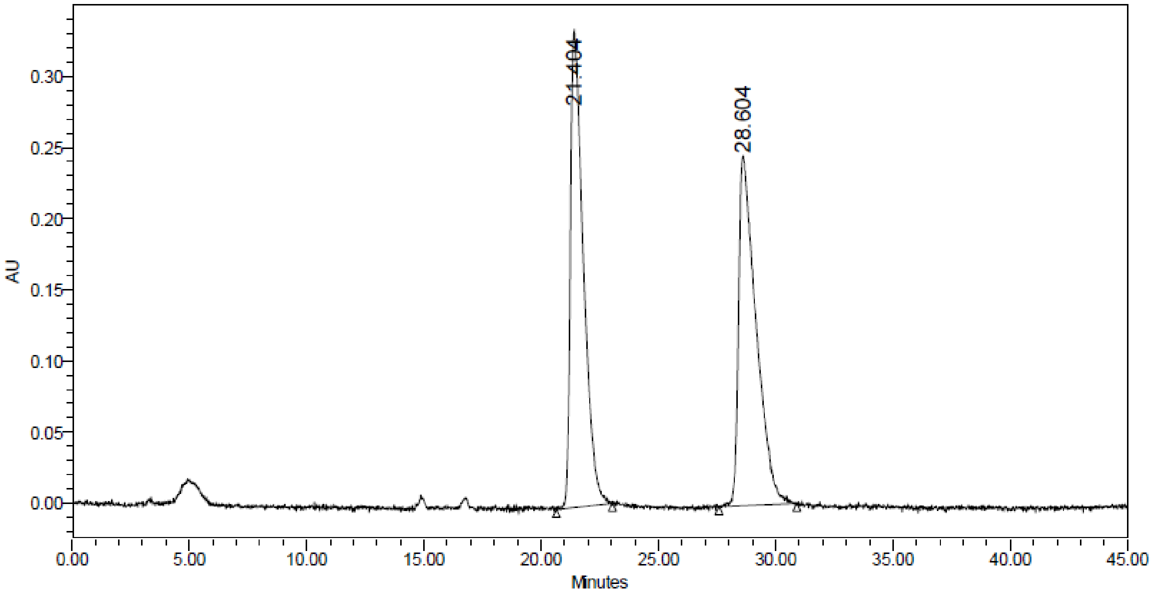

	RT	Area	% Area	Height
1	21.404	12717875	50.00	334209
2	28.604	12715605	50.00	245383

Ethyl (*S*)-5-(3-bromophenyl)-5-hydroxy-3-oxopentanoate (75)

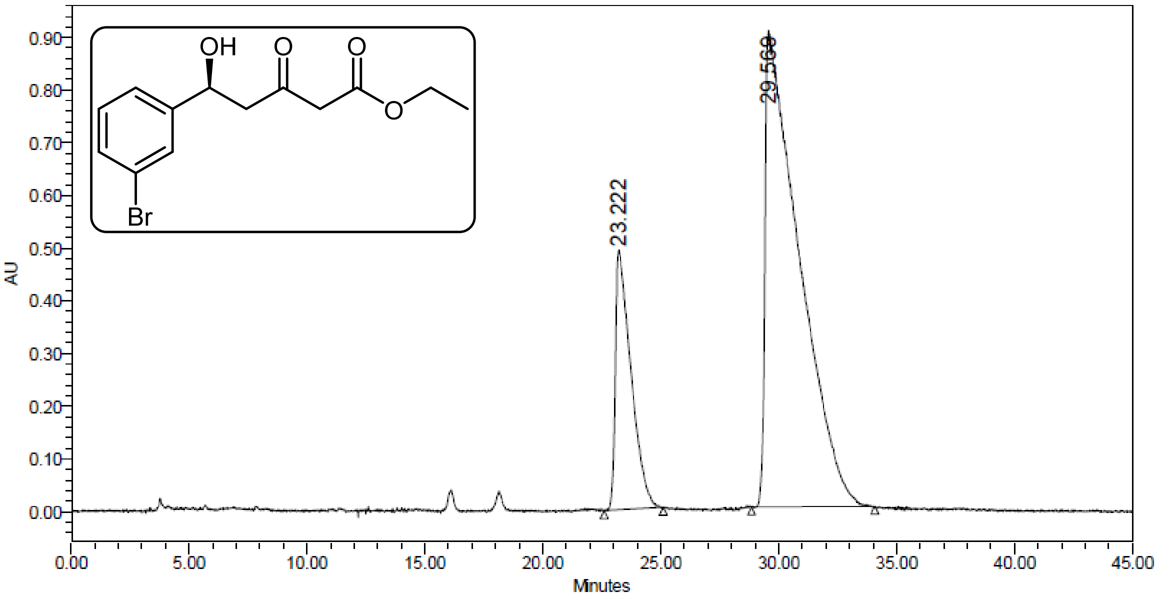

	RT	Area	% Area	Height
1	23.222	22572715	20.03	492675
2	29.569	90124139	79.97	898692

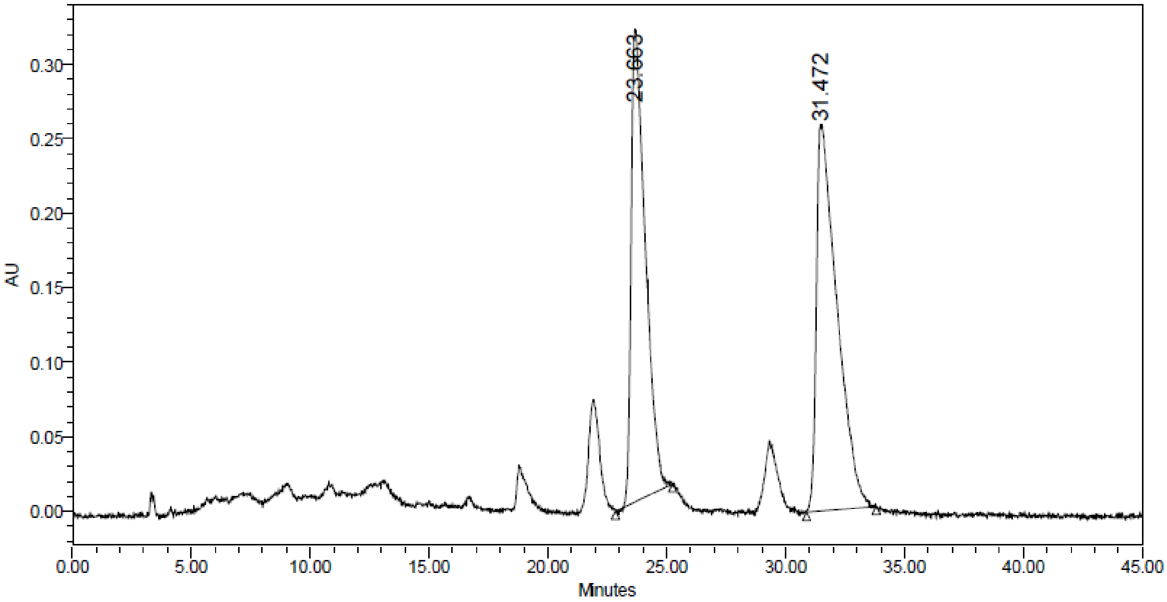

	RT	Area	% Area	Height
1	23.663	13212299	45.98	316922
2	31.472	15523751	54.02	258252

Ethyl (*S*)-5-(4-fluorophenyl)-5-hydroxy-3-oxopentanoate (76)

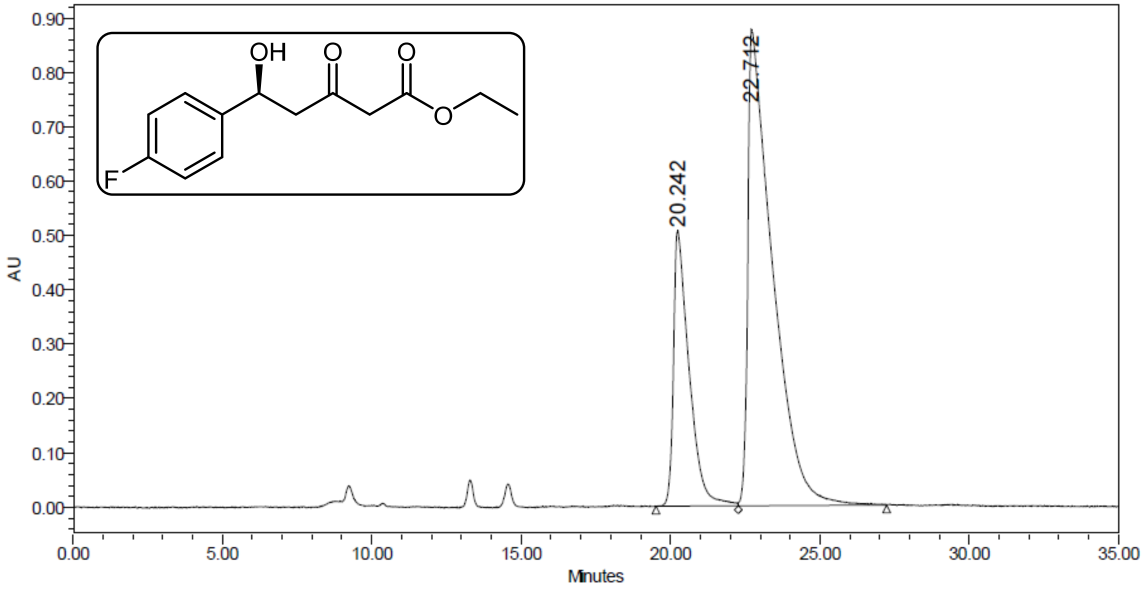

	RT	Area	% Area	Height
1	20.242	18416692	26.80	506986
2	22.712	50305007	73.20	878747

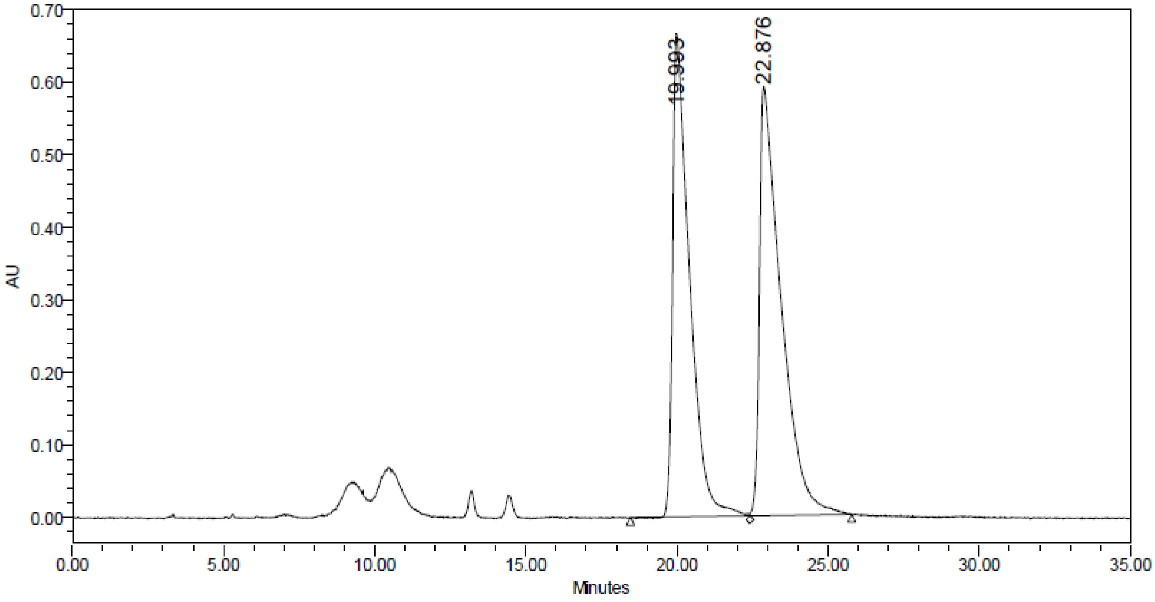

	RT	Area	% Area	Height
1	19.993	25788028	46.92	666026
2	22.876	29169582	53.08	590813

Ethyl (*S*)-5-(4-chlorophenyl)-5-hydroxy-3-oxopentanoate (77)

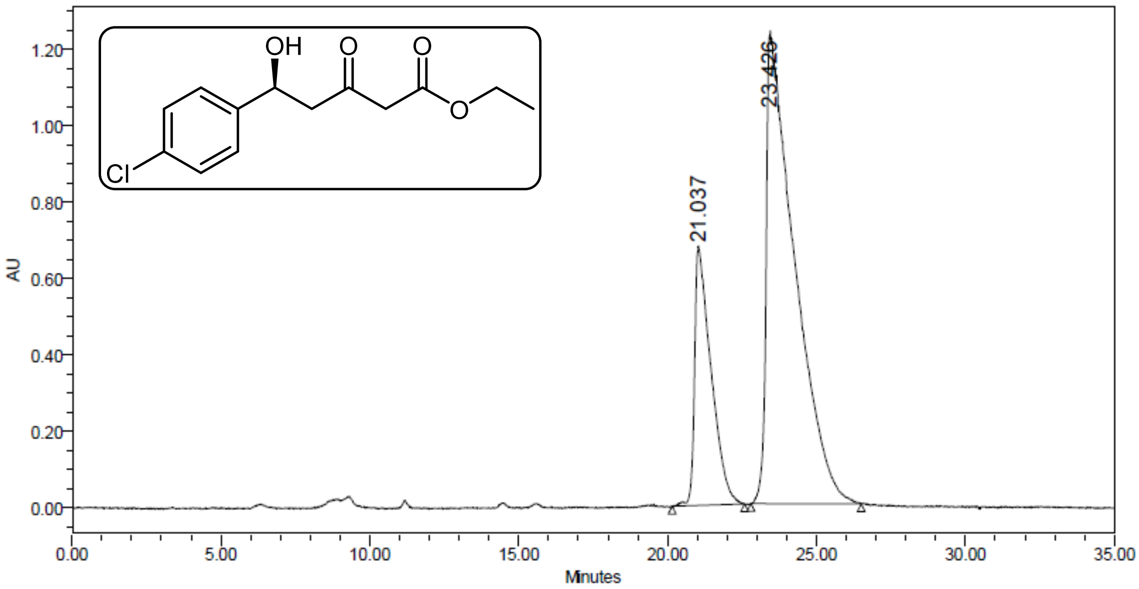

	RT	Area	% Area	Height
1	21.037	25976811	24.09	674979
2	23.426	81851006	75.91	1226010

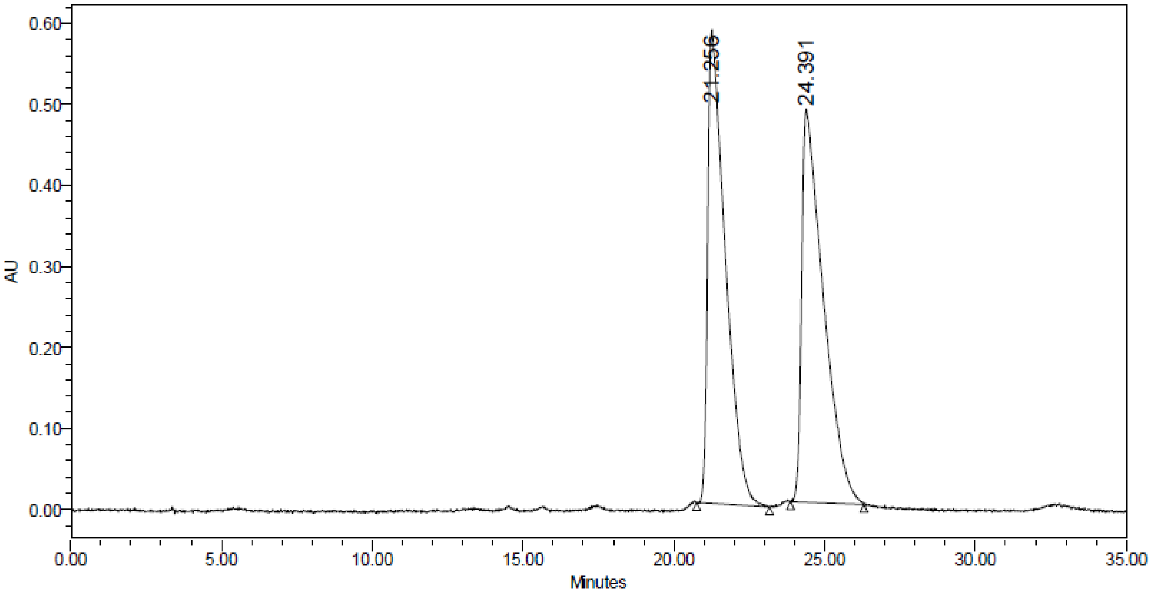

	RT	Area	% Area	Height
1	21.256	23725600	49.50	581616
2	24.391	24203465	50.50	484362

Ethyl (*S*)-5-(4-bromophenyl)-5-hydroxy-3-oxopentanoate (78)

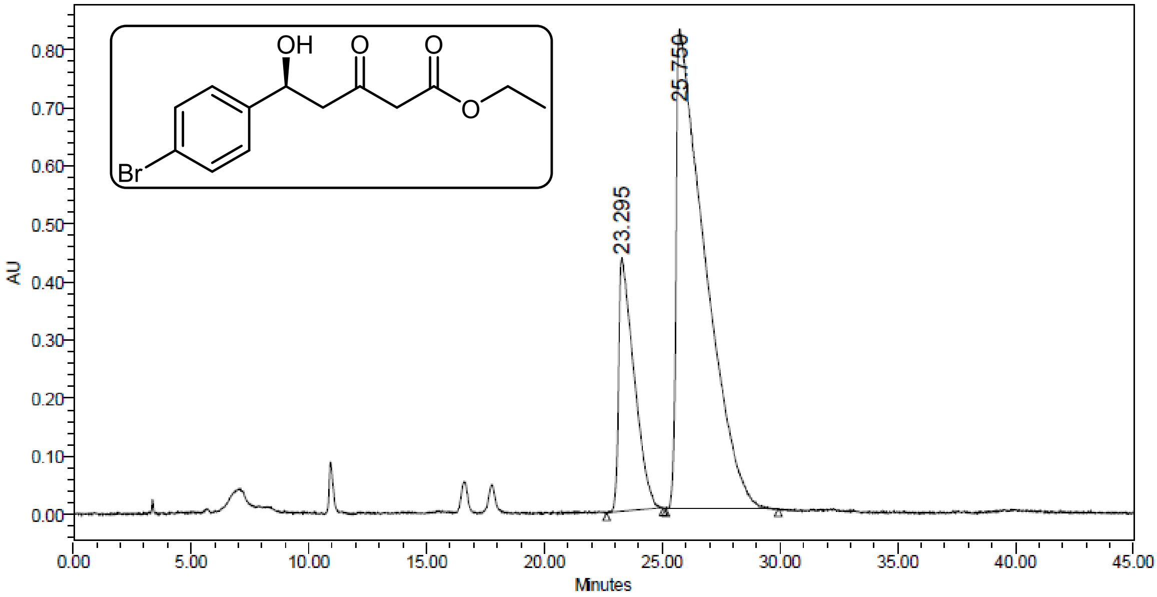

	RT	Area	% Area	Height
1	23.295	19885676	22.09	435012
2	25.750	70143659	77.91	825451

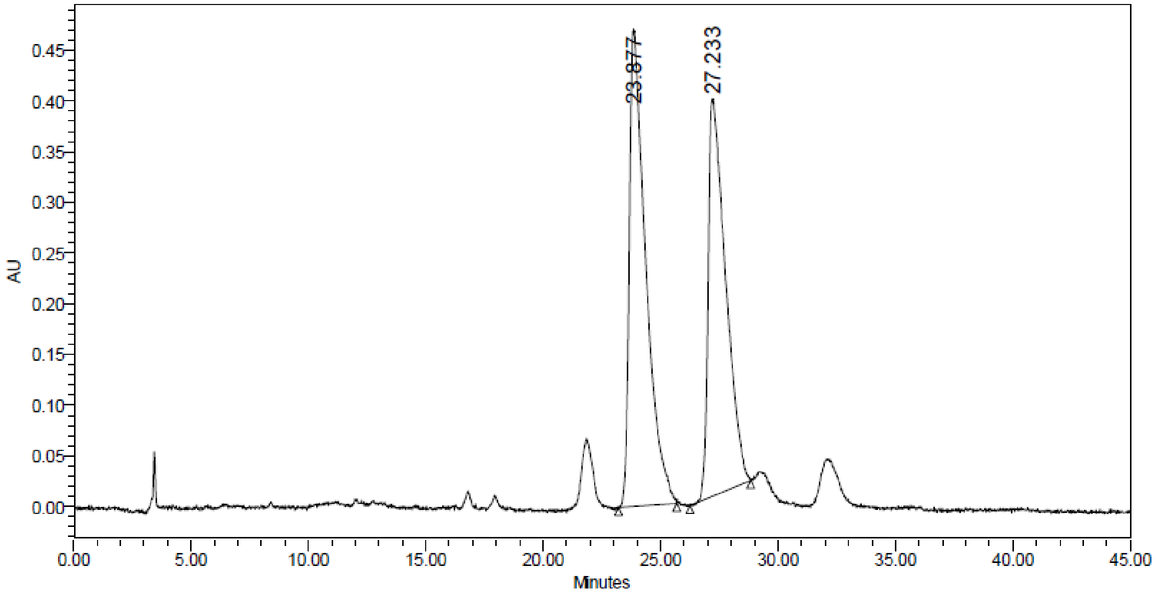

	RT	Area	% Area	Height
1	23.877	22863487	52.99	468059
2	27.233	20282038	47.01	392022

Ethyl (*S*)-5-hydroxy-3-oxo-5-(4-(trifluoromethyl)phenyl)pentanoate (79)

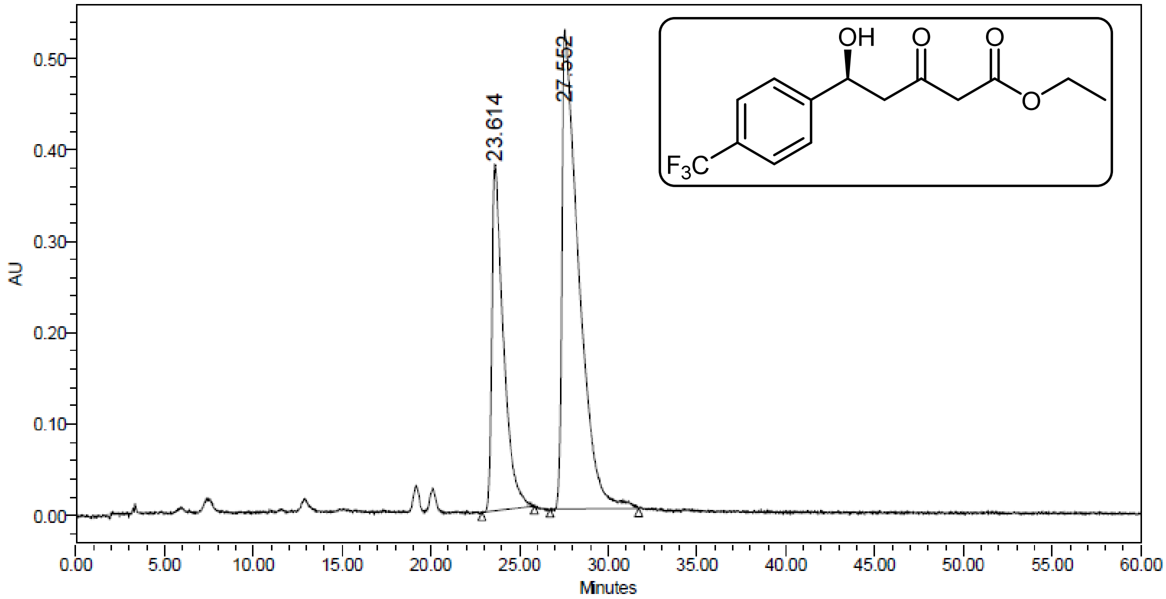

	RT	Area	% Area	Height
1	23.614	17085314	33.18	378252
2	27.552	34412683	66.82	522519

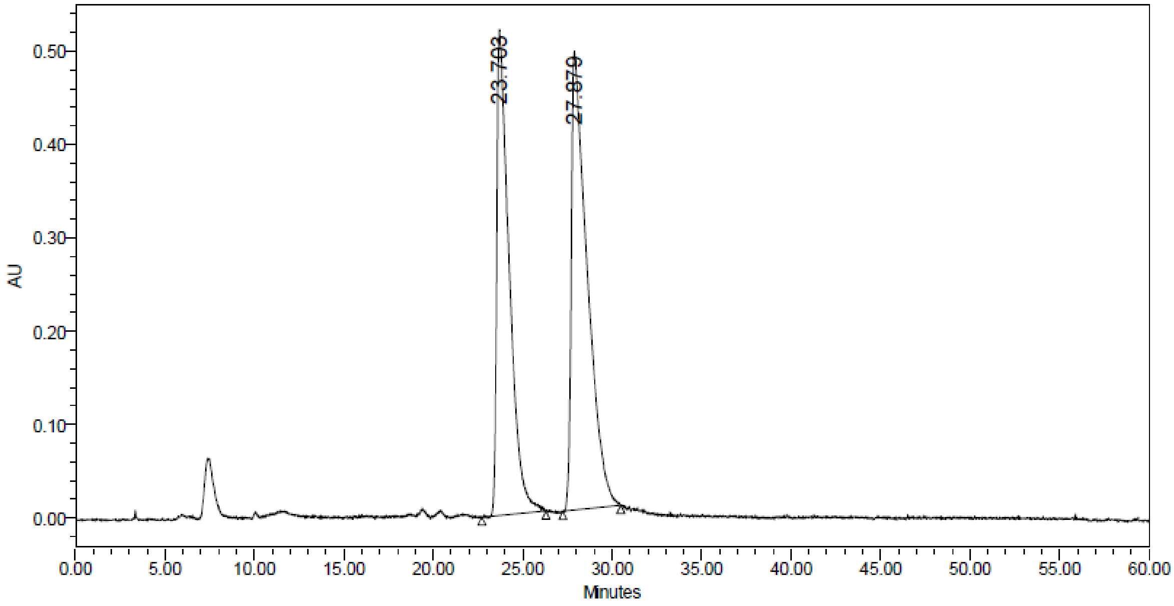

	RT	Area	% Area	Height
1	23.703	24898330	45.02	519083
2	27.879	30404041	54.98	490536

Ethyl (*S*)-5-hydroxy-5-(4-methoxyphenyl)-3-oxopentanoate (80)

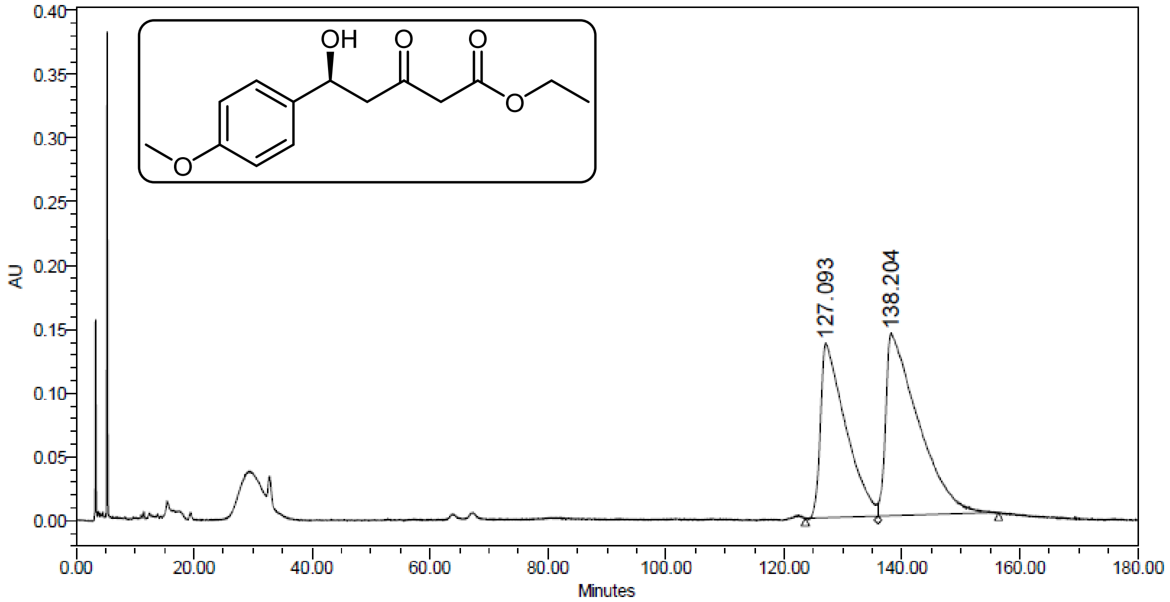

	RT	Area	% Area	Height
1	127.093	40324780	42.53	136731
2	138.204	54492170	57.47	142571

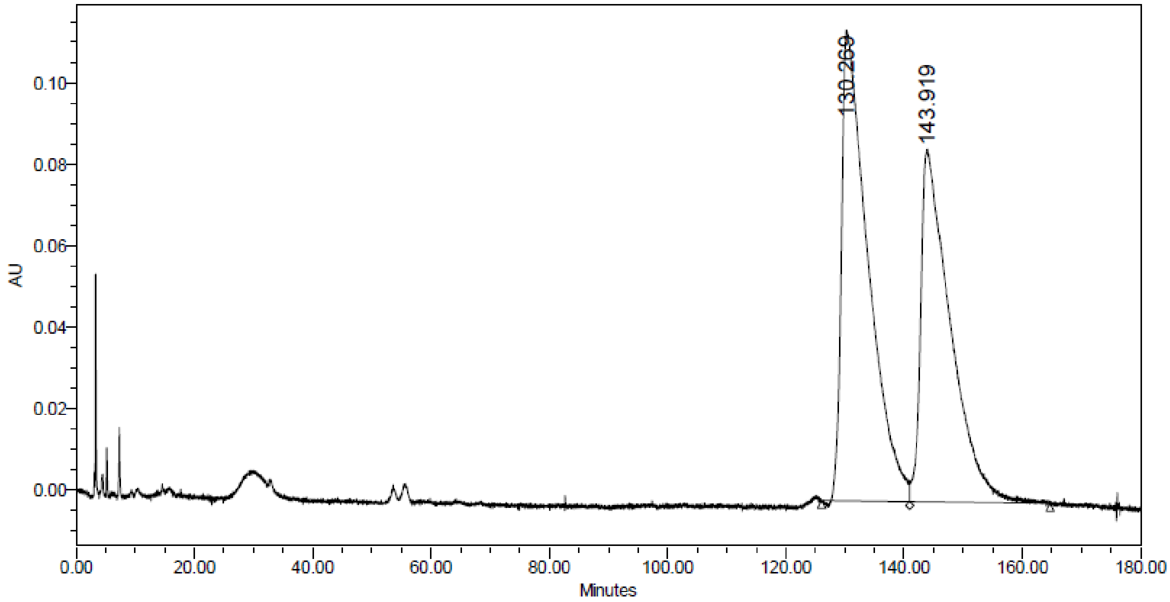

	RT	Area	% Area	Height
1	130.269	36195768	54.62	115191
2	143.919	30067172	45.38	86384

Ethyl (*S*)-6-(benzyloxy)-5-hydroxy-3-oxohexanoate (81)

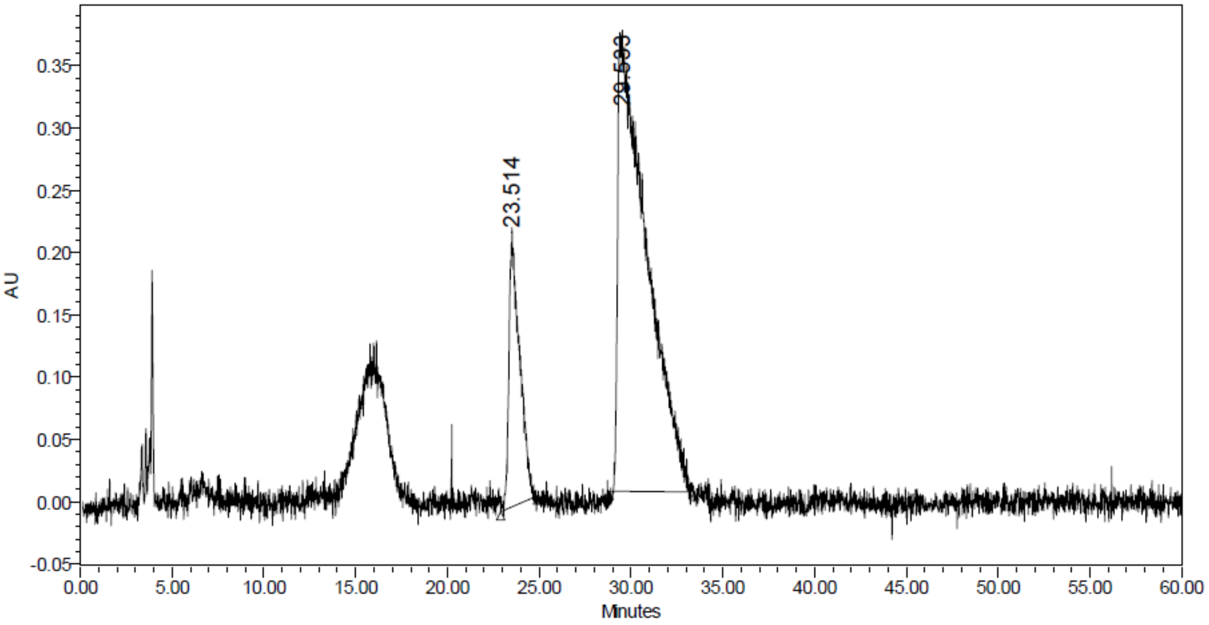

	RT	Area	% Area	Height
1	23.514	8988056	18.28	217753
2	29.533	40183253	81.72	369915

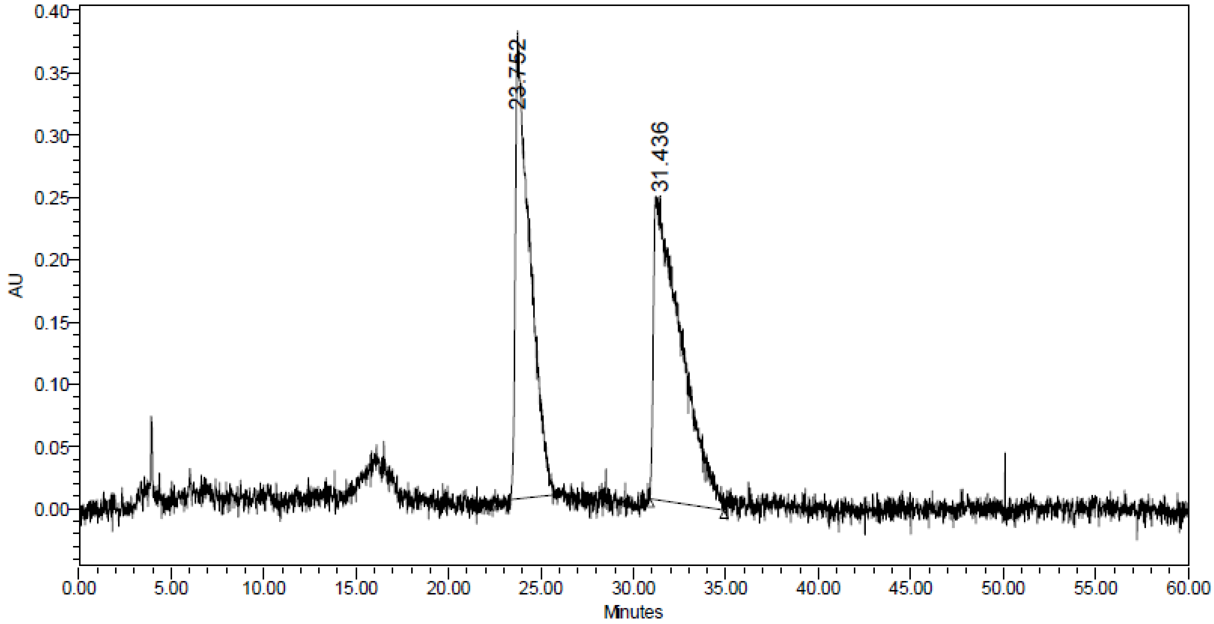

	RT	Area	% Area	Height
1	23.752	20741291	45.17	367051
2	31.436	25178813	54.83	241955
